# Genome-wide identification of sweet orange (*Citrus sinensis*) histone modification gene families and their expression analysis during the fruit development and fruit-blue mold infection process

**DOI:** 10.3389/fpls.2015.00607

**Published:** 2015-08-05

**Authors:** Jidi Xu, Haidan Xu, Yuanlong Liu, Xia Wang, Qiang Xu, Xiuxin Deng

**Affiliations:** Key Laboratory of Horticultural Plant Biology (Ministry of Education), Huazhong Agricultural UniversityWuhan, China

**Keywords:** histone modifications, *Citrus sinensis*, epigenetics, fruit development, blue mold

## Abstract

In eukaryotes, histone acetylation and methylation have been known to be involved in regulating diverse developmental processes and plant defense. These histone modification events are controlled by a series of histone modification gene families. To date, there is no study regarding genome-wide characterization of histone modification related genes in citrus species. Based on the two recent sequenced sweet orange genome databases, a total of 136 *CsHMs* (*Citrus sinensis* histone modification genes), including 47 *CsHMTs* (histone methyltransferase genes), 23 *CsHDMs* (histone demethylase genes), 50 *CsHATs* (histone acetyltransferase genes), and 16 *CsHDACs* (histone deacetylase genes) were identified. These genes were categorized to 11 gene families. A comprehensive analysis of these 11 gene families was performed with chromosome locations, phylogenetic comparison, gene structures, and conserved domain compositions of proteins. In order to gain an insight into the potential roles of these genes in citrus fruit development, 42 *CsHMs* with high mRNA abundance in fruit tissues were selected to further analyze their expression profiles at six stages of fruit development. Interestingly, a numbers of genes were expressed highly in flesh of ripening fruit and some of them showed the increasing expression levels along with the fruit development. Furthermore, we analyzed the expression patterns of all 136 *CsHMs* response to the infection of blue mold (*Penicillium digitatum*), which is the most devastating pathogen in citrus post-harvest process. The results indicated that 20 of them showed the strong alterations of their expression levels during the fruit-pathogen infection. In conclusion, this study presents a comprehensive analysis of the histone modification gene families in sweet orange and further elucidates their behaviors during the fruit development and the blue mold infection responses.

## Introduction

In eukaryotes, the dynamics of chromatin structure regulate DNA accessibility and DNA-templated processes, and affect various biological processes (Ho and Crabtree, 2010). Nucleosome is the basic unit of chromatin and it compacts DNA by nearly sevenfold with ∼146 bp of DNA wrapped around a histone octamer. The histone octamer is composed by two copies of H2A, H2B, H3 and H4 histone proteins. The histone tails are modified by dynamic post-translational modifications (PTMs) including methylation/demethylation, acetylation/deacetylation, and so on (Patel and Wang, 2013). Various histone modifications, which are termed as the “histone code,” collectively build up an enriched and complicated pattern of chromatin structure and powerful function modulations (Strahl and Allis, 2000). It is well reviewed that a series of gene families involved in the establishment of histone methylation/demethylation and acetylation/deacetylation (Berr et al., 2011).

Methylation of histone lysine residues is an important epigenetic regulation mechanism which can activate or silence gene expression. It is known that histone lysine methylation modifications (except the methylation of H3K79) are catalyzed by a series of histone methyltransferases (HMTs), which were mainly encoded by a family of SET DOMAIN GROUP genes (*SDGs*; Feng et al., 2002). The *SDG* family is divided into different classes according to the sequence similarities with the suppressor of variegation 3–9 [SU(VAR)3-9], enhancer of zeste [E(z)], trithorax (TRX), and absent, small, or homeotic disks 1 (ASH1). The functions of histone lysine methylation in plant biological processes are involved in floral organ development, flowering transition, shoot and root branching, endodormancy release, carotenoid biosynthesis, hormone regulation, thigmomorphogenesis, and fungal pathogens resistance (Dong et al., 2008; Cazzonelli et al., 2009, 2014; Berr et al., 2010a,b; Sui et al., 2012; Sun et al., 2012; Kim et al., 2013a; Saito et al., 2015). Moreover, histone methylation also occurs at arginine residues and histone arginine methylation is involved in many cellular processes including transcription, RNA processing and transport, signaling, subcellular transport and so on (Pahlich et al., 2006). Histone arginine methylation is controlled by a conserved protein family named protein arginine methyltransferases (PRMTs). Plant *PRMT* genes are involved in the regulations of several essential developmental processes, including vegetative growth, circadian cycle, flowering process, and response to ABA and high salinity (Ahmad and Cao, 2012). On the other hand, histone methylation can be directly erased through the action of histone demethylases (HDMs). So far, two types of HDMs have been identified: lysine-specific demethylase 1 (LSD1) and jumonji C (JmjC) domain containing proteins. LSD1 is an amine oxidase, which removes mono- and di-methyl groups from H3K4 residue. *Arabidopsis* has four genes encoding LSD1 [LDL1, LDL2, LDL3 and FLOWERING LOCUS D (FLD)] which can regulate the flowering time with partial redundancies (Jiang et al., 2007). The other type of HDMs is JmjC-domain protein family (JMJ famlily) which has been assigned to distinct groups including JmjC-domain-only group, JHDM1/FBX/KDM2, JMJD1/JHDM2/KDM3, JMJD2/KDM4, JARID/KDM5, and JMJD3/KDM6. Studies on *JMJs* in plant have uncovered their important roles in chromatin regulation and plant development, including flowering time, floral organ development, female gametophyte development, BR signaling and circadian regulation (Chen et al., 2011).

Histone acetylation and deacetylation underlie a mechanism for reversibly modulating chromatin structure and transcriptional regulation (Tian et al., 2005). The homeostatic balance of histone acetylation is maintained by two types of antagonistic proteins: histone acetylases (HATs) and histone deacetylases (HDACs). So far, plant HATs have been distinctly divided into four groups including: (1) HAG group contains GCN5-, ELP3-, and HAT1-like histone acetylases; (2) HAM group featured by a MOZ-YBF2 (MYST) domain; (3) HAC group is similar to p300/CREB-binding protein (CBP) co-activator family in animals; (4) HAF group is related to mammalian TAF_II_250 (TATA binding protein-associated factors; Pandey et al., 2002). Genes encoding HATs have been widely reported in the regulations of developmental transitions, responses to environmental signals and integrations of stress hormone signals (Tian et al., 2005; Sheldon et al., 2006; Chen and Tian, 2007). Plant *HDACs* have been classified into three families including RPD3/HDA1 superfamily (*HDA*), Silent Information Regulator 2 (*SRT*) and HD2 (*HDT*) families (Hollender and Liu, 2008). Currently, studies have revealed key roles of plant *HDACs* in regulating plant vegetative and reproductive development, stress responses, gene silencing, as well as cell death and cycle (Ma et al., 2013; Wang et al., 2014). Overall, these gene families involved in histone modifications cooperatively alter the chromatin structures and performances of nucleosomes in order to specifically control gene expression. Moreover, in spite of involvements of these genes in developmental regulations, a growing body of studies has been revealed their crucial roles in abiotic stresses and plant immunity (Berr et al., 2010a; Kim et al., 2010; Luo et al., 2012).

Citrus is an important and widely grown fruit crop with richness of nutritional components such as carotenoids and vitamin C. Its fruit development and ripening process shows a single sigmoid curve including two stages of slow growth with a period of rapid growth in between (Bain, 1958). After the fruit ripening, most of citrus fruits have been proceeded to the post-harvest storage. Blue mold (*Penicillium digitatum*) is the most devastating pathogen in citrus fruit post-harvest process and responsible for nearly 90% of production losses during fruit post-harvest handling (Macarisin et al., 2007). Although *HM* genes had been investigated during the fruit development process in tomato (Aiese Cigliano et al., 2013) and grape (Aquea et al., 2011), as well as the plant–pathogen response in *Arabidopsis* (Alvarez et al., 2010; Berr et al., 2010a), little is known regarding the functions of *HMs* in citrus.

Given the critical roles of plant *HMs* in regulations of fruit development and pathogen responses, it is expected that they are also involved in citrus fruit development and fruit-blue mold infection. In this study, 136 *CsHMs*, belonging to 11 families were identified in sweet orange. Then, genomic organization, phylogenetic relationship, domain architecture, and gene structure of these genes were comprehensively analyzed. Additionally, expression profiles of *CsHMs* were analyzed in six stages of fruit development and four periods of blue mold infection. Such a comprehensive analysis of these *CsHMs* will provide fundamental to understanding their diverse roles in citrus development and be useful for future functional genomic studies on regulations of histone modifications in citrus.

## Materials and Methods

### Identification of *CsHM* Families

The HMM files containing conserved domain of each *HM* families (HMTs: SDG-PF00856, PRMT-PF05185; HDMs: HDMA-PF04433, JMJ-PF02373; HATs: HAG-PF00583, HAM-PF01853, HAC-PF08214, HAF-PF09247; HDACs: HDA-PF00850, SRT-PF02146) were downloaded from Pfam protein database^1^. These HMM files were used as a query to search the two sweet orange genome databases^2^ [Orange genome Annotation Project (Xu et al., 2013); Sweet Orange Genome Project 2010^3^ (Wu et al., 2014)] using HMMER 3.0 software (HMMER 3.0^4^) with the default parameters. In order to obtain the complete catalog of *CsHMs*, the output results from two genomes were combined and filtrated the redundant sequences. For CsHDTs, AtHDT1 (At3g44750), AtHDT2 (At5g22650), AtHDT3 (At5g03740), and AtHDT4 (At2g27840) from *Arabidopsis thaliana* were used to perform a Blastp algorithm from sweet orange genome^2^ and two sequences named as CsHDT1, CsHDT2 were obtained. The final ID numbers and DNA sequences of *CsHMs* are listed in Supplementary Table S1 and Data Sheet 1, respectively. The ID numbers with red fonts were the additional predicted genes from the second sweet orange genome (Wu et al., 2014).

### Genomic Organization of *CsHMs*

To determine the physical location of *HMs*, the MapChart software (Voorrips, 2002) was applied to locate the *CsHMs* on sweet orange chromosomes according to their positions given in the genome database (Xu et al., 2013).

### Analysis of Domain Compositions and Gene Structures

To investigate the domain compositions of *CsHMs*, the complete amino acid sequences of these genes were subjected to SMART website, including outlier homologs and PFAM domains. The genomic DNA sequences and corresponding CDS sequences of *CsHMs* were submitted to Gene Structure Display Server (GSDS^5^) website to visualize the gene structures.

### Phylogenetic Analysis

The HM protein sequences from *Arabidopsis*, rice and maize were collected from ChromDB database^6^. Each HM family including citrus HMs was aligned with ClustalW program. The generated files were subjected to phylogenic analysis by using MEGA 5.05 program^7^ with Neighbor-Joining method. The phylogenic trees were constructed with the following settings: pairwise deletion for sequences analysis, poisson model for substitution, and bootstrap test of 1000 replicates for internal branch reliability. For SDG and HAG families, the conserved domain sequences of SET and AT1 identified in citrus together with the domain sequences from *Arabidopsis*, rice and maize were used for tree constructions, respectively.

### Plant Materials and Blue Mold Infection

To analyze the expression patterns of *HMs* during the fruit development, fruit samples were collected from the adult plants of sweet orange (*Citrus sinensis* [L.] Osbeck), cultivated at the Institute of Citrus Research located in Guilin, Guangxi Province, China. Fruit samples with three independent repeats were collected from different position and orientation of six different trees. The fruit samples were continuously collected from July to December in 2011 as six fruit developmental stages, which were 90, 120, 150, 180, 210, and 240 days after flowering (daf), respectively. The peel and flesh tissues were separated from sampled fruits, and then immediately frozen in liquid nitrogen and kept at -80°C until further analysis.

Sweet orange fruits were used as the materials for the investigation of fruit-blue mold infection. Mature fruits were treated with 2% NaClO for 2 min and washed with distilled water for three times. A uniform lesion (5 mm wide, 3 mm deep) was made at the equator of the fruit using a sterile nail. An aliquot of 20 μL suspension of *P. digitatum* at 1 × 10^6^ spore mL^-1^ was inoculated into each wound site. After the inoculation, fruits were incubated in a storage chamber with 95% relative humidity at 25°C temperature for 6, 24, and 48 h to collect the samples. An aliquot of 20 μL double distilled water was inoculated into the fruits as the control (CT). 10 mm of peel around the wound was collected and immediately frozen in liquid nitrogen and kept at -80°C for RNA extraction. Each of the inoculation experiment was performed with the three replications.

### Expression Analysis of *CsHMs*

To investigate the expression patterns of all *CsHMs* in different citrus tissues, the normalized RPKM (reads per kilobase per million mapped reads) values of these genes were extracted from the dataset of the *Citrus sinensis* Annotation Project (CAP) and visualized by the heat maps with transformed log_10_ values using MeV 4.7 software (Saeed et al., 2006). In order to gain an insight of their roles in citrus fruit development, genes whose RPKM values were higher than 5.0 in fruit tissues were selected to further analyze their expression profiles during the six stages of fruit development using real-time PCR. Total RNA was extracted from the peel and flesh samples of citrus fruits according to the previous description (Liu et al., 2006). First strand cDNA was synthesized from 1.5 μg of total RNA using the ReverAid first strand cDNA synthesis KIT (Fermentas). Real-time PCR primer pairs were designed by Primer Express software (Applied Biosystems, Foster City, CA, USA) and their sequences were listed in Supplementary Table S2. The primers were tested to ensure amplification of single discrete bands with no primer-dimers. The primers were diluted in Power SYBR^®^ Green PCR Master Mix (Applied Biosystems) and the amplification mixture volume was 10 μL per reaction. Reaction conditions were an initial incubation for 2 min at 50 and 95°C for 1 min, and then followed by 40 cycles of 95°C/15 s and 60°C/1 min. Reactions were run on a 7900 HT Fast Real-Time PCR System with 384-Well Block Module (Applied Biosystems). The β*-actin* gene was used as an endogenous control and comparative Ct method (2^-ΔΔCt^) was adopted to calculate the expression data (Liu et al., 2007). The expression levels of 90 daf flesh or peel were used as the calibrator for the relative expression analysis. Expression analysis of all *CsHMs* response to blue mold infection was performed with real-time PCR. The expression levels of control were used as the calibrator for the analysis. The heat maps and hierarchical clustering of gene expression data were visualized in MeV 4.7 software. Genes with fold change (log_2_ value) higher than 1.0 or lower than -1.0 were selected and their expression profiles were shown in Supplementary Figure S2. SPSS software was applied to the statistical analysis of these data in the present study.

## Results

### Identification of *HMs* in Sweet Orange Genome

In this study, a total of 136 *CsHMs* were identified in sweet orange genome, including 47 histone methyltransferase genes (*CsHMTs*), 23 histone demethylase genes (*CsHDMs*), 50 histone acetylase genes (*CsHATs*), and 16 histone deacetylase genes (*CsHDACs*). The 47 *CsHMTs* had 40 *CsSDGs* and 7 *CsPRMTs* in sweet orange (**Table 1**). The number of *CsSDG* family was closed to that in *Arabidopsis* (41 members), rice (37; ChromDB database), tomato (43; Aiese Cigliano et al., 2013), and grape (33; Aquea et al., 2011). The 23 *CsHDMs* were composed by three citrus HDMA histone demethylase genes (*CsHDMAs*) and 20 JMJ genes (*CsJMJs*). The number of *CsJMJs* was also similar to that in *Arabidopsis* (21 members), rice (20; ChromDB database) and tomato (20; Aiese Cigliano et al., 2013). The 50 identified *CsHATs* were classified to 45 *CsHAGs*, one *CsHAM*, two *CsHACs*, and two *CsHAFs* in sweet orange. Much more *HAG* members were obtained in sweet orange genome compared with *Arabidopsis* (three members), rice (3) and maize (4). Only one MYST histone acetyltransferase gene (*CsHAM1*) encoded a 449 amino acid protein was identified in sweet orange. Two *CsHACs* belonging to the *HAC* group were identified and two TAF_II_250-like genes named as *CsHAF1* and *CsHAF2* were obtained in sweet orange. In addition, the 16 *CsHDACs* have nine *CsHDAs*, five *CsSRTs*, and two *CsHDTs* (**Table 1**). All of gene IDs was listed in Supplementary Table S1.

**Table 1:** Gene numbers of each histone modification families in sweet orange.

Types	Family	Gene numbers
*HMTs* (Histone methylation)	*SDGs*	40
	*PRMTs*	7
*HDMs* (Histone demethylation)	*HDMAs*	3
	*JMJs*	20
*HATs* (Histone acetylation)	*HAGs*	45
	*HAMs*	1
	*HACs*	2
	*HAFs*	2
*HDACs* (Histone deacetylation)	*HDAs*	9
	*SRTs*	5
	*HDTs*	2

### Chromosomal Distribution of *CsHMs*

#### CsHMTs/CsHDMs

**FIGURE 1:**
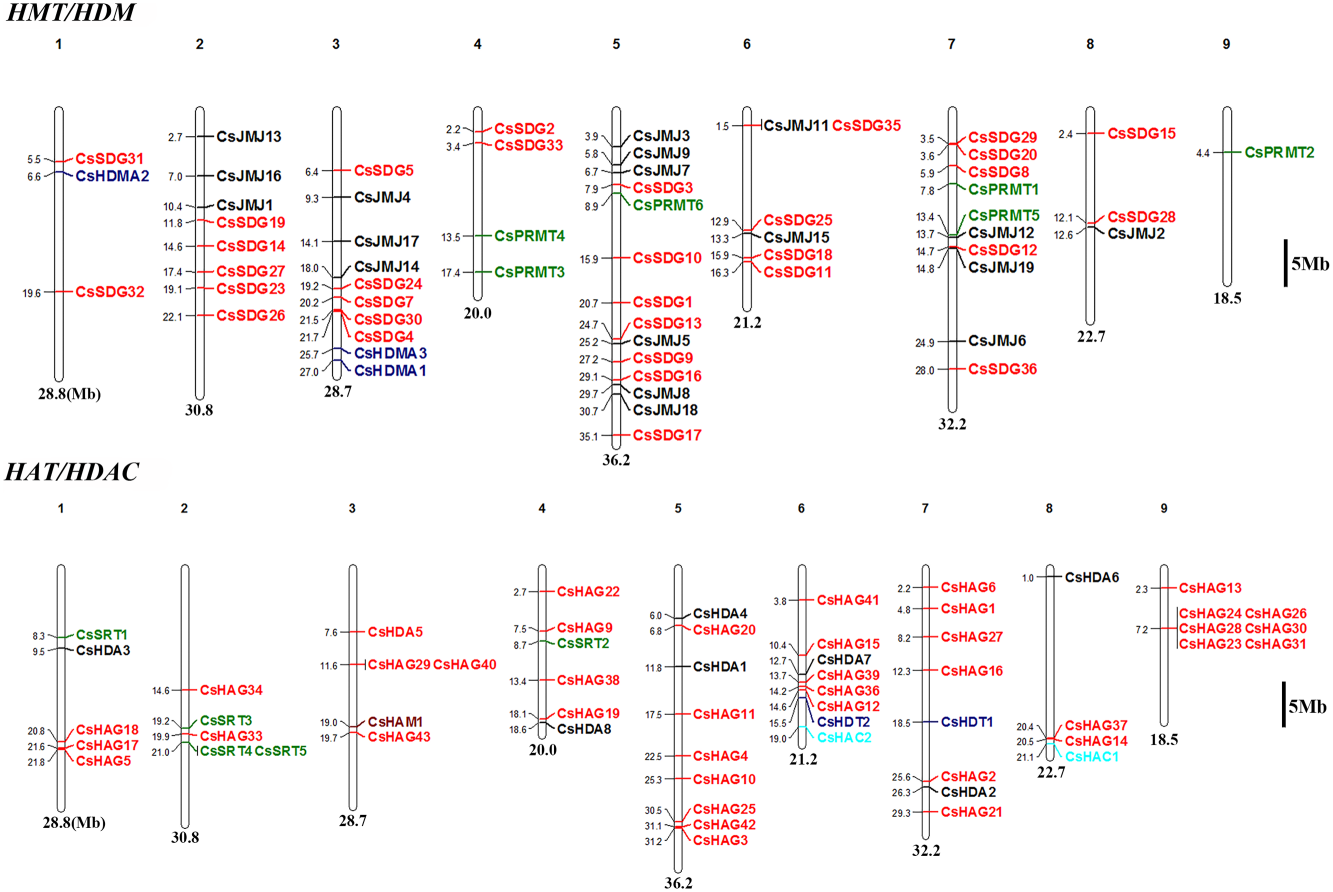
**Chromosomal localization of *CsHM* genes**.

The chromosomal locations of *CsHMs* were demonstrated on sweet orange chromosome available at CAP^8^. The members of *CsSDG* family were widely distributed in eight chromosomes with no distribution in the ninth chromosome (**Figure 1**). The largest number of *CsSDGs* was located on chromosome 5 (seven *CsSDGs*). However, eight genes including *CsSDG6*, *21*, *22*, *34*, *37*, *38*, *39*, and *40* were not determined because the physical map of sweet orange was incomplete. *CsPRMTs* were distributed at chromosomes 4, 5, 7, and 9 (**Figure 1**). As regard *CsHDMAs*, *CsHDMA2* was located in chromosome, while *CsHDMA1* and *CsHDMA3* were closely located in chromosome 3, suggesting the occurrence of tandem duplication. *CsJMJs* were widely distributed at chromosomes 2, 3, 5, 6, 7, and 8 and six of *CsJMJs* were located in chromosome 5.

#### CsHATs/CsHDACs

As shown in **Figure 1**, lots of *CsHAGs* displayed the close locations from each other. This might imply that the tandem duplication events occurred in this gene family. The citrus GCN5 (*CsHAG25*) and ELP3 gene (*CsHAG20*) were located in chromosome 5, while citrus HAT1 gene (*CsHAG38*) was in chromosome 4. Members of *CsHDAs* were distributed at chromosomes 1, 4, 5, 6, 7, and 8. Five *CsSRTs* named as *CsSRT1*-*5* were located at chromosomes 1, 2, and 4. Two *CsHDTs* named as *CsHDT1* and *CsHDT2* were located at chromosomes 7 and 6, respectively.

### Phylogenetic Analysis, Conserved Domains, and Exon/Intron Organizations of *CsHMs*

To explore the phylogenetic relationships among CsHM proteins and group them within the established classes, the predicted amino acid sequences of each *HM* family from various species were aligned and phylogenetic trees were further constructed. Furthermore, the gene structures of all *CsHMs* and the domain compositions of their coding proteins were analyzed.

#### CsHMTs

**FIGURE 2:**
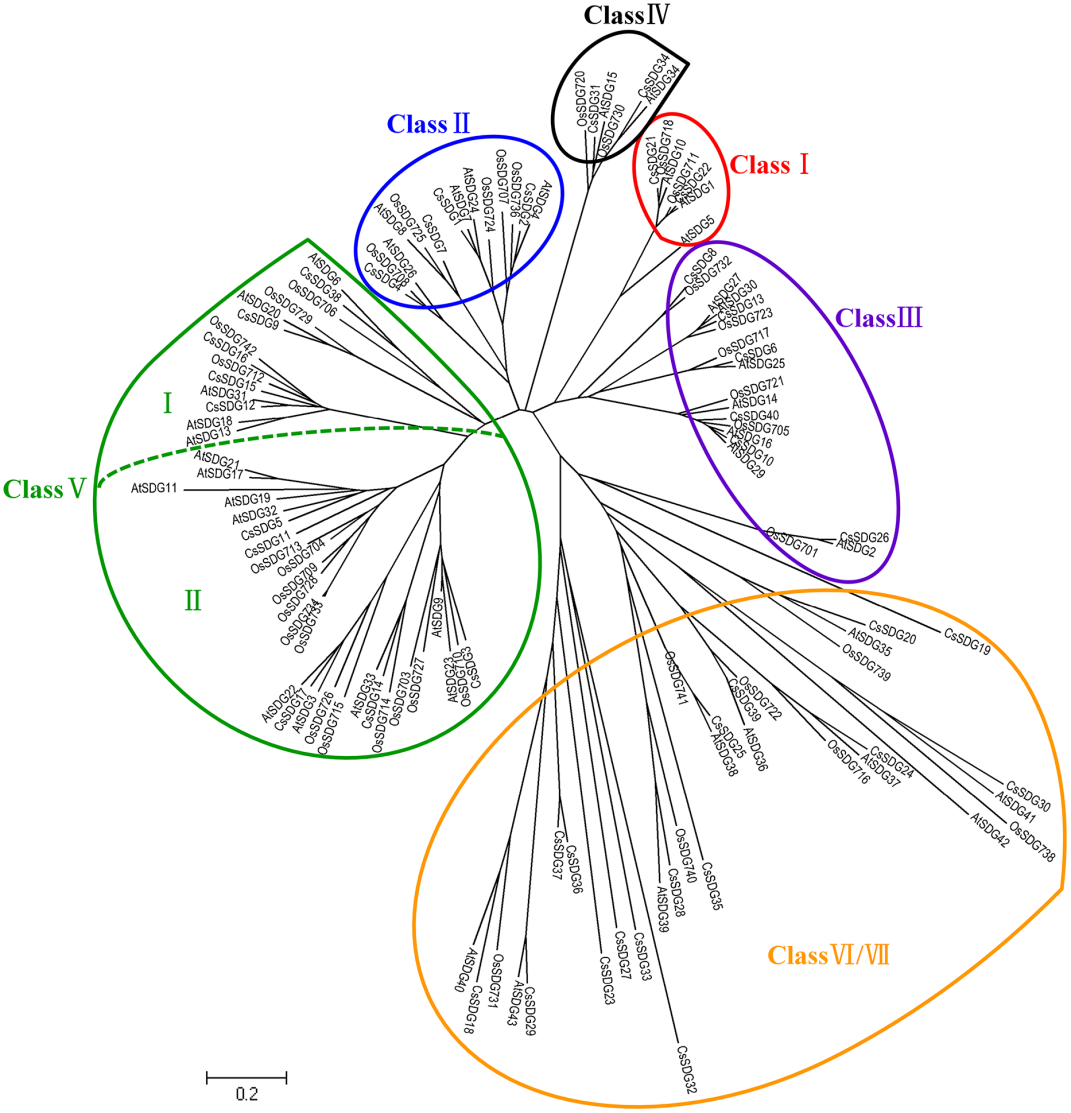
**Phylogenetic analysis of *SDG* family between *Citrus Sinensis* and *Arabidopsis*.** The tree was constructed by the Neighbor-Joining method with MEGA program 5.05 using the conserved SET-domain region.

All of the 40 CsSDGs were divided into seven classes according to the classification criteria of SDG family in *Arabidopsis* (Springer et al., 2003; **Figure 2**). In detail, CsSDG21 and 22, which were clustered with three AtSDGs, belonged to class I [E(z)-like]. CsSDG21 and 22 commonly had conserved SANT, CXC and SET domain and CsSDG22 had an additional SANT domain (**Figure 4**). Class II was comprised of four ASH1-like CsSDGs, clustered with five *Arabidopsis* and five rice ASH1-like proteins. CsSDGs in this class contained the conserved SET, Post-SET and AWS domains, while CsSDG2 and CsSDG7 had an additional PHD and CW domain, respectively. The TRX (TRITHORAX) family (class III) included six CsSDGs which was featured by highly conserved SET and Post-SET domains. CsSDG31 and 34 with a SET and N-terminus PHD domain belonged to class IV. Ten CsSDGs homologous to SU(VAR)3–9 group belonged to class V and were further divided into two main clades. Each of subgroups had five CsSDG members. Three members of Subgroup I contained WIYLD domain and Subgroup II was featured by a conserved SRA domain at the N-terminus. Sixteen CsSDGs clustered within class VI and class VII and eight of them contained Rubis-subs-bind (RBS) domain. As regards CsPRMTs, seven predicted proteins characterized by PRMT5 domain (**Figure 4**) were categorized to two classes (Supplementary Figure S1) according to the previous study (Aiese Cigliano et al., 2013). CsPRMT2 to 5 proteins were clustered to class I, and CsPRMT1, 6, 7 proteins belonged to class II.

#### CsHDMs

**FIGURE 3:**
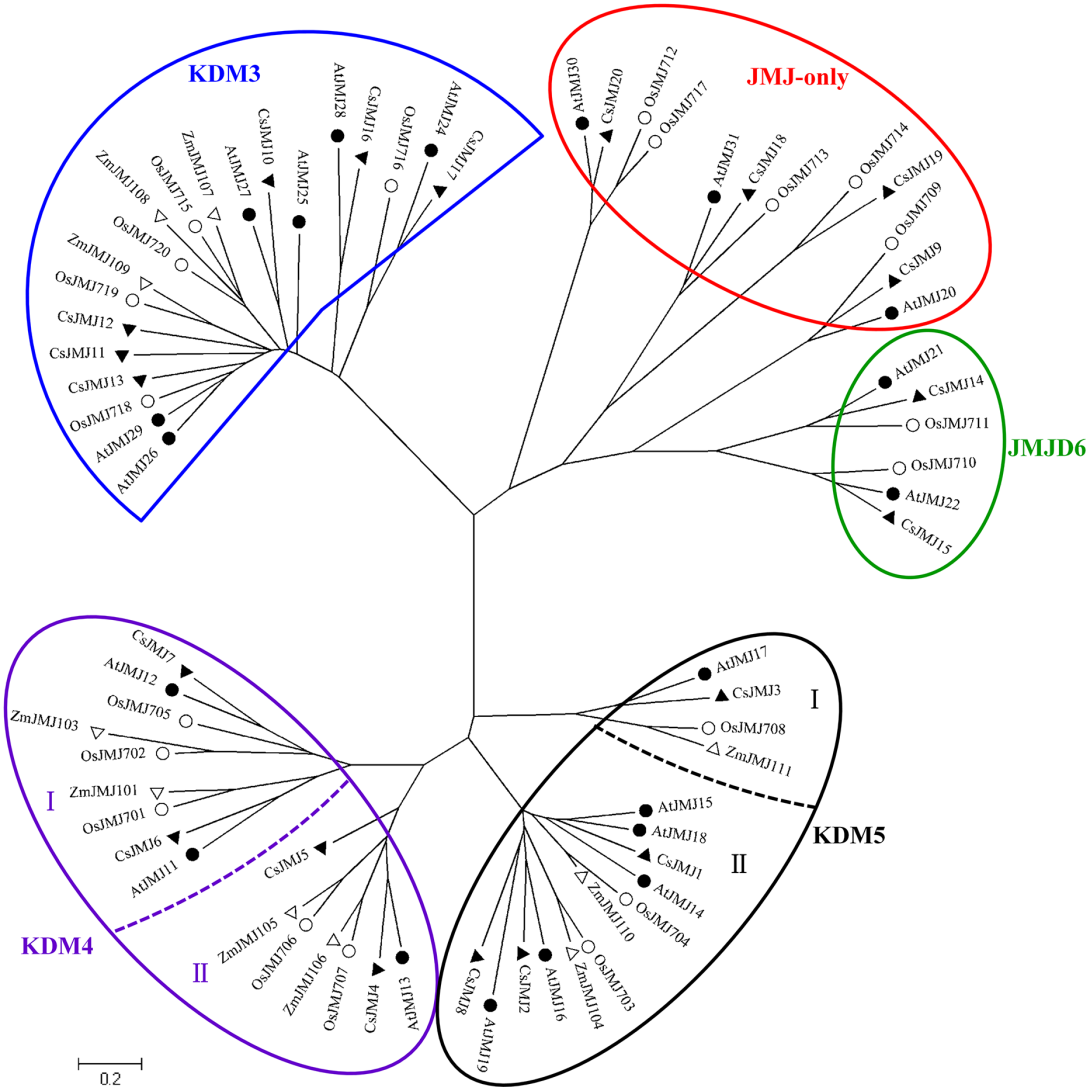
**Phylogenetic tree for *Citrus Sinensis*, *Arabidopsis*, *Oryza sativa*, and *Zea mays JMJ* family.** The tree was constructed by the Neighbor-Joining method with MEGA program 5.05.

The Phylogenetic tree of HDMAs was clustered to two main clades (Supplementary Figure S1) and all of three CsHDMAs were characterized by conserved N-terminal SWIRM (PF04433) domain and C-terminal Amino_oxidase (PF01593) domain. JMJ family was grouped into five classes based on sequence similarities, including JMJ-only (class I), KDM3 (class II), KDM4 (class III), KDM5 (class IV), and JMJD6 (class V) groups (**Figure 3**; Lu et al., 2008). JMJ-only class included four citrus members (CsJMJ9 and CsJMJ18-20) which only had JmjC domain and were not clustered to other groups. However, amino acid analysis of *Arabidopsis* and rice JMJ-only members indicated that they could be active demethylases (Lu et al., 2008). KDM3 class had six citrus members (CsJMJ10-13, CsJMJ16, and CsJMJ17), featured by a JmjC domain at the C-terminal with Ring finger domains (SM000184) ahead of it (**Figure 5**). The CsJMJs of KDM4 class fell into two main subclasses corresponding to the domain composition. Subclass I was characterized by four tandem repeats of ZnF_C2H2 domain (SM000355), while subclass II contained a zf-C5HC2 domain (PF02928) at the C-terminal. KDM5 group was also divided into two main subclasses which contain one (CsJMJ3) and three citrus members (CsJMJ1, 2, and 8), respectively. Additionally, the class of JMJD6 had two citrus members which had JmjC and N-terminal FBOX domain.

**FIGURE 4:**
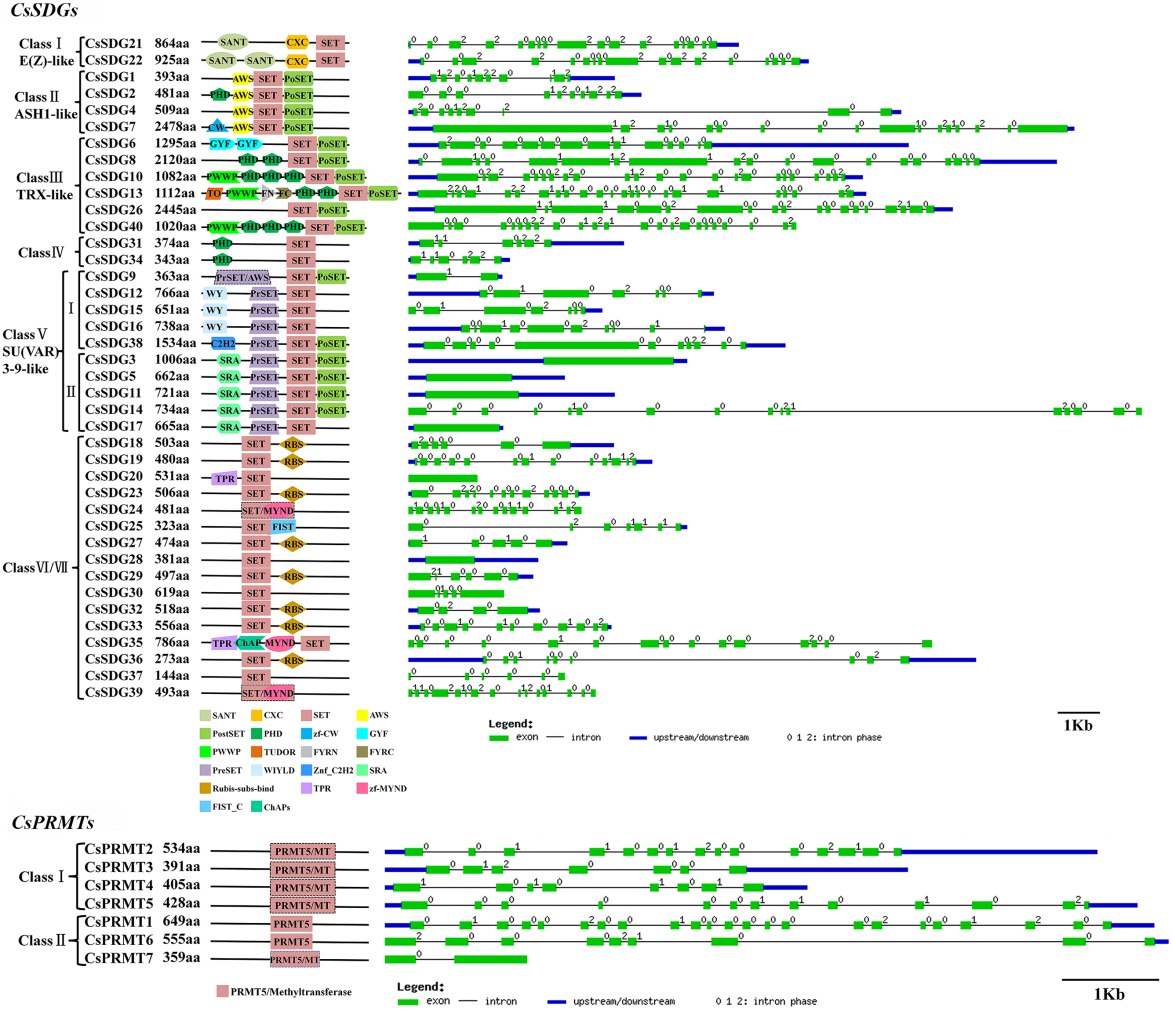
**Domain composition and gene structure of sweet orange *SDGs* and *PRMTs*.** Exon/intron structures of these genes were placed on the right side of the domain composition. Exon(s) and intron(s) were represented by green boxes and black lines, respectively. The blue box represented UTR region of gene upstream and/or downstream.

**FIGURE 5:**
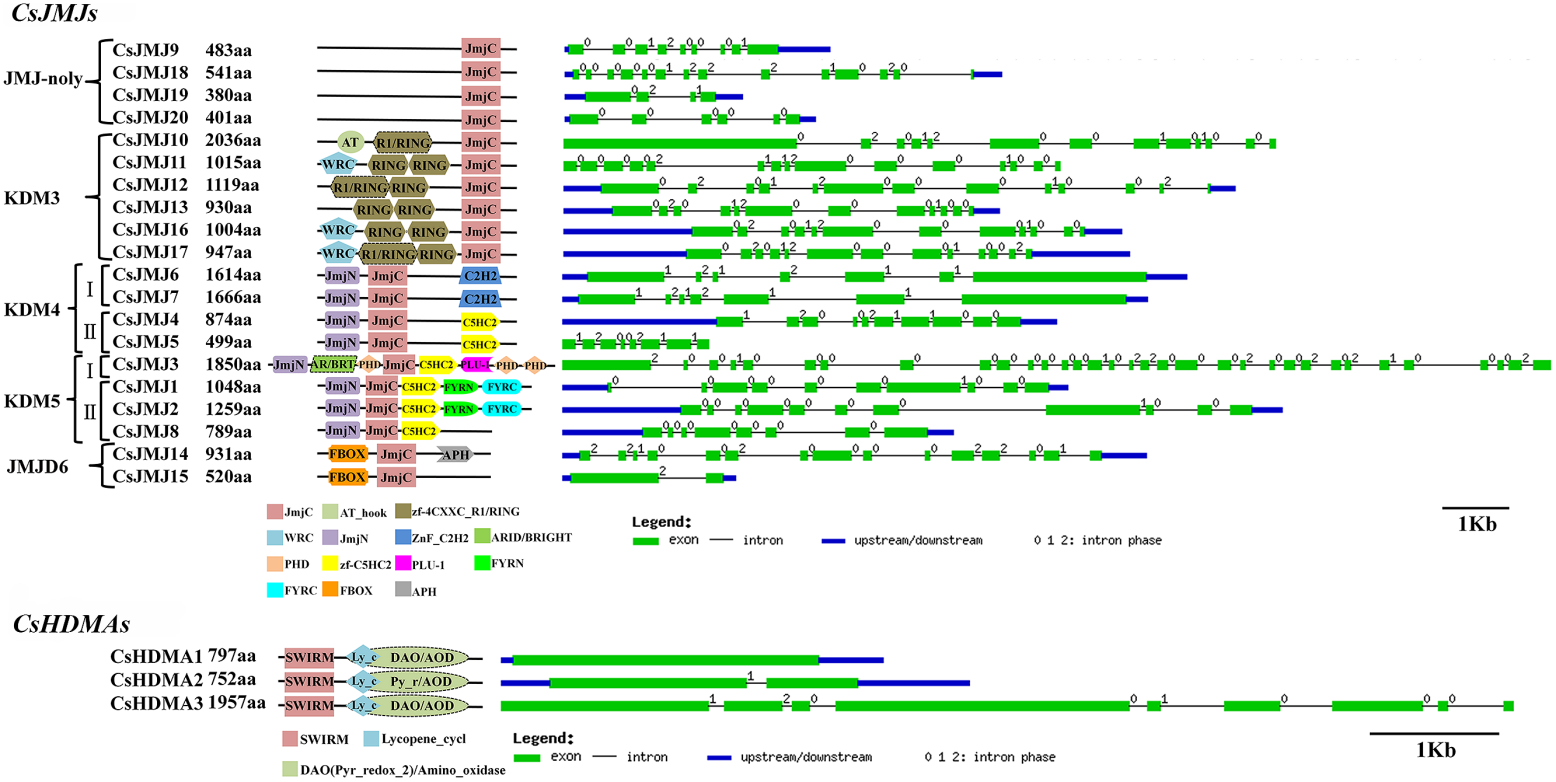
**Domain composition and gene structure of sweet orange *JMJs* and *HDMAs*.** Exon/intron structures of these genes were placed on the right side of the domain composition. Exon(s) and intron(s) were represented by green boxes and black lines, respectively. The blue box represented UTR region of gene upstream and/or downstream.

#### CsHATs

**FIGURE 6:**
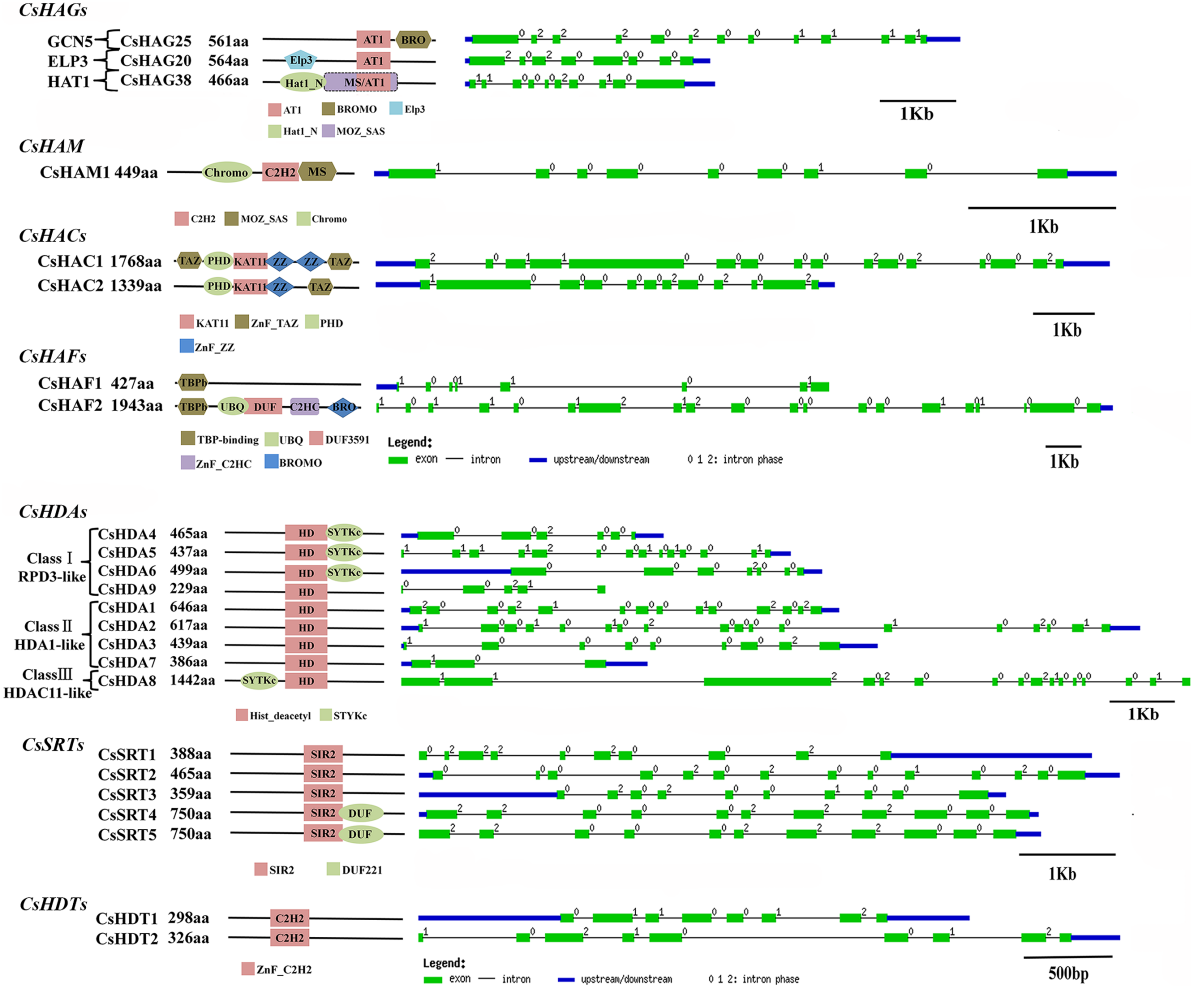
**Domain composition and gene structure of sweet orange *CsHAGs*, *CsHAM*, *CsHACs*, *CsHAFs*, *CsHDAs*, *CsSRTs*, and *CsHDTs*.** Exon/intron structures of these genes are placed on the right side of the domain composition. Exon(s) and intron(s) were represented by green boxes and black lines, respectively. The blue box represented UTR region of gene upstream and/or downstream.

The Phylogenetic tree and domain composition demonstrated that CsHAG25, 20, and 38 were belonged to GCN5, ELP3, and HAT1 class respectively (Supplementary Figure S1). **Figure 6** only presented these three proteins domain compositions, while the other members only contained the AT1 conserved domain. In detail, CsHAG25 carried a C-terminal Bromo domain (PF00439) which recognized acetylated lysine residues. CsHAG20 had an N-terminal Elp3 domain, which was conserved in AtHAG3, OsHAG703 and two maize ELP3 proteins. CsHAG38 was a GNAT/MYST-Like (GML; Aiese Cigliano et al., 2013) member featured by N-terminal Hat1_N (PF10394) and an additional MOZ_SAS (PF01853) domain (**Figure 6**). As regard CsHAM1, it composed by Chromo (PF00385), C2H2 (PF00096), and MOZ_SAS (PF01853) domain which was the typical domain architecture of HAM family. The two CsHACs presented the similar domain compositions, while CsHAC1 had an additional ZnF_TAZ and a ZnF_ZZ domain. Although the two citrus TAF_II_250-like genes CsHAF1 and CsHAF2 showed high similarity with each other, CsHAF1 only had the TBPb domain and lacked other conserved domains (**Figure 6**).

#### CsHDACs

As shown in Supplementary Figure S1, CsHDAs were divided into three classes according to the previous study in *Arabidopsis*, rice and maize (Alinsug et al., 2009). All of them contained conserved Hist_deacetyl domain (PF00850) and an additional STYKc (SM00221) domain was presented in CsHDA4, 5, 6, and 8. Five of CsSRTs are characterized by an SIR2 domain (PF02146) and CsSRT4 and 5 had an additional DUF domain (PF02714) at the C-terminal. In addition, the phylogenetic tree of HDTs showed that CsHDT1 was close to AtHDT1 and AtHDT2, while CsHDT2 was most closely related to AtHDT3 (Supplementary Figure S1).

### Expression Patterns of *CsHMs* in Different Tissues

**FIGURE 7:**
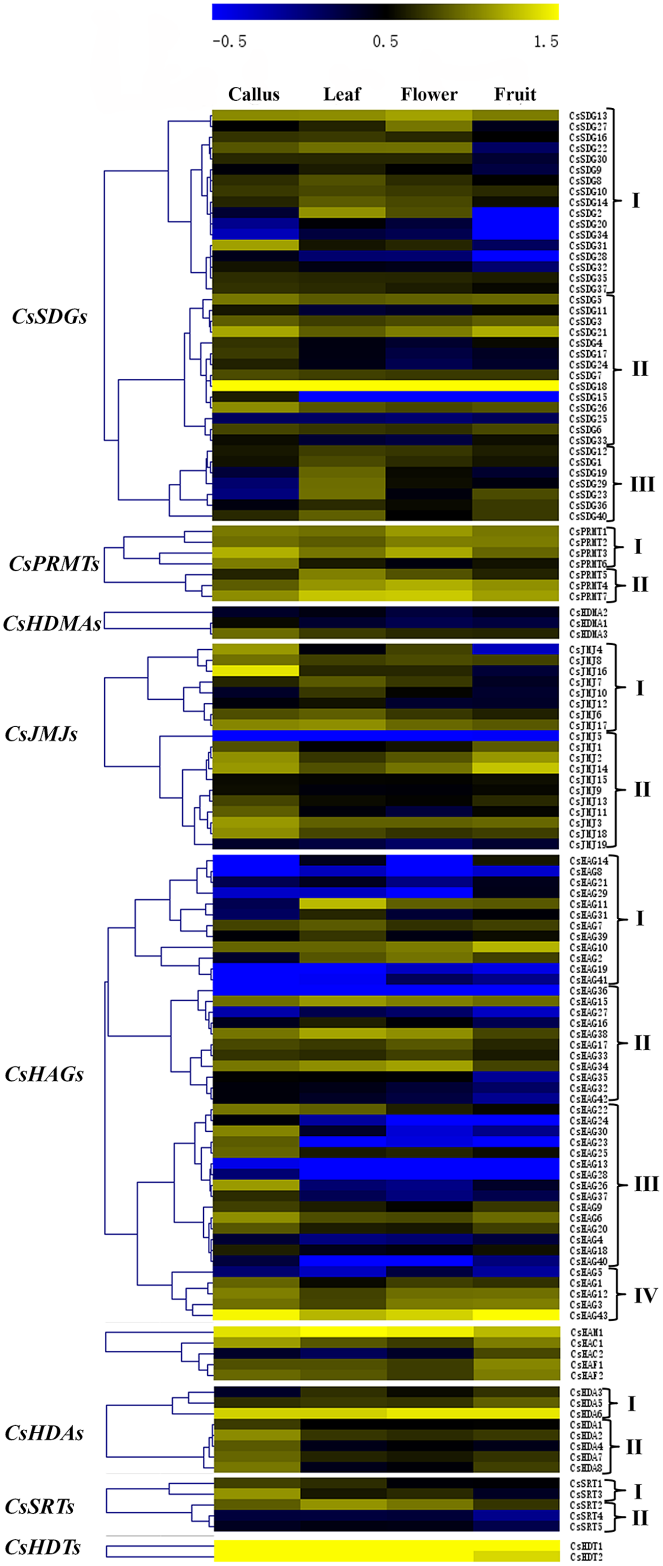
**Hierarchical clustering of each *CsHM* family.** The expression data were extracted from the *Citrus sinensis* Annotation project (CAP). The normalized RPKM values were transformed to log10 values and visualized with the heat map using MeV 4.7 software. The members of each family were clustered to different gene expression groups (I–IV) based on the hierarchical analysis with Pearson correlation.

The expression patterns of all *CsHMs* in different tissues (callus, leaf, flower, and fruit) revealed by RNA-seq data of the CAP (Xu et al., 2013) were demonstrated in **Figure 7**. Hierarchical cluster analysis was performed based on the expression data of each *CsHM* gene family corresponding to the four different tissues. According to the hierarchical clustering results, we classified genes of each family to different expression pattern groups (I–IV). As shown in **Figure 7**, the group I genes of *CsSDGs* showed a low expression level in fruit tissues, while the members of group III were expressed highly in leaf. The members of *CsPRMTs* presented a high expression level among these four tissues. For *CsJMJs*, genes in group I were expressed lowly in fruit, while group II genes showed a relative high expression level in callus and fruit. The *CsHAGs* mainly grouped to four expression patterns and the high expression in callus was prevalent among the group III members. Moreover, the genes belonged to *CsHDAs* group I expressed highly in fruit and the group II members showed high expression in callus. Above all, the various expression profiles of *CsHMs* among the four tissues indicated that these genes might take part in different biological processes in sweet orange.

### Dynamic Expression Patterns of *CsHMs* in Different Fruit Developmental Stages

In order to gain insights into the biological roles of *CsHMs* in citrus fruit development, genes with RPKM values higher than 5.0 in fruit tissues according to RNA-seq data were selected to further analyze their expression profiles during the six fruit developmental stages using real-time PCR. The real-time PCR primers of each *CsHMs* were listed in Supplementary Table S2.

#### CsHMTs/CsHDMs

**FIGURE 8:**
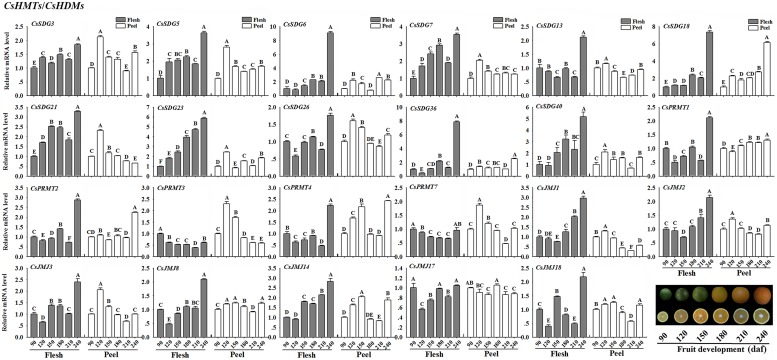
**Expression profiles of selected *CsHMTs* and *CsHDMs* (RPKM > 5 in fruit tissue based on RNA-seq data) in peel and flesh during six fruit developmental stages (90–240 daf-days after flowering) using real-time PCR.** Data were mean ± SD of three separate measurements. *Capital letters* indicated significant differences at *P* < 0.01.

As shown in **Figure 8**, most of selected *CsSDGs* were expressed highly in flesh of citrus fruit at the mature stage (240 daf). Notably, the increasing expression levels of *CsSDG6*, *7*, *18*, *23*, and *40* in flesh were strongly correlated with the fruit development process (**Figure 8**). For *CsPRMTs*, the mRNA abundance of *CsPRMT1*, *2*, and *4* were expressed highly in flesh at the 240 daf stage. As regard *CsJMJs*, all members of KDM5 class (*CsJMJ1*, *2*, *3*, and *8*) showed the increasing expression patterns in flesh along with the fruit development. Additionally, the expression levels of *CsJMJ14* also increased during fruit development in flesh. However, the expression profiles of these selected genes showed more complicated in peel during the fruit development.

#### CsHATs/CsHDACs

**FIGURE 9:**
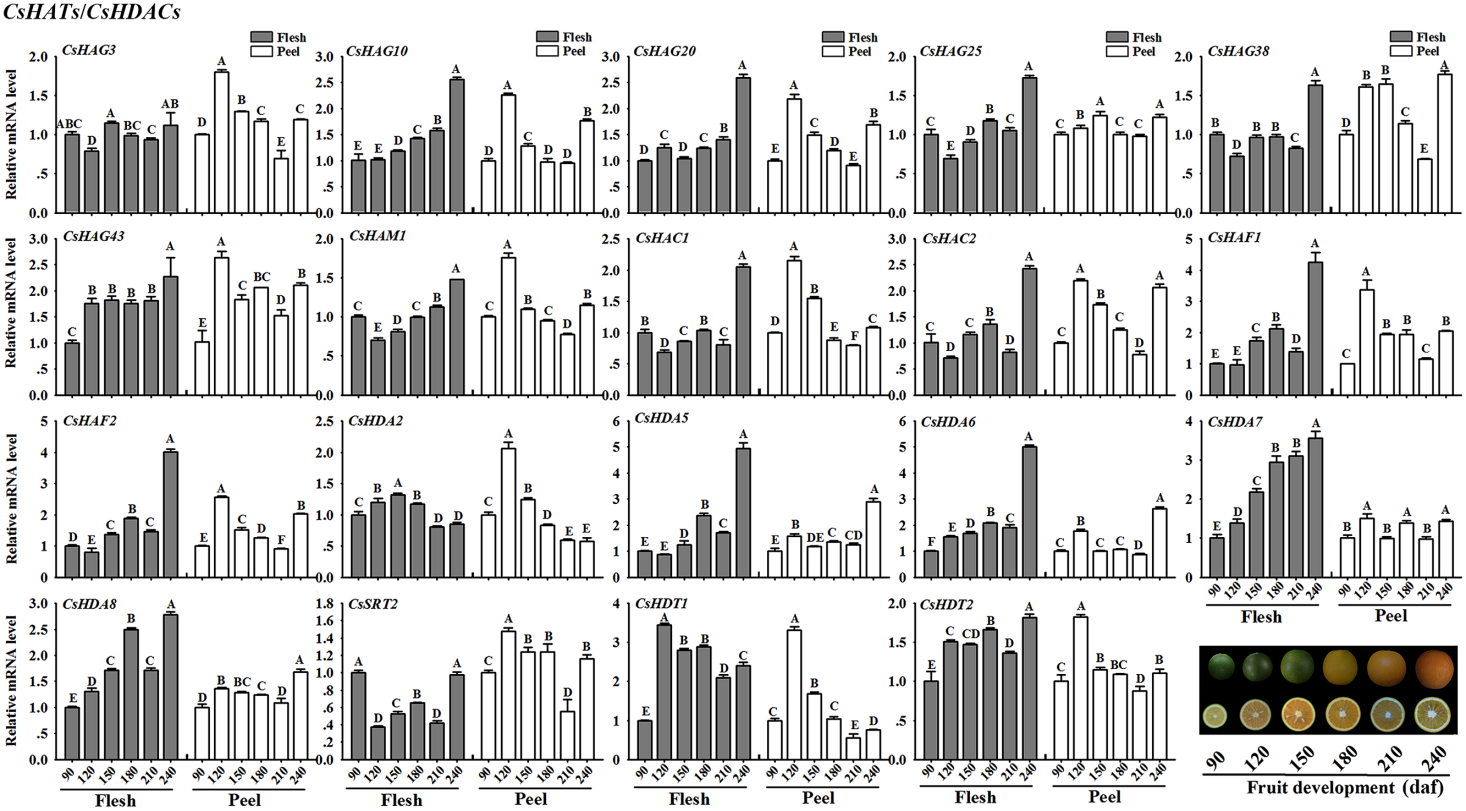
**Expression profiles of selected *CsHATs* and *CsHDACs* (RPKM > 5 in fruit tissue based on RNA-seq data) in peel and flesh during six fruit developmental stages (90–240 daf-days after flowering) using real-time PCR.** Data were mean ± SD of three separate measurements. *Capital letters* indicated significant differences at *P* < 0.01.

**Figure 9** showed the expression profiles of the selected *CsHATs* and *CsHDACs* genes. In flesh, *CsHAG10* and *20*, *CsHAM1*, *CsHAF1* and *2* presented an increasing expression level along with the fruit development. Additionally, two *CsHACs* showed a high expression level at the mature stage (240 daf) of fruit. As regard *CsHDAs*, the expression profiles of *CsHDA5*, *6*, *7*, and *8* presented an increasing trend during the fruit development. Notably, the expression level of *CsHDA7* showed the strong positive correlation with the citrus fruit development. In peel, the selected *CsHATs* genes showed a similar expression pattern with high expression levels at 120 daf stage of fruit development.

### Expression Profiles of *CsHMs* Response to Blue Mold Infection

**FIGURE 10:**
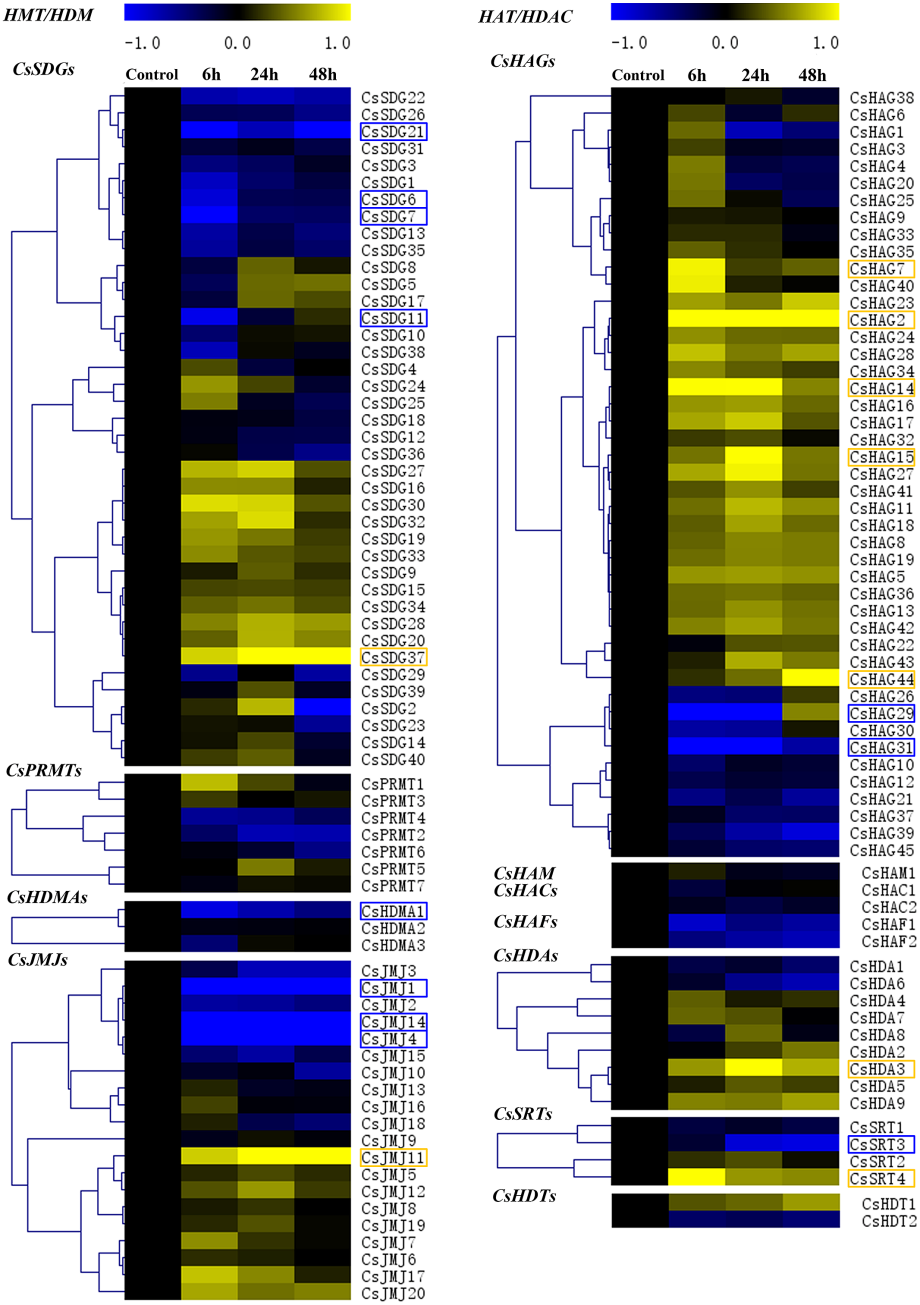
**Expression profiles of all *CsHMs* in response to blue mold (*Penicillium digitatum*) infection of citrus fruit at different periods (Control, 6, 24, and 48 h) using real-time PCR.** Transcripts were normalized to Actin gene expression and the expression level of control was used as the calibrator for relative expression analysis. Hierarchical clustering of each *CsHM* family was performed using MeV 4.7 software. Genes with log2 value higher than 1.0 (up-regulated) or lower than -1.0 (down-regulated) were marked with yellow box or blue box, respectively.

A growing body of studies had been revealed the crucial roles of *HMs* in various abiotic stresses and plant immunity. Blue mold is considered as the most devastating pathogen in citrus fruit post-harvest process and causes lots of rotting losses. In order to determine the *CsHMs* responding to fruit-blue mold infection, the expression profiles of all *CsHM*s were detected among the four periods of infection using real-time PCR. The results were visualized by the heat maps and hierarchical clustering was further analyzed in each *CsHM* family (**Figure 10**). A numbers of *CsHMs* were up (yellow color) or down (blue color) regulated by the blue mold infection. According to these data, 20 genes including five *CsSDGs*, one *CsHDMA*, four *CsJMJs*, seven *CsHAGs*, one *CsHDA*, and two *CsSRTs* were selected to present their expression patterns in Supplementary Figure S2 for their expression fold change higher than 2.0 compared with the control. The expression levels of *CsSDG6*, *7*, and *11* were inhibited at 6 h after infection (hai) and recovered at 24 and 48 hai. On the contrast, *CsSDG37* was up-regulated by the infection. Three *CsJMJs* (*CsJMJ1*, *4*, and *14*) were strongly down-regulated, while *CsJMJ11* were induced by the infection. Five *CsHAGs* including *CsHAG2*, *7*, *14*, *15*, and *44* were up-regulated, while the expression levels of *CsHAG29* and *31* were strongly inhibited at 6 and 24 hai and then recovered at 48 hai. For *CsHDAs*, *CsHDA3* was selected out and showed the increased expression levels under the infection. Additionally, two *CsSRTs* (*CsSRT3* and *4*) presented different responses to infection. *CsSRT1* was down-regulated, while *CsSRT4* was highly up-regulated at the 6 h and then slightly induced at the 24 and 48 h by the infection.

## Discussion

Chromatin based gene regulation affects various processes such as root growth, flowering time, floral organogenesis, gametophyte, and embryo development as well as plant response to pathogens and environmental changes (Alvarez et al., 2010; Deal and Henikoff, 2011; Gu et al., 2014; Kim et al., 2015). A series of gene families have been proved to be involved in establishment of histone modifications which can determine chromatin state to regulate biological processes (Berr et al., 2011). Here, genes involved in histone methylation/demethylation and acetylation/deacetylation have been genome-widely characterized in sweet orange based on the sequenced genome (Xu et al., 2013; Wu et al., 2014). Eleven gene families (*SDGs*, *PRMTs*; *HDMAs*, *JMJs*; *HAGs*, *HAM*, *HACs*, *HAFs*; *HDAs*, *SRTs*, and *HDTs*) containing 136 *CsHMs* were identified in sweet orange genome. The gene numbers of these families in sweet orange are close to the *Arabidopsis*. For example, *CsSDGs* and *CsJMJs* have 40 and 20 members respectively (**Table 1**) and the corresponding *AtSDG* and *AtJMJ* families contain 41 and 21 members (ChromDB database). However, much more HAG genes (45 members) were identified in sweet orange genome compared with in *Arabidopsis* (three members), rice (3) and maize (4; ChromDB database). But if we used the AT1 domain as a query to apply the Blast program, 33 HAG members were obtained in *Arabidopsis* (Aiese Cigliano et al., 2013), which was close to the number of *CsHAGs*. Moreover, 26 predicted HAG proteins were identified in tomato genome based on the similar searching methods (Aiese Cigliano et al., 2013).

Recently, an increasing number of *HMs* have been identified and unraveled their pivotal roles in regulations of essential processes (Berr et al., 2011). The involvements of *HMs* in citrus fruit development have not yet been described. However, numbers of tomato *HMs* had been characterized their potential roles in tomato ripening process (Aiese Cigliano et al., 2013). A study on grape *SDG* family has revealed that several *VvSDGs* were increasing their expression levels during the grape berry development (Aquea et al., 2011). In our study, we also revealed that a number of *CsHMs* showed the increasing expression patterns during the citrus fruit development (**Figures 8** and **9**). Regarding histone methylation in plants, SDG family controls the methylation of histone lysine residues, which are involved in various biological processes such as flowering transition, hormone regulation, and carotenoid biosynthesis (Cazzonelli et al., 2009; Sun et al., 2012; Kim et al., 2013a). Generally, histone H3K9 and H3K27 methylation are two repressive marks, whereas H3K4 and H3K36 methylation activates gene expression (Berger, 2007). A number of SDGs has been characterized their catalytic functions in *Arabidopsis* and rice (Liu et al., 2010). AtSDG9, 23, 31, 33 and OsSDG714, belonging to Class V (**Figure 2**), are responsible for H3K9 methylation, while AtSDG1, 5, 10 (Class I), 15 and 34 (Class IV) catalyze H3K27 methylation. For the activation marks, AtSDG27, 30 (Class III) and 4 (Class II) act on the H3K4 residues and AtSDG4, 8 and 26 (Class II) catalyze the H3K36 methylation. Although the enzymatic activity and specificity of citrus SDGs are not known, genetic data suggest that they may catalyze the same lysine residues and act the similar repression/activation functions with *Arabidopsis*. During citrus fruit development, *CsSDG7* showed the increasing expression levels and *CsSDG13* presented the high expression level at the mature stage (**Figure 8**). Moreover, CsSDG7 and CsSDG13 are homologous to AtSDG8 and AtSDG27 which catalyze the activation marks H3K36 and H3K4 methylation respectively, indicating that *CsSDG7* and *CsSDG13* could have similar functions and activate genes expression during the fruit development. Furthermore, *AtSDG8* encoding a HMT can affect the carotenoid biosynthesis via regulating the H3K4 tri/di-methylation on *CRTISO* (*CAROTENOID ISOMERASE*) which controls the carotenoid isomerization in carotenoid biosynthetic pathway (Cazzonelli et al., 2009). Citrus fruits accumulated nearly 115 kinds of carotenoids and the total carotenoids content increased rapidly during the fruit development (Rouseff et al., 1996; Kato et al., 2004). Previous study revealed that the ‘Anliu’ sweet orange used in this study showed a rapid increase of total carotenoids in flesh after the green stage (150 daf), which was attributed to the increased accumulation of β-cryptoxanthin and violaxanthin (from an undetectable level to 2.28 μg/g and from 0.99 to 4.63 μg/g, respectively; Liu et al., 2007). In this study, *CsSDG7*, being homolog to *AtSDG8* (**Figure 2**), showed the increasing expression levels in flesh during the fruit development process (**Figure 8**). This implied that *CsSDG7* could also be involved in citrus fruit carotenoid accumulations during the fruit development. Additional expression analysis of *CsCRTISO* gene during the citrus fruit development also showed that the *CsCRTISO* was expressed highly in flesh at the mature stage of fruit development (Supplementary Figure S3). For histone arginine methylation, *atprmt4a* and *atprmt4b* double mutant, *atprmt5/skb1*, and *atprmt10* display the late flowering phenotype by increasing the *FLC* expression in *Arabidopsis*, indicating these *AtPRMTs* are required in the *Arabidopsis* flowering transition process (Pei et al., 2007; Niu et al., 2008; Schmitz et al., 2008). In our study, *CsPRMT1* and *CsPRMT2*, being homolog to *AtPRMT5* and *AtPRMT4* respectively, showed the high expression levels in flesh of fruits at 240 daf stage. Based on the similarity and expression profiles, we expected that these two *CsPRMTs* could also be required in citrus fruit ripening process. Regarding histone demethylases, *Arabidopsis* JMJ14 catalyzes histone demethylation at H3K4 residues and represses flowering (Lu et al., 2010). A recent study has revealed that two novel NAC transcription factors NAC050 and NAC052 interacted with the FYRC domain of AtJMJ14 to regulate gene expression and flowering time (Ning et al., 2015). AtJMJ15 is an H3K4me3 demethylase and *AtJMJ15* overexpression resulted in an obvious early flowering phenotype (Yang et al., 2012). CsJMJ1 with a FYRC domain (**Figure 5**) is clustered with AtJMJ14 and AtJMJ15 (**Figure 3**), suggesting CsJMJ1 is a predicted H3K4 demethylase with repression of gene expression. Moreover, the expression levels of *CsJMJ1* and the other members of KDM5 class (*CsJMJ2*, *3*, *8*) presented an increasing trend during the citrus fruit development (**Figure 8**) and we expect that these genes could be functional during citrus fruit development.

For histone acetylation/deacetylation, one HAF gene (*SlHAF1*) was identified in tomato genome and it has the strongest expression in tomato fruit at 10 days after breaking, suggesting an important role in tomato maturation (Aiese Cigliano et al., 2013). Similarly, two identified *CsHAFs* (*CsHAF1* and *2*) also showed a high expression level in flesh at the mature stage (240 daf) of citrus fruit development (**Figure 9**), implying that they could have the similar functions with *SlHAF1* in tomato. The best studied *HDACs* in *Arabidopsis* belonged to RPD3 (Class I), including *AtHDA6*, *19*, *7*, *9* and pseudogenes *AtHDA10* and *17* (Supplementary Figure S1). AtHDA19 was a member of AP2-TPL-HDA19 repressor complex which negatively regulated multiple floral organ identity genes in *Arabidopsis* (Krogan et al., 2012). *AtHDA9* repressed *Arabidopsis* flowering by removing H3K9Ac and H3K27Ac on the flowering promoting gene AGAMOUS-LIKE19 (AGL19; Kim et al., 2013b). *AtHDA9* and *AtHDA6* worked redundantly in the repression of embryonic properties (Tanaka et al., 2008) and *AtHDA7* was required for female gametophyte development and embryogenesis (Cigliano et al., 2013). Moreover, a study on tomato revealed five *SlHDAs* (*SlHDA1*, *3*, *5*, *6*, and *7*) could have the potential roles in fruit development and ripening process by their high expression levels in tomato fruit development (Aiese Cigliano et al., 2013). In this study, *CsHDA5* and *6*, being homolog to *AtHDA9* and *19* respectively, also showed the increasing expression levels in flesh during the citrus fruit development (**Figure 9**), suggesting that these two genes could also have deacetylation functions with the repression on genes involved in fruit development.

A few studies have shown that the *HM* genes play vital roles in plant immunity (Alvarez et al., 2010). In *Arabidopsis*, HMT gene *SDG8* was confirmed to be crucial in plant defense against fungal pathogens by regulating genes within JA (jasmonic acid) and/or ethylene signaling pathway (Berr et al., 2010a). ATX1 (AtSDG27), a TRX (TRITHORAX) member involved in H3K4 trimethylation, activated WRKY70 and SA-sensitive genes and reinforced basal resistance to *Pseudomonas syringae* (Alvarez-Venegas et al., 2007). In our study, *CsSDG7* and *CsSDG13*, being homologous to *AtSDG8* and *AtSDG27* which catalyzed the H3K36 and H3K4 methylation respectively (both of them activated gene expression), were down-regulated under the blue mold infection (**Figure 10**). For *HDAs*, *AtHDA6* was involved in regulating tolerance to necrotrophic fungi by repressing the JA and ethylene signaling in *Arabidopsis* (Zhu et al., 2011). *AtHDA19* activated the resistance against *Alternaria brassicicola* and was also involved in JA and ethylene signaling of pathogen response in *Arabidopsis* (Zhou et al., 2005; Choi et al., 2012). In citrus, *CsHDA4*, being homologous to *AtHDA6*, was up-regulated responding to blue mold infection, while *CsHDA6*, clustering with *AtHDA19*, was suppressed (**Figure 10**). Overall, 20 genes including five *CsSDGs*, one *CsHDMA*, four *CsJMJs*, seven *CsHAGs*, one *CsHDA*, and two *CsSRTs* exhibited the strong alterations of their expression levels under the infection (Supplementary Figure S2). The expression change of these genes implied that they could be involved in fruit-blue mold infection process.

## Conclusion

This study provided the first insight into the *CsHMs* in citrus and their expression patterns during the citrus fruit development as well as response to fruit-blue mold infection. These *CsHM* genes were further characterized from the perspectives of genomic organization, phylogenetic relationship, domain composition, and gene structure. Additional expression analysis of these genes was measured in six different fruit developmental stages and four periods of blue mold infection. From the results, we obtained a numbers of genes with the increasing expression profiles during the fruit development and 20 strongly blue mold responsive genes. The comprehensive characterizations of *CsHMs* presented in our study will be useful for future research to unravel the mechanisms of histone modification regulations in citrus.

## Conflict of Interest Statement

The authors declare that the research was conducted in the absence of any commercial or financial relationships that could be construed as a potential conflict of interest.

## Footnotes

1 http://pfam.sanger.ac.uk/

2 http://citrus.hzau.edu.cn/orange/

3 http://phytozome.jgi.doe.gov/pz/portal.html

4 http://hmmer.janelia.org/

5 http://gsds.cbi.pku.edu.cn/

6 http://www.chromdb.org/

7 http://www.megasoftware.net/

8 http://citrus.hzau.edu.cn/orange/

## Supplementary material

TABLE S1 **List of the histone modification genes (CsHMs) in sweet orange**.

TABLE S2 **Real-time PCR primers of sweet orange HMs**.
