# Supplementary material for: Genome-wide identification of sweet orange (Citrus sinensis) histone modification gene families and their expression analysis during the fruit development and fruit-blue mold infection process
Source: Front Plant Sci. 2015 Aug 5;6:607. doi: 10.3389/fpls.2015.00607 (PMC4525380; doi:10.3389/fpls.2015.00607)
Supplement: Supplementary file 3 [file Data_Sheet_1.DOCX]

**CsHMTs:**

**CsSDGs:**

>CsSDG1

ATTTTATTGCCGTGCAATAGTTATCGGCTGTTATTTTCCTTTTCACTCTCAAAAACTTCCCTCCTAAAAACCCAAATCGCTCAATTCAGAGATCTGCCTTCTGTGAACTTGCCTTAAGGTAGATTTCTCTTCCTCTTTCACTAATTGCACTTCCTGTTCTCTTCATTACTTTCACTGTCTTGCCTACCTTACGGGCTTTAAGTTTTGCCAAATCTTGATATCAATTGTTTGTAAAACGAATTTGGGAATTTGAATTTGTTGGTTCAAGCGCTTTGTATATTATATAAATGATTTTTTTTTTCTTTTTGTACTCAAGATTTAGCATATTTAATTTCTTGGTTATCTTGTAGGCTGCTGCAGCCTTGGTGAAAGAGTTTTGATGCCATGCCGGCTGCAAAGAAGGTTAAAGCTCTCAGCTTTTCACAAATGTTTTATTGCTTTGTGTTTGTTTTGTCTACTGATTTTTAGATAGTTGAATTATTCTTACATTTTGGCTTTTTTTTTTTCTTTTATGTTCAGAATTCTGATAATAGCCAGATAGGCCATGCGTTTAATAAGTTGTTGAAACAGATTGGGAACCCAGTTGAGTTTGAATTACCCGATTGGTTTATTAAACCGAAGGCCATACCTTATGTCTTCATTAAGCGAAGTATCCACTTAAACTTCTCTTTCGAGTTGTTGTTGTTGTTGTTCTGTTGCTTGTTTTTAATTGCTTAAGTTACTGTTCTTTCTCTTTTGTGATTTTCTTGGTGTTTATGAATTTCAGATATATATCTTACAAAGAGGATTAAGAGACGGCTTGAGGATGATGGCATATTCTGCTCTTGCACAGCATCACCAGGTTCTTCTGGTGTTTGTGATAGAGACTGTCATTGTGGGTAAGAAATTGTTTTTGTCTATCTCAGTTTCTCTGCCTTGTACTCTGATGTGTTTGAGGTTGTGATGGATTTTTATCTTGGGTAATCTAATGAGAAATTATGTGTTGTTGTACGCTTTATTTGGGCTCTTATTTATTTGCAGTTTCCTCATCCGAAATATTATTGTGTTTAGTTTATTTTTGTTGAACCCCAAGACTTGGTTTGCTTTTTTTCCTACAGGATGCTCCTGTCTAGCTGCTCTTCTGGCTGTAAATGTGGGAATTCATGCCTTAATAAGCCATTCCAGAACCGACCTGTGAAGAAGATGAAATTAGTGCAGGTTGAGCATTGAGAATAAAACTTCACATGTTAAAAACATTCCTTCCTTTTCATTCTTCAAGCACAAATGCATTAGACTTGATTCCAGTCTTTTGTTCAATGTCACTCCTTATGTGGTTTTTGTTATTGTTTCTTCTGTGTACACTCAAAATATGAAGTTAAGTAAGGTATTGCTTGTAGACAGAGAAATGTGGAGCTGGGATTGTGGCAGATGAAGATATTAAACGAGGAGAGTTTGTAATTGAATATGTTGGGGAAGGTGACATCTTGGTTGTTGCTTGTTCTTCATCAAAATTTCTTTCCATATTCTCATGTTGCATTATTTTATGATCCATTAGCATCTGCTTAGATGTTTCTTCAATGCAGTTATTGATGACCAAACATGTGAAGAAAGACTTTGGAAAATGAAGCACCTTGGAGAAACTAACTTTTACCTGTGTGAGATCAATCGTGACATGGTAATTGATGCCACATACAAAGGAAATAAATCAAGATACATAAACCATAGTTGCTGCCCCAATACTGAGATGCAGAAATGGTTCACACTGACCCCTCATCACTAATTTTGTTGTCATGTGAGTTGATTTCTTATCTTACGTGTTTTAATTTTGTTTTGCTCAAGGATAATTGATGGTGAAACGAGAATAGGCATATTTGCAACTCGTGACATAAAAAAGGGCGAAAATCTGACCTACGACTACCAGTACGAATTCTTGCATGACTCTCTTATTGCATACTGTTAGACATCTCTCTTTGAACATTGGTATTTTTTTTTTTTTTTATTGTAAGCATGCATATTTATATTTAGTATTTTGTGTCATGATCTCTTATGCATATTGATGACAGCTGGTAGATTGGTTTCCCTTCAAATTTTCTATTTTCATAGCCCAATATGCACTAATCTGACAAATTTAGCAGGTTTGTTCAATTTGGTGCAGATCAAGATTGCCACTGCGGAGCCGCAGGCTGCAGGAGGAAGCTGGGGGCAAAACCTAGCAAGCCTAAGATCTCATCAGATGCTGCACTAAAGTTGGTAGCCTGTCAGGTGGCTGTATCTTCTCCCAAATTGAAAGCAATATTATCTGGAAAAGATGTATGTATATGTGGCTGTTTGATAATCCATAATTTTATATATATATATATTAACTAGTGTTCAATGATTATATGTTGTGTGTTTTGATTATGGATACCTGCGATTTTGCTAACTTAGCTATCCAAGCTTTTTTCATTGGTATGTATGTTGTATGTGTGTACATTTATGTATAGCAAGTGTGACTATACATCCTTTCATGTATTATTACTTGATTGGTCAAAACGGGTTTGGGTGAGAATACTTTTGGAATGGCTTATGAATTATTTCAAGTCACTTTTGCCGTTATTTCTAAGTGGAAGTATTTCTAATACTTCATAAATGAATAAATTTTGAGGTAATGTTTAGCAACTTTTTCCCTTGGCTGCTATGCAATTATAATGCTTTTTGTATTTTTGTTCTTAAACTATGATTTCAATTTTCAAGGTATAGATACATTTGAATATCTTCTCTCTTACATCATCAGTGCAAGAGAATGTGTTCTACATTTAATTTTAGAGTGTTGTGTTCAAATTTGTAAGTTTTCACATAACACTTTGAGTGGATTATAGTCTGGCTTTGGTATATCACTGTTGTAAAATAGTCTGAGTCAACCTCTGAAAGAGTGGCGCGTTGCTTTGAAATTTGGTGGCTTCTTTTTGATAGCTGAGCCACAAAAAGTATTCTAAAGCCTTCTTTTATATTTCAAATTTTGAAATATAAATGGACATTTTCTTTGATTGGATTGGATCGGATCAAATTCAAAATTCAACAATGCAGTTGGAATAGATTAGGCCATTCTGTTCTTATTTCACCCCCTTTTCTTCCCTTCATTCCACTAGTATGCTACATCTAATATATTGGAAGATTTGCAAGCTCCAAAAATTCGTACTCAATTACTACGTAAGTTTTGATATTCATTTTTAATGCATTTCAGTTTTACACTTTCTATCCATGTTAGTTTGTGCTTAAGTTATATACTGTACATTTTCTACAAACTTGGACTGATTCTGGCATTTTTTTTGTTTAGTTTTATCAGAATGGAGATTTGCATATAGGTAAGAAAACTTTATTCCTGGATTCTAGATTATGTATTGGTTGCTTTGTCAAATATCTTAGTCTTGTTGCCATTCAGGCAGTTCGCAACCTCCTTATAATCAACGACAAATATGCCCTCAATGTTGCATTGGCAAAGTGATTAGAATATCCCATCCCAAGAATGAGAGGTACTTCCCAGAATGTTTAATTCCATCTGTAATTTACATCGTCAATGACTATATTCTGCTGAATTTTTTTTTCTTTCCCATTGGCACAAATATTACAGTTCTTTTGGGATTATTAGACGGTTTGATGAGTATTCCAGAAAACACTCGGTAAGAGTCTCTTTTATTTGGCTACCATTTAAAGTTATAAGAGCAATATGAACATATAAGAAAATGTGCTGTTGTAGGTCTTGTTTGAAGATGGTGAGAGTGAGTTTATTGACATGGCTAAAGTAGATTGGGAACTTGTAACTGACTGATGTCACTCTGAGGAGTAAGTACAACTTTCTCTTTCAATGAACGATATACATTCTTCTTCTGACCATATTATCCAATTATCAGTGCTTCAAATGACATTTTGGAGACATTTTGTGTTTTAATTAGAGCGGTTAGTTAGTGAACTTTTGTTATTGGCTTTAAGAAAGTTCTGATCTTGAAATCTGCTGTAATCTGCTTAGGAATTTGTTGAGAAGTATGAGAGAATTTCAATATGTAGTTTGAATGAGACATGAGCTTCTGATTATATTGTAGACTCTGAGAAACATGAGGTAGTGAGAGGGTCATTGTCTATGTAAAAATGGTCATGATGGCTGCTTCTCTTTCCTTATTCATTAGAGGGGCATGATTATGATTTCATTAATTTTTTAGCTCATATTAGTCACTTTTATGCCTGCATCAAACTGCTTTACATTGTGTTACTCTTTTTTGCATCTTGCCTTGCTTGATCTTTCCTTTGCTTGGAACCTTGGTTGGATATTTTTTTTCATTTATCTTGATTATTCTGGTCAGTTGAAATAAAACTGAGTACATCAATCATGTTCAGGTAGCAGTAACCAAAATTGGTAACTGGTTAGTTAGCTGTGCGGAATTACAGTCGTAGAAAAAAGATTCAATCAAGTTCATACAAATGGTTCTGGAGACCACATTGTTGTTGTACCAGAAGTATGAGCTTTTCTTGTAAACAAGGAGCCATCTTCCTTGCATAGGATTCATAACAAATGTTGTATCAAATGTAATTTTTTCCTGTAAAAACCTTTTGGAACATTTCAACTGTTGCATTCATTCTTGCTAGGAAATTCTTTTATTTTAGATGTTGCAGCAAGTTGAAGATGGGTGTGTGGTGTCTGGGGCCTAACTTTGAACATGTGATCTGGTAACTCTCGTCTCTATATTGTTGTGTTGTGGCCCCAACGCATGTTGCTCGTTTTTGCATTGCAAGCACATTGCGTACGCAACAGACTACGTGTGCTCCGCCATAAACAATCAACACGGCA

>CsSDG2

ATGCCAGACCTCGCGAATCTCGCTCTCTCCTCCTCCTCCTCCTCGCTAACCCTAACTCGGTGCGCCTCTTCTCTGAAGCCACTGGCTCCTCCTCACTCAGCCACGGAGTCGCCCGGCTCCGATTCGGCGGTGGTCAAAACGCTTGCTTTGACCGGCGAGGAGGAGAACGTCTGTGCTAACGGAAATGGTAATTCCGTTAGGGTCATGAAACGGTGCCGCGGCGCCAAGAATATTCCCGGTTTAGAGGACCACGTGGCGGCTTGGGTGAAGAAGAAGATGGAATTGGGGGTCCCACAGTCGAACTGCTCTCTTCCGTTTCTTGTCGGCGCCAAGAAAATGGTTTATTCTGATTCTGTTTATTACAATTTATTTAATTAGAAAGAATTAAAAGATATTGGATTTTTCTTGCTCTGTAGTTTAATTCTTTGAAAGAATTTTATATCTCTTGGGAACTTCAAGATATACAGTGAATCTTTTTGTTGGGTATACTTATTCCTGCACTTGACAGTAAGATTTTAAGTGAAAAATAGGATATATTTTTTATGAGGCTTATGTTGTTGTAAGAGTAAGGGTGTGTTGTGGACAAAGCACTGAGGTGTTGTGGATTTGAATCCACAGCAAAATCGCTAGTTGGTATAAATCTAACACTTTAGCTGCTTTTGTTTTTAAAATTAAGCAGAAGCACAATAGTTCCAGATGTACCCTAATTTTATTTTTCTGCAATTTCATTTCAGATTGAATGCCGTGCTTGCCATAGGTTTATCTATCATGGGGAAGAGGTATTTTGTTCAGTTCGTGGTTGTGGAGGAGTGTATCATTTTATATGTGTGAAGGAAAGGCTTGGGATCTCTAATCCAAGAAATTTCAAGTGCCCACAGCATGTAAGAATCACGATTTTCTACATTATGATATGCTTCTTTTCTCTTGGAGATTCCTATAATATACTGCTACTGCATTTGCAGATTGTTTTTGCACGTTTCCCAGTTGATTTTCTTGTGATATTTTATATATGATTCTGATGTAATTTTTATTTTATGTCACGAATTTGATGTCATTCCTGATAATTACTTGCTCGGTTACTTATGTATGGGGCATCTTGTTGTCAACAGGCGTGCTTCATTTGCCGTCAGAGATTACAATGGCGATGTGTGCGATGCACAATAGCTTCACATGATAAGTGTGCACCTTGGCCAGACAGAGTGATTCATTTGAAAGACCAACCAGGTCGAGCAGTTTGCTGGAGGCATCCTGCTAAATGGCTGCTGGATAAGCAGGTCAATTTCTTCTTTCCTTAGTTGGTATTGCATGCTTGTTCTTTGGCCTTTCTGACATCATGTTGTGATTCTTGTGGTTATAACCCTGGCCTGCCATTTTCCTATTAAAATCATAGAATGGCTATGAAGTGGCTTCTATTGTAAACTTATGATGAGCTCTTACTCCAGATGCCATAGCTTGATTCACTCGTAATATCAAATTGATAGTCACCTATGTGCAACTTCAAACTGAAATATCATTTCGCTACATTTGAGCCGCATTCTTTTTTGTCTCATTCTCTTTTTTGATTGGAATTGTTAGATTTCTTTCTCAACTACGATTTTTCAGATTTAGAATTTAGATATGCCATATCATGCGCTTGATGAACTTTGCGAGCCACACATTGACTGTTGGCTAAATTTATTAGACTTCACAAATCCACTTACAATTTCTTGGCAGGTGGTAAACTTGATCATAAGCAATCAATTCAGATTTTCACCATGCCTTTAAGTCTTTAAAATTAACTTCAGAGGCAATTTAATTGTTTGAACTGTTAAACTTAATATCATTATTTTTGAAAACTACTTGTCAATCTCTTGCTGTGATATTGCAATAACGAGATGAGAAATTTGGCATGTGATAGTTTTAATGCTTCCGCTGAAACTACGGTAGTTTTTTGTGAGGGAGAGGTGTCAATAAAGGATTGAAGCAAGATTTATATCAGCTTATCAGTCAATAATACCAATCCCATGAGATGTTTCCATTTGCAAAACTGTGCTTGATTTGTTTTTTAATCAGATTCCCGATCTCCTTGCGCCTGCTTTCAAGTTGTTGTCCAAGGGACATTTGCAAATATAGCAGAGTGGGACAAAATTCGTGTGCTAATCATATAATTTATATCAACACAAAATTGATGATAACTTTTTGTGGTACTGTTTGGCCATTAATATTTAGAATAATCTAGACTGCTCATGTGTACAGGCAAAGAGAATCAAATACTTTTTAGTGTCAGACATTGTAACTATCTTACAATCTCTCAACTGCTTTTGTTGAGGTTTTATTTCTCCCTTCTTATTCATTGATTAGGCTGGCTGACAGGACTTATATATTTGTTGAATGAGCAGCATGCAGAGGCAACACGTGACATAGAGGTCAGACTTTTTGCACACTTTTTCAGGATAATTGTTGCATCTTATATCATTTAAACTTATTTATATTAATAATTCAAGATACATGAATCATATATTAACTTGTCCATTTAGAAAAATGATGCTGTAGGACAAAAGGAGGCTAGGAGTAGCCAGTGACATGGCTCCCAAACCTCCCACTATCCCTTCTTTCTACATGACAATATGTATACTTAGGCAACTCTTTGAATTAATCCAACATTTAACTAACCACTATCTAGATCATGTGGGAAAAAAGAGGTGAATGAATTGATATCAGAAAGTTCTCTATATGCATTTTGTTTGCTTCTAGTTGTCATAATGTGGAATGAGACTTGTCAATGCCAGCTCTTCTAGATTGTAAATGTAAGAAGGCATATAACCATAAAAAATAGCTCTCTTTGCTGCTGTCTGCTTGAATTGGTGAAGTATTCATGACATTTTCTATTAAGTAAAACTTGATCAAGAAAATTAGTAATTTGTTTTACCTTCTCCTTTCCTAGCCAATCTTCTTCCAAAACTGTGCAATATCTACATTGACAATCTTTGAGATTGAATGCAATAAGAAAGGATATAGTTGTGAAGTGCATGCATGCTTGTATCATTTATCTTCAATAAGCTGGTCCAAATTTTGGAATATAAACTGTTGTAAATGTGCTCTGTACAATGGATTAGATCTTATACCTGTGTAGCAAACTTTTCATTTGTATATTTCACAGGCTAACCTTTTCTCCTGCCTAATTTTCTAGGAAGTTTTCTGCCGCTTGCCTCTACCTTATGCTGATGAGGAGTTCAAGATCGATCTCACATGGAAAGATCTGATGGAGAATAAAGTTGGGCCACCTCCATATGTGCACATTAAGCGCAGTATCCAATGAACACTGTTTATTATCTTGAACCAGTTGTGTTTGTTTAGTGTTTTCTGCATTCCAAGTATCTCTTACTTAGTGAGATGGATAACGGTTTCAATATTTTTGCAGATAATCATTTTTAATGCCCTTTAATCATGTGTTTAAGTTATCTGTGCGCTAACCTTAAATGACATTAGATATTTACTTGGTGAAAAAGAAGCGTGACAATTCTGATGATGATATTGGTTGCACGAGTTGTAGTTCTGAATGCTCTGAGAATTGTGTGTGCAGGTATGTCCTGCCTATCTTCTAATTAATAATCCCAAAAAATAGCTAAGTAGATGCTAATGAATATTTAAAAAAATTATCTTTGATTTTCTTGTATGAATCAGCAGTTAACAGATTTATTCATTAAAGAGTTTTGTTGCATTATTTAGCACCTTGCCTTGCCTTGTTCCAGAGTTCTAACATACAGCATATAATCTTCTAGGGTCCAATATATTAGCTGCTCGAAGGCTTGCCATTGCTCCGAAAGTTGCAATAACAGGCCATTTCGTAAGGAGAAGAAGATCAAAATTGTTAAGGTGAGAAGACCTGCTGTATGAGTACATCAATTATGTTCCTCTGAACTTGCGTCACATTGCTATGGGATGTTTCTTTTTAGTCTGCAAATTTGTAAATTCAGGTGTTTTTTATTTATTTTGGGAATAATTTCTGTTATCCATAAAGGTTAATTCTTGTCCTTTCCAGACTGAATTTTGTGGTTGGGGAGTGGAGGCTGCTGAACCCATCAATAAAGGAGAATTTATAATTGAGTATATTGGGGAAGGTAGTTTCAGTTCTTGACCTTTCTGCTTTTATATATATGTATGAACATATTGAAGAGTCCTTGTTCATTTTTTTTATCATTATCACACATATTAATTAAAGTTGTCTTTTTCTTTCAGTTATTGATGACGCTTTGTGTGAACAAAGGCTCTGGGACATGAAATACCGAGGTGTGCAGAACTTTTATATGTGTGAAATTCGGAAAGATTTCACAATTGATGCCACCTTCAAGGGGAATTTTTCTCGTTTTCTAAATCACAGTTGTGATCCCAACTGCATGTTGGAGAAGTGGTTAGTATGATTAATGTTTGCCACTTTTTTTATATTTTACAGTAATCAAGATCGGAGAAGTGGTTAGACAGATATCTGCCATGCTTATCCTTGTTGTTTTATATTGCATCCTATTTCTTTGACGCATTATATATAATCTATCTGCTGAAGATATTGGAATTAAACAGCTTGTGAGAGTTGAATTTTGCACATTATTTGAGTCTAACCATTTTAATTTGCTTTTCTTGAAGGCAAGTTGAGGGGGAGACACGGGTGGGTGTGTTTGCAGCACGATCAATTAAAGCTGGAGAACCATTGACATATGATTACAGGTACATGTAGATCTCACTTCTATTTGTCCAGTAACCCTGTTGTGATCATTGGCAATTAGGGCGACATTTGGCTTTAGCGGGAGAGTTGTTCTTGAAATATAACTTGTGCAGATTTGTGCAATTTGGACCTGAGGTGAAGTGCTATTGTGGTGCATCTAGTTGTCAAGGCTACCTTGGAACCAAGAGAAAAATTGGTAAGTTAGAGCTTTGCTGGGGTTCAAAACGCAAGAGATCTTCTACTGCTTGCCTAGCTATTATAACTCTATGATCTGTTTCAGCAGTCTATTCTCAGACACCAGTCATGTCGAATATTTGATGCTTTCATCATAGATTGGAAAAAAAACAAAGAAAAAAATTGGAAAAAAAAAAAAGAACATGGGGGCAAGAGAGAAGGAAAAGGATGTTATTCAACTACAGTTCAGCATAACCATTAATAAGCATTCAACAATTGCATAAGCTTTGGTGGTTGTGCTAACCCTGTTTGTAGATTTTTCCTTTGTAACATTAATAGAACACATTAATTATTTAATTCCCGATGTCGGGATCATTGTATTCTGCAATGTACACTCTTCAGCTGTTATACACGATGGGTGTGAAAAAATTCTTAGTCTGAATTGTTCAGAGATCTCGAACTGTTTATGTGGGTGGAGTTTTCGTGTTTTGCCAAAGTTAAAACTGCCAAGTCCTGTATTTGCCTAATGATAATTGTACATCAGCT

>CsSDG3

CTTTTCACTCGCCTTAAAGCATGAATGTGAAGATAACAATTTTATTTTTCATTTTTCCAATACATCACACTGCACTTTTCGGAGAGGCCAAAACCTAAATCATTTCCGATATTGACTGTCATGCAGTTAGATCATGTGCTTTCTTGACCGTTGATTCAGTCTAAATTCCAAATCCAGGTCTATTATTTACTTCTATATTTTGTGCTTCTGGTGGATTTTTTAAAAATTAATTTGATTAATTGGCTTCACTGAGAAAAATGCTGGAATATTGTGTGTTATTTAACTGAAGATAGCATTTTCGTTTTTTTTTTTCATAATTAGTTCAATTACTAGGTTTTAATCATTTTGATACTCGCATAATTGTTGTCTTCAACTTTTGTTTAGGTTTAATTTGATTGTTGTGATTATGAGATTTGTAGCTCAAATTCTGTGTGCTTACTGAGAAAATGCTGGAAAATGGCCTGAATTAGAGCCTGAATCTTTGTTTGTTCATTTGTTGTTTTATTATTTTTTAAAGCCTAGTTAGTTATATAATGACATATGATTTTATTTTCATGGATTGTTTACTGTCGATTTTTCTCATTTTCTTTCCATGTATTAGATTCTGAGCTTTGGGGTATGTTTGTTCTGTTTGGCTTCCTAGAAAACAAGGTAAAAGAAACTTTCGCAGCCATTTGTTTAGTAGGATATTACAAATGTAGACATGAGATACTGTTAGCATATTTAGTGTCGGCATCTTATTGTAAATCATTGAAGAATTTGGATCACTTTTGCATGAAGCAAGAAGAAACACCTGTGCTTTACTTGTGGAGAATAAGGTTCCATTATTAGAAGACTCCATCCGTTATAAGGAGATTTGGCATTTATTACACTCTTTATTAGGGTTTGACATGTAACATATAAAGGAGGAGTTTCACTCATTGAATTTAACATTGTATATTCTGTGAACTTGTGATTAAGATTTCTATGTATTCTTGAGTGGAGAAGTATTTTAGGTTTGCGTCATTCAACCTTCCAATGTCTATCTGAACTTATGATTAACATCTTAGGGAGAAGTACTTTAATTGCGCCTATTCTCTAAAACTCTATTGCGATTATGATCTTAGAGAGAAGCATTTTAGGTTTGCATCTATTCTCTAAACTCTATTGTGATTAAGATCTTCAGGAAAATTATTTCATGTTTGCTTGTGTTGAAGAGATGGAAGACTTGAGTCTTCAACCTTCCATTGTTTATTTTCTAAACTCGTATTGTGATTAAGATTTTCGGGAGAAGTATTTTAGGTTTGCTTGTGTTGAAGGGATTTCAAGGAAGCAGTAGACAATTTGCAACGATTGAAGGAGTTCAAGTTGCAGCAGACATTGAAGTCAAGTGGTGATTGCTAAGCAAGTTCATTGGGTTTGGTTTCACCAAGGGGACCAATACTCTTAATTGAATAAGTCCAAGAGGTTGTAATCTTTATTACTTAGTGAATTAATTAAAATTTAGGCATGGGTCGTTGTGGGTGTGTCCCCGTCCAAGGACTTAGGTAGTCGTTGTGGGTGGGGCCCTGTCCGTCAGGGGTGGGCAAACTCAGGTTTAGATAAACCCAATTGGGTTTCGAGTTAATCAGGTTGGGAAAAAGTCATCTGAACTCAACCTGAGTTTTTGTTCGGATCCAAGCCGAACATGAATTTAGGTTTAATTCAGATTGGAGTGGGTTAGGGTTAGTTATAGATATAGAAATACAATACATAGAACAATATCGGGTTTAGATGTCTTGCTAATTCCGAAGTCAAGTTTATTTAAATTCAAACAATAAACCCCATTTTACAATTTTGTTTCAAAATTTTGAAATTAATTATTTAATATAATATAGTCATATACCATGTAAAACTTGGTGATGGTGGCTGTCGAGGTGGAACAAGCTGCAGGCTGGTGGAGCAAGCGGCAGCAGGTAGATTGCATCGGTGCGTTGCGGTAGCAGCAGTTGCGGTGCGGCAGATTGCTGTGGCACTTTGTAGCAGCAGCGATGGCTTGACTTCGTGCAGTCACGTCGGTTACTGCTGGCTGCTGCTTTGCACGGTTGTGTCCCGATTTCTATGTGTGTGTGTGTGTGTGTGTGTGCGCGCGGCGGTGCGTGCCTGTGGCTGCTCAGTGTTTCCAACCTCCGGGGCCTGAGCTGCATGTGTCGTGTGTCTGTGTGTGGTGTACTGGTGTGAGTGCTGTGGTCATGTGCTCTGTGTGTGATACTGTGGTTTACAGATTTGGAATTTTGAGTTTTGTTTGTAACTTTGTGTGCTGCGGCTCTGCGTGTGTGAGTGTGAGTTTTGTTTACAGATTTAATTTCGATTAGGTTTACTGGTTTAGTTAATTTTATAGTTTTGTTTTAGATTTTTTTTTTGACCCAACCCAATCGGATCTTCAGATTGGATCATGTTGTAGTAAAGATCACTCAATCGGATTGCACAAGTAATCCGATCCAATCTGAACCTGAACCTGAGATCGGATCGGGTTTGTGCCACCCCTCCTGTCTGTGGACTTAGGTAGTGTTCATGGGCGTGGCTCCATCCGTAGGTAATTGTTGTGGGCATGGCCCTGCGGACGTAAGAAGTCCTCATAAATCTTATGTCCCCTTTATCTTTATTTTTATTTTTTATTTTTACTTTCAATAATTTTTTTAACTAAATTTGGCTGAGTTTATAAACCGATCCGACAGATACTAATCTCTTATGTTGTTTGACTCATGTTGTTTTTTGCTTTCATTTATGCAAGGATTGTTAAATGTTGTTTAATTTAGTCTTTATGGGACCTGGAGACAACTGTAATTCTGAATCTTGAGATTCTTAGTAGTCTGCTATATCTAGTTTCTTCATATAAGGCTTTCTTTAATCTGTTTAATTCTGCGAAGGCATTTGCTTAAATTTCTGTTTAGTTAATGCAAAAAATTTAGTACAATTGCATTGTTCACTATTCAGTTATAAGTTTGTTGTTACTTCTCTTTTCTTACATTCATCTTTTGGCTCATGGGAGTCTTTGCTCATTTGGTTCACCTTGTTGCTGTTATGCTCAATATTTAGTTTCTGAGTTATAATTGCTTTTTTTAATCTAGAGATTAATGATGATTATGTTTATTTTTCAGGTTGTTTTAATATTTCATGAGTTGGGATGGGGGTTATGGACAGTTTGCTGCAGACAGAGTCAGCTAGAGTTGTTTCATTACCTAATGGTAGTCATTCTGATGGTAGGTTGGGAAAAGCGCCTATGGAAAATGGCCACTGTGCTTCGCAAGGTGGTCCCAAGCATAAGCGGCGAAAAATTTCTGCTGTTCGAGATTTTCCACCCGGGTGTGGACCGTCTGCCTCAAGGATCAATTGGATACCTAATGAAGAAGCCATTGTTGGTGTACTTAGGCCTGATGCTGAAAATGTTGTTGTCTCATCTAATCATGTGGATATGCTGGATCTAGTTAGTGCTGACCCGAATGGAACATTGTTGCTAGACACTGAAAATGTAAACACTTCAGGAGGCAAAATGTATGATGGGTCAAAAAATTTGAACATGATGCATATTGGTGTTTCAGATGAAGAGATGGTTCTGCAAAGTGGTTCAAAGGCACTATCCTCACCTAATTCGCGAAATGCTGTTCCACATTTAAGTAATCTGGAGAGAATTTTGACTAGAAATTATCCTCCTAGGAGAAGAGTTTCTGCTATCAGAGACTTTCCTCCCTTCTGTGGGCAAAATGCTTCAGTTCTTGGTAAAGAAGAGTGCATGGAAGCACATCCTTCTTTTAGGAGTTCACCTCAAGAGGAGTCTGATTCCAAAGGCAAACCATTAAAAGAGACTGTGAAGACTGATGAAAACCAAATCAGAGTGAACGGTTATGATGGAGATGCTTGCATGAATGAGTTTGGAGGCGATGTTTCTAAAATCACCAGTGGTAAAGTTCTTGCTGATTTTGAAGAGCATGCTACCATGGAAACAAAGAATCGTGATGGCTTCGGAACTTCTAAGAAAATGATGACAGTTGCTCAGGAAGATACGGGTGAAATGAGTGTTGTATGTCCTCATGCAACTAAACGATACCGGTTGGATGGTAAGACTGGGGCACTCATAAAATCAAGCGAAAGGGATGTTGGAGTTTTGGAGGAAAATCCAGTGAGGGATATCGTGGTTTATGGAGAGCACAAACAGCTTGATGGAACCCGGTCAGATTTTTCTGTAAGTGACAATCAATTCCAAGAGGAAGATTCTGAAGGTTTACAGCTTGCATTGAACAGGGTTATTGTGCAAGGTCTAATGGCATCGCTGAACTGTCCATGGAGGTGGGAGAAAGGGGTTTGCAAACCTAACTATGTTAGTGGTACAGGTCAAAGGGAAAGAAAGAAGCATAATTCGCTGCCACCTTCTAAATCTCCTTCTGAGGAAATTATCAAAGCAAAAGGCTCTGAAGGATCATACTGTAAGAGGAATTCATATTCAGGAAGAAATGCCTATGAAAATAGGAGTGCATTGGTGATGAGGGATGGGAAAGATTCTCTTGGGCATGATAGAGGACAAGAAAACTTTCATCTGGGTCAAAGATCGCATGTTTTTGATGTGACCCTCCCTCCTCATCCCAGGAGTTCAAGTGGTAAAGGTCCTGAAAATGATGCTATTGGTGCCCGAAACAAGGTGAGGGAGACATTGCGTCTGTTTCAAGCTGTTTGTAGGAAGCTGTTGCATGAAGAAGAAGCAAAACCCAGCAGACAAAATTCTCATAAGAGGGTTGATTATCTAGCAGCAAGGATTCTCAAGGACAAAAAGAAATACATCCCTGTCGACAAAAAAGTCATTGGATCGGTTCCAGGAGTTGAAGTGGGCGACGAGTTTCAATACAGGGTGGAGCTCAATATGATTGGTCTTCATCTCCAAATTCAGGGTGGTATAGATTATGTCAAGCACAAGGGAAAAATTCTTGCTACAAGTATTGTAGCATCTGGGGGTTACGATGATAACTTGGACAACTCAGATGTTTTGATTTACACTGGTCAGGGAGGGAATGTGATGAACGGAGGCAAGGACCCTGAAGATCAGAAGCTTGAACGAGGGAATCTAGCTCTAGCGAACAGCATACATGAACAGAATCCTGTAAGAGTGATCCGTGGTGATACAAAGGCTTTAGAATCTAGGACGTACATTTATGATGGTCTGTATTTGGTGGAGAGATATTGGCAGGATGTGGGCTCACATGGTAAGCTGGTTTTCAAGTTTAAGTTGGCCCGAATTCCGGGTCAGCCGGAGCTTTCTTGGAAAGTGGTGAAGAAGTGCAAAAAATCTAAAGTACGTGAGGGTCTATGTGTTGATGATATCTCACAAGGAAAGGAGTTGATTCCCATTTGTGCTGTGAACACTGTAGATGATGAAAAGCCCCCATCATTTAAATACATAACCAATATTATATATCCTGATTGGTGCCGTCCTGTTCCTCCAAAGGGTTGTGATTGCACTAATGGATGTTCAGAGTTGGGGAAATGTGCTTGTGTGGCTAAAAATGGAGGGGAGCTTCCTTACAACCACAATGGGGCCATTGTTCAGGCAAAGCCCCTTGTCTATGAGTGTGGTCCTTCTTGCAAGTGCCCTCCTTCTTGCTATAATAGAGTCAGCCAGCAGGGTATCAAATTTCAGCTTGAGATCTTCAAAACTGAAGCACGGGGATGGGGTGTGAGATCACTAAATTCCATTCCTTCAGGAAGTTTTATCTGTGAGTACGCGGGGGAGCTCCTTGAAGAGAAGGAAGCGGAAAGAAGAACCAGTAATGATGAGTATCTATTTGATATTGGGAATAATTATAATGATGGTTCTCTTTGGGGTGGACTTTCGAATGTTATGCCTGATGCACCATTGAGTTCTTGTGGAGTTGTGGAGGATGGTGGATTTACCATTGATGCAGTGGAGTATGGCAATGTGGGGAGATTTGTTAACCACAGTTGTTCCCCTAATCTTTATGCCCAAAACGTCCTTTATGATCATGAGGACAAGAGAATGCCTCACATAATGCTCTTTGCTGCTGAGAACATTCCTCCCCTGCAAGAGCTGACTTATCATTACAATTACGTGATAGATCAAGTTTATGATTCATCTGGCAACATTAAGAAAAAGAGTTGCTTTTGTGGTTCTTCAGAATGCACTGGTCGGTTGTACTGAGGGAGGTTAACAGTAGTAGTTTTGATTTTCATCTGAAAGGTACATTCAAGTGCCCTGTTAGTTATATCACTTGTTCCAAGTCCTAGTATCTTTCTGGCTCACCTATGCTCACAAATATGAGGATGTATGTGTCTGTCTGTATGTACGTCCATGAAATTGCATTATGCTGTATTTGTTTGTTGGACAATACATTTAAATTTAAGTGCCCCGTTAGTTTTTACGCCTTACTAAAAATACACCAAAAATTTAAGCCCCTTGTTAGTTTTTACGCTTTAATTTAAGTGCCCTAATATTCTACGCTTTATTAAAAAT

>CsSDG4

ACGAAATATTATTCTTCTTCTTCCGAAATTAGGCTGCAACTTGATGCTAATTCGTGAGAAATCATACTGCAACTTGATGCTAATTCGTGAGAAATCATGTATTCGAATCAAGAATTAATGCCATTGTTTCCTTCCGAGAATCTACAATGCGAAGAATTTCCGTCGTACCAACATATTTATCAAAACGAGTTCCTCAGTCGAAAGTAAGTTCAATTTTATTTTATTTTTTGCTTTTTCAATCGAATTTTATATTTCGTAATAAAATTTTATCAGCATATATGTATTTTTGTTTTTCTATAATTATAGGCACAAGAAGCAGAAGGAAGAGGATATTGCTATATGTGTATGCAGAGTTGATCCAAATAATCTAGAAAGTTCTTGCGGAGAGAGATGCTTGAATGTATTAACAAGCACTGAATGCACTCCTGGCTACTGTCCCTGTGGCGTTTTTTGCAAGAATCAGGTATATATTCAGCGGCACAATTGTGATTGAAATATTATCTGTTCAATTTAGAGAATCAAGTTTTTCAATTGACCATAATTTGGGATTGTGTTTGTAATTAAGGTGCTATAGAATTTAATTCAAAGCTGTTGTGAAATAGAATCACTAGTCTTTGAACACGTTTTCCCACTGGATGAGCTCCGTTGTTTCTCGAGATTCATTGTCCTTTTTTTTTTCTCTTTTGGTGGGCGTAAATTGGATGTTCTTTATTTATATGTGTTACAGAGATTTCAGAAGTGTCAATACGCTAAAACGAAGTTGGTTAAAACCGAAGGCCGCGGGTGGGGTCTTCTCGCTGATGAGAATATAAAGGTAGCACTTATCAAGCTAGAACAAGTTTGTGTAGTAATCTTTTGTGTTTTTGTTAGTTTTTGTATTCTTTGCTATTGTAAGCCGCACTTCATAAGATCATTCTGACCATCTTGGTTTGCTATTTATTGCAGGCAGGCCAGTTTATTATTGAATATTGTGGTGAAGTAATATCATGGAAGGAAGCGAGGCGAAGATCACAAGCCTATGAAACTCAAGGTAGAAAGTATTTTCATCAGATGAAAAGTTATAATGCTCTTTTGATTTCTTTTTCTTTTCTTTGAAAACCAGATATGTATTTTTAATAGCTTGATGCAGGTCTCAAGGATGCATATATCATTTGTCTCAATGCCCTTGAATCCATTGATGCCACTGTAAAAGGAAGCCATGCTAGATTTATTAATCATTCATGGTGAGTCACTTAAGTGAGGGCACAAATAGGGTAAATAATCAGTATTTGCTGTAAATAACTTCATAGGCACCTTATTGATAGCAGTTGTTCAAAGTTGACGTGACTGATTCTTCTTCTAGTTTGCAGCCAACCCAACTGTGAGACAAGGAAGTGGAATGTTTTGGGGGAAATTAGGGTTGGAATATTTGCAAAGCAAGATATTCCTGTTGGAACTGAACTGGCATACGACTATAATTTTGAATGGTATGGAGGCACCAAAGTTCGCTGCCTCTGTGGTGCAGCCACTTGTTCTGGATTTCTTGGAGCAAAGTCTCGTGGTTTCCAGGTAAATCGTGAACGACTGAGATCCATAGTCAATTTGAGTTTGAAAGAAAGATGTCTGACCACGATACTGCTTTAACAACTGAGGTCATGGCAAATAAAATGGACAGGCATCAAAAGTTACACAGTAAGAATGGATATGGAAACAGTTTTCAGTATTCGATAGGACATAAGGTTTAAACAAATCAAATAACTAAAACGAGATGTTACAATTGACAAACTTGCCTGGCAATGCCTGGCAGTGATACAAGATGTTTAAATGGGGAAAAGTTGCAGAATTGGACTGGTCTTTGGATGTCATTGGAGGTCAGCACAATAGACAGTTTACTTAGTTACTTAGGCCTTTTTTGATGCCTGAAGCAATGCATTTCAGAAACCAAATGTTCGTATCTAATGACCAATTTCATGGTTTAGCTTGCATTGGCTTATCCAATTATTTCAACTCTAATGTGATGTTGATGTTGCTTTCTTGTCTGATGGACTAGGTGTTCTAACAATTCTAGATTATGTCTACCAATTCCACTTATGCTTTCACACTGTGTTTTCCTCTTCCTTTTGTTTTATTTTGGCTTTTATTAAATAAAAAATGTTGTGTCTTTCTATTGATCTTGTTGTAATAATCTAAGTTCTTGTCCATGTTGTAACAGGAGGACACATATCTCTGGGAAGATGACGATGAAAGGTAATTTGCTATCCTTATTATGCATTTTTATGCAACTTAAAGTTTCCAATATTTTGAGCTAATGGGCCTACTCCCTACTGGATTATTTTGAGCTATCATTCAGAAGCTTCTTTTTCTTTCCCCTCTCACTTTGTCATCAATTTTCAAATTATGTTTGAAATTCAATTCTCTACTTGAAGTCACAAATATGGTATGAAGTATGGTAAATGAACCTAGCCTGCCGTTGCTGGTTGAATTTGATCTGCCCAGCGACTTGGCACTTTTGAATTTGGAATGTGTTAACTCCTTGATTTTGGTCAAGTCTTTGACAATACCATGTGGCATGGGAGTCAGCTTTGATGAATGATGGTTTGTACACCAAGTTTATCTTTGAAGAAAACCGTGAATTAGTTCCTCTTTTTCTTACTTTCCAGAACAGTATGTACAGGGAATATATACAGCAAAATAGGAGAGAAAATAGGAGACTAATAAATCCTAACTTTGAATCTTTATTTTCAACACTCCCCCTCAAGCTGGTGTGTGTATATCATACAAACCCAGCTTGTTACTTAAGTCTTCAAAGTTAGGTCTTGGCAGTGCTTTAGTAAGGATGTCTGCAATTTGTTGTCTAGTTGGAATGTACTTTAGTTCCACACTTCCACTAGTGACCTTCTCTAAGATGAAGTGCCTATCAATCTTGACATGTTTGGTTCGATCATGATGCACAGGGTTTTTAGCGATGTTGATGGCTGCTTGATGGTCACACCTCATTAGAATGGATGAAGGGCTGCACACACCGAGTTCCTTGAGTACACGCTTTATCCAAATTCCTTCACATATTCCATTTGCCAAAGCTCTGAACTCTGCTTCTGCACTACTCCTAGCTTCTGATTTCTTGAAAAACAATCCCCAGGTTACTAGATTTCCCCAGACATAGGAACAATAAGCAGATGTTGATCTTCTATTTGTGACGTCTCCAGCCCAGTCTGCATCGGAGTAGATCTCAATATTCCTATATTCTGATTTCTTGAAAAACAATCCTTTTCCAGGTGTCATCTTCAGATACTTCAGAATTCTAAAGACTGCATCCATGTGTTCCTCTGTAGGATTGTGCATGAATTGGCTCACAGCACTAACAGCAAAGCCTATGTCCGGACGGGTATGTGAGAGATAGATTAAACGACCAACCAGCCTTTGATATCTCCCTATTGACTGGAACACTATTCTCAACTGAGCCTAGTTTCTTGTAGGGATCCATAGGAGCAACTGGTTTGCACCCAATCATACCAGTCTCCTTTAGTAGGCCTAGGGTGTATTTCCTCTGGGAGACAACAATTCCCTTTCTAGATCTTGCCACCTCCATCCCAAGAAAATATTTGAGATTCCCTAGATCTTTAACTTCGAACTCCTGAGCTAGCTGTTTCTTCAACTTCTGCAGTTCTTCCTCATCATTTCCTGAGAGAATGATGTCATCAACATATACGATTAGGAGAGAGATCTTCTTTGCTTGTGATGTTCGGAGGAATAGAGTGTGATCTGCCTGACACTGAGTGTAACCAAGTCTCACAACTGCCTTCGCAAATCTATCAAACCATGCTCGAGGGGATTGCTTAAGCCCATATAAAGATTTCTTGAGCTTGCACACTTTATTTCCAGTAAGTTTCAGATCAAGACCCTGGGGAACTTCCATAAACACTTCTTCTTCAAGATCGCCATTGAGAAACGCATTTTTTATATCTAGTTGGTATAAGTACCAATCTAGATTTACTGCTAAGGAGAGCAAGATTCTGATGGTGTTGAGCTTCGCAACTGGAGCAAAGGTTTCAAGATAATCAATCCCATATGACTGTGTGAACCCTTTAGCTACAAGACGAGCTTTGAGCCTCTCAACACTTCCATCTGCCTTGTATTTAACAGTAAAAATCCACTTACATCCAACTGGTCGTTTCCCTGCAGGAAGATCTGTTATAACCCATGTATCATTTTTCTCAAGAGCACTTTATTTTCAACACTCCCCCTCAAGCTGGTGTGTGTATATCATACAAACCCAGCTTGTTACTTAAGTCTTCAAAGTTAGGTCTTGGCAGTGCTTTAGTAAGGATGTCTGCAATTTGTTGTCTAGTTGGAATGTACTTTAGTTCCACACTTCCACTAGTGACCTTCTCTGAGATGAAGTGCCTATCAATCTCGACATGTTTGGTTCGATCATGATGCACTGGGTTTTTTGCGATGCTGATTGCTGCTTGATTGTCACACCTCATTAGAATGGATGAAGGGCTGCACACACCGAGTTCCTTGAGTACACGCTTTATCCAAATTCCTTCACAGATTCCATTTGCCAAAGCTCTGAACTCTGCTTCTGCACTACTCCTAGCTACAACTGATTGTTTCTTACTCCTCCAGGTTACTAGATTTCCCCAAACATAGGAACAGTAAGCAGATGTTGATCTTCTATTTGTGACGTCTCCAGCCCAGTCTGCATCGGAGTAGATCTCAATATTCCTATATTCTGATTTCTTAAAAAACAATCCTTTTCCAGGTGTCATCTTTAGATACTTCAAAATTCTAAAGACTGCATCCATGTGTTCCTCTGTAGGATTGTGCATGAATTGGCTCACAGCACTAACAGCAAAGCCTATGTCCGGACGAGTATGTGAGAGATAGATTAAACGACCAACCAGCCTTTGATATCTCTCCCTATTGACTGGGACACTATTCTCAACTGAGCCTAGTTTCTTGTAGGGATCCATAGGAGTATCTACTGGTTTGCACCCAATCATACCAGTCTCCTTTAGTAGGTCCAGGGTGTATTTCCTCTGGGAGACAACAATTCCCTTTCTAGATCTTGCCACCTCCATCCCAAGAAAATATTTCAGATTCCCTAGATCTTTAACTTTAAATTCCTGAGCTAGCTGTTTCTTCAACTTCTGCAGTTCTTCCTCATCATTTCCTGAGAGAATGATGTCATCAACATATACGATTAGGAGAGAGATCTTCTTTGCTTGTGATGTTCGGAGGAATAGAGTGTGATCTGCCTGACACTGAGTGTAACCAAGTCTCACAACTGCCTTCGCAAATCTATCAAACCATGCTCGAGGGGATTGCTTAAGCCCATATAAAGATTTCTTGAGCTTGCACACTTTATTTCCAGTGAGATTCATATCAAGACCCTGGGGAACTTCCATAAACACTTCTTCTTCAAGATCGCCATTGAGAAACGCATTTTTTATATCTAGTTGGTATAAGTACCAATCTAGATTTACTGCTAAGGAGAGCAAGATTCTGATGGTGTTGAGCTTCGCAACTGGAGCAAAGGTTTCAAGATAATCAATCCCATATGACTGTGTGAACCCTTTAGCTACAAGACGAGCTTTGAGCCTCTCAACACTTCCATCTGCCTTGTATTTAACAGTAAAAATCCACTTACATCCAACTGGTCGTTTCCCTGCAGAAGATCTGTTATAACCCATGTATCATTTTTCTCAAGAGCACTTATTTCTTCATGAACGGCTCTCTTCCACCAAGGATGACCGAGAGCCTCTTGTATATTGTTGGGAACATGTAGATTATCTAAAGAAGAAACAAAGGCTCGGTATGATGAGGACAAACCATCATATGAGACATATTTGGCAATTGGATGAGTGGTGCAACTTCGAACCCCCTTTCTTTCTGCAATTGATTTATTAGAATCATCAACACTTGGAACCATGAACTCAACCGAGGCAAGACTATCCATACCTGTGTTGTCTTCACAAGAGCTTAGATTTGGTTCAGTTTCTTGGACATGTGGCTGAGTTCCGTGCTCTATCTGCTCTCTAGTTCTTCTCCTCCTATAAACATTGAGATTATCAACAGCAAGCTGTGGACGAGGTGAGATTAGAGGCTGTAGTTGATGAGCTGATGGATGACGGGGTGACACAGCTGATTGTGGTTGGGAGGACTTCTTGGAACCATGAACTCAACCGAGGCAAGACTATCCATACCTGTGTTGTCTTCACAAGAGCTTAGATTTGGTTCAGTTTCTTGGACATGTGGCTGAGTTCCGTGCTCTATCTGCTCTCTAGTTCTTCTCCTCCTATAAACATTGAGATTATCAACAGCAAGCTGTGGACGAGGTGAGATTAGAGGCTGTAGTTGATGAGCTGATGGATGACGGGGTGACTCGGCTGATTGTGGTTGGGAGGACTCAGATGACTCAAAAGGTTGTGAAGATGTTGGGAGGATTGCTGATTGTGGCTGTTGGATAGATTGATCATGTGTGGTGGTTGAGTTATGAGGGTTTTCATGAGGGGTTTCCAGCCAAGATTGCCAAGAAGATTCCCAAGGCTGATGTTCGCTCGTACTCTCTCCCTGAACAGCAGTTTTGGAATAGAAAGGCAGGTTTTCAACAAATGTTACATCTCTGGAAATGTATATTTTTCTGGTGTGAGGAGAATAGCATTTGTATCCTTTTTTATGTGGAGAATAGCCTAAAAATATACACTTGAGAGATCGGGGATCAAGTTTAGTCCTGTGTTGAGAGTGAACATGGACATATGCTGTGCAACCGAATATTTTTTGTGGTAAATCTGCTGAAACAATCCTAGTGTGTGGAAAGAAATTGAGAAGAACTTTTCTTGGGCAATGAAACCCCAAGACTCTAGAAGGCATTCGGTTGATTAAGTAGGCTGCAGTGAGAACAGCTTCACCCCAGAAAAAATTAGGAACATGAGATGAGAACAAAAGACAGCGAGCCACTTCAAGAAGGTGTCTGTTTTTGCGTTCAGCGACCCCATTTTGTTGCGGGGTGTCAACACAAGAACTGATGTGAAGAATGCCTTGATGGAGAAGATATGAGCTGAGGGAGTCTTTGAAATATTCCTTGGCATTGTCGGTTTTAAGGACTTGGATCTTGGTATTGAATCGGGTTTGAATCATGGCGTTAAAGTTTTGGAAGATTTGACTAACTTCGGATTTGTTTTTCATGAGAAACACCCAAGTGAGACGAGTATGATCATCCACAAATGACAAAAACCAGCGAGCCCCAGAAACATTTTTAATTCTTGAGGGACCCCACACATCACTGTGAATTAAAGCAAATGGACGAGAAGGCTTATACTTAACNNNNNNNNNNNNNNNNNNNNNNNNNNNNNNNNNNNNNNNNNNNNNNNNNNNNNNNNNNNNNNNNNNNNNNNNNNNNNTATTGGACTTGGCACAGTTTGCTCTCGAATCTTGTATGAAGGAGGATTGATCGCTTTTGAGTAGATAGAGTCCAGAACATAGCTCAGCACTGCCAATCGTCTTCCCCGAATCCAAGTCCTGAAAAACACACATATTTGGATAGAATTTAGTTACACAATTCTGATCACGAGTGAATTTACTGATTGAAAGTAAATTGCAAGCCAAATTAGGCACATAAAGTACAGAATTAAGGTGTAACTCCTTAGTGAGTCTGATGGAACATGTCCCTGCTATTTTTGTGTGAGAGCCATCTGCGATCCGGACTGAAGTGTTGTTGATGCTAGCTTTATAATCCTGCAACATTGAGACATCTCCGGTCATGTGATCAGAAGCACCAGTGCGTACATCCGCCTTGGCCGTACAGCAGGAGGTTTCCAATCAGCAGGTTTGCCATAAATTTTCCAGCAACTCTCCTTATAGTGGCCTGCCTTACGACAGTGATCGCACCATGGTCTTTCTTTCTTTGTACGATTTTCACTAACCCCTACAGAGTTAGATGCACGGGCAGCAAGTGCAGAGGCGTCCAGTATTGGTTCTGATGACCCCATCATCACCTTTTTCCTGCTTTCTTCACGCTTAACTTCAGAAAAGGCTTCACGAGGGCGCGGTAGAGGCTTTGTCCCGAGCACACGGCTCCTAACCTGATCAAGATCTTTATTGAGTCCGAGGAGAAATTTAAACGTTCTCTTCTGTTCCACAATCTTCTTGTATAAGGTTGTATCGTCGTTCTCTTCTGTTCCACAATCTTCTTGTACAAGGTTGTATCGTCGGTGCACTTCCAAGGGTATGTTTCAAATAGATCAAGGTGTTGCCAATGCCTAGTCAGAGTATTAAAGTACTGAGTGACAGATTGTTCTCCTTGGCGGAAGTCATGGAGGATGGTTTCAATCTGGAATAACTCTGATGTGTTCTCAGAGCTAGAATAGGTTTCCTTTGCAGCATCCCATATGTCCTTTGCAGTCCCAAACAGAAGAAAATTCTCACGAATATCATTGTTCATGGAATTGATAAGCCATGACATGATCATGTTGTTCTCTGATTTCCACCTTTTGAAACTAGGATCTGAAACCTCTGGCATGGCCTCCTCACCGGTGAGATAATCATCCTTACCCCTGCCACAGATGAACATCATGACAGATTGTGACCACTGGAGATAATTGTGGCCGTTCAATTTATGACTGGTAATTAAGATCGGTGAAGAATCAATTCCACCATGAGTTGCAGTCTCCACGTTTCCTTTAGACTGTGATATTGTAACGGATGTTGATGAAGAAGCCATCCTGTATTTTGTCACAGAGAATACAACTGATGGGAGAGGAAGGAAGGTGGATACTGTTGAAAAGAGGTGGTAGAGGAGAAGAAGGAAATGGCGGCTGATAGGAGAACTAGGGTTTAGAACCTGGCTCTGATACCATGAAGAAAACCGTGAATTAGTTCCTCTTTTTCTTACTTTCCAGAACAGTATGTACAGGGAATATATACAGCAAAATAGGAGAGAAAATAGGAGACTACCTCTATCCTTTCCTAAATAATAGGAAACAAAATCTAGAAAGAATCCTAATGAATCTTCCCATTATAATTTAACTTATTCCTAAAATAGCCTGGATTACAAATAAATCCTAACTTTGAATCTTTATTTTCAACAATCTTTCTGTGACATATTATTGCTTCAGTTAGTCACCCTTGTGAATTTGATGATCGAGAGATTTGCACCAGGCCTGTATAGAGGGATGAGATGAATGTTTTTCTCATCTTAAAATGGATATCTGGTCTTGCTTTAACAGGGCACTGGGAAGAAAATAGTGGACAACGCTGAACATTGCTAATTAACTAGAGATGGTGTAGATTGTTATACCTGGTTTATCAAGCAGACCTGGAATATGATATTAATTTACCACCTATGAAATAATTAATTTGAAACCAGAAACTGCAATGAGATTAGATACCACCAGTTTTAGATTTTTGGTAGATTTCTATTTAGGTGAAAGGTCTTTTCTTTGTTCCCCACAGAATAAAGGCCATGCCATCAGGATATTAGGTTGAATAGTGTGAAGTTCTGGGTCTCTTAAACTACCAAATCTCTGGATGTGGAACTTTTTTGTTTTGTTTCTTGTTATTTCTGGAGAAAGCAACTGGAAAACAGGAATAATATAGTGATGTTTAGGATATGGGTGTGCATCTAGAGAAAAAAGTCAAACTTTTTGTTTATGATATTGAGAGACTGTTACTAGCTGCAAACTGTTTGTAAGACGCTTCTTGAGTTTTATCTTGATCATACTTCTCTCAAATATTGCTCATTTTTCACAATTATATTAAGTTAACCTGTCTTTTTGTTTTGGTACAGGTATTCAGTGGAAAAGATTCCACTCTATGATTCCGCTGAAGATGAGCCTTCTTTAACTCTTTTCAAAACTGTTGAAACAACCAAAACTGAATATGTTGTTGATGGGAAAGAGGAATACTCCATGGGGATGAATGTTAGTGTGAAGCCGGAGAATCATTTGGATTCTACTTCTCTTGTTGTTCAGCCACTTGAATCTGTTCCAATGGAAGGAGTAGTTGTGAATGCAATTAAAATTGAAGAAAGTGAAGAGACAAAATTGTATCCCCAAGATACTCAACAACAGGTCTTTTCACAAAATAATGCAATGATATCTCGTATCCGAAGTAACAGTGCATGCCGGAATTATCATATTGGACCGGAGTCCATGCCCAAGAAAAGATCACAGTTGAAATCTAATGGAAAGTTGAAACATCTTGCTCAAAAGCATGTTGATGCAAAACATGTATGTCAGCTTTTAGCATTCAAGGAAGCTCAAGAGGAGATTCTGAGAAACGAGGTATGATTAGCCTTTCTATACAATTATAGATAGCTTTATGCTAGCTCGTAGGGATAATCTCAGCTTTGCTCATTTGAACTTCAAATTCCCATTTGCACTTGCATATGAGTGTGTACATACAGTCTTGTCCTTGTATTGTTTGGTGGCTTAATCTGGACATGCTGAAACGCGATACTCATTACACTTTTAGGACAAACTGCTGAGACAATATTCTTTCTTGGTATTTGTGTTGTAACTCTAGATAACTTTTGGCTTTAAAAGCCAACATAGTATTTCAAATTTTCGTTCAGGGAAAAAAATTGAAAATTAAGCCTAAGCGATTGGAATTTGACAAATCTTGAGTTAAGTTGAAATCGTTGTGCAATTAGAGTTTCGTGTATAAAAGCTTTTCTTTGTGAAGAATTCACATTATCATATTTCTCTCAGCATCTTACTGTCCTAATTGGTTGCACTTAAGTTCCTTTCAGTTAGTAGTTTGTTTAGCTTTGTGGTAACTGGTATACCATATTTGACTGCAATTCAATTTTAGTACACTTCTATTGCTCTGCTTAGGTATTAGTCATCTTCAGCACTGTCACTACATTGATGGATGTTTCATCCACTGCAATTCAATTTTAGTACACTTCTATTGCTGTGCTTAGGTATTGGTCATCTCAGCACTGTCACTACATTGATGGATGTTTCATCCACTTGCTCTCATTTTCCAAGTTGTGTGCAGGAAATGAAGAACGAGGCCTCCTCTCAGCTTGCTTCTTTGTACAATGACATACGCCCGGCCATTGAGGAGCACGAGAGGGACAGCCAAGATAGTGTTGCTACAAGTGTGGCTGAGAAATGGATCGAAGCCTGCTGCACGAAATTGAAGACAGAGTTTGATCTATATTCCTCAATAATCAAGAACGTGGCCTGCACTCCAACAAGAAAACCCAACCAAGCAAAAGCTTGTGATGCAAACAGTGTAACAGAAGTGAAGTATTTGGGATTTTGAAGATGAAGTAACTTTCCATCTTCTGAATGATGTATTAGCATTTCGTCAGATTGCCATTAACTTAGAAAATTTCTTTGAATTTTGTCCATTTGTTTGGAGTAAAGTCGATGGAAATTTTTTTGACTAAGAATTTCAACTCTTAGTTTTCTCACCTTGTGATGCTTTATTGTGTTTTTTCTCTAAATATAATGAAACTGACAAGGCTGGTAAA

>CsSDG5

AGTGTAAGAACAAGTTCGAAGTGGACGGACTAGAAAAGGTGAAACGCATTGGGCGGAGAGCAAGCAGGCTTTTCATTTCAATGGACTTAGCAGCCAAAGTCCCCCCATTTCTCAGTTCAAACCCTAGCAACTAACGGTACATGCAATCTCCAGAGGTAATAATAATACTCTTAATTTTAATCTGAAATTTTTTTTTTTTGTCAGTTCGGTAAGTTAGATCTTACAGTTTAAGTTGCTTGTTCTTTAGGACCATTTAAAACTTGTTTGATCTTTATTTTTTGCAGAGAAAAAAAATTAAAATTGAGCTTCTTTAAGGAAATGGGATTGTGAAAAAAGAAAGATTTAGTGCGTTTAATTCTGGGTTTGTTGAATTTAAGCTTTGTTTAGTGTGTTTTTTGAGTTAGGTTGTTTTAGTGATGGATGGCACTGTTCCTTCAGCTCCTTTAGACAAAACAAAAGTGTTGGATGTAAAACCTTTGCGTAGTTTGAGACCTGTGTTACCGTCAAGTCCTCAAGCACCTCCTTTTGTTTGTGCACCTCCTTTTGGCCCTTTCCCACCTGGGTTTTCTCCATTTTACCCCTTTAGTACGCCGGAATTTACTCCTGACAACAACCAAAACAACAACACACAGACACCTCCAACGTCGTTTGCTACCCCCATTCGGTCATTTAGGTCACCGGATGTTAATTTTGTGGATGGGTCTAATGGTGATTTGGGGTCTTCGGATGGGTTTCTAGACGGGAAAAAACGTCGTGCTTCTAGTTACAAGCAGAAGAGGCCCAAGAATGCTCAGGACTCGGATTTTTCTGTGGGGATTAGTTCATTTGAGCGAGATGATGGTAATAGGCAAGTTGTTAATAATGTGCTGATGAGATTTGATGCCCTTAGAAGAAGAATTAGTCAGATTGAGGATGCTAAGGAGACTTCTACTGGGTTGATTAGGCGTGCGGATTTGAAAGCAAGCAATATATTGATGAGCAAAGGAGTACGTACCAATATGAGGAAGAGGCTTGGAGTTGTTCCAGGTGTGGAAATTGGTGATATTTTCTTCTTTCGGATGGAGATGTGTTTGATCGGTTTGCATTCCCAGTCCATGGCCGGAATTGATTATATGATTACAAGGAGTGATTTAGACGAAGAACCAGTGGCTGTAAGCATTATATCATCCGGAGGATACGATGACGATGCTGAGGATAGTGATATATTGATATATAGTGGCCAGGGTGGGAATGCTAATAGGAAGGGTGAGCAAGCAGCTGATCAGAAGCTCGAAAGGGGTAATCTTGCATTAGAAAGGAGCTTGCGCCGAGCCAGTGAAGTAAGAGTCATTCGGGGAATGAAAGATGCTATTAACCAATCTTCAAAGGTCTACGTATATGATGGACTCTATATGGTTCAAGAGTCGTGGACTGAAAAGGGGAAGTCAGGTTGCAATATTTTCAAGTATAAATTGGTTAGAATACCTGGGCAGCCTGGTGCTTTTGCTCTTTGGAAATTGATTCAGAGGTGGAAGGACGGCATGTCTGGTAGGGTTGGACTTATTCTTCCAGACCTTTCGTCTGGGGCCGAGGCTATACCCATTGCACTTATAAATGATGTTGATGATGAGAAAGGGCCTGCGTATTTCACTTATCTCACCACTGTTAAGTATTCTAAATCATTCAGATTAACTCAGCCTTCTTTCGGCTGCAACTGTTATAGTGCATGTGGCCCAGGCAATCCAAATTGCTCTTGTGTCCAGAAAAATGGGGGCGATTTTCCATATACTGCTAATGGGGTCCTAGTAAGTCGGAAGCCGTTGATATATGAATGTGGTCCATCATGTCCATGCAATCGCGACTGCAAAAACAGAGTGTCTCAGACTGGTCTAAAAGTTCGGTTGGATGTGTTTAAAACGAAAGATAGGGGTTGGGGCCTGCGATCTTTGGATCCTATTCGTGCTGGTACTTTCATTTGTGAATATGCAGGTGAAGTAGTAGATAAATTTAAGGCAAGGCAAGATGGGGAAGGCAGTAATGAAGATTATGTTTTTGATACAACCCGTACTTATGACTCATTCAAGTGGAATTATGAACCGGGGTTAATTGAAGATGATGATCCTAGTGACACTACTGAGGAATATGACTTACCATATCCCCTAGTCATAAGTGCCAAGAATGTTGGGAATGTTGCTCGATTCATGAATCACAGTTGCTCTCCAAATGTTTTCTGGCAGCCGATTATATTTGAAAACAATAATGAATCCTTCGTCCATGTTGCATTTTTTGCAATGAGACATGTTCCTCCCATGACAGAGTTGACATATGATTATGGGATTTCCAAGTCTGATGGGGGTAATTATGAACCCCACAGGAAAAAGAAATGCTTATGCGGAACACTGAAATGCCGAGGTTATTTTGGATGATGTTGAAAGCGTGTTGATTAGTGGAAAAGAATGGTATTGCCAACCTTGGCACTGTAAAAGGTCAGTATCTCACTTATGTTGTTTTTCATGAACTTGTTACCATGAAATATGATGTTTTCAGAGATTCTTATGATTTTTGTTATTGTTGATTTATTAGACAACAAATATGAATTATTTATGCCAAAATTATGCTAGTAATTTTATCCATGTGGCACGCACGCATGCAGAAGTTGTTAGTTGGGCCACATATTGTTGTTATAAATATGAATTTTCTCAAGATAATGTTTTATATGATATTGTTGGAAGCAACAAATCTTTGCTTGTTCATGAATTGCTCTTCTATGCAAACTGATTCTTTGTACGGCGGACAGACATACCTTCTTTAAATATACAAAGTAATTCATGAAGCTCAAGTATTACTTTTGTCATAATAGATAATACTTTTTCTGTTTGATTATTTTTATGACTGAATTTCTGCTCATAATAATTTGAGCGTATTGTTATACTGCCAGACTCATTAGACATTTTGTTATATTTCGTGTTAGGCTGAAATTTTGCATCTTCTAGTCAATATTGAAGCCATTTTGATTGATCTATTGTCTTATTTCTCCAATTCATTGTTATACTACCATTTTGATTGATCTATTGTCTTGAAGAAGCTTCTTCTGCCTTTTTTGGGCCCCCAAGATGCACTGAATGTATCTACAAGAGAGAAAATCTCTCGATTTCTTATCTGATTTCTTATCTTCATGTATATCATTGACTAATTAACTTTTCATACAATTCTTAGGATGTAGTGGTCAATTCCTCCTGTTGATGAAGAAACTCTGCCTGCCGCCAGCTATTGGGTGCAATGTCACCAGTTAAGTAGGCACAAAACTCTGTATGTTGTCCCTAAACCTATAGCCAGGGAGGACACACTGAGGCTGTTGACAGTGATCTCATGGAGCACTTTGCTGGTCACTTTTTATGATGTTTGTAGTTTTTAGTTATTGTAACTAGTAGTTGCACTATTGTAAGAAACCGGCTTTGTGTACTACCTTTTTTCTTTCCCCCCTTTTTTATCTCTGCCGCGGCATAGTTATTCTGCTTTTCTGTGTTAATAATTATTTTGTATTGCCAATGTTGATGAGGTTGTGTATCTATCGTCTTGGAATTGGTTATGATAATAAGTTCCAATTCGAATTTTTTGTTAAGTCTCTGAATCTGCATTTTACT

>CsSDG6

GGTGGTCATAAATCTCAACCTGTAGAGAGAGAGAGAAGTGCTGGAGGCAGGAGAAAAATCATCTTCAGCGGCGGCAACCATTGCCTACCTAGTCCGCGCGCATCAGGTTATCATTATGAAGCGTCATCGTTATTTGTTTTTTCGGGTTTTGTTATCAGATCAGATTCGATATATCACTGCTGTTGTTCACTATGTCAATTACAACATGAAATATCTTCGAACGAAAAAGGATCGATTCGAAATTTGGATCGTCTTTTCTTTTCCTTTTTTTTTCCTGGGCTAATCGGTGAAACGAGTAACTGATAGAAAATAATCTTCTGATTGTCAGTTAATGGCAGCGCCCAAAAATCGCGTTGGTTGCATTGTCTTTGGAATTCTATATCAACTGCAAAATCGTTTTCATTGCTCTGTATTTTTCTTGTGGGGAATTTTTTACAAACTTTACATCTTTGCCATTTAAATCTAAACAAGCTGGTGCTCGATCCTCATTCAATTTAATCATCTATGTTTTGTTAAATTACATCTTCCCCTTGCTATTTTCTTATTTTCTGTGTACTGCTGGCATGTAATTACATGTTTTTCACTTTTTTTTTTCAGAGGTTGGTTTATGCATCTAAACGTCTCTGCTTGTTTATGGTTAATCTTTATTGTTTGTGTTTTGAAGATGCAGGTTGCTGCGCGGAAGATTCTTTTTAGACCATGGTTTTTTCTACAGCTATTGATCATGAAGACCATGAAGACCATCACTATTTCCTTTCTCGTAAGAGGCTGAAAATTTCGAATTTTGATTGTCAGCATTTTGTTTCTAATCTCTCCGAACACATTTATGCCAACGCTACCTCATCAATGCAACCAAGTGCAGAAGATTGCTCCACTAATGGGTATTACATTTTTGTTCCCTCGTCTTAATTAATTTTCCTCTTGGGTTTTTTGCTATTGCTGTTACAGACCGTCTAAAATTATCATCAATGATTTGTCAGGTGCGTTGTTATGGGGGACATTTCTTCATCTTGTTGCAATTTTGATGAAAAGTTTCATTCAGGGTTTTTCATGAAGATGAGGTGCCAATCAAATGGCACCGGTGGTGATGTTCAACAGTCTTCCAGTAGTGGAGGAACTTCATACCTGGACAAGAGGTACTATGGTTACACCCCGGCTGGTAGTGTGAGTGGATGGATGTATATCAATGAAAGTGGCCAGATGTGTGGACCTTATATTCAGCATCAGCTATATGAGGGTTTATCGACTGGTTTCCTCCCTGATGAGCTTCCTGTGTATCCTGTTGTCAATGGAACATTAATCAACCCCGTACCATTGAAGTACTTTAAGCAGTTTCCTGACCATGTTGCCTCAGGCTTTGCATATCTGAATACTGGCAACATGAGACAAGAGGGCCTTTTTCATCATAGTGCTCCAGAAACTGTTTGTTCTGATTCGCAATTAGTATCACAATCACTTGTCAACTGTAGTTATATCTACAATCCGATGGTGTCAAACCCTGAAGCAGCTAATTGTGTTCCATCATTTTTACCAGGGGTACTGTTTATTTTCTGTTTAAAATGTATTATTTTGTTCTCCTACAAAGTCATTTTGATTGTATTTCACTACATGAAATACGTGGTATGATATGCCTATTGAAGTGTTTTTTTTTTCATTTGTATATTTTTTCCAGTCAAGTGAAGATGCTTGTTGGTTGTTTGAGGATGACGAAGGAAGGAAACATGGACCACATTCACTTTTAGAACTATATTCTTGTCATCAATATGGATACCTCAAGGATTCGGTCGTGGTAAGTTATAAAGTTATGGCAGCATTTATTATGAAGGAGGCACTGGGCATTAAGTGTATGATCTCTTAATCTCCAATTTTTGGCGAAGTGTTTTAGTCTAATGAAATTTTTTAGAACTGTTTACTGTAAAGGATCATGTTTTGGCACACATTTGTCAGAGGGGAAGAGCACACTGAAGGATATCTGTATATTGTTTGCACACTGTAGGATAGTTGCTTAATATAGTGTTTACCTGTCAGACGGTGTCCTGGCTAGTGACTGGTCCTGAAGTTGTTGTATCTGATGTTATGATCAGTCATATTGTGATTATTAATCTTCAATTGCTTGGGGATATCACTTGGACACATTTGGGTATTCCACAAAGTTCTGTTGTTTCTCACTCTAGCTTTATGTTTATTTTATTATGCAGATACATCATGATGAAAATAAGGTTGGTCCCATCAAATTGCTGTCTGCTATAAATGCATGGAGAATAAATGGACTGGAAACTGTCCATGCATCTGATGCTAAAATTTATAAGGCCGGCTCATCCATGAACTTCATATCTGAAATTTCTGAAGGTGTTTCTTCTCAACTTCATGCTGGAATAATGAAAACAGCACGTAGAGTTCTGTTAGATGAGATCATCAGCAACATCATATCAGAGTATGTTACTTCAAAGAAAGCTCAGAAACACCTTAAGCTTCACCAAGTTAATCAGGCTGCTAACAGTGGCTATTCTGATGGCAGAATGGTAATTTACACAGCATTTTATTTTGCAAGAATGAGCTGTATATACAATTGCTGAACATTTACCATGCTGTGGCCTTATTTTTATCTAATATGTTGCAATACCTTGCTTTGGCAGTCTGAAATTGCCCGTGAGACAGATAATGGATGTGAGAGGAGCAATCATGCTACAACTGGCTTTGAGGCAGCAGCCAGTCACAATATTTCTAATCAGATGTGTAAACATGAAATTCATACACTATCTTCTGCTTGCACAAAAACTGGTGGAAGCATTGAGATCTTCTGGGGGTCTTATAACATTGTATGTAAAATGCTTTTTGATCACTGTATGCAAGTCATGTGGAATGCTGTCTTTGGTGACCGTGTAGCAGAATATTCTTCTGCCTGGAGAAAGAGAAAACTTTGGTCTGGACATCCAAAGATTACTGGACCTGCCAGTGACTATAAGGATGATCGAAAGAGGATGGAGCAAGCACCTTCTCGACATGTAAGTGGTGATCCTATTTAGGGATTGTGTAGTCATTTTTTTTTCATATTTGTTACACCTGAAGTCATTTGTAATGGTATGGACAGTTGCTATCTGAGCAGGACTCCTCTGTATCTGATGATGATTGCCCTCCCGGTTTTGGGATGGTGGAAATCAGAACAGAAAATGATGTACAGCCGTATCATTTGTCTTTATCTGTTCCTGTGGGAGAAAATTTATCTAAACAGAAGAATCTATCATGCAATGATCATCTATTACTCGATGATGTAAAATGCATCCTCGATGGTGTGGAAAATGAGCTCTATTTGTCCACAAAGGCAACTTATACTGAGTATGTTGAAATTCTCGTGGAAGATGAAGTGAGGAAAGTAGTAAGTGCTTCAAAGGGTATCAACATGAAAGAGGTAATATGAAAAGTTTAGTTAGACTTCTGCTGACTTCCTGCATATTGCGGTTTTTTTTTTTTTCCCTTAGCTCTTCAACATTTCAAATTGGTTATGGTAATCATTTCTTGAGGAAACAACTTTGTTGATGCAGGATGTTGTTGACCCTTCAAGTCATGATCTGCATACCTGCCAATGCGGTTTTGCAGATGTAAATGGTGGGATGAGGATTGATTCAAATGAAACATCTGCTGAGATATTTTCATCTGAAGATTCTAAGAGTCTATTCCAAGCTGGAAAACCCCTGTCAAAGGATCTTTTGTCTAATATTTTGGCATGTGCTTTTAAGAGATCATTTAGTGGTTTTGTAGACAATGTGGTTGATGAGCTAGAAACTGATGAGCCGTCTCCACCTGGATTTGAAGACAGTGTCAGAAAACTTGTTCCATCATGTAATGGCAAATTCCAATTTTCATGGTCGGATGAGTTTACCACTAAGATGGGAGAATATGTTGCCATAGCAATGTGCCGTCAGAAGTTGCATGCAATTGTAGTTGGTGAGTGGAAGTCATTGTTTGTTGATGATGCTCTTCAGCAGTTTCTTGCGTTGTGGTGTAATATGAAGGAATGCTGTGAAGCTGATGGCAATGAGAAAGCGGTCTGTTTTGTTTATTATTTTATTTTCTTTTTTAAATTTCACTCTTTAGAGTGAACTGTATATGTGTTGATTTTTGAAGCACTTGATTTCTTATATGTTCCTCGGGTGCTTATTTGGTGTTTCAGGAAGGAGCCTCTAATGCTCACAATGAACATCATGGTGATACCTCTACTGTCGTAGACAAACTTAAAGAAGGATCAAAGAGATTTCACAGTTCAGAAGCATCTACAATGGTTGAAAAATATACGTATCACCGCAAGAAAAAGTTGTTGCGGAAAAAGTTTGGATCACCCTCTAATTGTTCCAATTCTGTTGAGAATGCATTTCAGACTGAACATGTAGAGAAGTCGAGAAAACAGGGAGTTGCTGGAGATGTTTTTGAAAATGCCAAAGTTCAACCCAGTGCTGTATCTTCTAAAAAGATTGGAAAGAACAAGCTAATTGATGCATCTTCTAAGAAGATTGGAGCAAATAAATTCACTGCTGTACCTTCTAAAATGATTGGAAAAAACAAAGTAACCGCTGAATCATCTGCCAGTGCTGGGTCGTCTAAAGTCAAGAGTAAGTTACCCAGTGGCTACTCATCAGCCAAAAGTACAATCAGTCAGAAAGTAATGAAAGTTACCTCTGCAGTTCAAAGTATGACAATTCCTCACTAATGCAATTTGTAGTTGCTATAATTTAGATTATCATACACGTTTAAATTATAATGGTTTTTGTCCATTCTAGGAGATAAGGTGCCTGTGCCAAAACCTTCTGGTGAGATGTTGTCAACCTTGAGCGCTGATGGAAATGATGTAGGGAAGGTTGTTCGTGGCAAAGCCCATAATGTTGGAATTGAGAAAGATTCTATTCTTGATTCCTCTAAAAGCAAACCAAATGGTAATATTGCCTTTGTTGTTCTCTCTCTCCCTCTCTCTCTCTCTCTATATCCATCTATCTATCTATCTATCTATGTGTGTGTGTGTAAAATGAGGATCAAGCTATGAGACAGAAACAGAAATTCCAATATGCTAAGGTACACAAAACACTGTATTGGAATCCTTTTTTTTTTTTAGCTTGGTCAAAATGTGGGATTCTCTTCTGGAAGATTATTCTACACACACACACACACACATGGGTGGGGATCCTTCACTCAAAATTTTGACTTAAAAAGGTGACATATTTTTCGTCCATTGGATTCATTCAATCCAATGTAAGGATTTGAGTAAAAAAGTAGCATTTTTGTCTGTGTCCACCTACTGTTGGATTCAAATCCAATGGCTCATAAAATCTGCATACGGTGGGCCCACCATGTTTAAAAATTAAAAAACAATGAGCCATTGGATTTAATGGCTCCAATTACGATCCTGCACTTTAGAATATATATACATACATATACATATACATATACATGCATACACGCACATATATAATTTGATTTCCTTACTGATACAATGGATTTTTTCTTCACGTGCAGCTACCAAGGAATCAAAACAGAAAAGGAAGCGTACAATGGATGGTTTGGAGTTACATGCTACCAAGGCTCTGAAAGTAGCGAAGGGTACCGCTAAGCAAGCAGCTTCTAGACAGGTTGCCATGAAAAAGACTAAGGCTAGTAAATCCAGAACATCGAATCTCTGCCCAAGATCTGATGGATGTGCTCGGTCTTCAATCAGTGGATGGGAATGGCATAAATGGTCTCTGAATGCTAGTCCTGCTGAAAGAGCTCGTGTCAGGGGAGCTCAGTATGTTCACACCAAGTATTTAGGTCCTGAGGTCAATGCATCTCAGTGGGCAAATGGTAAGGGTCTTTCTGCAAGAACAAATAGGGTGAAGCTGCGCAATCTTCTTGCTGCTGCAGAGGGTGCGGAGCTTTTGAAAGCTTCTCAAGTGAAGGTGATTGACCAACAAAGTTGTTTTTGGAATGCCTTTATCTTCTCTTTAGTTGTTGTAAAGTACTGTGTAGGTTGTCATCCTAAACTATTATATCAATTGTCAGGCAAGGAAAAAGCGTTTACGTTTCCAACGGAGCAAGATACATGACTGGGGTCTTGTTGCACTAGAGCCAATAGAGGCAGAGGACTTTGTCATCGAATATGTAGGAGAGCTTATTCGTCTCAAGGTAGTTTCAATTAAGCTTTTTAAATAATGGCTTCTGTTTTTAGCTGGAATCAACTGTCAGACCCTTTTTTTTTTGGTCACTTTTTCAATTGAATGTACTAAGCTGTCCCTTTTCTTGTGATAGATCACCATTAATTATTTTTTTTAAAAAAAAATTCAACAGATATCTGATATACGCGAATGTCGTTACGAGAAGATGGGAATTGGCAGCAGCTATCTCTTTAGACTTGATGATGGTTACGTGGTGGGTAAAACTACAATAATATGCTACCGTGATTCGACATGTATCTTTTCTTATTTTGTTCGCAGTCCTATAGAGGGATTAAAGAGAATGCTGTCTGATACTCTCAATTGAGCTACAGACAGAGTCAACTTATGTTGGGATGGGAAAACTATCATTTTGTTCATGACATGCTGCATCTTCCACTTTTTAACTTTTTCTGTTTTATTTATTTATTTATTTTTAATAGGAAACAGAGAAACTTTTACAGGATTGAAGGGAATAAGGATTACAGAAGTGAACCAGGGGGGTGGATTGTTTGTTCTGTATTATGGTTACTACTATTTCATTATAGTTTGTCTGTGTATGTGCAGGTTGACGCAACAAAGAGGGGTGGGATTGCCAGATTTATAAATCATTCGTGCAATGTAGGTGCATGAAATGACATGTGAAGTTGGTCTGCTGATTGGAAGTTAGTTAATTTCTGGTATTCTTCATCTGAAATAGTAATTGCTTTTGCAGCCTAACTGCTATACCAAGGTGATAAGTGTTGAAGGTCAAAAAAAAATTTTTATTTATGCAAAGCGGCACATAGCAGCTGGTGAAGAAATTACTTACAACTACAAATTTCCTTTGGAGGAGAAAAAGATTCCTTGCTACTGTGGTTCAAAGAAGTAAGATGGAATTAGGCTTATTGCCGTTCATGCATCCAGCTTTATGATATTACCAACATTCTTGATTGTTCTGATTGCTTTAGGTAATTTTGTAGATTTTTTTAGTGGTATAACATCAAACAAAATTGTATTGGCTGGACTGAGACTCCTTTGGAAGTTTGATGCATTATCTGAAGGCTTTTGTCTATCAATGACACTTGGATTGTGTGTGTCCGCATGGAATTGACTAATATTTCTTCTTTATCTTCTGTTAGGTGTCATGGATCATTGAATTAGGAATTACTTTTGTGGTGAAAATCCAGGTATGTCTTTATATAATTGTTTCTTAGACAGGAGTTTTATTTTTTGATCCAATATCCACTGCTTGTGTCTTATAAATGTTTATGGGCGTTGGTTATTCACCTGAGGTTGGTGTATGACATTGTAGCGCTGAGGATAATTGATGTGATTGTTATGCAATTGCCTTTTTGGATCTTTGATGCCATAAAATTTTGAATTCCCTAGTTCTGTTTTACTTCATTTCTCCTTCACTAAGTATTCATATTTACTTTGGTTGTCTATGGTTTAGAAGCTTAATCTTTAGATGCCTTTTGGAGGAAATTTACTTATGGCTCAAAATTGGGGAAACTAGTTCATCTATAGATGAATCTGATTTCTATGGTGTGCAACTTGGTGATCTAACAAGTACTCCTAGTTGTTGAACAAAATGAATGATGTCATTAGGTGAAAGTATCTCTTCTCATTAGTGTATTCTCCAAGAGTTATTCTTCCGGATCATCAGAATATTGAAACTATGTTTTTCTCCTGAGCAATATGATAATTTAACTGCTTTTATGAACCTATTGAAATGCTTCCTGTTATCTGGTGTAATCAATCTGATTTATAGAATTGTTAATCGAAGATTTCAAGGGATTGGCAGAATATTCATGTACAAACTGATATTCAAACACTTGTAAAAACTGATGTGGCATGAAAGTATTGAATATTGGAATGTCAAAAATAAAATAGATTTGTTCAAAGAGATGATGTGGAGTGATAATGATGACATGTAAGTTTATACGAGAAGAGTTTGTATAGGAGTTTGTATCTTAATCCAATAATATGCCTCATATATTAGGAACTGAAAATATTATAATTGCAGAAGAGATTATGGATATTCAGCCGACATAATCTCTTTCCAGTGTAGATGGATGTGCTAAACTTTCATTGTGTGATTAATTTATTTTTAATCCAACTTACAGGTTAGTATACTTTGTAGCTTGACATATTTGGAGGAGCTGAGATTATCTGTCATCCAATAGCTAAAAGCATGATTTTGTTTTTCTCTCCAATGCTGTAAAAGAGCGAATCCAGCCAAGTTCTTGACAGTGTTTATCATCAATTGTATGTTCCTGACACCAAATTGTTTGCATATTCAAATAAATTCAAATTTACTTATTAAACAGTTAGTATCTTTAATTTTTTTAAACGTAAACTTGTCTATGTAGTAGATTACGTGCTAAGGGGATCCATCACTGACTGATATATTTATCATGTTTTAGCCTGCAGTTATGTTGTGAAAATGTGAATATTCCCGTATGAATAGTGAGTCAATAACTTATCAAATGGTCAGTTTAAATCCACTTTGCATGTCAATTTTTGAGTTGGAAATGGAAATTGAAAACTATATCATCAAAATGGTGTTCATAATTTAACTTGTATTTTAGAATAGTGATAGGTACTTTATTTACAAAAAAAAAATCATATAATCAGTTAGTATTGGTTGTAACAATATCAAATATACATAGTTTGATTTGATTTTGTGTACCCTTGGTGTTTGTGTATCCTTGGTTACATAATTACATATTTCCACGTAATTATTCTGTTTAGTGCAAATAAAAATTTCAAATGCCGCAGTTTGTCCTAATGTCCCTTGGTCTCTCTTGTGCACCTATGTATGAAAAATTGAGGAAGGCCAGAGAACTTTTAACCTTTAGTTAGGAAAGCTTAAGATATGATATTCTAAGTAGGGATGGGTGCTGTGTGTTTGTATCCTGTCAGCTTAAGGTATAACAATATTTGTTGCTTTAACAATATGATTTATTTATTAATCGTGCCAAAAACCCAAAGACTAACTCAACATAATTATTAAAATGGTTGTAATTTTGTCATCTGTTTAACATATCTTAATATATTTCACATGTTTTTAGTGTATCTTGTTAGTATTTCTAAAATAATACAATGCTTCCTACTCAAAATACTAATATTTATTTTGGTCCAAACATAAAGTGTGATGAACATATCAGAGTTTCATTGGACAAAAAAACGATTAAGGCAACATGTTAACTCTTAATTGTGCATTGTCAGGTTTGCTTTTTGTGAATTCTAAATCGGAATCCAACACGACTATTTGATGGGCGAACATACACGGCATGATGAATAGTCGTGCCGTATTAGGTCAACCTCAAATCTGCTAGTTTTGTATTGTGTTCGTGTTTGGTTTGACGGGTCATGTCGCAAATTGCCACCTATATGCCGTGGTAGGTTGGCAATGATGGTAATTGTTGTTTTTAGCTTGCCATGAAGGCGTTCTTCTGATTGGAATTTGATATGTGTCTAAAAATTGTCTTGAAAAAACAGCATGGCCTCATTAATGGCATATAGGATGTATCAGTGCCATTTAAATTTGCTTGCTTGTGCTATTTTGTCAGAGCTATGCTTTTAGTTTTGTTTGAATTTGACATTTGTCTCCTCTGAAAGAGTCAATGTCTTCTTTTGATGTTGGTAATTGTTTTTAGTGCTATGAAACTCTGTTGAAGACGTAATGAACCATTTCCGCTTACGTTGTGGGAATTGACACAAGTATTGTCATTTCAGGTTTACTATTACTAGTAGTAAAAGCTGGACGATCTGCTCTGTTGTTCATTTCATATCGAGTTGTATTTGACTACTCTTCGAAGATGAACTACTGACGTGTGTGCGGTCTTGTACACAGATCTCTGTTGCAGATGAGTTTCTCTTCTTAGAAACTGTAAATCAGTTTGTCTTTCCTTGCTAACAACATTTTTGGAACAGCAAATTTTTTGTACAAACATGCATATTTATTTGTTTATTCAATATATAAATAAATGGAATGAATATACAAGATAATTCTTGCAAATGGTGCTGCTACCTCTGCTGGTAATATTATTTGGTGGAGAAAATTGTCATGCAACTCAACATGCATGTCTTTAATTCTTTCAGAGTAAAATGGAGAATTGACAGTAAAACAGAGAATTGAGGACAACTGGAAATCGTTTCCCCTATTTTGAATACAGAATGCTTACGACCGGAGGGCTCAATGCTCATGCTTATTGCTTACCAACCATTGTTTTAAAAACGTTTATGGAGCTATGTTCTGAACATGTCCTGCTTGTCCCTTGGATATGCATAACAAGCAGTCATCTTGAAGGGGCGCTTAGTTTTGAGCCATGGCCTTAATTGAGAATAGTGCGGAACCTACTTGTACTTCTGCTTATCTAGCATTCTATTATTACCAAGGATCTCGTTTGTAATATTAAGCAATAAAAGTTTTCTGCTGAAAAGAAGAGGCAGAGTAGCAACAATGAAGTGGAAGATAATGACCAAGTTTGAGTGCTTTTATGGGATAAAGGATTGACCACTGACTTTAGATGTCATGTCAAGTGTTGGGAGGATTGATTAAGGAATTGAATTAGCTCCAAATTTCAGTAGCCATTAATTTGATTTATTATTGGTGCAGATGTCATCTCTGCATGGGAGAGTGGTGATTAAACAACGATGGGTAGTAAGGAGCTTGTCATGTGCATTTGATAACTGCCAAAGCATTAATTCTTTTTGTTTTTACTTGGCTTGTGTTTATTTCTGTGTCTTTATTTTGTGCCATTCCACTTTCCATTTGGCTGCAGGAGCGGGTCAGTTCACACACTGTTAGCTGCTGGATTGTGGCATTTTTATGCGTTGAGGTACGTATCTGCTTTATTTCCTTAGTTTAACAGCTCATTGTGGGCTGGTACTAGTGGGGGAGGTGCAATCCAAAACGTGAAGTGAAGCGACTCTACCTTAGTGCTGTTTTTCACCTTCATTTCACCTTTTTTATTAAATATTATTATCAATCCCAAGTTTCCCTTTATTCCCAAGTGAGAACTACTTATAGGAACTTGTAATGAACACTAGGGAGCTTGTAATGAAAAATTGAGAACTTCTAACTAAAAGCAGTAACGGCTCTGTAGTATAGCTGTTCCATTTTAGCCTTGCCCCCCAAGCGGGAACAACTCTACATAGTATAGTCACGCCTGCCAAGCAATATACAAGTAACAAATGCTCTAAAAGTAAAAAAATTACCCTCCTGGAGAGAAAAAGAAATTTTGCTGTAGATCCTTATATTTATGGGTCAGAGAGAGAGAGTTGGAAAGTATAAATGAGGGGAAAACCTTCGTGGATGATGTAAATCTTACTCAAAAAATCCAGGGCAACCATTTTATTTTGTCGGTTTGATGGATTGAGAGAGCACACCGTTGCAAGTTTTTCACTTAAGTAATTAACCAATGTTGTGCTAAATATTAAACATTAACCATAACGTCTTTTAACC

>CsSDG7

CTAACAAGCAATTCCTTTTCTCTTTCTCACTCTTTTTGTATTAAAAAAAAAAAAGCGAATTTTTTGAGAGTGATTAAAAAAAAAAAAAAAACCATCACTATTCGGAGAAATAAAAAAAATATATAAAAAACAACCGACTTCGTTCTTCAACGTCCCCGTTGCCTCTTCTCTGAAAAGGTATTTCATTTCGGTTTAATAATTTCCTTATGATTTTTAGAACTCTTATTTGTTTGTGTATGTGTTTTTTGTAATTTGTTTACGTTTTATTATTATTATTATTATTTTTTGCGTAGCTGCATTGGTTTGCAAATTTTTTTATCTGGGCAATTTTTTCTCTTTTGCAGATAAGGGGATATTAGGGTTTTTATCTCTGCGATATTGCTGCTTCTAAGAATCTAAAGCAGGTAAATTTTTATGGTTATGCTTTTTTTTTTTTTGAACGAAGACGCTTGACGGAGCAGACAACTTAGGGTTTTGATCGGTGGTGACTAGAGCTTGCATTGTATTGGGTGGTTCGATTTTGATCAAATGAATGATTGGTATTTCTTAGTGTTTTGGGAAAGTGCAAAAGGCTGGGATGGGTTCGTGTGAGAATTTGACATCAGTTGATGAACCCTCGTGTAACTCTGTTTTTGAACAGCATTCGAGCTTGAAATTTATGGAAGAACCAGTTTCTGAGCAGAGGATGTGTTTGAAAACAAGATTTGATGTGCTTGATGCTAATGTGAGTGGCAGTACGAAGTTGGATGGGCGTTTGGGTTTGTCTATTGATGATACCACTCATTGTGTGAGCTGTGGAGATGCTGGAGATACTGGCATTGGTAATAAAGATGGGCTGATTGATGAGTTTCAGAATGTGGTGGGTGTGAGTTTGGAGAAAGCGATAGATGATGAATGTGGGGTTAGCAGGGTTTGCTTCATTGAGAGTCAGGAGGAAATTGGTGTTTGTAGTTCCCCAGGTAGGTGTTTGGATTTGTCTCAAGATGGAAACAATAGTTATGTAAGTTCTGCTTATGTTACAGAGGCTGTTGTTGGTGATAGAGATGGGTCAGTAAGTAAGTGTGATAACGTGTCTGGGTCGGGTTTGGAGAAATTGGTTGATGAGAGTCAGAGTAAAGTAGATTCTGAGGCTGTTGTTGGTGATAGAGATGGGCCAGCTAGTGAGTGTAATAATGTGTCTGGTTTGGTTTTGGAGAAATTGGTTGATGAGGAGCAGGGGGTTTGTTTGGATGGGAGTCAGAGTGAAGTAGATGTTTGTGGCAATGGGTTGTGTGTGGAGAAAGGAGGGTTTCAAGGGGAGGATTTGGAATCATTTAAGCATCAGAAATTGCCCTTGGGAGAAGTGCCTAGCAACTGTTCACCGAGAAATTGTGATCGACGGGATAAGCAGAAGGATGATCAAGGGTTCAATCGTTCCTCTGTTGAAGAGACTGTAGAGGTTACGGGAGTGGAAACTAATGCTTTAGCTGAGGTAAAGTTGGGCAATGATAACCAAACATTATCATCTGATGCCTATGAAATGCCCTTGAAATCAATTCCTGTTGGTGGGTTGACAAGAAATTGTGTTCAACAGCATGGGCAGAAGGGTGATAATATTCTTAGTTGTCTCCCTGGGGAGGAAGGGGTTATGATAAAGAAAAGTGATGAGTTAGCTGAGTTGGAGAAAGTGCCTTGTGACTTGATTATGCCTTTTGGAAGCTTTGGGATTCCCTTGACTGGATCACCTAGAAATTTTGTTCAACACGGCGATCAGAAAGATGGTAGGACTGTCAGTTGTTCCTCTTCAGAAGGGGATATGGAAGGCAAGGAAGTGAAAACTGATGCCCTAGACGAGATAGAGAAAGTTAACTGTTTCCAGATTTCGCCATCACAATTTTGTGAAATGCCTCCAGAATTATTACCCTTCACTGGTTCACCTAATAATTCTGTTCAACAGGAAGATCAGAAGAATGATAACACTGCTGGTTGTACCTCTTCTGAAGGGGATATGGAATGCATAGAAGAAAAAACTGATGCTTTATCTGGGAAAATGAAAGTTACGTGTGACCAAATGTTGCCTTCTCAATGTTGTGAAATGGATTCAGAAGCTATATCTCTGGTTGATTCACCTACAGATTGTGTTCAACTGGGTAATCAAGAGAATAATAAGAATGGCGGTTCTCTCTCCTCCAAAAGTGCTAAGGAGGTTATAGAAGATAAAATGGGTACATGTGGCCAAATTTTGCCCTCACAGGGCTGTTCAATGACCCCAGAATTAATACCTAAGACTGATTCACTGAGAAATTGTACTCAACAGAATGAGCAGAAGAGCAACGAGTGCATCTGTGTTCCATCCTTGGAAGAAGAAGGGAAAAATTATGCTTCACTTGGAATAGAGATAGATATTTGTGGGCATATGCTGTCTTTCCAGGAATGTGAGATTCGCTCAGAGTCGACACTTGTGACTGAGAAACAATTAATTGTTGAAGCGAAGAGAGATATTGTACATGGGTTAGAGAATGATAGTGGCCACCCAAGATCACCTTTAGAGCATACTGAACCGCGCATGGAATTTGCATCTGCAACAGACTTGTCATTTCGTTGTATTCAAAAAAAGGAGCAGCAGGGCATTGAGAGCTTAGCAGAAGGGAAAGCCAATCTGTCAGCGGCTGTAGAAGCTAATATGTGGAAAGCCAATCTGGCAGCGGCTGTAGAAGCTAATATGTGCAACTGCATATCAGCTTCACAGGATGGTGAAACGCCTTTCAAAGTTTTTTATGAGGATGATTTAATGAGAACTTGTGAAGGACATAAGGATCATGTGGATCATGAAAGCATTGGTCATCTCGCTGTTGGAACTGTGGAGCAAAAAGATAACACTGACACATGCATCCTAGCATTGCCTATGCAGAGTTGCCAGAGCTCCTTGGAAAGTTTGCGTATAGCTGATTCACTGAGTAATTGTTCCCAGCAGAATGACCAAGGGAATAATAAGAGTGTTGATGGTCTTTCTGCAGAGAGTGCTACAGAGGCCGTGGAAGAGAAAAGTGATGTTACAACTGATATCAAGGTTGAGATTTGCAGTCAGCTATCACCAATAGAAGAAAATGAAAAGGAGCACTCCTCCAGAGTTATTGAGAAACCAATTTCTTTGCAGTCATGCCAACCCTTTGCTGTAGACGAGAATGGCTCTTGTAAGAGTTTAAATGTTGCGGGTCTCTCTCAAAAAGATGGTTTTGGTGCTATTAGTTCTAGTGGTGCGGTTGATGGTTTTGGACAGATCAATCATGAAGTAAAAGATGATGTGGGCACGAATTGCTTTTCTGAAACTAAATATCCTAACAGGGTATCATTATCTTCTAGAAGGAGCAGCCGTATAAGTAGATCTAGCCAGAAGACTCAGACCAAAAGGGCTGCGAGGAATTGCAGGACTAAGGCCAAAATTCAACATTCTCATGGGAGTATTGATATAATCCTCAACATTGCAAGAAGGAAGAGAAGCTGTTTGTCCAAACCAGCTCGTTCTTCTATCTGGGGGTTATTGGGCAGCATTACTCAAATTTTTGGTAAGAGTGGCATGAGTAGTTTCAATTTATCTCAGAACCAAGGATCACAAAAGGCAAGAGGTGACCATAGAAGTCAAAAGCGGAATAAGATTCAGGCTAGTGGAAGCTCATTGACCCCTAGTAAAAAATGGAATGTTTCAACTCGATGCCTTCGTTTGAAGGTTAAAGTGGGGAAGGAAATTTGTCAAAGTACTCTGAATGTTGTGGTCCCCAAGGTGGCTGACACAATGGGATCCAATGATATTGTTGTTGGTGATGACATTTCTGAGTCATACCCTACAAAAAATTCTGAGTTTCCAATATTAGCCCATGAAGATGAAGATATATTTGGGGAAGAGGGCACTCAAAGACAGTTTCAGTGTTTGGATAGTAACCCGGAGGAGGTAGTGAAACACCCAGGTAACTCTATCTTGGATGTGCACTTTGCAAGTCAGGAATTGAAGGCCACTGTGATTACAGACAATGCAGCCGGGGATGTTGCAGATGGTAATTCTGCCCATAAAGGGGTTGGAATATTGGGTGGAGCAAGTGAGAGCAATTATGTGGATCCTGGGACTTCACCAGATTCTGAAGTTATCAATACAGCTCCTGATTCTGAAGTTGGGACTAGAAGTAAAGAAGGCTCGCATAAAGTTGTTTTGACTTCCTCTGAAATCTTTGCTGCTCCTGGAAATGTCACCAGCAGCCGGAGAGGGAAGAAGAAAACTAACCTCCTTTTTGCAGGTAACTGTAGTCTGCATGACGATTCACCTGTTGCAGCCAGCAAAGTTAAACCACCAAAAAAACGTGGTGGCAGACAGAAACTGGAAGATGGTAGTCACTCTAGTGATTCTCTCGTTGCATTTCCTGTCACTTATGCTTCAAGTAATTCATCAAGCGGCAAGGAATTTTGCGGCGAACTGTTGCCTTCATCGAGAGACAGTGAACCTGGAATCATTGAAGAGGCTATGGTTCCTTCAGTTAAATGCAAGGGATCTGAGCTTAGCAAAAGTTTAAAATCGGGTGGAAGGAAAAAGGGAAGGTCCAAAGTCTCTAACTCAGCTAAGAGCAGGAGAAGAAAGGCTTCTACACAGAGGGGAAACCAACGGAAGTCAGTCAACAAGAATGAAGTCAAGGAGAAGGGTGTTTTAGCTGCCAAAAGAAGGGATGAAGGTGTTTTGGAGCTGGTTGAGGAAAAAACAGAAGTTCGACCACAAATAGGTATTGGAATGTTTAAGTTTTATGATTCTGTGAAGAAAAAATGTTTACCTAACAGGTGCTGAACTTTTTTATTTAGCAGGACCTCAACACGTTATCTGTTTGATTGTTTAATCTTACTGTTTTCTTCTTATAGTTCTCTTATCATGGATAGGAAAGATCTTTTTTGTTTCCCAATTTATTCAAACTAATGCTGTAGGAAGCCACATTGCAGATGATATTGGGAAAACAGATTCTGGCAACAATAGTATGTCTGTTGATGTATCAAATGCGGAGATTACATCAGGTGGTGAACCTGAGCATTATTGTCCTCCCGAAAGTGCTTGGGTGCGGTGTGATGACTGTTACAAATGGCGGCGTATCCCAGTTTCAGTTGCGGATTTGATTGATGAAAACTGCAGATGGTACATATTTTTCTCGTTCGTCATCTCATATTTTTCCAGTTATGTCATAATTGCAAATGTTTTTTTTTGTGTGTGTTTGTTTTATTCAACAATTTGGCAAGTGGGGTGGGATAAGTTATTTTAGACATCTATTATGTGAACTTTATTCAAATATTTCTTTAACTACAATTTTTTTTTTAATTTTAATTGAGCCTATGTTCACGAATTCAATTTTACAAATTCTTATAATTTTTTGTTGTTAGTACCACTGCACCAGCCTTAGTGTGAGTTGTCATGATTGTGTTTGCTACCATTTAATTTGGGTTTTTTCAGTCTTGATCATTTTTTCCCCTTCTCTAGTGTTGCATCTGAATTGAGATATTTTTGAGTTCTCAAATGTTATATTCTCAATTGCATGCCAAAATCTGGTTAAGTGAAGATCTAGCATCGGTTTAGTTGTCCGTATGATTGGTTTTTAGTGTAGTTATAGCTATATTTGGCATAAGTTTCAGGACATGGAAAACTATGTTTCTTTTCATTTTGTTTCTACCTTCTGTCTTATGAAGGGCTCACCGATAGCTATCCCTCAGTCTATATTTATATTTTTCTTGTATTATAATCTAATATCTCCTATTGGATTTGATATTAAATTTTTCGTTATTGAAGGGTCTGTAAGGACAACATGGATACAACATTTGCTGATTGTTCAATTCCTCAAGAAAAGACAAATGCTGATATTAACGCTGAGTTAGGCTTATCAGACTATGAAGAAGAAGACGGCCTTATAAATTACAATACATCAGGAAAGGGTTTGGATTTTCAGAGTACACCTGGTATGTGTAAGATATCTGGGCTTTAGGTTCTTAGGTTCCTTTTTCCTTTTACAGACATTTTAGTTTTCATATTTTGACCTGGCTTTGATGTGTTATGGCTCTTCCAGGGTCATCATTTAGGCGCATTGATTCAAATGTCTTTCTTCATCGTAGTCGTAAAACACAAACAATTGATGAGGTCTGGAACCTACTCGATCCATTGCCTATCATTTATATGGTTATTTCCATTTGGCTGTGTTACATTTATTTACTTTAGAAATATGAAGAGTTCAGTGTTCTTGCAACTGTTGTGTTTATGACTGTCTTGTGCTTATTTTACTTGGCAAAAATGCACCTAAAGTTCCATGCCTTGCTCTGTTTGTGTTCCATGTGCCAGTAGTCCTTGACATCAAACCGTCATATGCATCAAGGCTGTGTTCAAAGTTTAAAAATAAAAGCTCTTCCTTCCATTGCCAAATTGTTAGTCTCATTTAAAAAAAAAAAAAGAAATTTAAGGGTACATCATTTGCTACATTTACATCCATATTACTTTTCATGATCAATCTCTATTCCATTGCTAAATTGTTAATTTTAGGGTGCATCATTTGCTACATTCACAAACGTATTACTATCCATAATCGTTCTCTGTTGATTTTCTGTTAAAGATCAACCTTTCTCGGAAGCTTCTAATATTACCCTAGTATAGTGATTTATTTTCTATGGATGTGTAACAGAAATGTTTTAATTGATAGGAACTGCATAATTATGATACCCGTTTCTTATCAAAAAAACAAAAAAATTGCAATACCCCTTGATTTTCTTTTTCAATTTTGCTTTTTGGCTTGGAGAAATTTTCTTATGCTATGACAAGAAGCTAGCACCTAAATTTCTTAACCTTTTTCCTTTTAGTTTTCCAATTTGTCATAGCTTATATAAAGCTGATTAATTTCTTCCTGTTCAGGTTACTAATTTTTTTTTTCCTGATCAACATGTTTGCTAAATTTAATCAGGTAATGGTTTGCCATTGCAAACCACCTTTAGATGGTCGGCTCGGCTGTAGAGATGAATGCTTGAATCGGATGCTTAACATTGAATGTGTTCAAGGCACTTGTCCTTGTGGTGACCTATGTTCAAATCAACAGGTACTTCAGCTCTTCTGTGCATATAACTTTCATTTTCTTGATCAACATATGCTGTTTTGTATATAAAGGGCAGCGTTTGAGTCAAAATGTCTGGTTACAGTTCCAGAAACGCAAGTACGCCAAAATGCAGTGGCGTCCATGTGGGAAGAAGGGTTATGGACTAGAGTCACTTGAGGATATACTGACAGGGAAATTTATTATTGAATATATTGGAGAGGTAAATTCCTGGAGTTATCTCAAGTCAAGCATGAACTTCTTTATTGCGGCTAGACAAGGACAGATTCTTAGTGCTTATTGCCACTGTTAACCTGTATGCCAAATCTAAGTAAAATTGGTCCTGCAAATTAAGTGTAAGGGTGCAATTGTTTTCATTGAAAATTTGCTGATTTTCTTTTTCTTATTATTCGTTTTCATTAGTAGAACAATGAAACTGGTGGTTGTGTTTCTATCTCTTAGAACATGATTTCAGTGGCAAATAAATCATCTTTCGAAACCATTGGCAAAATAGGAATCTTTGTTATTTAGCTTTTGCCCATTATTATCATTTGTCGATCAAAGTTGGTCTTTTTAGTTTCAGTAAAGTGTTTAAATTTCTATCCTTTGTTCACGTATAACAATAATTTTTTAATCAGTGTCGGGGGTCTATTTTATTGCCTGGATTTTCTGTGAAAGAGGTTACTAATGTCCGGTATTTTGAAGTGGCTTGCACTTATACTAGTATAATTACGTTCACATATGAGTTCATCCCCCCTCTTGCATGTTTCAAGCAGGATCCTGTTAGACCTGGTGTAGGAACTATTGCCCCACTTTTGGGCTGGATGTCATCATTACTCTGGATTCCAGTTGCTGATCCATAGTTTCACATCCTTGACGTTCATGTATAGTTTGTTTATGGATAGAATAGATTCAAAATTACTTTTTTTCTTTTTGGTGGCCAAAATAATATGCATCCAGTAATTAGTAATGTTAGATCTTTTTTTTTTAAAAAAAAAAAAAGTAATTAGTAATTATAGACAATTCTATTGTAAGTCTTGCTCTTTGTGTTTTACAATTTGACTCTCTCCGGATCTATCAATCTTTATTTTATTGTCAAAATTTCTAATATATGGCAATCAGGTCCTTGACATGCAAGCTTATGAGGCACGGCAAAAGGAGTATGCGGCCAATGGTCATAAGCATTTTTACTTCATGACATTAAATGGCAGCGAGGTTTGTAAAACTTTCATAACATACTTTAATTTTAATTCTAATGATATGAGGTGATAATGTATTTCTGTTAAACAATATTTCTCATCATGCAGTGATTGTAAATGTTATGAACCATTTGTAGACTTGTAGTGGTTTGCTAGCTTCTTTTTTTTTCCCCCTTCCCTCCTTAACCTCAGTGGATCTTTGAGTATCTTTGATCTTTTTGAAAGGTTCAGGAGTTTTTCTAGGTGCTTTCAATATTTAACTTGTTATTGAACATGAACAATATTTTTTGGGCTTCTCGCTTCTGAATATGAAGGTTTTCTCGGGTATTTTTCTATTTCCAACAAAAGTAGTTCTTGTTGTAGCAAGTTACCTGAGCATAGGCTAAATATGCCTACTGCTGAAATCTTTTATGTGTATACTATGAACCATTTTAGACTTGTAGTGGTTTGCTAGCTCCTTTTTTCTTTTTTTGCTTTTTTTTGCTGCTGTACCGTACATCCGCCTTGGCCGTACAGCAGTTGTTAATTTTAGGGTGCATCANNNNNNNNNNNNNNNNNNNNNNNNNNNNNNNNNNNNNNNNNNNNNNNNNNNNNNNNNNNNNNNNNNNNNNNNNNNNNNNNNNNNNNNNNNNNNNNNNNNNNNNNNNNNNNNNNNNNNNNNNNNNNNNNNNNNNNNNNNNNNNNNNNNNNCCTGAGTGCTCTCTCAAAGGAAACAATGTTCTAGTGACCTTATTACTCAAATGTATTGAGCTTGTGATGAGTAGCAAGAGGAAAGATATCCCACTAATGATGCTTCATAATCTTGTATATGATCAATAACAAGTTAAATACTGAAAGCACCAAGAAAAACTCCTGAACCTTTCAAAAAATAAAAAATACTCAAAACATTTGACCTTGGTCATTTAAATTGAAGAATGTTTATTGCAACTTTTTTTACCATGGAGTGAAGCTGGACCTTGTATTGAAAACAACTGGACTTGTTTACCCTGATGTTTGTAAACTTAGGCCGAGATCTAGCTTGCTCTTGGGCCTTATGATCTTCCTTCTGAGCATGGTCTGAAGAGAGTTTGATATGCTTGGTTTGAATATAGAAATTGTGGTGTTCAATGGAAATAAATATGCAAGAAGCTTTAGGAATACACTGTGCAATGCGGATTTACGCGTTATGCTGGTTTATACCCTACTTTCCTTCGTAGCATCTGATTTTCTTGACATAATTGGTTGCATTTTAGTTTCTTATTCATTTTTTCTATTCTCTCAGGTAATAGATGCATGTGCCAAGGGAAATCTGGGCCGTTTCATTAACCATAGTTGTGATCCTAACTGCCGTACTGAAAAGGTGAACATCCATTTGAGCATCTTTTTCATGCAGTATGTATGTTGAAATTATATTTTTAGTTTACTATTTGTTGTTATCTCGTTGAAAATAGTCCACATCCACAGTGGCATTATGGGGAAATTTTTCCTATACTTATTGGGATTTTTGGTTCTTGTGAGATACTGATTATGGTTCTGTGATTTCACTGGATTTGATTTGCTTTGTTCTATAAGAATAGTTCATTTTGTGGAATAAAGCCAACTTTGTTCTTCTTTAGGCATTGGTTTTAAAAGAATCTAAAGACATTTCTCTCCCCATCACCTTAAAGAAATTGTCATGCATTCTGTATAAAATTTTTTTGGAATTTTTTAAAATTTTTTGTGCATCCATTTCGACTGTTCATTTTTGGCATTCTCACTGTGCCTCTTTTTGCTGTCTTTGTCTCATGTGAACTTCTGGTTGATATTTTGTTCTTCAGTTGTATTTCATTTTGTTTGGTAATATCATGCTGTCTTTCTTATGGAAAGAAAAGTCAACTCATAAGTACCAAACGTTGGATAAAATATTCTGATATCATGAAATATCAATTGTAACAAACATGAATACATCAATTATAACCAATTATTGAGAGTGAGCCAAATGTCATTAGACAGATTTTTTTTTTTTTTCGAAATTTTTGGGTTTCTTATCCTTATCGTGTTAATATGGCTTCCCATTTTATTGAAATCTCCATTCTGACATCAATGCATTTGATGTACTCATGTTGGTGAAGCTTTAATCTAACAGTGTTCACTGTTTTGTTTGCTATCGTGGTACAGTGGCTGGTGAATGGAGAAATTTGTATCGGACTATTTGCAATGAGGGATATTAAGGAGGTATGATTTCACCTCTCCTCTTCTCCTGAAGGGAAAAAACTTTCTCTATTGGCTGCTTCTTTTACAAGAAAAATGTGTTTTGAGAGAGAGACTCACGAATTGTCATGAATTGGCAATGAGAGATGAGTGAAAAAAATTGAGAGGCAGTAACTAGATGAAAGAACCATGTGTTTGTACAAGCTTCAGCAAGAAATTGTAAAATGTCTTTCTTGTATATTCCTGACGACAATTTAAGTCAGAGCAACATAAGATGGGCTCATACACAGACCCTGGGTTGTGAGATGGCTCATTGTTTGCCATAGCTTTTCCCATTTCTGGTTTGCTACTCAATAAAGATATGTCCACCGAACTGATTGGAGTGGGTGCCTGATGGGCCAGACAGAAATTGCTATACTAGTTCTTATCCCATGCTTTTCCTTCAAAACATCGCCTTACCGTAACCTATCAGATATATGTTATTTTATTTCATGCTTCATTATTTATAGAGTGCATCCTCTAAGCCTATTATCTTTCAGGGTGAAGAGCTGACATTTGACTACAACTATGTCAGAGTGTTTGGGGCTGCTGCCAAAAAATGTCATTGTGGTTCTCCTCAATGCCGGGGTTATATAGGTGGGGACCCGCTAAATACTGAAATAATTTATCAAGGTGATTCAGATGAGGAGTATCCTGAACCTCTGATGCTTGAAGATGGTGAAACTGGAGATGGTTTCAAGACAATGTCCAGAACCAGTCCCTTTTATGGTGACAGAACACAAATTTCTGAAGCCATAGCAGAAGACACAAATAAAATGGATGATTCTGCCACAGCTGTTGGACAATTGGAGATTTCTGGAAATGTTAATGACTCTAAGAGTCAATCCATTCCTGTTATTCCCCAATTACTCCATTCATTAGAAAGGGAGGATTCAAAAGGGAAATGTCCTCTACTTCAGTCACTAGAAACTTCTCTTGTGGTAGAAAATGAATCAAGCATACCTGTATCTTCCGTTCAGCAGAAGGAAACAATGAATAAAACTTCATCTGTAATCCCACAGGTGGAGACTTCTTTGCCGGCTCTGATATCTGGGAATTTGTTTACTGATGGAAGTGACGCTGGTAGGAAGTCCAAGTCTGATATTGTTGAAGACAATCAAAGTTTGCCGAAATCTCATCCTCGTATAAAGACTTCTCGTAAATCTGGATCTATTAAGAAAGGCAAGGTTGATGGTAGTCCTTTAAGTGGAAATAAAGTTAAGTCGGTTGCCAGCAAATCCCAGGTTTTCTTTATCAAACCCAAAAAGATAATGGAAGGTTCCTCTAATGGTCGTTTTGAAGCAGGTTAGTGATCCTCTTTTAATGTGTATTTGGGAAATGTGTTCTTGAGCAGGCTGATCATTCTAATGATAAATTGTGCTCAATGTTGCAGTTCAGGAGAAATTAAATGAGTTGCTGGATGCTGAGGGGGGCATTAGTAAACGTAAAGTGAGTAAATTCAGCATTATCCATTTTTACTATTTAAATCTAATGTTTTTTTGTTAGTTCGAATGGGGATGTCATTCTCAGTGTTGTCCGCCTTTTGGTCATTAAATGTGCTTCATTCTGATTCCTTTTGCTTCACCAAAGCACTGGTGCCATTCATTTTAAAAATTTATGTTGAAGTCTCACCTCACCTTTCACTTGTTAGTCAGCCTGCTTCTTGTGAAAAGATATTGATGGCACTGTCACGAAATCTCTAGTTTCATTTGCTTAAAGTATGCACTGTCACAATTCTTTTAAAATCTGTGATTTTAATTTTTTATTTTCCTTAAAAAATGTCCTATCATTTGTATATGCATGGTATTCTATCTTTTAAGGCACTATGTTTTCCAAACAGGACGCTCCTAAAGGCTATTTGAAGCTTCTGCTTTTGACTGCTGCTTCTGGTGGTAGTGGCAATGGCGAATCAATTCAGAGGTATTTGAAGTTTTCCTTCCTCCGTATAGTCTTAAATATAATTTTAGATATCCAATTTTGCTATCTTAGTCTCTTTCTTATGTGAGGTCCCTGTTCAGCATTTTTTAATTTTTAGCTAATCTTTTTTGCAGCAATCGAGATTTATCAATGATCCTTGATGCTCTTCTGAAAACAAAATCACGAGTGGTGTTGATGGATATAATCAACAAAAATGGTAGTTTTTCTTTTCATTGCATGAGACGCAATTTTATTTATGCTTGTTTTTGTGGGAATAAACTAAATTATTTTAACTGCACTTCTGACTGCTAGAAAGACTGATAATTGCTCTGCTTTCCAGGTTTACAGATGCTACACAACATGATCAAGCAGTACAGAAGGGACTTCAAGAAGATTCCAATTCTTCGGAAGCTTCTGAAGGTGCTCTATCTGCGAACCCCCTTTTAACTTAATTGAACACAATTTCCTTCTTTTAAAAAAATAATAATAATAATTAATCACTACTGCAAAGTTCCGCAGGTTAATGAGCATTGTTCTTTGAATTGTTTTGTGTAGTATATCTCAATGTGTTATATATAGCATATAATTTTTGACAGAGATACAAGACTCTTTGTGCATTTTGATTCTTGGCAGAAAAATAGGTTTTACTGAGTGATTTTAGATTAAGTTGGTAGCTGGACTTTTAATTGGCCTAATTTATGTACACTGTCTTTTGGAACTTGATGATTAGCAAGTAAACTAAGTCCCGTTTAGGAATATGATGGGATAACAGCATGTGTCTACTATTTCCTGCAGGTCTTGGAATATTTGGCAGTGAGAGAGATACTTACACGGAATCATATTACTGCTGGTCCTCCTTGTCCTGGGATGGAGAGGTAAAAAATACTCTGTGTTTTCCTTATTCAGTGAATTTTTCCCTTTATATTGTGTTGTTGGTCTTGATTCAACTTAAGCTACTCTATTACTGAAACTAAGGTGGGTCAGAACCTATTGGAATTTAAATCTTTAAAGGGTTTCTGGTCATTGGATTGTATTGTTCTATGATTTAATAAGAAACACAGAATAATAATAGATAATCAGTAATTTGAAACAAGATTTAGAGAATATAGACACATGATGTGGAACCATTTAAGCACCAGTCCAGGATCAGCAAGACAGCAGTTGACTCAAATCTGCTGCTAGGTTGTGTGGAGTCTAATTTTAGTGTCGTACCTTTAGTGGGTTTATTCCCAAAAATTGATCATACTTGAATAGAAATACTATTCAAGTACATATAGAAGCATCTTATTCTTCTGCTAAGGTTTATTTAGTCTTTTCATTTGCATGCATCTTTATGAGGAGGCAGGACTCAATATGTAACCTCACGTTTAAGTCATAGGACTTATAGAAAGAGAAAAAGAACTTCTCGAGTCTTCTTTAAAAATGACTGATTATACTACAAGAAGTTGAGGAATTAACTTGGAAGAATAATGCCAAAATCACATTGATGGATAAGCAGGAAGGTACACCCACCAACTAGTTCTATTGCAGATTTTTCATATAACAGATTAATGTGTTTCTTGGTGCAGCTTTCGGGGATCAATATTGTCATTGACAGAGCATGATGACAAACAGGTTTGTTTCAAGTTACTTTAAGTAGATCATTAATCAAGAACTTTTGTTTTAGGAGATGGAGATCTTTAGATTACAAGTCATTTGCTGCAGTGCTTAGCAATTTATAGATGTATCAATTGACCAAATTAAGTTTCCTGAGTGACAATGCGGTGCTAATCACTGTCCGTTGTTACATATAGGTTCATCAAATTGCACGTAGCTTCCGAGATAGATGGATTCCTAAACCTTTCAGAAAACATAGCTACAAGGACAGGGATGATAGTGGAATGGATATTCACAGGGTTGCAAACTGCAACAGGCTCCCAATGTTGCACAATCATCGGCGTGATGAAAGTTTAAGACCTTCAGAAGCAATTGATTGTGTCATGCAGTCATTGGTTGCAAAAACTTCTGTGGATTCTGCTGCCAATGAGGCTGGTTCTTCCCCTGGTGCAGGAGGGTGCCAGACCAATGGCCCCAAAGTTCGTAAACGTAAAAGTCGATGGGATCAGCCTGCTGAGACAAACCTAGATTCAATCAAGCACAAGAAATTGATGCTCGAGTCGAGGGTGTTACCGAGTAGAGAGGACATCAATTGTCCTGATCATATTCACAATCACTGTAACAAAGATGAAGCTGTTAGCTCCGAAGATGGAGGACAGATCACACAAGAGGATGTTCCACCTGGTTTTTCATCTCCGTTTAACCCTCCTCTGGTTTCATCCGATTCTTCATCAACTACTGACCTGTCTCAACAAAATGTTTCACAGTTGAGATGTGCATTTGATGTGGCTATTGCACATCCTCAGGGAAAATTCAATTCCCGCCTACCTGTGTCTTATGGAATTCCATTGCATATTTTGCAGCAGTTTGGGTCATCCCAGGCTGAAACTGTTGATAGTTGGGTTATCGCTCCCAGCATGCCTTTTCATCCTTTTCCACCTTTACCCCCCTTTCCCCGTGATAAGAAAGACACTCCACCTGCCTCTGCTGTTAGTTGTAAAACCATTGATGGACCTGCAGAAGAATGGCAACAAGATAGCAATCATGGACCCAGTTGCTGTCCAGATGAAGACAATCCAAGCATGACTGGAGCTAACCAGTCAGATGCAGACATTCCGGGCACAGACGGACAACACACGTTTAAAAGAATGAGGGGATCCTCTAATGATCTGGGAAAGAGGTACTTCAGGCAGCAAAAGAGGAAGGGACCCCCTTGGCTTTGGAGGAGAAATGAGCTTAGAAGTTCTTATTGTTCACAGGATGTTAGTTGTAGGGTGGATAAACCTGTCAGTAGTTTTATTCAGCGTCCACCACAACAAAATCATCATTGAATTGATTTATAGAAAATATTCTGAACAGATAATTGAATTTTCTTCCATACATTAATGTATTCTTGATTCTTTTCTTGTGGACTTTATTTATCTGTGAAAACTTTATTCAATTTCAGAATTACAGTCCCCGCCCAAGTTATTCTGTCACAGCTGAGAA

>CsSDG8

CAACTCCTCTCTACCACTCCTTACAACTTTTATCGCTCTCTCACTTTTTTTGGCTGCAAAATTTAGGGTTTATTCGTTGCTTAACTTCAACAGTGTGTCAATTTGAAACTCTGGCCGGGCATTTTGGCTTGGCCTCTGATTTTAGTGATAATATTTTTGTTTTTTTTGAGATTTATTTTATTTTGTGTTTTTTATGGAGAAAGGTTAGGATTAGGGTTTTGAGAGTAGCGAGGGATGGAAAATTCATGGCAGATAAAATGTGGTTCATCGACGCAACCAATGGCCTCATCAACATCACTGGAAACCCGAAATCAGGTTTGACGATAATGCGCTGTCGTTTCATTGTTACTTACCTGTTGTTACTGTTATTGTTTAGGCAAGTTGGCCAATAGTGAGAACTTCTCTTCATGGACAAGAGTTTACATGAAACTATTTTTTTTTTTTTTTTTGTTTTACGATATTGTCACATATATGGCACTGAAATGTCTTACAGAAAGTAAAGTAGGAGAAACATAATTCACTGTAATAGAACGAAAGAAAGAAATTGGAGGAGTGTTTCGTTTTGTTTGTAATGTTCAAGTATTTGTGTCCTTTCCTTAAAATTTGTATTATTAGAAGTTATGATTTGTTTGAACTTTGTGTTATTCAATCAAATAGTAGTTTTTATTGACGGTAATGAACTGATTTCTCAGTGCGTGGAACAGTGCAGATATGTATGGATTGTTTCTGTTTACTACTTGGTTTGGCATCTCCTTGGACTTATGAATTTATTTTTCATTTCAATATAGAGGGAGATGGATTCTGGCTACTGCTCTTATCCTCATGGTACGCATGGTTTAAGATCTTCTGGGCGTGGAAAGGTGCAAGATTCCTCAGTTCCCAACATCCGGATTGGATCTTCCTGCAGGCAGGGCAATGCTGAACTGGGAAATTCATTTTTGGCTCTTCTTTCTGCACCCCCATCATTGTTGCAGTGTGATTTTAAGGAACAGTCAAATCTGAAATCTTTCAATGCCTCTTCTAGCAAACTCCCTTTTGATGGTGGAGTTGTAATCAGTACTTCTGTTGGAAGTGGTGTTCCACCAATTGCTAATGGGTTACTATCTGAATGCCAGAGTAACCAAAATGTCCAAAATGGGGCAAGCCCCATCTTCTCATCCAGAGTTGTGGCGAATTCCAATTGTAGTACCAAATATGGCTTGCATGATGGTCTTGAAACTGTGAACGTAAGTCTTCAGAGTTCAGACCTTGCTAAGGCAATTATTCATCAGTTGGTTTCTAGTAATGAGAGAGCAAAGGATTTTTCTTCCATAAAAGGAAAATGGCATAATACAAGTCTGGGACATGCAGCAAAGATTCCTAGCTCATGTATTCCAATATCACATAAAGAGCCCCTACAATCAAATTCTTCTTTGCCTTGTCTTCCATCTGCTTGTACCAGCGAATGCCCTCGTGTAATTTGCTTGGGCGCAAGTAAGTGCTTTAGTTCCTCTCATCAAATAAGTGACACTGTTGTAGATGATATATACTGACATTTTTTGTTCTTTTTTATTGAAAATCGATTCGAGAAGGCGGGAATCTGCTTCTTAGCAATACAGGACTCCTTGGTATTGTTTGCTCATGCCATCATTTTCACACATCTGTTGCCAAATTTTGTGAGGTAGATTTTGCTTCGTTTTGACTTGATTTTTGTGTTCTTTAAATTATGTACAGTCTTCATGTTTTTGGAAACAATTTGCATTGCATTTGGCACTGGTGTTGTGCTTGAAGATTTGTTGTAACTTCTTTATTTTGCAGCATTTAGGATTATATGATGTTAACCCTGGGGATGCTGTTCGTATGGAAAGTGGAGAGACCATTGCTCAGTGGCGAAAGCTCTACTTCCGGAAATTTGGGGTAAGGTTTATATGCTTTTAGCTGCATTTTTTTGCATAAATACTTGAGAGTGTGTCTATGTTATCTTGTAAATACATGCACATAAGTATGAAGGAAAGTTTGGGTATTAACTAAGAAGTATGTGGATAGATAATGTGAAGTGTGATAGTCTAGTTATGAACTAGTTTTTGTTTTAGGAGCCTTTCCCCCTTCCTCCTTGAGTGAGTTTTCTTCCATTTTCTTATGCTTGAAACTTGCTCTTACTTTCAATGGTCCAACTTAGAGCTTTTACCATATTCCTTGAGGTAGAGACAACATGGCATTCTAACTAGCAATGAATTGGTGAATCTTCAATGCTTATGCATGTATGAAATTAAGCATTGTATTTAGTTGAAATTTTTTCCCCAAGTATTGCGTGAATGGAAATCAGATCCCCTTTCCACCCATGTGAGCAAGGTTTGAACTCTAGACCTATTGCACCACCTGCAAGATTTTACCAATTGGCTTTAGTATGATCTTATATCATTTATGCTCTTGGGTTTACTTTGTAAAAAATTTCTAACAACGGGTATTAATAGTTGTTTTCTAAAGCCAATCAATTAAGAATTTTCTAAGTTTTATGACTACTCATTGAGTTATTGTGTCTGTCTAGCTGATAGCATGCAAGGTGTCTTTGTTTATTTAACAAGAGAGTCGGCCTTATGACATAGAGTGGTGGTATTGGTTGTATTTATAGTTACATTCCATAGGCATGCTCACATATGTTATGTTATACTCATGTACAACAACTATTGAAAGTGAGTATTTCTTGGGACCTTATAGTTCTTTAGCTTCTCATTTTTCTTTATCTTTGGAATTGAGAGTTGTATTTCTTATTGGCATGTGTTTGAAGCAACGAGAACACTGGGTGGTGATGTTTTTATGATGCTTTAGATCAGGGTTCCAGATGATCAGACTGGATGGGATTGGCCTGAAGCGTTATCAGCACCGGCTGGTTTGGTGAAATCTAGCATGGCTGCATCCAACATGCCCAACTATTCTGACTTAGCTAAACTGGTCAGTTCATCTGGGGGTTTAATAAAGCGAGGACAGCCCTGGGACAGCATTGTTTATCCAAAGAACCCTTATACTGACAAGAATTCAGTGATTGATGCTTTTCGTGATAAAGATCACAGTAATAGTCGGGAAAGTACTAACCTAGTGATGGAGTGCCAAACGTCTAGGTGTTCAACATCTTCGAAGTTTGTGGATAGCGGACCAGATGGTGGCTTACAGTCCATACATGCTTACATTGATTCTTTTCTCAAGTCCAGAGACCCGTGCATCACCAACCCTGCACAAAATTCAAGAACTTATAATGAAAATTATGATGTCAGCAAAATAAAAAATGCATGTGATCCAGTTATTGCAGAGAGGGTTGCTACTTCATCGAACATAGAGTTGAGGCTTGGGCAACCATATCAACAAAGTCAATCTTCAGGAAATTCAGTTCCATTAGTCACCGAACCAAAGTTATTAGACACAGTTGTTGCTCAACCCAGGTCACTTTTCCTGGAGCAGATGACTAATAATGGTGCGTCATAAAACATTTTTACTTAGCATATTATTTATGGTATGTATCTTCTTTCAATATTAGCATGTTCATATGAATTGCTTTATGGATGAAAATTTGTTGAAGGATTAGTTTTTTAATTATGCAGCAGCTTATTGTGGGGAAAGAGTGGCACTCAGGCAAAAGTTCCAGTGCTCTGCCGGCCCTGCAAATTTATCTGCAAGAAATGTAAGCAATTTGAACATTGGTCGCCATGTATTTGGAATATCTAATGTCACAGATACCACCAAACTGGATAAATTTGATGGCAATGTAACCAAAACTTCTATGGTTCCATCATTAGCACATGTGAGTACAGCACCCGAGATGAATGCAAACTCTAAAGCTAATAATCACATGGTCAGTAGTGATCATATCATACCTAAGTCAGTTCATTGTGAGCCTTATTCTGCCAAGTCTAACCCAGTTCGTGTTCCCTGGACTGTTGTTGATGGTTCAGAAAGGCAATTGAATGTTTCTGAGTTGGGTTTCTTCAGAATTGAGGACAAGGGTAAGGGGGTAGGATGTACTGCAGATGGCTCTTATGCCAAAATAGATTCAGTTTCTAATATCGAAAAGCAGCAGGAGAGCCGGTGCACTTGCCCAGTAGCTATGGGTGGCAGCAAGGATCCTTGTTCTTCTGTTGTGCATGACAAGATCTATTATTCACATCAGTCATCTGGTGTGCCACCAGATGCATTTGATGCTCGAAACCTTTTTAACTATCCCGAGAAGGTACCTTCTCTTGGGAGCAGTAGACATACTGATCATCTTTTTCTCACATCGAAGGGTTCACCATGGGGTTCATCACAACTTCTGCAATCACAAGCAGTCTCGATGGCCTCTCCCCTTGCTACTTCAGCCTCTATGCAGGGGATGGCACCAGCTATCCCAACAGTAGAGGGAACTGGTGTAAGCCCTTATTTGCTTGATGATAATATGAGATTCCTTGCATTGAGGCAGATACTGGAGCTCTCCAAGCAGCAGCAAGCAATATCTTCCCTTGGGATGGACCAAGAGACTGGGAGAACAAGTAACTTTTCTAATGTCAACATACGCCCATTGGTTGGGCCATCAGCATTTGGAGAACAGACACCTGGACCTAATATTACCAGTCAACGAGATAGTTCTGCAGTTGCCATGCTATCACCCACATCTAGTGCTTATACTAAGCTGGGTGTAAATATTGAGAAATCAAGCCCTATAGCAGGCAAGAACTTTTACATTCTCTTCTTTGTTGTAAAATTTGAGTTATTACCTTCTTATGATCCATTTATTCCAGTTGTAGTATGAAGTTGTATGATAATTTTGTGGTTGTCCAGATTTGAACAACTCTTGTGAATTCTCAACTTGGATCTGTGGGAATCCATTGCTTTCTAGAGAAATTGACCTGCAGTGCCAATTTCCTCATGATCCTCCTTCGAATAAACAGCTTCCATTGAGGTTAGTTTGGAACTATCATATTCTTTTAGATCTTGAACCCATAATCACTGTCCATATTATTTCTTCGGGATGTAAGATTCATATGTATAACCTTTTTGATTCCCTTTGTTTTTTTGGATTTGTCAAGCTTATGACCTAGATTTATTTGTATTTTCAATACCGTGTCAGGCTATCCTCTTTAACCCTTTTAACATCAATTTTCAATATAATTTTTCAAACTTGGCAGAAGTGAACACATCAGTTCATCAATTGAAAATGCAAAATGCTATCCAGGAGTATCCTGTGCATATTTTCAGGGCCACTGTAGCTGTACAGCTTATAGTAAGTGTTTGGGTGGCAATTGTGAGTCAAGAATTGGAAATGCTCCAAATACCTTCAAGGACCAGGTGGGAAATGTCAATGGCGTAACCCCTACGTTAGTTGCTTCAGAGTTTGTGAAAGATGGCACTGATCTGAGGGAAAAAATAATTTCCTCAGATCAAAGGGCAAAAGTGACAGGGCAAGTACGTAAGAGTAATGTCTGTCATGCTTCTCAGTGGAAAGATGTGCCAAGCAAGTATAAAGGGGTTTCTACTGTGGCATGTTTAGACCTGTCAGCAGAAGATTTGTTAGATGGGAGAGGAAACATAGATGGACAGCTTGGAGATGCCACTTCTAAATGCTCTTATGGAACCATGAAGATACGTGACTCCTTGAAAGAGCAAGAAATGTCTAATATTTCGTCAGGATGTTCTGCTGCTGCTGTTACCCACACATCAGTTCAGGGCAACAATTTAGATTCTACTACTCCAGATGTTGGGAATGCCAGATATATAAACAAACATATAGTCGATGAAGGATCCGGAATTGACAAATGCTGGTCATCAGATGATGCACTTGAAAGTGAGAGAAGTGCTGAGTTCCTTGGCTCCAATTGTAAGACTAACTTGAGTAAGGAAGGATCTTCCAAAAATATTAACAATCTATCATCTCGTAGTCTTCTTGATGAGCTTAAGCTCCTAAATTCATTGACATGGAAAAAGAACAGGAAGCAAACCCATACCAGGCTTGCTGTTCATGGTAAGATCAATTTCAAAAAAATTGAGAGAGGCGTCAAAACTGGGAAGAAAAAGAGAGCAAGGAAAATCAAGATGCTGGTGCCACAGTGTCCAACTGGAGGCCCTTCTACTGTACCTTACAAATATCCTAAAGGTACGGACTCTTTGCCTTTCTCATCTGAAGATGTGGAAATGCATAACCCATCTTTTCAGGAAACATGTATATCTGGTGCTTGTTCACCTCAACCTATTTCTAAATGTGGAAGGTCATTGTCTTCATCCAAAGAACTTTTTCGTAAAAGAGATCTCCATATGATTTATGATGATAGAGATGGAAATGATTATCAGATAGAAGCTAATCCTTGCAAAATCCACGAGTTTTCTGGTATTAAGGAGTTTGGAAGGGCGTGGACTTCAGATTGTACTAGGAAGTCTCAGATGGCAGAACCAACGCATGTGCACACCAAAGATGGTGTGAGATGCAGGTCATTTGGTTGTATGAAGGCATTGTCTAGTGGTGAGGTAAATATTTGTTCAAGGAAGGTAAGGCCTGTTGTATGTGGTAAATATGGTGAAATATGTAATGAACTGATTGGAGATGTATCGCGGCCTGCGAAAATAGTTCCTCTAAGTAGGATTCTTAAAACTTCTAGAAGAGATACACTCCCAAATACTTGTGACTCTAAACAAACTTTCCCTGATGAGTTGAAGAAGGCTATCTTTTGCGGAAGTGATGCTGGTTACAATGGATTCTCTAACTTGAAAGAAGAAAAAAGTGCGATCCATCATTCTTCTATTTGTAATGAAATGAACGTTGACCTTTCTTTAGAAGAAGATGAGAAGATGTTCACCAATGGTGTTGATGAGGAGAACTCAATGTTGGAGAAAAAACTTGATCATAAAAGCAAGAAAAACTGCAGTAAGTTGAACAGAAAGGTTTTTACTAAATCAAAGCCCAAGAGCAAGGAAATACGGAAGCGCAGTCTTTGTGAGCTAACAGATAATGGTAGTTATACCAATATCATTTTTATGTTTTTTCTTTTGAGTATTGTTGATGCCATTAGTAAAAATTAGAATTCAAGTTGACTGATTCATTGCCAAGAGATTATGTAAATAAGTAAGACCTAACTATTATCACTAAATACTGCTTTATCTACAATAGTGAACATTTACAGTATAATTGCCTACCTAGGCAGTTCAAACAGTCACCTGCACATTCGATTGACTGAAGCTGCTAGTGCTTTTGATTACTACAGTCAGTTCAATGTGTTGATCTATGACCTTTGACCCATGTGGTCAGAAGGTTGATTGACAATTGGTATTTCTGCATGCATGTCATTAAACCTTTTTATTTTATTTATATTGCTAGCCTTAAGGGGATATCCTGCAGCCATCCTGACTTTCTGGTGACTGAACTTGCTAGTGCTTTTCAGAGTTCCAGTCTATTCCAGATGTTGATTTTTGCCCTTTAGTGCTTTCATGGAGTCAGTTAAGAAGAGTGGGTTTAGCTCATTTCACAAGTCAATATAACCTCGTGTCAATTGGTCTTATGTGACCAAACTTGTTTATTTTGCTACTTGGTTCATCCCATGGTTCCTTATTTAACACTTCATTTTACAGTACTAATACTTTTAATTACCAGGAAAAAAATCCACTTCCGAAAGCTTTTCCCTTGTGAAGATCTCAAAATGCATGCCTAAAATGGAAGCGGGGAAAGTTTCGAAAAATGCTGTGGGTAGCAAGCAAAACATTCGTGCATCGAGCGAAGTTAATTCTGAAAAGTAAGGAAGTTGATACTCAAAGATGTCTGATTAGATTATTTATACTGACAGAATTCCTATGAAATATAGTAATTGATATTCGTATGTAATTATGTATATCTATCATAGCTAGTTTAAGTATACTGATGGTTATTTTGATCTTATGGTAATACAGATTGAATCCAGAGCACAGATCTCTTTATGTTATGGATTCAGATGCATTCTGCTGTGTGTGTGGAGGCTCAAACAAAGATGAAATCAATTGCTTAATAGAATGCAGTCGATGTTTTATCAAAGTAAGTGGCAGTTCAGCACTATTTCAATTCTATTTCTTCTGAATCTCTCACTATTATTCCATTAGTAAAACCTTGATATATGAATGTTGGATAACTTAATAACCTTGATTAAATATTAAAATCTTCTGGTCTCTACTTGGGCCAATGTACTAAATTAATAATCTCACTAAATGCATGAGATAATTTTTTTTTTTTTCAAATCCTTTAGGGTCCAATGAAAATATAAATTAATAATTAGTCAAATACAAAAATACTAATAATAATTAACATATAAAATTCATAATATTAAAGGGCTTTTTTCTTGACAACTCTAATTTTCTCACTTCAACTCTTTTAAAAAATTCTAATGATTTGAGAGTAAACATAAAACTAGTGACCAATATATTTGTAGAGTTCATGTTCTATGTACTTTAAGCATCTATTCTAAAACACTTTATCTTCTAGGGTATTAAATTTGAGAAGTACGTACAACAATTTTATGTCTATGTACCATGTATTTACGTTAATTTTTGGGCTTCTATGTACATGTTCATAACATTTATTAATATATATATTTTTTATATACATGATAGCTTTTGAAATTCGTACATGAATCTAGGTCAAAGGAATAATACACATACCAAGATGACACAAGTAATTAAAATATAAAACTCTTAAATTAATAATTATTCATTTATCGATAAATAAAAAAACCCTGCTAAGATGATATTTTTTCTTAGTCCCAACAATATTATTTTATAGAGGTTTTATTGTACTAATTAGGGTCATGATATGTAGGTGCATCAGGCTTGCTACGGTGTTTCCAAAGTTCCTAAAGGCCACTGGTATTGCAGACCATGCAGAACCAATTCTAGAGATATAGTAAGACCTGGCTTTTACTTTAAATAATAAATGTTTTTTCATGCAGTTTTAGGCATTCTGTAATGAACTGCATTTCCTGTGCTGTGTCTTATACGGTGTTGTTGTATAATCTCTTGGTAAGATGCACATAACCCTGCTACCAATGTTTCCAACTAGAGTGTTTTGAGAGTAGTAGGGGTTATGTTTCTCACATCCATATAATGGATCACAACTTCATAGTTATGTAACTTTGGGTCCAAATTTGTTTCCTAGTTTTGAAATTACCATCTGATGTTTTTGAATTTGTTTGAAATTGAGGTGGGGAGAAAAGGGATAGATAGCTGCATTAAGTCCTCAGAGCATATTTGTTTAAGTGTGGGAACATTTTATGATTGATAACCTAATATTTATAATAAAATGTATAGATTTTGCTCGTTTACAAGATTATTCAATGTTTGTCAAACTGCTTTTGATCTGGTAATTTTGGTTTCTTTAGGGATGAGTTTGGCACATTTGAAGTCGCTAGATGCATATTTGATCTCATTTTGTTGCCTACTTACACTGAGTTTCTTTAATACTTCTCACTAGGTTTGTGTTCTTTGTGGTTATGGTGGTGGGGCAATGACTTGTGCGTTGCGAAGTCGCACAATTGTGAAAGGCCTTTTGAAAGCTTGGAATATTGAAACTGACAGTAGGCACAAGAATGCTGTCTCTTCAGCTCAAATCATGGAAGACGATCTCAATATGTTGCATTCCTCTGGGCCTATGCTTGAAAGCAGTATGCTTCCTGTTTCTAGACCTGTAAATACTGAGCCACTAAGTACTGCTGCTTGGAAAATGGATTTTCCAAATCAATTGGATGTTCTTCAGAAATCCTCGGGCAATGCTAATAACGTGAAGGTACATAACAGCATTACTGCTGGGGCATTTGATTCAACTGTTAAGCAGTGGGTTCATATGGTCTGTGGTCTCTGGACGCCTGGAACACGATGCCCAAATGTTGACACCATGAGTGCTTTTGATGTATCTGGAGCTTCCCATCCCAAAGCAAATGTGGTAAGTGTTGAACTTAATGAACTTTTTTGGTGGTAAGCTTTGAATTTAATAAACTCTAGGTGAATAGCATAAATTTGGCCTTAACTTTTAAACCATGGCTGAATTTAATTGTATGTGTAATCACACAGGCAGCTGTCATGGTAGTAAAACACGTTAGATATCTTCATCTTTAGAATAGTTTTTTTTTTTTTTTCACTTGTGCATTATCTGTATCTTCTGCCACATGATCGATGCATTTTTGCAAGTAAATGATAATATTCACTAATGTGCATTTGTCGTGACCTGACAAAAAGCCCATATTTTAGGTATATTGCTTCCTTTTTGTCATATGTAGTGAATTTTTGCTTAAGTTTTTTCAATCAAAGTTCCTGAAAGTCTCATAGGCTTTCTTTGCCATCTCAATAGCGCACAGAATGTCAAAATTTTCCCTCTATTCTTATATATTTTGCTTTTCTGTGAACATCTCTAGAGGCTTTGGATGAATAACCCCTGTAGGCCTAAGTTCAAAATTTTGGAATTTGAATAACATATTTGATTGGAAGCAGAATATTTAATGGATGTGAAGGAATAGCAAATAGGGCAGCCAGCTGCTTTATTTATGCAAAATTGCAAGCCATTCTGTCAAAAGTTTTGAGACTACAAGTGTAAAATAGGTTGTAAACACAGCCTTCAAATTGAGAATTTCTGAACTTCAAAATTTTGATAGTTGCTTGTCTCTCTTGTTCCTGTAATGAATTTCTAAATATGGTAACCTTCTTCCCCAGGTTTGCTCTATCTGTAATCGACCAGGCGGCTCATGCATCCAGTGCAGGGTGGTTAATTGTTCTGTGAAGTTTCATCCTTGGTGTGCTCACCAAAAGGTTTGGCTGATTATAATTCTTCTTGTACTCTGTGAAACTTTTGTCTGATTAATAATTATATTATAATCATGTCATTACTTCATTGTGGAGGTAAAGATTGGTACTTGGTATGCACGTATTTTCCTTTTTCATTGTTTTGCATAAATGCAATTATTCTTTCCAGTTTGAGGGTATTTTCTAGCAAGTTCTGTGTACCTGAATTTTTTTTCCCCTTTTTTTGAAGTCCAAACATAATCTGTATAGCCGTGTTTAGTTTGTTTTTGAGTCACGTATAGCTAATAACTTCATAGTTCCTTTCATACATACACGGTAGGGATTGTCTTTCCCTAGTCCTTAAAACTTGCTTTAGAAGGAAAATGTTGTGACATTTATGATAAATGAATGCTCCAGAAAAATAGCAAGATGTGATCTTTCTTGCTCTTTTTGAATCAAGATATCAGTTGTGGATCATGTATTCTGAATAGCACGAAGAGTAGAGCTGTACGCTTTCTCTGCCTGGAAACTATTACTAATGTTTTTAAAATTGTTCTGATGGTGTTATTCTTTCACTGTTGGCATTTCCAGTTATTTTTGTTTATCTTCTTTTTTACCTCTTTTCTAAAGCTGTTATGATTATAAAGTATAATAATCTTTTGAATGGTCATTTTTTTTCTTTGGCATGTGATTTTCAGTTTCTCTGTTGCACTGATTGCAGGGTCTTTTGCAAAGTGAGGTTGAAGGGGCTGAGAATGAAAGCGTTGGATTTTATGGAAGATGTGTGCTCCATGCCACTCACCCCTTGTGTGAGTCTGGTAGTGATCCCTTTGACATTGAGGTGGTTTGTTCAATAGAAAAGGAATTTACCTGTGCTCGTACTGAGGTATTAGTCCAGTGTTTGATTTTGTGGTTCCCACTCTATTCTAGGGTTTGGGGAGGAGACTAATATCTGACTGCCTGACTTATTATGACATCTTGTACTGATACTTTTGCTCTATGTGAAGGGTTACAAGGGTCGGAAGCGTGATGGATTTTGGCATAACCTTCATGGTCAATCAAGAGGAAAGAGTGCATGCCTTGTTCCCCAGGAGCAGTTAAATGCTTGGATTCACATTAATGGGCAGAAGTCATCCACGAATGGGCTTCCAAAGCTTACGGTGTCAGATGTCGAGTACGATTGTCGGGTATGTTGAAGTTCCTTTATACTGGATAATGGTTGCTTACTCATTTTTTTAACAACAATTGAATTGCATTATAGTTGAGTAAGCACCATTAATAATCGAAAATTCTGTCCTCATAAACGTTCTTTTCATGATGCAAGTGTAATGGTTACTTGAGACTTTTATGTGGTTGATGGAAACACTGACCAATATGGAAAACACGTGATCTTAACTTTATGGTTACATAAGGTGGATGAGATATTGTGGTCTTCAATTGTAATTACACAGCTCAAATTTTAATTTTTTACATTATATTATTTTGATATGATCCTTTTGAACTATAATCCTGTAGTTCTTACAATTAAAAGAATTTGCGGATTTGACCTTTGTTTCAGTACTTATAATGGCATCTTGAACTTTTTTATGATATTTTGTCTGCCAGAATAGTTAAAGGTACTGTCTGTTTTGCTTCCCCCAAAAATACCTATTTCAATTTTTGTTGCAACAGCTAATATTTGTTGCAGCATTTTTCTGTATTAAATTTTAGCAGATATGGTTCCATGATAAATATCCATGTTTGAAATTTTTGTCTTTCTAATTATTTGCAGAAGGAATATGCTCGCTACAAGCAAATGAAGGGTTGGAAACATTTGGTAGTTTACAAGTCTGGTATTCACGCCCTTGGTCTTTACACATCTCGGTTCATTTCCCGTGGTGAAATGGTTTGACCTGCTTGTCTCATGAAATGTAAAAAGTCGTTTTTTCCCTCTGGCTTGAAAGCATAATGCGCTGTTCCTTGTAGGTGGTTGAGTACGTTGGCGAGATTGTGGGGCTACGGGTGGCAGATAAAAGAGAAATTGAGTATCAGTCGGGTAGAAAACTTCAGTACAAGAGTGCCTGCTACTTCTTCAGGATTGATAAAGAGCACATTATTGATGCAACCTGCAAGGGGGGAATTGCTAGATTTGTCAACCATTCATGCCTGGTAATAAACCATTTTACCATTGTCCAAGTAGGTTATGAAACAAATTAACATTGGCCTATGAACTTCAAGTGTTTTAGTTTTCTGATTTAAATCTGATTTCTTTGTTTTGACCAGCCAAATTGTGTTGCCAAAGTGATTTCTGTGAGGAATGAAAAGAAGGTAATTCAGAACTCATATATTACTGTTAGTTGGTCATTGGTTTATTTATACAAATCTGTGAGTGATTCGAGGGCCGGTTTGATTGTATGGTTTGTCTGGATGCTTAAATACTGATATCTTGGTTGCAGGTCGTATTCTTTGCAGAACGAGACATTTATCCAGGCGAAGAGATAACTTATGATTACCATTTTAACCACGAAGATGAAGGTAAGAAGATCCCTTGTTTTTGCAATTCAAAAAATTGCAGGCGATATTTGAACTGATGAGATATCCAACCTTCCATGTAAATAGTCGTCCTTCATTTTTTGTATCACCGTCAATCCAAATGGGTTGTTGATTTTTGGCTTCTGCCTAAGGTAAAAGTATTATCCGAAGTACAAACATTCTGGTAATTTTGTTGATAAAAAAGGTGTACATATAGAAGAGCAGAAGCTACCAACTTTGGAAGAATTCTAATGTATATTGCCCTGCCAAGAAGAGTTCATTTTTATTTTTAAAATAATTGCCTCCGGAAGTGCATAAATGCTTCCGGTATCGACCATCTAAAGTTCGCTGAAAGTTGGAAGCAAACGCTAATTTCCCGTTGCGATGCGTTCGTTCTCCAGAGATACCATGGAATGGAAACAAAACTGGAACTAATAGTGAAGGCACAAAATGGCGAAATTTTAACAAAAAAATGGTTTGACTTTAAACAATCGTAACACTCTTGCTTGAGGAAAAATTGTTTATCGATGCTTGCTTCGGTTGCTGAGGCAATAAGGAAATAGATTACACTTGTTGACTACCATAATATAGCTACAAACTTTTTCACCTTTCCAGCATCAAGAGGGCACACGGCAACTAAATACATCGTTTATTCGTTTTCAAGGATTTCCCCAACTTACGCCCATCTGTGTGCTTAGTCAAACTAACAAGATAGACATGATATTAAAGATTTTTTCTCCCCCCTAATTACCTTTTTAACATTTGACATCCATGTGAATATGGAGTGCTTGCTTCTTCAACTGAAACTGCTTTGTTTCTACGCCACTTCCGAACATCAGTATTTCCACTTTATGAACCCCAGTTTGGAATGTTTGCTGTGGCTTCATTCACCATTTTCACCAAAGTCACCTGCCATTATATAAAACAAACAATACACACGACTCAGGTCACTTATATTCTCGTACCATCACTCTGAGCAAATAAACTCATCTAAAATATCAGTTGCATAAAGTAAAACCACTTCATTTAAAATTATGTATGGACATGTTTGAAATTTCAGTAATCCAAGGAAACAATAGCTCTCTTTTCTATATGCTCCTGTTACGGTACCGATTTAGGTAGGAAACTTAGATTAGACAACGAGATTAAACATACCACACTTTCAGGAACACCAGGTGGAGGAAGGGGAAGGTTTCTTGGGGGCGGAACACTATCATAAGATCTTTTGTCGAACCTCCATTCTCCAATCTTCCCATAAGTCAGATGACCCTGAAAATCAAAGCAGAAAACTCGACACTGTGAGTGGAACAACAGATATCCTTTTCTTGTATATCACAATGAGAACTCCCAACATATCAAAATGTTGGAGCAAATACAATACAAAATCATTTTCTATGGTAAGAAACAGAACAAAGAGAATACATGAGAGATCAGTCACAAACAAAATACCTTCATATCATAGTCACATCCATAAGTATAATGGATTATAAACTTGTTGCTAACTTCTGTATCCCAGGGAGGCTGATATATGTAACAGAAAAGAGGTTAAAGGTGCTTGGAGTGTATTGGAATCAGGAAAAAAATTCACCACAAAGAAGTTATTAGATGAGAAAGTTCAGCAAGATTTTCGAGAAACAAAGATCGTGCCGAGGACAAGCAGTTTTAGTTTTCATTTTTATTTATTTGTTTATGAGAGCGAAATGCATGTTCTAGAGGGAAAGTGGAGCATAAATTGCCATTTAGACAGTGAGTAGCGTCTCATCCAACCTGAATCATGAAGTCTTTGTATAAGATGTTGCCAACACCATTCAGAGCAGAGGCAACAGCATAG

>CsSDG9

CGAAACCCCCATGTAATTGTTTCGCTTCTGCTTAATAATGATAACAATTAAGACTTTTAGAGCAAGTGGTCGAAACGACATCGTATTTTCCGTGTGTACATTTTGTTCATGCTAAAACTTAAAAACCACAACAAACGAGATGCGACTATGCGAGTTCCAGACGACACAGCATTGAAAACTCGATGGCATTCAGCAATGCACCGACGGAGCTGATAAACTGTCACAAACGAACCATCGAGGTAGATGAAGAACAAAAGCAGAATCAATTCCTTCAGTGGGCTCGCTTAATACTCCCATGGCTAACCCCAGGGGAGCTCGCCAACGTATCTCTTACATGCAGAACCTTTTCCCAAATCTCCAAATCCATCACTCTCTCCCGCTCTCTCGATGCCTCCAGGTCCGTCGAGAATTTCCCGATCCCATTTCACAATGCCGTCGACAAAACCCCCTACGCCTACTTCATTTACACCCCATCACAGATAATCCCTCCTCCTTGTCCTGCCCAATTCCCACCACGCCAATTTTGGGCCTCCACCAATGCTGCTGCTGATGCTGAATCAAACTCTTCTCTGAGTCCACTCGGGTTTGACTCAGTGAGTCTTGTCTGCGAGTCAGACGAAAGTGAATCTGGGTGCGACTGTGAAGAGTGTTTTGAGGTGGGACTTGGTGATGGGGTATTTGGGTGTCCGTGTTTTTCGGGTCTGGAGGATGTGGGCATAGTGAGTGAGTGCGGACCGAGTTGCGGGTGCGGGTCTGAGTGCGGGAATCGGTTGACTCAGAGAGGAATATCGGTGAGATTGAAGATTGTGAGGAGTGTGAATAAAGGGTGGGGTTTATATGCCGATCAATTCATAAAGCAAGGGCAATTCATCTGTGAGTACGCAGGTATCGGCAAAATGGAAATGTATGTTGGCATTTTTGTAATATTTGTTTAGAGTTTTTGATGCTTGTGATTTTAATTATACATATGCCTAGTTGCATCTGCTTGATGATTATTTGATACAGTAAGACTTATTATTAGATTTAGCGCGTGCCGTTGTTGGAATTATATTTCTGCTGTGTTACGGTAGTATGGAATCATAAGTTCATAGTTTTAGAATGGGATTGTGCGGATGAAGGAATTACATTTGTTTATTTATTTTGACCAAGAAGAAGACCACCTTCAGTTCGTACAATTCTAGTTCAATGGCAGTATTATCGAATCCCATGTTTCAAATCTTATAGTTTTTATCCCTTATTATTTGGATTAATTTTGCCTAATTGGTAGGCAAATTTTGTTCGTCTAATACAGCTTATATTTTACATCTGCTACGTGGTAGAATAGAGTCTATAACCACTATGTTTTGCATCTACCATAGGGCTGTTTAGCTTGCCGTTAGCCATATCTTCCAGTTCTTATGACTACCATTATATAGCTGCTTACTTTTCCTGAGTTATAGCTTTGACTCTCTATTGTATTTTCTCCATTCAACTAGCCATTGTTGGGCACTTTGGGTGATGTTTTTGATCTTTCTTTCCATTCTTCCCACATCACGGGGTTTATGAAACATTATGGTAATAACTGATTGCTTTGAATTGGTCTAGTGTGTAAAACATTTTCATTTACTTGGATATTTAAGAAATAATTATTGTTTGTTTCTGTGGACATTTGTTAGAAATTGTCTTAGTTGATGTTTGCTTGAATAGGTGAACTTTTGACCACAAAAGAAGCAAGGCGGCGGCAACAAATCTATGATGGACTTGCATCGAGTCCCGGAAATTCCTCTGCTCTTTTGGTCATAAGGGAGCACCTTCCATCTGGAAAGGCTTGTTTGAGGATGAACATTGATGCCACAATAATTGGAAACATTGCCCGATTTATTAATCATTCTTGCGATGGTGGTGACCTGTCAACAACACTGGTGAGAAGCTCAGGATCTATACTTCCTCGCCTCTGCTTCTTGGCTTCAAAAGACATAAAAGAAGGTGAAGAGCTTGCTTTTAGCTACGGGGAAATTAGGGCCAGGCCAAAGGGCCTACCATGTTACTGTGGCAGCACTTCTTGTTTTGGAATCTTGCCTTCAGAGAATACTTGAATCTATCTATTGCAAGATTTACCCCAAGTTCACCCTACTCTATATGTTTAACGAGAAGAATTATCTTGTGTCTTCTTGGACCAGTATATATTTTTCTTAATCTTTGAGACCC

>CsSDG10

CAACAATTCGCAAGACTGCAACAAACAAATTGATTTGAGAAGAAGAAAATTGCACAAGCAACACGCAAGGCGCAAGATAGCCTCAACCAAATAAGCTTAAAAAAATTCACTGACATGTTAAAATATTGCTAACAATTTTAGATAACGCCGAGCGGCCCCTAAAACGCTTTCTTTTCTTTCCTCCTTCCTTCCGACTCTCTGTCACTCCAACGCCATTCTCTCTCTAAACAAATTTTTCATCAAAACCCCCAAAAAAAAAAAAGAAATTTAAAAAAATCATTTTTATTTTATAATTACATTACAGTTTGTTATAATTATATATTTTATATTACTCTGAACAAAATTAGATAGCTAGAGAGAGAAAGCTTCATCCAAACAGATCTCTAAAATTCCTTTTAACAAAATTCGATCAATTTCTCTCCAATTTCTCCAATTTTTTCAGAGAAGAAATAACTTCTCTTTTTATTCGTTTTACGAGAATAAATTAGAGGAATTCTGTCGTTTCCCTTATTGTTTTAGTTTCGAGATTTAAGGTTCCTGAAAAGGAATCTGGTTACAATTTAGGGTTTACGTGAATTTGATAAATTAGAGAAGCGGCGTCGTTTTTGGTTAATTAGATGTGGGTGCGTGCTGCAATTGGTTGATCATATTCATTCATCTTTCTGTTTTGATAGAATGATAATCAAGAGGAACTTGAAATCTCAAATGCCGAGTTTGAAGCGGTGTAAACTCGGCGACTCGGCCAACGAGGACAATGAAAACTCGGCTAAGCGGAAGAAACGGAAAACTAACGGCTATTACCCGTTAAGTCTACTTGGCGTTGAGGTGGCTGCCGGTATACTCCCCCTTAGTTTTCACGGAATTCTTCACTCCGAGAAGGGATTCGCTGCGTCTTGGTGCACGGAAGTGTCGTGTTCCCCCGGCGAGGAGGTGTTGAAATCCAAGGGCTCAGGTTCGGCCGGGTTGAAGAAGCCGGCGGTGGAGGTTTCCAGGCCACCGCTCGTGAGGACATCGCGAGGTCGGGTTCAGGTACTTCCCTCTCGGTTTAATGATTCGGTTATTGAGAATTGGAGGAAAGAGAGTAAGCGTGATGATTGTTATGATGATGAAATGGAATGTAAGAAAGAGAAGTTCAGTTTTAAAACACCCAAGAGTTATAATTCTAATGTGAAGAGTAAGTCGAAAGATGATAAATTTAGATATTATAAGAATTGTAAAAATGGTACATTGTGTGAGGAAGAGGAGGGAGATGAAGGTGGGTTTAGTAGGAGTTTTGATGCTAGGAAGTACTCGAGTTCGAAGAGTTCACTAACATCATTACATGAGCAACAATTTATTGATCTTGACAATGATGAGAAAAGCCCGCCTGAAGACATTGTTGAGTTTACGAGCGAGGAGGGCTTATTGAATGGCGAGAGAAAAGATGATGGGTTATATGGGCCAGAGGACTTCTACTCAGGTGATATAGTGTGGGCAAAGTCAGGGAAGAACTACCCTTATTGGCCGGCCATAGTAATTGATCCTATGACTCAAGCGCCGGATGTGGTATTGAGGTCTTGCATACCTGATGCAGCTTGTGTGATGTTTTTCGGTCACTGTGGGGATGTAAATCAAAGGGTATGGATTGTTTTCTATGTTAAGCTTGTTCTGTTTACTAACTTCGATGTTGTTGTTATTATTCTGAGGTTTTGGATGCTGATTGTGGTGCATTGAATGATTATGAACAGGATTATGCTTGGGTCAAACGGGGATTGATATTTCCATTTGTGGATTTCGTGGACAGGTGTATATCTGCATTGGTGCACATAGTGTTTATTTTTTTTTTTTTTTATTGATTCCTTTTGCTTACCTGAGGTATTTTTTGATGATATAGGTTCCAGGAGCAATCTGAATTGAATGACTGCAAGCCCAGTGATTTTCAGATGGCATTGGAGGAGGCTTTTCTGGCTGATCAGGGGTTTACAGAGAAGTTGATACAGGACATAAACATGGCAGCTGGGAACCCTACTTATGATGAATTGGTTCTCAAATGGGGTCAGGAGGCTACTGGTTCAAACCAGGATCTCGACTATCCGTTTATCGACAAGGCATGTTCCCTATATTGCAATCTTTCTTAAGCCTCTTGTTTCTAAACTTTAGTAACCGTGTGTAGGTTGGCATATATGGCTCACCAAGCCTTAATTCCAAAATTTTTGCATAACCGGGATCTCTTAGTCACCTAGACATCATGCAGGGAATTTGTATGAAATTGATGATAATCTTATACAAACAAGTATCAGGAAATTAGACAGCCAAAATTTTCAAAGATTGTAATATAAGATTTCCTGCATATTTTTTTTTCCTTAATTTTCAATTAGAATAAAATCACTGAAATACCTTCTTTTTAATATGTATTTTGCTATTCTGTGAAAACACATTTCTGCAAATCATTATTTGAGTGCATAATCAAGCAGTGCCCTAAAAGTTGGGATCCTTTTGGTTTTCCAAGAGAGCAATGAACAATTTATAGATTGGTCCTAAGGGGAACCATATTTTTATTTATATGTAGCCCTTTTGATTTTTTTCTTTTTCTTTCTTTTAAGGACCAAAGCAGTCCCCCAACTTTTAAGGAAAGTAATTTCTTTTTCTATATTTTTAAAGAAATAAATTGTTCTCTTTCAATCCAGAACACGTTGAATTTAGTTTTATCGATGTAGGTCTCATGGGCAAAGAATAAAGATAAAAGACCCTGTGATGGCTGTGGCATGACCCTTCCTTCTAAATCTGCAAAGAAAATAAAGGCTTCAACTACTGGAGATCAATTATTTTGTAGAACTTGTGCCAAGGTTTGTAACAAGTGCATCGCCCTCATGGTTTCCTTATTAAGTTTCATTTGCCATTATTGTAGTTCTGTGTTGGTTTTGTGCATTTTCTCACAACTGCATGAATATTTATTTCAGTTAATGAAATCAAAACATTTTTGCGGGATATGCAAGAAGGTTTGGAACCATTCAGACGGTGGAAGTTGGGTAAGGCTATCATTTATCTACTTTAGAGTTTTGGCTTATTTTATTTCTTTGGGTTGGACATAGTGGTAATTTGTTATTCATTTATTGTTCAAATCACCTCACCGGTTAGGTGCGCTGTGATGGTTGTAAAGTATGGGTGCATGCCGAATGTGACAAAATTTCCGGCAGCCATTTTAAGGTCTGTTCATCAGCTTTAGACATTGTATGACTGTTGTGCTTTATCCCCCTGATAATAACATGAACTCCTCCCTCGACTAAATTATTTTTTGCTCTGAAGGATCTTGGAGGTTCAGAGTATTACTGCCCTGCTTGCAAAGCTAAATTTAACTTCGAACTATCAGATTCAGAAAGAGGGCAGCGGAAAGCCAAGTAAGTGATTTCTTTCTTTAATTTTTTGACATTTGTGTTTGAACTCATATTATGCAGCTTTATTTCAGCGATGTCTTTTTTGACATACTTTTGCAGCAGTTGTTTTCCTTGGTCTTTGTTTAGTGATATTCGACCAAAAAGATTGTCTTACTTAATTTTCCTGAAAATTATTATGTGCATTTCAATCTAAGTTGGATGCACGGCTCTACATTTATTGATTGCAATTCTTTGGTACAGAAGCAACAAAAACAATGGTCAATTGGTCCTACCCAACAATGTTACAGTTCTCTGCTCAGGTGTGGAAGGCATATATTATCCAAGCCTTCACTTGTAAGCAAAAATACAACTCATGGATTTTATTTTATTTTATTTTTTATGGCACTTAGCTGCACTTAAATGGGAAATTGAGATATATCCCTCTGTTACTTTTACACCTCTGTGTGTAATTTCTCTTAAATTTCCTCTGTTTCAGAGTTGTGTGCAAATGTGGCTTTTGTGGGACGGAAAAACTAGCTCTTAGTGATTGGGAACGGCATACAGGTTCCAAATTGAGAAATTGGCGGACTAGTGTTAGGGTGAAAGGTTCCATGCTACCTCTTGAACAATGGGTTCGTACAACAGCTGATTTTCACAATCTTTTCATATTTCTTGTCGTCCATGTCTCTGGAATTCTTGGATTGTTTATATTTTTGAAAAATGAATATCTTGTGGGATTGGCAATTACTAATTTACTGTAGTATTTTTGTCTAGAAAAGTGATTGTTTCAATAGAAAACTATTAATTTATATGATGACATTAGATAGAAACCTGAAGTTAGGGTGTATGGACCCAGAATATTTTACTGGTGGTTTCTGTTGCATAGGAGTAAAATTTTATCTGTTGTCAACCTTTGTAGAATCGGGGGGTTGTTGTACTCTAAGAAGGCTCAATTTTCAACATACACTTATCTATCAAGAATTTTGTCAAAACTTGCTGTGCTAGCTTTTGGAATATAATGCTCTTACTAAGTTAGCATTCAACACAACCACTGTACTATGATTTTGTTCTAACTTAGTCCAGTGATTATGACGCAATAAACTAGGGTTTGTTTGAATTAATTCATCTGAAATATTAGAGTAGGTTTTTGTATAGCTAAATTCCATATATTGTAGTGCAGATTAGTTGTGATAGGATTGGGCTGGTAGCATATGAAATTATCAATGTCATTGGCATCCAACTTTTTTAAGCTTCTCTTGTTTTGGAGCATTGTCAACATGAAATATGTCCCTGCCCCCTTTTATGCAAAAACCCTCCGACCCATTCTGGATTTCTTTCCCCATAAAAAAAAAAAAAAAAAAAAAAAAAAAAAACCCATTCTGGATTTCTTTCCCCATAAAAAAAAAAAAAAAAAAAAAAAAAAAAATTGCAGTTACTGTCAATGCAAGCAGACTGGCCTAATGCAAGTCTCTGTTCTAAATGTCTTACTATTAATGTCTAGAAGGGTTCTCATCTCCTCCTAATTGGCTTGTTGGTGTTTTATGCCTTCATTAGAGGCTTTTGAATGGGCTTGCTGATTTGAAGATTAACAAAGTTATGAGTGGAAAATGTTGACTTTATATTCTAAAATCTTACTCTGGAAAAATTCTTTGCAAGATTTGTACTTGAGTTTTGTTCTTGTTTAACTTTGCAGATGCTGCAGTTAGCTGAGTACCATGCTAATACTGTTGTTTCAGCCAAACCTCCTAAGAGACCCTCTATGAAGGAGCGGAAGCAGAAGTTGCTGGCTTTTTTGCAAGGTGCATTTTTTCTTTTAGGATTATAATTTTATGATGGTTTTTTATCCTTTATTGAACCAATCACAGGTTTCCTGTCATTGAAATTACTGATTCTTTTTTTTGTTGTGACAGAGAAATATGAACCTGTTTATGCTAAGTGGACAACAGAACGCTGTGCTGTATGCAGATGGGTTGAAGATTGGGACTACAATAAAATCATTATTTGCAACAGGTATTGCCAATGGTGGTGATTAGTATCAATCAGGTGGTCGTAATTTATTGATATGTAAAATGACAATGAGATCAGGCAATATGGACTCTTGTTGTTTCTTGGATATGTTCCGAACAAATGCACACTAGTACAGGCTCCTTTCTCTGTCTAACTGACTGATTATAGTACAATATTTTTAGAATTTGTAATCATCGTGTTGTGTATGTAGCCTTGGTCTACCTATGCATATTGCATATGCTTTACTAGCTGTTACCCTACTGTTACAGTGCAAGCTACAGAATGACGGAGAACAGTAATACACCTTTTCATCCATTGTCTGACATTTTGCCTTCGTAATCAAAGGAAATCCTTTTTTGTTGCCTGAAGATTATGATATATGTTTGTTTATGATATTGTCATGTTAATGATATCACTATTGCTTATCAGATGTCAAATAGCTGTACATCAAGAATGCTATGGAGCAAGAAACGTTCAGGATTTCACTTCATGGGTTTGCAAAGCGTGTGAAACACCTGATATTAAGCGGGAGTGTTGCCTTTGTCCTGTAAAAGGCAAGTGTTTTAGGCTGTTGAATTTCATGTGTAATAAATATTCTGTGTATTTAGGTCATTGAGATTGTCTACACTGAAGGTGAGTAAAATTAGTTTTATAAAAAGTACCTGTTTTTCCACGGTTTTATATTTCTTTCTCTATGTTCTTCTACTCTACTTGGCTACAGTTACCCCACATTTGGGTTGACCCAGTCATAACTGCTGGCATTACTAAAGACTGAAATCATGTGATTTAATCACAGCTTTCTCACTATCAGATGATTTTCATTGTCGCAGATATTTTGCCTAACAGTTATCGTTTTGTGTAGATATTATTACTGAAGCAGTCAGATTCCCATCATGATTTGATTCCATGACCTTGTTCTGATTAAAGTATGCTTTTTTTCCCCCTTTTTTCACCAGGAGGAGCTTTAAAGCCGACTGATGTTGATTCGTTGTGGGTTCATGTGACCTGTGCATGGTTTCAACCTGAAGTCTCATTTGCAAGTGATGAAAAGATGGAGCCTGCTCTTGGGATCTTGTGTATTCCGTCAAACTCTTTTGTAAAGGTAGAATATCATGAGTATTCGATCAAATTATTATGAATTTTTGCTTGAGGTTTAGTGTTCGCTTTGTGAAGTCGATATTGCAATTTCATAGTCATTTACGCCTGTATACGAAATTATTTTCCCACTATATTTGTGTCATTTTGAAGCAAACTCAGAAATACTTGAAATTTTTATTGGTGAAACGGAAGAATATGTTGGAAAATGTTCGCTATATGCTTACTTGTTAGCCCGATAAAGTGGCCTTTTTTTACCCTCTCCTCTCTCTTTTCCTCTTTCAAAGTTGTTGAATGATTGTATTTTGGGTTGGTTGCTTTTTCTTTTCTTTGTTACTGGCTACATGTATACTTCTCCCATTGTCATGGTTTTGCAATGTTTAGCAGCTTTTAATTTATTATTTTACATTTTTCCTAAAGAAATGTGACGAAGTTCTATAAGTGCAGTCCTATAGTATACGCATTAATATTTGGAACAGCTCATCCCCTGGATATAAATTAAATGATCATGTTGACAAATATTTGTGTGATTGATTTGTCTTAGTCTAAATGTTCATGCTGGATTGCTGGTGATTATTGAGCTGGTTGTTAGTGCAAGAATATAGCTAATTTTTGTATGTGCTTTTTCTCAGATTTGTGTTATTTGTAAGCAAATTCATGGTTCATGCACACAGTGTTGCAAGTGTTCCACTTATTACCATGCTATGTGTGCATCAAGAGCTGGGTATCGGATGGAGGTAGGTTGCTTACTGCTGCATTTTTAAAACAGTTGGCTGAAATTGTGCTTCTGCCTACTTCTAGGCACTTGTCTATCTCTATGTTGTGACCATATGGAACCTGTAGCTGTAATTGCCACCATTATATACTAAAACTTACGTAGTGTGAAGGATGTTAATGATCATATTTCACCTTTCAGAAACTGTAGATTTTTCATTTTAGTGTTTTATTTTATTTCAAACCTTTACATGTGATTATTTATTGGTATATTATGTCAATCTTCTTGTTGCTTGAATGCCAGTAGGTTGAGTAAATTGATGTACCAATAAAATTAAAGCCATTATCTGCCTACTCAGCCTATGTTAATCTCTAATTAGACTGCTCTCTGCTCCAATAAATGTTGCATTGACTAAACCCTATGTTTTGCAGTTGCACTGCTTAGAGAAAAATGGAAGACAGATCACAAAAATGGTGTCATATTGTGCTTATCACAGGTTTCACCTAACACCTCTGAGCAGTACTTGTGTTTCATGAATGCATGTATTTTCTGTAATTCTCTTCTCATGTTTATTCTTTGGTTTTTGATGCAGGGCTCCAAATCCAGATACCTTCTTAATTATTCATACTCCTCTAGGTGTCTTTTCTGCCAAAAGCCTTGCTCAAAATAAGAAGAGGTCTGGTTCAAGGCTTATTTCATCTAGCAGAACAAAAGTTGAAGAGGTTACTGCAGTAGAATCTACTGAGATTGAGCCATTCTCTGCTGCAAGGTGCCGTGTATTTAAAAGATTAAACAATAACAAGAAGGTACCCTATATGTATCTGTTTCTGTTTGACTCAGCATATCTATCTATTTGTTTTCCAGTGATGATACAATAAGTGAATTTCAATGGGTCAGTTTCCTTTTAAGTTTTATTGTTGACATAGTAGCACAGGATGCCTTTGGGTTACATGGTCTTTTTGTCTTGAGTGTTTTGTGGTGGGTTGGGTTTGATTAGGGAACTGATGCTTGGAGAACTCTGTAGGTATTGCCAGGCTAATTGTCCGTCTACCAATTTATTTTTAACCTTCTTTTTTATCTCTAGGCACCATTTCTGTCAAAGAGATGATTGATGTTTGTTCTAAGATGATGAGTGGAATGTCAAACCTATGTGAGCAGTAAGAGGATACAATATAACAATAATGTGCAGCTTAATTTGTTTTCCTTGCTGCATAGAAAAATTTTGCTGGTATCATTCTTGAAGTGCTGAATTTTTCTAATTTTGCATGCATTATGAATTGCTTGGTCTCACTTTCATTTGCCTGCTGTTTTGTTTTCTTAAGTCTCTCAGTTTCATTAAGGAGGGACTTCTTTTTCTTTGTCATCTAACCCTTTTTCCCACCTGTTCTGTTTGGTAGAGAGCAGAAGAAGAAGCTACTGCCCACAAAGTGGGGGGAGCTTGCCATCATTCTTTAGCCACCATGCAAAGTTTGAACACATTCAGGGTAACTCTTGCTCTGTTTTTCCCCTTTTAGATTTTAGTGGAAAAAATTCATCTTATGGTCATTATCTTTCCTTCAACAGGTAGTGGAGGAGCATAAAAGTTTTTCTTCTTTCCGTGAACGCCTTTACCACTTACAGGCAAATATATATGCTTTCATTTTACCCTAGTAATTTTTAACTAATCGATTTGTCTTTCTGACTATTTTTGGGTTGGGAAATTTGTCTCCAGAGAACTGAACATGACCGAGTTTGCTTTGGTAGATCTGGAATACATGGCTGGGGCCTCTTTGCACGTCGGAATATTCAAGAAGGAGAAATGGTATGTGAATTAGAAGAGATCAACGGTATATTGTTTATGAAATCATGTCTTTCATTTAGTTATTGCTTTTTAGTTTATGAAGGTTGAGAAATTTTTCTTTATGAGCGATTTGCCATGGATGCTATGAAGATCCATAAACTTGGTACGTCTTTGAAATTAAAATGGACTTGAAATTCTTCCATTGTTCTTTGGTAGCATCGAAGGTATTGACAAAAATTCTGTTTAAAACTTAAAGCTAAATAACTCTTTCTTAAATGAAATTTTCATTGGAAAACTATAAAATTTTATGTATATATATCTGTGCTCTAAGGTAAAAGTGGGATGCATGATTAATTCCACTGTATGTCTGGCAAACCCTGATGCAGATATATGTTACATAAAAAAATTACACGAAACGTTGTGTAAAGGGAACACAACATTTGTATGTTGGCAGTTCCACTATTACGATACTATTTAAACAAATTAAAAACCAAATGCTTTCCTTCAGGTTCTCGAGTATCGTGGTGAACAGGTAAGACGCAGTATTGCAGATCTCAGAGAGGTGCGCTACCGATCAGAAGGAAAAGACTGCTATGTGAGTTATCTTGTCCTTTTCTGCTTTTGCCCTCCGCATCCTTGTGAAATCATAACTCGAAAATTGCAAAATGCTCAGGATTACTTCATCTCCATTCTTCTTCTTCATCTTCATTTTTTTGGGTTATATTTTCCTCTCTTCCTCTTTTGTTTCTGTACTCTTCATATCTAAACTTTAGCGAGATTTATGTTGCAGCTTTTCAAGATCAGTGAGGAGGTAGTGGTAGATGCCACCGATAAAGGAAACATAGCACGCTTAATCAATCATTCGGTAAGATATGGATATTTAAGTCATATTTGGACTCAACTCCTACATACTTTATCTCCTTTGCCCCTTGATTGATACAGTGTATGCCAAACTGCTACGCGAGGATAATGAGTGTGGGTGATGATGAGAGTCGGATTGTCCTTATTGCTAAGACTAACGTATCTGCTGGTGATGAGCTAACGTATATCTCTTTCTCTCTCTCCCCCTCTTTCTGGGTTTTAGGAACTTCACATAATATATTGATTGTTTGCTATTGGCAGGTACGATTACTTATTTGATCCTGATGAACCTGAAGAATTCAAAGTCCCCTGTTTATGTAAAGCACCAAACTGCAGAAAATTCATGAATTAGGATTACGCAGATGACACAGAGCAAAGTCATTGTTTCTCCAAAAGGAGCACTAACCCCCAAATCCCTCACCCTTGGGGATTTCACAGTTCATACAGCCAATTTGTAATTTTTCTCAGAAAGGAATGTGTATTCTTTTTTCTTCATTTCTTTGTCTTAGATTTTGGGAAATTTTCATGCTTAATAAAAGCAATAGATTTGCCGGCTGTTTGGCTTCTGGCATTTTGTGTAAGTAAATTTGACACAACTCTCATTTAGGTAATTAAACGCTAACCCCTTTTCCATCGCATCCTCGCCGACGCCGACACCGACACCAACACGTTCTTGCGAGGCATCAAGATAAATACAAGTGATTAGTCATTATTTGTACTTTGCTGTACGAATGAGAACAGTCAATGAAAATTTTTGTTTTCTCA

>CsSDG11

TCACTACAAAAATTAGAAATGAAATGATCAGGTAACGACAGTAGATGCAAATCCTCTCTTCGGTATTTGACTTTCCCCTGATCTGTTTGAATATCGATTTCTTATTAATTATTTTATGTACTCTTGAATTGTTGCTTGACGACATCTGTATCCATTTGCATGTTCATGTGGCAGGAATTCATTCTCTTCAACAAATTTAATTTAACCAAAGTTTTGCTTGCCGGTATGAAAATGAAACCTCCTGTGACTTTTAACATTTTTGTTTCCATCTTATGTAGGCTAAGTAGCTTTTATCCATTTTGGTTTAATTTTTATAACTGTCTTCGTCTAATATTAGGAGCTGATATTACTTGAAGAATTTGAGTTAGCATAACTGGGAATTTTGTTTGTAATGGATCAAACTTTTGGGCAAGACTCGGTTCCTGTTTCTGGGCCATTGGATAAGTCTAGGGTTTGGGATGTAAAGCCGTTGCGGCGTCTTGTTCCTGTATTCCCTTCTGCACCCAATTCATCTTCCTCTTCAGCTCCGCCAGGTGCTGCCCCATTTTTTTGCACCGCTCCGTTTGGTCCTTTCCCTTCCGAGGTTTCCCCCTTCTACCCGTTTTCTGCTTCAGTGGGGGCTCAGGGGCAGCCCCAACAAAATGCATCTTTTGGATATGGCAACCCAGTTACTGCTGCTGTTCCAATAACTTCATTTAGGACGCCGCCGCACTCAACAGCTCAGGTAAATGGAGATACTGGGCCATCTAGGAGTTCTAGAGGTCGTGTTCAATCGCAATCCCAGGGCAGTTTTGGAGATGAGGATGGATACTGTGACAGCCAGAATCAGGGTGCCCAGTTTGTGAGTAGATATAGTATGCACATTGCTGATGCGGAGGATAATAGTAAGGGTGGGAGGCGGAAGAATAAGCCCCAGAAGAGGACTAGAAGTGGGCGGGATATCAATGTTACCTTGCCAGATATTGATGTGGATTCAATAGTTAACAATATTCTCTCATCATATAATCTCATGGAATTTGACACTGTACGGCGAACTGATGGTGACAGGGACTCTGTGGGATATATACTTCTGATATTTGATTTGCTTCGAAGAAAACTATCGCAGATTGAAGATGTGAGAGAAGCAATGCCAGGGGTTGCTAGACGACCTGACTTGAGAGTGGGTACAATCTTAATGAATAAAGGGATTCGAACCAATGTTAAGAAGAGAATTGGAGCTGTCCCTGGTGTTGAAGTTGGGGATATTTTCTTTTTCAGGATGGAATTGTGCTTGGTTGGGTTACATCATCCAATTATGGCAGGAATTGGTTACATGGGTCTGACAGTTAATTTAGAAGAAGAGTCCGTGGCGGTTAGTGTTGTTTCGTCCGGGGGATATGAGGATAATGTAGAGGATGGGGATGTGTTGATTTACAGTGGACAAGGAGGGAATATTAATAGGAAAGACAAGGAAGTGACAGACCAGAAACTTGAGAGGGGTAATCTTGCTTTGGAGAAAAGCTTGCGTCGGGGAAATGAGGTACGGGTGATTAGGGGTGTGAAGGATCTGTCAACTCCAACTGGCAAGATATATGTCTATGATGGTCTTTATAAAATCCAGGAGTCATGGACTGAGAAAGGAAAGTCAGGCTGCAATGTATTCAAATATAAGTTTATTAGGGTACATGGGCAGCCTGAAGCATTTATGACATGGAAACTGATTCAGCAATGGAAGGATGGTATCTCTTTGAGAGTTGGGGTCATATTACCAGATCTTACGTCCGGGGCAGAAAACATACCTGTTTCTCTTGTAAATGATGTTGATGATGAGAAAGGGCCTGCACATTTTACATATTTGGCTAGTCTCAAGTATGCTCAACCAGTTGATTCACGTGAAATTTTTGGTGGCTGTGACTGCCGCAATGGATGTGTCCCTGGTGATCAAATTTGTCCATGCATTCAAAAAAATGCTGGCTATCTTCCGTATACTTCAAATGGTGTTCTTGTGACTCAAAAATCCTTAGTGCATGAGTGTGGTCCTTCTTGTCAGTGTCCTCCTACCTGCCGGAACCGAGTGTCTCAAGGTGGCCTGAGAGTACATTTGGAGGTGTTTAAAACTAAGGATAAAGGCTGGGGTCTTAGGTCTTGGGATCCCATCCGTGCTGGGGCTTTCATATGTGAATATGCAGGGCAGGTTATTGACATTTCGAAGATAGAGGAACTTGGAGGTGAAAATGTAGATGATTATCTTTTTGATGCTACCCGTACATATCAGCCTGTAGAGCCTGTGCCTAGTGATGCCAATGGGGTTCCAAAGATACCGTTTCCCTTAATTATAACTGCAAAAGATGTTGGAAATGTAGCTCGTTTCATGAATCACAGTTGCTCTCCCAATGTGTTTTGGCAGCCTGTTCTCCGCCAAAGTGACAAGGGGTATGATCTTCATGTTGCATTTCATGCCATCAAACACATACCTCCCATGAGAGAGTTGACTTATGATTATGGCCTGCCAGACAAAGCAGAAAGAAAGAAGAACTGCCTTTGTGGGTCATCAAAGTGCAGAGGCTACTTTTACTAATGTTTGATCAACAGATGACATTGGATGGGAATATGGTCCTGGCTAACTAATGCATTTAGAAGAAGGTTAGATTTCTCCAACCTACTTTTCTAGGCTTTTACTAGTGATTCCAAATTTTTAATTTGGATGACTGCTGTGAAGAAATAATATTGCATTTTCATATCTGCACTAATAGAATGTGGTTTGTTTGTTAATTAAATTTCCCTAATATCTTATAAAAGTTGAATTATCAAGTAGTTTGACATAGATCCAAACAATGTCATCGTGAGTATGCTTTTTTTATATAATAGTAAATATTTTACGGTCCAATATTCCTAAGACTAGTAATGCTATTCAGCATTCTTAAAACAATGCATAAGAATACTAATACTGTTCAGCATTCTTAAAACAAAGCATAATTCCTGAGGCACCAGATATATGGGTGAGGAGCTCAGCAATGAGCTAACCTTTTTTGTATAGTCTGCGTGGTGACAGGGCTTGATCCTCATCTTGTGGTGGCCTGGCAGGATAAATGGAAGGCATGAAATTAACATTTGGACCTTAACATTTGCATATAGTGCTCAAGGTATTCCAGTTATGGAGATAGTGATTTTACCTTAATTATCTTTTTCTGATTGCTGTGACACCAAAAAACATTGACTTTTTATTAGAGCTGCTGTGAGAGAAGGCTGGTTTTGATATACTGGTTGCAGCCTGAAAGCTTGTCTTGTGTAATGTCAGAAGGGAATAGATTCATTTTTTGATAATGGAAGTGAAGAAAAATTCCCTATTTGAGTTTTTTTAGGAGATTCATAGATTAATTTCTCCGTCTGGGTATGTATTGCTAAACAAGGGACTGAGATACTTATTTCAGTTTTGGTTAGTTATTTTACTTAGAATGAGCAATAACTTGCCTCCTCTCTCCATGGCTATAAGTGTTCATAGGTCTGAGCTTGTGCTTGTGGCTCACGTGCTCATGCTTTTATTTTTTATTATACAAAGTGCTCATAAGTGTTCATTTTGTTCTGATCTGTTATTAATAGTTTGCATGCATCTAAATTATATGAGCCATGGTTCTGTTATTTTATTATTGAATATTTACATGGTCTCTCATTAAATAGAACATCTGGGTGTGCTTAAACCATGTTCTCACTCTTGCTGGATTGCCTGGTCCTTTCCTTAGGTAATCTCTAGTTGCCAGGGGGACAAGGCTGCAGAGTGACTTGAAAATGCAAGTGTTTTTGCTGAATGCATTGTGGTTGAACTGTATTTTAGTTTTACAGATATTTGCAGGAAAGAATGCTGAGAACTATCTTGTTTGGTTTTGTAGTCATCAGCACAAGAAGAAGTTTTTTTGCAGCTTGTTCATGTGCTAACATTTTGAAGATGTAACATAAGAATTTTTTGTGTAGAATCCATCCAAGTTGTAACATGGAACACTATACAGTGCTCCTGTATAGTTTAGTGTAACTTGCTTCTCCCAATGATATTGCCTACACTATTTTGATGCATGTGGGTTTGTTATATAACATGGAGTCAACGATACTCCTGACCTAGTCAAGGATTTCTACTCTGTTAATTTATAATGCGTCACGGTACATACATTTCCAAAAAAGTCTATGCTTCAGGATGATATGGGTGGGGTTTCATTGTGCTTGTATGTATCATTCCTAATTTCATGGAGCCTTAGATGATAGTGGCCCTTTTGCTGTAATTGATTCTGTTGTTGCTTCAAGGTCTAAGAGAGATAAACTAGGGGTATTTTGGTTGCATATATTTTAACGAGATACATAATTGAATGTACACGGGTTGCTTCCCTTGAGCAGCCTAGAGATGAATCAGGGAGTTGGCTTGGAAAAGTCTCATCTTGTGAAGAGGAATTTACAAATGACAAAATTCCCTGCAAGGCATAATTTACAAATGAACTAGCAAGCCACCTGTGATTGTATCAAGTTCACAAAGATTATAATAAAATAATGAGAAGTTTTTCTTGATTGGTATATCATCAATTTTGTATACCCTTTAGTTGGATAGAATGAAAATTTGGCACCAGGTCCAAAAAAATTGTTACCGAAAAGAGAAAAAAAGAGAGAGAGAGAGTTTTGTACCCTTACTGCAAACTTAGTGAAAAGATCTACCCATTTAAAATAATAATAGTAATTATACCCTCCTTGAAAGCATGCTGAAAGCTTGTTTAGACCGAACTTCTTGATTGATTCATCTAATCCTCTGTAGACTTGATTGAATAAG

>CsSDG12

GACAAACAAACCCTAGATCGTTTTTTATTAAAAAGAAAAAATAGTTTTTAATATCTTTAAACTTATGAGATCTTATAAGTTGGACCAACTGCCACCCAAAAATCCACTAAAAAAATTGACAGCCGGTGCGGTGGGAGAAGTAATGGCCTCAGGCGTGTACTTCAGTCGTGGGGCCGACGCGTCTCTGGCTCTGTAAGTTGTAGTGTTATTCTTGCTTGCTTTTTGTATCAGATTTTTCAACTGTTATTTGTCGGCTCGAATCTTGGCACTTGGGATATAACTGGATCTAAAGAAAGCGGATGGCCGGGCGAACGAACTGGTCGCTTCACTCCGGCGGCTAGCTTCTCCGATCATTCCAAACTTGCCGGAGGCAAACATTTATTTACGTCACATCACAAGCAAGAAAAAAATAACGTGCGTCTTGGTTTAAAGGGTATTTTGGTCTTATCATGTCAAAAGATGGCTAATGAAGTTTTTGCTTGCGATTTTCAAGTGACATGAGGAAATAAATGTGATCTGATTTAGAATAAATTCTGTTAATATGATTAGTGGTTAATAAATTAAAAAAAATTAAATTTTAATTATTTATTAATATTAAAATAATAATATACGATCTTTAGTAAATATGATTGGTGGTTAATAAATTAAAAAAAATAATTAAATTTTAATTATTTACTTATACTAAAATAATTGTATAAGATCCGTAAAAAATCAAGAGTACTGATAGGATTTTTATCCAAAAAAAAAAATTGAGCTTAATTCACTTTCTACATTTTTTCCTTAACGACTATTTAAGTGTTTATATTTTATGAAATTTGAACTTGTTATGATTTTATTCCGATAGCATGAGTATTATCTTATTTATTAAGTGGTTGATAATTTTTTTATAAATTTTAACTGAACTTGTTATGACTTTATCTCACCATTCATCATTTGGGATGAAATTTTACATTTTGGGATGTTTAAAGATGCTCACACACAATTTTAGTTGATAGCTCCAATTTTAGTTGACTGCAAGTCATTTGATTCCACATAATTGTGAATTTAAACTGACATTGGCTCAATTCCAAACAATTTCTTGTTCTTGTAAATTCCTTAATTTATTTTTTGTTACTATAAATAACAGTATTCTGGAGGCATGTGTTTTTGTTGCAGTTGGATTGTTTTAAGGTTTTCTTGGTTAGCAAGTAAATAAAATGTGGTGTTTTTTAATTTTCGGTTTGGTGTGTAATATGTGGAAAGGCGGTAGACGGAGGAAAAGTCAAAAGTGAAATCTTGGCACTATTTCATTGCTGAGATTAATGCTGGAAGTGTCATTGTAATACTTCAGGGGGTGTTTGATGTTTGAAAAAGTGGGGGAAAATTTTTATTACATTGGTGTTAGTTGAGTGAAGGCTTTGATTACTCAACCCATGTAATGATACAACTTAAAAGACTCATAATCTCTTTCTTACAACACTGAATTCCTCAGTAGCCAGCCTGCCTGGGAATAAAAGAAGTGCAGATTATGTTAATGCTAATGCAGATTGTGCTTGTGTGTTTTGATTACCCTTTTACAATTTTAGTTCATTCGGATTGCTTTGACATGCAAGATCATACAAAATAAAAATTGGAGGCCACTGAGTACGAAAAGATTCCTAAAAAGTTATTCATCCGCGATGGACAGAGAGAGAGCTGTCAGAGCAGCTAATGCGATGAAGGCAATAGGTATTGTCGACAAACAAGTCCAAACAGTGTTGGTAAATCTCTTAGAGTTGTTTAATTGGAACTGGGAATATATAGAAGCAGAAGATTACCGTGCACTCAAAGACACATATTTCGATTTCAAGGAAAATCAGGTAGTCTGTGCAAAATACTAAACATATACATTCCACAACAACCACAATTAGGTCCATTAGGATGTCAATGGCTTTAGTTACTATTGCCTTAATGATTACTTTTTCTGTTTCCACTCAAACGGGTTTTGTCCTTTCAACTTTTTCTGCATAGGGAGTAGAAGACGAAAATGAACGTGTTATGGGGCAAGACAGGCTGGAAAGACCTTCTAAAAAGTTACATTTGGGAAAGCAAAAAGATCATGTTTCATCAGCAATGGCTAACTCAAGTACGACATTAGCACTAATAAAGGCTGAAATGCCAATGCCTACATCTGGACGGGGAAGTAAGAAACCTTCTCAACTATGTTTAACTGACATAAGAAGGGGATCTAGTTCTCAGATTCCTAATGCACTAAAAGAGGCTGAAATCCCAATGCCTACATCTGGACAGGGAAGTAAGAAACCTTCTCAACTATGTTTAACTGACATAAGAAGGGGATCTAGTTCTCAGATTCCTAACGCACAAGTTAGCTGCAAAAGGAAGGACCTGACTTCATCTCATTTGGCCTTTCGCGGAAGAAAATCAACTCATAATGGAGAGTCACAGGCAGTGTATCTTAAACAGCCACTGGATAAACATGGCTTTTCTCGTAGCTCAAGAAATTCTGCCACGTCTCATTGCAGCAGGAAATCGATTAAGAAAAAAACTATGCAACCTATTAGTAGCGTTACACATCATGCAGAACCTATTTCAGTTGTGTGTCCAGGTACTATTGGTAATATATTGTTTTCATGATTCTGATTCTGGTACCTCCATCGTAGTGCAAAAACTTGTTGCATTCTTCGGCTGTAGCATTAATTTTGTCCAGTATCAGTTTTCCACTCAATAATTATAACTTTTTTAGTCTTAGGCACAGAAATGGTTACAAATTTTTAGGATGGTATGGGCTTTCTAGATCAGTGTTCCTATGAAAGAAGAGGCAATGATTGATGCTGGGCTCTTATCTGCAATGTATGCAACCGCTAACATCATTAACCATGCCCCAAAGTGTTTAGAGAGATGTAGGAGTTGTTACAAAGTTATAAAAATTCAAGCGCCATCGTTTGTAATTTTCCATTGTGTCATGCTCGTTGGTGACAATTATGTTATCATAATTATTATAGAGGAGCCCAATACATTAGCTTAACAAATATTATATGCTGAGTTACATTCTTTTTTATCATATCTGCTTCCTTGATATACCACCATACTTCTCAGGTGTTTCATTAACATTTGTAATTGAGAGAAGCAATGCATCAGTTATGTTACTTGATACCTACATTTTCTTTAGCTGGTCCAGGCTCATCGGCAGGTTCTTCAACAGGAAAACACTTAGTAAAATCTTTGGCATCTCAACATGAGCGTACTGTTAATGAAGATGATGCTTCAGTATCCAATGACAGTGCAAGCAGCAACAACAACTTCAGTATTGCTTCATCAGCAATGGGAGAGGTGAAAATATTTCTGAACTGTAACCCTGCTTTTGAAAGGCCAAACTTTCAGAGTCCAAATTTTGATGCGGTATTTAAATACCTTGAGTTTAAAAATCTCATATCTGGAAAAGTTAAGCCTCAATTTTCTGTTAAGAAGTTACTGGAAGGTTTGTGTGATACTTTTTTGGAACTGGGTAATAAATCCACCAGTGGGCCAGTTGCAATTGGCTCATCTCCTGAAGCTATCATAAATGTTGAGGACTCTCTTTCCGCCACAGGACTGAAAAAACAAGCTTCTAGAGTTTGTGATTCTGAAAGGGATCTGAATAAGAAGAGCTCAAACCGTTCTAACTGTTTAAATTCATCTAATCTGGCAACTGTTCAGCAGCAACCTGTCACTTGTAATGAAAAGAGATCTATCCGTATAACTGACATAGCAAAAGGTTTAGAAAATGTGCGAATTCCATTGGTAGATGAGACTTGCGATGAGGATTTGCCAAAGTTTACTTACATCCCACAAAATGTAATCTACCAAAGTGCTTATGTACACATCTCACTTGCTAGAATCTCAGATGAGGATTGCTGTTCAAACTGTTCAGGAGATTGTCTCTCATTGTCAATACCATGTGCGTGTACACGCGAAACTGGTGGAGAGTTTGCATACACACAGCAAGGTCTGCTGAAAGAGGAGTTTCTGAGCGCCTGTATGTCTATGAAGAAGGGGCCTTGTGAGGAACATCTTGTTTATTGTCAAGACTGCCCAATAGAGAGGTCTAACAATGAATACTGTCCTGAAAAATGCAAGGGCCATATTGTCAGGAAATTCATCAAAGAATGCTGGAGAAAATGTGGCTGCAGCATGCAGTGTCAAAATCGAATTGTACAGCAAGGTATTACATGCAAATTGCAGGTACGAATGACTTAATATTAATTTTCCATTCGGAATTTTGTTTTCTCTTCCCTTTTTGGTCATCGTTACATGTCATTTTATTATGAATAATTTAAAATTTTGTACTTTACGACTTCAGTTGTGAAAACAAATTTTCCACTTCGTAATACCCTATGGCTTTTGTTACTCTTTTTGGCTAGGTATGCTGCTTAAACCAAACAAAAAAAGTGAGTCCCTGCTATAAGAAATTTTTTTGAGAAACCATTGAAGCTCACAGTATATTCACCTTCTTGATCTTTTTTTCTCTTTTTTTTTTTAATTTCAGTTTTTCTGCTTAATAAGATCTCTACAAAAGGCATCGCCGTCCTTTGTCATTTCAAATATCACTGTATCTATCTGAAAACACCAATCATATGATCGTTCTCTTATTACTTTTTCATGCTATCTTAGATATATGTATTGAAACTTTGCAAGAATTTGAAGTAAATTTTGTCTATCAGTCATGTGATTTATAAATATTTATTTCTTGTTTCTTCATCATTTACGGATAATCAGTATGCTCTTATGCTTCAGGTGTTTCTGACTGATAGACATAAAGGTTGGGGTCTTAGAACACTTCAGGATTTGCCTAAAGGATCCTTTGTTTGTGAATATGTTGGGGAAATATTGACTAACACCGAGTTATATGAACGGAATATGCAAAGTAGTGGTAGTGAGAGACATACATACCCAGTTACTCTAGATGCAGACTGGGGTTCTGAGAGAGTTTTAAGGGATGAAGAGGCACTTTGTTTAGATGCAACCTTTTGTGGAAATGTTGCAAGATTTATTAATCATAGGTAATTTTTTTCACTTATTAGTTCTACATACAAAGTCACTGTACCCCAGTTTTGGAGACAAAATGATTTGGCACTTTAGGGACTTTCATATCATTTTTATTCTATTATCTAATCATCCATGTGACACTTGACTGTATTTCTGTTCACTTTATAAGAAAAACTTGGGATAAAAAACTTTGTATACTTAGCGTTATTCTTTCACATCGGGCATATGCATCATGTTTGTTCTAGTTGTTGCATGGGTTCTTTTTCTTTGATAATAAATGCTATTTTGGTCCATGAATGTCGCAGATGTGTTCTGCATGCTGAAATAAAAAGCTAAGATAATGCTACACTTGTTGAGTAGTGTCCCAAAATTTACCATCCCACGTGTGCTGGCATTCATCCATTGGATGGTGGGATGGAATGATGGATAAGTTTAAAAGTTATGAAATTGATCATTCAATCGATGAATGCCACATCAACACTTGGAATACTACTTGGGAAGTCTAGTATTATTCAAAAGCTAATGTTTCATGCTGTACCCATTTAGAGTAAAGAAATTTATCTATATTATCTGTTGCATGGACTTTTAAATGTTTTATAATCATGTTGATGGCTAGCCTTTAAACTTTTCTTCGGAGATTGGACTCCTGTTAGAGATTGTAATTTGGTGCGCATCATACCTCTCTTCTACCATTATTTGTATCCTTCACTGCTCTTTGTCAATATAATTTATTTTTTTTCCCCCCCAATTGTAGATGCTTTGATGCAAACCTGATTGATATACCGGTTGAAATAGAGACCCCTGATCGCCACTACTATCATGTATGGATCTGACTGGCTACATCTGTGCAATATTTCCTACATTCTGCTGTCATAAATTTTGAAAGAAGTGCATCTCATACTTTCTTGCTTGTCTTGTGCAGCTTGCTTTTTTTACAACAAGGGATGTGAGTGCTTCTGAAGAGCTGACCTGGGTAAGTGGGTTTAGTCAGTGCAGTTAACAAATTATCTGTTTGTGAATTTAACTTGGTTGAATGTACTGTCGATCATATGCTTTACAAGCATAGGATATATGTACATACATTCATAGAAGCAATACATGTTTTACGGCATGTCAGGACAGTTCTATTTATAGATTATTTCCCTCTGTTTTTAAATTTCAATTCTACAACCTTATTGAAATGTAACTTATAACTGAAGAGTTCAGGAAATACACATCCAAAATTATTCCAACAGCATTGCAACTCCTGCCAGCTGTGAATCTTTCTAACTGGCAGTAGTGTTTTTGAAAGAAAAGCAATCGGACAATCTCTTAAGACCTTCAAGCAACATTAGAGTTGTAAGGAATGGCTGCAAATGTGTGACACTTACCAACTTCCATTTGCCTAAGTTCTTATAAAGAAACAAGGTTGTTTTCAATCAGCAGGCCTGTTGGCTTTTGTCTTCAATTTTCATGGAGCACTAACACCTCATTGTCATAGACAGTGAGGGAACTACCATATTCTTTCTTTTCTGTTAAGTGATGAATAGATAAGAAAATATTTCTTTTTCCCCCTCTTGGTTAATTATGGTGTTGTAACATTTTGTGCCTCCCTCTGATTATATTTTCTAGTCTATAACTGGATAATTTTTTTCTTGTAGCTGATTAAGAACAACTCAAAAATTGAGTTGAAATTTGGTGACAAAATTAATCAACATTTTGCTCATTGTCGTGATGGTTTGGATGACATTAACTGTAGGACTATGGAATCGATTTCAGTGATCATGATCATCCTATAAAAGCATTTCATTGTTGTTGCGGAAGTGAATTTTGCCGAGACGTGAAGTAAAGAGTTCGTGTATCTCATCTACGATAGTGAAGGTAAAGGATTTCATTCTTTATTTTATTAGAAATTTTCCCTCTGGTTCCATGCTACTGCAGGACTATGGAATTGACCTCAGTGATCATGTTCTGCATGTTTCAGTTTCCTTATCCCCTTGGTTGTGGCTAACTGTTATCATTATTTGCTACTACCGGAATTTAGTTGACTTTTCTGTAGTGCTTGGATGAACTTTGACTGGTGAGGAAGTAATATTAAGCATTGACTGTTCTTGTG

>CsSDG13

AAAATAATAAAATAACAGGTATCCCTGGTGAGAGATGCAAAGGTTTCTTCTGAACACGGCGGGAAAGCGATGATTTAGGGAAAGCTCACTCCCTCCAAAATACAACATGTCTCTCTTTCACTTTTCTTTTAAACCCTAACAACCCCGAGAAAAAAGAAAAAAAGAAAAAAAGAAAAAAATCCGTAGCGCCCTGACGAAAATCCCCAAATCCAAATGGCGTTTCCTCAGTCGCTTCTTCACCTTAAACAACAACGACAGCACCACGAAGAAGAAGAACACCTCGAAGAAGAAGAAGAAGACGACGATGACGACGACGTTCTTCACAAGAACGCCGGGACTCCCATTCGCTACGCGTCTTTGGATCGCGTCTATTCGGCCTGCGTCACCGCCACTTCTTCAACTGCGAACGGCGGTTCGTCGAACGTCATGTCTAAAAAGATCAAAGCTTCTCGAAAGCTCTGCAGGCCGCCCATCGTCAATGTTTACACTCGTCGCGCCAAAAGACCTCGCCGCCGACAACAGCATTCTTCGTTTCTGGAGTCTCTGCTGGGGGCGCGTGAGGCCGAGGCCGAGCGCGTGGACCGTTCTTTTGCTGTTAAAGATGAAATTTGTGAATTTGAGAATACAATTGTCGCTAATGATAATCACCACGACGACCATCATGATTTGAGGGTTCTGAAGAAAAGGAAGAGGTTTGGGAGCAGTGAGTTGGTCAAATTGGGGATTGATTCTATTTCTAGCGTTTTCAGCTCATTTGACCGTCCACGATTGAGGGATTGTAGAAATAATAATGGTAGTAGTAATAATAATAAAATTAACAATATTAATTTGAAGAGAAAGAAGACTGATTCTAATTCTAAGAAAATTTTGTCAGTTTCGCCTACTGCCAAGCGATGGGTGAGGTTAGTCTCTCAATTGCAGATTTCATTTATGCCCGTCAAAGCTGTAATTCTTATTTTTGTTTGGTTGAATTTTCATTATCATGAACTATAAATTGTGCATTAACAGGTTGTGCTGTGATGGTGTTGATCCCAAAGCATTTATTGGGTTGCAATGCAAGGCAAGCATTTGTTTATTTTATTTAGTTTTTAAACACAAAAGTTAGTTTCGTGCTTCACACAAATCAAATCTAAACTAGTTGAGTTCAGGTTTATTGGCCGTTGGATGCAGATTGGTATTCTGGTTTTGTTGTTGGGTATGATTCAGAGAGTAATCGACATCATGTAAACCTTTCCTATCTTTTCTTTGGTTATTTAAACTGCTTACAATTTTCCAAGCTATATTTTTTAATTTGTTGGGCCTTTTTTTTTTTTTTGCCCTAAAATACTATACAGGTGAAGTATGTGGATGGAGATGAGGAAGATTTGATTCTCTCAAATGAGAGGATCAAATTTTATATATCTCAGGAGGAAATGGACTGCTTGAAACTGAGTTTCAGCATTAATAATGTGGATAATGATGGCTATGATTATGATGAAATGGTTGTGTTGGCTGCCAGTTTAGATGACTGCCAAGAACTCGAGCCTGGGGATATTATTTGGGCAAAACTTACTGGTGTGCTCCAAATCTATTTATTTCTTTTTATTCTCATTCACGCGTTGTTTCTTCACAAAAACTGAAGTTAGTACAAGAATGAATCATTTAAATATTAGTTGAAAGAATTTAACACGAAAAGGCTTTTATATCTTAAGATATGAATTGTATGCTCAGTTTTCCATATGGTTAGTGGCTTGTGCAGTTAGGATATCTGAAAACTAAAATCTGTGGCCATCAATAGTGCAAGGGCACCAAAAAGTATTTAATATTATTTGGCCTGAAGTTTACATATTTATTGTAACCTTTGGTACAGAATCTTTTAAATTAATATGTTTTCCACTTGTATGTTTTGTCTATGCATTAGTAATCTGATTTTCAGTTGAAAATGCATAGTTTGAATTATGCTTATCCTCATGCTTGGAAAATTTCTTGCCTTTGGCTGCATATATATGAAGTATTGCTGTCAAAACTCAAACTTGATGTGCCACTGAAAAGAAATTAGTATTCCTATAGGAATATTTGAATCCTAGCTCATGCAGACATTACTTGGGGAAGAAAATGTTGTTTAATTGTTGGTCCTTATTTGGTGATGGCTTCATTTGCAATTTTTCTTTTTTTCCTATCTGTGTATTATGTATCATGTGAATATGATTTTATTAATTTACTTATATTTTTTGGTGCACCAACTATATCTCTTAATTGCTATAGTGCAGGTCATGCTATGTGGCCAGCAATCGTAGTGGATGAATCACTTATTGGTGATTACAAAGGTTTAAATAAGATCTCGGGAGGAAGGTCGATTCCTGTGCAATTTTTTGGTACGCATGATTTTGCAAGGTTTGTGGTCCTTTTGAATTTGTATTGTGTGCAAATAATTTTGTAAGTGTTCAGAGTTACTATATTCATATCTTTTAGGGGCTGACATTACTATTCTTTTGTAATTATCCAGAATAAATGTGAAACAGGTAATCTCATTTCTCAAAGGACTTCTTTCTTCCTTCCACCTGAAGTGCAAGAAACCTCGGTTCACTCAAAGCTTGGAAGAAGCAAAAGTGTAAGTTTATTTATCTGAGCCCATTTATCTTGAAAATATTATGTATCGTGAATTGGCATTTTGCTTCCATCGGTAATTATGCATTTTCAGTTTTTTGTTTTGTGCTCCTCTTATTCTTCCAATTAATTGGCTTCTATGGGCTTGGATGAATCACCTCAAGTGCTGATTTTCTGTCCACCCATTAAATGACTTTACTAATTGACTTGCTTTTTTGTCATCAGAGCTGATGCTTGTTTATCCTCTAGGAAGAATGACACTAATATTTGAGGTTATGAAGAACATTGTTGGATGGGTGTACTTTTTAATTTGTTACAGACTTCACCATCTCGGATTCCTATTCTTTTCTTCCTCTTCAGATGGAATTTCACTAAACATTCTTAATGTTGTCAAGTATATATTAAATTTTCAGATTGCCTTTATTTCAGCACTACCAAGATGATAATTATATCTATTACCTCATTTTATTAGGTATTTGAGTGAACAAAAGCTTCCAAGAAGAATGCTACAGCTGCAAAATGCAATTCGTGCAGATGATGGTGAAAATTCTTGGAGTCAGGATGAAGGAAGTTTAGGTTCAGGTGAAAATTGCTTTAAAGACGAAAGGCTTCAGGGAACACTAGGAAGTATTGGAATTTCTCCATATGTCTTTGGGGATTTGCAAATACTAAGCCTTGGTAATTTTTTTTCCTTTAAATTTCTTGGGATTTACCAATCTTCCTCCTTCTCTCTTGCCTTCTCTTTTCATTGTGGATTTCTTCTGCTCTTGTAAGGGGTAGGCCATTGCTTGAACATGTTGCACATTTGGCCTTTTTCCCAGTGATTTTACTTCTGGGCGTATAAAATTTTCCTTGCAGGAAAGATTGTCAAGGACTCAGAGTATTTTCAGGATGATAGATTCATCTGGCCTGAAGGATATACAGCCGTTAGGAAGTTCACTTCATTAGCAGGTGGAGTAATTCTCAAAGGCTCTCCAAAATCTTCATAATTTGTTTACTGTGTTTCTTTGAGTATCTGATTTTAGTGATAGATAACATTCTATTGGGTACAGATCCACGTGTTTGTAACTCTTACAAGATGGAAGTGTTGAGGGATACTGAGTCAAAGATTCGACCTCTATTTAGAGTCACATTGGATAATGGAGAGCAGGTTAGTTGTGATCTGCTTTGAAACTATCCAAATCACAGGTTTCATCGGCTACCCTGTTTCACATTGTATTTCTTATGCCTTTCCTTTAGTTTTTTCCATTAGTTCATCTTGTATCTCAATATGATATTGCTCATCACGCATCATGGACAAATCTAAATATTCTTAAACATGTTATTTTGTTTTACAAGTGTTGATCCTACCTGATGCAATATTAGTTGCAAAAAAAAAATTTCTTTACTTTTCTTTCTCTTGTGCAGTTCACTGGATCTACTCCATCTACTTGCTGGAGTAAGATATGCATGAAAATAAGGGAGAGACAAAATAATACTTCTGATGATTTCAGCGCTGAAGGTGCCGCAGAGAAAATCTCTGAGTCTGGTTCTGACATGTTTGGTTTCTCCAATCCTGAAGTGATGAAACTTATACAGGTATAACGTTTAATGCGTCTTAATCCTGGAATTTGAAATACTGCATTTAGCTTATTCAAATGTCAATTTTTATTATTCAACATCCATGTTGAACCTTTTTCGATCAGATGCCTTTTCCATTAATCTTTTTTCCCTAATGTGGTGTGGGCGGGATCAGCTTGAATACCTCTATTTGAGGTACATTGTGATATGAATGTGAACCTATAACTGCCACTGTTACTGACAGGGGTTAACAAAATCCAGACCTACTTCAAAATCCTCTTTGTGCAAATTAACCTCAAAATATCGAGATCTCCCTGGTGGCTATAGACCTGTTCGTGTTGACTGGAAAGACCTTGATAAGTGCAGTGTCTGCCACATGGATGAGGTAATAGCTGTATCTTTTTCATTTGTCCATAGGTGGCGCTGTGTAAAACTTTTGGCTTTTTTGTTCTCTTCACGTGTTTTTTCCTTTTAATATCTTTACATTTTCCACAAGCTTCCAATATTATTTACGTTTTTTGGCATTCAATGGTTTAGTTTTGCTGATTCTGTTTTTTTAACTACTAGGAGTATCAAAACAATCTATTCCTGCAATGTGATAAATGCAGAATGATGGTAAGCACTCTTTACTTGCAACTGTGACAGATCTTCAAATGAGTTTAAGTACAGTTTTCAACATCTTCTGTTGGTCAATTAAGCTCGGTATTTATTGTTGACAAAGAGCATTCCTCTGCATATCGAAGGACAAAGCTCTCTCGTTTTCTTTTTTCTGAAAGATTTTAGTCACATCAATAGTTTCTTCAGAAGAATTAATGTTTTGTCAGGTTCATGCTAGATGCTATGGGGAGTTAGAACCTGTTAATGGTGTGCTATGGCTATGCAACTTATGTCGCCCAGGAGCTCCTGAACCTCCCCCTCCTTGCTGCCTTTGTCCTGTTGTAGGTAAGTTGATGTTAGAATGACTCTTTTTGTGTAACGGCATGAATTATGCTTTTATGAACCTCTTTTTGATTTTCCTTTAGGGGGTGCAATGAAACCTACAACTGATGGACGCTGGGCTCATTTAGCTTGTGCCATATGGATACCTGGTTTGTTACCATATAACCTACTGATTGTTCTTTGGTCGTTTTCTTTGTAATAAGTAGATGTATTAACTTGACTAAATATGCTTCAGAAACTTGCTTAACTGATGTCAAGAGAATGGAACCCATAGATGGGCTAAACAGAGTCAGTAAGGTACCTGCCTGTTGTTGTTTATCTTATACTGTTATGTTAAGCATACCATTATTATTACTATTTTCTTAATTTTAAAAATAGTTCTATAACTTTTTTAATTAAGGAGGTTGCTGCTAGAAGAAGTACCTCGAAAATTGTAAAATAGTGCTCTTCAAGGTTTAAAGTTTTCTTGCCTCTCATCCTGCTTTATATGACAGGATCGTTGGAAGCTGTTATGTAGCATTTGTGGTGTATCTTATGGTGCTTGCATCCAAGTAAGCTTATAATTAATCTAATTGTTTTCAATTCAAAGTGATGGTTTATTTTCTTTCTAGTACTTACAATCACTGTGAGTTTATTTTTCTTTCAAGGTTAGTATATAGGTCTTACTTAGCAAAGTGGTCATTCTGTTTTAGTGGCTCAGTCTGACTAACGAGTCGAAATAAATGCATTTAGCCAAAAGCATATTTCTGTATGACACAGTCCAAATATAATCTCAGTTATTAAAAGTGTACTAGGGTAATGTGCAGTAGGTTGTCAATAAAAGTGCTTGCTGATCCAAAGCCATTTGTATATCATTTCTTATAAGTCAATTGTTACAGTGTTCAAACACTACTTGTCGGGTCGCATATCATCCGCTTTGTGCACGTGCTGCTGGCCTTTGTGTTGAGGTATTTTTCCTTGTTACATGCTACTTATATTGCCAGATGTTGTAGTTGTTGAGCGTATGATTCTTGTTTTCTAATTCATTAATTTGCATTTTCTTTTTTATGATTTCGTAGCTCGAGGATGAGGATCGACTCAATCTTCTCTCTCTAGATGAAGATGATGAAGATCAGTGCATTCGTCTACTTTCCTTCTGCAAGAAGCATAAGCAGCCACTGAATGACCGTTTAGCTGTTGATGAACGTCTAGTGCAAGTTACTCGTCGATGTTGTGACTACATCCCACCATCTAATCCATCTGGCTGTGCTCGTAGTGGTAAATAACTCTTGTATTGTGTTATTAATATGATCAATCCCTTCTCAGAGATCCATATAATTTAAATAAATCCATCCGGAATTGAGGGCCAAAGGGTCAATGGCCTAAAAAATGAGTGTTGTAGCCCATCTATAGGATCTGCTGATCTGGATGAAAGCACTCAGCCAATAGAGTTCAAATGACATGCTATATTTAGTCATAAGACATTCTGTGGCTTAATGTTATGGCATATTTAAGAGCTAGACGTTAATTTGACCGGTGAAAGTTGATATTTTGAGCAAGCATTGTCGGTAATGCTTGTTTGTCCATGCAAGTCTTCCACTCACCCTAGTTGTCAACCCAAGGTCATTGAACTGTCACATATTAAACAGTTCTCCCTGAACCTTGCCATGAACTTAATCTTGGCATGTTTAACTGTTATATTTTGGCATTTAACATGCTTCCACAAATTCTGCATGTTATCAAGATGCCATGTGAGAAATTTCACTTTAGCTCTGGTAGTTGATTATCTTGAATGAGGGATGCTGACATGTGGCAGGAAAATATCTCTGTGTTGTTAGATCAAATACTGAATATGACCTTAATTAGGATCTTTCTTTCTATCATTAACAGAGCCTTACAATTATTTTGGAAGAAGAGGGCGGAAGGAACCAGAAGCTCTTGCTGCTGCATCCTTGAAGCGTTTGTTTGTAGAGAATCAACCTTACTTGGTTGGTGGTTACTGCCAAAATGGATTGTCAGGCAACACATTGCCGTCTATTAGAGTCATTGGCTCTAAGTTCTCCTTTAGTCTACACAGAGATGCTCCTAACTTTCTTTCCATGGCTGATAAATACAAGCATATGAAGGAGACCTTCAGAAAGAGATTAGCATTTGGTAAGTGGATATCAATATCAATGAATTCCTATGAGTTGATGATTTATTATTACGAGCAACAGCTTGGTATCTCAGACCGCTAGTTTGCCCAATGTCATGCTTTCATGCTTATTGCAAAACTGCAACATGTGTTTCAATTCCTGGAATACATTACACTTTTATGATGTCAATTTTCAGTATCAACTTTTTTATGATTGTTTTCATATGTACGTAGTACTATTTTTACTAACTATAATAGTACTGGAGAGTCTACCAGCTGAATTTCTTCTAAATTGTGTCTCCTTAGGTCCTTTGGAAACTAATGGTGAATATGACAGTTTGTGAAAGCGAAATTGTTGTACTAGTCGGGAGTAAAAGTCAGTTGTGCAATTGTGAGATATTATGCTATGGGGGAAAAAAGTACCATAAATACAGTGTCTTTACAAAATGAAGCACTAAATGGTTCCTCAGTTATTATCCTGTTTGTCAATAATTTTTCTTCTGAAAATTGATGTTTTTGGGACATCCTACCATGGTCGTCAAAAGCAGCTAGTTATGTCTATCTAAAATGCTGAATAAAATCTATTAGTTGTAATAAAACTTGTAAATGTGAGGATCAGATAGTAAAGTTTATGGGGTAAGAATGTGAACAAAAGTATCAGCTACTCGTCAAAAAACATTGGACCAAATATATTATATATATTCAAAATGTGTCACCATATTCTGTATTTTCTCCACTGATTCCACATTAGGTTGATATTTTTTCAAGTTTTTCAGATGCATCCAAATATGAAGGGACTTTTTTTTTGATGATGAAATTGTAGTGACTAGAGAAGAGTTAAAAAACTTTTATATAATCCTTTGTTGCAGAATGACATGCTTGTGAAATTATGTGGGCCCAGTTTTACTATATGTGATTGTGTTACACTGTTAGTCATCATGCACAGGTTTTTCTACTGAATTTTGTGAGGAATTAATGGTTGTTGATTGAAGTTTTGTATACTCTTAAAGCTTTTCTAGTAGAAGCTTGGAAAATGAGGTACTTGTTTCTTTTTTGAAGGATAGAAGAACTTAACTAATAGTTGGAATGTTATAGAAAAACTCCTACATGAACACTCTGTTCTAGTTTTCTATTCCCAAACTTAGAAAATTCTTGAAAAGAGATCACGGTCTGTGATGTTATTGTCTTCTTTTATTATAACTTATGAATTTTCATGAACAGGAATTACATTTGTAGATCAAGAAAATAATTGCATTTGTAGATCGAGAAAATAAATAACATGCATTGGCCCTTGACATTATCTATGTTATATCTTGCTTATATTCAGAATTTTAAGTGGTCGTTAATTCTTCTTTTTTGTCCTTGCAATTTCAATGCTCTAGGGAAATCAGGAATTCATGGATTTGGCATATTTGCTAAGCATCCACATAGAGCAGGGGACATGGTGAACTGATTCTCTCTTTAAGTTTGGTATAATTGTTTAGGAAAATATATTGGGAATAATCTAAATTTCCAAATTCTAGGTGATTGAATATACTGGAGAACTTGTTCGTCCTTCTATAGCTGATCGGAGAGAGCACTTCATATACAATTCATTGGTGGTATGTATTTAGAATGCATGCATTTGGTTTGCTTATTTTTAGTGCAGTATCATGGTACGACTGTCACCTATAATAGCAATATGATAGTACTTTAAAGTGAGTTAAATCACTAAGTTCCTGAATTTTATGATAATGTTGCAGGGTGCTGGAACTTACATGTTTCGAATTGATGATGAACGAGTCATTGATGCAACAAGGGCTGGAAGCATCGCTCATCTGATTAATCACTCATGTGAAGTACGAATCATCTTTTCTTGCTTGCTCATTCTGTTGCTGAAATTCCATTCATGTCTTACAGAATGCCTCTCTAACTAAGCAGTTGGGAGATGATAGGTTTACTTAGCCATTACTCTAGTCTTAAAGGGCCTGTAGCTAGATGGATGGTTGTTCGTATTCACCCTTTGAACCTTGGGACCGTAGCTCATAGTAAACCTAACACTAGTGTTTAAATCCAACAAAGAAGACCAAATATGTTGCTGACTCCTATTTGGTTTGTGATTTGTGGGGTTGGAGAATTCTGATAGTTTGTTTCTACATTATCCTATCACGCTGAGCTTGTGAATAAGTTGTTTACACGTGGTTTAATCTAACAAAGCAGACCAAAAATCTGTCTCCTTATTGGCATGTAATTTGTACGAAGGGTTGGAGGATTCTGATAGTTTGTTTCTTTACTGTTGTAATGCTCTGAACTTCTCTATGGATATTTTCCAGGCTGTAGATTGTCTCAAGTGACCTCAGCTTAATGTCCTGATTTATTTATAGGAGATGTTTAACTTTGTGAAGGAGGGAGAAAGGCAAGGGTGTTATGGAATTGATGTGTTTTAGCTGTTTTTTGGGTTCTTTAGATGAAAAGAAATGGGAGGATTTTTGGGAGAGGTTAGGAAGGTTCTGATTTCAATTTTCTCATGGGAATAAGATTTTGCTTTCTTTTTAATTTTATTTTCTTACTCTAATGAGGAAAAAGAAGTATTTTATTGAAATGAAGGAGAACATGATATAAGTCGACCCTGTTATATCCTTTTTTCCTTTTTAAATTCTAGGTTAAATAAAAAAAATTTAAAAGACGGTATTACTTGAAGCAGGATGCTATGTGCTATTAACAGTGGTACAGTTGGTTTTAAATGGCTTTTTCTATTGCAAACTGCTGATAATCATTGGTAACATCATCATTCTGCTTATATATGGTTATTGCCGGACATCAAGAATTTTCATCTTAATTTTATTTGCTGCAGCCAAATTGTTATTCAAGAGTTATAAGTGTCAATGGTGACGAGCACATCATTATATTTGCGAAAAGAGATATTAAGCAGTGGGAGGAACTGACATATGATTATAGGTCACCTTCAAGTCCTCTGTGTTTATGTTTATTCCTAAATTTGTTGGGGGTTCTGACTTATCAATGTTGCAGGTTCTTTTCAATTGATGAACAACTAGCATGCTATTGCGGCTTTCCAAGATGCCGGGGTGTAGTTAATGATACCGAAGCAGAAGAGCAAGTGGCAAAGCTATATGCACCTCGGAGTGAGTTAATAGATTGGAGAGGAGATTGAGAGTGGTAAGCAGTTTTAAGACCCATTTGTTGGGTCACATGATTTATTGTCTCTGGAATTCAATCATTTTTCACTTCAAGAACTAAAATATATATCCTGCAATGGTTATTAAGTCATGATATCTCATCACAAAAGTGTGGTAGTATTGGATGTGTCATGTCTGCATCAGAGTAGTGTCAGTTTCGACTAAAATGCGTTTTTAGGGTTTATTCAACGTTCTTAAATGCCAACAACCATACGGAAGGAATACGTCTGTAAACGAGGGAGGTAGGGAGATTTTAAGATCTGTA

>CsSDG14

ATGGTGGTGGTGAAAGCGCAGAGGAGAGTGAGCGCGAGGATTCAAGAGAAGCAAAGAGAGGAGAAGCAACGGGAGGAGAAGCTGCGTTTGGTGAAACGAAGAGTTGATCTTCTCACTGATGAAATTATTGGTAATAATGGAGCTGCTGTTGATGATGGTGACGTTGATGATGAAGAAAAGAGTACTATTAATGCGTTCAGTAAAAGTAATAAAAGAGGAAGGAAGGTGAAAGATGCGGAGAATGTCGAAAAGGAGGAGGAAGTTGAAGAGGAAAAGTTAGAGAAAGGGAAAGTTTCTGTTGAATCGACGTCGTCTGGAGCTGAGTTGGTGGATGGCAATGTTGTAAATTTTGCTGAGAAGAGTGATCATGCTAAAGTTAAGGAGACTCTGAGGTTGTTTAACAAGTATTATCTTCAAGGAGTCCAGGTGTGTTGTTTTATTTTTAGCAAATAGGTATAACAACACATTTATTGAATTGTTTGAATTTTTTTTTGAATGTTTTTGGTTTTTTTTTTTAATTTTATCTTATAGTACAGAGTACAGACTAAATAAATTTTATTTTTCAACTTTATCAAACTTTTAATAACTTTGAAAACGTTCAAAAACGATTTTTATGACGATTCCAAAATCAATTGAGCAACAATTGTTTTTTCCGCTCATCATCAATTTTTTTTTTATAGTCTTTCTAAATATAGTATTAAGCAAATCTCTTTCCTGGTTAGTTGGCTTAACTGATGAAATCTCTTGTGTTTGCTTAAAGGTCTGAGGTCTGAATCCTGCTTTGAGTTTCCATAACACAATACTTTAAAAACCAAAACTCTGGGTTTAAACACATGCCTCTTGTACCAATTAGCTCTCACTATGTGCTACATTGTATTTTTGGATTCCAATATGAGTGATAATTGTTTGTGGTTAATATGTTGAATAATAATATTTTGGGCTACCAGACATTTTGTACCAATTAGCACTCGTGCTTAATGTTATATTCTCTTTTTGTAACTTTTTGGTTCCAATCTGGATGCTTATTGTTTGTGGTTTGTAGGAAGAGGAACAAAGGTGTTGCAGGATAGTGGAAGTTGATTCGAAAGCATCAAAGAAGTCAAATAAGTCAAAATCTAAGGTCAGCATTGGCTTCAAACTTTTTCATTTCTTGCAGATGTTGATGTTGATTTTCTTTTATCTGTTTACTCTTATTCTTTTAATGGTTTAAAAATAGAAAGTTAATATGAAGATTCCATAGAAGTGAATCAATAGAACTATTGAAGTCAAGCTATTAAAGGAATCGAAAAGAGAGAAACACATTGCTGAAAAATCAACCAACTAAACAAGAAAATATTTTTTTTGTTAGGGTAGATAAAGGGAAACATAATCTTGCAAAAGTGATTTCAAAACTTGTGAATATGCCAAAATGTTCCTTCAATGTTGAAGTTCCTCGCATAATGAATCTTCAAATATGCACTTATTTATTTCCATTCAAATAATTTGAAATGAAGTAGCAGTAGAGAGACAAATTCTTTGTATAAACTATTTTATACCAAATGATTTGATAATATTGTATGATTGCAACCAGGAATACATTATTTATTATTGTTAATCACATTGATGTTTTTGTTTTCATTCAACCAGTACTTTTATACCACACGAATTTCTAAACAATAGTTTTTTTCTTTAGTACCACTAAAAAATGTCAACCATGTTTAATATCAGTTTGTATACAGTAGTTTGTACCCTACTACTATTCAAAAGATGATGTCCAATTATGGGAACTTTCTTCCCTTAACCAAGAGCACAAAATTTCTTAATAATCCAAGACATGGAAATCAGAATAACCATTTGAACATAGCTTCCAAGGTGGGAGCATTCCATAATATTTGAGCTAATGGCCAGAGAAGAAGAAGATGGTTTATGCTTTAAGTTTCATTTGCAAATAACATATAGTTAAGGAGAATAACACATCATAGGTCTTCTTCCAGAAGTCATTTAGTCTTTCGAATCTTTGATAGTGTTCAAACACTTATAGTGGATACATTACTTCAAATTACACAATGCCAAAAGATATGTCTTCCAAAATTAATAGCATCGCAATTTTCCTAATATAGCCAAATTTTTCTTTTATAAATAACCCACTTCTATTTCACACTTAGAATGAGTCACCAGATTGCATGACATTAGGTGATCTGAATTGCATAAGAATATTCTCATATGCTTCTCCAATCCAATCTCTTAGCCGCCTTAGTCCTAATCCTGCAAAGAGTTCTTAGTATTTGTAACAATACTTGACAAAAAGCCCTTCTTCAGATACTGTCTTCATGTTCTATTTCATTGGATCCTAAAATATATGGCGCTCTTGGATTTCCACTGTTAAACCTTAAGATTTCATGGGTTTCCTTCTCTCCCCACAATCAATGCAGGAGCATAAATGGTATCAAATAACAGAATTTTGAAGAAACTTTGATTTCAGATCAGTTACAAGTCAGAATTTTGATGTGATAACCAATGTTTCATGTAAATTTCTGTCTTGAAATTCTAGTTTGATGGCTTGAAACTTGTGGTGGGCTTAGAAATCACACAACTTTTATTGTGGGCAAAGTTTTTAGACTAGTTAGTTGTAGTACGTATAACTTATCATGTCAATCTAGGCATTTCAGTCTCTTCCAATTTACCAATTATTATTTGCGTATTTCCAACTGGAATTAAGTTATTCCTCCTGAAATTGAGTGTCTGATTAAGCAGGGTTGAATTTTTTGCCTATTTTATGCAATTTGAAGTCGATATCTGAGTTGTGGTGTTGTTTATCAAAGTTTTAATTATACGTTTATTGAAAGTTAATTTGCATATAAATACAGAAAGCTGGATATAAATTTGCTGAGTCTTGGTCCATAGGCTTTTGGATATTCGATAGTGACGACTGATGACAAAATTTCTGCAGAAAGGAGATGTGCCTGAAGACGAGGCTAAAAACAAGGCCAAGCGACCTGATTTAAAGGCAGTATCCAAGGCAAGTCTATATTTGATGGGCTAAAGGCTTTTTTAGAGTCTTAGCTGCACACATACCAGCAGTATTGTCACTGCTCTCAATCAAAAGTGCCATTTTATTATTCCTGGTCTTTAGTTTGTAATTTTTTTTGTTTTGTTATGTTCAACCTTTCGGCTTGTTTAGATGAATGTATTTTTCTGCAATGGTTTAAATTTATTTGTTAATTGCCTTTGATAATATATTTGTAAAATGACCAAGAAGCTTGAAACTGGCCCTTAAGGCCTTAATGCAGATTTTGACAATGGAGGATTTTGACGTCCATAGATTTGCTCAAGTTTTGGATTTGAAGTCTTACTTTAATTTTAGTATTAAGACCATTTTAATTGCATCTAGCAAATCTATCCTTAGCTTGAAAGCTGCAACTTTACTTGCATATTTGCGTTGTTACAAGAGCCTCAAGGTGCAGATAAAAATTTTATAACCTTTAGCTTCCTTAATTAGTTACATACTGTTGACGTCCCTAATTTTTGCTGCCTATGGATTATGTATGATGGTACATAAGATTTGCAAAATGTCTTGATGGTAGTTATATTTTTGTCAACACTTTCATCTTGTCCTGTTGCTGTATTTGTAGAAGCATATACTACGTTAACAACAAATAATGATGGTTGAAACTATTGTTTATTGTTGCAATATTCTTACGTAAATTAGTATTTTGTCCACTATCCTTCAAAAAATTATTGTTCATTGGCAGATGATGAAAAATAATGAGATATTGTACTCCACAAAAAGAATTGGTGATATTCCAGGTTGGTTTCTTCACTAAATTTTGCCAACATGATGCTGTGTTTCACTGTTATTGAGTTTTATTGGCTATCATATATACCTATTTAACTCTTTTACAAGCTTGACCTGAGCTAACAAGGTCTTGGACTTGCTCTCGATGATAGGTGTTCATGTTGGGCATCAGTTTTATTCACGGGCTGAAATGGTTGCTGTTGGTTTTCATAGTCACTGGCTGAATGGAATTGATTATATGGGGATGTCCTACAAAGGGGTAACACATTTTTCTCTGAGATTTGGGATATCTGAAAAGGAGTATAATTGGATATTAATGCACATTCCTTTTATGTGTAGTTTGTTTCCAATTTGTATTGAATTATGTTTCAGAAGAAGGGTGTTTGTTAAGTGTCTTTTAACCCTATTCTATATTATTTTATTTGCAGGTCATTGGATATACTTTTACCTTTATTTGGACTGTGAAACTATGCGTGATTAACTGTACATTATTATTTACTGCTCAATTTCAATTAGACTTGTATGAATATTGAGAATTTAAGGATGGGATGAAGGGCTTCTTTATGTGAAAATTCCTTTGAAGTAGATGTGTGTGCGTTTATAAATGGCCTTTTGATGTATAAGTTGGTGGTTCCTCTTTTCTTCTTCCCGAAGTACTCTTGAGGATTTATGTAGTACCATCCAAATTTTTACTTTGTTGGTAATGGGGCAATGATAAAGTTTTGGGAAGTCATAGGCATTTGTGTATATCCTATCTTTAATTTGTTTCCATGGATTTAAGTTACTGGTGAACCATGACATGACTGTTAATACTATGGAAGGTTGATGAACATATTTTTGGCTCATGGCTTTAAGACTATTAGCATGAATAATTCGGCTTCGAAGTCCAGACTCTTAATCATAGACAAACTTGTACTGATCTCACCTCCTCATCATAGTGTAAATTGAATCTTAAATTGCATAGGAATCTTATTTGGAAAGGAATCTTATCTAAGGAGTATATGATGTTTATGAAATCTTTAAAGGGTTTTTTCTATGTGATAATTGAACTTCTTTTTGATCAGTTTGATAAGAGCAAAAGTTTATTGTAAGAATAAGATCATAGAAAGATGAACTTTGATGAAACCTTAAATGTTATAATTGTGCTCCTTACCTAAAAGTTTAACTGTTTAGGCTTGTAGGTCAAGTGGTTTAAGTGATTGCTTAAAGAAGATTACTGGGATAAATTGTGTCTTTCAGTGATGCATAGTGTGCAAGAGAGATGTTTAGAGTTTAGAGCATTGGTCAAGGACCTTATCTTGCAGTGCCTCATTAGCACACATCTTTGACTCAACAACTTTAGGGCTTTTAATCTCAGTTTGATGTGGCCGTACTTCTTATTGTATCTTTATCAAAAGAGTTCTTGACTTTAGTAGAAGAAGAGAACCACTATCTTGTGGGGCTCATGTGCAAGAAAAATCATTGATACCTTTTTTTTTTAAATTTAATTTTATTTGGAGGGAAATCGAGGATACCTGGAAACGTTTTGAGGAGAATCTTTGGTTTTCTAGTAATCATGTTTTTCCCACCCGCCGCTGCCCCCTTTCCCCCATTTTTCCTTTCCCTTTGGAATGTGTTCTTGATTGGTGGTTCTATTTTTTGTTATTATCTACCAAGGGTAAAGTCAATCCTTTTGAGTCTTTTCTATATTGAATAATTAGACTACATGATACCGTTCTTTTTCTCCTTGTCATTACACTGAATCACTTCCTTTTATGTCATTCACCTCCCTCAATTTCAGGATTACAAAAATTATATATTCCCACTTGCAGTAGCCATAGTCTTGTCTGGCATGTATGAAGATGATTTAGATAATGCTGAGGATGTTGTGTATACTGGTCAAGGAGGACATAATTTAACTGGTGATAAGCGTCAAATTCGAGATCAAAAGTTGGAACGCGGTAATTTGGCACTAAAGGTTTGTACTTATTTTTTTCCTCTGGATAAATATTTTGTGTTTCATCTTCCCTGGATGTCTTTTCGTCACTAGGGACTAAAAATTGTGTCATAGCATTATTGCTAATATATAAATGCTAAACTGACTCTCTGCCCTAGATTCCCTACAATAATATGGCAACAATGTGAGCATGGTGAAGCCCTAAACCTCGTGTACCACTCATAAGAGATTTTACCAATAAACTAGCACCCTCGCAACCATGCTACATTCTTTATCCACTTTTGCAGTAATGATATCATATGACAACAGTTTCTCTCGTACTACTTTTACATAGGAATATCGATTTTGGCTTCTTTAAAGTTTACTTTTTTCCTTTGGAAAGGAAGCAGAGAAACTTTTCTAGAATAATATAAGATGCAAAAAAGGATTACAAAAGTGAACCATAGATCCGCAATCATAGATCCATGATCTAAAAGGCCTTTTCTTTTAAGTTATAATGCATTGGCTATTTTTTTTATCATGGAAAAGGCTTTTTCTTTATAAAGAAAACTATATTTGATATAATTTCTCGAGACTAATTGATGCTGATTTTATGAATAACTATTTTAGATCTTTATTCTTTATTGCTCTGAATGAATGCAGTTATTACTTGCTGTAACTGGTTTGTCCTTTTAAGGAAACTTAAGGCAATGTTTTGATGTACTTTTCTTTTGTTCTTAAGGGGGGCCTTTTTGTTTTCCCCTTGCCTGGATATTTCCTTTTTGTTTTATTAATTCCAGTGCTCGCTCGCTTTGGGGAGATTTATTAAAAGAAAAATCAATGTTTTGCTAATTACTTTTTGAGCATTGTAAACTAAAAGTCTTTTAAGTTCTTAGTACACATTTGATCATATTATGAAAACAATTTTTGAAAAGCTACAATAGCAAAATGATTCTCTTTCAAGTATTTATCAAATTATTTTTCCAAAAATGGCTCCCAATGTCTTTGGGCTGCTCTTGAGTTTATCTGCGTTGGCATTTTTATTTTTTGGGTCTCATGTTAAGTGTAAGTTATAGCAAAAATTTGTTCTACATGGTTATGACCCCCTAATTAGTCAATTAAGAGGTAAAACACAACTATGACCATTACAACCTGTTTATCAAATGGGTGACAGACATGACACAATTAGACACGTTTAAGAAACTGGTCATTTCTTGTATATTGACACAAATTGACTTGATAGATACATTACACCCATTTTACACTATCTGTTTGGTATCTTTAAAGAAATGTAATCTATTTCAACTTTTTTTTTCTAAAATTTAAAGCAATAGTGCCTAATAATAACAATTGGTCAAATCTTATGCACATTAACAATGTATGTTGTAGTGTAGGTACTGTGGTATGGTACCTGAATTATCTTTAATTTTGTCTGCTTTTGATTGCAGAACTGTGTGGACCAAGATTTGCCGGTCAGAGTAATTCGTGGTCATGACTGTTCTAGTAGTTACACAGGCAAAGTGTACTCATATGATGGCTTATACAAGGTGTTTTCTCAAATTAAACTTTAATGTCGTTCTGTATATTTGCCAATGACATTTGTATTTCTGCATTTTTCGGCATTCAATAATGTTTACTGAGGAATTGGTGAACACTAAGATTATAAGAGATGCTAATAATCAAACTGTAGCATATTTCTTTCCTGGTGGTTAATGGTTTTATTTTGAAGTTAGAGACCAAAACTGTGCTCAATAGTCCAATCAATTGCTATGGACATTTGAATGGTTAAAAAATTATCTTTTGTTAAGAATCTTGCTACCCTTCTCTGCATGTATGATGGAGTTGGCCGATCAAGATTAATCAGAGTTGAAAATTATTTAGTTTGGCCAAATCAGTGTTGCTTTCACTTCCTTGTTTATCTTGCCATGTTGTTACTAGGCTTTTATTAGTGAATTAGATTGTAATATTCTCTTTTTGGGCATGCTGTGAATATTGTTTTTGGGCTAGAGTCGAGTTCTGTCATTCAGCACTTCAGCTTAAGCTTTTTGGGATATTAATGGTGTTTTGACATGGTATTAGAGTTTGTCTAACCTACAGATCCTAAAATGTTCACAATTCTTTTGTTTGTGTTTGGATCAGAGACAAGTGTATGAACTAAATTCAGCCTTGTATTCTGGGCATACTGGTGTTTAAATACTATTGTTGATTAGAGTTTTCATATTGTTGCTGGGCTTTCTGATCTGCTTAAGTTTTTGATACAGCGATAGCAGATGAAGCAATGGTCCTGAAAGAAGGCCACACAGCATACTACTTAACCATGCGTGTGATTGCTCATCTATAATGAGCTTGATCTATGTTCGAATAAGTTGAATAGGGGATAGCCAGCTCTTGAACTCAAATCCTTCATGGAAAATGACTTAATAGTATTCTGTAGAAAATTGCTGCTGGGGCTCATAATAACCCTTTTAATGTGACTTTGGATGACTGCCTGAATCCTATTATGTTGTATTTAATTTTTCTATTGTAATATGACCAGTTCAATATATAATCTTTTGTTTCTAGCAAAAACCAACCAAAAAAAAAAAAAGAGACCAGATTTTTCACAATGTGAACTTTTAATTATACAGTGTATACAATATGCTAGACCTATTTAATGTAGTCCACTGGACTTTCTCTGTTTGCAGGTTGTTAAGTACTGGGCAGAAAAGGGCCTTTCTGGATTTACTGTCTTTAAATACCGCCTGAGGCGGCTTGAAGGGCAACCAATACTGACAACTAACCAGGTTTTTATCTATGTTTCTGGTTGCAATTTTCATACTTCTGGATGTTGTTTTATATTTTGTTGGATTTTGCATTTCGTTTTGTGGTGCTAAATTTTCTTTGATACCTATATTTGATGATTTGCTTTGCATGACGGACTAGTGACCATCCTGATATCGATATTCAGATGTTTGTTCTCTGTAATCACAGGTTCGCTTCATTAATGGACGTGTACCTCAGTCTCTGTCTGAAATTCGCGGGTATCAAAAGAATTGTTGTTTATTTAGAATAGTGTTTATGACATCACATATTTGAAATGTAATTTTATCCCATTCTTTTATTGGCATAGGTTGGTCTGTGAGGACATTTCGGGGGGTCAAGAGGCTTTTCCAATTCCTGCTACCAATTTGGTTGATGATCCACCCGTTGCACCCACAGGTAAGCTGTTGCTGCAATTTAATTATTACTTTGTATCAAAAAAATGCAGAGTTGACTGAGACATTTGGCATTATTTACTTGGTTACTTATGTTTTATTTCAATTTTTTGAGTGACTTTTAAATGTTCCATAACAAAGTATAACACAGTTAAGTGCACAGGCATCTCTATTTTATATATCAAGGACGTGTTATTTGAAAAGAGAAAGAGAATAAAAAGATGTTGAGATTTATAAGTTTATGTTCCATACCCTTCTTAGAGTTTGTAGTGAAATTAATGTCCTATTAGATACTGAAAAGAGGCTTGTATTTCAACATTCTGGCGTTCCTTGTCACCCACGTCCTCTATGTATGTAAATTCTAGGTTCTCTAACCAAATTTTATATTTTTGATAAGAATTATATTTGATTTAAAAAGTGATAGGATTACTGGTTTCCTTTTTAGCATGTCATGATAAGAAATGCTTAACTACGTTCTTTGTAGGCATTTTGTTATTAGGTCAAATTTGTTTGGTTTTTAATCCCAATGATGTGCACAGAGTACTTTATTGGTTCTGTGCTCGTTTTTGTAATGTAAGCATGTAGGTGTTTGTCTTTGATTGGCCTCAAGTTTTGAATAACTGTATTGAGCATGTTAACATGCCCAAAATGAGCTGTTTGTTTATCTTTGGATTTGTTGAAATGCGCATGGCTAAATTCTCTTTCACTTGATTGGAGAAAGATCCATTCTTGTTGGCTTGTTGTTTAAATCCATCTATGAAAGAGGACTGCTAATCTCCTTTTAGTTTTTAATTTTCCCATTTATGTCGAGTTTTCGCATTCATCATGTGATTTAATCATCTTTCATTTATTGAATTGTATTTCTCCTTTTCCTACTAATTTTAGTAAATGTCATTGCTTCATATGGGTACCTTTATTTATTAATTTTTGTTTTGTGGATTACAAATGAGCAAAAATATTGGGAAAATATTCCTCATTCATAGAAGTTGCAATATTTACATTATATATTAGTCTGACTAGGCTACATAAGGATGCTATGGGAAATTAAGAAAAATAAACTATACAAGGAAAGAATAAATATATGTACACAAAATTAGGCTATAATCTTTGACTTTCAACACTCCCCCTCAAGTTGGCTCGAAGATATCTATCATTCCCAGCTTGGAATTCAGTGATTGAAAGACTTGTTTTGATAATCCCTTGGTGAGAATATCAGCTACTTGCTTTTGAGTAGTAACATATGGAATGCATATCAGCCCTCTCTACAGCTTCTCTTTGATAAAGTACCTATCAATCTCAATATGTTTTGTCCTATCATGTTGGACTGGACTATCAGCAATGTTAGTGGCAGCTTTATTATCACAGTACAGTCTCATTGATCCAGTTTTCCACACTTTTAACTCCATTAGAAACATTCTCAGCCACATTAGCTCGCAAATCCCCAATGCCGTTGTCTAAACTCAGCTTCAACACCTGATCTTGCAACCACTCATTGTTTCTTGCTTCTCTAAGTAACTAGATTTCCCCCAGATTTCCCCCAACAAATGTATAACCCGATGTTGGCTTTCTATCAACAAAGGATCCAGGCCAATCCGCATCTGTATATGCCTCAACCCTTAGGTGATTATTCATAGAGAATAAAATGCCTTTTCCTAGAGATATCTTCAAATATTTCAGTATCCTAAACACAGCTTCCATGCGCTCTACATTAGGAGAATGTATAAATTGACTCACAACGCCTATTGTAAATGTAATGTCTGGATGAGTATGACACAAATAGATTAACTTTCCAACTAACCTTTGGTATTGTACCTTATTAACCAAACTACTTTTAGTGCTTGCCCCAAGTCTATGATTGGATTCTATAAGAACATCGCTAGGTTTGCATCTTAACATTCTAGTTTCTTTCAAGAGATCCAAAATATATTTCCTTTACGACAAATATCCCTTTGCGAGACCTAGCCACTTCAATTCTGAGGAAATATTTCAGAGAACTAGGTCTTTAGTTTTAAACTCTTTAGTTAACATCTTAAGTCTTTCAGTTTCATCTCCGTCATTTCCAGTCAAGATAATATCATCCACATACACGATAAGGACAACGAGCTTATCTTTTTTACCATGCTTAAGAAACATGGTATGATCACTATGGTTTTGTCAATATCCACATTTGAGAATAAATCTTGCAAATCTGTCAAACCAGGCTCTTGGTGATTGCTTGAGGCTGTATAAGAACTTTTTCAGCTTGCAAACTTTTCCTCTTTCAAACATATTCTGAAACCTTAGTGGTGGATTCATATATACCTCCACTTTTAAATCTCCCTGGAGAAATGTATTCTTCACATCTAGTTGTTGTAATGGCCATTCTAAATTTGCAGCAAGTGACAATAGGACCCTAATAGAGTTCATTTTGGCAACAGGGGCGAATGTCTCTTGGTAATCAATTCCATATGTCTAAGTATATCCCTTAGCCACCAGCCTAGTCTTCCTAGTCTTGAATCTCTCAATTGATTCATCCGCCTTATGCTTAACTATAAATACCCATTTACAACCCACAGTTTTCTTTCCAGAAGGCAAGTCTGCAAGAGCCCAAGTTCTATTTTCATACAATGCTCTCAGTTCTTCATAAATCGCTTCTCTCCACTTTGGATTTGCAAGGACATCCTGTACTGTTCTAGTAGTAGTTACAGATGACAGATTAGTAGCAAAAGCTTTAAATGATGATGGCAATCTATGATAAGAGATAAACTTAGAAATAGGATGTTTAGTACAAGTTCTAACACCTTTTCTGTGAGTAATAGGCAAATCTAAATCCAAATCAAATGATGGAAGAGAATTTAAATGTAATTCAGACCCAAATTGAACAATACCTGTGTCTTTTAATAACTCTATATTCGGTTCAGAATTTTGGCAATCAGGAGATGTACAAACTGTGTTATTTCATCGAGAATACTCCTTTAGCTTTTGAGCATCCTCTTGATTTTGTTCGGTGGATGGTTGAATTTGTGAAGAAACTTGCTGTTCATCAGTTGGGTTTGGTTGTTTCTGTGGGGAAGAGCAATGTTGTTCCTCTTACTGATTGTTATTGGAAGGTGGCAGATTTTGTGAAGAGCAAACTATCAAAGGAGGAGTTGAATTTGTGATATTATCTCTAAAATATGGCTCACCTGAGTGTGTGGGTTTTGGAAAAAATTTAAGAAACTGATCTTTAATATTATATTCCTCCTCCTAAAGAGAGGTTTTGGAGAAATAACGCTTAGTTTCAAAAAAGGTCACTTCCATGGTTACAAAATATTTCCTAATTGGAGGATGAAAACACTTGTACTCTTTTTGTATAGGAGAATAACTCAAAAACAAATGCTTGAGAGACCTAGGATCAAGCTTACTTTTATGATGATTCTTATCATGAACAAAAGCAACACACCCAAAAACTTTAAGAGGAAGAGAATTAAAGGACCAAGACATGGAATAAAGTTGCTGAAGAAGTTCGGTTGGAGTTTTAAACTTCAAAGTACTAGTAGGCAATCTATTTATGAGATAACAGGCAATTGAAACCATTTTACACCACAAATAATTTGGTACTCCAATGGTAAGCATTAAAGATCAAGCAACTTCAAGTAATTGTCTATTTTTTTTTTCTGTAACTCCATTTTGTTGGGTGGTATCAACACAAGAGGATTGATAAACAATTCCATTCTCAGTCAAATAATTTCCCAACATAGAAGAAAAAAATTCTTTGCCATTGCTAGTTCGAAACACTTGACTTTTTGCTTGAAATTGGGTTTGAACCACTTTGAGCCCATTTGGGAGTGTGGTGAGGTAGAAGCCCAGCTACTAAGGTGTTTGGTAACACTTATAGCTGGGCTTTGGTAGCTTAGCAGATTTTTTTTTTCCAACTTTTACTCCACAACTATAGTTTTTACTTCACAACAGCTGCAGTTTAAAAGATACAACACCTCACTCCCAAACACACCCTTTATGGAAGATTTGGCAAGTTTTAGAAACTTCATACTTTTTTTTTATATTAAATACACCGAACAAACTCGAGTATGATCAGCAATAAAATTGACAAACCACTTAACTTCGAAATTATTGGCAAAACAAAGAGGACACCTTACATCACGATGTATAAGAGAGAAGGGTTGTGAAACTTTATAAGTTTGGGCAGGAAAATGAGTACAATGATGCTTGGGCAATGCACACACTTGACATTTTGAATTGATGTCACTCTTATTCTTAAATAATGTCGGGAACAACTTTAAATATGAAAATTTTGGATGTCCTAATATGAGATGCATTAATCTAATTTCATTCTCACTAGGCACTTGAGAAACAATTTTGTCAACTAAGGATAGATTACTCAAAGCTTCAGGCTCCTCTTCGAAATAGTAAAGTCCATCCACCTCCTTAGCACTACCAATCTTTTTCCCTGTACACAGGTCCTGAAACTCACAATTAGAAGCAGAAAATTTAGCCATACGGTTCAGGTCTTTTGTAAGTTTACTGACAGACAAAAGATTATAAGACATATTAGGGACGTGCAACACTGATTTAAGATCATTGTTTTAGAAACTGAAATTGAACCTTTTCCTGAAGAAAGAGACCCATTAGCGATTTTCACCTTTTGTTTACCAAAAAGAGGTATGTAAGTAGAGAATAAGTTGGCCAAACCAGTCATATGATCGGTTGCCCTTGAATCAATGATCCAAGGGCAAGAAGTATTTGAAGAGATACCGAAAGCATTTAGAAAATTACTTGTTTGGGCTAGATTAGAAGAAGGTGTTTGACTCGTGAACCTGGCAAGTTGTTGACGTTGCTCCTTAGTGAATAGATATGATCCTTGATTCCCTAAGTTTGCATTGGTGTTATTAGTCGCAGCTTGAAAGCCCTTTGAATTTTGTCCAGAAGACTTATTACCCTTTCTTGAGTTTTGTGGCCTTCCATGGAGCTTCCAACAAAACTCTTTGGTATGTCTCAGCTTATGGCAATAATCACACCAAAGTTTATCCTTATCATCTTTCTTTTTGCTCATATTCTTTTGTTCACTTACCAATGCTGAATCAATAGCACAAGTAATCAAGGAAGAATTATCAGTATTAGAGTTGCTCATCATTATTGCACATCATTACCTTTCTTCTACTTTCTTCCCTTCTCACATATGCATTGACTTCCCTAATTGTGGGAAGAGGTTCTCTGCTTAGTACTCATCCTCTAACTTCATCCAAATCTGGTTTTAGACCAGCCAAAAATTCAAACACCCTCTCCTTCTGTAACCTCTTAAATTTTTTACTGTCATCTCTACTATTTCATTCTCCATCATAGTACAAATCCAATTCTTGCCATAGGCTATTCGTGATATTGTAATATGCAGTCCCAGTGTCACTTCCTTGTTTGGTTTCATGAATACGACTCTTTAAGTTTGTACAACTGTGCAGTATTTCCTAGATCAGAATTTGTCTTTGTGACTGCATCCCATAAATCTTGTACTGATGGTAAGAAAAGAAATGCCTGACTATTGTGAGGATCCACTGAATTAATAAGCCATGACATTACCATAGAGTTCTGTTCTTTCCATATTTTATATGAAGGGTCGTCGGCCTCTGGTATGTCTATTGACCCATCATGATAGCCCATTTTACCTTTTTACTTCACATACAACTTCACAGACTGTGACCACTGCAAAAAGTTTTGGCCATTCAATTTGTGAGTTGTAATCTGCAATGACAGATTGTCAACTATTGGCTTGAACATGTTTAACATTGACACTGGAGCTGGATTCGCTCACATCTGACATTTTAAATCAGAATTTTAAAGTCACAGAACAAGATAAAACCCTAAAGGTATTGGCGGCTGAAAATTCGTGGATAAAAAACTTGCCCAAAAACCCTAATGTGGGCTCTAATACCATAAGAAAAAATATTGGGAAATCATTCATAGAAGTTGCAATCTTTACATTATATACTAGTCTGACTAGGCTATATGAGGAAGCTATAGGAAATTAAGAAAAATAAACTATACAAGGAAAGAATAAATACATGTACACAAATTAGGCTATAATCTCTGACTTTCAACAAGAAAGATATTTTATATATATTAGAATTATCTTTTCTTTTCGGTGCTAAGTTGTCAAAAATTTCAGGTTTCACATATTGCAAGTCAATGCAAGTTGCAAAAGGTGTAAAGCTTCCAACTACTGCTATTGGATGTGATTGCAGAGGAAATTGCTTAAATTCCCATGACTGTTCGTGTGCCAAGCTAAATAGTACTGACTCTAAGCATTATGACTTTCCATATGTGCATCGTGATGGTGGCAGGTAAAATTTCCTATGCATGATGGTTTATCGCTGGTTTATTCATATGCCTTCCTATCTGATACTATTTTTGTTTTTGTACCACCAGATTAGTTGAGGCGAAGGCTGTTGTATTCGAATGTGGTCCAAAATGTGGGTGTGGTCCTGATTGCATCAACCGAACATCTCAGAGAGGGTTGAAGTATCGGCTCGAGGTTTGCCACTGTGACTTTGTATTTTGTTATTCCTTGTGTAGAATGTTTTTCTTCATTGCATGTTTAGGCATGCTAAATTAGTGAATGTAACAGGTCTATCGTACTCCAAAGAAAGGGTGGGCTGTTAGATCTTGGGATTTTATACCTGCTGGGGCACCTGTCTGTGAATATATAGGAGTACTCAGGAGGACAGAAGATCTTGATAATGTGTGCGATAATGAGAATAATTTTATCTTTGACATTGATTGCTTGCAAACAATGAGGGGGCTTGGTGGAAGAGAGGTACTGTTAGAGTTCTTTAGTGCATATTTCATTTTTGTGTAATTCAATCACTTTCATAGTCAAGTGGTTCAGTAATCAAGTCTGTCAGTTTTAAACTTAAAAGTACAGACAATATTAATGACCGACTTTTCTGATGGTTGTGCACTCCTTTACTGGCAATGCAATACAATTTTTTTTCCCTGTAACCCAGTTTTATGGTTTCTGCAATAGTAATTTCAGGTGGGTATAATTTTGTGAAATTAATTCATTATTTTATCTATAACTCTTTGCAGGACTTAGGGCAACAATCAGCATGCATGTTTATCTTCCATTTATAACATTTTTTCCCTCTCAATGTTCTACACTTATTCGCTCATTATTTTTTGAGTTCACTGTCAGAATAAAATCATTAGATGATGTCAGTTAAGAATGGATAGGGACCCTTCCAGATCTGGGCTCTCTCAAGCTTTTTCTTAGGCCATGTGCTTATCATTGTTCTTGTATGGCCAAAATTTGTTCTTTTATTGTTGTTTATTTTCTTTGTATTTGCTCGGATAAGATCTCTTACATGTATGAACAGAGGCTAAATTGGAAATGAATCGCTGAAAACTAATAATTTTGTTAATTTCTATTTATGAATGCATATATTCTTTATAATTGCTATGTTCATTAACTGTTTCATCTACTCAGTTGTTATTTCATCTTCATAGGTTTGTTTTTTCTTTTATGCTACAAGGGATAATTATTCATTTGAATTCTCATATTACAGAGGCGATTGCGTGATGTATCCATCTCGACTATTTATAATTCAGACCGACCTGATGATCAGAGAGTTGAAAATACACCAGATTACTGCATTGATGCCGGTGCTGTTGGAAATGTTGCAAGGTTCATCAATCATAGTTGTGAGCCAAATCTATTTGTGCAATGTGTTTTGAGCTCACATCATGATTTGAAACTTGCGCGAGTGGTGTTATTCGCAGCAGACAACATACCTCCATTGCAGGTTATTCAATGTTCCTCGTTCTCTCTTCTTATCTCGGTCATTATAAACAAATTTTCTTTAGACTTTTGTTCATACTCACTTAGAACACTTAAACTTCTAGGTAAAGGCCTATAGATAGTATTTAAACTTTAATAACATACCTCAGCCAGCAATTGAATTTAAAGCTCTACCTTATATCATTCTTTTTTTTGGCAATGTATCAGCTTGAACTTTCTTTCAATCTAGTCTTACCATTTAAGACTAGAATTTGAACTTAACATTGTTCTTGCTGATCTATAGGAGCTTACTTATGACTATGGGTATGAACTAGATAGTGTCCACGGTCCGGATGGGAAGGTAAAACAGATGGCGTGCTACTGTGGGGCAGAAGGCTGTAGGGGTCGGTTATTCTAG

>CsSDG15

ATGAAGAGTTTGTATGGCATGCCGGAACCGAAAGTGAAGGCAGCTTTGAGTCGTCTTCTTAGAATATATCGTGACGAATGGGGTTTTATTGAAGAAAATAACTATGAAATTCTCTTCGAGAACTGCATGATAGAGGATGAACAAAAACAAGAAGAAGAAGAAAAGGTATATATATACTCACTTTGTTGCTTTATGTGATTCTCTATATTCGCAACAACTGTGGATTAAAAGCATTTGCATATTTTCGATTCACTTTAAATGACTTGTGTTAATTTCTCTCCCTCTCTCTCTCTCTTACATAAACATGTGCTTTGAATGACTTGAATTTTACATGCTTATGACGTTTGGCATTTGACAAGAAATCAGAATTGAAGATTGTACAAGTATTGTTGGATGATAGTTCAGAGCCATTATTAAAAGGACATTTAACAGATCACAGTGATGAGCCTTCAACCTCTATCAACAAAAGTGAAAGATCAGCAATATGCTCATCAGAGGATGCGGAGGCTGTGATTGAAGTAAAGGAACCTGTTCAATTATCTAACCACAAAAGAGCTAGGGCCTCGTGTTCTGAGACTGCTACACGAAACATCAAAGGAAAAATCCCAGAAATTGAGGAAACTGCTTGTACGAGAGCATTAACCATTGATAATACAAATGGATTGTTCTCGTACGACAGTTACTCTCGTGAATTTTCCCAGGAAGCGCCTCCTCTTGCTGTTAAATATCCAGGTATCTACCCTACAAATTTTGTTTATGGTGATAATTGTTGAGACTTGTTTTAATGCTAATGTCGAGCTGTTAACCATCACCTTAAGCTTTTTTCTCTATCATTGTTTAAATCAAAGGTATAATCTCATGTTTGATTCCCTCATATGAAAACTCTATCTGTCAGGTTTTTCTTGAGTCCATGTTTTATGTAGTATTTGTTAAGACTTTTCCCTAATTAATACTTATGGTGGACCTTTTATATTTGACTAATTCTTTTGCTCAAGCGGTGTTTTTCTACTACATGGTCAGTTCAATTGGTAAGTTTGTTTGAACGGTATAGAAAGTTTGGGATTTGAATCCCCTCGTACATATGGGGAGCTAGTGTATTTAGTTTCTGTCAACATGACAAATTACTTCAAATAATAAAAGCTTTTTTTTTCTCTAACATGATATTAGAGTCAAAACTTCATGTTTATTTTTGTCTGTGTGAAAACTCTTATTTTTTAATGCTCTTCTTTGAATGTATGTTTCAAATGAGAACGCCGGTTGAGACACTGTTTTTAATATTGAAGTTGGTCTTTACTCCATTAACTTTTGGGGTCTGAGTGGTTTCTCTTACAATAATAAACTTTAGCCATTAGGTTTTGTATACCTCTTCAAGTGTAATAACTAAGCAGGTAATTAGCTAAGATTTCGTAGCAGGTAAATGTTTTATAAGATTGAAGTGTCTGCATTACTTCCACTGTAGGTTGACATACATGATGGAGGTATATTTAAGATTGAGCCATATATTGTGGCTTTTAATCCATATTTGTTGCGGTATAAAACCACTACATGTCAAGTGTAAGTGCTTAAATATTATTGTGGCTTTTTATTCTTTAGTTGTTATACGCCTTCAAAATTTTTGGAAGCTAGATTGACATTGAGAGTAATTCTTTTAGTGATGCTTAGAGGTCAAACCAATTGATCTTATGATTTGCTATTAAGTTGGTAATTTTTTATGCTACGTTTTGTGTTCATTCAAGATGAATATTTAACAAACTTGATATGGATGGTTATTGCAGTGACTGGTGTTTCTTATTCAACTCCATTGCAACTTGTTTCATCCAACAATGGAGAGGCGATACTCTCCATAGTTTACAACTCTGTATTAAACAATTATAAACACTTTCCAAGTTCGGATGTATTCTTGAAAGTGGTTGAAGATAAAATTTGCAAGAAATATGGAATCACAGATCCTAAGTTTCGCATGAAAAAGCTAATGAGGGAATTATGCGAATACTTTTTGGCAGTGGGCGCTAATGTGACCAATAGTGATCTACATTTACCGCCCATTCTTGATACATCACATAAAATTGAAGCAGAGTTTGAGGATAATGGCAAACATTATCACCAAAGCAACTCCAAAAATCAGCCAAGCTCTCCAAATGTTGACGATTGCTGTAGTAAATTTGTGAATCCTCCATTTAACGTCACCAAGAATCCCATTTTTGTTGAAGATATAACCCGAGGAGAGGAAAAACAACCAATTTCCTTGTTAAATGAAAATGGAACATCGGAGCTGCCCAAGTTTTTGTATATCTCAAAGAACACAGTTTATAAAAATGCTCATGTGAACTTTTCACTAGCTCGAATAGGTGATGAGAATTGTTGTTTAAACTGTTCAGGGAATTGTCTGTCAGCGCCGGCAAATTGTGCCTGCACTAGTGAAACAAGGGGTGACTTCGCTTATACAGCCGCAGGACTACTTGATGAGAAGTTTTTAAGAGAAAGTATTGCTATAATTAGGAGGAAAAATGATAAAAAGCATCTTTTTTACTGTGAAAATTGCCCACTTGAAAACAGATTGGTGAATGGTAACAGGAACCACAAAAGAAAGAGATCTGTAAAGCCATGCAAGGGCCATTTGATGAGAAAGTTCATTAAGGAATGCTGGGCTAAATGCGGATGCAGCTTGAATTGTGGAAACCGTGTTGTTCAGAGAGGTATCACAGTGAAATTGCAGGTAAATATTTCAGCTCTTTCACTTCTGCATTTTTTTTTTTTTAATCTTGAGTTATGAGTAGCATATCATTAATTGAGGCTTGTGCACAATAGTCTGCACAAAGAGTTCGTGTTTATTTATAGTGAAGCTAGCTGTTGTTGTCAGGTGTTCCAAGCATCTGAAGGGAAAGGATGGGGTGTTAGAACTCTTGAGGCATTGGAGAAAGGCACTTTTGTTTGTGAGTATGTTGGCGAAGTTGTAACAAACCAGGAACTAGATGAGAGAAATGAGGAATTTTCCGGTGATCGGCATACATATCCAGTGTTACTTGATGCAGATTGGGCTTCTGAAAGATTCTTGAAAGATGAAGAAGCTCTTTGCTTGGATGCAACGAAATTTGGAAATGTTGCAAGGTTTATAAATCACAGGTACTTTTGTCACACTTTTGAGTCAATGCTTTGTACATTTATTTTGACGAAGAAGGACACATATGTCCTAATATCTTTTAATTAATTACCGATCACACCCTTAACGTTAAACCTGTAATAGAAAAATATAGTTTATAACTTTATGGTCTTTTAATTACTACTATTGTCAGTTTCATAGCATGTGTTTGTCAAAGGAAGGAACAATTTTATTTGGTGAAATTCACATTTTATCCTTTAATAGTAAAGTAGGAACAACTAAATTATAATGACAAGGACGCACTAGGTAATTAAAAAAGAGGTCTGGTTAAGTTGGATTAATTCCACAGTAGAGCACTTCTACTGACCGTACGATCATTGAAATAAATATTAAATTTGTACTTATTATTATGATTTAACAGTCATCATCAGTACTTCACCATAGAGCTTGATCCAACTTAGCTACTGTCTTAAAAAAGATTTGAGACTTGAGTAATAAAAACGAGGAATATTACAGGCCTATTAAAGATCATTCTCCATTCTTTATTAGAAAAGTTTTGTTTTTGTTGCTTAGTTACCACTTAATTTAAACTTTTTTCAACATGTATATCTATGTGTTACAATTTACAGATGTTACGATGCTAATTTGATCGAGATACCTGTAGAGATAGAGACCCCGGATCATCACTATTATCATGTATGTAATCTCAGAATTTCATTGTTTTTCGTTGAATCTTATTATGTAGAAGATAACTAAAACTTTCTACAGGTGGCTTTTTTCACAACAAGAAAGGTCGAAGTGAATGAAGAATTGAACTGGGTAAGTAGATTTTACCTCTTAGAGTGTGCATTATTTATCATGTGGTAATTGAAATGTTTAATTCTGTCTTGATTCATGGTTTAAGATTCAAAATTCCATTGGCCGCAGGATTATGGTATCGATTTTAGTGATGAAACCCATCCAATCAAAGCATTCGACTGCAAATGTGGAAGCTTTTTCTGTAGCATGAAGTCACAATCTTAAGTTTTAAGTAAATCTTGTTCTTTTGTCTTATATCTGAATGGAACTGAATTGCTGAATTGCTGATCTCAAAAAATGCCCTCTTTGTTAAAGAATTAACCTTTGTTCTGCACACTATGTCCGTTCTTGCTAAAGTAAATTGGTACATAATCGTTATTTCATCTTCAGGAGTCCATGTTCAACTACACCGTTAAGCCGTGGAATCGCTTTTCTAGCAGATTACTCATCAAAATTCTGTGATTTATCTCTCTTTTGTAATGCATTATTCTGTAACCTTCGAACCCCATTTTAATTTTTTCTAGTTTAAATCTGTGTGTGTTGATTTATCAAATTCGGTGCCATTGAACCATATCTTTAGAGCTTCCGAGATAATT

>CsSDG16

CACGCATCGAAGACTCGTGTCGACAGCTTAGCTCCCACGCCCCTCACGAGCGTGGCCATTTACATCAACACCCCACCCGAGCCTTTAAACTCTTCAAAAAAAAAAAAAATCACTTAAAGTTCAAACCTCCATTCAGTGCCCTAATTCACAGTCTTACTCTTCACTTAAAATCACTCTCAGCAATTTCTCAATGCAGGTATATTCCTTTCTCAAAAAAGCATGATGTCGTTTCCCTTTGTACGATCCATCTGTCCTTCGACTAAAAATGCACTTTTTTGTTTAAATTTTGAAATTTTTGTTTCTGAAGTCGCCTTTTTCTGTTCTGATGTTACAGTGCTGCTCCCGGAGAAACTATTTTATGAGTATCATCGCTTCTGCACGGTCGTTTTAGGATTCGCTGTTTTCGTTAGATTGCTACATTTCTGCTCTTGGTAAGCTATTTTATCTCTCTTTCCTTGCCTGCACTCGAAAAAGTAGCCGTATCAAACATTAGCGATGTATGCTCTTTTAGTCCGTACAAGCTGAAAATTTAAAGGGTTCTTTTTGTGTGTTTCTTGTATCCCCTTTTTGGTTCTTTAAATTAATTATTATGAAATTTGGCAAAATAGTGAGACTTTTAAAAGGCAAGGAAAAAGAAAGTAAAAACTTGTATTTTTGTTTTTTTTTTTCTAAATTCAATCTTGGATGCAAACTGTGGAACTTTTGTAGTATTCGATACTAGTAGTATAGGTCATGGGTTTATTCTTGTATTTAGAAATATACCTGTAGTTCTTACAATGCATGTGGGTTTTATTTTGGGTAATTGTTGAAATTTTGCCTGGAAAATTTCTTCTTTTAGATTTAAGGTCTCATTCTAGTAATATTTTTTGGTACCAAAATGAAGGCAAATATTTTTTTGATCTCTTTATCATAATCAAAAGCATTTTTAAGGAATTGTTCTTTACTTGCCTAAGTTTATGCAACTATGATTTTGATTAAATAAGAACTATTTCAGCTCTTAACTAGCTTTCTTTCAAAAACTGTCGTATTGTTTCTATTGATCACTTTTTCTTTTTGTTTCTGTTTTTTTGGTTTAATTCCTATTATTGTGTGTCATTGAAACCGTTGGATGAAATTATACATGCTTTATAGTTTCAAGACTCTTGTTATTCACTTTTACTGATGACTGAAATATTGTTGGTAATTTGGTGATCAGAAGGCCAATTTAGGGGAACAGTTAGTCTGTGATCAGTTATGGCACCCGATCCCAAAATCACAAAGGCTTTTAAGGCAATGAAGCTTCTTGGAATTAGTGAAAACAAAGTGAAGCCGGTATTAAAAAGGCTTCTAAAACTGTATGATAAAAATTGGGAACTCATAGAAGGGGAAAATTACAGAGTTCTTGCTGATGCTATTTTCGAGGAAGAGGATAATAAGGTTCATTACTTGTCTTTGCCTATCACGGTTTGGTTTGTGATATTTATTCTTTTATCATCTTCTTTCCGTATTTTATTTTTAGTATGCTTGTCTAACCATGTTTCTTTTGTTTTTACCGAAATCAGGTGTCAGAACAGAAGAAACCTAAAATTGCTGTAAGTATGCAATCATTTATTTTTCGTTTCCAACCTGGCATTTTATGTTCTGGTTTTTTCCTTTTAGTCCCCTCACTTGTATTTACTTAATGCCTTCTGATTTGATTAACAGGAGGAAAAATTTGAGGAAGAATCTCTTGAACATGAGGAGCCTCTACGCCCCTTAAAGCGGTTGCGGAGAGGCGTGCAATCGGTAGTTCCACCTTCTCCCAGCAACTCCAGCCCTGGCTTTGGTGGAACTTTGTCAAGAAGGCCAAAAATGGATGGGGATGAATTACCTGCCTCTTCTTTCCAGCAGCAGTCACCAGAAAAGACAAAGTCTCCCAAATTCAATTTGGGGAATGTAAGGCTTGAAAACCATAGTTATAAAGGAAAGGAGCCTGTCTCACCTCAAGTTGCTTCCGCACAGAACAGAGCATCTCATGCATTGTGCATTAGAGGTCCAACCGTTGAACCTGGCATTGTTCCTTCGCCTAAGAAGGTGGTGCCCAGCACTCATGTATTTATCAGGCCCAAAGATGAGCCATTTACTGATGACATGTTCACAGATAACGCACCTCAGTATGAGGCTCCTATTGCGGTTATCCGTCCAGGTACAATTGATAGTAACATTTGCTATGTTAATGTTTTGAGTTAATGATTGGTTTGTGCTATTTGGTTGGGGGTGCCTGAGCCTGACGATGCAGTTCAATGAACTTATCCATGAAGTAAACAAAGGATTTTTGCATATAATGCTAGTTGATGCTAATATCACTATTTTTCTTTAAAACTGAGGTTATTGGTTTTTGTTTAATTTCATAGATTCATTAAGACAAGAAGATTCATTACCTGGGAACATTTCAGTGCAAGAGCCAGTTTCTCAGGAGCCTCCAGCTTCTCACCATGTACAGGGAGAATATAGGGGAGATGGTGCTCTAGCTTCGTTGGGTGAGGGAAGTTCCAACTGCAAACTTGCTGGCATGCCTTTTGAATTTCCTCCTAGTTTGGAGATTGCCTCATCATCCATGGGAGAGGTGAAGATTTCTCTTAGCTGCAACTCTACTTTTGGAAGGAAAAATTTCCATATGCCTAGTCTAGATGAGCTAAGAGAATTGTTGGAGGAGAGGTGTCTACGATCTTATAAAATCATAGACCCAAGTTTTTCAATCATGAACCTGATGAAAGATGTATGTAATTGCTTCGTGGAGCTGGCAACTAATACATCTCACGGATTGCAGGAGGAGTTAAGGAGCGTGACACCGAATTTGGATATATTGAAGAAATCTACTGCACAAGATGCTATTCTTGTTGGGGGTAGCAAAGAAAATATGTTCATCCCATCTGGTATTCGTAGTGGATCTGCACAGCTGGTTCCACCTCAAATTCCTAGGCCTCTAAAGTCATTAAATGGTGCGGATGATCACGTATCTACCAGTGAAGAAATCGTTGCAAATGGTGTTGTGGAAAGTGGTCTGGCAAAGGAATGGGGAGATCTTGAGTTTTCCAATTTGCATAGTTTGGTACCTGTTCCACTATGTCGACTAACTCCTGATGAGTTGAGGGCTATACATGATGTTAAAGACATAACCAAAGGGGAAGAGAGAGTTGCAATCCCTTGGGTTAATGAGATCAACAATGAGCGCCCACCATCCTTCTACTATATATCCCACAGTTTAGTTTTCCAAAATGCTTGTGTGAATTTCTCTCTTTCTCGGATTGGTGATGAAAGTTGTTGTTCTGCCTGTTTTGGAAACTGTTTACAGTCAGGCCTAACCTGTGCTTGTGCACATCAAAATGGGAGATTTGTATACACGCCTGAAGGCGTTCTTGAGGAAGAATTCTTAGAAGAGTGTATCTCAATGACTCGTGGTCCCCAGCAGCAACACCTCCTGAATTGCAGAGATTGCCCTCTTGAAAGATCAAAGAATGAAGGCATTTTGGAACCATGTAAGGGTCACTTGAAGAGGAACATTATCAAGGAATGTTGGAGCAAATGTGGTTGCTATAAACAATGCGGCAATCGTGTGGTGCAGCGTGGTATAAGTTGCAAATTTCAGGTTTAAACCTTTGCATCTGGTTGATGTTTGAGCTCTAATTTAATTTCTGGTGCTGGTAACTGTTAAAGTTCTCCAAAATGTTTATGGACTGTTCATTTTCAGGTTCTGTTTAAGTTGTTGCATCACTTTGTCATTGCCATCTAATCTTTTGTATTTTCCATTGTTAATTTTCTCTACAAATTAGCTGTTTTTTACATCTGATGGAAAAGGGTGGGGGCTGAGAACTCTAGAGAAGCTGCCAAAAGGTGCTTTTGTATGTGAATTTGTTGGAGAAATAGTAACCATCACGGAGTTCTATCAGAGGAATACTAGGAAACATAACTGCCCAGTTCTACTGGATGCATTTTGGGTCTCCCAAGGAGTTTCAAAGGACAAAGAAGCTCTCTGTTTGGATGCCACATGTTATGGAAATGCTGCCAGGTTCTTAAATCATAGGTAGAGCCCCATAGTGTATTCCTTTTGCCTTTTTCCTGCTTCCACTCTTGATAATCTTTCACAAGTCTTTATAAAATTGGCATTTGGGGTTTTTTTTTTTTTTTGACGAGTATAATCAATTGTTCTTGTTGTTACTTCAGTAGTTAGGTAAATGATATTTTCTTATGTATAATGAGAAACATCTTTTCACAAGTCTTTATAAAATTGGCATTTGGGGTTTTTTTTTTTTTTTGACGAGTATAATCAATTGTTCTTGTTGTTACTTCAGTAGTTAGGTAAATGATATTTTCTTATGTATAACGAGAAACATCTTTTCCTTTGTGTGTCTCTAGAATTCAATAACTTACTTAAACTATCTGTTTGCTTTTCATGTTTTTGGAGCTCACTAACTTTACTACCTAAACTATCAGTTTGCTTTTTCAGGGTGGCTGATTGTGAGGTGTGCTAAATTAGTTAAAAATATGTTACTGTCTATAGATTTCATTTGCTGAATAGGTAGCTAATGACTTTTTCCCTACATTTTCTTTTGTTTTTGGTGTCTTTAGATGCTTTGATGCAAACTTGATAGAGATTCCGGTTCAAATTGAGACTCCAGAGCACCACTATTATCACGTAAAAAAATCCTAAAATTCTTCTTTGGATCCAAATTTAATACTGGGTTTAATGTACATGGCAACTCTAATGGAGAATTTGCACCTTTCAGGTTGCTTTCTTTACAACAAGAGAAGTAGATGCATTTGAAGAGCTAACTTGGGTCAGTAGTTTTATTTTTAGTTGTACTTTCTCAGTTTTGGAATATAAACAGCTGGAGAAGATAGTTATATGATTTATGATGCCAATTTTTTTACTTTTAACTTGTGCTTCTTGTTGCTCTCACATTGAGAAATATTTGGTGTCATGTTATCATGTTTTGATGTTATTAAAATTTGAGAAGGAATATTGAATGATTTTTTTTAATGTGTTGATTTTGACTTGCTGCAAAGTTTTGCAGTTTGAAAGGGAGCAAACTGGTCTACTATTCTCTTATAATACATAAGCTTGTGCAGTACCGCCAAAAGAGACAAAAAGTAGAAGAAAGAACTAACATATAGTGCCAACTTTTTAGAAGCATCTTCTATTTATTATATCTTTGTTTGCATTTGTTGCAATGTTTATGAATTATGAGGAAGTTTATTTAGTTACTAGTCACTTATTTAATAAAGAAAAGTGAAGTCACTATGTATTGTAGAGCCTACAATGTACTTATTAAAGTTATTAATGAATATCTTCTAGTTCATTTGACACATCATGCAGAATTTTCTGCTTCTTCATGGTCTAATTTCGGGGTTATGGTAGGACATCCATGAGACAGCTTTCCTTGCTGCTAATATTTTACTCTCTATATAGTTCAGTGCTTCGCTACATATATTGATTTGCGTGCATGAGCTTACTCAGGTTATTCTGCCATATTCCCTTTTTAAATGTCTGAAAAAAAGGGCATCTCAATTTATTGAATGCCTACAACGTTTTACTCTGTATAAGTATCTTTTGAGTTTTTAATTTGTTCTATATTAAGACACCATGTGGTATGTGTCTGCAGGATTATGGTATTGACTTTGATGACCATGATCATCTAGTGAAATTCCGGTGTCGATGTGGCAGTAACTTCTGCAGGAACATGAAACGTTCAAGCAGTAAGGACCTTTAATTGTCTTTTTGTGGATGTGATTTTTGTTTTGCTAGATGCGCTTCTTGTCTGCATTGGTGTGCATCCGAACTGGCTACTCCGTGAATGTGGATGCTTTTTAGTTTTGGAAGGCTATGGGTTGCCTTATGTTTTGCAAATAAATTTTGATGCATTGGTGACCTTATTTTGAAAATAAGTTGCTCTTTCTTAGAAGCAGCTATTTTTCACCATCAAGCCTGGGTTTCTTTGAGACAAATTGCACTGATGGCATCTGGAAACACTTTTCTCCAAATGAAATGCAATGTAATGTGGATTTCGAAATAGATTTATGTAGGAAAGTGGCTAACCCATTTAATCTTTCATACCCTTGCAACATATTTATTGTTTCATACTCCTGAAACTGTTTGTATTTTGTTAAAATGTGGTGCTTTCCTAGCTTCTGCGAGAAACATTGGGATTGGGAATTCACACTGTTTGCTATTACTAGTTAGGCCTGTTCCTAGCATAATTGTTCTGCAATCTACATGTCTTTGGTGATGATGGGGGAAATACTGCTGGAGAGAGTGTGCAGAGTGCCTACACTGTTTGATGGTTCTAAAAAAATACCTCAAAATTAACTGTTTTCACATTGTAAATAATGTTTTAAATGTCTTTCACAGTCAGAAAGGAGTTAACCACTGTTGGGTACTTTCTCTCTGCTATTTGGCCAAAATTTTAAGTAGATTTTGGTGCCTGTAGCTGCTATTGGTCTAATGAGTGGCTTGCTTGAGATGCTATAAGTGTTGCATCACTGAAGTAAATTTCATTTTTGCATTATACCAGGCCTTTGAAGGAGGGCAACTATAAATTTTGGGGCTTGGGATGACATGTTGACTTGCTGTTGAATAGTTTTGGCAGCCCTCATGCTTTCATATATAGCTGTTTGGAAATTTTATTTTATTTATGTAATATTCATCACTGGGGACCTGCTTTATTCTGCTGAAATCTTGTTCCTTGGTTATGGCGTTGTGCATGTCTTTCTAATTTGAACTAGTGTTCATTCTTGAATTTTTAGAGCCTGAGAAGGTTGGTCTAAATAATATGATCTATTTAATGCACTTTTATGAGTCATTTCTTGTATTTTTCTCACCCCTCTTTGGCTACCTATGATCATGTACGTCATTGGTTCAGGATCCAAATCTGTATCCATTGCAAGATGATTTGACAAGAGGCATCTGATTGAAGAAATGATTGCTCTTATGTTACAAGCTTTTACAGTCTTGTTGAAGATAAAAGAGATCATGAAGTGCACTTTTGTTTGGCTCCCTCTTGATCCCCCTGAGTGATGGGGTCATTTTCTTTGGATGCTTTTTGTACAGGTTTCTGTATATACATGTGTAGAGACCATCAGTGGACCCAAATGTTAGCACAAATTTACAGATTAGTGTGTAGGCATTTACATGATCCATATTTAGCTGCTGAAACCTCTTGGGCTATTATATAACACAGGCTGCTGTTTTGTTTTTATTATTTTGTGGTCAACCAAAGTTCCTCAGCCATTTTGGACAAAAGTTGGTTCTTTTATATCTCTGAGTTTTGATCTATGATATGTCAATCGTGTAGCTGGGATTATCATCTATGATTAGGCTTGCCCTGGAG

>CsSDG17

CAAAACCACCATGAAAGGCTTAAGCTCCTGAACAGCTTCACCGTTAAAACCCAAAAGGAATCCCCATTTCCCATGCAATCTCCGCCTAACTGACTCAAAAGTTTCAATCCTTGAGCAAAATGGGTTCGATTGTTCCGTTTCAAGACCTCAATCTGATGCCGTCACCTAGCACCGCCGCCAGCACCGCCGCAGCGGCCACCCTGCCTCTGCTAACCCCCAAAATCGAGCCCAAAACCGAGCCATTCGACGAACCCGTCCCGACCCATCAGCTCGAACGGGGGCAAAACACCCCAGAATCGCTTCTTTCCGAATCTGCCCCTGGTTTCTTCTCTAATTCCGAAAATACCCCCGAATCTCAGCCCCCCGATCGGGACAACGTCTACTCCGAATTCTACCGCATTTCCGAGCTCTTCCGTACGGCTTTTGCTAAGAGGTTGCGTAAATATGGCGATGTTGATGTGTTGGACCCAGACTCTCGCGCTATCGTAACTGTGACTCATCAGGATGCCCAGTTATCAAACGCTGTAGTTCCGCGTACAAAACCGATGAAACGATCCGGTGAGCTCGTTCGCGTTACTGATTTGAGCGCCGAAGACGAGAGGTATTTCCGGGACGTTGTGAGAAGAACGAGAATGCTTTATGACTCCTTGCGCGTTTTTGCAGTGTACGAAGAGGAAAAGAGAAGAGGAATTGGACAGGGGAGGCGGGCGCGTGGCGATTTGACAGCCTCTTCGGTGATGAAAGAACGGCAACTGTGGCTGAATCGCGATAAAAGAATCGTGGGCTCGATCCCAGGTGTTCAAATCGGTGACGTGTTCTTTTTTAGAATGGAATTGCTTGTTGTTGGATTACATGGACATTCTCAAGCTGGGATTGATTACTTGCCCGGAAGCCAGAGCGCAAATGGGGAGCCAATTGCAACGAGTATTATTGTTTCTGGTGGTTATGAGGACGATGAAGATGCTGGGGATGTTCTTATATACACGGGACATGGTGGTCAAGATAAGTTGAGTAGGCAATGTGAACATCAGAAGCTTGAAGGAGGGAATCTGGCTATGGAAAGGAGTATGCATTATGGGATTGAGGTAAGAGTTATAAGAGGTTTTAGATATCAAGGTAGTGTTAGTAGTAAGGTTTATGTTTATGACGGTCTGTATAAGATTCATGATTGTTGGTTTGATGTGGGTAAGTCTGGTTTTGGTGTTTACAAGTATAAATTGTTGAGAATTGAGGGACAACCAGAAATGGGTAGTGCGATTTTGAGGTTTGCAGATAGTCTTAGGACTAAGCCGTTATCAGTGAGACCAAAAGGTTATCTCAGTCTTGATATATCAGGAAAGAAGGAGAATGTGCCAGTTCTTTTGTTTAATGATATTGATGGTGATTATGAACCATTGTACTATGAGTATCTTGTGAGGACTGTTTTTCCGCCCTTTGTGTTTACTCAAGGGAGTAATGGCGCTGGTTGTGATTGTGTTTCGGGTTGTACTGATCGTTGCTTTTGTGCTGTGAAGAATGGCGGTGAGTTTGCTTATGATCATAATGGGTATCTCTTGAGAGGAAAGCCAGTAATATTCGAATGTGGAGCATTTTGTCAGTGTCCACCAACTTGTCGTAATCGTGTTTCTCAAAGGGGGTTGAGAAATAGATTGGAAGTGTTTAGGTCGAGGGAGACAGGCTGGGGAGTAAGATCTTTGGACTTGATACATGCTGGTGCTTTTATATGTGAATATGCAGGAGTTGTTTTAACAATGGAGCAAGCCCAAATTTTCTCAATGAATGGTGATTCTCTGATTTATCCCAATCGGTTTTCTGCAAGATGGGGGGAGTGGGGGGATCTTTCTCAGGTATTTTCTGATTATATGCGCCCTTCTCATCCCTCAATACCACCTTTGGATTTTGCCATGGATGTTTCAAGAATGAGAAATGTCGCGTGTTATATAAGTCACAGTCCAACTCCTAATGTGATGGTACAATTTGTGTTGTATGATCACAATAATTTGATGTTCCCTCACCTTATGCTTTTTGCACTGGAGAATATCCCTCCTTTGAGGGAGCTTAGTATTGATTATGGGGTGGCTGATGAATGGTCAGGGAAGCTTGCTATTTGCAACTAAATACTTAGTGGACGTGTTGTAATTTTTTTATGTCACACAACTCGATGTTAAGACCATGAGGCGTTGAGGTAAACTCAAATTCCTATCCC

>CsSDG18

TTATATGCCCTTGATCTGAAAACAGGCCGTCGGTGAAAACTGAAAAGGGTTCGACCTCAACGTAGTGAGATGGAAGAAGAAGATGAAAGCCTTGAAAAGTTGCTGAAATGGGCAGCAGAAATGGGAATTACAGACTCAACAATTCAAAACCCTTCTCGCTCTCGCAACTGTTTGGGCCATTCTCTCACCGTCTCTCATTTCCCGGAGGCAGGTGGGTGAGTTACAGACTGAGCTCTGCTTATTCGAATGGAATATATATAATTTTTCATGTATGCATATAATTTTTCTTCTGTTACAGGAGAGGATTGGCTGCCGCTCGCGATCTTACAAAAGGAGAATTGATTCTCAGAGTTCCAAAAACAGCCTTATTTACTACTGAATGTTTATTGAAAAGTGATCAGAAACTATCTCTTGCTGTTAACAGACATCTCTTTCTCTCCCCTTCTCAGGTTTTTTTTTTTACTTTTATAGATATAATGTTTCCGCAATTGGGATTTTTCATTTTTTTTTTTGGTTTATTTTCTTATTACTAAGTGTAATTTTGCAGATATTGATTGTTTGTTTACTATATGAAGTGGGTAAAGGAAAGAGTTCACGGTGGCATGCATACTTAATGCTATTGCCTCGTTGTTATGAGATACTTGCAACCTTTGGTCCATTTGAGAAGCAAGCTCTTCAGGTTCAGTTTCTTTATTCTTTTGTTATTTGGAAGTAATTATGTTAGTTATTTGCTTCTCTGATAGTAAATTCTTCCTTAGGTGGATGATGCTATCTGGGCTGCTGAGAAGGCTGTGTCCAAAGCTGAATCCGAGTGGAAGCAAGCCATTAAACTAATGGAAGAACTTAAGCTTAAGCCTCAACTGCTTTCTTTTAAGGCGTGGCTTTGGGCTTCCGCAACTGTAAGAATATCTAGACGCAGCAAAGACAGACCCGGACAGTATGAGAGCCCTGCTGTACGGCCAAGGCGGATGTACGGTACAGCAGCAAACATACATAATAATAAAATGTTATATATTTTTGGAATTTTAAATATTCAGTTGCTTGATGAAATGATTATATAATTTATATGCCCTTGATCTGAAAACAGGCCGTCGGTGAAAACTGAAAAGGGTTCGACCTCAACGTAGTGAGATGGAAGAAGAAGATGAAAGCCTTGAAAAGTTGCTGAAATGGGCAGCAGAAATGGGAATTACAGACTCAACAATTCAAAACCCTTCTCGCTCTCGCAACTGTTTGGGCCATTCTCTCACCGTCTCTCATTTCCCGGAGGCAGGTGGGTGAGTTACAGACTGAGCTCTGCTTATTCGAATGGAATATATATAATTTTTCATGTATGCATATAATTTTTCTTCTGTTACAGGAGAGGATTGGCTGCCGCTCGCGATCTTACAAAAGGAGAATTGATTCTCAGAGTTCCAAAAACAGCCTTATTTACTACTGAATGTTTATTGAAAAGTGATCAGAAACTATCTCTTGCTGTTAACAGACATCTCTTTCTCTCCCCTTCTCAGGTTTTTTTTTTTACTTTTATAGATATAATGTTTCCGCAATTGGGATTTTTCATTTTTTTTTTTGGTTTATTTTCTTATTACTAAGTGTAATTTTGCAGATATTGATTGTTTGTTTACTATATGAAGTGGGTAAAGGAAAGAGTTCACGGTGGCATGCATACTTAATGCTATTGCCTCGTTGTTATGAGATACTTGCAACCTTTGGTCCATTTGAGAAGCAAGCTCTTCAGGTTCAGTTTCTTTATTCTTTTGTTATTTGGAAGTAATTATGTTAGTTATTTGCTTCTCTGATAGTAAATTCTTCCTTAGGTGGATGATGCTATCTGGGCTGCTGAGAAGGCTGTGTCCAAAGCTGAATCCGAGTGGAAGCAAGCCATTAAACTAATGGAAGAACTTAAGCTTAAGCCTCAACTGCTTTCTTTTAAGGCGTGGCTTTGGGCTTCCGCAACTGTAAGAATATCTAGACCTTTTTAGTAAATTCTAAGATTTATTTATTTAGTTACTTGGCATAGCTCTAGGAAAATCATATTCTTGTTGATTGAACCACATGTTTCTAAGAAATGGTTTAACCTCAAGATCCCAGAAATGATAGTTTACTTGGATTTGTGCACAAGTCAAATAATTGTATGTTCAATTTTTATCGTGTTTACAGGTTTCCTCTCGGACAATGCATATATCATGGGATGAAGCCGGGTGTTTATGTCCTGTGGGAGATTTATTTAACTATGCTGCACCTGGAGAGGGAGAAGAGTCAAATATTGGCATTGAAGATGTTGAAGGCTGGATGCCTGCCCCATGTTTGCCAAAGGGGGATACTACAGATGTCTTAGATTCTGAGAAATTCAATGATCATCTGCATAGGTTAACGGATGGTAGATTTGAGGAAGATGTTAATTCCTACTGTTTTTATGCTAGGAACAACTATAAGAGGGGAAAACAGGTACAACAAATTTCTTTTGACTAATTGGGCATGCGATAGCTTTCTGTTTTGTTTAAGAAGAAAAAGTTTGGAATCAAATCGCTAGCTTTTGATCCTATATCAAGTGTGGATGCTGACAGAATTGACATGTGATTTAATCATAAAAGCATGTTGTTCATGAATGGTCAAGTTTTTTCTACTCTACTTTATCCTGATATTGCCATTGATGTAAGCTTCCTAAGTACATAATTTTCGGGTTTACTTTTGATCAACTGCCAGTGCTGCATAATGATGTATTACTTCTCTTTGAAGAGCTTCATCTTCTCTCGGCTGTTGTCATATTTAAGAAGGTTTTAGATGTTGAAAGAACTTGTTGCTATAGCAACGGTAATAATAAGACAGTCTTGGAAGAAACTTGCTCTTTGAAATTTTGTAGCCTTTATAATATTCTCTTAGCATCATCTTAGTATCTTAAAAATGATTCAACAAAACCCCGTTAACCATTGTGGCTTTTTCTTGTGTAATTATATAGGCCTGAGTTATCCTAATTTGATTTGAATGTTACAAAGCAAATCTTAGGTTTGAAGGGAAAATTAGACATTTATCATGACATTGTCATCTGCTTGCAAAATTGTTTTTGTCAATATCCATGAAGCTAGATGAGATTAAATCAGTGTCTTGTTTGCTGCAGGTTCTATTAAGTTATGGAACTTACACAAATTTGGAGCTCCTTGAACACTACGGGTTTCTTTTAAATGAGAATCCAAATGACAAGGTTTTTATATCCTTGGAACCTGGCATGTATTCCGGTTGTTCGTGGCCCCGTGAATCACAATACGTTGACCAAGATGGTAAGCCATCGTTTGCTCTCCTCTCTGCTTTGCGGTTGTGGATGACACCAGCGAACCAACGGAGGTCAGTTGGCCACCTTGCTTATTCAGGATATCAACTTTCGGTGAACAACGAGATATCTGTCATGAAATGCTTATCAAACAACTGTTGTGTTATGCTGAACAGTTTGCCGACATCAAAAGAGGAAGATGCCTTATTGCTGTGCGCCATTGATAAAATCCAAGACATCAATACGGCAACAGAACTGAAAAAGGTTCTGTCAGATTTTGGAGGTGAGGTCTCTACATTCTTAGAAAACTACTATGTGCAATGCAGGCAGAGGGGTGCTAAGTTATCATTATCCCGGAAGACTAAATTGTCTATGCAGAGGTGGAAATTAGCAATTCAGTGGAGACTTAGATATAAGAAAACTCTTGCTGACTGCATTTCTTATTGTGATTATACAGTGAATTGTCTTCCTAATGATAACGTTCCGACTGGTGGAATTAAATAGTGCCTAATGTAACTCACATCTTTGTTACGAAAGTGTAGACTCACTATTCCTGATTTATTTTTGAGCCAAAATTCATCTCCAGGTAAAATTTTTGGATAGAATTGGATCGATAGCCAAAATTCATTTCATATTCTTGAAGCTGAAAATTCAATCCCGTTATTATTATTATCTAATGATTGAGTTGACAATTGAATATTGATGGATCAGATTTCTGGCTTGGATTCTCAAAAGAGAGAAAAAATCGCACAGCTTAATCAACTTCGATCATCTGGGGAAATAATCATCACATCCATCATAATCTCTTCAATTTCCTATAAAGTATTATATGCCATTGCTTCAAAACTATGTACTTCAAAAATTACAAGATATAAAATGATAAGAAAAAGAATGGATCAGAATTCTGGCTTGGATTCTCGAAAAGAGAAACAGATCGCTTAGTTTAATCAGCGTCGATCATTTGGGGAAATAATCCTCACTTCCATCCCTAAATCATATAATATTAAAGATCTTTTTTTTTTAATTTCTTAAAAAAATTCAAATAAACAAGGAAAAAATTCAAATACGAAAATGAAAATGGATCAGAAGACTGGCTTGGATTCTCAAAAAAAATAGAAAGGAAAATCGCACGGTTTAATCAGCGTCGTTCATCTGGGGAAATAATCATCACATCCATTTTAATTTCTTCAGAAAACTTAAAATTTACATAAAAGAAAATAAATTTATCCGGATCTGCAAAAAAGAAAACGAAAAAGAAAAAAATCTCTTAGCTTGCAGAAAAGGAGCAGATGTAGCGTCGTCTCATACACAGCACCCAATTAGACACAAAGCGCTGGTAATCAGTATTCGACGCTGTAAACGTAGTTCGGAAAAAAAAAAAAAAAAAAAAAAAGGGAATACAAAAGCTAAGAAATTATAGCATTGAGTTAAACGACAGCGGCGACCGGGAGAGGGTTCATTACTCACAAACGATGTCTTATGACTACTTATAGGAAGAAGAGTAGGGTTT

>CsSDG19

GGCAAAGCAGACGACAGTTGAGTGGCAAAGCGGATAACGTTGTCAAAATTTGTAAAACCTCTGCACTCCAAAACCTTCAGTATGAGTACAAGTTGAGCTTGTAATTGTGAAGTTTGAATTCTTTCTTTGCTACCCCTTAAATGGCCATTTCTGTTCCTCTTCATCAGCCCACTTATTCTTTCTTCTCCAATCCTCAGGTCCGTTCTATTTTCTTTTTCTGTTTTTGTATGTCTTCTTTCTCTAATTTTAGCTAATATAGTATTGACTTTCTTTCTTTTTAATGCAAAAGGAACAGTGGAAATGGTGCGCAAAACCCAGTTACTCATTCAGCAATAACAGTCAAAACAATATCAGACCCATCAAAGCTTCTGTTGAGACTCCTCCATTTCCTCTCTTTCAGAATCCCAAACTGGAGGAAACACCAGCCGACGGGGTAATAATTTTTAGAGCTTTTAATGGGCATGTTAGAATGTTACTTCATTTGAGCTCAGCATAAGCTTTAATAATTCAAAAGTCAAGCTGGAGATAAGTTTGCCTTTGTTTCTTTTCATTTTCCTTTTAAATGGAATGGAGTTGTTGACTGTAATGTTTGCATTTCTTCATTTGTATATAGTTGGAGCCAGCAGACCCTGATTTCTACAAGATAGGTTATGTCCGGAGTATGAGAGCATACGGGGTTGAATTTAAGGAAGGTCCTGATGGATTTGGAGTTTTTGCTTCCAAAGATATTGAACCACGCCGTCGTGCTCGGGTGAGCTCAGTTGATTATTTCTTATTGGATGACCATATGATTGATGGCATAATAGCTATGCTTTTGCAGTATCCATTGTAGTACTTTGGATTGGCTGTGGTTTTGGTGTTTCCTGTTGTTTGCATGACCTTAATTGACCTCTAATTTTGATTCCTATATCTATATTTACTTACAGTGTGGGGGTTCTGTTCTTACTGTGCAGTTAGTCATGCAAATACCTCTAGAGTTGATGCTAACCATAAGACAGAAACTCCCATGGATGTTCTTCCCCGATATAGTGCCATTAGGCCATCCAATATTTGATATAATTAACTCTACCGACCCTGAGGTAACTTAGTTTCTTCTAAATATCAGAGATTAGCTCTTGATCTTATTTTCAATTATTGAATCTTCACCATTGCATTGGCATATCTTTTTCTTTTTTTTTTAATTTCTCTCTTTTTGTGTATTCCATTACTATATATTCTGTTGATAATGGAATCTTAAGAGCTGAGTTCAATATCTTTTTTTATATCCAAATGTGTAATTATGATAGATTTCTCTATTGGCAGACAGATTGGGACCTTAGATTGGCATGCCTTCTTTTATATGCATTTGATCAGGACGATAATTTTTGGCAATTATATGGTGACTTCTTACCTAATGCAGATGAGTGTACTAGCTTGCTTCTTGCGACAGAGGTGCGTGATCCACAGAATGGGAAACCATATATGCATATAGATTGTGACTTGTGTTCTTCCAGTGATATTACTGATTATTTCATGCAATTAACTCTAAAGTTTGTAATCTAGACATATATGGGACATTCCTTGTATTATATACTGACATTTTCGTAATGCATATGCGCTTATGTCTTCCGCTTAGAGTCCTACCTTGATCAAGGATGAGGGAAATGCCTTGGGATGAACCTCAAGGCTCATGTGTCAAATATTTATGTGCACATAACTTGTCTTCTAATGTCTCAGGCCCATGAAGATAATGATCTCATATAAGATTCTACTAGCCAGCTGTTGATTGAAAGGTTCTTAATGTTTAGTATCTCCATCTCTCACAAGACATCCAAATGTTAGCCGACTGATTTCTCCATTTTTATTTTTATTTTATTAAAAAAAATGAAAGTATGAGTCTTGAGGATTTAACATAATGAAAACATTTATTTGTTTGCCTTATGACATTTCATTAATTCATTATGCTTTCTTTGCTAAATTGCAGGAGAGGAAAACATGACTGAATGACTACATATACAAACATATTAACTTTTTGAAATTAGTTCTTTAACTTTGACATGCTTATGGGCAGATTATATGTTTGAAATAAATAATCTATCTTCAAGTTGAATGAGATCATTGTAAATTCTTAAGGTGGTTTTGGGGTTAGTGGACATCTCGTTCACCTTTTGTAGATCTTGTTTTGTATATACAATCTACTTAACTTTTTTCTCCAAAAGAAGAAGAACAAGAAGTTGAATGAGATCATCGGCTGTGGAAAACCCAAGCCTTACCACTTAAAGTTATTTTCTGTGTCATTCTAACATTAATGTTATTTATTTCTTATCTGAACTTGCTTTCATTCTAAGTGTCTGTTAGATAATCCTTTGTCACTAATCAACTTGTTATCTTTACAACTCTTTTCTCTACAGCTCTGTGAAGCAAAATTAACATTTTTCCTCTTCCTCATTGTTCTATGTGTCTTGTAGGAGGACCTTATGGAGCTGCAGGACCCAAATCTTGCTTCAACTATGAGAGAACAACAAAAAAGAGCCCGAGAATTCTGGGAAAAAAACTGGGTACCAAGAAATTAGATGTTGCACAGTATAAAATGTTCTTATTATCTCACAACTTTTATCTACCCTTTAATTGATGGAGCTGCTTGTCTTTTTTAATGTCGCTCAGCACTCAGGTGTTCCCCTCAAGATAAAGCGTCTTGCTCATGATCCTGAAAGATTTATTTGGGCAGTAAGTATTGCCCAGTCACGGTGCATTAACATGCAAGTGAGGATTGGTGCTTTGGTACAAGATGCAAACATGCTAATCCCTTATGCTGGTAAACATTTGTCGTCACATTCTTCTACTTATATTAGTTGATAGCTTGTCATCATATTTATGATATAATGTAGAGAAATGCATACAGGATTGCTGTATATCATCTGATGTTTAACTTCATTAAAAAAATTCTCACTTGTATGTTGTACTTGACAGGAAGTATTCTATGTAAGCTTTAATTAACAAGAAGAACATGAAGTTTTTCATTCATAAATGTTAATGGCAAGTAGATTGAAAACAATTGCATAGATGTTATGTGATGACACCTTCTCTTTTTTTATTTTTATCTGTTAATCTTTGGTTGTTCTCTCTTTTCTAGATTCCAACCATTGCCATGTTCTTTATATGTCTATCCTATATTTGTTCTCAATTTATTCACCATTTTGTTTACTTATTAGTTGTCTTGGATGTAATGTTATTTCATTCCTATTCATCTGCTTTTCATTAGCCACATTTGCTTAATTGTAGTTGCTTTGGCTATTTATTAATATGTTCCTGTTTTGTAACTTCAATCTTATTTCGCAGACATGCTAAACCATTCGTTTCAGCCAAATTGCTTTTTCCATTGGCGTTTTAAGGATCGCATGCTTGAGGTGATGGTAAATGCTGGACAACACGTTAGAAGGGGAGAAGAGGTAATATATTCTAGAGTTTGATGTTTACATATGCCTTAAATTAGTTTGAATATATATATCTATATGTAGAAATGTGCACAGAGCACTGATGGCAGAGACAAAACTGAGTGAAACATTGTTTGTCGAGCAAGTGTTTAGTGGATGTAGAAATGATTTAAGATATTTAGCAATATGCTAAATTATGATATATTCTGATGCATGATGGCCAAAACCAGTTTATTTTAGTGAACTTTTTCAGTTATAGTCGAAACAGAATAGCAATAGAAATTGGAGCACTGCAGTTATAAAACCCCATGTCAACCATTATAGGACCAATATGTTGCTTTCTAACTGAAAACCAAACTGCATGTACATTTTTCCAACTACAGCAAACACTAATCTGTTCATATCATCAAGGGATCTGCCTTATGTTGAAAATTGGTAGTCATTTAAGCTTTGTCCTGTGAACAATTCTTTCTTCATGACTTCAAAATAAGTCTTTTAGTGACACCATTGCATATGAGAAGCCTAGAGGATTGGCTTCCAGCATTCACAAGCTTTCTAAGTTCTATGATGAGTGTTATTGCCCCCATTGTTGGCCTAAATCACTTGAGTACTATCAGTAAAGTAGTTGCAAAAGCTTCCACAGTTCCTGGGTCTATACTGCATCCCACGAAATTAATGTTGACTATTTAGAGTTTGTCCCATTCTGATTCTCTTATCTTATTTTATTACTCTTTTTCTTGTTCTCAAATAACCCTTTTTACATAAATTGACATATTGCATTAGTTCCTCATTGATCTTGTAAAATTTTTACAGATGACTGTCAATTATATGCATGGGCAGATGAACGACATGCTTATGCAAAGATATGGTTTTTCGTCACCAGTGGTAATTTTCTCTCAATTTGCTCATCAGTAACGGATTCCTGTTTTAGATTATGTGGTTCTGGTTGCACTAAGAGAGTAATGTATTGTTTATTCTCTCAAAAGACTCTTCTAAAAGTATTTAATCTTGATTACTTTTAGTGCTTTTTAAATTTGATTTCTTAAATGATCACTTCTCTTCAATATTAACACACACGTATGTCTCGCTTGTTAAACTGCAGAATCCTTGGAATGTAATTCAGTTTTCTGGCGATGCCCGCATACATTTGGATTCTTTCTTGTCAGTTTTCAATATATCCGGTCTTCCTGAAGAATATTACCATAATAGTACGTGTTCTGCATAGTTCTTTGATTTCCTTTTCACAAAAAGCTGTATATTACTATCTAGCAATTCGATTCTAGCATCACTGTAAGATCTGCATAAAATTGATAATAGGTAAAATATCAAGTGACGAAGAAAGTTTCATCGATGGAGCAGTCATAGCAGCAGCAAGAACACTGCCCACTTGGTCAGACGGGGATGTGCCCCTGGTTCCAAGCATAGAAAGAAAAGCTGTGAAGGAATTACAGGAAGAATGCCGACAGATGCTAGCAGAGTTCCCTACCACTTCTAAACAAGATCAGAAAATGCTGGGTAAAGTTGCTATTCATATTTAACTCCTTCCTAGACTTTAACCGCTTGATACTCTTATTATAAGCATATTTACTTTTGTTCCAATAGAAATATAATTGTTATTCAGTGCTTATGTTTATTTTTTTTCATCTTTTTACCAACTCACAGATTCTATGAAAGAACCTAGGAGGACCCTTGAAGCCGCAATCAAGTATGTAGTTTAGTCCTTTTGAGAACTTTTTTGCTTCTAGTTGAACTGCAAATCAAAAGCAATTTTTAGTGATTCGTTTCTTGAAAATCTGAAATAATGCAGGTATAGATTGCACCGGAAATTATTCATCGATAAGGTTACCAAGGCATTGGACATTTATCAAGATCGGATACTGTTTTAACCATGATGATCTTGAGTTTGCAGGATCTGAAACAGTTTTGTTACATAGCTAATGTAATAAAACGATCATTGTGTTAAGCTATTTCCTGTTTGTGAAGACCTCGGTTGTAATTTAGTTGACAGAATTAGTTTTCTCTTTTTTTTTTTTAATCTCTTTTATGTGTCTGTCTCTTTCTCCTAATTTTTTTTTGTTAGTCATATAAATTATAAGAATAATGCTCGGCACACAATTTGTATCTATGTAATAACTGACCCGACATTGGATATTCAATGCTGAACAAGATAATAGCTGTTGGCTAAATTGCTTAATACAAATAGTTTTTGAGAGGGCTATTTTTATAGTTATTTTTTGATTTTTTTTTTTTAAATTCAGC

>CsSDG20

ATGAGAGAAGATGAACAACAACAGCTTCAAACCGCAGAAGAGCTGATGCAACAGCTGAGATCTAAAGCCACTGAGCTACTCTTGAGAGAAGAATGGAAAGAATCCGTACAAGTCTACACTCAGTTCATCGGTCTTTGCCAAAGCCAAATCACAGAAACGAAGCAGGAAGCAAGCCAACTATCCAAGCTCAAAAAATCCCTCTGCTTAGCCCTGTCAAACAGAGCCGAGGCACGATCAAGGCTTCGAGATTTCGATGATGCATTGCGTGACTGTGAACAAGCACTGAAAATAGAGAGTTCCCATTTCAAGGCTCTTCTTTGCAAAGGTAAAGTTCTGCTTAGTTTGAATAGATATTCAATGGCTTTGGATTGCTTCAAGGAAACTTTAGTTGATGCTCAGGCTAGTGGAAGTCTTGAAACTGTTAATGGGTTTTTGGAGAAAAGCAAGAAACTTGAGTACCAATCAAGAACAGGTGCTTTGGATCTTTCGGATTGGATCCTTAATGGGCTACGTGGCAAGTGTCCAGAATTGGCTGAGTACATTGGTGCTGTGCAGATTAGTAAGTCTGAGATCAGTGGACGAGGCTTATTTGCAACAAAGAATGTTGAGGCTGGGACTTTATTTCTAGTCACAAAAGCAATTGCTACAGAGAGAGGTATATTGTCAGGTGAAAATTCAAATGAGAATGAGCAATTGGTTATGTGGAAGAATTTCATTGATAAAGTTATGGAATCGATTTCAAAATGTCAAAGGACTCGCCACTTGATTAGTATATTATCTAGTGGTGACAATGAGGATGAGGTTGAGGTTCCTGATGTAAGTGCTTTTAGGCCCGAAGCAGAAGAGCGTAGGAGCTCCAATGAGAAGCTTGATATGGGTAAGATTTTGAGTATCTTGGACGTGAATTCCCTTGTTGAGGATGCAATTTCAGCAAAAGTTTTGGGGAAGAATAAAGGTCTTTATGGTCTTGGGCTATGGGCACTTGCTTCATTCATCAACCATTCTTGTAGTCCTAATGCAAGGCGTGTTCATGTAGGAGATTATATCATAGTTCATGCTTCAAGGGATGTGAAGGCTGGTGAGGAGATCACATTTGCCTACTTTGACATGCTTCTGCCATTGGAGAAGCGCAAGGAAATGTCAAAAACGTGGGGGTTTCATTGCAAGTGCAAGAGGTGCAAGTTTGAGGAAGGAATGAGTTCAAAGCAAGAGTTGAGTGAGATAGAGATAGGACTTGAAAGAGGCATAGATGCAGGCAATGCAGTTTTTAGGTTAGAGGAAAACATGAAAAGATGGATTGTGAGGGGAAAAGAGAAGGGCTATTTGAGGGCATCGATTTGGAGTGCATATGCGGAGACTTATGGTTCAGAGAGGCTGATGAAGAGGTGGGGAAAGCGAATTCCTGCAGCAGAAGCTGTGGTGGATAGTGTTGTGGAAGCTGTGGGGTGCGATGAGAGAATGCTCAAGGTTTTGATAGAAGGATTGAAGGGAAGTAGTTGTGGGATGCCGGAGATGGAGAGACCAATTAAGTTAGGAAAAGGTCTCTATGGAAAATTGGTCAAAAAACAAGCTTTGAAATCTCTACTTGAGATCTAA

>CsSDG21

AGAGCATAGCCAAGAATGGTGTCCAGAGCAAGCGACTCCTCCTCTAAATCCAGAGTTAGATTTTCTTTTTCAGATCTTAATTTTTTAATTACTTATCTATTTTGGTTATTTTGTGAATTAAATTATGAGTACTGATCATCGGAATCGAAATATTATCACGATAATTAAGGACTTTTACTTGATTCTACTGAATTGTTTACAATTATTTATCTGTAGTTATTAAAGAATTGATATTGGAGACCAGCTACGATTAGCCGCTGGGAAACTGTACTTAAAATAAAATTTCATGTTAACTTTTTCATTTCCTAAGACATCTAATCAACCAAACAGACCATAAACTTGAAGAAAAAATTCTAGAGACGACGTCAAAATTCTTGATTATAAAAAATATACTATAATTCGATCAATGTTTTAGATTTTTGATAGGCTTGTTTTGTGAATTGGCAGAAGTCCTATAGCGAGCAGTCTAATGATGGTCTTGGAAATTTGACATATAAGCTAAATCAGCTCAAGAAGCAAGTTCAAGCGGAGAGAGTTGTTTCAGTAAAAGTAAGTCACTTGTGCATTTACTTTAAGGTTCTTTAAAATTTTCTCTTTTCGCTGCTGACACTTAGTAACTTTTTTCATGTAATTTTAGATTAAAGTCACTCTTGGTTTATAGAGCTTGTGTTTTGCTTAGATGAATTTCTTCATTGCTGCAACAATTTCACATAGATCGTTCTATTTTCTCTTTCCTAATATTGTTCGCCAATAAATATCATTTTGAAGAAAATACCATATGTTTATTATATTTCTGTCTTTTATGTTGGCTTTTAGTTGTAGATGATGTTCCTGAAGTGATTTTAAGTTAAATTCATTTGTGAAAAATGAAAAAAAAAATCAATTGGAATGTGGAATGAGCACGCTTTAGATGCTAATTAATATGCTAGTAACTTCTGTATGTATCATGTACAGGATAAAATTGAGAAGAATAGAAAGAAAATTGAAAATGATATTTCTCAGCTTTTGTCTACAACATCAAGAAAGAGTGTTATATTTGCGATGGATAATGGATTTGGTAACATGCCTCTCTGCAAATACAGTGGATTTCCTCAAGGACTGGGAGATAGAGACTATGTCAACAGTCATGAGGTTGTGCTCTCAACAAGTAGCAAGCTTTCACATGTTCAGAAGATACCGCCGTATACCACTTGGATCTTCTTGGACAAGTATGATAAATTTTCCATATATCTTCTCTCCACTTCACCGTACAATAATTTGTATCAGTTGTGCTTTGGTTTTCCCATGGTGAAAGAAAGTATTAGTAAAGTTTACGGTAAATTTGACAACCTCAATGCTTCTTTCAAAATTCACTTCATGAATGTTGATTGGTACTGATGACATTAGTCGTTAGTCATTAATTCCACGAAGATGAAAGCTGAAAAATATGTTTCTTTGCTCTTACTTATTCTGATTATAATTATTGAAATTTATTTCATCCAATCTGCCTGGTTGAATTATTTATGAACCATAGGATAGAGAGGCAATTGGAGGGAGGGGTTGTGGAGGATGTGTTTTTGTCTATGTGGGTTTTTGAAATGTAATAAAATGCCATGAGTTTCGGTAGGTCTTTGCATCTGTACTGCTTGTTTGCTTGATCACCCAGTAAAATGGTGAGCTGGTTTTTTCTTGTGGAGCATTGTTTAATGTAGAAACATAGGTTTATATATTCATAAAATGCAAAACATGAATCATTTTGTACTTTACTTTATTAACTGTCTTTTAATGTTACGATTTTATCGTTGCTCTTCTTATCTCAAATATGCCTTACACAGTACAGAAATCAGAGAATGGCTGAAGACCAGTCAGTGGTTGGGAGAAGACGTATATATTATGATCAACATGGAAGCGAAGCACTAGTCTGCAGTGACAGTGAGGAAGACATTATAGAACCTGAGGAGGAGAAACATGAATTCTCTGATGGTGAAGATCGTATTCTATGGTATGTTAAAAGAAGATGCAAGAACTGCAAAATTAGTGTCTCTTCAGAAATGCCATACAATTTTTTGGTGATCCTGCTGATAAGTACATTAGTGTTGGATTTTCCTATTTAAAGTTTGACTTTGATTGGAATTCGGGTTTTATGATGGTATTTAAAGGTTGAATATAATTTGCATCTTATTATTCTTTGATTCAAATTCAGGTTTTATGATGCTATTTAAAGGTTGAATATGATTTGCATCTTATTATTTTCTTAGCCTGTATAATATATGGTGCCAAATGAATATCACCAAGAGTGAACAAGCAACAATTGTTGGTAAAAATTAAAAATCCATTAGTGGAGAGATCATACTGTGAAAATGACTGTCAGAAATTTCTGAAGGAAAAAACCTGCTGTCAGTTTCTATTCAAACTGAAGACTGATCCTTTTGAAGTTACTATAGCACTTCGCTTATTGCTTTCTGAACTTATATATGGCCCTGCTGTTTGAAGTTTTCCAAAGCGAAAGAAGCATTCTGATCATGAAAGATCCTACTTGATTATTTATTATGCTTCACCATGAATAATTACCAGTTCTACTAATGTTGGCAGGACGGTATTTGAGGAGCATGGGCTAGGTGAGGAAGTAATAAATGCTGTGAGCCAGTTCATTGGAATAGCCACCTCGGAAGTACAGGTATTTTCTGTTTGATCTTGATGTTTGGAGTATGCTTGGCGTTATTTTATATGAAATATCATCTGTATTCATTTACTATTCTATTGATGTTGTTGAAACATCTTTTATATTTCTGGGAATGTAATCTGTTTGGTTTGCAGGATAGATACAGCACACTAAAGGAGAAATATGATGGAAAAAACTTGAAGGAATTTGAGGATGCCGGACATGAAAGGGGGATTGCTCTGGAGAAGAGCCTAAGTGCTGCTTTGGATTCTTTTGATAACCTCTTTTGTCGCCGTTGCTTGGTATGATAGTAATTGATTCAACCATTTGAATTGATTGAACAACTTTGCTTTTCTAACTGTATCTATGAACAGTTATTTGACTGCCGTCTGCATGGATGTTCCCAAACTTTGATTAATCCTGTAAGCCGACACATTTTTCATCTTTCTACAACTTAATCGGGTTACATTTTGTTAATTTATATGATCAAGTTCTTCTACCTCACAGAGTGAGAAGCAACCTTATTGGTCTGAATATGAAGATGATAGGAAACCTTGCAGCAATCATTGTTACCTTCAGGCATTCTCTCTCTCTCTCTCTCTCTCTGCGTGTGTGTGTGTGTGTGTGTGTGTAAGTATGTTCAAATAGCATGTGTGTAAGTGAGTATGTACAAATAGCATCTTCTTGTTTTGAGATGGGATTGTGCTTGTTTATACTCTCAGGATTTGCTAGAAGGTTGTATAATCCTCTTTTTGTTTATGGTTATCGAAATATATGATTCATCATTTTGATTCTAATTGCATGGAAAAGTTCTGAAGTCCAATTTCCATATGAATGCTTTGCAGTCAAGAGCTGTTCAGGATACAGTAGAGGGGTCAGCTGGTAATATCTCTAGTATAATAACAAATACAGAAGGGACTTTACTGCACTGCAATGCTGAAGTGCCAGGTGCTCATAGTGACATCATGGCAGGTGAAAGATGCAATTCCAAAAGGGTGTTACCTGTTACATCAGAAGCTGTGGACAGCTCAGAAGTTGCTATTGGTAATGAAAATACAGATACTTCAATGCAAAGTTTGGGAAAGCGCAAAGCTTTAGAGCTAAATGACTCAGTAAAAGTTTTCGATGAAATTGAGGAATCTTTAAATAAGAAACAAAAGAAACTGTTGCCTTTGGATGTACTTACAGCATCAAGTGATGGTATACCACGTCCAGACACCAAAAGTGGGCACCATGTTGGTGCAATCAATGACAATGAACTTCAAATGACCTCCAAAAATACAATAAAGAAATCTGTTAGTGCTAAAGTTGTTTCCCATAATAATATTGAGCACAATATAATGGATGGAGCCAAAGATGTCAATAAAGAGCCTGAGATGAAGCAGTCATTTTCCAAAGGAGAACTGCCTGAAGGAGTCTTGTGCAGCTCTGAGTGGAAACCTATTGAGAAAGAACTATACTTGAAAGGAGTGGAGATATTTGGGAGAAACAGGTCTATGGGATTCCTTTGCATCTGAACTTTTACTCAGTCTATAAGTATGATATGCATAAAGATTCTGAAGATATAGAAAAAGGATCCATATAGTTTATATGTTCATTGGCATTCTCCATTCAACTTAGTTGCAAAACTGACAGTTTCTGCTTTTTCTTTTCAAATGCATGTGTTGGACTTGATAAATTTTGATGTTTGTTGTTGCTTTTCTTCCTTCTGTTTCTGCTTCAAAGACTATATCTTTCTTTTTTCACTTTGTGCTTAATCCTCTGCAGTTGCCTTATAGCCAGAAATTTACTTTCTGGTCTGAAGACTTGCATGGAAGTTTCTACTTACATGCGTGACAGTAGTTCTTCAATGCCCCATAAATCTGTTGCACCAAGCTCCTTTTTGGAAGAAACTGTGAAAGTTGATACAGATTATGCAGTATGTAAATTTATAGTTTTATATTGTTTTTAGTGTTTTAAGGAAAACTAGTTGAATATTATTTTTATCTCGGACATAGCCTCGAAGTGAATCTAGTACTATTCTTTTCATCTTATTGTTGTTTAAGGAGCAAGAGATGCCTGCAAGACCACGGTTGCTTCGTAGAAGAGGAAGAGCACGGAAACTTAAATATTCTTGGAAGTCCGCTGGCCATCCATCAATTTGGAAAAGAATTGCAGATGGTAAAAATCAATCTTGTAAGCAGTATACCCCATGTGGGTGCCAGTCTATGTGTGGAAAGCAGTGCCCTTGTCTGCATAATGGAACATGCTGTGAAAAATACTGCGGGTAGGTCGAATTGTTATTTAATAGGTATTGAGGGAGGTCTTAGGGAATCCATGGGTGATTTTTCTCCATCCTCTTCAATAGAGATAGGGCTAGGATCTATCATGCTTCTTATGACTTATGAGTAAAGATCATCCTGGTGGAATAGAGGTTCTAGAATGGACTATAGCTCCGCTTGGGAGTGATGCCAAGAGGAAAATAGGGATCGATTCCTCCTTTTTTACAGCTGGGCTGCTGCTTGGCAGTGCTTCCAAATGGAGCTTAAGTTGGATTAAGGCTGATTCTGTGGTCAATAAACCCTTGTCCTTGTTTTATTTAATTGCAAGTATAACTTTACATAACTGTTTTTATTACAATTACATAAATTTACACATCTCTTATGATTACAATTCAACTCCACTTGTGGATCATTTCCTGAATTCCAACTTATATAATTTGGTAGATCAAAGATAATTATTTTTGTAAGATTTTGTTCATGCAATAGCTATTCCTTCTTAAGGTGCTCGAAGAGTTGCAAAAACCGGTTCAGGGGATGCCACTGTGCAAAGAGTCAATGTCGAAGCAGGCAATGTCCATGCTTTGCTGCTGGACGTGAATGTGACCCAGATGTTTGCCGGAATTGCTGGGTTAGGTAAGGAGCTTTATATGCTAATATCTTATACCTATCTAGGTGGGTTTACGATTTCGTTGGGCAAGTTAAATTTGGTTTGCTTATAAGTACTTTATACTTCCATTTCCATCTTAGATTAGCTTTCATCATCCATTAACTACACATTTCAGATGTTTTGAAGGTTATTATAAATGTTTACATTGTTTATAGTAATATAATTGTATTTTCCAGCTCTTAGTTGTAGTTGATACCCAAGTTTGCGTGGACTCTCAGACTGCTGAAACGATTATTTAGTCCTGCTACAAGGTTTTTTTGGGCTCTACTCTTGAAATAGAAGTGGTGACCTGTAATAGAAACAATTTTGAAAACAATATAGAGGTTTCTAGAAATTTAGATATCTTGCATTTATCTTTCTGATGGGCACTGGCTTACTACCTTTCAAGAGTTCTATGGGGGAACAAAAGACTTTCCTGAAATTCTTTTCTTCTTTCTTTTATGTTACAGTATTTTGAGCTCTCAGGTCACTAGAATTATTCAAGACCTGAAGTGGTTCTATTTCCAATTTGAACTGTTTTGGAGATTGTAAAATATTACAGTTTAGTCGATACTTCTGTCTGGGTAAGTTATTATTTTTTCATTGCAATCTCAGCATCTCTGGTTTATTTTATACTTTAATGCAGTTGTGGGGATGGTTCATTGGGTGAGCCACCCAAACGAGGAGATGGTCAATGTGGTAACATGAGGCTGCTTCTAAGGCAGCAACAGAGGGTGAGTAATTCAGAATGTTTCAACTCTCAAAAGCTGTCGAAAGTTGAAAACATTTTTAATTAATAAGTTGTATCATACTTCCTTTTATCCTCCTTTTCAGATACTCTTGGCAAAGTCTGATGTTGCTGGATGGGGAGCCTTTTTAAAGGTAGGGTAACGAATCGTCTCATGTTTCTCTGCAATTACTTGTTAGTGCCGGTACTGAACTTGATGATGTGATTTCAGAATTCTGTCAGCAAGAATGATTATCTTGGAGAATATACTGGGGAATTGATTTCCCACCGAGAAGCAGACAAGCGTGGGAAAATTTATGATCGTGCAAATTCGTCTTTTCTATTTGACTTGAATGATCAGGCAAGTGACATCTGGTGCATACAAAATGGACTATGAGCTGAAGAATATATAATATATTGTGATGTACTTTAGCATGATAATGTTTTCACTTCCACAGTGATGATCTCATAAATAAATTCCATGCTTTCTGTTTACTATCTTCATAATCACCAGCATCTCTTAACATTGCACTGTATGGTCTCTTAAAGCTTCCACCTCTTCTTTGCAGTATGTCCTTGATGCTTATCGCAAAGGAGACAAGCTGAAATTTGCAAACCACTCTTCAAATCCTAACTGTTTTGCCAAGGTATAAGCATTTTGCTAGTTTCCTTTGACTCAATTCTCATTTGTTTAAGCTAATGTGTTGGCTTGTGCAGGTAATGCTGGTAGCTGGAGATCATCGGGTGGGAATATTTGCCAAGGAGCACATTGAAGCTAGTGAGGAGCTTTTTTATGACTACCGTTACGGACCAGACCAAGCACCAGCATGGGCTCGGAAACCTGAGGGTTCCAAGAGAGAAGATTCATCAGTGTCTCAAGGCAGAGCTAAGAAACATCAATCTCATTGACGTACAGACTACATGGTTTTTCAAATACATCTGGGTCATTTTCCAACAAAATCAGCAGTGTTTTTGTCCATATAACCTATTAAAGGAAAGGAAGCATGTATATGTCAGCATCATTTATCTCTGTATATATGTGCCCGAGTGTGCCTTCAGAGGTTTTGAAGCTCTAGCAATTATAAGATAAAGATTGGGCAATAAGGTCGAAACATTATTATAGTTTGACAATAAGCTAAAAATTTTTCCAGCAAGCCTCTACAACTGGGTAGGCGACGAATAAAGTTGGGCAATATCGTTGTGAGTGGCGTGTAGCATAATTACGGTTGATCCATTTATTTTAGGGAGAATGCTGCAATTCTGATTTATATTATCAGGCCGAGACCTGTGGCTTCCCCGCATATGCCTTAAAGATTTGTTGTTTTCCAACAGTATTCAAGAAACATTTGTTGAGCTCATAAGTTCATATACATCGCCATTTTAGCGGTGAATTATCGTTGAGTCT

>CsSDG22

GGTTCACACGCACTCCCTCACGCGTTTCACAACAATCAATATTTAATTAAACCAAATTATTACAAGAAAAAACAAAAAAGCCCAAATAAAATTTTGAAAAAAAAAGAAAATCAAACCAAAACAAATTAAAATAGAAAGAAAATTTGAGGGCGAGGTAGATGTTAGCGTTAGGGTTTCAAGCCGACGCGCCAAATCCTCTCAGAAACCCTACATTCTTGTTCGGCATTGTATCTGAATGTATCGGATCTTGATCCGGACCCGCATTGCCGATTTCGGTTCGAACTGCCATGGCCTCGAAGGCATCGCCTTCTGCCTCTCCTAACAGATCGGAGCCTCTCAAAAGCTCTTCGGTATTTGATTTTGAATTTTATTTTTTCTGGAAAGTCTTTTCAGAAAATGCATGTCAAAGTCGTCGATAATGCTCATCTGTAAACCTTTTTCAGAATTTCATGTCAAAGTCGTCGATAATGCTCATCTGTTAACCTTTTTCAGTATTTTCTTCTTTCGTTACGTGTTGTAGCTGATATTCGAAAGACATATATCTGCATTCGTTCTTAGTGGATGATTTGACCTAATTATGATTTTTTTTGGCGCTTGATGCTGGACAATTTGGTGCATTACGTGGAGAATACTGAAAATTGTCGTTGAATTTACTTTATTTTGGGATTTCAGAATGCATCTGTGGATGATTTAACGGAAATTGCATAAATGAAATGTAGAAAAAAATTATAAATGGAATGAAAAAAAAACAGAAGAACGTGCATTGACTTTTAAACATAGGTGATCTCTATTTGAGTTTAATACTTTTATCTGCTTATTTTTTACTACTGAATGATTTGTTGATTATTGTATGATAAGCAATACTTTACATTGCAATGCAAGCGGAAAATTCTGTGTTTTGCTATGTCTTCTTTTGGTTTTGTATTATGATTTCATTTTCTGTTAACTTTTGGTTCTTGAAGTTGTTCTATAGTGTTGTTTACCCTTTTAGCAGCTCTTTCAACCTTTTGTTCAATCATTTGATCTGTTGAATTCTTACAGTTGACAAAAACAGAAAATGGAACTCTGACTCGTAAGGAGATTTTATCTGTTATTGATTGTTTAAAGAATCAGGTTGCTGCTGACCATTTTGTTTCCGTTCAGGTGCATTTTTACTGCACGAATTCACTGTTGATTTAACTAGCATTCTTTTTTATATAGTCCTCATAACATGTTAGTTAATTCATTTCCTCTTTTTTTTTTCCTCAGAGAAGGGTGGAAAAAAATAGGCAAAAGTTGATTGGTGTTACAAATCATCTGTATAGACTATCATTGGAGAGAAGAAACAACCAAACAATAAATACACATGGTAGTGTAGATTTACTAACAAAGAGGCAAAGAGAAGCGCTTGGTGTGCAAAATGGTATTGATGTAAGTAGTGGGGATAGAGATAGTCACATATCTCAGGAAGATGGTTATGCTTCTACCGCAGTTTATGGATCTAGTAACCCTACCAAGAATGTTATTCGTCCTATTAAGCTCAATGACAATAAAAGATTACCTCCTTATACCACATGGATATTTTTAGACAGGTAATTTTGAATCACATTGTATTTTGCGTACTTACTGTTGAGCTAATATTCCTCGTTTTCTGTATACTTCTTCTTTACATACATCTTGATTGTGAATTATTTCCATTGTACATTGGTAAGTTCTTGTCTATTTCTATACCTGACTGTAAAGTGCACAAGGTCAATCTCCTTTGGATTGCAACTTGACAATCATGTTGAAAAACCATAACATCTTGTAGAGAATTTTGATCTGGTTGCTGATAACGAGGTTGAGTGATGTATTCAACCAAGAGGTTTTGGGGTTCAAATTCCCTTGGGGGCTACCTATTTAAGACATCTATTTTTCCTGGATGCCAAGATTTTATGGCATGTTTGGCTTGAAAAAATGAAAGGAGAGGAAAGGAAGAGAGAGGAAAGAAATAAGGAGAAAAGAGATGAATTCCTTTTCAAACTGAACAATGAAATTGAGTCCCTCATTTTCTCTCTGCCTCTCTCCATTCCCCTCCTCTCCTTTCATTTCTTCCAACTGAACATAGGCATATAGTCCGGCAATAGTCGTTAGTTTAGAACTCGGTAAATGTAGAAAGGTCGAGGGATTTCTCGGTACACGGCATGAGAGCCAAACTGGGTTGAGCTTGACTTAAATTTTAGACAAAAAGAGCCTAAATGATTGGCAAGTAAGCATGACTTTTTTATTTTTTTTAAATGATGGCACAACATGGTACAAAACTCTTAGTTATTGGCTAGTTATATTACTTGTGAAAAAAAAAAGGAAAAAAATGAAAATTGCTGAGTTTTGTATTAAATTTTTTTGGTCTTGCTATTTTATTTTTACAAATTATATAGTTATTCTCCCTTTTTAAGTCCTTGTCTCATTAAAACTTTTTTTTTCAAGTTTAAATTCTTTAATATTCACGTTTTAATAGTAGAATACAAGTAAAATAATTAAAATTCTATACTTTGTTTTTCTCCAATTACTTCTATGTTCTACTTCAATCATTGACTATCCAAAAATAAATTTGGTATGGGATACTTAGGAGTTTTTGTTTATTGATATTCCATTTATCTTTGGTACCTTTATTTGACAGATTGAAACATACTTTTGCCTACTCTTCAGACTGTTCTTCTATTGGATTGTTCAGGATAGGCATTTTTTTACTAAAAACAACTAGAATATTCTCATTTCTGCATTGGAGCTGCTAGAAAATCTGTGTGCTGGACATGCAATCTTGTACATCCAAAATATATTTGTGAAATACTATTACTTATGTAAACATAGTCCATCTCCTTAATTGAGAGTTTATTTAAGTTGTATAATAATTTTGGTGCAGAAATCAAAGAATGACTGAGGATCAATCGGTGATGAGTCGAAGGAGAATTTATTATGACCAAAATGGTGGTGAAGCACTAATCTGCAGTGACAGCGAGGAGGAAGTAATTGAGGAAGAGGAAAAGAAAGATTTTGTGGATTCTGAAGATTATATTCTGCGGTTCGTATAATTAACTACCGATCTAAATTTGGCATCTTTTCTTAATTCCTTTTTTTTTATAATGAATTATATAGGAGTACTTTCAGTTGTTATCCAGATCACTGTTTGTCTACTAAGATTTGCTCTACGTAACTTAACTTCTTTATATTCTCCTCTTTCTTATTAAGTGCTTCACCTGAACTCTACGCTCATTCAAGTCCTTGCACTAACTGCTTGAATTAGCTTCATGACAGCTTCTCTTCTCTTGGATCTCTAATCAGTTTGATTCTTCTATGATTGCCTCTCCATCCCAGTTTTTCATGGCTCTCTAGTTAATTTCTCTTCTTTATGTTCTTGTTACTAGCAAGGGTCATCATTGCACCTGACTTTTTACCTGATTGAAAGAAGTTATTCTTTCAAGGTTTCTCTTTATGGACTATGACAGCATGCCATATGTTCTTGAAGAAACAGACAATTGGTAGTTGTGGGCATCATTTAATTTCAAGCTTGTTGGCTTGGGTACTTTTGAAATTTTTGCTTATTTGTTGCCAGGCATATCAATCCTGTTTGGCTTTAGTTCGCTATATCCAGTTTTTATATCTTAGAACCTTGTTTTCTGAAACAATTCTCATTTCTGTCACTGCCCATTCCAAATTATTCAGTTAGGAACTCAGGTTTTATTATACATATTGCAAAATGTTATATGAAGTCTTGTCTTTAACAAATTGTTCTTTATATTTTCAATTTTGAAATTACTAGCATGACTATTAAAGAAGTTGGTTTATCTGATGCCACGCTGGAATCACTGGCCCAATGTTTCTCCAGAAGCCCTTCTGAAGTCAAGGTATGCCCTTCTGTCCCACTATATTAGTGGTCTGTGTTTTGGTGCATTTTAGTTTGAATGAATTAGCATATTTCTTTGCTATTGTTTTCTCAACCATTTATTATCTGATAAAAACTGGTCATAGTGTAAGCTTTGGTCCAGCCGTTTCCCATTTGCTTTGTGCTGGCTTTACAGAGATATGAAACACCGTCAAACATCTTATACAGGCAAGATATGAAATTTTGTCAAAGGAAGAGAGTGCTGTAGGGGGCTCTAACAATGGGAATGATGAGCACACTATGAATAACTTTCTTGTAAAAGATCTTGAAGCAGCTCTGGATTCTTTTGACAACCTGTTTTGTCGCCGATGTCTTGTAAGAACCACACATCCATTTAGATTATGTTGTTTTGCTCCATTTTCGCACAACTAAGTCCATTGCTCTTTTCTTTGAATTTGCAGGTCTTTGATTGCAGGTTACATGGATGTTCACAGGATCTTGTCTTTCCGGTAAGTAGATTTTATTACTCACTGTTAAATGTTTATATGATTTGCTTGCCAGATTCTAATATTTTCTTGGTAAAATATTCTCAGGCTGAGAAACAACCTCTGTGGTACCATCTAGACGAGGGAAATGTACCATGTGGACCACATTGTTATCGATCGGTATATGCTGGGTGTTTCATTGATATGATTTATGTGGCTTGGGTTGTCTTCTTGAAGAATAGGAAAACAGTGTATTAAGTAGCTTCATATTCACTGTCAGGTTCTCAAGTCAGAAAGAAATGCTACAGCGTGCTCACCCTTGAATGGTGATATCAAAGAAAAATTCGTCTCTTCATCTGATGGTGCTGGGGCTCAGACATCATCTAGGAAGAAATTTTCTGGTCCTGCTAGAAGGGTGAAGTCCCACCAAAGTGAAAGTGCTTCTTCAAATGCAAAAAACCTTTCAGAAAGCAGTGATTCAGAGGTTGGGCCGAGGCAGGACACCACTTTTACTCACCACTCATCACCCTCAAAGAGTAAGCTTGTAGGGAAAGTTGGAATCTGCAAGAGGAAGAGCAAGCGAGTTGCTGAGCGCGCTCTAGTTTGCAAGCAAAAGAAACAGAAGAAAATGGCAGCTTTTGATTTGGATTCTGTTGCGAGTGGAGGTGTTTTGCCCAGCGACATGAAACTTAGGTCAACTTCTCGCAAAGAAAATGAAGATGCTAATTCCTCTTCACATAAGCATGCAAAATCTTCATCCTCTGGGAAGACCAGGAAGAAGGAAATGCAAATTCAGGACAGCCGCAATTTGATGCATGTCAGAGTTCCCCTTGGCTCGTCACAGGAGATCGTTTCTAATCCACCTGCAATCAGCACTAATGACTCCTTGAGAAAAGACGAGTTTGTGGCTGAAAACATGTGTAAACAAGAACTAAGTGATGAGAAATCTTGGAAAACTATTGAAAAAGGCCTCTTTGATAAAGGTGTTGAAATTTTTGGCAGGAACAGGTCAGTGGTTCAGATCTATATTCTGCCCTATTCAACTTTTTGCTTCCTCCTTTCATTAAATCATTTTTTGATATTAAGGTTGGACATGAATCTCGTTTTGTGCAATTCTGATTTGACCATTTATGATGAAAGTTCTAACTTATTCTTACCTCTTTAGTGGGAAATAATTTGTGTTCAATGGATTCTTGTACAAAAAAGGAAAATGACCATCATTGAACTCAATGATATTTGAAAGCTAACCTTTTTCAAATATTCCTTTACATCATGAAGTTATATACTAATTAATTTGGTTTTGTAGTTGCTGTTTCCATGCACAAGCTGTTTGGTTGGCAACTGTAAAACTCTGTTGCTAAATACAGCAATACTCTTTTCCCTTGTCTGATGCTTAGTTTGAAATCTTCACAGCATTTTGCATCTTCAGTCCTTATGCATTATTTCCAAGAAATTTTGCTCCCTGCCAGTTTATTGATCTTTCCGTCTAATAGATAGTCTTATTTTCCTGACAGCTGTCTGATAGCCAGAAACCTATTAAATGGTTTGAAGACTTGTTGGGAGGTTTTCCAGTATATGACTTGCTCTGAGAATAAACTATTTTGCCAAGCAGGTGATGCTGCAACTTCTCTTCTTGAAGGCTATTCAAAGTTTGATTTTAATGGAACTACGGTTAGTATTGTGATGTTGTGATTATATAGTCATAGAAAACTGTACTTTCTTGTCTCTAAGAACTAATCTTTCTTGTTTCTTTTTCATTTTGTGCTGGTGTATTTAAATGAACACTAGGGTAACAATGAAGTGAGAAGAAGATCAAGATATTTACGTAGGAGGGGTAGAGTTCGTCGCTTGAAGTATACTTGGAAGTCTGCTGCTTACCATTCAATTAGGAAACGGATCACTGAGAGAAAAGATCAGCCATGCCGGCAGTACAATCCTTGTGGCTGCCAAACTGCTTGTGGAAAGCAATGTCCTTGCCTTCTAAATGGAACCTGCTGTGAAAAGTACTGTGGGTAGGCCCTTATTCCCTTGTAGATCAGACAAATCGAACGTCAAGCTTTTCTTCTTTTGCATTCCCAGATTTTGCAATGGGAAATGGCATAATACTAAAGTTTCCTTTGAAGAAGGCAGCATTCATCCCAACTCTTTGGTATTTAACTTTGTATGTGCATCATGGGAATGAATGTAGACATTTGTTGATGCATGACTGTTTCTCACTGCAATTTTGGCAATTATGGTTTTTGTATGGAGATGGTTTCTTGTGAGTTGTGTACATATTATATACGCTATGGTGACTGTTATGATGCATGAATATCCTAATTTAAGGTTATATATTGATCTAGATGTCCCAAGAGCTGCAAAAATCGGTTCAGAGGCTGTCATTGCGCTAAAAGTCAATGTCGCAGTCGTCAGTGTCCATGTTTTGCTGCTGACCGGGAATGTGATCCAGATGTTTGTAGGAACTGTTGGATCAGGTGAGTTTTAATTTTGGATGCTAAATTTTCATTCAAACACTGACTAAAAATATCCTTTGACGTTTCTGATTAATCAGAATGCAGTTTGAAATTATAAGATCGATAATGCATGACTAATGTATTAAGCTGACCTATCTGAGGGTTTGTTGCTTGAAGTATTCTCAATTTTAAGGTGCTTTTAGAAACACTGACTTTTTGTACTTGTGTCATTTTATTACAATTAGATTAATCAGTATTACGAAAGAGAATTGTTTCTGCTTATAATGGGTCACAGAGGAATTGAGAATTTCCCTATTGAAAATTGTTTCTGCTTCCTGAAGAGATATATCTTTCCTTTCGTGGGTGGTTTTTGAGGGTAGAGATGCCTTCTTTTTCCATTTTTCTTGTATTTTCTCTTGGATAGAAAGTTAAATATATTTTTACTTTTAGAGAGCTTTAGCATCTCAAATGGTAGTTATTCATGGAACTTCCTCAAATTGGGAGTGAATGGATGTCATGGCCTTCAGACAGTTTGGTATCTTAAATTTTCCACTAGAAGAATCTATTGGATTGGACATCATATTGCCTAAGAGCCTTCATTCTAAGTTTAAATGTGTTGTGTTGGTATTCCTTTTTTACTTCTCATACAATCTGTTGTTTGAGCTTGCTAAATTGGTTTTGCAGTTGTGGTGATGGTAGTCTTGGGGTTCCTGACCAAAAAGGTGATAACTATGAATGTAGGAATATGAAGCTTCTGCTCAAACAACAACAAAGGGTAATTGTTTTTTGATTTCTCCATTTCTTTATTCTTTGATTATTGTCTTTACACACTGATGAATGTTTTTGAACTGTTAAGATATTCTCCTAATATTTTCTTACACGTGCTGGCTTGGGGTAAATTTTATTTTGGCTAAAGGGCTCCAGTTTTTATACAAGCATCTAATATTGATCTGTTTAAACTGCTCAATTTTGGGATTTCTTCACTTGAAGCAATATTATTTGTTCAATTTTCTACTTCAGTCATTGACCAATAGAGAATTACTTGTTTATTCTGGTCATAACTGTAAAATATAGATGTGATGCCCCTCTCCTGTAAATCAAACTGGAATAGTGTCTTTTTTGTGTTCTTCTGATGAAGTCATCAGAAATAAATGTCGATTACAGATTTATTAAAACTATTCACCTCTGCCAATTTTTAGATTTAGTGGTAAATTAAGTATATAGTAAAATTGTTGAACCTGTTTTTATAGCCTGTGAGAGAATTGATTTGTCCTGTACACTGTGTGGACAATTCCAGATATGTGATGATTCAACTTAATAATTTTAAGCGGCAATGTTTCAGTTTAGTTGAAGTCTCAAGTCTTTTCCCTTTTTTAAAAAAAAAAAAACTGTGTGTGTATTTGCTTGTGTGTGATATTCTTTTCCTATGCTTTGTTTTTCTTGTTCTTTGTATTTTGGCCTTTATTGATACTGGAGGTTGATACTGAAAATTCTATGGTATGTTTGCTGTTACTTGGGTCTAATTCTCATATATACGTGTACAGGTTTTACTTGGAAGATCCGATGTTTCTGGGTGGGGAGCTTTCTTAAAGGTAATATTCAGTTTTAATATCAGCTGAATCAATAAAGGGAAGCAGACTTGATGTGCTTGGTTTTCTTCTTGCTGCTGAAATTTTGATCTGCTCTCAGAATAGTGTTGGAAAGCATGAATACCTTGGTGAATACACTGGCGAGCTGATTTCTCATCGGGAGGCTGACAAGCGTGGGAAAATATATGATCGTGAAAATTCATCATTTCTGTTCAATCTGAATGATCAGGCAACATACATTGCCCATTTGTATTTTGACTAACTTCTTTTGTTTTGTACTTAAGTGATAGTCAGTCCATAAATTCATAAATCCACTTGTGTGCAGTTTGTTCTTGATGCTTATCGGAAGGGTGATAAACTGAAATTTGCCAATCATTCTCCTGATCCAAACTGCTATGCAAAGGTAATCAACCATTTGTCTAGTAGTAATACCTCAACCTGTTTCTCTAAACCAAAAGGTTATGTTGATGTTGTATGATTAGATTCTAGAAAGTACTGGAGCATTGCATTTCATGTGGTGTGCCAATTCTAAGTTTGTGCTCATTGTTTGGCAGGTCATCATGGTTGCGGGAGACCACCGTGTTGGCATCTTTGCCAAGGAGCGAATTAGTGCCGGTGAGGAACTGTTTTATGACTATCGTTATGAGCCAGACAGAGCTCCTGCTTGGGCTAGGAAGCCTGAAGCATCTGGTTCAAAAAAGGAGGAAGGTGGTCCTTCAAGTGGTCGTGCCAAGAAGCTTGCTTAGTATGAGCATTTATTTTTAACTTAAAATCAAATTTTATCATTCTTTTTACAGGTAGAAGCATCGCTTTCATTGATCTTAGGGAAAATGTGTATTCTCTTATCATTTTGTTGTTGGAGATGCCGAGATGCCATTGTCGTCTCCATTGATAACCTATTCAATTCTAACTTTAGATTCATGTTAATTCCAATGCAGCTTGTCCG

>CsSDG23

ATTCTCTATTCATCTTCTAATAGATAAGGCATTTCTCTCTCTCACTCTCTCCCAAGGCCATGGCCGAAGCTTCTAGAACGTTCCACACTATACTTCTCCCTTCATTCTCGCATCTCCACAAAGCACAATCTCCCGCTGGCTTCACAGCTTTCCCGCGCAAAAGATGTGGGCACCGAATAGTAGTTCACTGCTCAGTTTCAACAACTAACGACGCCTCTCGCACCAAAACTACAGTGACCCAGAACATGATTCCGTGGGGATGTGAGATAGATTCATTGGAAAATGCATCAACCCTTCAAAAATGGCTGTCTGATTCAGGCCTGCCGCCGCAGAAGATGGCCATACAAAAGGTTGATGTGGGAGAGCGCGGTTTAGTTGCTTTAAAAAATATTAGGAAGGGCGAGAAGTTGCTTTTTGTGCCTCCTTCTCTTGTTATCACCGCCGACTCGGTAAAATTCTTTCTAGTCCTCCTCCTCTTAAAACATGGAGTAAACTGTTAGCTGGATACTTGGTCATGATTTCCATTCTTTGACCATGTGTATTAGTTAGTGAGTCCTGTGGTGTATATGGCATTAGGGTATGCTTGAAATTGAAGTGCTGTGGCTTTTAAGTGACAATTGCTGTGAAATGGAAGTGCTTGGTTAACTTTAGCTGCTATGTAAAAGGAGGTGTTTACTGAACATTTTGGCAATTGTGCTTCAAAACTAAGCAAATTAAATCATGATTTGGTACATTAGCAATTTGAGAAAAAGCAAATTACATTTTTTGCAATTAATGAAACCTGTGATGCTTCAACTGATTTTGTGGTAGATTATAACTTACTGGAGCTTAAGATTTGTGTTTTCATATAGTGCACAGAGCAAAACAGCTTATGAATCAGTTTATCATCTAGTTATGATGGCTGTAGTTCCCTAGTTATGGGATCTTTTCTTAGTTTTCTGCTTTAGTGATTCTTCAAAGTTATTCTGCTGTCTATGTATAAAGTTTGACTTTGTGGGGGTGATAATTTCTCTCTCTCCATGGCATTTTGGATGATAGAAATGGAGTTGTCCAGAGGCTGGTGAAGTGTTGAAACAGTGTTCTGTCCCAGATTGGCCATTGCTTGCGACATATTTGATTAGCGAAGCAAGTTTCGAGAAATCTTCAAGATGGAGCAACTACATCTCAGCCTTACCCCGACAGCCATATTCTCTTTTGTACTGGTAAGTTTCCGTTCTTCAATTCTGGTTCGTATGTGCTTATTAAGGAGTTTATTCTTATACCACAGAAAGGGTCTACTCTCAGTTCTTCCCCCTACAGCCTCTTTATGTCATTTCAGGACTCGTGCCGAGCTAGATAGGTACTTGGAAGCATCACAGATTAGAGAACGGGCAATTGAGAGGATTACCAATGTTATTGGAACGTATGACTTTTTGAGCCCTTTTTTCTTCCTCCATGCATCTGCATCAGTTTATATTTTCATTTTAGTGCATTTCTGGTAATTCACTATTATCCCATCCCTCCTTGTGCAGATACAATGATTTGAGGCTCAGGATATTTTCCAAGTATCCTGATCTATTCCCTGAAGAGGTACTTATTCTATTTATCAACTGATATTCTTAAATGTTGTTTTGAACCATATCTTTTTCTGGACCAGCTGCTGGTATTAGAAATGTTCCGTTTATTGTCTTTTGAATTTTCTTAACAAATAATAAATATATAACTTTTTTTTTTGGGTTCCTTGCTTAGACTTATAATTATTAGCACATGAAAAACTATGATTTGATCAATATAGTCCATTGTTTAAGCTTCTGATGGATGTTATGAACATTCTGCACTACAGAGTTCCATAAACAATTAATTGTGTTGCTGTTATTACCCATAACCATCTCTAGTTTGATGTTGCTCCCTACTATGGATCACAGGTATTCAATATGGAAACTTTCAAATGGTCATTTGGCATTCTTTTCTCACGCTTGGTAAGTGAGCTTATTCAAGACGTCAAGGTTACTTTGCTGCTTGGTTCTTCTTAATGAAGTGAAACTATAATATGGTATTTAAAGATCACACGGAAAAGTTTTTTTATTAGGGTTGTAAATAAACACGGAAAATTTTTATCGCACAAATTCATTGTCAAATTGACTTCCATTAAGAATTTAGTCATATGTCGACTTTAGATCAGTACTTAACCACCCCCCCAACACAGGTTCGGTTACCATCAATGGATGGAAGGGTGGCCTTGGTTCCCTGGGCAGATATGCTGAATCACAGTTGTGAGGTATGTTCACAGATTAGTCTCTTTCTGAGACATCTATCATTTAAAATCTGTGCTGGTAGGAACATATTTGATTTCTAACTTAGTCAGAATAATCTTTACGTGATGTAGGTGGAGACATTTTTGGATTATGATAAATCATCACAAGGAGTTGTGTTTACAACCGATCGGCAATATCAGCCAGGTGAGCAGGTAAAGAATATTTAGATATCCCAAATAATCATATTTTCATGTCATTTTAGAAAAAAAGGGACTCATTCCCACTCTCCCAAGTATAGTATGTATTGCCTAGACTTGTCTTGATCACTAGTTTGTGTAGATATAATATTCTTCAGAGCATTTACAGTGATACTGTTGGGTAGAATGTTAATTCATTTGGAAAACTTGTTGCATCGATTAATGCAGGTTTTTATATCATATGGCAAGAAATCTAATGGAGAGCTTTTGCTATCATACGGATTTGTTCCAAGGGAGGGCACCAATCCCAGTGATTCAGTAGAGTTGCCTTTATCTCTTAAGAAATCAGATAAATGCTATAAGGAGAAGTTAGAAGCTCTGAGGAAGTATGGATTGTCAGCGTGAGTACCTGATGGATTTGCTCTGTTAGTTGGTTGTTCCAAAACCTTTTATCAAATTTTGGTCAATATAAGGATTAAATATATTCGCTGTCAACCTCTTTATAACTAACGTCAGCCTTTAGATTGTGATCCTAGTCTCTATCTCCCAATGAAGATTTAACCATATGCCATAATTCCCTTCTTACGTGATGTCAGCCTTTGGATTGTGATCCTAGTCTCTATCTCCCAACGAAGCTTTAACCATATGCCATAATTCCCTTCTTATGTGATGCTTTATCTTTTTTCACTTCATTTTAGAAACTGATCAATGCAAGAATGTAATTTCCATGGGCAGATCTGAGTGTTTTCCCATACAAATCACTGGATGGCCATTAGAGTTAATGGCATATGCTTATCTAGTGGTCAGCCCTCCAAGCATGAAGGGGAAATTTGAAGAGGTTCGACAATTTTAGATTTATTTTCTTCAGTCTCAGATTTTTTCTAATATCATATAACATATCTAAAATATCTTTTAATCAGATGGCTGCTGCAGCATCAAATAAAATGACCTCGAAGAAGGACATAAAATGCCCTGAAATCGATGAACAGGCATTACAATTCATATTGGACAGCTGTGAATCAAGCATATCTAAGTACTCTAGATTCTTGCAGGTGAAGGAACTTCTTTAACCGACTTATGATTCTGTACTTCATTCAGAAAACTTCTTTAATGTATTATAACATGTCGGAATTGGGGGACTTTTAGGCAAGTGGATCGATGGATTTGGATACGACATCTCCAAAGCAACTCAACCGAAGAGTGTTTCTGAAACAGCTAGCTGTGGACTTGTGTACAAGTGAGCGGAGAATATTATTTCGTGCACAATATGTAAGGATACTTTACTGATCTTTTCACCTTTTTAAATCATGGTTAATGTAAGCTGCATCATTAGTACTTCAAATTGTAGTAGCTCATGCATTATAATCTTTACATACGCCTTCAAATGCCATGCAAAATGTTCAATATTTGTGATTGAATGTTGCTTATATGACTGGTGTTGCAGATACTGAGGAGAAGACTGAGGGATATAAGAAGTGGAGAACTTAGAGCTCTAAGACTATTTGACAATTTCGTAAATCTTTTTAAATGAATACTTTGAGATGGGAATCTCTCCATGCCATCGCCAAATGCGAGCAACAAAGTTGTGTCGGGTGCTGCACATTTGACGGGCTGGACTTGTGCTTTTGAGGCTACAAAGTATATGGCCCAATTGGGCTTCCCAGCCATGTTCCAATCTTCTACTATTTTTGTTTTTCTCTGGCCAAAATGTTAATTTTTTTTACAATAATATGCTCTAAGTTAAATATTCATAGCCATAAGTTTTAAAAAAGGAAAATTGTT

>CsSDG24

ATGGAGGAATTGCAGAAGGCTCTTCAAGACCGTGGCTTGACTGTCACCGGTTTACCAGAGAAAGGCCGCTGTCTCTATACCACCAAAGATTTTTATCCAGGTCTCTCATTTTATTGTATTTATTTTATCTTATTTTCACTGGGGAACTATTTTTGAATTGATTATGTTTAGCTTTCACGTCTCAGGAGAAGTGATTATAAGCCAAGAGCCTTATGTGTGCGTACCAAACAACTCGTCTTCTATCTCAAGGTGTGATGGATGTTTCGCTTCGAGTAATCTCAAGAAATGCTCAGCTTGTCAAGTTGTGTGGTACTGCGGAAGCAATTGCCAGGTACTTTCAATTGGGTCACCACGCATTGTCATTTCCAATCGTAAGTGTTTAGCGTCGCCGTGAAATATCAAGTTGTCTTTGAGCTGAAATAATAGTGACTGTAATTAAATTCCAGAAATTGGACTGGAAGTTGCATCGCCTTGAATGCCAAGTTCTCTCTAGGCTCGACAAGGAGAAACGAAAATCAGTTACACCTTCTATACGTCTCATGCTGAAACTCTACCTTCGAAGAAAATTGCAAAATGATAATGTAACTAATTCCACGGCTTGTTTCAACTAAGTTGCTTGGTTGATTTTGTGAGACTACTTTTACCAAGTAATTGGTTTGTTTGACTGCCAGGTTATCCCTTCTACTGCCACAGACAACTATAGTTTAGTGGAGGCGTTGGTTGCTCGTATCCTTTTTGAATTAATTATTTGGTTCAATCAATTTGGTTTAGTGCTCTGCTTCAGTTACAACAAATCCCTAATGCCTGCTTTCTTTAACAATCTTTTTGGCAAAGACATGTCAGACATTGATGAGAAGCAACTACTTTTATATGCTCAGATGGCCAACCTTGTCAACCTGATTCTTCAGTGGCCAGAGATTAGCATAAAAGAGATTGCCGAAAACTTTTCCAAGGTGTCTTGCCAAATTTATTGGTGTATATATATTATTAAGTGTAGTCAAGCATAATTTCTTGAAGCACCCATAAATTCTTCTATGCCTTTATATCTAGATGCTAGAGAAATTCTCAAGCTTCTGATATCAAGCAGCAGTGCAATTGATTCGGGAATTTAATATTTAAACATCCCAGCTGCTTCAATTTGGCTGTGCAAAACATTAATAATGAAGATAAAAATTTAAAAATTAGGGTACTTGGAAACTTTTGCAGAACTCAGGAAGGTGTTTAAATCATGCTGTTTACTGACTTTAAGGGTACGAAATTTAACTGCAATTTCTTTACCTTTGGGAATTGTGTAAATAGAAAGATAGACATCTAGGTGCAATCAGGTTACCAATTGATTTCTCATTATTTTGTTTATGAAAAGTATTTGTCATGTATCATATCATGGAAAAATGTCACAATTAAGATTCATCCTGTAGGAAAGCCTTGGTATCTGATGAATTGATACTGTTATCTGATCTATCAACTATCTGACACAGCTTGCTTGCAATGCACATACCATTTGCAACAGTGAACTAAGACCCTTGGGGACCGGACTCTATCCTGTCATTTCTATTATTAATCATAGGTAAACTACTCAAGGAAAATATGTTTTTAATCCCTTAAGCTAGTGGTATCCTAAATGGTTCTAGTGTTGCAGCTGTTTGCCCAACGCTGTTTTAGTATTTGAGGGAAGGCTGGCTGTGGTGCGTGCAGTGCAGCATGTACCCAAAGGTGCAGAGGTGAGACATGATCTTTTTGGGTCATTTTGACATGTAGTACAAGAAAAAATACATTAACTAAATGCTTTGCTAGAATATCCCATATCATAAGGGTTAATAGATGATTACCTGGTTACCTGACATAACATATCCAAAAGTGATCTCTAAGTAGTGTTAATTGATTAGGAAAGTCTATTTAATGTGAAATGTAGGTGGTATAATGCTGCTTACTCTTCTTATTTCATGCGAAATCTGAATGTTATCTGTTCTGTTATATTCTGTTAGGTATTAATAAGTTACATAGAAACTGCTGGAAGCACTATGACTCGGCAAAAGGCACTAAAAGAACAGTACCTTTTTACTTGTACATGTCCTCGCTGCATTAACTTGGTACCTGTATTTTCATTTTAATTTGTTGCATTCTAGCTTTCATTCTGGATTTCCTCTGTTCCTCTCTCTCGCAGCTGATTAATCGAGCTTATATATATATATATTTTAAAGGGTCAGTTTGATGATATCCAAGAAAGTGCTATTCTAGAAGGTTACAGGTGCAAGGATGATGGCTGCAGTGGCTTCTTGCTTCGAGATTCTGGTAAGAAATAACTTTCAGTTTCATCATTGAGATGGCACTTGTGACTGATTCTTTCTTTTCTTTTTTTTTTGCTCTGCTTGAATCTGCAGATGACAAGGGATTTACATGCCAACAATGTGGCCTTGTTAGGAGGGAGGAAGAGATAAAAAAAATAGCAAGTGAAGTAAATATATTGTCAAAAAAAACTCTTGCATTAACATCCTGCGGCAGTATCCTTTTCTTTTTCTTTCCCCCATTCTGTTCCTGTAATATTTTTGCTTGAACTTTTTTTTTCTTTTTTGGCTATCATTTGGATAAATATCCTTTCTTTCACAAGAGGAGCAATGACTATCTGTTTCTGTTATTTGTGAACAAATAGATCCTTGACTGACAATAGATCATCAGGAAGTTGTCTCTACATATAAGATGATTGAAAAACTCCAAAAGAAATTGTACCATCCCTTTTCAGTCAATTTGATGCAAACCCGGGAAAAACTTATAAAGGTACATTATGAATTAGTTTTTTTTTTTTTTAAACCTCTCTAGAAAATTTGTTCTTTCATAACTTATAAAGGTACATTATGAATTAGCCGGGAAAAACTTATAAAGGTACATTATGAATTAGTTTTTTTTTTTTTTAAACCTCTCTAGAAAATTTGTTCTTTCATGATTGCCATTTCTTTTCAATCAGTAAGCATGCAGCTCAGCCTTTCACATTTATGTTTGACTTAGTTAATCGTTTAAAATGGTTTATCCTTGGTTTCGTCTGGATGCAATTTGTTAAGGTGTTATGGTGAGACAAGAAATGCTCTAATTGCTTCATTAGAAGAAAAATAGTCAGTAATTGGAAGGGTACATGGGTAAAGAAGTATAAATTGAACTACCATTCCTCTTCTTAGTCATTTGGGGCTGGATTTTCGAGACTAATAAGGATTTGTTGAGGTATGAGATCACATGAAATACTATTGAGATAAAACCGTTACATCCTGCTTCGGTGCATCCGAAATCAATCTACCCCAAAATCCAAATGTCCTTACAGATGAATCAACCAAATAACTTGTGTCACAATTTCATGACTTTTAGCAATGTATTTTGAGGTTAATGAATTTAGTAATGTCATATTATTTGTCTCTTCAGATACTGATGGAGCTGGAAGATTGGAAAGAAGCGCTTGCATATTGCCAATTAACCATTCCAGTGTATCAAAGTATGTTGTGTTTTCAGCAAGCGAGTTTAAAGCCATCTATGAATGCTATTGTCTGCTACAGAATATCGTGAAATTATAATAGAGAGCACAAATCTAGTTCAATGCATTCCTAAGTCCAAAATGCGTCTAAAATTTTACACTTAGGTTTTCTGTTCTACAATTGAGAATTTTGGATATTATTGCTGCCAGAATCCTTAATTTTACTCTCTTTTGAATGCAGGGGTATATCCACAGTTCCATCCTTTGCTTGGGTTGCAGTATTATACCTGTGGGAAACTTGAATGGTTAGGATTTGGCTTTGCATTGTTCTTATTTCAACTCCAAAACTCCTTTTTATCCATCTCCATGTGCTAACCTGTTCTATCATCTGTTAAAGGTTTCTTGGTGACACAGAAAATGCAATCAAATCCCTGACCAAGGCTGTGGAGATATTACGAATTACTCACGGGACAAATTCACCTTTCATGAAGGAACTCATTTTGAAGTTGGAGGAAGCACAGGCTGAAGCTTCTTATAAACTTTCATCCAAAGATGAGTAA

>CsSDG25

ATGTGCTTACGCCGTTATAGCCGTTGCCTGATTTCCCGCCTCGAAAGTCTTCATTTAGAAAAGAGGCAGCTTTGCTCCGCCGCAACGCACAATGGGAAACCCAGTCAACCCTCGCCGCCGCCAATCCAAGTCGCGCTCACCGAGTCCGCCGGTCGCGGCGTCTTCGCCACTCGGAGAATCGGGGCCGGCGATCTCATCCACACCGCCAAGCCCATCATAACTCACCCGACTCTTTCCACACTAAACAGCGTGTGTTACTTTTGTCTCAGAAAGATTACTTCGAGTTCGCAGCATTTTCAACATCATAATGCTCGCTTTTGCAGTGAAGTGTGCAAAGATAACGCTAAGGTAAAATGGGCTTTCTTTCAGGGGAAAAAAAATAAAAAAAATTCTTCATGGATTTTGAGGTTTACGATGTACCTATCACCTGTTTGCAGTTATGCTTCTATGACTAACTGGATTTTAAGTTTTAGGTTTGGGTCCCTAAGTTTTGACAAAGTCACAGGTAAGGACATTACTTGAAAAATTTTCTTGTGGGTCTTTGCTGGCTGATTAACATCATTGGCTTTTCAGTCACACCTTGAAATGAGTTTATTAAGGGGACTTAATTCTTTTTCAAAATTTTGATGAGTATAATGTTAAATCACTGAGGGTGCGTTTGGCACACTATAGTGTATTATGCTGTATTGTATTATTTTAATATTTGTGTATTGTATTATGGTGTATTGCATTAGCACTGTATTATAATAATGTTGTACAATGTTTGGTGCTACACTGTACTGTATTAATTTTTTTGTGATTAACTTAAATTATATATTTAGAAAATAATATTTATTAGTACCTAAAAAAATACAAAATATAGAAGTCTTAAATTTTAGAAATTTAACATTATTTTTTAGTATAAATATTAAAAAAATTTCCATAAAGATTGAAGCTTATAGCCTTATAATCGACAACATAACATTTATTTTAAATTAATATTATTTAAATTTATTCATATATAAATATTTTTATTAGTTATATTAAATTTTTATATATTAATTACTATAATATAATTAAATAATGTAATATCAAGTTATATTCTTATTTTTATATATTATTTAAGTACTTATTAATCAATTTGATATATTAATTATTATATAATTAAATAATACATTATTTAGTAATATTTTTAATATAATAAAAATTAAAATATGAATAATAAATTATTAATTTATATTAACAATTATGATATAAAAATAAAAGAAAAGAAAATATTATATTATATTAAAAAGTTATATTATTTATTAGTATTGAAAAAAAAGCAAAAGCAAAAGTAATAAAAAAAAAAGAAGCAGCAAATGCAAAAGCACAAAAGCTAATGCGGCAGTTTGCTAGCTTTTCTCGACTCCCGGTCAGGTTTACCTAATGCGGCAGTTTGCTGTATTAGTATACCCCTTAATCCCTTGTAAAATTTGCCAAACATGATATTGATATTAAGATAATGTGAACAATATTTTAATACCCCTTTAATACTATAACCAAACATGCCTTGATTGTATTATAGGATTTTTATCATTGTATGAAGGCAAGGGACTACATGGGAAAAAATGTAATCTGTGTTCTTAATTGCTATTTCATAAAAAATCGGGGACCCATATGCTATTAAACCCTGTAATGTTTTGCCTAAGGAGAGTTTGTGCCATAGAATGTTAACATTTATGTTGTGAAGCTTTGAGAAATAAATAATGGTGGAGGGATTTTTGACAGATTGTTTATAAGGATGAACAGAGTTCTTCCCTTTTAGCTATTAATGCCATCTGTTGCTAGCTGAGATTACAATTTGTGAATCCTTTATGCTTTGGTAGAGTCAAGGCATTATTCATTAGCGTGAAACCCTTATCTAAGCTTTACTGTGTTGTAATTTATGCAAGGGTAGTGTATGTCTTTACAGAGTATCTAAGGTAGTAGGAGAAGAGCTTGAACATTTTGCATGAACTTCTCAGAATACATAGTGTCATATAATCCAAGTTATGTTCATGACAACACTACTAGTGTGCATATTAGTTTTTGTTGCTCATTGGGGTATGTTTATAATTGTTGTGCTTATTGTTCAATGGCGGATCTAGAAAAATAATTTACCGGGGGCTAAATTAAATGACAATAAAATATAAAAACTGAAACTTGAATATATAAAATTTTTGATAACACAGTTACAATTATTCTCTACGCGATTTCATATTCTGAAAACGTTGTGCAATAAATTATAAAAATAAAAGAAAAGAAAATATTATATTATATTAAAAAGTTATATTATTTATTAGTATTGAAAAAAAAGCAAAAGCAAAAGTAATAAAAAAAAAAGAAGCAGCAAATGCAAAAGCACAAAAGCTAATGCGGCAGTTTGCTAGCTTTTCTCGACTCCCGGTCAGGTTTACCTAATGCGGCAGTTTGCTGTATTAGTATACCCCTTAATCCCTTGTAAAATTTGCCAAACATGATATTGATATTAAGATAATGTGAACAATATTTTAATACCCCTTTAATACTATAACCAAACATGCCTTGATTGTATTATAGGATTTTTATCATTGTATGAAGGCAAGGGACTACATGGGAAAAAATGTAATCTGTGTTCTTAATTGCTATTTCATAAAAAATCGGGGACCCATATGCTATTAAACCCTGTAATGTTTTGCCTAAGGAGAGTTTGTGCCATAGAATGTTAACATTTATGTTGTGAAGCTTTGAGAAATAAATAATGGTGGAGGGATTTTTGACAGATTGTTTATAAGGATGAACAGAGTTCTTCCCTTTTAGCTATTAATGCCATCTGTTGCTAGCTGAGATTACAATTTGTGAATCCTTTATGCTTTGGTAGAGTCAAGGCATTATTCATTAGCGTGAAACCCTTATCTAAGCTTTACTGTGTTGTAATTTATGCAAGGGTAGTGTATGTCTTTACAGAGTATCTAAGGTAGTAGGAGAAGAGCTTGAACATTTTGCATGAACTTCTCAGAATACATAGTGTCATATAATCCAAGTTATGTTCATGACAACACTACTAGTGTGCATATTAGTTTTTGTTGCTCATTGGGGTATGTTTATAATTGTTGTGCTTATTGTTCAATGGCGGATCTAGAAAAATAATTTACCGGGGGCTAAATTAAATGATAATAAAATATAAAAACTGAAACTTGAATATATAAAATTTTTGATAACACAGTTACAATTATTCTCTACGCGATTTCATATTCTGAAAACGTTGTGCAATAAATTCATTATCAATACTATTAAATACATCTTTCTACATAAACAATCAAATTATTATTTGTCCATTCATCTCCCATCCGATTCTGAAGTTCATTCTTCACAAATTTCGTTGTTGAAAATGTTCTCTCTACTGTGGTTGTAAAAACTGAAAAAACTTGGACTATTTAAAAATATGACAGGGATTATTTAAAAATTTATTAATTTTTCTTTCAAACAAAAATAGTTTTTTTTTTCAGTGGGGGCTTGAGCCCCCACTAGTCCTACACTACATCCGCCTCTGCTTATGTTTAACTGTGAAAGCTAATGTTTACCAACGACGAAGCACTAATACTTGATAGTTAGTGATTCCATTTTACAGCAGGTTGTAGTAGCTGAAGTCCCCCTTAGTATTTCAATAGTTTATTTGCTTAAGAATACAAGCTGGCTATAAGAGCTCTTACTTTCAGTGAGTTGAATTGGTTTGAACTTTAAGTCCTTTTTGCCTTTTAAAGAATCTCTGTTTTATTCTCATATTCTCAGGCATTTTATGATGTTGAGAGGAGAGCGGATTGGTCTGTTTTCAATGACTATTGCCGGTATGTGAGTTTCTGAAATTCTTTTAATTTCACTTGCTTGAAGGTTGATAGATTTTCCCAATTACATTTTGTCTACATCATTTTTTTATGGGATTTATCTTCTTTTTTTGCATTCAGTTACATTTCATATTTGTGTCATATTAGATTTGTACATATCCTTTTTGTCCTACTTTGAGATTCTTTTAGCAATGTTCAGTGCATCTAAAGGTATTAATTAAGACTTGTTACAGCAGAATTTGTATTTTCTTCATTCGCTTTCCCTGAGTAACTGTTGGTATCACTACATGGAATGAAACGAATAAAATAACACAGTATGATAAATTCTTAAAAATAATTGGCATTTTTGAGTATATAGTATCATGCATCATGTTAACATGGTGCAAATTTTAAATAGATGGCAGCTCATCAAGTTTTTTTTGTTAGGGGCAAACACGCAGGATTTTCCTTTAAGCCATTTGAATTACCTTTCATAGTTATTATGGGTTTATTGCCAGCAAGAGCAGTAATGCTATTAGAAATATTTTGAACATGATAGTGGTGTTTCTATTTTTAGTTTTCTGTGGGACTATTGATTTACCAACACATGTGGTCTCCTGAAGTCCAACAGCAGTTTATGTTTTCATGTCAACATGATTTCCTAAATTTTCCTAAATTTGTTGTAATTTTTAACACTTTTCAGTGTCTGTTCTCATTTTCATATTGTATTTCTTTAATTTTTCATTTTAAATACATTTTCTATAAAGGAGTCAAGGATTGAAATACCCTCTTCTGGTGAAGCGGTTGGCTTGTATGATTATATCAGGAGCTGAATCTGCTGACTGTATTGACATACTTCAACCTGCTAGTTTATCCCCTGAATTGATTTTGGCGGTAAGAATCTTATATTAATATTGATCATGACCGATGTAGAAATTTTTGAAATAAGAAAGAATTGTTGGTAGAGCTGTTAGATCATGAATTCTATTCAAATCAATTCATTACTTTGTTGCACCAAGTAATTGGAGCTTGAGAACCTATTATTTCTGCATGTGATTTGATCACAGAAACAATAATTGTTGCTTGTCTACCAAAGGAAATGCATTATTCATAGTTATAGAAGCAATAATTAGATAATAAATATTCAGTGAGCTGATCTATTAATGACCATGATGATTAGGGGTTTAGCATCCCTTGCCATTATTTTTGTGCATATATGTTCTTCTTTTGGTGAAATTTTAAATTTGCGGCATGCCATCTTACTTTTATGAGAATTTTCAGATGGAAGAGGGTTTTGTTATGCTAAGGAGTGCCTTTAAAAAGGCAGGTATAGATGATGAACAGATGAAATGTATCCTCCACATCAATTATCATTATTTTTTGTTATCTTATACTTTTTTCTAGCTCTATGCCAGTGTTTTTTGGGAGAACAGTGATCTTTGACATTACAAGTTCTAAACAAGCAATGGTACACTAATGTACTGGCTCAAATTCGTATCAATGCGTTTCGTATTGAATTGGCTGGAGGATTGTATGAAGATCTTCTTTCATCAGCTGCAGCCTCCATAGAATCTGAAATTGCAGTTGGGAATGCAATTTATATGCTTCCATCCTTCTATAACCACGATTGTGGTTAGTTTTTCCTCTTCTTCTTTTTAACTGTTTTACTTTATTTTTCCTCTTCTGAGGTTCTAGGTGCAATTTAACCTTATTTACATAGTGCATGATCAGATATCAATTGATGTAGACTACAAAATAGAAGAAAAATTAGGACAATTGGAAGAGAAGAAAAAGAATTAAAGGGAGATAGAATATAAGATTATTGGGGAAGGAAGAATCTATGGGGATTGGCGAAATAACAATTCAATGATCGCCCAAAACTGGTTCAAGTGCAGATCTAGTGATTGTTAAAATGTTCATAGAAATAGATGACATGCTAGGATAAGCCAAACAGAATTTTTGTGCCAAAAGAAGAGAGAAAATAAGAAAGGAACTGAATGAGCTGAATTTGGATTGCAGATCCGAATGCACATATTATGTGGATAGACAATGCAGATGCAAGATTGATGGCCCTTCGTGATGTTGAGGAAGGTTTGTCCAGCAATCGCACAGCTTTCATGTTTCTTTGTTTTTCCGGTTTCTTACCCAATATTAAAATCTCTTGTAGGTCACTTCTATAGCATGCATGTTAGGGAAATGAATTTTCATCTGTTGAGTAGCCAATGAGAATGAAGAAAAAGAATAGGTTGCTTTCACATTAAAAATTTCACAGCATTGTTAAAGAAAACGAGGGAAATTAGCAGGCAGAGCTATGAAGATGCATGCATTCAAGATTAACGTTTTCATTCCTCAAATCCCACCATGCAAAAATATTCAACATAATAATAACAATGCCATAATCTTAATTTTATGTTTATTTCACAGGGGAAGAACTTCGGATATGCTACATCGATGCAAGTATGGCTCGTGATGCTCGTCAAGCTATCTTAACGCAAGGGTTCGGTTTTCAGTGCAACTGCTTGCGGTGTTCATCTGGTGACTGAGAAGCGTTTTCTCGTGTTTTCCTCTGGTTTTAGCACTTTGGTAACCGAAACGATCTCTTGTCGACATACCAGTAATGTTATTGATCTGCTAGCAGTGATAAATGATAAATCGTTATAAATAGGGGAAAAGAAAAATAAAAAT

>CsSDG26

AACAGAAAATTTAAAAAAAATAAAAAATAAAAATAAAAACACGCACGAAGGTTTCGTCTTTCAGACCAAGAACAAGAAGGCACAACGCGAGCGCGAGGAGAACACAACACAGATATTTTAGCAGGGAAAAGAAGAAAGAGAGAACGCGAATGATTTACAATTGAAAGATATTTCATTATCTCGAGCTCTTGCTTTCTCGCGATCTAACGAGACGAGAAAGTCCTCCTTAATAAATTTTCCTAGTAACGCAGAAAATATATCTTCGCCGATCTGTTTATTCAGCTCGTGGATTTTTCCGGTAATTTTTTCTCTGTTTCCTTCTTCGTCTCTCCATCTGAATTCAGTCACTTTTTTCCCTTCACATTTGTTCTTAAATCCAAACCCTAGGATTTCTACTTCTCAGTTCGTTCAATTTTGACCTATTTTTTATTGTTTCTGCTAACATAACCCTACATTTGGTTGTTTTTTTTAAATTAGGGTTTCGGAATTGGACGTCGCGGTAAAGAGAATTGAATTGAATTGGTTTGCAGTGATGAGCATCGACAGCCCCTGGAGAGGAGAGAGCTAGGGTTTGTAGGTGCTTTCGTTTTATTAAAAACGTGGAAAGTGGAGAATTCCATGGGCGATGGAGGTGTGGCTTGCATGCCTTTGCAACAGCAGCAGCAGCACAATAGTATCATGGAGAGGTTCCCAATTTCTGATAAAACAACAATTTGTGTAGGCAACAGTAGCAACAACAGTAACAAGACTAATAACAACAGCATCAGTAATAACAATGATAACAAGACTAACAACGACAGCAGCAACAACAATGGCGGCAGCAGCAGCAAAAACAATGAGACAAATAAAAGCAATGTGAAGAAGAATGGGGTCAGCACAAAGACAGTTAGAAAGAAGATAGTGAAGATAAAGAAAGTTATTGCTGTGAAGAAAAAGGAGGTGCAGAAGAATAGTGGCAGCAGTAAAAGTAACAACAATGGTGAAAATATTGACAATAAAAATGTTGAAAATGGTGGAGTAGTGGGGGAAGTTGTTACTGTTGATAAAGATAATTTAAAGAATGAGGAAGTTGAGGAGGGTGAATTAGGGACGCTGAAGTGGGAAAATGGTGAGTTTGTGCAACCAGAGAAATCTCAACCACAGTCTCAGTTGCAGTCCCAGTCAAAGCAAATTGAGAAGGGAGAAATTGTTGTTTTCTCTTCTAAATGCCGTAGAGGAGAGACTGAAAAGGGAGAATCTGGGTTGTGGAGAGGAAACAAAGATGATATCGAGAAAGGAGAGTTCATTCCTGATAGGTGGCATAAAGAAGTAGTTAAAGATGAGTATGGTTACAGCAAGTCACGCAGATATGATTATAAACTTGAACGTACTCCGCCGTCGGGTAAGTACTCAGGTGAGGATTTATATAGAAGGAAGGAGTTCGATAGAAGTGGTAGTCAGCATAGTAAGAGTTCATCCAGGTGGGAGAGTGGTCAAGAGAGGAATGTAAGGATCAGTTCTAAGATTGTGGATGATGAAGGATTGTACAAAGGTGAACACAACAACGGGAAGAACCATGGAAGAGAGTACTTTCATGGCAATAGGTTTAAGCGGCATGGTACTGATTCAGACAGTGGTGACCGTAAGTATTATGGGGATTATGGGGATTTTGCGGGTTTGAAAAGTAGAAGGCTCTCTGATGATTATAACTCTCGTTCTGTTCATTCGGAGCACTATTCACGTCACTCAGTAGAGAAGTTTCACAGAAATTCTTCATCGTCGAGAATATCTTCGTTAGACAAATATTCTTCCAGGCATCATGAACCTTCTTTATCTTCCAGAGTGATTTATGACAGGCATGGGCGTAGCCCGAGCCATTCTGATCGGTCTCCACATGACAGGGGCAGATATTATGATCACCGGGATAGAAGTCCAAGTCGTCATGACAGATCCCCATACACCCGTGATAGATCCCCTTATACTTTTGATAGGTCCCCATATTCTCGAGAAAGATCCCCGTATAATCGGGATAGATCCCCTTATGCACGAGAGAAATCTCCATATGATAGGAGCCGCCACTATGATCATAGAAACCGAAGTCCCTTTAGTGCAGAGCGGTCCCCACAAGATCGAGCTCGATTCCATGATTGCAGTGATCGAACTCCAAACTATTTGGAGCGGTCCCCACTTCATCGGAGCAGGCCTAATAATCACCGAGAAGCAAGTAGCAAAACTGGAGCTAGTGAAAAACGTAATGCTCGGTATGATAGTAAAGGGCATGAGGATAAGTTGGGCCCAAAGGATAGTAATGCACGATGCTCACGTTCTTCAGCAAAAGAATCCCAGGATAAAAGCAATTTGCAGGATTTGAATGTTTCTGATGAGAAAACTGCCAACTGTGAGTCTCATAAAGAAGAGCAGCCTCAGAGTTCAAGTGTAGATTGCAAGGAACCACCACAGGTCGATGGACCTCCTCTTGAAGAGCTTGTTTCTATGGAGGAAGACATGGATATATGTGACACGCCGCCTCACGTCCCTGCAGTGACTGATTCATCTATAGGGAAATGGTTTTACCTTGATCATTGTGGCATGGAATGCGGGCCTTCAAGATTATGTGATCTGAAGACACTAGTGGAAGAAGGCGTTCTTGTGTCGGATCACTTTATCAAGCACTTAGATAGTAACCGTTGGGAAACCGTTGAAAATGCAGTTTCACCATTGGTCACAGTGAATTTTCCATCTATTACATCTGATAGTGTGACACAACTAGTCAGCCCTCCAGAAGCTTCTGGTAATCTACTGGCAGATACTGGAGATACTGCACAATCTACTGGTGAGGAATTTCCAGTTACTTTGCAGTCTCAGTGCTGCCCTGATGGCAGTGCAGCTGCACCTGAATCTTGTGAAGATCTTCACATTGATGTGCGGGTTGGAGCTCTATTGGATGGTTTCACCGTTATCCCCGGCAAGGAAATTGAAACTCTTGGAGGTATAAAGTTATTTACTGTTTTACTTCACCTTTCTTTTAGAGTAATTAAACTTTGCTTTTCTAATTATGGTTAGAATTTCAGTATAATGTTTTCTACTGTTTTATTTCACCTTTTGTTTAGAGTTATTAAACTTAATTCTTCTAACTACAGTTAGAATTTCATTTCTTGATTTGATGTCTTTTTTATTCCCCTGTTTCTGAAATTTCACGATCTTGCAGAAATCTTGCAAACAACTTTTGAACGTGTGGATTGGCAAAACAATGGAGGTACATATTCATAGTTGAAGCCTTGTTGTTCAAGTTTTGTAAATTATATTGAATGGTTTGTAAATCGTAAATTGTACTTACCTCAAGGATACCTACAGGGCCCACTTGGCATGGAGCTTGTGTTGGGGAACAAAAACCGGGTGATCAGAAAGTTGACGAGTTATATATTTCTGACACCAAGATGAAAGAAGCTGCAGAATTGAAGTCAGGCGACAAGGACCATTGGGTTGTCTGTTTTGATTCTGATGAATGGTTTTCTGGTCGATGGTCGTGCAAAGGTGGGGACTGGAAAAGGAATGATGAAGCAGCCCAAGATAGATGTTCTAGAAAGAAACAAGTTCTAAATGATGGTTTCCCTCTGTGTCAGATGCCAAAATCTGGGTACGAAGACCCTCGATGGAATCAAAAGGATGATTTGTATTATCCTTCTCACAGCAGAAGGCTCGATCTTCCTCCTTGGGCATATGCCTGCCCAGATGAAAGGAATGATGGCAGTGGTGGTAGCAGATCAACTCAGAGTAAACTTGCTACAGTAAGGGGAGTGAAGGGAACCATGCTTCCAGTTGTCAGGATAAATGCTTGTGTGGTTAATGACCATGGTTCATTTGTTTCTGAGCCTCGTTCAAAAGTTCGAGCAAAAGAGAGGCATTCTTCGAGGTCTGCTCGTTCATACTCTTCAGCTAATGATGTCAGGAGATCATCAGCAGAAAGTGATTCACATTCAAAAGCTAGAAATAATCAGGACTCACAGGGCTCTTGGAAGAGCATTGCATGCATTAACACACCAAAAGATCGTCTTTGCACTGTTGATGACTTGCAACTGCAACTGGGTGAGTGGTACTACCTTGATGGTGCTGGGCATGAACGAGGGCCCAGCTCATTTTCAGAGCTACAGGTCTTGGTGGATCAAGGTTGTATTCAAAAGCATACCAGTGTTTTCCGGAAATTTGATAAAGTTTGGGTACCACTTACCTTTGCTACAGAGACTTCTGCATCCACTGTCAGGAATCACGGGGAGAAGATTATGCCATCTGGTGATTCTTCAGGACTTCCTCCTACACAATCTCAGGATGCTGTCCTTGGTGAAAGCAATAATAATGTGAATTCCAATGCTTTTCACACCATGCATCCACAGTTCATTGGTTATACCCGTGGGAAACTACATGAATTGGTAATGAAATCGTACAAGAACCGAGAGTTTGCTGCTGCTATAAATGAGGTTTTAGATCCATGGATCAATGCTAAACAGCCAAAGAAAGAGACAGAGCATGTGTATCGGAAATCAGGTACAAAATATGTCGCTTATATGTTTTTGTTGGCATCTTTCTCTTTCTCCTATTAGTTGCATGATACAGGCTTGTAGAATTTTCTTCCCTCCTAATGTCACATTGGTGCATACTAAGTTATATATCTTAGCTTTTAGGTTTTACTTATAAAATGTACAGTAAAGCTTAGTTTCCATTTAGCTATCTATGCCATAAATACTGGTATGCGTAGGCAATCGTGCCTTTTTCCACGGTTTCAACCCATATGCTTCTTGATTGCTCTCCTGTATATTTATGTCTGAGTGCATCTTTCTAATCTGAGGCCTTTTTCTTTTGTTGTTGGGTGGGGATTCTGTAAATTCAGAGGGGGACACACGTGCTGGCAAAAGAGCTCGGTTGCTGGTTAGGGAAAGTGATGGTGATGATGAAACGGAAGAAGAACTACAGACAATTCAAGATGAGTCCACCTTTGAGGATTTATGTGGGGATGCTTCTTTTCCTGGAGAAGAGAGCGCAAGTTCTGCAATTGAGTCTGGAGGCTGGGGCTTATTGGACGGTCATACGCTGGCACATGTTTTCCACTTTTTGAGATCTGACATGAAATCCCTTGCCTTTGCTTCTTTGACTTGTAGACATTGGAGAGCTGCTGTCAGGTTTTATAAGGGCATTTCAAGACAAGTTGACTTGTCATCTGTAGGTCCTAACTGCACTGATTCACTAATCAGGAAGACCTTGGTAAGTTTGGTACATGTTTGTTGTAAACACCATCGCAAATGTTTTGCATTACGCGAGTTTTAACTTGATCTTCTGCTTGCAGAATGCTTTCGACAAAGAAAAGTTAAATTCTATTCTTCTTGTTGGTTGCACAAATATTACTTCTGGGATGCTGGAAGAGATTCTTCAGTCATTTCCCCATTTATCTTCTATAGATATTAGAGGCTGTGGCCAGTTTGGGGAGTTGGCCCTCAAATTTCCAAATATTAATTGGGTCAAGAGCCAAAAATCACGCGGTGCCAAGTTCAATGATTCACGTTCCAAAATAAGGAGTCTGAAACAGATTACAGAGAAGTCTTCATCAGCTCCTAAATCTAAGGGACTTGGTGATGATATGGATGATTTTGGTGATCTGAAGGATTATTTTGAAAGTGTAGATAAGAGAGACTCAGCAAACCAATCATTCCGCAGAAGTTTATACCAACGCTCAAAAGTTTTTGATGCTAGAAAGTCCTCTTCCATTTTATCTAGGGATGCTCGTATGAGGCGATGGTCCATTAAGAAATCTGAAAATGGATACAAGAGAATGGAGGAATTCCTTGCTTCAAGTCTGAAGGAAATCATGAGGGTGAATACCTTTGAATTTTTTGTTCCCAAGGTAGTCATTTTTTTCAGCATCTAGCTATCATCAGTAGTATTTAAAAAAAAAAAAAAAATCTGTAGTGTTTACATATGGAAATAAGTCTTCATTCCATATTATTCTTTTGTTTATCAGGTTGCAGAAATTGAGGGAAGAATGAAAAAGGGATATTACATCAGCCATGGGTTGGGCTCTGTCAAGGATGATATCAGTCGGATGTGCAGGGATGCAATAAAGTAAGTTTCAGTAGTTTTTTGTATCCTGCTTCTTGTGTTTTTGTTAAAGATATACCAAATCATCAGGACTTTAGATCAATTCAATCCATGTAGCTTTTGCTGACAATGAGATAGCTGCTCATAAGGTTGGACTGCAATTTTGTCATCTGTTGCACCTACTTCAATATAATATTGTAAAGTCAAAGGGAGTTAAGATGCTATTGGTTGAGAAGGATTTTGTTCCAGTTAAGCCTGAACTTGAAACATTGAGTCTGGGATTTATCATCCTTTAGCTTCTGTGGATTTTATTCTGCTGCCTGTCATTTTGTTCTCTGTTTGAAGTTTAGAACTCAATTTCCCTTTTTTTAAGGTTCATGTATGGGGAATTAGAACCCTTGACTTGCTTCCATTCCACTGCAAGAGGCCTAGCCAGTTGATTTGTCCCCTTGTGGACTGACATTATTGCATTCTTATATACCTTGTTCTATCAAATTTATTTTATTGTCTGTAACTATTATATTTTATCTCTTTCTACTTTGCTGCATATTCCTCACCCCTTTCTTTTATGTGTTTGTTATCAATAGTGCATCCGTATGCTGCTGCTGAATTCTCTTCTTTTGATTTTTTATTTGCCTGTATCATTCTGTGGGTGCAGAGCAAAGAATCGCGGTAGTGCTGGGGACATGAATCGCATTACAACATTATTTATCCAGCTTGCTACACGATTGGAACAGGGTGCTAAGTCTTCTTATTATGAAAGAGAAGAGATGATGAAAAGTTGGAAAGACGAATCTCCTGCTGGGTTATATTCTGCTACCTCAAAATATAAGAAGAAGCTCAGTAAAATGGTATCTGAAAGGAAGTACATGAATAGGAGTAATGGCACTTCTCTTGCAAATGGCGATTTTGATTATGGAGAATATGCATCTGATCGAGAAATCAGAAAGCGTTTGTCCAAGTTGAACCGAAAATCACTAGACTCAGGAAGTGAAACATCTGACGACCTTGATGGATCTTCTGAAGATGGCAAAAGTGACAGTGAAAGTACAGTGTCCGATACAGACAGTGACATGGATTTCAGGTCAGATGGTCGAGCTCGAGAGTCCAGAGGAGCTGGAGATTTTACAACAGATGAAGGTTTAGATTTTAGTGATGACCGTGAATGGGGTGCTCGGATGACAAAAGCTAGCCTGGTTCCTCCGGTTACTAGGAAATATGAGATCATTGATCAGTATGTTATAGTTGCAGATGAAGAAGATGTGAGACGGAAGATGAGGGTTTCTTTGCCTGAGGATTATGCTGAGAAGCTCAATGCACAGAAAAATGGAAGTGAGGAGTTAGATATGGAACTTCCTGAAGTCAAGGACTATAAACCTAGAAAACAACTCGGCGACCAAGTGTTTGAGCAAGAAGTATATGGAATTGATCCTTACACTCATAATCTTTTACTGGACTCCATGCCTGATGAGTTGGATTGGAATCTTCTGGAGAAGCATCTGTTCATAGAAGATGTGCTCCTTCGTACCCTTAATAAGCAAGTTAGGCACTTCACAGGCACTGGAAACACTCCGATGATGTATCCTTTGCAGCCTGTGATTGAAGAAATTGAAAAAGAGGCTGTGGATGACTGTGATGTTAGAACAATGAAAATGTGTCGAGGTATCCTGAAGGCCATGGATAGTCGCCCTGATGATAAATATGTTGCTTATCGCAAGGTCCATTACATCATCCATTTATTACCTGGTCTTAATCTTATTTTCACTTGTTTCCTTTAAACATACAGTTTTTTACCATGAGATGCTGATTCTTCACCTTTTTTGTACTGTCCGTTTGAATATATGGAGCAGGGTCTTGGAGTTGTTTGCAACAAAGAAGGTGGTTTTGGAGAAGATGATTTCGTTGTGGAATTTCTGGGAGAGGTATGGTTGGTAATCGGTTCTTGGGTAATTTATGTTACACTGCAGTTTTCATTCTGAATTATCAAGTGTAAATATATGCTTTCTCATAGATAAGTTGAAATTTCAGATTTTGATCTCTGCACTTCACCTTTTTAATGCAAAATTATTTTACAAATCTAAGAAATGGTATCTTCGACAAAAAAACTCTTTGGCCTTTTCTATTCCTTGTTTCTTTTATCATATTTAATATTTTATTTGTTACTATTAGTATTATTTTTTTCGGGGTGTCTTTCCTTTTTTTTTTGTTCCGCCCCCCACCCCCCCGGGGGGGGGATGTTGGGTGGGTTGGGGGGGCTCTTTATTTTAATAAGAGTGTGTCCAATAGCTGTTCAATTTTAAATGCATCTAATATACATTTAGCAATTAGAGGGTAAAATTATGCTGTGTTCTACTATTTATATTCATGAATCTTCTGTCTTTATGATGAATGCTGAAGCTTCTAAATTTTGATAGGTTTATCCTGTTTGGAAATGGTTTGAGAAGCAAGATGGGATTCGATCATTGCAGAAAAACAATGAAGATCCTGCTCCAGAATTTTACAACATTTATCTTGAGAGGCCAAAGGTATTACTTGGACGAAATTTACGCTGTAGACAGTAGATCTTTCATTGTGCTTGTCAACATTTTGTTGAACTTTGCCTGATCAGTTGTCTTGATTTTCCAGGGTGATGCTGATGGATATGATTTAGTTGTTGTTGATGCCATGCACAAAGCAAACTATGCAAGTCGAATATGTCACTCGTGCCGACCTAATTGTGAAGCAAAGTAAGTCCTCTTGTTTCTGGATGAATAAATCATACCCATTCTATATACACAGCACAACTGCATAGTTTTTTAATTTTCCTCGCAAGTTACCATGACATCACTCCCTTAGATTCGAACTGTTTAACTCTTGCTTCCACCGGAAGAGGCCAACTAATTGGGCTATCCTTTGGGCCTTGGAACGCGATATCTGAGTATTTATGAGAAGATTAATGCTATTTTTGCTGACAACTCTTCAAAGTTTGATTTTAAATCTCAGGATTCTGAATCATTCGCTTTGGAGTCTACTGAAATCATGTGAAATATTCCTTTTTTTGACTGTTTGACTTTTTGGAGAAATGGATTGGTGACTCACAATGTAATTATTTTTTATTTAATTTCCCTTTTGTAGATGTACATACTTGCATGTATGCTAGAAATTTCTGTTTTCCGGCTAGTGAATTAAAATCTTGTACATGTATTTAATTTTTTTTTAAAGTATTGGAAATAAAATGTGTTGTCATTCTATATTTTTATCAGTTAAATTTTTTCTTTTGCTCTCTGGGTCTATGCTAAGCCTGGATTATATATTTTTTAGCCATCTGTTTTATTGATATTTGGCTGTTTGAATCCCGATATGTATCTGAAGTCTGTCCATGATCATCAAGTAATTTGTAGCAAATAAGTATTCTCTTGGATCAAGATTCTGGCAATGCTGAATTTGTTATCCACATTTTTTTCCGAATGCAGAGTTACTGCTGTGGATGGTCATTACCAGATTGGAATCTACACAGTACGGGGAATTCATTATGGTGAGGAGATCACATTCGATTACAATTCTGTTACAGAGGTATATCATCTACCTGTCAGGCTAGCTTTCATCGCAGAAAAATTTCTTTATGTTTTAGTATCTGATGTTAACAACTCCTAATATTGCAGAGTAAGGAGGAATATGAAGCTTCTGTCTGTTTATGTGGCAGCCAAGTTTGTCGAGGCAGCTACTTGAATCTGACAGGAGAAGGGGCTTTCGAGAAGGTACAGACTGCTAAGTCATTCCTTTAGCTTTTAAAACCTGGTCTCAATTTTTGTGCTCAATGACTATTAGTCTAACTAGAAACATTAGTGGCTCTGGCTCTGCTCAAGAGCAACATTCTCTCGTGCTTGATTGGAGATGAGTGGTGTACTTTCCTTTGAGCAGATAAAACTCTGCAGCCAATTGATCTGCTGATAGTATTCTGCTTTTAAGTTAGGAGTAAGTTCATTTTTCTTAACTGAAGTGTATTGCCTATCAGGTACTGAAGGAGTTGCATGGATTGCTGGATCGTCATCAACTAATGCTAGAAGCTTGTGAATTAAATTCAGTGTCTGAAGAAGACTACCTTGAGCTGGGGAGGGCAGGTTTAGGCAGTTGTCTGCTTGGCGGGTTGCCGAATTGGGTGGTTGCATATTCAGCTCGTCTGGTGTGTCTGCTGGATTGTTAAATTTTTTTTTTTTTGGATTAAAGTCTTCTGTGTCTCTTTTGTTTTCTTTCGAGGGGTTGTTGATTATTTGACTTACATTTTGACAAAATTCATTTTGGTTGAAGGTGAGGTTCATAAATCTCGAAAGGACAAAACTTCCTGAGGAAATCCTAAGGCATAATTTGGAAGAGAAAAGGAAATATTTTTCAGATATATGTCTTGAGGTTGAGAAGAGTGATGCAGAGGTTCAGGTAATGTTTACACTCTCTATTCCATTCTCGTCTCTTCAACTACCTGTACACCTGTCTGAAGACTCATATTTGAAACCCTCTGTGTGAAGGTATGCTTGTGGTTGCGATATCTGTGTTCTTACTCTCTTTCTCTTTCTGATTGATGCAGGCGGAGGGTGTGTACAACCAGAGGCTGCAGAATTTGGCAGTTACTCTTGACAAGGTGACTCTCTTCACACATTTTGCTAATTTCTATTGGATGTTAAGACAGAGTTTGGTTTGAGTACAACCAATATTTGTTATGATCTCGAGCCATCCTTATTTACTGTTGTTATGGTGATCCTTTGAATTGAGAAGTTTTGGGATAAGAAGTTTTTGGACCTGTTTTGCCCCTATGATCATGAGTTATATTCCAGCTTTTGATGCTATATATTTTTAAACCACTGGGTTATTATTATTTTTTTCATGTTGAGTTTTTGGTTGCATGCTGATCTGTTGGGTATCAATCCATTCTTTCCCAACTGAATGTCTCCATGAGACTGTATATCTGCTGAGCCCCAGATTTTTGGGGCTAAATCTTCGGTCATAAAAATATTGATGTTTTGATTTCATAGGATTTTTAAACGTTTATTCTTGAATGCCCTTTACAGGTTAGGTATGTTATGAGATGCGTGTTTGGTGACCCCAAGAAGGCTCCACCGCCGGTGGAGAGGCTTAGTCCTGAAGAAACTGTTTCTTTCTTGTGGAAAGGAGAGGGTTCACTTGTTGAGGAACTTATTCAGTGCATGGCTCCTCATGTGGAAGAAGATGTGCTAAATGATCTCAAGTCCAAGATTCAGGCTCATGATCCATCAGGTTCTGAGGACATTCAAAGAGAACTTCGTAAATCTTTATTATGGTAAGCAGAATGTACATGATTTATTTTCTACCAAGTGCTATGTTTCAACAGTTTCAACTTTGTTAAGAATGCAATCTTTGTTAATTTTCATTCTATATGTTTGAAGGCTGAGGGATGAGGTCCGAAATCTTCCATGTACATACAAGTGTCGGCATGATGCCGCAGCTGACTTGATCCATATTTATGCTTACACAAAGTGCTTCTTCAGAGTTCAGGCAAGTCTTCTAGTTAGAAATCACCGAAAATGCTTTATCATTCTAATTGGATGTTGCTATTCTATCAATCTAAATCATGTTTTACTCTGTACAGGAATACAAAGCTTTCACTTCACCACCAGTCTATATTAGCCCTCTTGACCTGGGTCCCAAGTACGCTGATAAGTTGGGGGCAGACTTGCAGGTGTATCGGAAGACATATGGTGAAAATTATTGTTTAGGGCAACTGATTTTCTGGCATATCCAGACAAATGCTGACCCAGATTGTACCCTGGCTAGGGCGAGCAGGGGTTGCTTGTCACTACCTGACATTGGTTCCTTTTATGCCAAGGTTCAAAAGCCATCACGACATCGTGTTTATGGCCCAAAGACTCTGAGGTTTATGCTGTCAAGGATGGTGAGTTTTCTAATATATTTTGTGGTTATGGGGTAGTCTTAAAGACTCAGATCTGAAGATTTCTAATCTATCATATTTGAATTACTTGTGCAGGAGAAGCAGCCTCAGAGACCATGGCCGAAGGACCGCATTTGGGCATTCAAAAGTTCTCCCAGAATATTTGGAAGCCCAATGCTAGATTCCAGTTTGACTGGTTGTCCATTGGATAGGGAAATGGTACATTGGCTGAAGCACAGACCTGCAATATTCCAGGCGATGTGGGATAGGTGAAAATTGATGTAGTTAGGGAAGCAATTATAGGGATTATAGGTAACGGTAACAACCCACCCTTTGTAAATGTTTTGGAATGGTTTTTTTCGATATTAGTTCGCAGCTGCCCCAATTTGAATGTGGGAGTTGGTAATCATTCCAAAACATCAGCGCTTGCGCTGGTCACTTCATTCTTGTGTACAGTTTAATCATCTTTGTATTTTTGTTTGTAATTCATTGTCTTGTAGTAGAGCATTTTTTTTTTTTTGCATTTTCTCTGTTTCTTTTATTATTTTGTCCCTGATTGAAGGAGGAAAAAAAATGAAAGGAAAACTTAAAAATTGTTTGAAGCCATTTCTGCCCTTTGATCTTACTTTCATCGCACTCAATCAAAGTTTTCAGATTTCTATCCTTGCACTCCATTCTACCTATTATTCCCTGGCTTT

>CsSDG27

CCATGGCAGTCTCAAAGATTGTACTATCGTCCCTAATCCACATCCGCCCGATAACGTGCGCTGCTTCTTACCATACACGGCTGGTACCTCACCCACCGGACTTGGTCAAGTGGGTCAAGAAAGAAGGCGGGTTCGTGCACCAAGCAGTCAAGATATTTCCTCAAGAAGATAATGAAACTTACGGTCTTGGATTGGTCGCCTCACAAGATATTCCAAAAGGGTCACTTCTCATTGCTCTTCCTGATCATATACCGTTGAAGTTTGAATCCGACGCTGGAGATGGGGCTGATTCTGTCTTAGTTGATTTGGCTTCCCAAGTTCCTGGTATGTGCTTTTCGGTTCTACTCCAACTGAAACACAACCGCACAACCACCACCCCAACCCTGCTGTACGGCCAAGGCGGATGTACGGTACAGCAGTACCNNNNNNNNNNNNNNNNNNNNNNNNNNNNNNNNNNNNNNNNNNNNNNNNNNNNNNNNNNNNNNNNNNNNNNNNNNNNNNNNNNNNNNNNNNNNNNNNNNNNNNNNNNNNNNNNNNNCCCACCAAATTTTTTTTTTTTTCTTGGAAGAGTAGATGCTTGACTGGATATATGGTGACATTGCTAATTGGGCTGTGTTTTGAGTTTATCTGAGCAGAGAAAATGGGTGAAGAAATGATTTATTTTGAATGTTCTTGTAGCTGTAGATAACTAGTGTTCATTTGAAGGAAATGGAAAATCTTTCTCCCTGAATGCAATTCAATGCAAAAGTTTTGATTTTGTAATGTCAAATAAGGTTATTCTAGGATTTTAAGCTCATTTTGTATATTTTCCATGTTATTCAAATGCTACTATTCCAATATTTTTCCTTGTTAATACAAACAATATTTAGGACTTTAAGTTACTAGTCTAGGACTTCATTGTGTGATAGACCACCTGAAAGATGCATAGACTCTGTTGCCAACTAAAAACGACCAGATTAGGTTCTTGTTACTATAATTTGGAAATACATGAAGGCATCTTAAGTCAACATTTTCTTATGGTGACTGGTCATGACATTATTATTTTATAATCTCACGGCAGTGTATTTTGATATGTCACGGCAAATTGGCAATGTATTAAGGAGTGAATTGGAAGGTGGTTGAGGAGTAGAGAGGAAAGAGGACATGAAAAACATTTCATTCGTATAGGCACGCTTAATTTTGGGAGTGCTTGAACTTGTGTCCAACTTTTCTTATGGTTTTAGGGAATGGGAAGAATACTATATATAGAAATTCTGTTTTATTCAGTTTGAGCATTACCATTAGTTGTGTTCATGGGGTATGTGTGGCTATAAAAAGTTTGTGTGAATCTTTGATCAAGTAAAATTCTGCTGTTGTGTGATGTCACATAAATATATTTTACTCAATATAGGCTAGTAACTTCAAGTTCTGTAAATTAGGCTGTTTAGAGTTTTGAATATGAGTAAACTGGACATTGTGGGAGCATTTGGCAGAGGAACTATGGGCGATGAAATTGGGTTTGAAGCTGCTGCAAGAAAGAGCGAGGTTGGGAAGCTTCTGGTGGCCGTATATTAGCAATCTTCCTGAAACTTACAGTGTGCCAATCTTCTTTCAAGGGGAGGATATTAAGAACTTGCAATATGCGCCACTTCTCTACCAGGTATACCATTTAACCAACTCAAAAGTGAATATGATCCACTAAACAAAAATGGGGGGTTTTGGGTTCAAACTTTTGAGGTTGTGCTGGAAATTTGGAAATTCCTTCTGCCTGCATAATTCTGACACTAAACTAAGAATAATTGTTATTGCAATTATTATTTCCTGTGCTCTAAGACCTCTGCAAATGGCTGATGTTATAATACTGTTGGTTGGCTCGTGAGCTGGCTAGGTGAAGGCAAAACATGTTCTTGTTTAGATTCTTCAACAGCCTTATATATACATGGCTTCAAAGGTAATAATGATCTGTTTAATTCTATTTTATAAAAACAGGTAAACAAACGTTGCCGTTTTCTTCTTGATTTTGATCAAGAGGTCAGACGTGCTCTTGCAAATGTGAAACCAAATGATCACCCTTTTGGAGGCCAGGAATTAAATGCATCATCTCTTGGATGGGCAATGTCAGCTGTCTCATCTCGGGCATTTCGTTTGCATGGGAAAAAGCTAGCAGCTGGGACACGCAATGAGGTCCCTGTGATGCTTCCTCTCATTGATATGTGCAACCATAGCTTCAATCCGAATGCCCAAATTATCCAGGAAGAAGATGATTTGCAACTTCTGATAAAGGCAGGTCCTAATCTTTTTCTTTTATTCTTTGACTCCTTTTATTTTCTGAAAAGAAGTTTCCCTTGTTTTCTTTGCTGTGTTAAGGTTAATGTCAATCTCTCTCTCTTTCTTTCCCTGAAGTCAGGTTGTTGCAGAGACAGAGATTAAACAAAATGATTCTTTACTTCTTAATTATGGGTGCCTAAGCAATGATTTCTTCTTACTGGATTATGGATTCGTAATGCCTTCAAATCCCTATGACACCATTGAGCTCAAGTATGAGGGAGCACTTATGGATGCTGCTAGTATGGCGGCTGGGGTGTCTTCACCAAACTTCTCTTCACCAGCTGCATGGCAGCAGCGAATTTTATCCCAGTTAAATTTGGTTGGAGAAACTGCAATTGTCAAGGTCTCAGCTTACTCTCATCTGGTTAATTAATTATTTATCTTTTTGAATTGCTTTCAACAAATTGAAGGACACACGCGTTTTTCAGGATGAGTTCCATGATGGTAGCTTTAGTTTCTATTTTGTGCCTTCCCAATTATGGCCAAGATACTTGTGTTATCTTTGGTTATTTATAAGAATTTAGCCTTGGTAAAAAATTTGTAATCCACCTCCTTATATTGCAGTTATAACTTCCATCTTGTTTTCAAGCCTTTTAGGTATCAGCCACCAGCAGCTTCTTAGAAGCCATGAATATTACTAGTGACCTGTTTAACTCTCAACAGGTTAGCTTAGGAGGTCCTGAATTAGTAGAGGGACGCTTGTTGGCAGCCTTAAGAGTACTACTTGCAAGTGACTCAGAAACCGTACAGAAGCATGATTTGAACACACTCCAATCTTTGTCAGCTGAAGCTCCTCTTGGAATCACAAATGAAGTTGCTGTTTTCCGCACGATTATTGCCCTATGTGCAATTGCACTAGAACATTTTCCGACAAAAATAATGGAAGATGAATCTTTACTGAAGCAGGGAGTTTCAGCTTCTACTGTTTTGGTCATCCAGTTCAGAATCCAGAAGAAAACTCTGATTATAGATATTATGAGAAATCTCTCGAGGCGGGTGAAATTACTATCATCAAAGGAGACAACCGCTACTCAAGGCTGATATTTGATGGCTGGAGGGTCATTACTCTGCTCATCATGTATGTTTTGATCTCCCATTATCCCCATGTTTTGTTATGCACCTTGAAAATTTTGACTGGTTTACTGGAAGGGACAGCCTCTGTGCAATGGATTTTTAGATTCCTTTTTCACAGTTGAACTTTTATTCAGCTTATCTTTTCATTGATATTTGGCAACATACTGATGGAAAATTTGCATTCCATCATGTTATTTATAGTTCTCCATGTGAATTACCCTGAATCGACTAAACAAATTGTACAATTTAAGTTTGCGAGTGAATTGTATAATTTGAGTTTGCAAGTGGATGCAAATTTATTCGTTTCGT

>CsSDG28

TTCATTTTCACTAATTAGGATTTCATGAATTTTTTTATGTGGCAAAAGTGGCAGAAAGGCCTCAAAGAACTCATTCAAATTTAATCCAATTCTAATTCGAATTATTTTGAAATATTAATGAGACCCGAATTCCAGTCCGATAATTACAGTCGGATTGACATTTTTTTTTTCCTTTCAACCCGCTTAAACGAAAAATCTAGCGGATTGACTAGTTGACCAGCCGTATTTATGCATTTTTCCTTGAGAAATTTGGTATAAAATGTAGTTTGTTTCATCCGCTTAATAATTCTTTATAATTTTTTCTTTAAAAAAAAAATTGAGTCATCCCCACTGAGCACCCAAAGAAGTAGAAAGCGAAACCCATAGGCGAAGAAAAACCCTAACCACACAAAATGTCGGCGGTGAGTGAGACGGTGACGGTTGCTGAGATAGAGGGAAGAGGGAGAGGTTTAGTATCCACACAATCACTAAAAGCAGGTCAAATAGTCCTCAGAGATTCTCCCATTCTCCTCTACTCTGCTCTTCCTTTCATCAATTCTTCTTCATTTTGTCACAACTGCTTCAGGAAAACAATGCATTCTTCATCGTCAATTTGCCCTTCTTGTTCAGTCGCCTTCTGCAGCCCCAAGTGCTCAACAGCCGCCGCGTCATCCTCCCACTCTCCTTATGTCTGCCAAGCTCTGACCCGTCTGCTCAATCTCAACTCCCCTGACGCCGCCAACTTGCCTTTGGATCGACAAGTCCAGGCCCGGTTCTTGATCGCCGCATATAATTTAGCCGTTGTTAATCCTTCCCAGTTTCAAAATTTGCTTGCTCTTCAAGGCACTGTTACTGATAACGATACATCGGCTGCTCATTACCTCTCCTCACTCTGCCCTCCTCCTGCTTCAGCTACTATTGAACTTACGGCTGCTCTTCTCACCAAGGACAGGCTCAATGCATTCGGTTTAATGGAGCCTTACATTGAAGGCCAAGATGGCCGGCGATCAGTTCGTGCTTATGGCATTTACCTCAAGGCTTCTTTCTTTAACCACGATTGCCTTCCAAATGCTTGTAGGTTTGATTATGTGGACGCTGCTGCTGAGAATAACACTGACATTATTGTTAGGATGATTCATGACGTTCCCCAAGGCAGGGAGATTTGCTTGAGTTATTTCCCTGTCAATTATGATTACTCCACTAGACAGAAGAGATTGCTATATGACTATGGCTTTGCCTGTGACTGTGATCGCTGCAAGGTGGAAGCTAACTGGTCTGACAATGACAATGATAATGAGAACAATGAAGAAGTTATGGATGAGGATCAGGATGAACAAATGGTGGCCTCTGATGATGATGCTGAAGTTCACGGTGACACTAATTTTCCTCATGCATATTTCTTTATGAGATACATGTGTGATAGAGATAATTGTTGGGGAACATTGGCTCCCTTACCTCCTTCCGATGCTACGCCTTCTACTGTTATGGAGTGTAATGTCTGTGGAAATCTAAAGAATGATGCCATTGGAAGAGAAGACACTGTTGGTATGGATGACTAGCTCAGATATCTGCACTTGCTGTAAGTCTTAAATTTCTTTCTTTCTGTCAGTGATGATATGATGTGTATTTCCACCTTGCGAAACTATTGAGCTATGTAGAACAAAATGGATATTTAGTTATAATTCAGCTTTTCTGTTAGTGATGATATGATGTGTATTTCCACCTTGCGAAAATATTGAGCTATGCAGAGCAAAATGGATATTTAGTTATAATTCAGCTTGTTCAGGTCCATGCCAGCTTGATCCTGTCTATAATGGACGGGTGGTACATTTGTTCACAGTTGGTTGTTGCATCATGTGATATGCAATTTTTAATGGATGTCTGTACAAGGATCACAATTTCTTTCATTTCAAGTGTGAGTAAAATACGAAGGGAGTTGTAGATAGGTGAAACCAGTAGATAGGATGAATACTAGCAAGAAACTACCATTTGAATAGACTGGGGCCAGATTGATGAAGATTGTCTTATGAGCCTTTGCTGCATTTGCCATTGATTACCCGAAGTTTTATGCTTGCAATTTTGTATTTGTATACCATTTTTCCTTTTTAATTGCAGGTGATGTTTCCAGTGGTATGTCGAATGTCACTGCTACATCATTTACCATATTCGATCAGAATATATATGGACAGATGCAGGCTTTCACAAACCAAGCAATTTCTGCTGGGCCTTTATCCTTTTTTTAAGCTCATTACGATTCTTAATGGTTAGAATTTCTAGTTATCTCGATGGGAATTAGCTTTCTTTATTTCTGCGGCAGCAAATAATTTTTTTTCAACAAAGCAATGGTGATGTCTTTCTATGGTATCATGGGTTAGTTCCTATTTGTCGTGCACAGTTACATGGAAATTGGAATGTAGGTAGGTTATAATCAATTTGATTAAAAAGAGGGAATACCCTTTGCATACGGCAGTTTCAGATTAAGCTTGACATGTCAGATGGAATTAAAAAACTGGGATATAATTAAAACATTAATAGTCACCTTGACTTGTTTGATTGGGTAAATGCTCTAAATATTTTTTGTATTCTATGAGTATACATATAGTTGTTAGTGTGTGTTTAAGTGGGTCTATTCAATTTCCTTTTGTACTTTTCCCAGAGATTTTTGTCATTATCTCAAAGCTGCAGATTTTGTTGTATGATTTGGTACTTCTAAATTATTTGTAACTTTTGCTTTCTGTCTATTTATGTTTATTAGCCCATTGGTTGTTCTGGTTGCAGGTTTCTGGTGAGGACAGTGCTGCTTCTTTTCTTCTTCATCCTCCATAGTTTTTTTTTTTTATATATTTAACTTAGGTCACACATTTACAACATAAGTTATTGTGGGTGCTTGTTGATTCCTTTGGACGGATTTTGTTTCTCAGTTGTTGCAAAAAGACTACTTGGTTATCCAACATGTGTTTTTTTTTTTTTTTAAATTACCTCTCAGAATTTTTCTCCCATTAGAGAGAAAGTGCCAACTAACTCAACTAAAAGATG

>CsSDG29

ATGGCCGCACTCTCGAATTTCTCATTCTCATCATCTACCAGTCCTCCGACATTCGTTTCCTCCTCAAAAACCCTAAAGCTTCTTACTTCCCTTACCAAAAAACCATCTTTTCGTCTAAAAAGCAGAGCTTTTGCAGCAACTTGTTCACTTCATTCAGCATCAGCAACAACTAACCCACCAACAGCGCAAGTCGAAACCTTCTGGCAATGGCTCCGCGACCAGAAAGTGGTCTCCCCAAAGTCACCCATAAGGCCAGCTACATTCCCAGAAGGACTGGGGTTGGTTGCACAGAGAGACATTGCTAAAAACGAGGTCGTTTTGGAGGTTCCCATGAAGTTCTGGATAAACCCAGATACGGTTGCAGCTTCAGAGATTGGAAGCTTGTGTAGCGGATTGAAGCCTTGGATTTCAGTGGCTCTGTTCTTGATCAGAGAAAAAAAGAAGGAGGATTCTCCTTGGCGGGTTTATCTTGATATTCTTCCCGAGTGTACGGACTCTACTGTGTTTTGGTGGGTGTCTCTTTGCTTTTTCTTTTCTACTTTCTCTTTGTTTGTTCTATTCTGTTTGGGTTTGTTTTGGTTTTTTGTCTTTGAGGTTGATCAAGTTATATGTTAATGGCTTCAGTGCAGGTCTGAAGAGGAGCTTGTTGAGCTTCAAGGTTAGTGAGGGAGGCTTTAGAAATATTTGATTTATAGATTGAAGTAAATCAGTTAACTGTGAACTATTCTCTGTTGCTGTATATGTGAAGCATATGTTTTGGTAATTAGAAGCTGCTAGTGAGTGTAGTTTGAAAAACTTATTTATTTAGTGATATAAAGAAACTGAGCTTTTGTTTTTTGAATTAGTTACGTAGCTGATCTTTCTGTCACTGCCACTTTCTATCACTACCAAATGTGGCTAGAGCAAGTGAAGCGCATGATTGAGTCAAATTAAGTCTATATAATCATTGTTTCTTTTATAGTTGAGATTTTGGTTTATGTGATGCAGGGACTCAACTATTAAGCACAACTTTAGGTGTGAAAGAATATGTCCAAAACCAATACCTAAAAGTCGAAGAGGAAATCATACTCCCTAACAAGCAACTATTTCCTCGCCCTATCACATTGGATGACTTCTTGTGGGCCTTTGGGATTCTAAGATCGAGAGCATTTTCACGTCTTCGTGGTCAAAATCTGGTTTTGATCCCCCTTGCAGATTTGGTAAGGCTTTTTGCTTTTGCCTTGAAGTGGTTTTCCATAGGAAGAAACTAAAATATGCGGGCAATGAATTTTCAACTATGTTTTGGAGGGCCTCTTGTTTCTTTCAGTTTCAAAATTTAACTTTCTACGACCTTGGATTATCAATCAGATAAACCATAGCCCTGGCATAACCACAGAAGATTATGCATACGAGATTAAAGGAGCAGGTCTTTTCTCAAGGGATCTTCTATTTTCTTTGCGGACTCCTGTTCCTGTTAAGGCTGGTGAGCAGGTGAAGCTTTCCATCTTTTAACATTTATTATTTTATTACTTCAGCTACTGTAGTTCCAAAGTTCCTTCCAAAAACTATATTTGATGTTGAGAAAATCATCGGATCATCAGCAACCCTACTAATACAATTTTTTTTTTCAAAAAAATTTACTTTTTGGCTCTCTTTATGTTGCCGTTGTAAAACTGAATTTGAGTTTTACATTAGACAAAAAATTCTCTTATAAGTCGTGAGCTTCAGAGGATTTTTATTTACACCAAATTTTAACAACTGCTGACTTGGACTACCATTGATAGGTTCTGATCCAATATGATTTAAATAAGAGCAACGCTGAATTGGCTCTAGACTATGGCTTCATCGAATCAAAGTCCGACCGCAATGCATATACCTTAACACTAGAGATTTCTGAATCAGATCCATTTTTCGGTGACAAACTCGACATTGCTGAGACAAATGGTTTGGGAGAGTCTGCATACTTTGATATCGTCTTGGGAAGAACTCTTCCACCAGCAATGCTTCAGTATCTGCGTTTGGTAGCACTTGGGGGAACTGATGCTTTCCTTTTGGAATCAATTTTTAGAAATACCATCTGGGGTCACCTTGATTTGCCTGTAAGTCGTGCAAATGAAGAACTCATATGCCGTGTGGTAAGAGATGCCTGCAAATCTGCTCTATCTGGCTTTCATACTACCATTGAAGAGGTAAATGTTGGTCCTTTATGTGCATGACTAATAGAAGTTAGTTCTCATGCAGTGATTTTAATTTACCATTCATGCATGTACTACAACACAGTTGATGAGATGCAGTTTGAACTTGTTTATATCTTTTCCAACGCTCCTTTTGTGCCGGTATCAAATAAACTTGTCTTGCAGGATGAGCAGTTGTTACAAGGAGGAAATCTTGATCCAAGATTGAGAATTGCAGTTGGAGTAAGAGCAGGAGAAAAGAGGGTGCTGCAGCAAATTAATGAGATTTTTAAGGAAAGGGAATTGGAATTGGATGAGTTGGAATATTACCAAGAAAGGAGACTTAAGGACCTTGGTCTGGTTGGAGAACAAGGTGACATAATCTTCTGGGAGCCAAAGTAAGATTCGACAAATGGGTTCCAAATGGCTCAGGCCTCAAGGGCTTGCGCTCAATGTATTGTTATCTAAGCTACCAGAATTGACCCTTTTTTTTCTCCTTTTTTTCTGGAAAGATTCTTCTCCATTTTACTTTTACGCTGGATCCAGCAAATAAAGCATATTTGATCATTCGGGAAGGAATTCTGATGCAGAGCTTTCATTCCAACTTGTAGTGCTCTTGAGGGAAAAGTAAATAGGATAGTTTGCAGGTGTTTTAACAACAGTATCTGCTCGGGGGGCCGAAAAAAAAAAACAACAGTATCGGCATTTCTATAGCCTGAATAATTGCACCCTTTTATTT

>CsSDG30

ATGGAAATGGAAATGCGTGCGAGTGAGGAAATAAGGCAAGGTGAAGACATAACGCCACCATTATTTCCTCTCACTTTCGCATTTCACGACTCTTTACTCGACGGTCACTGTTCGTCTTGCTTCTCTCCACTTCCTTGTTGCTGCTCTTCCCTCCCCCTCTCCTCCGCCGAACTCCGCGCCGCTCTCTACCTCCTCCACTCCCCTCTTCCCACTTCCTCTCTTCCTCCTCCTCCTCGTCTCTTCGGCTTACTCACCAACCGCGATAAGCTAATGTCTTCCTCCGACTCCGATGTTGCGTCCAAAATTCGAGAAGGAGCGAGGGAGATGGCGAGAGCGAGAGGGAATTTGTCCGACGACGTCGCTTGGGAGGAGGCTGCGTTGTGTTTGGTGATGACCAATGCCGTTGAGGTGCAGGATGATAAGACAGGGCGTATTTTAGGTATCGCCGTGTACGATAAGGATTTCTCTTGGATCAATCACAGTTGTTCTCCCAACGCTTGTTACCGCTTCTCGCTTTCTGAGCCAAATGCTCCGTCGTTTCGCAATGAAAAGAAAATGCGGATTGCTCCTCACGTTGTTTTCGACAGCACTGAGGCTGAAACTCCAGTAAAATCCCAGCTATGTTGAAATTTTTTTGAATTTTTTAACTGACTTGAGCAAGTCGATAATTGATTTGATGCAGGGAAAGAGTGATGTTTGTATCAGTTGTGAATTAAAAGAAGGTTGCTAATGCTAATTGTTGTTCATTTCTATATAAATTTTGCACATTGTTTGATTTTTGCCGTTCTGAATTTCATTTAATTTTATTTTCAAGGAAGTAAGAGACATGGTCCAAGGATCATTGTAAGGAGTATTAAGCCAATTAACAAAGGCGAGGAGGTAACTGTTGCTTACACTGATTTGTTGCAACCCAAGGTACTACATTCCATTTAAAATCAGCTGATCAGGCATGTGTTTTATTTGATAATTTTGCATAATTCTAATTGTTCAAACCAATTTTATTTTTGTTCAGGGAATGAGGCAATCAGAGTTGTGGTCAAAGTATCAATTTGTCTGTCACTGTAGGCGATGCTCTGCATCACCCCCAAGTTATGTAGATATGGCCTTAGAGGTAAAAACTTTATGTAAATTGTCCGCGGGAAGATCTCAGTTGCAAACAAGTTGGGCGACAAGTATTGAACTCTCGAGGGCTTTTTTTTGTTCTTTCCCCAGGAAACCTTTTCTTCAAATCCCGAGTTTTTGAGCTTGAGTTCTGATTACAACTTCCTCAAGGACGAGGCAAATCAAAAGTTGACTGATTGGATGGATGAAGGTACATCTGAATATCTATTAGTTGGTGATCCTGAATCTTGCTGCCAGAAGCTGGAGAACATCCTTACCCAAGGTCTGCAGGGTGAGCTTTTAGAATCCGAAAAAGTGAAAATACAGCTGAATTTGAGGTTGCATCCCCTGCACCATCTCTCACTGAATGCCTATACAACACTGGCATCTGCATATAAAATCCGTTCAATTGACTTATTAGCTCTCAATTCTGACATTGACGGACAGCAATTGGAAGCTTTTGATATGAGCAGGACAAGTGCCGCATACTCATTACTGCTTGCTAGTACGACTGACCATTTGTTCCGATCTGAGTCATCTCTGATTGCAGCGTCTGCAAATTTCTGGGCAAGTGCAGGGGAGTCCTTGTTAACTCTTGCTAGAAGCCCAGGGTGGAATTTATTTGTGAAACCGGAATTGCCTATTTCAACCTCGTCCCCTGAGATTCATGAGTGCTCCAAGTGCTCACTAGTGGACAGATTGCAAGTGAATCCATTTCTTAGTCAATCTCGAAATGCCGATTTCCAGATCATATGTAATGAGTTTCTTGCTTGCATCACTAATATGACACGAAAAGTTTGGGGTTTTCTTACACATGGATGTGGGTACCTGCAAATGTTGAAAGATCCCATTGATTTCAGCTGGCTTCGGCAGTCTTCCAATTTGTGCCACACTCCATGCTGCAGTGATGAGGAATCCAATAAGGAAACCGGGTATCAAGAAAGTATTTGCAGAAGGGTAATGCAGCGGTGTGATGGTGAAGAAAGGATTACTATCTTTCAGCTTGGTGTTCATTGTATAGCTTATGGAGGATATTTAGCAAATATATGCTATGGTCCGAATTCCCATTGGCCTTGTAAAATTAAAAATGTTGTACAAAACGAAGAGAAATTGGTTCATTGTTGA

>CsSDG31

CGACAGCGTAATACCTGCGAAGATGCGCACCTCAAATTCCGTGCGCGCAGTCGGCGCACGTTAGCTTCGGCCCCCTCTTTGCGCCTCTCTCTTTTCCCCGCGAGACACCAAGGCAAAAAATCTCCTCCATTTCCTGCAAAACATACAAAGTTTTCAGCTCTGTTACAAAAATCCATCAGTTTTTTCAAAATTTAATTTCTGCCCTCGATAATCATCATAAATTTCAAATTAGTCCTTCCTCATTTTTTCGTACAATGGCTCCGGCCACCACCTCATCGGCGGAGGCTAGGCGGCTCATCGGCTCGCGCCGGAGAACGGAGGCGCCGCGACGAATGCTGTCTCCGTCTCCGCCGCCGAAGAAGGTGAAGTCGATGGAGGAGATACTTGCGAAGGCGCACTACGCGGTGGTGGAACGAGGCGACTACGGCGACGTCGGCTGCGAGCAATGCGGCTCCGGCGAGCGAGCCGAGGAGCTGTTGTTGTGTGATAAATGTGATAAAGGCTTCCATATGAAATGCCTCAGACCGATCGTCGTTAGAGTGCCCATTGGAACCTGGTTATGCCCCAAGTGCTCTGGCCAGAGACGTGTAAGAAGTAGGGGATTTTTCTTTCCGTTTTTGTTATTTTTTATATTTTATTTGTTGTTTTTAAATTATCTAACGTAAAATGTGGCAAAATAACTTGTGTTTTTATGCGTGTTTCAGGTTTTTCACAGAGGAAGATAATTGATTTTTTCAAAATTAAGAAGCCTAATCTGCCAGAAGAGAAATGTGACTCTCCTCAAGGTGCAATTTTGATTATTCAAGAACGTACATCTCTGTCTGTCCTATTAAAAAAAATTTAAAAACAATAACAAGGGTAAAATTTATCATGAACACGAGAACCAAATTAAAATTTTGAATGAAGGTTTGTGAAGATTATGTAATTAATTCTATGGGATTTAAAGGTGTGACCTACATGTTAATCAAGGTTTATCAAGTGGAGTCATGAGTTTAGTAATGGAATCACAAGATGTCGGCTTACTAATAATGACACAATGCAGGAATGTATTTATTAACTTGTTGAACAATGTAGAGTTGGGATCAATCAATAAATTATATTACATATTCAACCCCCTATAGAAACTATTCTCGCACAAACTGAGATGGCAATCATAAATACTGTTACATTAGTTCGCCGAAAATTTCTACTCTAGGAGTTTTCACATGCAGTATTGTACTTAATGAACTTTTATTATTATAAAAATTGATTCCATTTGGAATGCTTCATCCTTTTCATTTGATTACATTTTGGTCAAATACTTTGTTTCCAGCATTCACCTCAAGGAAGAGGAAAGACGAATAAGACTCATTATCGTCAACATTTGAGTTGTCTCAGCTAAAACAATGAGATAAAGAACCCAAAGCCCCATTGATCCCAAGTATATTCTGGATTACCACAAAATCAACTGAAGCCTAATTCATTCAATATAAAACCAAGAATTGAACAAAATAACTGAAGTGCAAATATGAATCTGCAGAAAACTAAATCCTAAGATAGAAAGTTTTAGATTAACTTCATCCATGTAGTTGATCCTACTTTTGGTCAGATACATGGTCGCATCAGCTAGGTTCTTGACAAATTTGAAAATGGTAAGCTTAATGAATTAATGGAACTGTATGATCTGTTTAAACTTATTGTAAAAGCAATTAGTTTAGATTTTAATACAAGTGAAGTTTTAGGATAACCATTTAGATAAAGGAATACGAATGTTGAATGGTCATCTAATAGTGAGCAAAAAGATTACTGCCACAGTGGATATATGCAAACACGAATGCTTTAAAATGTGCATCAGTAATTCTGCTTAGCAAATGATGAACTTGTCACTTCCTAACTGATAGTCATGCTCCCGTGATTCCACTTGAGGTAGCTAGGTATTCTCTAAGTTATGACAAGTGAAGGCCAGCAGGATGTTATAGTAATCATGCAATGAACATTATGAACAGCAATCTTGTTACAATAAATAAAAATATTCTGTGAGTTCAGACTTTAAAGAGCCTTTCTTTTTCATTCACTAGAATTGGAAGGCAGTGGTTGCCTGTCACAGAATATAAGTAAATAAAATTATGGGCTTAGATTGCTTCTCCTTTGCATTCGATGAAATGATTATCTCTAATTGGTGTTTTCTATTTGTTACTTCTGGCTCAATCCTCAGATACTAGAAAGCGTCGAAGACGTTCTGCATCATTGGTGTTGCAAAAGAAGAGAAGGAGATTACTGCCTTTTACACCATCAGAAGATCGTTCCCAAAGACTAAGTCAAATGGGCTCTCTTGCTCATGCTTTAACAGCATTGCAAATGGAATTCAGTGATGATTTAACTTATATGCCCGGCATGGCTCCTAGATCTGCTAACCAGGCTGAATTTGAAGAAGGTGGCATGCAGGTTTGCTGTTGTAACTATACTTTCCTATTTGCTTTCCCTGAAGAACTTGTAGGCATTGATATTGCACATTAGTTAAGTATATTGTTCTATTTATCAGGTTCTTTCCAAAGAAGATACGGAGACGTTGGAACAGTGTAGAGCAATGTGTAAAAGAGGCGAATGCCCTCCCCTTGTAGTAGTTTATGATTCATGTGAAGGGTATTTTTGTCTTTAACTAATTTATAGTGCTAGCATTTGTATTTGATAATAGTACATTACGTAAAATGATGTTCATTTTGGGATGCAGTTTTACCGTAGAGGCAGATGGCCAGATAAAGGATATGACATTTATTGCAGAATACATCGGCGATGTGGATTTCATTAGAAATCGGGAACATGATGATTGTGACAGTATGATGACCCTTCTTCTGGCAACAGACCCATCTAAAAGTCTTGTCATATGTCCTGATAAACGTGGAAACATTGCTCGCTTTATCAATGGCATAAACAATTATACTCTGTAAGATTGTGTAACTCATCTCATTGTTTGTCCGTATTCTGACTATAGCAAAGATATATATATACTTGTTTAGTTTATATACTTATACAAACTTGTTCCAGGCCATGAATTCTGTTGTTATCCCTCAACAAGTTGACTAGTGTAGCTCCAATGTACTCAGTACAATTCTTTGTTTCTTTTTGTAGTTTGGATTGACAGTATACTTAAGTCCAGGGATCTTAAAACATCTCCAATTTTGTGCAGGGAAGGCAGGAAGAAGCAGAACTGTAAGTGCGTGAGATACAGTGTAAATGGTGAATGCAGGGTCTTTTTGGTTGCTACTCGTGATATTGCTAAGGGAGAGAGGCTATACTATGATTATAATGGATATGAGCAGGAATATCCGACTCATCATTTTGTCTGAGAAATCATAACGATATCACTCTAACAATCATACAGAATTGAGATGCAATTTTGGAACCAAAATAGATCTCTTTCTTTCTAGCATTCTTTTTGGCAGATTACTTATTATTGTTGCATGGTCAACATTTATTCAAAATTGTACCCATGGGTCATTTAATGTAATTTATGATTTAGGGCTGGCCCATTCTTTGGTAATGAGGGAATAGAGCAGGCGAAAGAAAATCTTTTTGGGAGGTTTGCATGTTAAGAAATATAGAAGATGATGTTTAGTACAGAAACATTTTTTTGCAAGTCGATTTAATGGAAATCTTTACCTTTCATCTGATTTCTGTCAGTCTTGGCATTGATGATGATTTATTATCTTTTTTATCTAATATTTCATCTTTCCATAATTCTTCTTCAGTCCAAAGAAATTTTATATTCCACCCATCCTCTTATGATGCTTACCCACCATTTGAGGGAGATGAAAAAAAGTGAAATACAAAATATATATATATGTTGTCAGTTCAGTTTTGATGCTGTATTTTCTTGCAGTCGCACAATTATTTTGATGCAGTTTGACCCTTCATATGATGCCTCAGAATCGGATTGAGCACTTTGTTGCAGAGTTCTCCAGAGATGTAAATTTCTTCTTCGCTTCATCCCAAGGATTTTACAAGTCAAGGATTCTTATCATGACATATACAACAAGGTGGTACCTTTCTCCTCTTTTTTAATTTGTACCATTGCATAGGTACAAAAATTTTCTTCATCATGGGATGTCCAAATTAGGAACACTTGCTGAGTTGATTTTCTGAAATCTTTTGTATCAATTGATTGTTCTCACTCTCACTCATACTAGCAGAGTTGCATGTGAATCATATATAAAATAACTTCTATTTATTTATAAACCTTTCTGACTGCATCAGGAAGAAACGTTTTAAAATATGCATCTTCTGTCTTTCAACCTCTTGTTTAGTTTCAATACCTTTTCTGAATCATATTATCGTTTAGATTATCAATTGGCTTTTCTTATGTTAATACGACTTTCAGATCCTCGGAGGTACTGCTATGCCCTGTGTATTTTCTGAAAGGCTCTGTGACATTATTGTGTTTGCGATTTGTTTAATTTTGGCAACTGCATCTAATATTTTATCATTACTCTAATTGCTGTTGTGTACTATATATATCTCTCTTTATATTTCGATCATAATACTCTTACTACTATTTAGCTTCCATATGAATCAAGTGGATTTCATCTAGACTAGACAACTCAATATTCGCTCGCTATTTTATAGGTGCATGAGCTTTCATTTTTATGATTTTATTATTGGTCTTCCTTCTGTTAAATCCACTGAATCCTCGTGCATTTTGTCCATAATCAGGTCTGGACAGATTCAAACTCAAGGCTCTCAAGGATCAGAGACATTGCAAATCACTTCATGCTACAGATGTTTTATTTGCCTCGTACATATTGATGTACTATGTTCGTCATATCAGTTTGATGAAAGCGTTGACGAAGAAGTCTGTGGGAGTAGTTTATGGTGTCTGTATATAGCATGGATGGTTTATTGTCGAGTTGAGTTGATTTTAATTGTTTTTGAAGCTGCCTCAATATTGGAGTAGACAAGAGCTTTTACGAAATGTATTGTTCATTTCATTTCAATTTGTTCAATTTGCATCTTTTACATTAGGGAAATTATCAC

>CsSDG32

CTTCAAGCCCCGGGCCTAGCCTGGCAGTTCTGCCATCTCCCTGAACTTAAAACCCTACAAGAAACAGAACAGAAACTGCTCCCGCCGGCTGAACTCAATCATGGCTTCCAGGTAAAACCTGAACACTGTCACATTTTTAAGAAATATGTATTTGTGTCTACTGAAAGTTTTTGTTTTTTTTAAAAAAATTGTATCATGCTTTTATGTGGTATGCTCAGGCGAGTAAGAGCGTTCAAGCGTTGGATGAGATCACAAGGCTTGGAATACAGCGACGTACTCGATTTCAAAGATGACCCGGAACAGGGAATTTCTGTTATAGCATTAGGTGATTTGAAGGAAGGCGATTTGGTGGCTACTATACCAAAGAGCGCATGCCTCACTGTTAAGACCAGTGGGGCAAGCGACATTATCGAGTCAGCTGGTTTGGGGGGTAGTTTAGGCTTGGCATTTGCTCTTATGTATGAAAAGAGCTTAGGTGAGGATTCTCCCTGGGCCGGGTATCTCCAGCTCTTGCCTCATCAAGAGTGCGTTCCTTGTGTTTGGTCACTTGAAGAAGTGGATTCTCTTCTTTCCGGCACTGAGCTTCACGAGGTAGTTTAATTAATGTCTGATGCCTTTGTTAAATTGTGGAAATTGAAATGGCTGCTTTATAATATAATATAAGGGGTTTTGAGCATTTCTTGCTTTTTCTGTAGGCAGATAGTGAAAGAAGACAGAGGTCTTATTTTTGAGGACTGGAAACAAAATATTTTACCCCTTCTGGATTTGAATTCAGCTAATGTGAAGCTTAATCCAGACTATTTTGGTGTTGAACATTACTTTGCTGCAAAAAGCCTTGTTGCTTCTCGATCTTTCCAAATTGATGATTTCCATGGATCTGGGATGGTCCCTTTGGCAGACCTGTAAGTATAAATTCTCTCTTGGCATTGGTTTCTCTCAGAATTTCATCTGTGTTATTGGTTCAGATATTTTTGGGTCAATGGTCAGTTAACAATGAATTGATAGCTTATACACTGAATATGTAGATGTTGACATCGTTTCTGATAGTAATGGTAGTGCAGACCTTAAAATAACTGATTTGAAACTCTTTTGCAAAGGTGTCAGCTTGCCAAGTGTCTCACCGTGCAGATCAACAAGATAGGTTTCAGTTCTATGTGGTGTCACAGGTTCATCAGTTTCATCTATTTTAGCTTAATCTCATATTCAAAATAGACATATTCTTGTAGGCCTTCTTGTCTTCTCTAGGACCTGATTATATCTTCTTCTTTCTCCTCATTATTCCTGGTTGTCATAGCAATTTGTTTTAAGTTGCCAATCTTGGTCCATTTTTATCTTGTCTTCAAAATTCCATGCATTTACCAGTCCTTTTATTTAACCATATCTATTAATTTATAATGGTCTTGAAGTTTCTCTTCCAATAGTTTGAAACTACTGAACATTACACTTTGCTGCTTATGCTTACCCTGAATTTTACCCATTCTTTCTCGATGTATTAACTCTTCTTATCTGACACATCCTCTGTTATGCAGCTTTAACCATAAAACTGGGGCCGAGGATGTACACTTCACCTCTGTATCTTCACATTGTCAATCTGACAGTGATGCTGACAATGATGATCATGTGGTTGCCAATTCTGATGATAATGAACCATCAACTGAACATCCTCACGGGGATGGAGAAGAATTTTCTGCTCCTTCCAATGGGAAGAGTCCTTTGGGTGGAAGTGACTTGGAATCTTCGGCTTTGGAGGATGTCCCAACAGTGTTGCAAATGATCATGGTGAAAGATGTCAGAGCTGGAGCTGAGGTTAGTGTGTGCTATTTTACAGGTATCCTAGCATTGTTTTCTTCATCCTTGTTATTGGTTGTCCTAGTCAAAAGGATTGAAAGATGATGTACATTTGATTGATTTCTTTGGATTTATTACTTCAACATGCTTTCATTTGTTTGAGTTGTGCATTATTCATTGTCTTTTGTTTTCAATTTGCAAAGTGGACAAGGATGGCTAGAATGCTAAGAAGGTGGAGTTCTTTTTTCTTTTTTTTTTCAGTTTCTATTCGTTCTTATAAATGTATTCTTCTCTTAGGTCTTCAATACATATGGGTTGATAGGAAATGCTGCACTGCTCCACAGATATGGGTTCACAGAGCCGGACAATCAATTTGACATTGTGAACATTGATATGGAACTGGTGCTTAAATGGAGTTCATCTTTATTTTCTAGCCGGTATATTAGAGCAAGGCTATCCTTGTGGAGAAAATTAGGTTACTCTGCATGTCAAAGCCAGAACTCTGAATATTTTGAGTTAACTTCTGATGGAATGCCCCAAATTGAGCTGCTTATATTGTTATACATCATGTTACTAACTGAAAATGTGTATCATAAGCTGGATCTTAAGTTATCGACTATGGAGAGTTACGATGAAGCCTTATGCATAATTTTGTCTGAGAAAAACAATATTCAATTGGGAAAAGGTTCGGAGATGAGCGAAGAACAATTGCTAACTGAAAGTGTTTGCAATGCCATCTCTTCACTTGCAGACATGCGGGAGAGTTTATATCCCTCAAATTCAAAAGAAGATGATATTGAACTCTTGAGAAGTTGTTCAATACGGGATAGGAAGCTGTACCATTCTTTGTCGCTGCGTGTAAGTGAGAGAAAGATTCTAGAGAAGTTGAGAACTTATGCAGCTACTCATATGCTGTCAATTAGAAATGCTAAGAGAGGCCCTACTGGAAAGAGATTGAAGAGGAACGGGCTGCAACATTCTTTCCTCTAATTTTTGCTTCTCCGTACAGGTTTCCCTTTTCTCTTTACAATTTTGTGAGGAAAATCACATGTTCGGAGCCCCTTTTTGCCACATAATTTTACCCAGTTGTATCAGCAACCCCAAAATGTACCGCAAGGTAATCGATATGTTGTTGAATCCACCACTGAATTTTATTTTATTTTTTTAAATGATGACAATTACTGTAGCGACGCCGTCGTTATATTTTCATATGCCTCATTTTTGTAGCTGACCCTATTTAGTTGAATTTTATTCGTATTAAATTTCTACGC

>CsSDG33

CTTGAATTTGAGTTCTTCAATGGCGGATGTTGAGGTAAAGCCAGACCCTTCACGTTGTAACTTTATTTATTTATTTGTTTTTTCAATTAGTCATGCTAAAACTGATTGATTATTACCTTTACTTTTGCTTTTATTTTTTTGGTTTTGCTCCACCAGTTCTTAGCAGGCAAGCTGACCCTTTTTGTAGCCATTCTTTGTAAAGCTTGAATTTTCGGGTTTATTTTTTTAGCTTTATAGAGCTGCCTGCGGTTTTTGTTTGAAGTAACGTGAATTTATTTCAATAAATTGCAGGCATGTTCATCAAGGGACTCTTTGGAGAATGGTGATGGATGTTCATTGGTTCTTGAGCTCACTGAAGATGATCCTTTATTCCATAAAAAGAAGGTGACAGATGCATTGGATAATTGACAAAAAGTTTAAAGTTTTTATTTTTATTTTTCTAAAAGAAATGTTGATTCATGGATTATTTTAGTCTAGTACATTGATTGTAGCTTTCAAAAGAAGTTCTCTAACTTGATAAGAGTTGCTAAGATGCTAAGTTCTAATTATATTTCTCTTTCTTTTCGACAGAAATTACTAAATGATAAGGGTTTCGGTGTCAAGGAACAAATACACCTGCAGAGCTCGATGTGTCCTGATTCAATAAGTAACAGTTTGGAAACAATGCTTAGGATAGGAAGAATTATCCACTTAGATGAGGTAAATTGCAACTCAAAAATGCATATTTTTTACACTTGAGACATGTTGTAAAGCTGTTCACAGGGAATCTGTTGAACGAAATATCCGGTACATAACATGTCCTCTTTCTATTAAGTGAGCTCATGGTGGGTGCAGGTGGAACTTTATTTTGGTGAATTTGACACACAAATGGGATTTTACAGCCCTAGGAATGAGTTGGAAACCCTCAATTCGATCCTTGCGCGTGTTAACACCTTACTCTCTCGTCAAATGCACAAGAAAATGAATGTCTGGCAAGGATTACAAGCTGCAATTATTCTTAAGATCCAGGAGTTTGGGAACCAGATCAAAGAGGTCGCCAGAATTGATAAAAACTATACTTGTGAAAAGGAAAAATGTCTAGTGCAATGGGGTGAAAGCAATGGTGTTAAAACAAAACTGGAGATTGCATGTAAGTTTTCAAGGTTAAGATGGCATCTTCGATGTTCACTTATCTAGTCTACTTTTTATGATGGCTTTATTTGACATTGAAGCATTCAAGTTTGGCTTTTAATATATTATGTTATTCATGCTGCATCATCATATAGATGTAGGCGGAGCCGGTAGAGGAGTTGTTGCGATGGAAGATTTGAGAGTTGGAGATATTGCTTTGGAGATCCCTGTATCTATTATCCTTTCTAAGGATCTTGTCCACAAATCTGACATGGTACTTTTTCATAATTTAAAAACTTTCAATGATTTTATGATGTCACCTGCATTAGGTTACACCGACTGTTACGGTTCTAAGAAAAATGTTCCGGAAGACTCAAAGGAGAAAGTAGTAATCATCCTGCCATTTAAAAGAACTTTGTCAGATTGGTGCTATACTGAGACAACAAATTGCCTTTCTCTTGAGCTTCAACTTCTATGTTTCTATCCTCAGTATGAAAACCTATTTGGCTACGAGAATAGGAATTGAACCCACATGTTGTAGATTCATGATCTACATCCACTACCTGTGATTATGCTAGCAGCATCTGCCCTATACCATTCAGAAAATTGAAAAAAAAAATATTCAAGGATTTCAAATGCTACAACTGGTCATCACCTTATGAAGATAATGAAGGGATTAAAATATAAGAGGTAGAACATTCTTTCCAATAGAAGCTATACAGGATTTAACCAAAGAGAGGGGAGAGATGTCTGTGCAGTATGTATAAGGCTCATGGTTTTAGGTGCCTGAGCAGTTGACATATGAAAATTACAATTTATTAGATAATACGCTGTTGTAAGCTAACTATCACTGAATTTAGAGCCATTTGCAGACTCATACTGAGTCTAAAAATTTTGGTCCAGTTTAGTGTTGTTGAGGACTTTTAGACTTTATTTGACTGCAGGATAATGTCTTGGGGAAGATTGAAGGAATGTCCTCTGAGACTATGTTGTTGCTCTGGAGCATGAAAGAGAAGCACAATTGTGGTTCAAAATTCAAGAATTATTTCGACAGTCTGCCAAAAGAATTTCATACAGGTATGCTTGCTTTGACATACTTTTCTGGGCTAGTTTACGTGTAGGTTCTTGCAACTACCATTGGAGGTGTAACTGCATTATGGCTTATAAGATTTTCCTTTTGCATGTGGTTGTGTGTGCGGGAAAATAAATATTTCCATTGTAAAATTTCTAACACTATTCTTTCTGCTTGGTTCCTTTTAATTGCTGCAGGTTTGAGCTTTGGAGTTGATGCAATTATGGCTCTTGAAGGAACCTTGCTGTTAGAAGAGATAATGCAAGCAAAAGAGGTTATTTCAGCTGGCCATGGATTATTGATAAATTTAACCTTTGTGCTTTTCTTATTTCAAGAAAAATTTGCTTGTCAGAAAAATGTTGATCCCGAAGGAGATGAGTTTCGCATCTTTAAGTTTAGAATGGTGATCTGGTAATCTGCAACTGCTCGTTAAATTTTCCTGAATCTCAGTGGAGTTCAACATATAGCCTAGAGTCCTATATCAATTCTTACTATTGTGCCAGATCTTGACATGTATAATAATTTGGATCAATTAACTAAAATCAATTCCTTGTATCCCTTTGGCAATATTTTCTCATTTTTTCCCAAATCTGAACTTTTTGGGAAATAAAATAAAATATAAAAAACTGAATTGTTCTGGTTGAAATATTTCATGTTTTGCTTGCTTGCTTACATATTTCTATATTGCATGAAGAAAACTACTTCCTGACCACTATTTCATCTTACCCTTTTGCAGCACTTGCGCACTCAATATGATGAGTTGTTTCCTCGACTATGTAATGATTATCCTGATATATTTCCTCCAAAGTTTTACACGTGGGAGCAGTTTTTATGGGCTTGTGAACTCTTTTACTCAAATAGCATGAAAATCATATTTGCTGATGGAAAGCTGAGGACTTGTTTGATTCCTATAGCTGGCTTTCTTAACCACTCGGTATTTCATTCCTCCTTCCTCCTTCGTTTGGTGCTCTGTACCCTTTTATGCCCTGTATGGAAATCAGAAGTCATTCCTCATTAAGTTCTGGCATTTACCATCAAACTATGAGCAATCTGAGGTACTTCAGTTTAGGCTTCAAGTAGATGCTCTATGTTGGATTATACTGATTTTTTCCCCCCAACAAAAGCTTACCGACCATGTGCTTCCTTTTGGTTGCTACAGTCATTTGAATTGTTCTAGTTTGGCCCTTTTTTAATCTTTGCTGTTAGAGCTTATCATAACGTCCTATAGACATTAGAAACGTTATACTTTGACGTCCTCTTGTCTGATTTTTCCCCCTCCCAATTTTCATGTTCATGCATGTGTTTTGGTTCCTTTTTCAGTTAAATCCACACATAGTGCACTATGGCAGAGTAGATTCAGCTACAAATTCGTTGAAGTTCCCTTTGTCAAGACCTTGCAATATTGGAGAACAATGCTGTCTTAGTTATGGCAATTTCTCTACTTCACATCTAATTACATTCTATGGCTTCTCACCACAAGGAGACAACCGATATGATGTTATTCCAATAGGTATATGTTTTTGTTGTTGTTAATTATTTTTTCTTTTATCGCATTCTTATTACCGTCCGTAGACATCTTTCCAGGTGTTTGAGAAAATCCAAGTTGTATCCCCATTTGAGATTGGTTCTCATTCTTACTTGAATTCACACTCTTCTCTTGTACTCTACTCCCTATCGGTACGTGCTCATGTTTTGATAACATGTCTATATATTATATCATTGTTTTGGTGCAGATATTGATGTTGGTCAGGCTGATTGTTTTGATGATTGCCCAATGTCTAATTGGACCACCCACATGGTGCGGGGTACTTGGCTCTCAAAGAATCACAATATTTTCTATTATGGATTGCCATCTCCATTATTAGACTATCTAAGGAGAGTTCGAAATCCAATGCAGTATGAGAAGACCCATGTACTTTTTCTTTCTCTTCTTCATATGTAATAATTACAGCTGGCAAAATTTTATTATATTTCTTCCCAATTTTTGTTTTTGCCAAACTATGTTTTCAATTTTCAATAAGGAACTTGACTGCACTTCAATGATTAGTTCATACCTTAGTTAATTTTATTTATTCAAATCTTGGATGTGTATGATTTCATTTTTAAATTTTTTTTTTTTTGGAAATTTCTAGCAACAACCTGACCTGGAAATTGAATTGGAAGTCCTGGAAGATCTTCAATCCACCTTCAGCAACATGATGGAGATTCTGGGAGACACGGATTTTGGCGATGGGTAAAGAAATCTCTCACTTATTTAACACAAACTCTGTAATTGAGCATTAAAAACTTTCTTTTGAAAAGAGATTGTGCCATCCTGCAGTGAAAACACAAGCTGGGATGTAAAACTAGCATTGGAGTTCAAAGATCTAGAACGGAGGATTATATCTTCTATTTTAACTTCATGTTCTGCTGGTCGCTCGTTGGTTGAAAGTGAACTGTCCAGAGTTATGTCTAAGGATTGAAAACACCATTGGCTTGTTCAGCTGGCTGGATTGATATTTCCAATCGTAAGCCATAGGTAAAAGGTTACCAATATATAGGG

>CsSDG34

CGAAAATTTACCATCCGTACCTGCTGTTTCTTCAAACAAAATGTTACTGAGACGCAGAACCGAAGCTCCAAAACCAAATACCCATCGTCCAATTACATACGAATCCCCCGATGACGACGATTCTCGCTGCGAGAAATGTGGGTCCGGTGACTTTCCCGACGAGCTTCTTCTCTGCGACAAATGTGATAAGGGATTTCACCTCTTTTGTCTCAGACCCATTCTTGTTTCTGTGCCCAAAGGCTCTTGGTTTTGCCCTTCTTGCTCTCATCATAAGAGGCCTAAACGTATGTCTATGTTTAACTTATTGAGTGATTTTCAATTGGTTTATGGGTTTTATTTGATGAGTTCATTTTATTTTATTTTAATCGTATTGCTGGGTTTTTTTAGTTTTTAATTTTTTCTTTATTGATTTATAGATCTTTATGAGATAAATTTATTGTACCTTGAGTATAATGTCGTTGATTGATCATATTAATTACGTAATTCATTTACAAATTATGTGCAGCTTTCCCTCTTGTTCAAACCAAAATTATTGACTTCTTTCGGATTCAGAGGTCCACAGACTTAACCCAGAAACTGACTCAAGGTATTTGGTAATTGGAACTTTCATTTTATTGGTAATTAATTATCCCCAATTATTATTATTTTTTCTTCATGGGTTCTATATATCTTATTGATGAGTAGAGATTAATTATAATTACTATTTTCAGCAGACAATCTTAGAAAACGAAAACGAGCAAGTGGTTTAGTGATGTCAAAGAAGAGAAGGAAATTGCTGCCATTCAACCCAACAGAGGATCCTGAAAGGAGGTTGCAACAAATGGCATCACTGGCGACGGCTCTGAGAGCTTCCGGAACAGAATTTACTGATGAACTTACTTATGTGACTGGCATGGCACCGAGGTCTGCAAATAGTGCAGTTCTTGAGAAGGGAGGAATGCAGGTAATCTTTTTTGTGGTATTATTTATTATCTTGTTGTAAAATCTTCTCCGAGCTATGCTCAAGCATGTATATTGCTCCACTAGACTAATGAAATGGCATTTTAAGCACCCATTAATCTACTCCGATGTGTGGGCATAATCTTACATGGTTTTCCTGCATTATGCATATGTTAATCGATAAAATAGCTCTCAAATTGGAGAATAAGCATTTGATAACCTGGTTAGTTCCTCTGATTAGATACTATATGACCTGCACTATTGTGTTTGTAACATGTTGGATTGAGATTTTAGTGAACTTTTATCTAACTGCATTTCAGGTATTGTCAAAAGAAGATATTGAAACTTTAAATTTATGCAAACACATGATGAATAGAGGGGAATGGCCTCCCCTGATGGTTGAATATGATCCTAAGGAAGGGTATGTGTACATAAAATAATTTATTTTCTTATTTTGTATGCATATATGCATGTGGATACTTAGCTGACAATACTTGTTTGTCACAGATTCACTGTACAGGCAGATAGATTCATTAAAGATTTAACAATAATTACAGAATATGTTGGAGATGTTGATTATTTGAACAACCGTGAAAATGATGATGGAGATAGCACAATGACACTGCTCCATGCTTCTAATCCAGCTCAAAGCCTTGTCGTTTGTCCTTACAAGCATGGTAACATTGCCCGTTTTGTTAATGGCATCAACAATCACACTGCGTAAGTTAATTTTTAGACTTGTATCTAGCTTTCTTCCTTCAGATAATTAGTTTACACCCATCATCTCTATGGCATCCGAAAAAATTGATAAGCTAAACTGGCATATTTGAGAGTTGACCTATTCCGTAGAAGTCCATAAGTTGTATTGCATTTGGACTGATGCCATATTAATTTAACATACTGGATGGTCACGGCATTAAGAACCAAAGCAAAAAAGATGCTAGGTTTATTTATATACATGGAAAACAAAGTGGACTTGTAATGCATTATGCTATGCATTAAGGCATTGCATTTGTTCCACATTTTGATCTAATGTAAATGCTGTGTCTTGATGCAGTGATGGGAAAAAGAAGCAAAACCTGAAATGTGTGAGATACAATGTCAATGGAGAATGTCGAGTTTTGCTGGTTGCAAACAGAGATATAGCAAAGGGAGAAAGATTGTATTACGATTACAATGGATATGAAAAAGAGTACCCAACTGAGCACTTTGTCTAACTTCCTTTTTTTTTCCCTTTCCTTTTCCTATTAGTAAGATAGGTTGAAGTAACAAGCTAATTGTTTAACCTTCACTGCTTGTCCCCAGCTCACAGACTCACAGTTATGTAACATAACAATTTTTTTCTGGGGAAATCTGAAAGAGAAACACATGTTGATACACATTTTAATTTTTAATGCTTCCATTCACTCATTGCTATTTT

>CsSDG35

ATGGAGAAGCTGAAATCACTGGTTCCAAACAATCTTAAGCAAATGGTGGCACATAGCACTCCTGATGATCTTCCTTTAACATCTTCCTCTCTACTCGACTTCTTCCTCAACTTGCAGCAGTTTCACCAAGTAATGTCACTTTTCTTTTTCTTTTTCTTTTTATTTTCATGTTGGACAAATCTTTATTTATGTTTTATTTATTCTTAGATTGTTAGAGACTTGGCGGGTCAAGAGAGTGGTCTCTGTGCCAAGAACATTGACTCTGCTTTGGAACTGAAGCAAAAGGGAAATCAATATTATTTGAGCGGAGATTACAGTCATGCATTGAGCTGCTATACAAAGGTCTAAATCATGATTCTTTCTTTACCCCTAATCAGTTTTCCTTTCTAACTTGTTACCTGTGCAGATACTACGAAAATGAATTTACCTCATGCTTAATTCAGTCATAAATGTGAATGAATGAATGTGGGGTTTTTTTTTTTTTTTTTGAAACCTTAGTAAATTTCATTTTAAGTGAGTGGAAATAGCTTAGGCTGCAGGGATCAACAAGAATATTTGAATTTAAAGAGGAGCTAATTACAAATCTGTTTTGAACAAAGAATAAGTGATTGAGAATGACCTAAGAAATTGAGCTGGGAAAATGAGGGATGGCTTTGTCGATTTTGTAACATTATACTTGCAATAATGGAGTATAGAACTCACATTGATCATCCACTTGGTTTTCATATCCAAGATTGCTTATATTCCAAAGTAAAAAGCAGAAATCATGTATTTAGCTCTCTGGGGATGTACAGTTACATATCAGTTGATCCTTTTATTTATTCTTTTTTCTGTTCTGATAATTTGTGGAACAGGCTCTGCGTGTTGCTCCAATGGATGCCAATGACAAGGACAGGAATTTGGTTGCAACATTGTATGTGAATAGGGCTTCAGTATTGCAAGTGAGTCCGGTTTCTCTCACCATCTCAGTATGTAGGTCAGTGTGTGGCTTGCATAAAATTGAATTACCTCATAATTCAGTTGAAATAAAAAACCATGAAAGTTTATAGGACTGCCATCTGGTCCCCTTGTGTATCTGTTATCAAAGTATGTTATTAATGGTCGCACTCTTTAGCTTCACTGAACTTTAATTATTATGTGCAACGGCTGAGAGATTTGGACTGGTGCATCAGAGATTGATGCATCTAGGATTACTGTTGTTGAGCCCTGTGTAATGCCCATACTGATGGTGCAAGAATAATATCTTTTACCACATTAACTTATATCTTCATCGTTTTAAAAGAGCCAAGTTTAACTACTTTCCACTTGGCTTGTTTATAGTTTAGACTGAAATCAATTTTGAACCTGTTGAATAATGTAAATCATCTGAATGAACCAAAGCAGGCTTTTATTTACTATTTGAGCTGACTTATGCCATATATGGTAGGCTATTTATTTTTCTTGGTTATTGATCCTACTACTTGTTCAAGTGAATCTATAGTAGACTGGTTTACGAACTTATCATAGCAAGAAGTTAGGGCAAGTAGACATTGTTATAAAAAAAAATAATAATAATGACAAACAATAAATAGTGAGAGGTGTACGCATTATTTACAAAGATATAAAATATATATCATTGGGAGAATGTTCACTTTTCAATCTTTATATTGATTTAAATCTTCTATCTTCCTAAGCTCATTTATTGATTTATTACAGTGTTGAAAACCTTTTTTAGAAACGGGATCATCTAGTGGAGTGCCTACAAGATTGCAATCGAGCTGTTCAAATTTGTCCAAGCTATGCAAAGGTATTATACTTTTCGGATTTATTACATAATTCTGATAGTGTATGTTTTTAAAATAACTCAGATTGATCGAACATAAAATTTTAATATGTCCCTAATGGGATATCCCAGTAGTTGTTTAGACCATTTGCCGTAGAAGGAATATCAATCCTTCTCTTACCAACCTTGGAGGGGAAATTATTTATACTTTCTGTTCGTATTATGGTTGGACTGGGAACCTGAAAAAATACATATCAGATTTTACTATGACTAATTATGTTGCAGTGCACTTCATCAGTTGGCTTGAGTTCTTGAGTTGAAAATGTCTAACATGGTATCAAAGGCAAATCTAGTTTTGTACACCAATAAGAAATGTAATCTATCTTTTATATCTTTTATTCATCTTTCTGATGTCTAATCTCTTAAATATAAGCTCAAAATGAGAAGTCACATGTGTTAATTTTGTCACTAGCATCATTGTGAACGTGATAATTGCATAATTTTCAAGGAAATTTTGTGGTGTTAATATCAATGTTTTGGTTTGAAAATATTATCCACCTACACCGTGATTGTGTGTACATCAAGTTCAGGAACTTATTTTGAAAATTGATTAAAAGCCTACAATATGTAATTTTTGTCCCTTTTTTAAATGATATTGTCTCTTTTCTGTTACTTTGTACTAAATAATGTACACTTTTAAACCAGATGTATGAACCTAATACTAAGGAGACATAATGTAGGGAGATGAAATATTTTCCCTTTCTTTATCGTCTAAGAAAAATTAGATTTATTAGCTTCAGATCTGGCTTGTGATACATATTACAGATATGCTATGTATATTGGAAGGATCCAATCTTTGGTGAAGGTGGAGGTTCACTATGATACTAGCTTCCACAGATCACAGCTTCCAGATTTTGGTTTTCTATGTTGAAGGAGGGCAAAGACAAAATTATCAATCAAGGTTTCTTAAATTAAATAATATAATGTAATGTAAGTATGATGTAATGAAGTATCTATATGCTATTCTGAATACGTGGAGATACATTGAGTCTGAACATATTCACATTTCAGTCCAATCCTTGATACTGGAATTAAATTAAACTCTCAAGAAATTGAGTTTGGCTCTGAAAGGTTGGACCTCTATGATTACTACTTTGACTTTCCTTTTTGGTACTTTGGCCCTTTTTCTTTTATCTTCTGTTAGCAAAACAGGAAATAGTGTTCCTTGTGGAAAACGTAAGCTTGTGTTTAACATGTGCTTCCCACTTTATTTTGCGTTCACATGTATTTCATTTGTAGTCGCATCTCCATGCGTCCACTCATAATCCAAAAGAACTTTCAGGGGAAATTTGTTCAGATTTGGTATTATTATGTATGCTTATCATACATAAGGATATAAAATGTTACAATCTCTATTCTAACGCCCTTTTGGATTATATGATGGCATACTCTTATTCATATGACAGCTTCTGTCTATCACCAAAAATGACATTTTCCTCTCACAGGCATGGTACAGGAGAGGTAAGGTGAATGTTTCTTTGGAAAATCATGACGATGCAGTTCATGACTTAACTATTGCTAAAAATAGAGAGTCTTCATTGGCTGGAAAGAAGCAGATAGAAAGTGAACTGAAGATAATTTTGGACCAGTCTAATAGAACAAGCAATAAAGTGGTTCAGCACACCAAGAACAATCTCAGAGTTTCAGGTAAGTGGCTTTTACCATTGGGCAGTTTTAGTGTATGTTTTGACTTTATCATTATCATTAATTTATATTGCACCTAGTCTTATGTAAATCGTTAAACATATATTAACCATTTTGCTTCTGTATCTGGGAGAAACTATATTTGATTCCTAAAATTGTGAATTTGATGACACTGTCTGCCCTTTATTTCCCTATAAATCACATCATAGCAAGCACACACCAGTGTAATGTCAATTCTATGTTTATTAACTATTTTTGGCTCTTTATTCATATTTTCTTTTTGCACTTTGGTTACATTTACAATCTTATTTTTATTTCAATTTTATGATCAGCCAAGCTTTCTTCTCAAAATCAATTTTTCCCCTAAGGTGTGGTAATGCATTTGGCAAGCCTTCAAGAAATGAGAGCTATAAATTTTGTATATTTGTTCTTCACCTACCTAAATTTTTTCCGTCTTGTCTTGTGTATAATTACGTAACTTCGTCATCATGACAGCACCATGACTAGTAAAACAGTCCATCATCTCTACTGATAAGTCATAGGTATGCCTAGTGAAGTTCATTTCTGTCCAAATTGTACTTATCGAGATCTTTTGTTCATCAATAGCAAAGCTAGGAAAGAAAGAACCTTTTTTCTTCCTTCAACCCCATCAGATTATGAAGATGTGAATGCACCTTTCTATAAGAATGCTTTGGGTTTCAAGGAACTTCAGTGTAACATTTGCATGCTCTGATTCCTTGGTTAATTTTATTGTATTTTGTTATTCCTAATATATCAACTTGTAATGAATGGTGTTCTTATTTTGATTGCATGACTAACATGGTACCTGCATTTGTTACAGATGAGTCAGTCCAAGTCCAACTACAGTGTGTTACTACTCCTGATAAAGGAAGGGGCATAACTTCACAGTATGACATTCCTGAAGGTTCCTTGGTGCACAGTGAAGAACCATATGCTGTGGTATTAACTTTAGTACATTTTGTCTCTCCTATAATGCTTACTTTAGTTGAATTCTGATCTATATGCTTAATGGAAGCCCAATTGTCTCTTGGAATAAGCATAGTGTGATGTCCAACTAAAGCTATCATCTTATTCAAGCACGGTAAATGACAGATGTACTAAAGTTATGACAAAAACAAAAGTTTTCACAGTCAAGAAGATGGAAGATGACTTCAAAAGAACTTTCTACAGTAGATAATATAAAACACATGCTAATTTTCTCACACAATCTAATTTACATTCAACTGATTCAAGCACTGTGAATCCATGTGCTAATGTACTCACACAACTTAATTGTTTCGTTTCTTTTCCCCATTGACATTGGAGAAGTACTTGATGTTTCCATGAGAGTCAAAAACATGTCTTTTGATCCTCGTCTAAACCACAACAATTATTCCCTATTCAGTGTCCAACAGTATATTATAAGGCTAGATCAAGAGTACTTATAGTTTACATAATTTTATTACATGATTTTTTTCACAATTGGATTAGTATACTGTAAACTATTAATATTGTGTCTCACATGTGCATTGATTATGGAGCATTAATTACGCACATCAGATTCGATCTCAACCATTAAAAGTGCAATAATCCAATGGTAATATAGTACCACTGTGCATGTAATGGAGGAAAATAATAATAACATTAACGAAAGGAGATGGAGATTGAAAACTTGGAGGGTTTTCAATAGGTTGGATTGTGTCCTTGCAAGTATGGGCTCTCCATATAGAGGACCATGAAGTAGATCTTTTGTCATCTTTCAAGTCTTGTTTTTCACTGGAATAGAGTGGGTGCCTTGTCATGTGGATATTGTTGTTGAACCATTGTCCTAATTTGGCCCTTTCTGTGCCCTTTCTGGGTTTTATTGATTGGACCCTTCACATGTGTTGTGTAAGGTCTTAACCTTTGTTTCTGCTGAATAGAAATATAATCTTTAATTGGCACCATAATCTGCAGACTATCTCGAAGCACTGTCGGGAAACTCATTGCCATTACTGCTTAAATGAACTACCAGCAGATGCAATCCCTTGTACATCATGTTCAATACCATTGTATTGCTCCCGTCGTTGCCGAGGACAAGCAGGAGGACAAGTGTTCAAGAACTGTCCAATGGAACGTAACATTAATGATAGCGTCTTTGACAATCTTGAAGAGTATATTTCACAGATTACGTTAGACAATGATTTTTATCCGGAAGATGAGCACATTTTCGAACATAAACATGAATGTAAAGGTGTGCACTGGCCTGTAATATTGCCTTCTGATGTAGTTTTGGCTGGACGTGTCCTGGTCAAATCTGTACAGAAAAATGGAGTTTCTATGGATGTCCCTAACCTCTTAGGAAAATTGGTACATCTCTTGACTGAAATTTGTGCATTATATTGAGTTGCAGTCTAGTTCAGTGAAACTATCTTTTTATCTATTTTAGTTGTCTGCCTACGGTCTTTATATTTTCTTGTAGGAACTTTCGCACAACTACTCGCAGGTTTCTTCAGAAAGCAAATTAGAATCACATATATATGCGATAGTATTGTTATATTGTCTCCAACACTCTTATGGCTTTGAACTTCCAATAAATGGAGCCTCGGTATCCCAGGTTGGTTCTTTCTCTCTTCTCTCTAAATAGTCTTTAATAGTAATTCAGTTGAAGTTGTTTGGTCAAAGAAAGGGACAGAAAGAAGATTCCTTTATTCTTTTTGAAAACTAAGAATAAAGTTATCTCCTCTCTCACTACTATCCACTTTGATTGATTTTATTCGTTGGTTACTGAACTGAGCATGTATTATTCATGTCTTTAATATTGTTTCTGTGCTCTTGGTGTGCAATATATCCATTATTTCTGTGTGTGTTTGTTATATGTAGTATTCTTCATGAATTGAGGGTCAATCTGCATTCCCTAGTAGTTCTGTTTAAATTTTTACATGTCTTGCTTTCAAATATGTTTACATTCTTGATTTATTTAATAAAATTGTTGATATATTTTGCTCGTAATCTTCAGATTCCAAATTCATCAATTTCTTCCTTGGATGAATTTTAATCCTTAGCATGTTTTGTGCCATTTTCATGTTGTTACAGGTTGTCATACTTATATCTCAAATAAGGGTGAATTCTTTGGCAATTGTCCGCATGGATTCCAATAATTATGGACAATCGGATCATGTATCAAGTGGAAGCACTTGTACTGTGGAACAGGTTTGTGTTAATTCTTAAACCTCTATCCATCCTCATTCCTTCTCCCAACACTTGTATTGCTTTTTTCTCCATTTTCTGACAACTATTTAGAAATGCCAATCGGAGGTATAATCCTTATGTACATTGCAAACATGGGAGCACTAAATATAAAAACATAAACATTCATTCCTACAGCCACATTGATGAACTCCACAAATCAAACATGAAAATTAAGTGGAAAAAACAAGAAAACAAACTCTGTGACAACCTAAGTTTATTCCTGTAGATCCAACTGCGATTCTTCATCTAAGGCTAAATGCCTTTTCCCTTCAGAGGCTGTGAAACACATTTTCAAGCCAAAATCAGATATCCAAGCCTTACACTGTCTGTTCCTAGTATGTCTTAACAGAATCATATAGGTATACTGGACGGTATTGTTCTGAGTTGTCATTGAAAATTCCCTAGTATGATAATCAACAATCTTTTACTGTAGTAAGACTTGTCTCATAAAACCATGTAATTGTGATTGCTGACAATCAACTTCAATCAATACATGATGTTCATCCTCAAACTATATTATCTAATGATATTTGTTACTTGTACAGGTCAGAGTAGGTCTGGCTATTTATACAGCTGGCAGTTTGTTCAACCATTCTTGCCTGCCAAACATTCATGCATATTTTCTTTCTCGCACACTCATGATACGAACAACAGAGTTTGTGCCCTCAGGTTATCCCCTGGAGTTGTCCTATGGCCCTCAGGTATTTATAGTTTCGACCTCTTAGATTATCAAATTCAGTCCATCACTGTATGACTGTGATGTTTCCTGTGTGCTCTTGACTTATTGCATTAAAGCAGATATATTGAAGTTGGTAAACTTGTTTCCCTTTTTAAATTTCAGATCACCAAATGGTGCGAACAATAAAATGATGTCTAGTTCACTTTATTTTACAGGTTGGGCAGTGGGACTGTAAAGACCGCCTTAAGTTTTTGGAGGATGAGTACTCTTTCCGTTGTCAGTGCAGTGGTTGTTCAGAATTGAATACATCTGATCTGGTTATAAATGCTTTCTGTTGTGTCGATCCAAATTGTCCTGGTGTGGTTTTGGATAACTCCATCCTGAATTGTGAAAAGCAGAAACGTAAGCACTTACCTGCAGTTCCTCAATGTAGTAGCTCAGTACCTCACTTGCAGGTACTTCAATTGCTCTGTCATTTAAGTTTAGCAGGTCCTTGATGCTTTTATTAGATTTTAATTGAACATATTCTCTCAATAACTAAAAGCCCCGGATATTTCTTGAGCATTTAAGAGTGCATTTGGTATTTTTTATGAAATTCCAAGATTTGAGTATGCGTCTGGTATTATTCTTCCTACTCTGTATTTTAAACACAAAAGCTGTAATACACATTCGGTAGATGTAATTGAAAATAATAAAAACGGTGAATATGTATTAGGTAGTGCTATTTTAAAAAACAGTTGTTATGCAAGTTATATTTGTGGTTTAAAAAGAAAGTTATTTAAAGACTATTTTAACATATGGAAGACTTTTACTTCTACAAGGAAACATTTAGTTTTCAAATTTTGCCTTAGAAATTATGAATTTATCTTCTATGGACCAAACTTGAATTCTTGTACTCTACCACCAACAGCGGGCCCCTGATTGTATTCAAGACATTCCCTAAAGACATGGGCTATTTGATCGTTATTTCTTCAAGTCCTTTTAATAGGTTGGTTAAATCTATATTCTTAGTGCAGTAGTGCATTCAGCTAAGGCCTTCTCCACTCTACCTATTTTTTGACCAAAGAAGAATCCCATTTGTCACACACCACCTAAAATTCCAAGCCAAAGTGGAATCTGGACCCAAAAATTTGTTTTGATTTATATTTAATTTTCCTTCACCAATTGATCCGCCATGATTAACTCTCTGTTATCAGATAAAGCATTGGAGAGCTAGACTAACACTTCCTATTCCATATTTCTCTCAAGACCCACACTCATTAGGAAATTTTCTTGGATAGAATATGGATGAAGAAATACACTGCAAGAAGCCAAGAAATTAACAATATTGGTCTTCTTCCACATTAGTTCTCTTTCGGAGTCAGGGAGAAAGGCAGACTCTTTTGTAGAAGGGGAGCCCCAAAAAAAAAAATTCTAAGAATTAAAAAAAACCAAAAAAAGAGGGGGAGAAAAGTTATGGTAGAGATTGTGTATTTGTGTTTAATTTGGTTGGAAAGGAAACCAGGTAGGTTGCGGAGAAGTACACAGATTCGCGTCGCATGTTTTAATTTGTTTCAAGGAATATTTTATAATTTGTTTTGCAAAATGCAGAGAACGCCTAAAAGTTGTTTCCTATATCTGTGCCACTTGTGTTTTGCATTTTCTGAATCTCTTTTTATAATATAGTCACCAAGCATGTACTTCACCCAGATTTTGGAAACAAAAATGAAAGTGGGCTCCATAAAAGAGTACGGAACAGGCCCTAATTTTATGATCTTTCAGGTTGGCAAGCTTAGTAGTGATTACATTGGTTTAGTGGCTTACCTTTTACTTGAGGAAAACAATAGAACCTCTCGTTATGGTCCTGGATACTGTTTGAAATGTGGTTCTGATCGTGATCTGGAATCTTCATATGCAACTGTGGATGAAGCTTGGATTTATATCAGAAGGTATAATTTACTATTTTTTTATCTGCACTACGTTGGTTTCATTCTTCTTACCGTTTTCACAGAAGAATATTCAATTCTTGATAGGTTGCAGGATGCAATAATTTCAAAAGAAATCTCAAGAGCTGTACTTTTAGATGCTTCAAGATTTCTTGGTCTGCTGAGATCAATATTGCATGCTTACAACAAGAGAATAGCCGAAGTGAGTTCTAAAGTCCCTAGTTTAATACATGTTTCGTTATTTACTTATTTGCAGTTGCTTTTATCTGCAGTGTCACTATATGAATTGAGTGACTATACTATAGCTCCATTCATCTGGTCAACAGAGAAGTACTTAAATGATTATGATTTTAATAAGAACAACAGGTAAATAACAGTGTGCATGACTTCTTTGCAGGCAGAGGACAATCTTGCGCAGGCATCTTGTTTGGTTGGAGACTTAATTTCTGCAAGGGACCATTGTAAAGCGTCAATTGAGGTAAACTTTTTGTTAGGACAGTGTTATAACAGGCTACAAATCTTGTTACAAGATTTTATGGTAGATAGTCACCTAGTTATTGTGTGAGGATGAATTAGGAATATTCAATTATATGTTGCTTGGTACGGAAATGTGCAATTCTTTTGAGCTTTTTTTTTCTCTTTTTTTCTTTTTTCCCTTTACATAATGTGAAGTTTTTTATGCTGCAAGGTTGTTGAGAGAGAGAGAGCGAGAGAAATGAAATGTGTTAAATTATTTATTTATTTTTTAATTGTTTTGATCTGTTTGTTCTTCTAAAGTTTGATTTATTCATAAAAGAACATGTGTAAAACAAGGACAGTAGGAACAAAAAAAGAGGGGGAGAAAAGTTATGGTAGAGATTGTGTATTTGTGTTTAATTTGGTTGGAAAGGAAACCAGGTAGGTTGCGGAGAAGTACACAGATTCGCGTCGCATGTTTTAATTTGTTTCAAGGAATATTTTATAATTTGTTTTGCAAAATGCAGAGAACGCCTAAAAGTTGTTTCCTATATCTGTGCCACTTGTGTTTTGCATTTTCTGAATCTCTTTTTATAATATAGTCACCAAGCATGTACTTCACCCAGATTTTGGAAACAAAAATGAAAGTGGGCTCCATAAAAGAGTACGGAACAGGCCCTAATTTTATGATCTTTCAGGTTGGCAAGCTTAGTAGTGATTACATTGGTTTAGTGGCTTACCTTTTACTTGAGGAAAACAATAGAACCTCTCGTTATGGTCCTGGATACTGTTTGAAATGTGGTTCTGATCGTGATCTGGAATCTTCATATGCAACTGTGGATGAAGCTTGGATTTATATCAGAAGGTATAATTTACTATTTTTTTATCTGCACTACGTTGGTTTCATTCTTCTTACCGTTTTCACAGAAGAATATTCAATTCTTGATAGGTTGCAGGATGCAATAATTTCAAAAGAAATCTCAAGAGCTGTACTTTTAGATGCTTCAAGATTTCTTGGTCTGCTGAGATCAATATTGCATGCTTACAACAAGAGAATAGCCGAAGTGAGTTCTAAAGTCCCTAGTTTAATACATGTTTCGTTATTTACTTATTTGCAGTTGCTTTTATCTGCAGTGTCACTATATGAATTGAGTGACTATACTATAGCTCCATTCATCTGGTCAACAGAGAAGTACTTAAATGATTATGATTTTAATAAGAACAACAGGTAAATAACAGTGTGCATGACTTCTTTGCAGGCAGAGGACAATCTTGCGCAGGCATCTTGTTTGGTTGGAGACTTAATTTCTGCAAGGGACCATTGTAAAGCGTCAATTGAGGTAAACTTTTTGTTAGGACAGTGTTATAACAGGCTACAAATCTTGTTACAAGATTTTATGGTAGATAGTCACCTAGTTATTGTGTGAGGATGAATTAGGAATATTCAATTATATGTTGCTTGGTACGGAAATGTGCAATTCTTTTGAGCTTTTTTTTTCTCTTTTTTTCTTTTTTCCCTTTACATAATGTGAAGTTTTTTATGCTGCAAGGTTGTTGAGAGAGAGAGAGCGAGAGAAATGAAATGTGTTAAATTATTTATTTATTTTTTAATTGTTTTGATCTGTTTGTTCTTCTAAAGTTTGATTTATTCATAAAAGAACATGTGTAAAACAAGGACAGTAGGAAGAAGAAATATGTGGGGAGTTAAAAAATATATATATATATATATTGTAACTCCGGACGATTGTACAAGAGGATTAGGAGGATATCATATGTGTCCATTATGAACAAAAGAATTCAGAATTATGTTTAAATTCAGCATGATCTTGATTATAGGGATCTATGGTCAGAGATAAGAACTGATTAAGTTTTGGTGTCTCTTGCTTATTGCTTGGTTTTATTAGGTTTCCCTTTGATTTTTGAGCCTATGGTAAATTTGATCATTTTTGGAAACTGTTGCAATTCTAGTGAAATGCCATGAGTCCTAAATCAATGTTTAGTTGTTTCATTATACAAAGTGTTTATTTTTATGGCCAAAGTTTTAAATATCTCTATTTCCTATTTAGGAATCCTCATTTATCTATTTAAAACATAAAGAGAATAACAGAGAATTTCACTTCTCTAAAGTTTCATTTAGAAGGTGTAGTTTCCTTGTTCTTCCAGGTTATGAGATTGGATTACAAACTTGCGTGTTTTATTATGCATTGGCAGATACTTGAAAAGCTTTATGGTCATAATCATATTGTCATTGGATATGAACTGGTGAAGCTTTCATCCATTCAGCTGTCCTTGGATGACCACAATGCTGTAGACACCATAAGTCGTCTGGCTGCAATATTTTTACACTACTTTGGATCACATGCAGAAACGATGTTCCCACATCTGCTATTCCTTCAGAGAGAGGCTCTTAAACTGCCCCAATAA

>CsSDG36

AGCTCATGGCAAAGTGCTCATATTCGTTTTATGAACATTTTTAATTTTTGTGCGGACGCTGGAATCTGGATATAGTACGAAGTCCTAAGCCTCGCATCAGTCAATTAACTATTCTCTGCAAATGCTAGTAGGTGCTAGACTCACAGGCGCATGGTGCTTTCGACATCGACGCCCCCACTGTGCCAAGGCCAAACTCACTTTCTCATCCTCATCTGAATCCAAGGTTAGCTGAATTCACAGAACTATCTTACTTCCTTTCTGTTTGGTTGCTGAGGAAATGCAAGAAAAGAAAAGAGAATTTAAATCATCACGTTACATGTTGTTTTGTTAGCTTTAAATTGCATGCAGCCATGTTTGGGCTTTGTATTTTTCGTTTTCACTTGAAAAACCTTGACGTCGTACGCATAAACACATAGCAACGTACGTACATAAATGCGAACTCTATTAAATTTTGATTTAGGATCCAAGCTCATGTTAGCTAAACTCTCTCCTGATAAGTTTTTTATTTAGCTCGAAAATAGTGTGAGGTAAGTGTAATTGAACAATTTTTTCAATTATTTTTTTCATCGATTCTGTGAAAGAAAAGGAAACAGCATAAACATATAATAGATACTTACGAGTTAACGTCCATTGATGAGGAATGCGGTGATGATTGTTAACTGTTAAATGATCTCACTTTAGGTAAGTTGTTGCGTTTTATTTACCCGTTAATTTATGAGATTTTTGTCAGGTATTGCATTCCATAGATGACGAATATGATGGTGATTTTTTGCCATGGTTGGAGAGAAAAGCTGGGGTTGAGATTTTATCAGTGCTTTCAATTGGGAAATCTGTATACGGGAGGTTGTTATACACTTCTTTGTTGCAAATACTCATAAGTCATAATTATGCCTGCTTATAACTTTTATTTAAATTGGAACATGGTAGCATAGTTCCGAAGATCCAAATGTCTGTTCACTGTTTAGTTTTAATTTGAGGTGCTTTCAGTGTGTAATGGAAGTTAAGGAAGTTCTCAAACACCATTTTTGTGGTTAGTGGGGCATATCACCTTTTTATTTTGTTCCTAGGATAGGTTGTTTATAATTGAACAGGAGAAAATTTAAAGAAGCCTTGAGTCTGATTGTTTCATGATTATGACAATGAGTGTGTGTTTGTTCTTCCTCCTTTCTCGTGCTTTTGTCATCTCATTGGCATATGCTAAGTCATTTTTCCTTTCTTCCTTTTCTAATTTTATATTTATTATTATTAATGTTTGAATCTGAATGCAGATCACTGTTTGCTTCTGAGAAGCTTCGAACTGGAGATTGTATATTGAAAGTTCCTTATGCTGCAGTATGCTTCTCTTGCTAAACTTATCCCAAACTAGTCTTTGATGTCCTTAACCTACTTTCTAAAGTGTAGTTCAAAGTAGTCAGCATTTTAAACTGTTATTTATGTATTTCTTACTTGATTTCAGCAATTAACCCCAGATAATCTCCATCCGAAGATCAAATCTTTGTTAGATGATGAAATCAGCAATGTTGCTAAGCTTGCTATTGTCATTTTATTTGAACAGAAAATGGGTAAGGTAATATTATCCATCTTGATTTGAAAAAAGTTATTGATGAATAATTCTGCAATTTTCATTATTTTGCATATCTGCTTGAGGTTAGGATGTGGTTTGTTTTGTTTAGCTTTTTTCAGCTTATACAGTGAAATGCGAGAAAATCTGAAACTGAATGACTTATTTACTTTTCTTATGAGGCAGGATTCAGAATGGGCCCCTTACATTAGCAGACTTCCTCAGCTGGAGGAGATGCATAACACAGTAATTTTCTTTTCTTATAAATTTATGAATGTTTCTTTCCTTTAATATTGATAAGCCACAAACATTTTGACTATTGAAGCACCAATAAATATAAGACAATGAATTTTGATAATTATTCCTCTTATATGTATAATATTATTGAAAATGTTAAGTTTTAAAGAGTATGGTAGTTAATGCTGCTTAATTTCTGATTTACACATACAAAACTGTTTGTCTTTTAAAGGCTACTTGACTCAACAATTTGTTCTAACCATCATCTTTCTTCTTTTCTGTCAGAAAACCAAACTTTTATGCATTGTACAAAAAGATCTTTGCAAAATTACCCTTTCATGTATCTGATATATGGTTTTAAGTACTGAAGAAAGTAGTTCACAAGATAAGCTAAACGCTTGCTACCAAATCTTTGCAGATATTTTGGAGCAAAGATGAGTTAGATTTGATTTGTCCTAGCTCATTATTTGAAGAAACAGTTACAAAAAAAGATCAAATCGAAAGTGAATTTCTGGTAACTAAACCAGTGAGTACATATGTTCTCCACTGGAATAATTATAACTCATATGTATAGTAGTTTGATTGATATTATAGTTTTAATTGCTTTTTTCCCTTGGAATTTGCAATTATGATACGGTATCTTTACAATTTCAGGCTCTTGAATGCTTCCCTGAAGTTTTTGATCATATCAAACTGAAGGATTTCATGCATGCATATGCTTTAGGTTAGCAATTCTATTTGAATCATAATCTGTTGTTATCTTCTTGAGTTATTGTCAGCTTCCAATTTGAAAAAAGGAGGCTACCTTCCTGAATCTATTTGGAGGCTATTTTCTTCTTCTTCAATGTCAAAATTTTACCATGAATCTACTCTGTCATAAAAATGGACAATATATTGAAATATCGATCTGGCTTTGTCTCATATGATAATTTTTATGTGTTTACGCATTCAGTTTGCTACTGAAGTTATCAACCTCTATAATATTGATAGAATATAATGCATCTTGGTTCACTCTTTTCTTTTCATACATAAGTCACTGTTATGAGATTTGAGGCTGTTGTGAAACCACTGCCAATAATTTGCATCTAAATCCTCCATCCTTTTGTAAATAACCGGTAACTGAAGAAAGTTTGATAGCATAATATGTGATACCACTTTAGTTTCTTGCAGCTTGAAAATCCTATACTGTTTCAGATTCTAACTTTATGGAATAATCTGTTCACTTTGCAAATGATATGCAAAAATTTTTGGAATTGACTATTTTTTATTGTTTTTTATTTTATAGCTTATTGTGTTAGAAGTTCTACTTGCTTTTATCTTTATTTTCCTATATTTCTGGTTTGTAATCTGAACTCCTGAATTAAAAATAAACTCTTGAATCTAAAAATATAATAAGTTATCTTGATTAGTTTGGTGTGAATGTACCAGTCGAGTCTCGAGCATGGAGAAGCACAAAAGGGGAATCACTGGTATCATATTATAAAAATGCCATCTGTATAGAACTTTCGATGTTCACTTCATTTGTATTCTTTTTTCCTTATTTTGCTCAATTCTTAGAATTATTAAATTATTTTATAGATTCCATTTGCAGATTTTTTGAATCACGATGGGCTTTCAGAAGCAGTTGTGTTGCATGATGAGGATAAACAATTGTCAGAGGTATATTCTCTCTTTTTCTCTTAGTTTTTCCAGCTCTGTAATGAAGGAGACAAATAGTAGAATAGAACTCTGTTCTATATGGTCTCTAATGTTTGATAAATTGTTCTTTATTTTTAAAATCAATAGGAAAGCTTGATTTAAAACTTAAAACAGAATAGGTCTGGACAAGATGCTTGTTCTCATTAACCATTGCTCTTATTTCTGCTCCTAATAAAGTAGGAAGTCTTCATTATTGTAGGTAATTTTGTAAAATTTGGTAGGCTTGAAGGAAATGTCTATTTCTAATATTAATTCTTTGAATTTGTCAGTGCAGGTTATTGCAGATAGAGATTATGCTCCTAAAGAAGAGGTCATATGCAGTTTTGATGATTCTTTTCTTCTATTAAAATAATTTTATGATTACTCTCCGGAACTTACTTCTGTTTCTTCTGTTTATATGTGTGTATTAGATGTGTATACTCAGTTTCTTGTGTTTTTTTTTTCCCCTGCTTGTCTGCCTTCTAGTGTTGTCTGGATATAGCTGTAAACATTTATGTGGCCATCTGGTTAACACAAGAGCCTGAAAATTTTTTCTTTACATTGATTAATAATTTTGATGTTTAGTGGCTCATCAATGGCTTTTCTTTCATCTTAAAGGTTGGCATCTGCATATCTAATCGTTGTATGCAGATAGAAATTGCTGCTTAAAAATATTTATATTTGTTATTGATTCAAGAATATATATATATATATATGTTATGTCCTTGGACAGTGAAGAAAACTATGAATTGGTTCCTCTTTTTCTTACACTCCAGAACAGTATGTACAGGGAATATATACAGCAAAATAGGAGAGAAAATAGGAGACTACATCTATCCTTTCCTAAATAATAGGAAACAAAATCTGGAAAGAATCCTAATGAATCTTCCCATTATAATTTAACTTATTCCTAAAATAGCCTAGATTACAAATAAATCATAACTTTGAATCTTTATTTTCAACACTCCCCCTCAAGCTGGTGTGTGTATATCATACAAACCCAGCTTGTTACTTAAGTCTTCAAAGTTAGGTCTTGGCAGTGCTTTAGTAAGGATGTCTGCAATTTGTTGTCTAGTTGGAATGTACTTTAGTTCCACACTTCCACTAGTGACCTTCTCTGAGATGAAGTGCCTATCAATCTCGACATGTTTGGTTCGATCATGATGCACTGGGTTTTTTGCGATGCTGATTGCTGCTTGATTGTCACACCTCATTAGAATGGATGAAGGGCTGCACACACCGAGTTCCTTGAGTACACGCTTTATCCAAATTCCTTCACAGATTCCATTTGCCAAAGCTCTGAACTCTGCTTCTGCACTACTCCTAGCTACAACTGATTGTTTCTTACTCCTCCAGGTTACTAGATTTCCCCAAACATAGGAACAGTAAGCAGATGTTGATCTTCTATTTGTGACGTCTCCAGCCCAGTCTGCATCGGAGTAGATCTCAATATTCCTATATTCTGATTTCTTGAAAAACAATCCTTTTCCAGGTGTCATCTTTAGATACTTCAAAATTCTAAAGACTGCATCCATGTGTTCCTCTGTAGGATTGTGCATGAATTGGCTCACAGCACTAACAGCAAAGCCTATGTCCGGACGAGTATGTGAGAGATAGATTAAACGACCAACCAGACCCAAGATATTATGGGATGGCCGTTTAAATTTGTAGAAATTTTCTCTCTTTGTGTTATGAGCTAAGCAAGTTAGCGAAATCATGCAGAGATCATAGCAATTCCCATTTGCTATTAGTACTAAAGGGTTTTTGTAACAGAGATAAGTTCAAGCAGGCTGCTCAGTATGGAAGAAACAAAAGCAACCAAGATCTTTACACTTCAAATCTCTTGAAAACTCTTTCGTTTTAACAAAGAAAAGTAACCTTAGATTTTAAGTCACAAAGAAATTGGACTATCCAGCATTTTTGGTATGGGGACAACTAAATCATTCTTTGCTTGTTTCCTGCGTCCATAATGAGAAGATATACCATTTGATTGCAATCTCACAACCATTGATTTGAATTCTAAACTCGTCAATTTCTGATGACTGCAGCAAAGGTTTTACAGGCCCTACCTTTTCTCTATCTGAATGAATTCTTCCCATTCTCAATAAGACACTGTGACAGTAAAGCGAATCTCCTTACCTCCACTCTCCAGAACTACCAGCAGTGCCATTGTCCACCTCACCAACCTCAGAATTCAAGACCTCTTTCCAACAAACTGATTGGGTTAATTCCAGGTTCATTGCAACAATACAACTTGGTTCTCCGTCTCCAGTAATTCCAGCTTAGCAGACTCAACTCCAACAACAACTTTACTGGCAGCTTCTGCCCCTGCAAGTGTTTCACTAGTTGCTCTAATATAAGTTTGTAACTGCTATGTATCGCTTCATGTTCAAAGAACAACAGCTAAAGTATTTGGTGAGAACCCTCTTGTAATTGGCAGCTAACAGGACCAATTATCCACCTACTTATACTTGATGGTTAGCGATTCGAAAGCAAGTAAAGAATTTAATAGAGGATTAAAACAAAGCACAAGAACAAGTATTTGGTTCAGCCACTTGGGCATACATTCACGGGCTAAAATGGCTAGTGTTTTTTATTCAAGCTTTCGAGAACATAGCATAAGAATCACAATGTTACAAGGACAAGAATGAAACAAATGGCTCTCAAATTAGTTAAGGTCTTCATGAGAAGGACCCAAGTTTGTGCTACAATTATCAGCCCCGGCCACAGCAGTTTCCCAGTGGAAAAAAGTAATTGACTTTAATCAATCAAACTAGCTATCACTGCCACATAAGCAGTCCCCTAACAACCTTGACACATCAGCATTCTCCCTAAGGAACTCAACTAAATTTTTCTAACCAATTTTCAATGAAGAACTTGTGGAAAAATAACAATACTTGTCAGTTGATAGAAAATAATTAGTCGTCAAACTTTCAAAGTATACATCTCCATATATTCAAATCTACAAATGAGGGATGACATAATCCTATTTTTCTGTTTTTTTAATATTTTTGGCGATTTCTCACTTCTCCACAAGAAAACTTGAAATCTGAGTGACTTCTGATCCTGGGACCTCAAAAAGTAAAACAAAAAATGGCATTACAGAGTATGATATTAGCATAAGAAATGAAACAATTGCAGCTGCAGCTACACCTGTCGAAGGAAAAGCTTATGAGAATCAAGTATGAGCATGATTCAAGCTTTTGCACAAAACGTGTATGCTTAAGAGTTTCTATGCAAAAAATATAAGTCAAAAAAGGAAAAGAGAAAGAAAATCAATTCAATATGATATAAGACCAAGAAAAAAGATCCATCTGAAAAAAAAAAATAAATTATTGGACTGCAGAAGACCAATTCAACAGTCAAGTCTTCTCCATATAAATTGCTGATACCTAAACCCATATATGATAATCAATCCATTCCTGAATGAGAAAAAATTCAAAATACAACCCACTATTTTATTCAGTAGCAAGACATCATCAAGTTCCAAAAATAACTATTTTAACTCTCAAGTTCATTTGAACCCTTTCTGAGAAAATAAGAGAAAAATATACACTATTTCCTAAGTTTTAAATTCTCAACAAACAATTTAAAAATTAATATAGTACCTGCAGCTGGTGGACAAATCATCAGTTGTCATACGGAAAAGTCTATCCTCAGAAAGTAAATGTTAATGAAGATTCAAATCTTTAATTTTCTCAGATGACTTCAGGATGTTATTGTCTCTATCACTAGTCATGGAGGTTTTAATATTCTAATGCAACCTGCTATGAACAAGTTAGTCCAAGTGATGCCAAGAAGATTCCCAAGGCTGATGTTCGCTCGTACTCTCTCCCTGAACAGCAGTTTTGGAATAGAAAGGCAGGTTTTCAACAAATGTTACATCTCTGGAAATGTATATTTTTCTGGTGTGAGGAGAATAGCATTTGTATCCTTTTTTATGTGGAGAATAGCCTAAAAATATACACTTGAGAGATCGGGAATCAAGTTTAGTCCTGTGTTGAGAGTGAACATGGACATATGCTGTGCAACCGAATATTTTTTGTGGTAAATCTGCTGAAACAATCCTAGTGTGTGGAAAGAAATTGAGAAGAACTTTTCTTGGGCAATGAAACCCCAAGACTCTAGAAGGCATTCGGTTGATTAAGTAGGCTGCAGTGAGAACAGCTTCACCCCAGAAAAAATTAGGAACATGAGATGAGAACAAAAGACAGCGAGCCACTTCAAGAAGGTGTCTGTTTTTGCGTTCAGCGACCCCATTTTGTTGCGGGGTGTCAACACAAGAACTGATGTGAAGAATGCCGTGATGGAGAAGATATGAGCTGAGGGAGTCTTTGAAATATTCCTTGGCATTGTCGGTTTTAAGGACTTGGATTTTTGTATTGAATTGGGTTTGAATCATGGCGTTAAAGTTTTGGAAGATTTGACTAACTTCGGATTTGTTTTTCATGAGAAACACCCAAGTGAGACGAGTATGATCATCCACAAATGACAAAAACCAGCGAGCCCCAGAAACATTTTTAATTCTTGAGGGACCCCACACATCACTGTGAATTAAAGCAAATGGACGAGAAGGCTTATACTTAACACTTGAATAGACATTTCGAGTATGTTTTGCAAATTGACAAATTTCACAATGAAAGGATAAAGGGTTTTTATTGATGAATAGATTGGGAAAAATTTTTGCGAGATACATGAAACTAGGATGACCTAATCGATAGTGTAACAGAACAATTTCACTATCGTTATTGACATTTGGACTAACAATAGAGTGAGGATAACTAGTTGACAGATTATTGGACTTGGCACAGTTTGCTCTCGAATCTTTTATGAAGGAGGATTGATCGCTTTTGAGTAGATAGAGTCCAGAACATAGCTCAGCACTGCCAATCGTCTTCCCCGAATCCAAGTCCTGAAAAACACACATATTTGGATAGAATTTAGTTACACAATTCTGATCATGAGTGAATTTACTGATTGAAAGTAAATTGCAAGCCAAATTAGGCACATAAAGTACAGAATTAAGGTGTAACTCCTTAGTGAGTCTGATAGAACCTGTCCCTGCTATTTTTGTGTGAGAGCCATCTGCGATCCGAACTGAAGTGCTGTTGATGCTAGCTTTATAATCCTGCAACATTGAGGCATCTCCAGTCATGTGATCAGAAGCACCAGTGTCAATTATCCAGGGTTTGCTTGTTCCATTAGTGGCTGCAAATGCTGAATGAGTCTCTTTCTGAGCCACTAGACTTGTCCCTGCATTGGAGTTCAAAGTGGCTTGATCGAAAAGCTTTTGAAGAATCTCCATTTGCTCCTTGCTGAATGGATTTGGCTCGGGTAGGGCCTGATTGTCCGTGGTGGATGCTGCATGAGCACGAGATTCACGGTCACCTTTGGAACGAGGTTTCCAATCAGCAGGTTTGCCATAAATTTTCCAGCAACTCTCCTTATAGTGGCCTGCCTTACGACAGTGATCGCACCATGGTCTTTCTTTCTTTGTACGATTTTCACTAACCCCTATAGAGTTGGATGCACGGGCAGCAAGTGCAGAGGCGTCCAGTGTTGGTTCTGATGACCCCATCATCACCTTTTTCCTACTTTCTTCATGCCTAACTTCAGAAAAGGCTTCACGAGGGCGCGGTAGAGGCTTTGTCCCGAGTACACGGCTCCTAACCTGATCAAGATCTTTATTGAGTCCGAGGAGAAATTTAAACGTTCTCTTCTGTTCCACAATCTTCTTGTACAAGGTTGTATCGTCGGTGCACTTCCAAGGGTATGTTTCAAATAGATCAAGGTGTTGCCAATGCCTAGTCAGAGTATTAAAGTACTGAGTGACAGATTGTTCTCCTTGGCGGAAGTCATGGAGGATGGTTTCAATCTGGAATAACTCTGATGTGTTCTCAGAGCTAGAATAGGTTTCCTTTGCAGCATCCCATATGTCCTTTGTAGTCCCAAACAGAAGAAAATTCTCACTAATATCATTGTTCATGGAATTGATAAGCCATGACATGATCATGTTGTTCTCTGACTTCCACCTTTTGAAACTAGGATCTGACACCTCTGGCATGGCCTCCTCACCCGTGAGATAATCATGCTTACCCTTGCCACAGATGAACATCATGACAGATTGTGACCACTGGAGATAATTGTGGCCGTTCAATTTATGACTGGTAATTAAGATCGGTGAAGAATCAATTCCACCATGAGTTGCAGTCTCCACGTTTCCTTTAGACTGTGATACTGTAACGGAGGATGATGAGGAAGCCATCCCGTATTTTGCTACAGAGAATACAACTGATGGGAGAGGAAGAAAGGTGGATACTGTTGAAAAAAAAAAAAGGTGGTAGAGGAGAAGAAGGAAATGGCGGCTGATAGGAGAACTAGGGTTTAGAGCCTAGCTCTGATACCATGAAGAAAACTATGAATTGGTTCCTCTTTTTCTTACACTCCAGAACAGTATGTACAGGGAATATATACAGCAAAATAGGAGAGAAAATAGGAGACTACATCTATCCTTTCCTAAATAATAGGAAACAAAATCTGGAAAGAATCCTAATGAATCTTCCCATTATAATTTAACTTATTCCTAAAATAGCCTAGATTACAAATAAATCCTAACTTTGAATCTTTATTTTCAACAGACAGTTTGTGAAGTCCCTACATAGATTGTGGGAAATGGCACGTACACACTTTTTATACATGTTTCAGTGTAGAGAAAATATCCATTAAAATGTGTAAAGTGATGCTCTGGGGTTTAAACCTTTGATCCATGTCATAATTGTGAAGTTCACTTGGTACACTTGTCCAGGTATGGATAACATATGGAAAATTCTCAAATTCAACCTTGCTATTGGATTTTGGTTTTTCCCTTCCATACAACAGCCACGATGAGGTTAGTTTTGAGTTTCAGTTTTGCTTTGGTTCCTGTTTGCAAAAGGAATGGTTATTATACAAACCAGATGGTTTTGCTTGCAAGCCATACTTATCTACCAATATTATCTGGTACCTCTTTGTTTGATTAAGAGCAGGTGGTTCTTTAGCCCCTTTTTTTTTAAGTACTGATTGCCTTTATTGCATCTTTTAGTCTCTCTTCGCATTTGCTATGCATTACTGTGTATGCATGGGATATACAGATGTGTCTAAAGGCTTGTTTTATGCATGTTACTCTGGGTGTAAGCTTTATCAAATGATGTTCACAAACTGCCAGGTTCAGATCCAGATAAAAGTACCTGATCACGATCCTCTACTTGAAGTGAAGTTGGAAGTTTTGCAAAGTCATTGCCTGCCAAGAGCCAGAGATGTCAATGGCTTCAAGTCTTCCAATGATTCTTTCACAATCAAGTTGGTAGCTAGTACTTTGTTCTGTATAAGTTTATTTGATATTCAGATTACATATTAATGAAACTAAGTTGCATAGAAATATAGTAGAGCTTTAGTTGAGGGAAAAAATGGGGTTAAAAAAAAGGGAAGAGTTTTCGATTTACCGTGTCTGAAGTGCCCATGAGGTGTGCTGCTACGTACTACTGAAGATTTGCACTGGACTGATGCTACAAAGTTTGCGAAAGACCTTAATTTGGGAAAAGTCAACTCAAATAACATATTGGGAAGCATCTACGTTGACACATTGTGAAGGGGGCCACTGGGATATCATTTAGAATCCTGCTTAGGATGTTCCTTTGATGACTGGATGTACATGATGGTCTTAATACTTCTTTTAATTCTGCATTTCATGAGGTCATTTAAAAGGCTTACAACTTTATCCTTCTGCAAGATCTTCTAATAACTACCTTTTTTTTCAATTGTGTTAAAGTAGTAAATTTATAAACCTATATTATTAATGCCCAGGTCATTCTGAGTTTTCTAGATTTCTTATATCTTCATCAATGCCGCAATGGGATGTGCCTTGGCTAACCATGTCTTGGCTAACCATGTCATCGCTGTAAATTTTCTTCTCTGATGACTTCATTATTCAATAGGGAAGTCAGATCTGCTAGAGGTAGAGGGAAGGGTCTTCCACAATCACTACGTGCGTTTGCTCGTGTTTTATGTTGCACTTCCCCTCAAGGTTACTTAAATCTTAATACTTTTGTTATATGTAGTTTAATATCTTTCAGAACTAGAACTGGTATTTCTCTTAGGCCATGCAACTCTCCACTTACTGTTTCTTGGTTGTAAAATATTCAGAACTGTGTGATTTGGCCACGGAAGCTGCACAAAATGATGGCCGCTTAGCCCGGCGTCCTTTCCGAAACAGCTGCCAAGAGATCCTAGCACATCAGATCTTATTATCACATATCATTCAATTAACCAAGGAATATAGTGCTTCTATTGAGGTAGGTACTGCACAAATTTTAACTGATTCATTGGTTGAGGCTACTTTTTCTAATTTCAGTAAACAATTTGGAGAATTAAACTCTATTGTCTTACCACAAAGTTTATTATGAACCAAAGGGAGATAATTTGCTACTAATTCTCCTGGAATTTTCAACATATGATTTGACACAGGCATTCAACATGATTTGTACAACTGTTATCAAATTTTTTAAGGTGTCATACTACTTTCTAAAAATGGGTAGATGTGTTAAATGAGTTATCGTGATTAGTAATTGTGATATATGAAGAGGTACAAGGTGGACATATTAAACCAATGAATATATATGATTCTTATTATACCAGAAATAAGATAAAACCTATCTGATTAATAGTAGTTATGTTCATTTGTGTGGTGAAGTTGCAGAGAGAATATTAACAAAATTTTGGTAGGATCTCTCTTGCACCAAATAAATTAAAAAGCGAATGGAATAAAGTTTTGCCTGTCTTTGTGAAAACATTGTTTCATTTCTTTTGACTTCTGAGATGTTTAGTGTGTTCACATCACACAGTTGCTGGAGCCTGTTACTTCTCCTTCAATTTGTAAAAGACTTGCTTTTCGGAAGCAAATGGCTCGAGATCTCCTTATTGGTGAACTTCGGATCCTCAAATCTGCTTCTGCATGGCTGGAGAATTACTGTGCAACCTTGGCATGAGCAGGCCATGATTGCTGATGATAAACTGATTGCATATCCACAGGAGCAAAGGCAAAAGGAAGAGTGTTGTATTTCTATGTTGTCAGATACTGATAGGCAGGAATCTGATTAAAACTGCAAGCAGAATATAAACAAGTGCCCACGCTAGTGATGGTTGGCAAATGCGGAAAGTGAGAGTCTTATCTTTGCTCTTCTAGTTCTGCATTCTTGAGTGGGTTCATCTATTTCGTTGAGGAAGCAAATGCCCCTATTCAACTTTATGAAGAACAAGAAGGATCTGTTGATGTTCTTTTGTCGCACTTCCTTCTCAAGTTAAACAATCTTCGAAATACTTGTTGCATAGTAAGCATACAAATTGCTTGCACGGCCTAGAGGTTTGGTCGGATGATTTTCGAGCCGTTTTCTCAATTTGAACATCCAAATTTTAGCCATGGAAAATAAAAAATACAAACTTCTACCGAATGATAAAAGTGAGGGAGTGACAACAAAATAATCAATGTTGCTTGTATGGATGTTCCTATATTGATATATATTTTTTTAAAGTTTGGATGATAAAGTAAAATCTCATTCAGTTACTTTTTATTAATTGAACTTAGGTGAGTGCATTTTATATCAGTTATTTTGGCAGACATAGTGTAATACTAAAATGTGAGACAAGTGTCACATGTATATGAATTATTTATGCAAGTTTTCTCACA

>CsSDG37

ATGAATTCTCGCCATCTACTTGCAACAAGTACAAACCTCACGGTAAGTTTTTTAAAATTACCCGTGTGAAAAAAAAGGAAAAAGAAAAAAGCTCGCACCTCACGCAAAGCTAGTTCTTCTCTCCTCTTCTTCTTCGGCAATGGTGAACGGCACGATGAACGGCGGCACAACTTCTACAACGGTGAACGGCGGCGCATGAACAACCCCCTGCCCCGCTCACTCAGTCTCACTCTCTCACCTGACGCAGGCTTATGGGCAAAAAAAATTCAAAATCCCTAATTACTAATTCCTCTCCTCTTCTGCTTCCACAACAGTCAACGGCGGCACAACTTCCACAATGGTCAGCGGCGGATTTGATTTTCAGGTATTTTGGTTAACTCTAAACTGATTACATAGCCTTTCAATTCTTTTTTTTGCTAATTTTTTTTTGGCGCACGCTGGACAATCCTTGCCATTAAACAAACCATCACAGCCACTAGCCACCACCGACATGCATGAATGTCCAATCTCTTAGCCGCAATCGATATTCTTTGTCGACGTCGTGAAGAAATTGGGTATGATGTTGTTAATCAATCACACATGCTACCAAAAAATGCTAAGAACCAATTTTTAAAATTTCGTTAATATGATTATGAAATTGGGCTTGCTTTTGTGTGAATTTTTAGGGTTTGTTTGCTTTTGGATGAACATTAAAAATCAGATTTTGAAATTGTTTGTTCCTCATTTTGTTGAGATATATCTTGTCTTCGTTTATTTATTTGTGCATGTCTGGATAGATATGCCAACCTAATCGAATGCTTATTAAATTCAATGAAATGTGATGGCCTTATGCTTGCGGTGAATAGCTTTATTCAACATATATATTTTGGAAATTAGTTTTATTTTGTTTGATAACATAATATGTGATAACACTTTAGTTTCTTGCAGCTTGAAAATCCTAAACTGTTTCAGATTCTAACTTTATGGAATAATCTGTTCACTTTGCAAATGATACGCAAAATTTTTTTAATCGACTGTTTTTTTATTTTTTTATTTTATAGCTTATTGTGTTAGAAGTTCTACTTGCTTTTGTCTTATTTTCCTATATTTCTGTTTTGTAATCTGAACTCCTGAATTAAAAATAAACTCTTGAATCTAAAATATAATAAGTTATCTTGATTAGTTTGGTGTGAATGTACCAGTTGAGTCGCGAGCATGGAGAAGCACAAGGGGGGAATCACTGGTATCATATTATAAAAATGCCATCTGTATAGAACTTTTGGTGTTCACTTCATTGGTAATTTTTCCCCCTTATTTTGCTCAATTCTTTGAATTATTAAATTATTCTATAGATTCCATTTGCAGATTTTTTGAATCACGATGGGCTTTCAGAAGCAGTTGTGTTGCATGATGAGGATAAACAATTGTCAGAGGTATATTCTCTCTTTTTCTCTTAATTTTCCAGCTCTGTGATGGAGACAAATAGTAGAATAAAATTCTGTTCTATATGGTCTCTAATGTTTGATAAATTGTTCTTTATTTTTAAAATCAATAGGAAAACTTGATTTAAAACTTAAAACAGAATTGGTCTGGACAAGATGCTTGTTCTCGTTGACCATTGCTCTTATTTCTGGTCCTAATAAAGTAGGAAGTCTTCATTATTGGAGCTAATTTTGTAAAATTTGGTAGGCTTGAAGGAAATATCTATTTCTAATATTAATTCTTTGAATTTGTTAGTGCAGGTTATTGCTGATCGAGATTATGCTCCTAAAGAAAAGGTCATATGCGGATTTGATGATTCTTTTCTTCTATTAAAATAATTTTATGATTACTCTCTGGAACTTACTTCTGTTTATATAATGTGTATTAGATGTGTATACTCAGTTTCTTGTGTTTTTTTTTCCCTGCTTGTCCGCCTTCTAGTGTTGTCTGGATATAGCTGTAAACATATATGTGGCCATCTGGTTAATACAAGAGCCTGAAATTTTTTTTCTTTACATTGATTAATAATTTTGATGTTTAGTGGCTCATCAATGGCTTTTCTTTCATCTTAAAGGTTGGCATCTGCATATCTAATAGTTGTATGCAGATAGAAATTGCTGCTTAAAAATATTTATATTTGTTATTGATTCAAGAATATATATATATGTTATGACCTTGGACAGTTTGTGAAGTCCCTACATAGATTGTGGGAAATGGCACGTACAAACACACTTTTCATACATATTTCAGTGTAGAGAAAATATCCATTAAAATGTGTAAAGTCGTGCTCTGGGGTTTAAACCTTTAATCCATGTCATAATTGTGAAGTTCACTTGGTGTAATTGTCCAGGTATGGATAACATATGGAAAATTCCCAAATTCAACCTTGCTATTGGATTTCGGTTTTTCCCTTCCATACAACAGCCACAATGAGGTTAGTTTTGAGTTTCAGTTTTGTGTTGGTTCTTGTTTGCAAAAGGAATGGTTATTATACAAACCAGATGGTTTTGCTTGCAAGCTATACTTATCTACCAATGTTATCTGGTACCTCTTTGTTTTATTAAGAGCAAGTGGTTCTTTAGCCCCTTTTTTTTTAAGTACTGATTGCCTTTATCGTGCATCTTTAAGTCTCTCTTCACATTTGCTATGCATTATTTTTTAAAAAAAATTAAAAAATTTCCAGGTGCTGTTTAGCTGCTGTTATGCTGCAAAAACAACACCAGGAAATTTACTGGCACTGTTTTACTAATGTTTGTGTAAAAAGACTTTAACTGCTCTTTTATATACCTATAATTTGTATTTCAATATGATCTCCATTTTGCTTTTATTTTTTAATTTTTAAAATGGGAAGATTCCCTTGTATTTTATCTCAATTGCAATTTTGTCTAGTATTCGTTCTTGGGATTATATCTTCTTTTACTTGTTTTTAGGGTGCAGCTCTCAGTTTCAAGTACTACCAATATTGTTTCTCTTCCATGAACGAATCTGTAGTTTTACCCAGCTATGAACTGAGTTAGCCATCGGTTATTAGCAGTTTCTGGACAAGGAGCAAGAAGATACCAATGAGGACCTGGATTTCAGAAGCAATTCTCTTATTTTTCTTTTATTTTGAATCATTAGCATTTTCTTTTTCTCGTTTGGTTTCAGATATTGGTGGCATTGTGTAGTGGGTTTGTTTCTCGAATCATGATGATACCAAATGTAATTGCAAACAGCAGGGTCTAAAATTTGTACGACCTTGTGATTTGTCGTAAATTTTTTTACTGATGTTTGCTTAATTTTATTTTCCTTTTTGTTCTTTTTTGAGTGATCTCCATAATTAATCAAGAACTAAAATATCAAGTTTGTTAATCTTTTAATTTAATAGTATTTTGAGAATTATTGGCTTCCATCTTGCATTAAAATAATCCCTCAATGAAAAATCATTGTTTTAACATTAAATTTAATTTTGCAATATGTAACTGACCAAAATAAAATTTTCAAAATTAAAAATTTTTGTGAGAAGAATTATAATGTTATATTTAGAGGTGGCAAAAATATCTGTCCGGACCGTACCCGGGCGTTGTGGGTTGGGTATTATTCGGTTCGGGTATGAAGCCGGGTCGGTCTTGGGCATCACTTGATAAAACGGCCAAGGCGGATGTACGGTACAGCAGCATATAA

>CsSDG38

TGGATGAGTTGCGATTATAATGTCCCAACCACATTATACTTCCGGTTGTTTTGTAAAAATTGTGTGAATATTTGCTTTTAAGGCCTCTCCAAGTGGCTCTGGTGGGCCATAAGTTGTCAGTGCATTCTTGGTTTTTTTAGTCCAAATTACTGTAATCATGCAAACACATGTAAATATACATCAAACTTTCCCGCGTCTTCTTTAAGGCTGTTTGGAATATATATTTCTTTAATTACATTATTATATTCGTCTGATGCATCCAAGCGGTTGTACTTTACTGAGCAGGGACTTGATTTTGCTGATGGTCAACGATTATGCACACATTATAAATTGTACTTATTTTACTGCAAATTATGGAAGTGCTTCCTCATTCTGGTGTTCAGTATGTTGGGGAATTAGATGCTAAACAGAGTTCAGGGACTGAGTTTGTTGATAATGGAGAATCTAACTGTGTTCAACATGAAAATCAAGTTCAAATGACAAATGGTAAAATGGATGACATGTTGTCAAATGTCGAAGGGCCTGTATCAGAAAGACGAGGTGAAGGTCAAAGAACTGGGGAAGAATTGCCTAGTTCAGAAGGACATCTAGGTGGAGTTTCCTATTTTGATTGTCAGCTGGAGGGGCAAGGATTATCTTGTGGTTCCCATGATTTCGAAGATGACGACGTGAATGCTCAAAACGAGTGCACAGGGCCTTGCCAAGCCTCTGAAAACTCCAACTTAATTGTTGACACTATTGAAAGTGAAGTGCCAAATGACAATAAGGAGGGAGAATCATCTTTTTCAGAGCCCAAGTGGCTAGAACATGATGAATCTGTTGCACTGTGGGTCAAGGTAAGTTGCTCATGTTTACATCCTTCACTTCTATAAAAAATGCTTTTTGTTAGTCTATTTGTTTAGTTATATGTGCACATGATGTACTGGTTCTTGTGCTTTCTTTACTCTTTGACTGTGTATTTCTATTGAGGCTTAATTTTGAATTGTGCCCTGAATTTGTTATCATTTTGGTACCTTTGTGTTTTTTCCAAGTGTAGTGGAGGGGGAAGTGGCAGGCTGGAATCAGATGTGCTAGGGCTGACTGGCCACTACCAACTTTGAAAGCGAAACCAACTCATGACCGGAAGAAGTACTTTGTGATTTTTTTCCCCCACACCAGAAATTACTCTTGGGCAGATATGCTACTTGTCCGATCAATTAATGAGTTTCCTCAACCTATTGCATATAGGACGCATAAAGTTGGACTAAAAATGGTTAAAGATTTGTCTGTGGCACGCCGATATATAATGCAAAAGCTATCTGTTGGCATGCTGAATATTGTTGATCAGTTTCATTCTGAGGTCTGTTTTTTGGCGATTGTTTCTTTTGTGATTCTTTGTTCTCTGATGTGAATTTTTGCCTTTATTTTACTTTTCATCCTCATCATATGTGATTTGTTCAAACTTGTCTAGGCTTTGGTAGAGACGGCTCGTAACGTGAGTGTTTGGAAAGAATTTGCAATGGAGGCTTCCCGCTGTGTTGGTTATTCTGATCTTGGAAGGATGCTCGTGAAGCTTCAAAGTGTATGTCTCTGACCCTTCGAGAGTTGGTGAAATTGGAACAGCAATTCTCTAATTAAAGTTTCTATATATTGATAGATAGATAGATAGATAGATAGAATTTCTGTTCTCACCCACCCCATCTCCAAATTAAGCGCATCCCCTTGAAATTGAACCCTAGACCTCTTACTTCAATTTTAAGCGACCAACCAAGTGAGCTATCATAGATGATTATTTACCTTCTAGATAATTTATGTGTTTTTGTGTACTGTTGCATTAATTTGAGCTGGGTTATACTTATATCAACTCTATCTGTAGCTGTGTTTTCACTTCACATTTATTTCTCTGCAGATGATATTACAGCAGTACATTAATTCTGACTGGCTACAGCATTCTTTTCCCTCTTGGGTACAACGCTGTCAAAATGCACGTAGTGCTGAATCCATTGAATTGCTGAAAGAGGTACATGTCACCGTTGACCAGCCATTTAAGTTAAATGTCAATAGATTGTGATCAAGGCAATAAATCAGATAAGCAACGTAATTGGAAAATGAAATTCATTTTCGTATATAGAAACTTTAGTTTTCTAATAAAGTATGCTCACCAAAGTAATTCTGGCTAGCTACAACATTCTTTTCACTCTCTGCAATGTAACATATTTATGTTCAGTCTTTTAATTATCTAATTTTCATGCAAGTTGGTCTAATTTTCTTGCTAGTTGGATGATGAGTAGATGTAATACTATTAACTAGTTGCTGAACTGGTGATATACTGTAAATTATTTCTTAAACGGAGCTGGTTCTGTTGTTTTTGCCTTGTGAGAAATGTGATGCAGGTAATATAACTTATAACAAGTTTTCTTGTCATGTATGGCAAGTTATTGACAGTTATAGTATGAATTTAGTTGTAAATATTTTGCTGATATCTTTATGCCCTATCTGATTCAGGAATTGTATGATTATATACTGTGGAATGAAGTTAACTCTCTCTGGGATGCACCAGTGCAGCCCACACTGGGTTCTGAGTGGAAAACTTGGAAGCATGAAGTTATGAAATGGTTTTCAACATCTCATCCTCTTTCTAATGGTGGCGACATGGAACCACGGCAAAGTGATGGTTCCCTGACCACGAGCCTTCAAGTTTGCAGGAAAAGGCCCAAACTTGAAGTACGTCGTCCTGATTCACATGCTTCCCCATTGGAAAACAGTGATTCAAATCAACCTCTGGCTCTTGAAATTGACTCGGAATATTTTAATAGTCAAGACACAGGAAATCCAGCTATATTTGCATCTGAGCTTTCTAAAGGACCAGGTTTGAGGGAGGAAACTGCACAAACAAATACACCAAGTACTGTGAGTAACAGGTGGGATGGAATGGTTGTTGGAGTGGGAAATTCTGTGCCGATCCATACCAAAGATGTTGAATTGACCCCTGTGAATGGAGTATCTACAGGTCCTTTTAACCAGACTAATATGGCATTAACTCCTCTGAATGAGTTGGTCACTAAGAAACCCTTAGAGCTTGGGCAGAGGAATCGCCAGTGTACTGCTTTTATTGAATCAAAGGGAAGACAGTGTGTGAGGTGGGCAAATGAAGGCGATGTTTACTGTTGTGTGCATTTAGCCTCTCGTTTTACAGGTAGCACTACAAAAGCAGAATGTGCACTCTCTGCTGATTCACCTATGTGTGAAGGTACTACAGTCCTTGGCACTAGATGTAAGCATCGGGCTCTATATGGCTCTTCATTTTGTAAGAAACACAGACCAAGGACTGATACAGGAAGGATATTGGATTCACCAGATAATACACTCAAGAGAAAACATGAGGAGACTATTCCCAGTGCAGAAACCACATCTTGCAGAGACATAGTATTGGTAGGAGAAGATATCAGTCCCCTCCAAGTAGATCCGCTCTCAGTCGTGGGGAGTGATTCTTTCCTAGGAAGAAACAGCTTAATTGATAAGCCTGAGCATTCTGGTAAAGGTTATAGTGCTACTGAAGCGCAGCATTGCATAGGCTTATACTCTCAGAATAGCAGCAATCCATGTCATGAAAGTCCAAAGCGGCATTCATTATATTGCGATAAACACCTTCCAAGCTGGCTTAAGCGTGCAAGGAATGGTAAGAGTAGGATAATATCAAAAGAAGTGTTTTTAGAGCTTTTGAAGGATTGCTGCTCACTTGAGCAAAAATTGCATCTGCACCTAGCATGTGAACTATTTTACAAGCTCCTGAAGAGTATTTTATCGCTAAGAAATCCAGTTCCTATGGAAATTCAATTTCAATGGGCCCTATCTGAAGCATCAAAAGATGCTGGTATTGGGGAATTCTTAATGAAGTTGGTATGTTGTGAAAAAGAGAGACTTAGTAAAACATGGGGTTTCGATGCCAATGAAAATGCCCATGTTTCCTCCTCTGTCGTGGAAGATTCAGCTGTATTGCCATTGGCAATTGCTGGTCGCAGTGAAGATGAAAAAACACATAAGTGCAAAATTTGCTCACAGGTGTTTCTCCATGACCAAGAGCTTGGTGTCCATTGGATGGACAATCATAAGAAGGAAGCACAATGGCTGTTCAGAGGTTATGCTTGTGCTATTTGCCTAGATTCTTTTACTAACAAGAAAGTTTTGGAATCCCATGTGCAGGAAAGACACCATGTGCAATTTGTTGAGCAGTGCATGCTACAACAATGTATTCCCTGTGGTAGCCATTTTGGGAATACTGAAGAGTTATGGCTGCATGTGCAATCAGTTCATGCTATTGATTTCAAAATGTCAGAAGTTGCTCAACAACATAATCAGTCTGTTGGTGAGGATTCTCCAAAGAAACTTGAGCTGGGATATTCAGCATCCGTTGAAAATCATTCCGAGAATTTAGGCTCTATTCGAAAGTTTATTTGCAGGTTTTGTGGCTTAAAGTTTGATCTGCTACCTGATCTTGGCCGCCACCATCAGGCTGCTCATATGGGACCTAACTTAGTCAATTCTCGGCCCCACAAAAAGGGGATTCGGTTTTATGCTTATAAATTAAAATCTGGTAGACTTAGCCGTCCTAGATTTAAGAAAGGTCTTGGGGCAGTGTCCTATAGGATCAGGAATAGGGGTGCTGCAGGTATGAAGAAACGCATCCAGACTTTGAAGCCACTTGCCTCGGGGGAGATAGTTGAACAGCCTAAGGCAACTGAGGTAGTGACACTTGGTACATTGGTTGAATCCCAATGCTCAACACTTTCAAGAATATTGATTCCAGAGATTCGGAAGACAAAACCTCGGCCCAATAGCCATGAAATCTTATCCATGGCTCGCTTAGCTTGCTGCAAGGTGAGCCTTAAAGCCTCACTAGAGGAGAAGTATGGAGCGCTGCCAGAAAACATTTGTTTAAAGGCAGCCAAACTTTGCAGTGAGCATAACATTCAAGTGGAATGGCATCGAGAGGGATTTCTATGTTCTAATGGATGTAAGATTTTCAAGGACCCACATTTGCCTCCACATTTGGAACCTCTACCCAGTGTTTCTGCAGGAATACGATCTTCAGATTCATCAGATTTTGTGAATAATCAGTGGGAAGTGGATGAGTGTCATTGTATTATTGATTCACGTCATTTAGGGCGAAAACCCTTGCTAAGGGGCACTGTCTTGTGTGATGATATAAGCTCGGGATTGGAATCGGTTCCAGTAGCTTGTGTAGTGGATGATGGTCTTCTAGAAACCCTATGCATCTCTGCTGATAGTTCAGACAGCCAAAAGACCAGATGCTCCATGCCTTGGGAGAGTTTTACGTATGTTACAAAGCCATTACTTGATCAATCCCTTGATCTTGATGCCGAGGTACCTGTTTGTGGGTTATGCATTTTATTATTAATGCAATTTCATTTGGATCCTTTTCTCTTTATTTATACAAATTGTGACACTTATCTCATAGTGCTTGCACAAATATGCTTTCCGGCACATGCATGCACTGTAAATTCATGTTGCCAATACAAATGAATGAGCTAGTTTGGACACTCATCTTCTGTTTGCTCTCTTTTTCTTCTTCCTGCAGAGTTTGCAATTGGGATGTGCCTGTGCAAATTCAACTTGCTTTCCTGAAACATGTGATCACGTATACCTCTTTGACAATGATTACGAAGATGCAAAAGACATTGATGGGAAATCCGTGCATGGTAGATTCCCATACGACCAGACAGGAAGGGTTATTCTTGAGGTATTGTATTTTATTTTATGATCATCAAATTTGGTAGTGTCCTTTTGCATAGCTTATATATATTTGTGTGCTCTTTGTCAGTAGATGCCTGGATTTCCTTTCTTCTTGCAACTTGTAAGTCAACCTGTTAGGAGATAATTTGGCAGAAAAATCAGGCTTGCTGGTGTTTTTCTTTTTTCTTTTTTATTATCCCTGTAGGTTCTGTCAAGCATAAAAAAAGCATTGACTTAGTTTCTTTTTTAAAGGAGCACTGACTTGATTTGAGTTTGCAAGTGTCCAACATTGGTTTTGTTTAGATATCTTTACTGTCCAAAACATGTACCAGAAAATTCTCAATGTTTGATAGTTGTCCCATCAGCTATGTCCTATCAAAAAAAACTCACCAAGTTATTGACTTTGTTTATCTTTGTAATTTGAGTTGTCATATCGCGCAGAAATTGTGCGTAAAAGGTGGGAATGTTTGTCCTGGTAACTCTGCTCTCTTTCAGTCAGGTCTTTTGCTGTTAATACTGGAGTTTAAACAGTTCTGTTGCAAAATGTGCAGGAGGGTTATCTTATTTATGAGTGCAATCACATGTGTAGCTGCGATAGAACCTGTCCAAATAGAGTTTTGCAGAATGGAGTACGAGTGAAATTAGAAGTCTTTAAAACAGAGAATAAGGTAATGCTTTCAATTTTCATTATTTTCGAGAGCTTTATCTGTGGCCTCACACGTGTGTTAATTTATAGGGTTGGGCCGTTAGGGCAGGTCAAGCAATTCTGCGAGGCACATTTGTATGTGAGTATATAGGGGAGGTTTTAGATGAGCTGGAGACAAATAAGAGGCGTAGCAGGTTACTTTTTGACTAATAAAGTTTCATTTCTACCATTTGTTAGTCTTAATGTTGTACATGGTAAGTAGTTATTATGATTGTTCAGGTATGGTAGAGACGGATGCGGTTATATGTTAAATATCGGTGCCCATATCAATGATATGGGCAGATTGATTGAAGGACAGGTCCGGTATGTCATTGACGCCACTAAGTATGGAAATGTTTCTCGATTCATCAATCATAGGTGAGGGAAGGCATTGTCTGTGTTCTACTTTTCCCTGTCTCTCTTCTCAAGTATGTTCTCTTACACACCACCTTGGGCTTTAATTGAATCTATACTTGGCTTTCATGCTTGACTGTAAGGGTAGTAATGCTTTAAAGTACTATGAGGTTTATAATTTTAATGAATAAGCATATATTTCATGTCCAGTGATTGTGACGAAGAGAAAAATATGTATTAGCTTTTGATTCTATGATAGCCCAGCTGTATGGATAACTTCATTAAAATCTAATAAACATTACTCAAGCTACCCTTTAGTAATTTTGTTTGAAGCTGTAAAATTGTACTTTTTTAGGCGGTGGATTATGGAGATTGTTATGTTCTCATGGAATAATGCATGATTTTAAGCTAATTGAGAGTCGAGGTTGTTTAGTGAAGTTGAATTAAATCTATTTTTAATTGATCTGTTATTTGCCTTGCTCTGGGTGGCATAGTTTTTTTCTGAATTACTGATTTTTGTTGGTAGCTGCTTTCCGAATCTTGTGAATCACCAAGTTCTCGTGGAAAGTATGGATTATCAGCGTGCCCATATTGGTCTCTATGCAAGTCGAGATGTAAGCATTCTCCCGAGCTTCTATGCTATTAATTTGTCACTATAATACAGAGGTTCAGACAGGTTTGCCCATAATTTTATATGGATCTTTAAGATGATTGATTCTGATGGGGTAAAAGGTAGTGTTACATGGATTTTCTTTGATCCCAATGATCTAACCGAGCTATCCCCTTGAAGACAACTTGTTCAAATTTTTAGCATTTCAGAGTCTTGCCTGGCCTGGCATGGGCATGATGCTGCAGGAGTTCATCTTTTTCTGTTCATATAACTTCTTAATTGCATCTACCAGCAAACTGTAGAAGGAATTAATGGTCCAAAGTATTTCCTTGTTCTAAATATAGTCATTTCATGTTATAAGCAGATAGCTGTGGGTGAAGAACTGACGTATGACTATCACTACGAGCTTCTGTCTGGAGAAGGATATCCATGCCATTGTGGAGCTTCAAAGTGCAGGGGACGCCTTTACTGAATCTTTAAAGGACATGATTGTAGTCTCCCCAGCATGCAGAAGTTTTGTTTTGAACTTTGGCAAATAAAAGGGGGCTATATAAGATTGAAATATATGTGACAAACTGGTCAAACTGGCCGGTTATCAACTTATTGCCATAGTAGCTGCTTACAATATGATTGCCAAGCAAGTCTTCTTGCACAGAGGAAAATTCACCAGCAATGGCAATGGGTTTTCTGTGCCACCTGATCACGTGGTTTCTTTCCTCAAGTAGATATGATACCCAAATGCTCTTTTGTGTACTCAGCACTTGGAAGAAAAGAGGATGCAAACTCGTTATTTATTTCGGGATTTGTTTTCTTTCTGTTTGTAGTTTGTAGTTTGTATTTTGTAGTTACTTGCTTAGATTAGCCTAGAGGAACAGAAGGTAGTTTTTGATGGCCAATGTTTGAAACTATGATTTTTCATGGCGATGCTGTTTTCTCCTTCATCACTGTATAAAGGTTCTTGCGACCATCATTCTGCTTCTGTATTGCATTCTCACATAAAACGGTAACCGAACAGAAACAATTGGAATTAGAAGATATTGGGACATTGGAGAAGTACATAATAATGTTCATCCGCAATTCGCGCTCCATATATTAATAAAACCTGATGGATCCTTATTACTTCTGCATTGATCATAACTTAAAGTTGTCCTTTTTCAACTACAAAGAAGATGAAGGGGAGTCCATAACTGAAGCCTACCAAAACCGCCATCACTGCAACCCAAAATTCACCGGTTACTATCAGTGCCGGAGGAAACTATGTTCCAATAAGTATAATACATATTTAGAAGAAGAATAGGACTAGTTTTAGTATCTCACCAACGTCAATAAAACTTGAAATTTTCTTGTTCTTTCTTATTAACAGATGCAGCTTCAAGATGCAAAA

>CsSDG39

ATGGAAATTGTTTGCCCGATCGACGAAAAGTGCGCAAGTGAAGTCTCTTGTCTTCTGCGACCTCCTTCACCTCTTCAAGTCCAGGCAACTGTGCTTCTTTATCTGCAAACTTATTGGCACCTTTTTAATTTTCTTATTGACGAATCCTTTTGATTTTCAATCGCCAGGAGTACTTCGACCAGCTTATTTCCACTAGAAATTGCCATGGCATTAAAGTTAAACAAATTAGTGAGCGTGGAAAGGGTATCTAAATTGAATTTTTTTTTTCTATAGCCTATCTTCGCTTTTTAATGCGCTCTTTTTTCTGTTCTTTTTTTCTTTTCGTTTTTTTTCAAATTTAATTTGTCTTGATCGCAATTTGTTAACTTTAGGTGTCTATGCTGGCATGGACTTTCAAGAAGAGGAACTTGTTTTGAAGGACCAAATGCTTGCCGGAAATCAGCATTCTTCAAACAAGGTCTTTTTATTTTTATTTATTCATTGTGTTTGCTTGCCTTGAGAGTTGAACATTTATGGCATAAATATATATTTTTATTTTATTTCCATGTTGCAGATGGATTGTCTTGTGTGCAGCTTTTGTTTTCGATTTATTGGTTCTATAGAACTTCAAATTGGGCGGAGACTGTATTTGCAAAGCCTAGGTGACTCTGCAAATGATAAATGTCATATGGGGTCCTCTTCACATACATCGGAAGATTGTTACAATACTGATTCATCCGACATGGAGGACGATTCTTACATGAAAAATCATGAAGATTATGGAAATTGTGCTCCTGGCAGTTCCAAAGATAATATCTCTCTGCCTAAAGGGTTCATTGAATCATTGATGAATGGTGAACTGGAATTACCCTTTTCTGACAAGTTTCCCTTGCCTTCCACCATTCCATGTCCTGGGGGTTGCGGAGAAGCTTATTACTGTAGGTAAGCATGTTAATTGTTGTCACCTCAACAATACGAACCATGAGTACAATTGACTTTTACCTCAACAATACAAACCATGAGTACAATTGACTTTTAAGTGAGTACTACTGAGACTTCTAAAGATTTTCCATTTAATCCCTTGTAATAATCTTGTCAACATGCTGCAGCAAATCTTGTGCAGAGGCTGATTGGGAATTGTTTCATTCTTTATTGTGCACCGGGGAGAGGTCAAAAGCACTATCTAGGGCAGCGTTATTGAAATTTATAGAACACGCTAATGGTAAAGCTCTAGATTTGTTGGTAATTGCCTCTTAGCATGTACTTAAACCAGGTAAAATGTTCACAGTCACTGATTTCTGCTTCGCTACTGGTTCCTTGTTCCTCTTTTAAACCTCAGGAACAAATGATATTTTCCTTCTTGCTGCCAAGGTATGTTCTGTTGTATATATTTATTGTCCATATATGTTATTTACCATTGCACACAATACAATACTTCAACTACCTGCAATAGAACATACTTTTAACGTTAGTTCATTTTCAGGTGATTTGTTCCATCATTTTAAGATATAGGAAGCTAAAAGCCGCTCATCTTGAAGAACAAGGAAAAACCAATGCAAATTCGAAAAGTTCTAATCTTTCCTTGCTTTTGGAAGCTTGGAAGCCAATATCCATTGGATACAAGAGAAGGTAGTTGCTAATGCTTCTTCTTTATATCTTGTCTAGAGACATTATTTTACTTGTCTTAGGTTAGATTTACAACATTCTACGTTTATGAATCTGTGTAGAAGGTTCTAAATGGAGTGTTAATGTTATGTCAATCTCTAGTTGCTGCAACATCAAAACAGTATTAGTGTTTTATATGATTTCTATACTGTTGCGTGGTTATAAGGTATGATTTTATTTTTTTTTCTTTTTGGGGAGGGATGAGAAGAATTTTTATAAATTGCTTTGTATCAGGAGATTCACTAGCACTTATCATTTGCTTAGGTGGTGGGACTGCATTGCTTTGCCAGATGATGTGGATTCTTCTGATGAAGCCTCTTTCAGGATGAAAATAAGAGAGCTTGCATTCACTGTAAGACAATTTTATAAAGATGGTGCATGCATGAAAAACCTGACTATACATTTCTTCTGATATGTCCTTTTAGTTATGTGTTCAAATACTGTGGAAACATTGTACATAGTGGTTGCTTATTGCTAAAGCATATCATAGTGAGAAACTAGGAGATTTTATTAGATTGAAGAACTAGAAATTATTTGACCCTTTGGAACAATTATTCTCATCATTTATTGCTTTTTTCTTCTTAGAGTGAGGGAACTTGCTTTCTTGCTATTTATTTCACCATAATTGAAGGTAGATCTTTGTATCCTTAACTTTGACATATCTCACTTTAAATTCTATAATTGAATTGAAAGGAGGTAGCCTTGGTCTGGTTGCTTATAAAAATCTTGTAATGTTTATCATGTTACCAAGTACATTAATTAATCCCCTCGACTTCTTGTTTGATGCATTTTCTTATAATTATTAACTTCTCATCTTCAGTTTCTTGCATTAGTATTTGGCTGTTATAAATATTAGATAATATCAAAGTTGCTTGTTATCATGTGTCTAAATTTTATCCTTGTGAATTGTTTCAGTCGCTGCAGCTCCTAAAGGCAGCCATCTTTGACAGCGAGTGTGAGCCATGTATCCTTTTCCAAAAGTTCTTATTGAATTGGCACACTTCTTTGAAGTACTGTTGCATTACCATTCTCAGAATTAATAAGTTGAATTTCTTGACTCTACAAAGTATTCTCCCTTGAAATCTACGGACATATTATTGGCATGTTTGAGCTAAATAATCTGTAAGTCACTTTACGAAGGCTTCTCAGGGGACATATTTACGTATTTAGGTAGTTTACTACTCTTGCAGGAAGAATGTTGTTACAACAGCATATTGGCATGGACTATATAGGCTCTCATTTTCAAACGTTTATTTAATTTGTCTTCCAAATAACAATTTGCAATTGTAGACAATGTATTTAATTTTGGCTTCCAAATAATAATTCAAAGTCCTAAATTTACAGTGATTTGGTTGTAGCATCTCCAGTAGAGGATTACTTTCTGTATATTGATGATCTTCTGCATGGCGAAAAGGTTAGAATGAATCTTGTGATTTTAGAGTCCTTTCATTTTTGTATGCGACCCTTCCTTTTATGGTTTGTTGCAGAAAGAGGCTGAGAAAATTACTCGACCAATCCTCGATGCTCTTGGTGATGACTATTCAATTTGTTGTCAAGGTATAAATTAAGCTGCCTGACAGATCATCATAATTGGAGAACAATTTTTCAGTTTATTTCCTTTTTATAAATGCTGAACAACTTGTGCGAAATGTGTCCGTAATAGAAAATGCTTTTTCTTGTATATGCTGTTTATGTAAAACAGATTTTGTTACATAAGATATGTTAACTCTTGCACTCCTTGATATATTATTTTTTCTTAAAATTTTCCTCAAATGGCAATTACTAATGCTTTGCATTTATTTCCATATCTTAATGAAATGAGATAACCAAGATACCAAAAATAAGACTTGTGCTTTGTTTCTTCTGTCCCACACTTGGACTATAGTGTTCTAGTTGACTTTTGATGATACTTGGTGTGGCAAACTGAATTCTTTCTTTCTATCCTTTTTTAGTTGGCTAGTCATATACAAACTTTATTGTGAAAATTTTTATATTTTTTATTACATAAATTTTTTCTTAATGAGCCTTTTTTCCCCCCCTATTTTGAAATGTTTTAATGCATCAGGGACTGCATTCTTCCCTTTGCAGAGTTGTATGAACCATTCCTGCTGTCCTAATGGAAAAGCATTCAAAAGAGAAGAGGTAATTTAATCTTCCTCCAGATACAAGGTGTTTGTTATTGGTCTCAGTGCAGACACTGAGATGCAGGCAATTTAATTAATTATTTATATGTGAAGGATTCACTACATAGTCCTTCTCACCCAACTAATTGTTAAGTTTCCAAAGTTTACAATTATTCTGGCTTTATGTGTAACATTGGCATATAGGAACGTTTTATTTTAGTTTCTTTCATGATTCTAGACTATATCTCATCTACTTTTTTGTTCTCATTTGCAGGACAGAGATGGCCAAGCAGTTATTATTGCACAGAGACCCATTTGTAAGGGAGAAGAGGTAAGCCAACTCTGTTTTTGTATATTACTTCGGAACCTCTCATGGATTAAAAATCCCCAACTTAAGATTGCAACTTCTGGGGTGTGGTGTGGAGTTATTTTATTTCCATTTGTCTTGAGCAAAGAGTATTTAAATTTGGCATTCCAGGTTACCATTTCATATATAGATGAGGACCTTCCTTATGGAGAGAGGCAGACATTACTTGCAGATTATGGTTTCAGATGTAGTTGCCCCAAGTGCTTAGAAGAAGAGCCATGA

>CsSDG40

ATGCCAAATCCACAATTTTGCAAGATTGCAGAGTCGTGCGAGGAAAATGAAGTCGCCGATGAGTATGCTTACGTAGCAAATCCAAAGAAACGAAGAAGAAGAGGAGGCGACGATTGTCATCAAAGCTTAATGCATGTTGAAGTCGATGATTTAAGCAGTGGCTCAAGTTCTTTTATTAGCGAGGAAGCCACATGTTGGGACCCCGAATTTGAACCTGATTTGAACAATTTTAATTATAAAGGAAGAGGCACAAACCGGAGCTCCGACAGGTTTCGACCTCCAGCATTGAAACCTTCGAAAGGAAGGACTCAAATACTTCCTTCCAGATATGATGATTCGGTTCTTGTTGTTGGTGACACGGATTCAAGCTTTGACGAGGAAGATGATGTTGATATTATAGAAGTGAACGGGGATTTTGATAAGTTGGGTTTTACCATGGATAAGTATAGATTTGGAAATTCAAATTATAGAGGATATAATGGTTTTGATCCGAGAGAGTATTTGGTTTCGCGTCGCCCTGTTATGCCGGCAGGTAATGTAAATTCTTTGCCAATGGCGGGTAAGAAACAGTTTATGCCAGGGTTTAGTTCTAGGAATGTAGAGAGAATTACAAAAGAGAAAGAGAAAAAGAAAAAGAGGAAGGATGTTTACAAGCCTGAGGATTTTGCTCTGGGTGATTTAGTTTGGGCTAAATGTGGGAGGTCGTATCCAGCTTGGCCTGCTGTTGTAATTGATCCCATTTTGCAAGCGCCTGAAGCTGTGTTGAGGTGCTGTATTCCTGGCTGCCTCTGTGTCATGTTTTTTGGCTACTCAAAGAATGGAACACAAAGGGTATATAAGTCTCTCATGTTTTCATTTTGTTGGTTTCTTTTGTTTAATATATGGTCATCATGTTTTCACTTAAATTGTTTGTAATGATGTTGATGGGCAGGACTATGGATGGGTGAAGCAAGGGATGCTATTCCCTTTTGCGGAATTCATGGACAAGTGTGTATATTTCCATTTTTGGATGATTGCACAATAATATTTTTAGAGTATAAAGGGTTTTCTTGATTTTCTGTTTGTTTAGATTTCAGGAGCCAACCCAGTTGCACAAGAGCAAGATCAGTGGTTTTCAGATTGCCCTGGAGGAGGCAGTTTTGGCAGAAAATGGTTTTTTGGACTTGAACCTTGGTATTGGGCAAATTGGTCCAGAAGCTTATTCCCGCAGAGGCCAAGAGGCCACGGGTTCAGGTCAGGATCTGGAATACTGCCCTCAGAATCAGGTAAGTTCTTCATAATTTCACTACTTTGTGGTGCATTGAAATTTCACGGATGCGTTGCAAGGCTCTAATCACTATTCAGCAAGATTCACATGCATATGATTAAATTAAATTAGCTTTGGTTTTTCTGTCTTGTTGTTTACCATAAAACTGTTACTTAAATATTCAATTCTTGGATGATTTGCACAGAAGAGGCTTGTGTAACATCTGAATTAAGTTAATAAATTAGGTAAACTTTTCAAAGTACTGCTACACAGATGTACATGTTGCATGCAGTTATGAACTGAATCTGCTTTTTTAAATTTGTGGTGATGAATTGAAAGTTTATACACTAGCTTAGGTTTACCATCCATCTACAATGTTATATCCTTCTGCATGACTTGTTTGAGGCTGTTCTTTTCTCACTCTCCTCTTACTTTGATATTCTGGCATTTGAATTATTTTTGTCTTGTTATCAGGTGGTGCTGATGGTACCATTTGATATATTCTTTGTGCTGTTTCTGGCCCTTTCCCATTGCTGTTGCAGTCCTACTTCAAGTTGGAGGATTGCTTGTACTTTTTAAAATTTTTCATGTCTTGTCGTAGGCGCTAAAATCCAATCTTAGTCACTTTTCCCATCGTGGAATTTTCGTGCAATGTTTTCTAGTGGATTAGTGGTTCTCATCCTGTGTGATAGTCACTCTACTAGACTGTGCGGGGACTGTACCCGTTCAATAATATTGTTTGTGGTTGTACACACAAAATTCTGCTTTCCTTAACACTTATTTTCTAACACTTTGGATGTTTGTGTTTGTATTTTCAGCTGTTACTTTGGGTTATTGGATAATATATTTCCGTAGGTAGTGATCAGATAGTTTTGTTTTGTGTTTCAAGAATTCATTTGTTTAATCAACTGCTGCAGAATGCATGTTATAAGGTTGCAAGAGTGTGTGACGGTTGTGGCTTATTTCGACCTTGCAAGTTGAAGAGAATGAAAGGCTTAGTATCTGAAACCCAGTTTTTATGTAAACATTGTTCTAAGGTTCGTAAGACTCTGTTCTTGAATTTTATTTGACATGAATGTAGCTTCTATTTTTCTATTGATGGAAGAAATAGATTAACTGTTTTTCTTAACATGATCTCCACATTTATTCCTTTTACAGTTACAAAAATCAGAGCAGTACTGTGGCATATGCAAGAATATTTGGCACCATTCAGATAGTGGGAATTGGGTGAGATTATTCACACTTCCGGTGGGGCTAGTATATATATATTTTTTTGGTGGGGGTAAAAAACACTGAGAAAATATTGTTTTACTTCTCTGGAGCAGGTATGTTGTGACGGTTGTAATGTTTGGGTGCATGCTGAGTGTGACGAAATTTCTGGCAAACATTTTAAGGTACACCTACCTAATTTGTAATTTTATTTTGTTTTTATTTATGTTATTCATTATGTTCATGGAGTTTGGGGGTTCACTGACTTGATTATGCTGTGAAGGATCTGGAGCACATTGATTATTATTGCCCGAATTGCAGAGTAAAGTTTAAATTTCAATCATCAAATATAGGAAAATGGCAGCCAGGTGTCAGGTATGTTAACTTAATCCTACAGACATCTGATGTTTTACTTTGGCTATGTATTATGGATGTTCATTTGATGTGAATTTTTAAACATTATACCAAACAATATTTTTTGACAGTGCTGTAGAAAATGACGGGCAAATGGTGCTGCCTGACAAGATAATGGTAGTATGCAATGACGTCGAAGGAGCATATTTTCCAAAACTTCATTTGTAAGCCTCAGAACTTATTAATGTCCTTGATAAGTTTAATAAGACCGTTATTGTTTTTTTCTTTTTGTTAAAAGAATCAAATTGAAAAATTCTGTTAGGGCTGACATTTTCTATCCTCCTCCCCATTCTTTCTTTCGGAAAATGTTTGTTCACACTCTTTATTTGGAAATACTTATTAATAGCCACACATACATTGTCAACAAGTTATTTGATTATTTGACTTGGTTTTTTCAGTAGGGAATTCTGGTTGCACTACTTTGGTTTATGGCAAAGTTGTTTTGAATAGAATATTCTAAAAAATAGCTGAGCTAAACTGATGTTTTGCCCTTTTTCCTTTAGAGTTGTGTGTAGGTGTCGATCATGTGGGCCGAAAAAGCTAACACTTAGTGAATGGGAACGCCACACTGGCTGTAGAGCAAAAAAATGGAAGTATAGCGTGAAAGTTCTGGGCACTATGCTACCACTGGGGAAATGGGTTCGTATTGATTCTTTCATCTTGTTTTTATAATTCTTTTTATCTGTATATTAAATTGTTTAGAACTTTTGGTCATTATTGGAGGCTATTGGCTTAATGTATTGATGATTAAAAGAAATCACAGCTATCTACTATCTTGGTTCTCAGTGATTGTTGCTCATAAATGTAGTTTCCTTCCTTAAAGCCATAGCTTGCTTCATCAGTTTCCCTTCCAACTTTAGTGAATCAGCAAGAAATGTAATTACCTTATCTATAGTGATTGCTCCTATCAGGGAATATTGGATTATTTTTGTGGACAGAATGTAACCCTGCTCCCCTCTTCCCTTTTCTATGTTTTATTTTCTAATTGTAGACATGTGAACAATTGAACATCCGGATAGCCATCTGATCTGTCAATTAACAAAACATTATGGCTATATATGTACATATTAAGTTTTGATTCTTAAATGTATGGTTTTTATTAACATCGAAGACAATGCAGATCACTGAATTTAATGCTGATGCTATGGATCCAGTAAAATTAGATGAGAAAAAGCTACTTGCTTTTATGAAAGGTAATAGGCACATCCTTGAAATATTTACATGTTGATATTATTATTATTATCATTATTGTTGTTGTTGTTGTTGTTGCTTTTGTTTTTTTTTTTTTTTGTTCGGGGGGGGGGGGGGGGAGTTGGGGTGGCGGGAGGGTTGATGTGGTGTTCTTAGGGGTTTCAAAAATTTTAACTAGAAATTTTTGTGATGTTTCAGTATTGGGAAACTTACATAACAGTGTTTTATTGCTACAGAGAAGTACGAGCCTGTTTCTGTAAAATGGACAACTGAAAGATGTGCTATCTGTAGATGGGTTGAAGACTGGGACTACAATAAAATTATTATCTGCAATAGGTAGGTCATTATTGGTTGTCTCTTGATACCAGGTAGATTGGGGTGGTTAAATGATAAACCATTTGCAGGCAAGGGGATTGAAGCTTATATTGTATTCTTTTGGAGATTTTTTTCTTTATTTATTGATTTTTTTCTCAACCTAAATGGCATTGTTATTTGTTCAGGTGCCAGATAGCTGTTCACCAAGAATGCTATGGGGTAACTGATGTTCAAGATTTTACCTCATGGGTTTGTAGAGCATGCGAAATGCCTAATGCTGAGAGGAAATGTTGCCTCTGTCCTGTGNNNNNNNNNNNNNNNNNNNNNNNNNNNNNNNNNNNNNNNNNNNNNNNNNNNNNNNNNNNNNNNNNNNNNNNNNNNNNNNNNNNNNNNNNNNNNNNNNNNNNNNNNNNNNNNNNNNNNNNNNNNNNNNNNNNNNNNNNNNNNNNNNNNAGAGGTATGTCCTCGTAATTGAATGTGGTAATAGTTCTTAACTGTTCTGTTTTTTTTGTATATGGCATGCGTAGATAGTGTATTTACACACATGACACATGTAAGTATTACCTGTAGTTGAAATGGAAAGAAAATGGTCAGTGCCATAATTTATAGTTTCTGCATGGTCTTGTGAAACTTTTTTTTTTTTTGAAATATGCAAAACAAAACAGTAGTTATGTCGCTTCTTTTAGGACTTCTAAAATATTGGTTGATTCCAATTTAATTTAGATGATGCTGCAATCGTAATAGTTATACTTCCATTTATGATTTCTCTTTGCCTATTAGCAAATATAAATGTGATGATGTAGCATTTTGATTATGTATTCTGAGCATTAGTATTTAATGCAGTTATGATTTTCTCTGAAAATGTTGGCTCCAATCTCAATGCTTGTCTGTCTCATCATATTTAGAACTGATTTTTGATTATGCATACTGAGCAATGTAAGTCAGACTACATGTCTACCTATGTCTGACAAAGGGCTTGTCTTCAATGCCGGCTTGTCTTCAATGCCAGCTTGTCTTAACATGTTGAAACTGCTTTTTTAGGGGGTGCACTGAAACCAACAGATGTTCAAACTCTTTGGGTTCATGTTACATGCGCTTGGTTCCGGCCTGAGATTGGTTTCTTGAATCATGAGAAAATGGAACCGGCAACTGGAATTCTTAGAATTCCAACAAATTTGTTTTTAAAGGTATGATGTGGTTATGATGATCATCTAATGAGCTTTCTTTCCTCCTACTTCAATCAAGCATCTTTTTAAGGCAGTGCTTAAAGACTGTCCCTGCCATGCTTGGGTTCTATTTCGCATTCATTTAAAGAAAGTTCTCTTGGCATTGGTTTATTTATTTATTATTATTATTTTCTTTTAGTGTATGGGTGTTGAGTTGACTGCTTGTCTCTGAAATTTTTCTTAAATGTATTGACTGCAGAGTTGCATAATCTGTAAGCAAACTCATGGTTCGTGCACTCAGTGTTGCAAGTGTGCCACGTATTTTCATGCAATGTGTGCCTCAAGAGCAGGATACTGCATGGAAGTAAGTCTTATTGTTCTTTGCTCGTAAACAAATTCATTTTTTGAGAGTTGTAATTTCATGGAATATGTCTACAATGGAAGATAGTGATTAAAAATGATTTTTTTTTCTTATTTTTCACACATGATTTTGCTTACAATGGTCAGTGCATTAAGGAAATGAGGGTTGAACGTTGTAAATTATTTCATTAATTTTTAATATTGAACTTCAGATACACAGCTTGGAGAGGTATGGCAAACAGATAACAAGGAAGTTGATATACTGTGCTGTCCACAGGTTGGAAAAATTTACTGTTCATTGGTTTCTTTATTTTTCATATTTTCTGATTTCAATCTGTTCTTCCATTGAAAGACAAAAGAATTGTCCAACTTTATGCACCATATCTTATGGTTTTTGGTCTTCGTTCCTTCGTATCTTGATTTTTGGTACAGGACCCCAAATCCAGATGCTGTTGTAGCTTTCCACACTCCAACAGGGGTTTTTGCTGGTAGAAGCTTGCTTCAGAACCAGAGAGGCTGTTTCCGTGGTTCAAGGCTGGTTTCAGCTAAGAGAACAGAGGACTCTGAATCACCATCCCCTGATACTAATGACTTCGAACCTCTATCTGCTTCAAGATGTCGTGTTTTCAAACGATCAAAGAATAAGGTGATGAATTTGGGAACCTTATACTTTTTAAAAAAGGAATTGTTGAAATTTTAGCCATGCTTTTTTCGAGGTTGCGTTTCTTTTGGTACAAAATGTCATGTCAGATGTGACTTTTGTTTAGTTTGTAGCTGACTTCTTGTGATCTATCTCTTTTCTTCTTGTTTGCAGAGCATGGAAAGGGAGCCGATATGCCACCGACCAATGGGGCCAAGACATCATTCTTTGGATGCAGTTATTAGCTTGAACACATATAAAGTGATATTCTTGTCTTTGTTTTATTTTTTTCTCATCGTTTCCTTAGATTGTTTTTGTTTGCTGGTAATATTCCATTTTATACTTACATGCTTATTTTGTCTTATGACAGGAAGTTGATAAACCTGAGATTTTCTCTTCATTCAAGGAAAGACTTTACCACTTACAGGTACACCATCATTTTTAATGTGAAATGACTGATGCCTACAGGCCATCATCTCAATTTTTTTTTTGTTTGGGGGGGGGGGGGGGGTGGGGTTGTTGTCCTCAGAGAACTGAAAAGCATCGGGTTTGTTTCGGGAAATCTGGCATACATGGATGGGGCCTCTTCGCTCGTAGACATATTCAAGAAGGAGAAATGGTACTAAATTAAGATTTTACAATTATCCATTTTCAAACTTGTGGGTTTAAGTATTCAACTTGTCTGGAATTTTTTTGATACCTCGAATAAGACAATCAGTGTCTGGTTTCCTCATTAGTGATGTGATAGTTATAGAAGGCATTTATATCTCCAGGTTGTTGAGTATCGTGGTGAGCAGGTAACTCAAAGCATTGCTGATCTAAGGGAGAAACAATATCGTAAAGAAGGCAAAGATTGCTATGTGAGTTCCTTATTCAGTAATCCATGGCCGTGACCATGGGATTGTTGTGGCAGAGCTTGTATTATTTGATTGCAATTATAACTTACTGTTCCTTTCTTGTGTGATGAAGAGTTTGAATTTCTCAATGTGGTGAACTATCTATCCTTTCCTAGGTGTTTAATGGTAATAATTGATTTGTTTGAAACTTGGGCTTTTGGGACTTTTGAAAAGTCTGTCAATAGTATAAATATGATGTGAAAATTGTAGATGAGAGAAATTGATAGGCATCTTCCTAAAAGAAAAGGTTCAATGTAGGGCAATGGAGATAATGGAGTTCACAGCATTCACCTGTCTGGCAAGCAGGTGCCAACATGGTATATTTGTTGAGGACTTTTCCTGCTTTGGTTTCTGTGGGCTTTGTGAAAATGGCTACCTCCTTTTCATCCACCTTGCTTCAACCCCCACTTGATTTAGGGCAGAACAACTTTTTATTATTGCTGCTATATGCTTTGTTTTAATATTTATTGGATTATTTAATCTTTAAATTAATGGTTCCTGCAGCTATTCAAGATCAGTGAGGAAGTGGTAATTGATGCCACAAATAAGGGGAATATAGCTCGCTTAATCAACCATTCAGTAAGTTCTTGTTACAAAAGTTAATAACAAAATCTTCAAGATTCTCTTTGGGGTGTTTGTCTCTCTCTCTCTCTCTCTATATATATATATATGAAGCTCTACCCTAACTTGTAATTTCCTCATTCTTTTGCCTGATCAATTAGTCTGAGTTTGGATTTGGTTGGCTTGGGTTAGGTTAGTGAAATTTGTGTTGACCGTACACAGATTCTGTCAATTAATACTTATATCGGTGCATGTGGTTGTGCAAAGGCATTGTATACCCTGCTTTTCTTGGTTCTGCTCTCCTGGTATCTGAAGTTCTCAGACCCTGGTTACTTGCATTGCACCATTTGAGCCTACGACAAAATAACGCCAAAAAAGGAAAAAAATGCATTATTTTAAGGAAAGTGATTATAAGGAGACAACAATTTTGATTTGGTCTGGATCTTTCAGGTTCTAATGATTAGTGTTGCTTTTAGAACCTGAGCTTCACGCCTGTTTCCACCCATTGGTCTTGTTGTAGTCACCAAAAGTTGCTTCTATTTTGTGCTTAAGTCAGTAATTGTTGTTGCAACTGTTTTATGAACCAAAAATTACTTCTGTTTCATTAATTGTAGTTGCAACTGTTATATGAAATTCGACCTTGATTCTTAGTTCATACATTTATTTGGCCCTATGTGGTAGCATTTTTAAAATGATGTTAAAAATTTATTCCTTCTGTTGCAGTGCATGCCTAACTGCTATGCAAGGATCATGAGTGTGGGAGATTGCGAGAGCCGGATAGTTCTTATTGCTAAGACCAATGTGTCAGCTGGTGATGAATTAACGTATGATCTTCCCCACCACTACCCCCCAACAAACAATATAAATTTTGGTTTCTTGGTTGAGGAATAAATTATTGTGCTTTTATTTATTTATTTTACAGGTATGATTACTTATTTGATCCGGATGAGCATGACGAATTGAAAGTCCCTTGCCTATGTAAAGCTCCTAACTGTCGGATGTTTATGAACTAG

**CsPRMTs:**

>CsPRMT1

GCACAGCACGGGAATGAATGGTGAGTAACAATTACTACTGTACTAGTCATTAAGCAGCTGATCAAACAGAGGTGGAGTGAAAAGTGAAAACCCTATCAAAACCCTTGTACTTTGCTTCTTTCTGTTGTCGGGTTTATAAAATAATTGCCATCACTTCTCGTTTAGTCCAAGTCGATCCATCCAACAGTTTACAACACTCACTGGTAGTCTAATACTACTACTATTGAATATTGAAGAAGAAAGAAAGAAAGATCATCAGAGAAAGATGGCGCTAGGTGAGAGAGGAGGATGGGATAAGAGCGAATCCAGATACTGTGGAGTTGAGACCGACTTCAACGATGACATGCCTCACCTTTTATCCTTCAATCTTTCCTCTGGAGGCTTTGATTTTGTCGTTGCTTCCTTGGTTCGTTCTTTTTTCTACTCTTCTCCACTTTTACTCTTTCTTGCAATTCGTCTTTGCTTTCTTTTTTTTTTTAAATGTTTCATTAGGGCGACTTAGGTAAGCATTCACATAATTTGGTATCGTATGTCTCTGTCTCATTTCAATGGCAATATAGTCACCTACAATAACTTGTTAATTGCCTACTTCTCTTCTCTTCTGTGGTGAAGCAGAAGCACAGCATGCCCTGCTTCATTTGTATGCAGTTACAGCATTATTGATTTTCTTTGTTTTTGCATTTGCTTTTATGTTGCGACTCACAAACCTTTTGCATTCACATTCCCACCTGAGGCAGTTGCATATTTGAATTCTTACTGGCACTATTAATGCTTTAAAACCTGATGACACAGATGGATCCCAATTATCGGCCAAGCTTGATGGAAAAAGACATTGGTGGTGGATCACATGTTCTGCCCTTTGCTGGCTCTGACTTAGTCTTGAGCCCTTCCCAATGGAGTAGTCACGTCGTGGGTAGGAACTGTTCTATACCTCATTTCTCTCTTAATTATGTTCCCTTATGGTCTTTCTAACATTTTTTTTTTCATTAGTTGTCTCAATTTCCTTTGGTCTTGTGCTTTTGTTTGACTTAACTATTGGTGTGTATTCAACTTGGAATAATGATATTATTGTGTGTCTGACATATAATATCACTGAATTATTTTTATCTTTGTGTGCATTCTCATTGCCTTAAAGTGGCTTTGGTAAAGTGTGAACTCTCTCAGTTGATTTTTGAATCTACTCAGGAAAAATTAGTTCGTGGATTGACTTGGATTCAGAAGACGAGATCCTGCGGACGGATTCTGAAACTACTTTGAAGCAAGAAATTTCATGGGCTTCCCATCTTTCACTGCAGGTCTGCACTTTATTATGATTTTGTTGCTTTTTGTTGTCAGTAGGTTTCAGTTGATTTCTTATGCTATACTTGAAAAATGAAAATTGTCCTTGCAAGGAAAGTCTCTGCCTACTTCTTTTAAGAGCTTATGGTTTTGCTGTTGCAGGCTTGCCTTCTTCCTTCTCCTAAGGGAGCATCATGTGCTAATTATGCTAGGTGCGTGAATCAGATTCTTCAGAGCCTAAACAACATGCAGGTGCTTATGGTTTTCCCTTTATTTCTTTTAATGCAATTTTTCTTTCTATTTCTTTTCTCATTATTATCTCTATTTAAAGTTGTGGCTTAGGATTCCATTGGTGAAGAATGATGATGATACTACGGACTTTATTCCTTCGGTGAGTACAGTCACTTATGCTCAAATCTTATCATGGAGGATATTTAGCATACATGTGTACAGCCGCGTGCGGGTGTCTAATATATGATATGTCATATTATATGGATATACATACATTTTTGTGATGGAACAGTTATTGGTTTCCTGATTCTACTCAGAAGTAATCTTTTGACAACATGGATTTGGAAATGAAATATTTGGGTATAGAACTGGTATTTATTGAATTGTTAATGACATAGTCTCTTTTTGTACTTATACCTAACAGAATCTCATGATGAGTCAAGTTATTCATTTCATTTATAAAGGAATATGCCTAGGACTAGCAATTTGTCAAGACGTGTTCTGAAGATGCTTACTTTATCCTTTTGACTTTTGTTCTTACTTGTAGGGTGATTCCTGGGAACTCTGGAATTCATTTCGTCTTCTCTGTGAACATCACAGTCAGCTGTCAGTTGCCCTTGATGTTCTGTAAGTTGAATAGACTATGATCCATTGATTTTTACTTTTCATTTGACCAGATATGTTTTTTATATACTCATGTTTAATGCAATGGACTTTTTTTCACTAGGAGCTCACTGCCTTCAGCAAACTCACTTGGACGATGGCTTGGTGAGCCTGTTAGAGCTGCCATAATTCATACTGATGTAATATATCTCGAGTTTTTTATTATTGTGTTCATAAAATTCTAAATTAATTGCATCCTCCATTGGCGATGCCTGGCTATACAAAGACAATTTTTAGTGTGTCTTTGCTCGGTTATTCAAAGATGTGATTCTTAATTTTGCAGTCTTTTCTAACCAATGCACGGGGTTATCCATGCCTGTCTCGGCGCCACCAGAAGCTCATTACTGGGTTTCTTAACCATTCTATACAGGTATATGAATTGCAGGGATGCTTCGACATCAATTTGTTCAATTAATTTTCTTTTTCTTTTAATTTTTTATTCCTATTACATGTGACCATGTATCTTAATGATTCTCTTGACTGATTATTCTTATAAATTTCTTAAAGTAATTTTATTGGAAAAAAAAATTCTTCATAACATTTCTGGAGAATCTCTGGCAATTGGTTTTTGTTTCCAATGCTATTTAAGCAGAACGCAGTGGTTTAACAATTTTTCAATGGAGTCTCTTGCTAGCGCTGAACATTAGTATGGAAGGCTTATTGTTTGTGATTTCTTTATGATTGTGTTATCAATGTGGTTCTTATTTCATCAAGAATGACCGTACACTGAAGTAGTTCTTTGGTTTCCTGTAATTTTTTAACAGTTGTTTTCTATGATGTTTCTTATTGCCAGCTGCCAGTTTACTAATTATTTTCCTTCGGAATGTATTTTTAGGTAGTTATTTCAGGAAAACTTAACCATGGTGTTCCCAGTGTGAGCTTGGATTCTGCTTTAGACCATACTGGTACTAATGTTGAGAGTAAGTTCCTAAGTTCCTATCTATGCTGAAGTAGTGTCTGGTTGTCAATACTTCTTAAGTGATTTAAAACAGTTGTATTTAGTAATGCTACATTGAAATCACAAATTTTTCATGTGTTATTGCCATCAGGCTCTCAGAGACATTCCCTGAGGCCATATTTGGACTATATTGCTTACCTCTATCAAAGGATGGATCCCCTTCCAGAGCAAGAACGTTTTGAGGTACAATTCTGGATTGGGTTGTGACGTCTTTCATTTTATTGTCTTCTTCTGACCCCTGTATTTTCTAATGGCAGCTCAGTTACAGGGATTTTTTACAGTCTCCATTGCAGGTGAGTGTTCTTTGCTTCTGCTCCCTTTTATTAGTGTTTTTTGACTATTTTTTCTTGTTAAATTTTTGCCTCTACCAGGGACATAATCCACTGTTCCACATTATTTCTGATGAATTTTGCACCATAGAATTTTCTAACTACTACCTTTCATTCCAGCCTCTTATGGACAATCTAGAGGCTCAAACATATGAGACATTTGAGAAAGACTCAGTAAAATATATCCAGGTTGGATTATTTTAGTGACTGTTGGAAGTACTATTCTTCTAAAGACAACTTGTTCTTTTCTTTTTTTTTTCTTTTTTTTTTTCATTCTGATCAGTATGCATGTACTTGTATTTTGCTAGTACCAAAGAGCTATTGGTAACGCCTTGGTGGACAGGGTTCCCGATGAAGAAGCATCTTCTTTAACCACTGTAAGCTACCCACACATATCGGTGACACCCTTTTTTCAATTATCTGTATTTTTAGTTCATAGTCTAAGATATTGCACACACATAGGTGTTTCTCAAAACTAGATGCCATGGGATATGCGAATTTCATGACATTGGTGTACTTTTAAATGTGGCAAGGTACTGATGGTTGTAGGAGCAGGGCGAGGACCTCTTGTTAGGGCATCATTGCAGGTTTCTCCCTGCAGCTTCTTTATAATGGGATGTCAACTAGTTTGTTTGTTTTGAGAATCATGCTACATGTATCAACTTTACTTTGACTGGACCCTTTTTGCCTTTTCTTTCTGTTTGTATATAGGCAGCTGAAGAAACAGGACGCAAACTGAAAATTTATGCTGTGGAGAAAAATCCAAATGCAGTTGTTACTCTTCATGTGTGTTGCAAACTGTGTAATTATGCTTCAGTAGCCAACATAATGTGTCCCAAAAGTGGCAGAGGGCATTTGCATATTGGAATATTGGTGCACAAAAATTGGATTATGTTTAATTATTTGTTGCCTAGTTAATCTTAGATTCCAGAAGCCCTTTCCTTATTGATAAGATACCACGTGGGTCTCTGACTTCCTAATAAAATCAATTTTTTCATTTAAGTGCACAAAATCTTAGAATGATCTTAATTCATAATTATTTATCATAGGTCAATTGACAATAATATTCTACAGTTCCTCATGCCTACTTTGCATTAACATTCTTGATAGCGAAGATCATATGTTTTGCTAGTAATCAAAAGATACTTTTCCTCCCTGTCCTTTTTATCCACAATAAACCTTTGTTTGGATTCCATGTTTTAACTTATTCATTGAGGTTTAATTCACCTTGACTTCCTGCCGTCATTCCTGAGAATGACCAATCTCTCTCTTTCTTTCTATTTGTGTGACATGGATGTACATTTGCCTGTAATATTGAACTGGCAATGAATTCACGGCCAAAAGAATATTGCAAAATTTTGAACTGCTTTTTTGAGGGTGTTTCTTTTTTAGTTCCCCAATTAAAAGTATACCCTACCATTCTTTTTCATTTTTCCTTGTATTTAAGAGGTAACAAAGTGCTTTTTGGGGCAGAGTTTGGTTAGATTGGAGGGTTGGGAGAAAACGGTGACCATAGTTTCTTGTGACATGCGTTGTTGGGATGCTCCTGAGAAAGCTGACATTTTGGTACACGAACTTTGATTTGGTTAATTTGTTTCTTAATTTTCTTCTACTTCCTGCATGTTTGTTTCCTTACGTTTGCCATATCTGCCTTTAGGTCAGTGAGTTGCTTGGTTCTTTTGGTGACAATGAGCTGTCTCCTGAATGCTTGGACGGAGCCCAACGATTTTTAAAGCAAGACGGGATCTCAATACCATCCTCGTAAGTGTATCTTCTTTCACATATTATTGCTATTGTTCTCTGAATAGAGGCTTGTGAAATTATACCAAGGGTATTTATAGTTGCTACTGAGATTCGATTATTTCTAATCAAGAGTGTTCCCACATTTTTATACCTGAATCATTTTCAGGATGATTGTGATTTATATTGCTGTTCGAGCTGAACCCTTTACACTACTGCTTCAGTCTTTGGAATGAATTATGAATTTATGTCATTACTGTATAGATTACTCTTCTAATTATTGTTGAATTAATTTCTTTATCATGGCATGACTTGGTGATATGGTGAGCAATGTATTTGAGCTTAACGATATAACGTTTGATGTTAATGAACACATGATGGTTTAGTGTAAATTTGTTTTGATTAATAAAGTTCTCCAATCATTGACAATTTCAGGTATACCAGTTTCATACAACCCGTGACAGCTTCAAAGTTACACAACGATGTAATTCCTTGTCTTTGTGCTCAAGTATCTCCCCTTGAAGCAATTTCTTTTTCATCTAAATGTGCACTCGCATTGCAGGTTAAGTCTCATAAAGATGTTGTGCACTTTGAAACTGCTTATGTTGTCAAAGTGCACAGTGTAGCAAGGCTGGCTCCTTGCGAACCTGTGAGTTTTTTGTTCTATCCATCTTGCTAACTGTAATCGTAAATCTTCAGCTTATTGAATGTAATTATTGATTATTATAGTCTTTTGAATTTTCTGTTGAGGCATTACAAGATTAGTTGTCATCATTAGACCTCTTTTCTGCTCTTAAAAACCTGAATTCAGGTTTTTTCAGCTGGTTATTTCATGTCTTGTTTGTTAATAGGTCTTTACATTTACTCATCCAAATTTCTCAACTAAGAAGAGCAATCAGCGCTACAAAAAGCTGAGGTTTGAGATACCTAGTGATACTGGGTCAGCCATGGTGCATGGTATGTAGTAATGAAGCCACATTTGCACTAATGTTCTAGAAAGACTATTTTTCAAACCGATCATGCTTCATGGTGTTTAATATGAGATTTTCTAACATAAATTGCTGTTATTGGAAATAAAGGTTGGTTGGTGCAGGCTTCTGTCAAAGAATGAGTTCTTTGCCAGATTTCTATATTTTATAAATAGATAATGTTCTGAAAAATACCACATTTCTAACCGATTTTGATTATGCAAAATTTTGATGATTGATTTTTGGTGCTTTGTAATTCAAACTGATTGTACTACTGAAATAATTTTGGTTGGAGTTGGCTTTTATCAAAGATAAACAAGTCCATGTCATTTCAATTGAATTTGATTGTATAACTGTTAGATAAGTGGAGTGCATTCATGCAATGGAAACAAGGCTACCCGAAAAAAAATTCTTGATGCTAAGCTGAAATTTGATCCATCTATAATGACATAATTTAGTTGCTTGTCTACTTTGAGAAACAATATATTTGCTTGATAAGAAATACTATATAAAGACAATTCATTCCCCAATGCAAATTTTGAGCGTGAAATATTTGTTTACCTGATTAATAGGGTTCGCTGGTTATTTTGATGCGATGCTTTACAAAGATGTGCATCTAGGTATTGAGCCATCAACGGCAACACCAAACATGTTCAGCTGGTATGACACTAAAATCTCCTGGTGTTTGTGTCTGAAGTATGCATTCATTTGTTCATCACATCATTCCTTAAGTATTGCATAGCATCTTATAAACATGTTGTAGTTGTGCTAGCTCCTCTGTTTGCTTTGTTGAGTCTTACTTCGATACTGGGTTGCAAATTCCGGCATGGTAACTGGGATAAAGTTTTTTTTGGTCCCCAGCATGATGCAGTTGTTCTGTGAAACTTCATACTTTTCAAAATGTAACTTCAAACTCGTGATAAAATGAGTTGTAAACTTGTAAATAAAAGGTAATCAGATTTGTAATATAGAACATGAAGCTTGTAAATGGTCTGAGCAATTTAGAGGGACCTCAGATATATGATGGTACTGTTCTTTCCTTCAGGAATATTTGTTTCTTTCCTGTTCAGGTTTGCAATATTTTTTCCACTGAGGACACCGGTATGTATTCGACCTGGTTCTCCACTTGAGGTGCATTTTTGGCGTTGTTGTGGTTCTACCAAGGTAAATTGTTCCAAATACTGTCTAGAATTTATTGGCACCATCACTTGTTTTCCTTTATTTTCTTTATTAGTTTACTTCCATATTATTTTGTATTGAGGAAAACAAATGCACAGAAGACATCCTGAGCACTCTTTTTAATGCATCTCGTGCTCTTCATGGGCACTAGAAGTGTTTGATCTTGCTCTAAACGTTTAGCGTCCCTGACTCTGAATGTTGCAGGTTTGGTACGAATGGTGTGTTGCGTCTCCCAATCCATCACCTGTTCACAACAGCAATGGTCGTTCGTATTGGGTTGGCCTTTAGCATTTCCTCTTTCAAAGGTTAGAACACTAGTTACAGACACAGGTGTATGAAAGCTGTAACCTTATAAAAGTAGATAGCCATTTCAAGAGGAAGTAGAACAGATTTTCAGCTGTTCAGCTGTTAAGGAACAGAAAAAGAGTGTAAAATTTAATCCAGACTTTGATTTCAACTACAGTGACTATTCCAATTTATTAGGCTCATCTTGAAGTATGATATAACGCACCTCCAATTCTCCAATGTCCAAATGCGGGGCTGTTTAGTTCCTGCCAAATAGTTTTCTTCACGTGCGTTTGACTGATGTCTTCGAAACAATGTCATTTCTTGAACGTCTGTGCAGACTGTAGTTATGATTTTCTTGTTGAATCCTTGCATGTAATCAAATTTATGCCTTGTTTAAACTACAGTTATGGTCAAGTTTAAAAGTTATAATATTAAAATATTTGAT

>CsPRMT2

CGGCTCCCATAAGGCAAAATTTATTCGTACCAACTCTGACGCTTTTAGCTCTCTCCCCTTAAAACCCTAATTCTCACTCTCCAATGCTCACAATAAGCAAGCGCAAATGAATACTTGTTAATTAAATCATTTTAATATACACATCTGAAAAATATTTTTGAAAATATTCGCGAGTAAAAAGTTTTCAAAACTGAGGTTTTGAGAGTGATGGAGCAAAAGCACAAACAAGAGTTCGCTTTGGCTTCGGTGACGGAGCTTTCTTCGTCTTCGGCTTCTGCATTGACGCAATCTTCGCCGGTTTTTGCGCGTTTTAGCGCCAGAAACGGACTCGTGGAGCTTCGATTTTCCGAAGAATCTGAGGCCATTGGTGGTTATAACGTCGATCCTTGTACTGCTCAGGTAAGTGTGATTTTTGACGGATTCTTTGTGTGTTTGGTTACTGAGAAAGTAGAGAATCTTTAGCTTTTTCGTAACGTTCTTTTACATTTTCTTGGCAACCAAACACTGGTGGCGGCACTGCATTTCTTTCATTGTTGTCATGTTTATTTTTCCAATTTACATTCGATTTAAGTTGATGACATTTGCTTCTTTGTAATATAATTTTTTGTTTGGACTGAGAAAGCAGAGTGTTAAAGTAGAGAAGTTCATATTTTAGCTCTAGGTTGGTTAATGATTCCACATAGCTGAAATGGAATTGGAATGTTAGCTCTTTTTTTCTTTTTCTTTTTTTCCTTTCTGTCTTTCTGTTTCAATGTACAAATTGTTGAATTCTTTTGCTTCTTAGGTTTATTGTTAACCAAAATGTAGTGTTTATGTGTGGAATTTGTGAAAACCGGTGAATCTAACATTACGGTACTATTATTTTGTGCTCCTTTGTTGCCCAGTTACTCAAGTTAGGACCTGTACAATCCGTTTGCATTTCTAAAGATTCTGATACCAATGAAGAGGTAACCAAACACACTTTTTTTTCCCGCCTCTGTGTTCCTTTTTCTTGAAAACAGTGTGGGCAGCATGTTTCTTTTCCATATTTTATGATCTTACTTTTATTTGAATTCCACCAGGACCCCTGGTACAATGGTTCATATTATGTTTTTGCCCTCTGAAAATCGTAGGTGGCATACATACATACATACATACATATATATATATATATTACGTTTTATGATTAGCATCATGTTTGGTTTTGTTATGCATAGTATGATCCTATGAGTAGTTCCAGATGTTGCATCATTTTTAACAGTATCACTATTGAACCAGAAATCATATTCACGGGGAATCACTATCCAGTTTACAGATGAGGAAGAGAGCAGGGCCTTCCATTGTGCATTTGAGCAATGGAAGACTGAAGTTAAGGACCAAGGTACTCCCTTGGACATCTGCTTATGATTTTGTTATAAAATGTCATTGATATATGATATCAACTTAGATCACTGGACACTCCATTTGGCATGTTTCTTTTTTCCTTTTTTTTTTTCTCTTATTTATTTGTGCTAGCCACTCATAGTTCATTTGTTCACGTATCATTGTTATGATCTCTCTACATACTTTTTGTGTATGTGCTTGGCACTCACTTAACAATTTAGTGCTTTAACATTTCCTTTCCCAGCCTCTTTTAAATTCCTTTGTGAATGAAGTTTAAGTCGGGTTTTAGTGTAACATATGTAAAGGTGAAGTTGCATGAAATCAAGAACTAAATTGGAGAAATACCACTCCACATCTTGTATTTTATCTATACGTAATTATCTGTTTCATAAAGAACATTTTGACCAATACAAAATGTCAAGGAAACTCTCCTTTTGACAAGCCAAGTCTCCTTAATATTCTATATCTATTACTATTGTGGTCAAGGAATTGGACCCTAGGTAGTTAGTAGGAGAAATTTCTTTCCAGCTAGGTAGGGGTCAATGGATTGTTTATCCAATCTAATCTTCTCTTTAAGACGATTATAGTCTGATTAAAGCTTGAAATCAAATGCAGATATGCACCCCTGAGTTTTGCCTCAGAAAAAGGGGGAGGAACAGGGCTGGGTACTATATTACCTTTAGAAAAGAACTGCCGATGTGTGATTGTAAAATTTCTTTTTCTTTACTTCTAGATGCAGTGGTACGCTTTGCTGTATACCTGTTCTCAAATCCTCTTCTTTTGGTTTGCTTTCCAGGAATGCATTTACCAAATGGAGATGTGTCAGGTTCTTTAAAGAGCAAGTTTGATGATAAAGTAGAGCCATCTTCTGCTAAGATGTACTTCCATTATTATGGGCAACTGCTACATCAGCAAAATATGTTACAGGATTACGTCAGGACAGGTTATGTCATGGACCTGTACAAAATTTTCCCTTTGTCATGCTTTTCACAATTAAATTTGGATTTGTTTTAGACCTGATTAAACTTCACATACCATTTTACCACTGAATGTATTATATTAATACAAGTAAATTGTATGCTATAGAGCAACCAAACAGAGTTACTTAGATTTGCTGATGATATATAAGCTATTTTTGTTTCAGGAACCTATTATGCTGCAGTTATAGAGAATCGTGCAGATTTTACCGGTCGTGTAGTTGTTGACGTTGGTGCTGGTAGTGGTATATTGTCATTATTTGCCGCTCAGGTTTGCTTTGTCTTTTTAGTAGTGAAATTTCTTATGATTATAAACCTATAATTACATTTATTTTGCATCTTGCTTGGGGATGTCCTTCTTTTATTTTATTTTTTGGGGCAAAGTTGCAGTTTGATATTGTATTCCTTCACTTTGCCCAATTAGGCTGGTGCAAAACATGTTTATGCTGTGGAAGCATCTGAAATGGCTGAATATGCTCGCAAACTTATTGCGGGAAATCCATCGTTGGGAGAACGAATAACTGTGAGTTTTTTTTCCGTAATGTCAAATTTTCTGTCTTTGACCTTGTCAGTCTGTTATCTTGTTTTATTCCTGCACTATGTCATGAAATTGTAGGTGATCAAGGGTAAAGTTGAAGAAGTTGAACTGCCAGAGAAAGCAGATATTCTAATCTCTGAGCCAATGGGTACGTATATATATCATGATTGCTTTTGTTTCACTAATGGACAATGAAATGTGTATGTTGTTTAACTTTCATAGGGTTTGAGCCTCAGCTATGGTCAAAGTTCAATTTGGTTAGGGTGAATTTTGGTCGGCCAGAAAGTAGAGTGCTTTAGAGTTGTAATGGAGTCAGCATAGCATTGCAAATGTGTTTTTAGGCAAGTCTGCATTCGACTTTTCTTTAATTGCTTTTGGATGTGGCTTGTAGGCACCTTATTAGTGAATGAAAGAATGTTGGAGACCTATGTGATTGCTAGAGATCGATTTCTTGTCCCAATGGGAAAAATGTTTCCCTCAGTTGGAAGGTAAATATCACCCATGATATCCCTTATTGTCCCGCATTATTTTTTGAATCTTGAATCTCCCTGCTCTTAACCTCCAATCATTTTGATTCAAAGATAATTGCTGTTGTAGGATTCATATGGCTCCTTTTAGCGATGAATATTTGTTTGTTGAAATTGCGAATAAGGTGTGCAGTCGGCTCAAATTATATTTTGTGTTGTGCCTCATGTTTTGACTACACAGTAATTAACTTTTTGTTATTTATTTTCTCTTTTAAACCTTTTTGCAGGCTCTCTTCTGGCAGCAACAAAATTATTATGGGGTTGATCTGACACCCTTATATGGATCTGCATTCCATGGATACTTTTCTCAGGTCTATTATATTTTCACGACTCTATGTTGTGGTCAACCTCCATGATAGTTTTTCCTTTGTGGAAAGTCTAATAAGCTTTAGAAGCTAAGTCAACCTTGAGATAATCCTCCTCACATCCTTTTTATCTTTCCATCTGGTCAAATTGGCAGTTTTTTCCGTTCCTTATTGAAGTCTTGTAAACAATGGATGCTCTCGAATGAGGATAGAATGTTACATCCATCCAGTTATTTGATGCTTTTTGTTTGAGTCCAAGCAGCACCAAAAACTTGAAAGTTTTTCTCATCTTGCATCTGTACCCAAATCGAAAAATAGGTAAAATATACACCCAAATAATAAAAGCATTGAGACTTATCTGCTGCTAGTGCTTTCCTAATTCTCAGAAGCCTTGACAAGCTGTCATTAAAGGGTTAAAGTGTGTCTCGGTGTTTTCTTTGCATTACATTGCCTGTTGGGTTGCTTGATATTTCATTAAGTTGTTATGGTTACTTCTGTGACCATATCTGCTTAACCCATCTTAACTGATAGTCTCCATCTCAATATTTAATGTAGCCTGTGGTAGATGCATTTGATCCGAGATTATTGGTGGCCCCTGCGGTTTCTCACGTGATAGATTTTAAGAAAACAAAGGTATTAAATGTTTGGTAGCTTTCAAATGTTGTGATTCTTTTCTTATGCTGTCATTTTCTGAACATGTGCCAAGCACCTCTCGTTTATTACTGTTATCTTAAAATTCTTTCTTGGTTTCATTGTTTTTCTTTAAATCGAACTATACTGCTTTTGATACTTTATCGCAAATCATTATTTAACCTTTTCTTACTCCCATGTGCAGGAAGAGAATTTATATGAAATTGATATTCCATTGAAATTCATATCTTCTGTCGGCACCAGAGTACATGGTTTAGCTTGCTGGTTTGATGTACTGTTTGATGGGAGGTAATAAATCAGCAATCAATCAACCAGTCAAATATACATCAGCCATGGAGTTAATTTCTTTGTACATTGTATTGTATCTTGAGATCATGGGGTTCTATCTAATTGAACTGCTTTAATCAAACAGTACCGTACAAAGGTGGCTAACTACTGCTCCTGGTGCCCCTACTACCCACTGGTACCAATTACGCTGTGTTCTCTCTCAGCCGCTGTATGTTATGGCAGGACAAGAAATAACAGGTCAACTCCGCATGATTGCACACAGTGCTCAGAGTTACACCATATATCTAACATTGTCAGGTTGGTTCTTGTCTTCTATTTTGTTGGTGTTGGTGGCTTGTCGTCTTGGTTTTCTAACAAGCGCCAATTATAACTAAATTTAGTTAAAATGTGGGGACCTGGTGCTGACCAAGGAGGAATACTTCAGACATCATCTTGCAAGCTTGATCTTAAAGAACCCTATTACAGAATGTCCCAACCGCAACCCTATGTACTGACACAAGATCAGCAACCACATCAGCTAATGCATTCACAGGTATTGACTAATTCTTTCATTAGATTTTTGCCTGTGCAATACTGAATTGTCTAACCTTGTACCAGAATGTCACCCAGGATTATTTGTAGTTAAGCATCATGTGATCATATTGTAAGATTACTCCATTATCATGGCAATTGCAGTAAATTTTTTTAACCAATGTAACAGGATATACCAATTCAAGCAGAGGATTTAGAGGAACCGGAGTTAATACAACTACAATCTCAATGTTCAGGTGCTCAGCTTCAATAAGTAATCATCATGTATGTTTCGAAATCTAGGGTTGGATAAAAATCAAATATACAAGTCCGTGTTTGTAACTTCAAGTTTTCTACAGCAGTCCTCTTGGTCACTTTTGATCATTTGTCATGATTCCTGTATATTAGAGCGCCATGATTTTTAGCCTTATTGTTGCTGGAATACGCATTTTGTATGATTACCTTGTGACTTGGCCAGTTCAAATAATCAGATTCAGTTTGGTGGCTTTAGTTAACCTCCTTTTTTTTTCCGTTCCTTATTGAAGTCTTGTAAACAATGGATGCTCTCGAATGAGGATAGAATGTTACATCCATCCAGTTATTTGATGCTTTTTGTTTGAGTCCAAGCAGCACCAAAAACTTGAAAGTTTTTCTCATCTTGCATCTGTACCCAAATCGAAAAATAGGTAAAATATACACCCAAATAATAAAAGCATTGAGACTTATCTGCTGCTAGTGCTTTCCTAATTCTCAGAAGCCTTGACAAGCTGTCATTAAAGGGTTAAAGTGTGTCTCGGTGTTTTCTTTGCATTACATTGCCTGTTGGGTTGCTTGATATTTCATTAAGTTGTTATGGTTACTTCTGTGACCATATCTGCTTAACCCATCTTAACTGATAGTCTCCATCTCAATATTTAATGTAGCCTGTGGTAGATGCATTTGATCCGAGATTATTGGTGGCCCCTGCGGTTTCTCACGTGATAGATTTTAAGAAAACAAAGGTATTAAATGTTTGGTAGCTTTCAAATGTTGTGATTCTTTTCTTATGCTGTCATTTTCTGAACATGTGCCAAGCACCTCTCGTTTATTACTGTTATCTTAAAATTCTTTCTTGGTTTCATTGTTTTTCTTTAAATCGAACTATACTGCTTTTGATACTTTATCGCAAATCATTATTTAACCTTTTCTTACTCCCATGTGCAGGAAGAGAATTTATATGAAATTGATATTCCATTGAAATTCATATCTTCTGTCGGCACCAGAGTACATGGTTTAGCTTGCTGGTTTGATGTACTGTTTGATGGGAGGTAATAAATCAGCAATCAATCAACCAGTCAAATATACATCAGCCATGGAGTTAATTTCTTTGTACATTGTATTGTATCTTGAGATCATGGGGTTCTATCTAATTGAACTGCTTTAATCAAACAGTACCGTACAAAGGTGGCTAACTACTGCTCCTGGTGCCCCTACTACCCACTGGTACCAATTACGCTGTGTTCTCTCTCAGCCGCTGTATGTTATGGCAGGACAAGAAATAACAGGTCAACTCCGCATGATTGCACACAGTGCTCAGAGTTACACCATATATCTAACATTGTCAGGTTGGTTCTTGTCTTCTATTTTGTTGGTGTTGGTGGCTTGTCGTCTTGGTTTTCTAACAAGCGCCAATTATAACTAAATTTAGTTAAAATGTGGGGACCTGGTGCTGACCAAGGAGGAATACTTCAGACATCATCTTGCAAGCTTGATCTTAAAGAACCCTATTACAGAATGTCCCAACCGCAACCCTATGTACTGACACAAGATCAGCAACCACATCAGCTAATGCATTCACAGGTATTGACTAATTCTTTCATTAGATTTTTGCCTGTGCAATACTGAATTGTCTAACCTTGTACCAGAATGTCACCCAGGATTATTTGTAGTTAAGCATCATGTGATCATATTGTAAGATTACTCCATTATCATGGCAATTGCAGTAAATTTTTTTAACCAATGTAACAGGATATACCAATTCAAGCAGAGGATTTAGAGGAACCGGAGTTAATACAACTACAATCTCAATGTTCAGGTGCTCAGCTTCAATAAGTAATCATCATGTATGTTTCGAAATCTAGGGTTGGATAAAAATCAAATATACAAGTCCGTGTTTGTAACTTCAAGTTTTCTACAGCAGTCCTCTTGGTCACTTTTGATCATTTGTCATGATTCCTGTATATTAGAGCGCCATGATTTTTAGCCTTATTGTTGCTGGAATACGCATTTTGTATGATTACCTTGTGACTTGGCCAGTTCAAATAATCAGATTCAGTTTGGTGGCTTT

>CsPRMT3

AAAGATTCCAGAGGTTTAAGGGCTGAAAGAAATAAAATGCGTCTCATTTAGATTATTGTATTAATTAATTTTTTTCTGTGGTTAATAAATTAATTTTGATTATTGTATTCATTAATTTTATCTGTCAACGTTGGTCAAAATTTTATGTCAAAATATTTGCAAGAAAAATATCTTCCTCGGCTCAGCCCTCGCGCGGTTCAACGATCAGATCAACCGAAGCGAGAATAATTAAATAGAAGCGCGCGCACCAAGTGTCGACGTGGCATGCAGTGATGGCAGAGCGTTCTCCCACCAGCCAAAACAAACACAGCAGCTAAACCCTACATTTCAATAGCGACGTCGCCCTAAAGCCCTCTCTCGCACTCTCTCTCGAGCCTTCTTGAATCTCTCACTCAATACGGTGCGTAGCGAAACACCGTGTGCGAGTAGACGCAGCCATGGGGAGCCACACAAACGGCGTCGTATCAGGTGATCTTGCGGGGAGCAGCAATGGAGGAGGAGGAGGAGGAGGAGGAAGAGGAGGCGGAACAGCAACGGTGGACAAAGAAGTGGACTTCGCGAACTACTTCTGCACCTACGCGTTTCTCTATCACCAAAAAGAAATGCTCTCCGATCGAGTCCGCATGGACGCTTACTTCAACTCCATTTTCCAAAACAAACACCACTTCCAAGGGAAAGTAAGTTTCCTTTTACTTTCGTGTCTCTACTTAGTTTGCATTTTTGCAATTGTACAATACGCGCTCTTACTTCAGCTTAATTAAATCTTATTAGTTAATAGTTGTAGGCTTTGGTGTAGAAATTTGTGATTAATTCTGTGTTGTTGTGGTGAAGACTGTGTTGGATGTAGGAACTGGTAGTGGCATTCTTGCAATTTGGTCCGCACAAGCTGGTGCGCGGAAGGTATATGCTGTGGAAGCTACCAAGATGTCGGACCATGCTCGGACACTTGTCAAAGCGAATAATCTTCAGGATGTGGTTGAAGTGATTGAGGGCTCTGTAGAGGATATTGTCCTGCCGGAGAAAGGTTCATGTCATTTGCTTTTGCTGTAGCTAGATGATTTTGAAATGGATTGAGTTCATTGGGCCTTGTGGTTTTCGATTCTGATAATAGATTTATGGTCAATGTGCAGTTGATGTGATTATCTCTGAGTGGATGGGATACTTCCTTCTGCGTGAATCTATGTTTGATTCAGTGATATGTGCTCGTGACCGCTGGTTGAAGCCAACTGGAGTTATGTAAGTAATTTGAGATGCCAAAGGGTGAAGAAACACTTTATATATGCGGCGCAACTGATCATCTTGTGGATTGTCTCTTATTTGACATTTCAACGTAAAAACACCTAGTACAAAGTGATTGGACTTTTGTTCTCTAACTGTTATCCTTGCCTTGATTCTGACTAGTGAAAGACAGCTTATATGTTAAATTCTGAGAGTAGGTTATATAGGGATATAAATTTGTACCTGTTGGCTGATGAATGATGTTTGATTTTCCTACGGTAAATGTTGATTGTCAACGGCTAGAATTGACTACCGACACTAGTAGGGGTGAATGCTATGTTAAGATTCTGGAGCGATTTCCCATCATAGAAAAATAATTTCTCATGATGAGGGGGAACAAGTGCAATTATGATTCAGATTGAGTGCAATTAACTGTAGGAAGGAGTATAGTGGGAATTTATCATTTCTCGGCAAGTTAGTGGAATTACCACAACTAGGGAGCGAATAACGACAATGCCAATCACATGGTAGAAGGGCAGATTAGTGAAGGTGTTCAAATTGACAATTGGATGTTCTACTGAGCTTATCATTCTCTTGTCCTTAGTTTCCTTTATATTGCAGACTTAATGTTTCTTCTGTCTTTTGGTTTGAAAAGTTTATTGCTTTTATAATCAGCGACTCATTTGTGAATGGTTGACCAAACAAGCATTTTTCTCTCAGGTATCCTAGTCATGCTCGCATGTGGGTGGCACCAATTAGGTCTGGTTTGGGGGATCAAAAACAGCAGGATTATGAAGGAGCACTGGATGATTGGTATAGTTTTCTGAAAGAGACTAAAACTTACTATGGTGTTGATATGAGTGTTTTAACAAAGCCCTTCTCTGAAGAGCAGAAGAAATATTATCTGCAGGTAAGTTTTAGAACTTTGTTGCTTTTGAATTTTAATATTGTGAATTTATTAAAATCAACGGCACTAAATCTTTACCTTTGATGCTGCTTAAATTTAGGTTAAATTTTATGTATGATGTTGTATGTTGTCCAAACACCTTTCCATTAATGTAATTCAGATTTCAGAAGCTATAGCAATGGGTCTAACATCCCTATATTACACTCTTTCCTCCCCCATGCCCCCTTTCCGATCACTCTAAAGTGTTGAAGTGCTAACAAACTGTGCCTTTATGTGAGATTGGCTGAGTGAATGATGATGTGACTATACCTGCTGATGTGTTACTCAATTCATGTCATTTTATCAAAGGAGAATTTATCTTTGAAATCTCTGGCTGATTGGAAGGGAGTTAGGTCAGTGTTTTATTGACTATGTATCTAATTGATGCTTTTTTATTGAATACCTCATTCAATAAGAACTTGGATTTCCCAAGTCTTGAGTATTGTATCATTGAACTTGTGAACCATATTGGACTTAGCTTCTTGTATAGAAGGTACTTTCTAAAGCACCTGACAAAAATCCACTTTGTTACTCTTATATCACATTCCCTTGAAGAATGAAAGAGCTTGTTTATCAGATTGACCTTTTATGCACAAGACATTCTGCTTTCTCCTTTTTTCTGTCCTGCATTTAATGAGAGAATTCTATTAATTTTGCAGACATCATTGTGGAGCAACCTTCATCCTGATCAGGTTATAGGGACAGCAGCTGTTGTGAAGAATATCGATTGCTCTACTGCTACTGTGGATGACATTCGTGAAGTCAGATCAAAATTTTTGTCATCAATCAGAGGAGAGGGCACAAGGCTTTGTGGGTTCAGTGGATGGTTTGATGTCCATTTTAGAGTAAGTTGAGCTCCTGTGCACCCTTATTATGGTGCATGCTAATGTTCTCAGTCATCCACATACTGAGGAAAGTATGATTCTGATGCCTTTTTGTGCACCAGGGAAGCACGGAGGATCCAGCTCAGCAGGAGATTGAGCTGACAACTGCCCCTAGTACTTATAATGGCACACATTGGGGCCAGCAGGTGTATATCTCCCAAAAATATATCCTCTTTGGATTATCTTCAAATTTAAAATGCTGAATTGATTCATGGATGGCTTAATTTACCTGTCTTTATTTCTCTTTGTCTCAATATCATCAGAGTTATGCATGTCATAAGTATTAGCCACATTGACCTGTATATCATGCAGTAGCTAAGTGTGTGTTCTGCTGGTGTGCAAAATGGTCAAGCTTGCACAGCATTTAGCGACTTCCATAAACTTTTCATCATATGATTTGTTGCAATACTATTGCCCTTGTCAGAATTTAGGCCTCAGCTCTTTTTCTTGCATGCATAGTGTGTTCCTATATAACATTTTGCAGTGCTGGTATGTTTTGCTTATTTCTTATGTTCGTGGCTTCTGCTGGTGCACCATCCACTCCTTTCTAATTTTTCTGTTTTCTCCATTTAACAGGTTTTTCTCTTCCGTCCTTCCGTTCGTGTTAGTGAAGGGGATGATCTGAATGTTTCTTTCTCAATGACTCGTTCTAAGGAAAATCATCGGTTGTTGGAAGTTGAGTTTAGCTGTGAGATTAGAGAGTCTACTGGGCAGATACTTCCGCCAATCAAAAACAAGTTCTATATAGAGTGAAGAAGGAAACAGGTCACATAATTGGTACTCTTCCATTCGCTGAGGTACATATTATAGCTCCTTTATTTTATAACAATCACAAAGCATGATTTGTACAGAGAAACTATGTTAAAAGAAAATGAGTATTCTATATTAAGCCTTAGCGCATAAGCCTGGGCTAAGGCCATAGATTAAGATAATGGCACTCTTTGCCCAATCTCAGTTTTGTGGGCCTAATCCTGTGCTACTTGTTCTTTTCGTGTCCTCTTGCCTATGTAGCCTAGAATCTTCCCATGCGTCCAAGAACTTTTGCACTCACAGGCTTATTATGACTAGAGTTGGCAACATAATATAGTCTGTTACTTCTTATCAAATATTTGAAATATTTATTAGAAATCTGTTTAACTTGTAATTTCTAAACATTTTAAAGCACTTGATCTGAATTTTGGAAGCTATTAATTACAACTTTTTATGTTATTGTTTGCCTAGATCTAGCTAGTTTCTAAAGGCCTCATTTTTAAATTTGCAAATACAAAATTTTGATTACATTACGAAATTGGATTGGCTCATATGAAAAAAAATTATTTTTCTTATTATTGTCTGTAGATCTCAGTGTTTTGAGGTTGGATCCTCCTGTTTCAGAAACTGATTGTATGAACAGAAAAAAGTTTTGCCATAAATAATCCCATATAGACTGGAAAAGACGAAGCAACTAGAAGAAATAATAATTTGGAAGTTAGAACTCCATGGTTATGCACGGATATATACATTTTTTTTGTTTACGTGCATCTTCATTCCTGTGGTTTAAAATCTCTTACCCCAGTAGTTCACCATCAGTTGCAAACCAATTTTTGCTAGAGTCAGATTGTAAGTATGCTTCCCTGCTGCACTGGGGTCAACATGTGATTAGTGTGCTAATATCCTTTATATGGCAATTCCTTCTTGGATTTTTTTGTTTCGTTATGCATTTGATATTTCATTCTTTAGTATAAACTCTCCTGACTATGTTTCATTTCCCTTTCCCAGCAGTGCCTGTATGCAAGAGATGTGACCTTTATGTTTTGGCACTTCTGATCGTGTTCTTGGATATGGCTCTTTCCAAAATCCCATAAAATGGGTGTTTTGGTTCCTTACTTTGCAGCATGAATGTTCTGGCCTTCCTGTTGTGTTTGAAATGATAGTTTTTGTTAACTTTACTGGAATTGTCTCTATAGTCTTTCTTTCCTGCTTTCCTTCTCTATAATTTTCCTTAGTTTCCCCACCACCACCACCATCCCTTCTCAAGTTTTTCGTTTTTGTTCTCTTGGGGGGTAGGTGGGATCTGATTTTCATACAAGGTCAAAAATATACAAAACGCTGCTGCTTTTGACAAAGGTTTCAGGCAAATGTTTATAAATGACTTACTGCTGTTGTTGCAGTGGCTTTCAGACAAACTGTTGGACAGAGGACTGGGAATTGTATATCATTGATAGTTCTAGCATCCATGGTTTCAATCCTTTGTGGGGGTAGTATTTGTTATTTACAAAAACTCCCGCCAGTACTCAATTACGGGATTTATTCCCACGAAATCTTCAGATTCCGACTTAAATTAACTTGCTTGCACATTTCAGATTGAAATTCACGTCATTTTTTTTTAGAAGTGTTAAAAGTTTGTGGAAGCCCCTGACACCGAGTCTAAAGAGAGTTTGACGGACCAGACTTTCTATGCACTGTGTAC

>CsPRMT4

AAAGCAAAGCAAGCAGCCAAACTGAAACCAAAAAGCTATACCCGCACGCCCATAAACCCTAAACGAAAATTCTTCGAAAACCCTAGTCGATGGGCCGTCGCAAGAACAACAAGAGCAGCGACAACAACGAAAGCTCAACGAAAGTCTCCGACTCTAATCCCGATCGGCAGCAACACAACACTATTACTCGCTTCGCAGACGCGGAAGAAGACGACGCCACTGCGAGCTCCATTCTCGATGATTCGGTTGCGGCGCCCGTCGATGGCACTGCTATCGAAGATGAGGCCATGTGCGACGCTGACGTGTCGATGATTGACGGCGAAGACGATAAGACCAGCGCCGATTATTACTTCGATTCTTACTCTCACTTTGGTATCTATCCATTCTGTATTCTTTCATTAGCTGAAACTAAATGGGGTTTTTTGAACTTACTAATCGATCGATACTTGGATGAATGCAAATTTTATTGCTCCTGTTTCTTGCAAAACTCATTTTGGCTTTCTGTCCTTTTGTTTTTGTTTGCAGGTATTCATGAAGTATGTACGCTGAACCTGGACACAAAACTCTTTGGCTTTTGCTATTGAAACATTATATCTCTAACACATTTGTTCAGATCCAATTTGGATGTTGTATTATTTTCTTATCCGACGTAATGGAATGTTGGTGTATGTTGTGCTGTCTGTAGTGTGTAGAAGTTTCTTCTGCCAATAACTTTCTTTTAAATACATTTTTTTGTTAATGTTTTAAAGTTTTAATCCTTAAATGATATGTTTATTGTTAGACTGGCTATTCAATATTTTTGATTATCTGCTTATTGTGCTTAAATGATTGTGTTGCACTTAGAATATAAAAGCAGTTGCAAGATAATAAGCTTCTTGTTAGAATTCAATACATTAATTTGCTTTAGGGTCTCTGTGGCATGAGTTTCGAAAATATCTTGCTTTCTTTAGCTATCTGAAAACCTTGCCTGTGTCAGTGAATTTTGCTCAATCACTTGTTCTTTAGGGGAATTGTTCTCCCATTTTTCCTTCGGACTTTGTGCTTAGAATTTCTACACATGGTAGACTGGCAACTTTGTTATTTGCTGTCTCTAGTTTATTCTTCTTTATTTCCTCATGGAAGGCCAATTGAGTTCATCCCAATTTGAAAAATAATTAGTAGATCATTGCTGACAGTTTATTAAATTGCAGGAAATGCTAAAGGATGTAGTGAGAACTAAGTCATATCAAAATGTTATTTATCAGAACAAGTTTCTTTTCAAGGACAAAGTAGTTCTTGATGTTGGAGCCGGGACTGGAATTTTATCCTTATTTTGTGCAAAAGCAGGGGCAGCTCATGTTTATGCAGTATGTATTGCCCTTCTTTTATTTTATTTTATTTTTTAATTAATATCTTCTCAACCAATATAGTGCATACGATACCTGGCTTTTGTTTGTAGTTTCTCATGCGTTTATCTATGCTCGCTGGATGTGGCTGACAAATTTCATATCTCCAGGTTGAGTGCTCCCAAATGGCTAACATGGCTAAACAGATTGTCGAAGCAAATGGATTTTCTAATGGTGATTTCACTCAAGTCCATATCCCATTTGTGACTTCTTGTATAATTGCTTTGAAAAGTTTAGCCTTTTGCTCACTCATTTTTCATTTTATTATTACAGTTATAACAGTTTTAAAAGGGAAGATTGAAGAAATTGAGCTCCCAGTTACAAAAGTGGATATAATTATTTCAGAGTGGATGGGTTATTTTTTGTTGTTCGAGAACATGCTAAACACAGTCTTGTATGCTCGTGATAAATGGCTAGTAAGATGCACCTAAATTTTTATATCTCACTGTGCTTTCAGTTAAATTTATAATATATTGAATTTTGGACGGTTACTATCATATATCTAATCATTATTCCATTGCAGATATATGAACACATTTCTTCTTATTTTTCCATATTTTTTTTTTCAAAAAAAAAAATCCTTGATGATTAATCAGTGTCATGGTCAAAGAATTGCTATCTTTTTTTCTTGTGCTAAGTTTCTAGACTATATACTTTAAACTACCATATCAGTTAAATATTTTCACACAGTATATATATATATATATATAATTGTGAATTGTTTGCTATGCTGAGCTTGTACTGGTAGTTTTCCATTCACAAGCCAAATTCTGTGTTTATCCATGTCATATGTTGACGTGTTACTGACAAAACATCATGTGTGAGCATGCGTTTTTAAGTCTGTTGGGTCTTCTTGTTACTGAGGGATGCAGGCATTCTTTGAAAGTCAACCTACTCTGAACTTCATTTTACATATATTTAGCTTGTTAAATTATCTGGGCATTAGCTATGTGGTATACATTCTCTTGATGACTTAATCTGAGTACTAGACGTGTGAAGTCTGCATTGAGGATATACATTGATCCAAAATTAATTCAGATTGATAGAGATTAATTGTCTTGTTTAAATGAAATGGCAATGTATGGTCAATTTGATGGCTTTATGAAAAGACTTAGTTAACTTGCAACTTGGTTGAGGCCCTCATAGGACCTTCGGGCTGAGCTGGGTCATGTGTTTGTTTGAGTTGCAGTCTTATTTTTCCATTCAATATGGAATTTCCATGAAGTTGAATTTTTAGTTATATTGCTGCTTGCACCCTGTTTGGCTAGCTACCCTTAATGTAATCTTTTCCTCGCCTTCTGCCAAGTGCTTTTTGGTAGTGCCCCAAATCGAGTTTACTTTTTGTTCTTACTAGACCATGTTTTGGCAATTTAAATCAATTAACATATGCCTTGACTGATTGTAGGTTGATGATGGAATTGTGTTACCAGATAAAGCTTCTCTCTATTTGACAGCCATTGAAGATGCTGAGTACAAAGATGACAAGATTGAATGTGAGTCTATTGCCCTCTTGCATGGATTCAATTAAGTAATCTAGTTCATATAGGGAATATGTTAAAGAAATTAGTTTGCATCGTGTGGTTGGGGTTTCAGCTGCTCTTGAAGTGTTTTTATTACTGTGCATTAATTTTTAATGCGTTGCATTTTATTGGCTCACGCATACTACTCTTTCATTTTACCATTCTCACTTGTTCAGTTTGGAATAACGTCTATGGCTTTGACATGAGCTGCATCAAGAAGCAAGCTATGATGGAACCTCTTGTTGACACAGTTGATCAGAATCAAATTGTTACCAACTGCCAGCTACTCAAGGTTGTGGATGAAAACTTACTAAATGCCTTTTCTTTCAACAGTGTGTGTGTGTTCCCTCTGCTTTCTGAGCCATTCCCCTGAAGATATTAATAGACTTGTTGGCTGATGCACTTGAATTGTTTTTACAACAGACAATGGATATCTCCAAGATGGGACCTGGGGATGCTTCCTTTACAGCTCCATTTAAGCTTGTGGCACAACGTAATGATTACATCCATGCTCTTGTAGCATATTTTGACGTAACCTTTACCAAGTGCCACAAATTAATGGGCTTCTCAACAGGTTCATTTCTCTGTCCTTCAACTTCTTCACTTCATTTATCTTTATCTTTATCTGGACAAATGAGCATTGAATCTGAAAAGTGACCTCCAAGCATTTATGAAGTTTTTTCTCGTTAGTTAAATGGGCTTACTCTAGATATCTGCTGCTTCAAATCTTAGTTCTCTTTAAAATCAATAAGCCTGTGAATCACGGTTCAGAATGGTTTTTTCCAAAAATCTGTACGGTTTTTGTAGTTAAATTGCTGGCAAAATTTTGATGTGTTTTTGACAACTTGAAGTATATAATACAGGTCCAAAATCACGGGCTACACATTGGAAGCAAACAGTTCTTTATCTGGAAGATGTATTAACTATTTGTGAGGGGGAGGCAATAAGTGGGAGCTTGACTGTGGCGCCAAACAAAAAGAATCCACGAGATGTTGATATAATGCTGAAATATTCATTGCAGGGTAGGCATTCTGCTATTTCCAGGATCCAATATTACAAGATGCGTTGAGTTTGGCCATTGTCAGCGATAACTTGTGTTCCCCCACCCCGCCAAAAAAAAACAACCCCCCTCTCTCCCTCCCTTCTTTTAGTTCTCATTGCTCCAGTATCTGGAAAAATGAGTTGGTTTTATTAAGTGTAACTTTGTCTGTAGTTCGTATTTATTCTTTTTAACGTATGTTCCAGCTGATGGAAATGTATTCTTAAAATTTTGATATTTGTACCGCTGTACCAATTTTTCCCCTCCCAGTGAATCTTTCCTAAGGGTCGCTGACCTAACAGTTTTGGCACATTTAAACCCTTGTTCCCTTTCAGAAAAACAAAGCCCTCGTTCGCATAAAAGATTTTCCTTCAAATATCGGTATTCTTTGGGTCCACAGCTGACTGAGTATTCGGCTTTATTGGATCATGCCTGCATCCAGAAGTTAGGCTCCATTATTGTTGCATGTTGAAGCATGTTTCCAGTGCCATTGATA

>CsPRMT5

CTCTAATTGAAAACTCTCAAAACAAATCCCTCAAACGACATCGTTTGAACTATCTTTCTCTGACCATCCTCCGTCTTCGTTATTCAGTCCTTTAAGTTCCCCGTCTAGGTTCCGCCACTACCACTACTACCAGCACCCGAAAAAACAGTACAAATTAGCAAAAGTAATACAATGTACTCGAGCATAGGATACAGCAACGGACACCATCACCACCACCAGCCGCATCAGCAAGAGCGCGAGCGCATGGGTGGAGGATTAACGAGTCACAAAGACCGAGCTAGAAGAGGAGGTCGCAGGTCACGTGACTCGCTTAGGGCTTCCGAGCATCAGCAGCAGCAACAACAGCAACAGCAGAACGATCGCAAACCAGCAACACCTTGTACGGACTTCGACGTCGCCTACTTCCACTCCTACGCTCACGTTGGTATCCACGAAGAAATGATCAAGGTCCCTTCTTTCTTATTCTCTTGTCGTTAGTTGTTGTGTCAATTTTTCATTTTCATTCATGAAATAATTCGTGAAATTTGAAGTGTACTTTTTAATACTAAAGTGAGCATTTGCATATTATGTCGATAACTAAGTGAAATTATGAGACAATGGTTTCTTTGAAGGCACATTATGCCCCTTTTGAACTGGTGACGCAAGGATTTTTAATTCCCTTACTCAGTTAAATTAGACATTGTTTCTTTCATTCATATAATCAAGTTATCCAACAGAAAAGGGCTTATATAATGAATTGTTTATACTCTCAGCCGAACCAAGGCTCTTTTAGACAAATTTGTTTCATTTTTGGGAAATTTTTGTTAATAATTATATCTTTTTTCTTGAATTCAGTGGATTATTACAAAATTTGAGTTTCGGAAATTGTCTTAACATTTACTCTCTTTTAGTTTTTGTAAGTTTGATTCACTGCCTTGGAAGCACCTAACTTTTCTCTGACTTGTACAGGATCGTGTGAGGACAGAAACTTATAGGGCTGCAATTATGCAGAATCAGAGTTTTATTGAAGGCAAAGTAATATTGTTGTTACCGTGGTTTCAGTTATTTTCCTCATAAAAAAGCTTATATTGAGATAGTTTATTGTCTTTTTTTTTTTTTTTAAATGCAGAATCAGAGCTTTATTGTCTACATTTCACTTGATACTTGTATTTTGCACATATTTGAGCAACTGTTTCAGCAAACTATGTTGTGCACCTCCCTACTAATTGATGTGATCATCCGTGCAGGTTGTAGTGGATGTTGGCTGTGGCACAGGCATTCTTTCTATATTTTGTGCTCAGGCTGGTGCAAAACGGGTATATATTCTTTAAAATGCACATAGATATTTAAAAAAAATTGTTTTAGCTTTGAAGCCAATCATTTTGCTAGTCCATGCTCTTGTCAGTTGAAAATAATTGTAGGGCCCTAATGGAATTGATGTACTGTAGCTTTTAATCTACAGTCGCTGTGAAATGAAATCACTAACTGCTAACTAAAAGTGGAATAAAATCACTAACTACTAAGTTAAAAGTGGGTAGGTGAACAGTAAACACAACTGTCAAGTAAAAAGTAATTGTGCTTTCCAAAATTATTAATGTGAATCAGCCGTGGAGTAAAATCAGATTAAATTAGAAACAAAGCTGAAACACAACTGTCAAGTAAAAAGTAATTGTGCTTTCCAAAATTATTAATGTGAATCAGCCATGGAGTAAAATCAGATTAAATTAGAAACAAAGCTGAAAGTGTTTATCAAACACTTCTGCTGATGCATTTTGAAAATTCAGCTGAAACACCGCAGTTACAGACGGGCCTATACGATGTTTGTCCAAAGAACACAAGTTTATGGTGCACATAGTTTCCATGCCATTCTCTTTAAATACATATGAGGACTGCACTTCAAAGTATATGCTAGTTCATATAGTGTAAATCTTTTATAATTGATCAATTAACTTGCTGCATTCAACATGCTTTTTTTTGAACTTGTGATATAACTTTTATTCCGTTAAACACTATTCGAGGTATTGGAAAATGTTATATTACTCTTTCAAACAATATATAGTTTAGTTGCTTAATTGTAGATTAAAAATTAATTCTTTCCTTTGCAGCAGAAATTTGGGGTAACAATTTGGGTATAACAATATACCCAAATATCTTTTACTGTTCAATGGATTCAATTGAATCCTCTCACCATTTAATCTTGTTTTTGTTATAGTTTAGATTATACTTAATAGGTTTTTTAATGTGTATGGTCTTGTTGTTGTGTTGCCAGGTGTATGCAGTGGATGCAAGTGATATTGCAGTGCAGGTACTTTGTTCCTGTAATGCTTTTTTTTTTGGCATACATGCTGCATTTTGTTTCAGCTATCTAATTTAAACAATGTAGTCATGAGAAGTGTGTAAATATTCTGTGCGCTTGTTGGCTATGGAACCTTGGTAGCATTTTGTGAACTATTTCTTGATAGATTATTCCAGGAATTATAGTAAAAAAAGAAGTTAATGGTTTCTTTTAAAAAAAGAGCTAATGTAGATTAGTTTTATTATTTAAAATATTGGTTTGGAGGAATGATAATATCAGCTTTATCTTGTGTGCCAAAGAATGGGATCAAACTAATTTCATTTTGTTATCAAATGTCCGGGTTATTTGTGCTCAAATTATCTAATAATTCCCTTTGGGATTAGTGTTCTAAAATCCTTCATCATCACCATACAACTTTATTTATTTGAGCATTCTTCCAGCAAGGCAAACCAAGGAATCTTTCAGTTATAATTAATCAAGTTATCTTAATCAGACTGATTCTTTTTTCTTACTTCCCCAAGAACGATTCCCCTTCTTCATAAAAGATTACTTGTCAGTGTTTTCTACATATGTTTTGGAAATTTTGGTTCACACTCACTTTTTACCAGGGCATTACAGGGATGGCTTAATTGCTTATGATTTGCTCTAGTTAATTTTGTGTTAAGAGTGAGTTCTTTACATGACCTGGTTGTTTCTTGACCAATATTGCTAAATTTTTTTTTTCCCTAACACTTTTGGTACTTCCCTAATGAATCATTTGAGACTTTGGCCCTTTCTCATTGCTGGTTCACAGAATAAAGTTTCTGCTCATTTAAGTAGGATTAGACCATCTAGCCTATGTGGATATTCTGGTTCTCTCTATATTTCCCCTTCTCAAATTCCCTCTTCTTCTTCATCCTTTCAGTCTTGATTAATTAACATTTCTTCGAATACTTTCCTTGGATTTATGCTATGTGCTAGTTATGTCTCTACGGAATCCACATTATTGATATGTGTCACTTCTTGTAAGAATGCATTCTCTGTGGGAGTTTGAATCCAAGTGGGGTTTGAAATGACCTTTTTTATTGTTGTGAATAGGCAAATGAAGTCGTGAAGGCAAATAACTTAACTGACAAGGTCATCGTTCTACATGGACGAGTAGAGGTGAGCTTCTGTAGACTGGCTAATATGTTGAATCTCCTGTAAAGAGTTGCCTTCGAGCTTCTATATCTAGGTTTAAGTGATAGATTAATTTTGAAATTCGCTTAAGTTTTGTTTTTTTTCTTAAGAAACCTCAAACTTTCTTATTTCATTCAGTTTTGCTATCTCTGGATAACAGGCATGTTTTAGATCTTAGTATTTTGATTTCTGCAGGATGTTGAAATTGATGAGGAGGTTGATGTTATAATATCTGAGTGGATGGGCTATATGCTTTTGTATGAGGTGATTATTAAAATATTGCATATTTGATTACATATTGAAGTGCTGTTTGTAAGCATGGAACTATAACTGGAACACATCAAGATCCAGGCATAACATTGTAGTTTCCAACTCTAACAGTGGATGCCTCTTTTTTGTGGTTAAATATTGTAGAGTATGCTGGGAAGTGTCATTACTGCCAGAGATCGTTGGCTAAAACGTGGAGGTCTTATTCTTCCTTCATATGCAACGGCATGTGTTCTTTCTTACTCTGCCTTGTAATCAATTTTAATCCCGGTTTGGGAGAGTCATTACCATGGTTATACTTCCAATTTACTTCATTGTTTTCCCTCTCAAGCCAAAATTAGTTTGTTCTGCATTAATAATTGTTGTTTTTTGTAAGTAATAACTCAAATCTATAACCCATAAGCTTAAGCTTATAGGGAGGGTCTGATAGTATTTTGCTCTAATGATTTTTATTTCCTCTTTTTCTCCAGTCTTGTGACAACGTTATTTGGTTGGGGCTCCATTTTATTTGACTGTAGAGTGGATCGTATATTGCTGATTCTTGTTCTTTGCTGCCTGCAGTTGTACATGGCACCAGTTACACACCCTGACAGATACAGCGAAAGCATTGATTTCTGGCGTAATGTTTATGGAATTGATAGTGAGTACTAACATGTAAATTTTACATTCAATTTTCGTCAGTAGATGCGCTCTTGGAACAAAATGTTAATTATATTGATCACTTGTCACACTTATTTGTATGTTTTTTTTTTTTTTCCTTTTTAATTATATTGATCACTTGTCACACTTATTTGTATGTTTTTTTTTTTTTTTCCTTTGGCTTAGTTCTGGTTTCAACTATGTGAATATTTGAATATTGTCCCATTCTTAAGAGTGTTTGTCTTTTAAAATTTCCTTTTTCAATCAATATTATCTTTGTAGTTACTTGTGATCTAGCAAATGTCATATTATTGGTTGCAGTGTCTGCAATGATGCCACTAGCCAAACAGTGTGCATTTGAGGAACCATCTGTGGAAACAATAACGGGCGAAAACGTTTTGACATGGCCACATGTGGTGAGTCCTCTCGTCTTAAATATATAGTTTCATTTTATTCTAAAGTGGGTGGGCAAAGCCCAAATCCATTTTTTGGCCCCCTACCTCTCCTAAAGATTCAGGTGGCCAGCCAAATAAAGTTACTTGATCACCACTTGGCCAAGCAACTAATATTAATGTCTAAACTTGTCTATAAGAAGAAAAGATGAGCTATTCTCGGGGCTTATGTGGGTCATCTAAGATGATTTTTTCTTCCCTGTGGTTAGAAACATTATTAGATATTAAGTTCTAATTGTATACCATAGAAACATTTATGGCCTATAACTGGACTGTGATGTCCCTAAATTTGATGGCCCCCACAAGGAGAAGAGTATTGTTAGATAACCTACTGTATAATTAAGGCTTCAAATTTTTGGACACAAATTAGATGTGCCCTAATATCCATGGTAGCATAATGTTCTTTGTTAGGCTGGGACAAGAGCTGTTGTACAAACTATTGACTTTTACTTTGTATAATTCCCTTCAGGTTTTGAGACAGTATGAAGGGTCATCTGTATCATTTTGACTGTGACAATTTTAGCGGAGTCATGGCTCTTCTAGAATTTCGTATGTGATTGTCAAGAGCTTAGTGAAGTAAAGGGAACTTTAAGTTTGGTGATGTGGTCTTGTGTGCTTTGACCTGAATCAATTCCTTGAGGTTGTGCCTCTAAGGCTGTCTGCTTTAAATGATGCTTAACTTGCTTTATGTGTCGTACAACTCTGATTAAGCATATGGTATTAAAACTTTGTATTTTGTCTGCACTTGCTATGGACAAATTTATAACAATTGCCATTTACAGGTTAAGCATGTAGACTGCTATACCATTCAAATTCATGAGCTAGAATCTATTGCAACAACGTTTAAATTCAAGTCAATGATGCGCGGTACTCTCTTTCACTTATACATCATATACACCACTTTTACCTCTTCATAGTATTTATATTTTGTCAATCCAACCATTGAATCAAAATTTATGTTAATATCTGAAATGCCCGTCAGATATCATACCAAGTGGATACTGATCCATAATCTCACGTTTATGTACAGTAATGAAGTTTTTTTCATTGGAAAAAAAAAATTAAATTCAGTAAGATAGTTCTAGTATCCAATAAACTTGAACTTGGTGCATGTAGTGAATGCCAAGGGCTTCGGCTTCAAATAGTGGTTCGGTCAATTGTTAGTCCTAGGATAAGATATGAAGTGGTATTAGTTAAAGGAAGTTTCTGCTAGTTGTAAGTAGTATAAACTACAAAAGAATCCATTAGACCATTTGGTAGTTTATGGCTTACTTTTGTCTCTATTCTCTATTCTAAACTAAACTCTCTAGGCTTGCTTTTGTTTCAAATCTTTTTTTTTTTTTTTGTTATTCACTTTACATTTCTAAAATTTTTACTTTCTTCCAGCACCACTGCATGGGTTTGCTTTTTGGTTTGATGTTGAATTCAGCACCCCTGCAATATCTCCTGCCAATAACCACATACCACCTGTAGTCGTTGGTTCATCTAACAATCATCCAATGGATGGTTGCCAGAAGAAAAAGCGTGCAAATCCTAATGAAGCACTTGTGTTGTCTACTGCACCTGAGGACCCTCCAACACATTGGCAACAGGTATTGAATAGTTTCACGTTCCAGCAAGAATTTTCATGTCTTGTTTACGTGGTCAGCTCCTTGAAACCTGTTATTAGTCAATAGAACTGTGACAGTAACATAGAAAAAATAAAGTGCACTTGCAAAGTAGGTGCCACATACATAGGAATTGGATATGAGAAAGCTCCCAGTGTCCTTTAGAGATTGTTGACAATCTTGTGGTTCACGATTTTGGTGAATGTATAACACGGTTGCTTACACCATTGATACTACCCTTAGTTATGTGGCAAGTGAAATGAATGATCTCACTGCACCTTTCTCAAGCCAAATGGTGTTTGATGAATGATGTGCACGGTTGTTGAATGACCTAAATGATGATTCAGAGTGCATAATTAAATGTAGATTTTAAAATCCTTAGACATCATTAGCCGATAGATCGGTTAATGCCATGATCGTCTGATGTACCAGCAATAGTAACATTTCCCTTAAAATAATCTCTTCAGTTTAAGCAGCTTGGAAAATTATTTTGCCTATGTCACCTAAAGGCATAGATGACCAACTCAGTGTGCAGTGGATTGGTGACAACACTAGTTGATTGTAATTTGATAATTTAAGAAATCTTGGACAATGATATTGGTATTAAGTTATTGCCAAAAAGAAGTCTGGTCATTTTCTTTGCTCTTTTTTACTACCAGTTTGCTTGGTGTTGAAAACTTGTGGAATGTTTTACATGAAGTTCGTTGTGTGATTGGTTTACTTCTTTGCAGACAATGATCTACTTTTATGACCCAATAGAGGTGGAGCAGGATCAGCTCATTGAAGGCTCTGTAGTACTGTCACAGAGCAAAGAAAATGCTCGATTCATGAATATTCACCTTGAATATGCGTAAGTCATAGCATATATTTGTCAATCCTCATGCTCAATTCTTGATGGTCAAAGTGGATATGGCACAATTTCCTGTTGCTGATCAATGCTGCGATCTTTGTTGTTGCAGTTCAGGGGGTAGATCCTTTGTAAAAGAGTCAGTGATGCGGTGATCCTTGTAAGCATTCTGCTCCTTGTATTCTGTTTGGGTTTCTTACACTGAAAGCTTCGTGAGTCTTGCATGGCTGAACATTCAAACTTTCTTGCCCACAATTACGGGCAGCTGTTTGTGCCCTAATGGACTGCCTTAGTTGACCTTGTGGAGGTGGTGGATCTCGCTATGTTTACTTGGCAAAACCTCGAAAAGGTGAAACATTTTCAGCGAATTATAAATGTAAATGTAGGCGAACCATTTTGGGAATGTTTTATGTGAATGTACTTACGAAAGGAAGTATCAAGTGCTGGGTGAGGTTGTATCTTTTCATATATTATAGGTGCTAGCTGTAGGATGAAAATGTTAGAAGTTTCTTTTAGAGTGAAAAATTGGTTCATTGTGATCGGGTTCAAAGACACTAATTTCAACTTCTGTATGAGTAGTTCAGATTTGTTTCAATGTTGTAACATCATTGTACACAGCCATGATTTCAATCTACCCGTGGACATTTACCCGGTTTTT

>CsPRMT6

ATGCTCTCACTGATACCTAAAGCCCTAACCCTCTCCCTTTCTTCCCGCACCACAACTCGTTTGATTTTGATCCCTCTCCCACTCAAACCCGTCGCCTCGCGCCCCATGTCGACTCAGCGAATGTTCCAGCTGAAGCTCGACCCACTCACCGGTAACTCGGAGTGGGTTGTCATCGAGGAAAACGAGGATGTTCCGGAAAGTTCGCAAGAACCGCTTCTGGCCACGACGTCGTATCTTGACATGCTTAATGACTCGTACAGGAATAGAGCCTATCGCCTGGCCATCGACAAGATGGTCACTAAATCTTGCCACGTGCTTGATATCGGGTACCATTTCCAACAATATTCTTTTAATACAAGCGCTTCCAAATAGTCCTGTAGTTTAATAGAAGTTCCTTCCATGAATTTTCGAAGTCATTAGAAACTTATCTGATTCTTAAAAAAAAAAAAAAAATTAAAGAAAATAACTTGGCTTAAATTGTGCCAAAATTTGTCAGGAGCCCATTGTTAAATTCGTCTAGCTTTTCTTAGAAATTACATTTTGTCAATTCTTTTCTCAAACAAGTTTTAAACTGTTCCATTTTTCTTCCAAAATAAATATTTTAATATATCTCAAATGAGTAATTTCTATTATTGTGCAGTGCTGGAACGGGATTGCTTTCAATGATGGCTGCTCGGGCAATGGGTTCTAGTGACTCAACAACTAGTCTTAATACCAAAGGAATGGTTACAGCTTGTGAGTCTTACCTTCCCATGGTGAAGTTGATGAAGAAAGTTCTGCATGTTAATGGTATGGGGAGGAATATCAAAGTTATTAATAAGCGTTCTGATGAACTTGAAGTCGGTGTTGACATTGATTCTCGTGCTGACATTCTTGTGAGTATTGATATTTTCATGTTTGCTATAACATCATGTTTTCCCCCTGAGAATAATTATACATGGTTACAGATTACTGAATGGTTTAAAGAAGGAAAATTTGAAAATTGAAATGTAATTAAATAGTAAGATGCTTCTCCTTTTGCAGTATCCCATAAAGATCAAACCAATATTATTTAAAACTTGTGCCAAGGTGCAACTTCGTGTTTGAAGTGGGGTCATTGATAGACACAGGAATGTCTTATATCTGCCATTGTGTCCATCACAATATGTCAGTCTTATCAATTATACACAGCTGGGAGTTGGCTTCTTTTCGTTTCCTAAAAATTATTACATTTTCAATCTAAAATGCAGGTCAGTGAGATACTGGATTCTGAATTGCTGGGTGAGGGACTAATACCAACTCTACAACATGCACATGACAGGCTGTTGGTGGAAAATCCACTAACAGTACCATACCGAGCGACTACTTATGGTCAGGTATCTAGCTTTCTTCTTCTTCTTTTTTTATGGCCCAATATTTCCAGCCTTTTACAGAAAGGGATAGCTTTGAAGGTACTATTCCTATAGAATGGAGATTGATCAGAGTTCAAAGAATATGTTGTCTTTTAAGTCAAATGTAAAGTTGGGGCAAGCCACTGTTATCATCAAACTTTTCAGATAGTAGGACCTGGACTGGTATAACTCATGTTTGGTTGTATTCATCTATTATAATTGCAATTTCTCTTCCTTACAGCAAACATGAGCATTGGTTTTCCCACTTACATTGCCATGCTTGTATGGCAGTTTAACTTATCAATTAGGGGCCTTTTGGCAACATGGAGTCTGTTTAACAGCGAAAAGGTTAGTTCTCTAAGATAGACAACATTGCAGTTGAAGATGGTGAAAGACAAAATATTTTAACTGATCCACAAGGCCTTCCTCCGTGACATTGCAGTGATTTCTTTTCTTCTAGTGGTTTTTCCTTATACTATCTGGCCAAACTGGACTCCACAACACTAATATACATTTAATGCTCTTTTCCCATCCCAATACATGATAATACTTGATTGTTTTTACTAAATTTTTTATTGCCTCAGGGGCACTTTGATTTTGCTATCTGCTTAGTCTTCCCTTAGTAGCTCATTGCTACCTTACTGTATTGTATTGATTTTGCTCTTCTTTGGTGGAAATCATATTTTCTAATAATTTTAAAGCTCATACAAATGTATTTATCTCTTCTCTGGGTTTGTCTCCTTTCTTCCCTTTTTAAGCTGGTTGAAAGCACGTTCTTGTGGAAGCTGCATGATTTATATAATAATGAAGCAAAAGCATTGGACGGCATTCATCTTGTCCCAGCTGGCATGGATTCTATCTTACATGTCAAATCACAACAATATGCCATGCACTGTGATGCAATAACAAAAGAAATCAAACTGGTAATGATGTTAACCACTCTTCTACAGAATTCTTAGAAAGTACCAGTAGTTTAATTCACACTCATCCAGCTCTCAGAACCCTTCAAAATATTTGAATTTGACTTTTGGAAACGGCCAGACAGTCATGGAGAAACTGAGCTGCAGATAAAGTCAACTGATGATGGTAGAGTCCATGCTGTAGTTTCATGGTAATTATATCTCTGCTATTTGTATCCGAATTTATTTTATATGGGGCATCCCTGGAAATTTTTGTCTTTTGTTTTCAGGTGGGTTCTTCAGCTTGATCGAGAAGGGACAATCTTTTATTCCACTGCTCCTAGATGGATAAGTCTGCCAATTCACAAAAGTGAGTCACTGTCACCATAATATCAGTGCTCTAGTATCATGGACTTATGGTATTGTTGAAAGCAAATCAAGTAATGTAATTAAGAAATATATATATATGAAAAAAAAACCTCAGTATTTCCATATTGTTTACATCTCTCTTTGTTTGGATATAGAATTTGGCCATGTACTTTCAACAGTTACACACCCAGTTTTGATTCTGACTAACACAAATATGCAGCTCCCAGATAAAAGACTTAACAGTGATGAAAACTAATAGCTATAGCAGGCAAATTATATCATATGGCAGTTCTCTGTGCAACTATACTTTTCTTCTGTCAATTTCTCCAATTTATCAAAAGAAAAAATGACTAATTTTATACACATCTTGGATGCATCTGTCTTTTAAAAATGATGGCAATGGTGAGATGCAAAGCTTGCTATAAAATAACTTTAGAAGACATTGTTCCTTGATGTTACTTTTAGATGCAACCTTAAAAGTTTTGTGCATTGCTTTGAAATTCAACCTTCACAATTTCATGCAGCCAATACAACTAAAAGATCTTGGTTTATGTTGAACTAAATAGGGATCTTTTTTCGAGCTTTGAGCAATTTTTTTTTTTTAGATGATTAAGAAACTTACATGGCATTTCAAAATTTTTATCAGAATAGTTTAAAGATCTATCTCCAGCTAAGAGTTGCAGTTAAGTAAATCTGGAAAGATGCTTAACTTAAAATAAGAATAACCTTTCGAGTTTGCTTGGTAAAATGCCATGGTTTCATGTTAATTTGACAAATAAAGCTTGTTTGATGCTTCAGGCACTGGGAATTGGTGTGATCACTGGAAGCAGTGTGTTTGGTTTATTCCAGGTAAGGGTATGTCCATATGCAAAGGTGAAGAATTGTTGTTTCATGCTTTGCATACTGAAACTAGTGTATCATACGAACTCAAGTCCCAAATCCCAATAACTGACGAGAGACAGCATAACTTAAATGCTAAGGATTTTCAGCTCGCATTACCACCAGAAAGAATTGCCATCTATGGTGATGGCGAATGGAGGCTTTCCATGGTAATGGCCATGAGAAATGCGGTAAGATCTTCTTCTCTCCTTGCGTGCTTATTCTCACTCAAAACTGAGCTTATACACTCATTCTATCACAAAAGTAAAGAGTTACCCAAGGAAATTGAGCACCAAAAACTGAGCTTATAAAATCTGTTTTCAAAATATTTGGACTGTTTCCCCAGGTCTAATGTTGATCAAGACTATGCTTGTTGATGTTACCTTGTCAAAATCAGTCTCAGCGGTTATTAGTCAGGTTCTTACTACTCAATGTCTACAACAATGATACCACAGTTTTGGCTATATCTTCATGCCAACTGACCAGTTGGTAGGCCAGCATACCTGTCGAACCAGAAATGGAAGTTGGAAGAGAACCATTAGAATATGTTGGCTTTTCCTCTCATGGAAATACACTGCTAGGATATCTATAAACCTCGATACCTATTAGATCTACTATGAAGCCTAATAATCATAAGACACAACATCAAAATATAAAGCAATACGACGTTGCATAAAGTAACTGGCTTATGATAGTTTTTGTTGCGTAATTAGCAAGTGTACTAGTCGGCACAAGTAATATAATGAAGAGTAGAGTATCGTCCCACTGAGATTGTATATCCTATTAGCTACCGAAATTATTCTAACCCTGCTTTATTTGAACAATCGATTTAGGCAAATTTAGATTAAAGTCAAATAAAATAAAATAATTAAAAATTAAATGAAGATGCAGGCAGTAATAGAAATCACCAAGAGATTAAAACACTAGGACATCCGATTTCACCATAACCAATCCAATTTAATCCTCCATCTATGCTATTAAAATTTATTACCCATGTTGACAGTGAATTTTCCTAACTTATTCAATATCCTCTCTCGAGTAATATCAAAAATATCTCCACATATCAACCCACTATATCTCTATGTGAATTTAAATATGCAAAGATTCATTAAGCTCTATGAAAATTCTTAGAAAAACTGCACGAATCACGTTGGCACATCTCTGTCTTTCGTTCAATTCATGACATTCATTACACAGAGCAAAAATACATAAATCTCCTCTCGGTCTCATTATGTATCCTCAAATCATTCAAATATTGGCCAGATATTTAAAAGCATTAAGCACAATAATGAACACTCAATCATGGAATAATTAATTAAAAATAACATAGATTTATAGAATTAAATCTTCATAGTTAGGCTACATCGTAGCCCTAGTAAATAAATTAGTTCATGGGAATATAAAGAGAATTCATTAATTTAATTGGAAACATTCAAGCAAGATAATAGAGAAGAGAGAAAGAAAAACTCAAGAATTCATGCGTCTTCCTCATGCTCCAAATTATTCCTGCTGCTCTCGGTTTTGCTCAAAACTTGCGGCTGCTGGCCTGCTGCTCTATTATTTCTTTTCTCTTGGCTTCAATTCTTCTTGTGCGGCTGCCTTTCTATATCTGTTGCTCTCGCTTGGCTTTTCCCTTTTATAATGTTGTGACTTCTTCCAAATCCTTAAATGAAAAGGATTAAAAGCAAAAGCCCAGCCGCCTGTTTCATTCCTAAGCCAATCAGTATTCAAACCAAAGCCCATCCACGTGAATACCCTTTTCAAACAATGCCAAAATATTTGCTTCCTTTATTATATTGTAAGCTTGCCATTTAAATTGTCAAATGGGCTCCACGTAACTAGCTTGAAATTAAAAGCCAATTGACCTTTTCTTCTCTTCCTTTATTCTTCACATGATTGCCTGCATGATGCTTCTATTCAATCTTCCTTGATTTGGCTTTTTATTTCTTTCCCTCTTGCTTCCACATTATTTCTATTATACTCCAATTTGTTTCCTCTTTAAGCTTCTTTTCTCATGAATATTCCCAATATTCCTACAATGAATAAATATAAAAATTAAGATAAAAATTAATATAATAAAATATAATTTAACATAAAATTAATTATGAAAGCATTAAAATAATAGATAAATTATGAGCACATCAGTTTTTATTTGGAAAGTGATGATGTGAAATTAAAAATGCAGATTCTCAAAGTTTCCTTGCCATTTGGCCAATGTCTAATGAATTAGCTTGATTAATTTTGAACCATACCATTGTTTAGGCTATAACTGAGTGGGCGGATTATATAGCTTCCCTTAGTGATTTATGTCAGAGAAAGAGAACTTCGAAACAAAATTTGGAGGTTTAACAATTCTAGAGTGACAAAAGTGATTAGATGTGTTTATCTTTGCAGTCTTAGAAACCTGAATTATAGCTCAACCTCATGAAACAGACGGGAGAATCTTCATATACTTGGTGGGATTTTTTTGCTTCTTGGCTCCCATTTTAGACATGCCAAGTATCATGTTTACTCACTATCAAGTCCTTACCATCAGTCCTCAACTTGAATGTTGAGACCATCTTTTTTGCTTTTTCCCCTAAAATACTAACGAAGCAACCCGAGACTGCTGTACGGCCAAGGCGGATGTACGGTACAGCAAATGAACAAATTGATGTGTAATGAAAAATAAAGAGATAAGGTAAGAGAGAGCAAACGCGTGATTTTTACGTGGATCGGCCAACCGTGCAAAGCAATTAAATTCGAAATGAACAAATTGATGTGTAATGAAAAATAAAGAGATAAGGTAAGAGAGAGCAAACACGTGATTTTTACGTGGATCGGCCAACCGTGCCTACGTCTACGCCTCCAAACTCACTGGATTTGAGGATTTCACTAAGCGAGCCTCCAAGGCTTTAACAACGCTTACAATTGACTTTCAATGTGTTAATAGACCTTTACAACAAAGTAATTAAATTCGAAATAAACAGATTGATGTGTAATATTCCTCGCACAATGTATTTGATTACGAATGAAAGCCCAATGTGTAAATGAATGAAATGAATACACAAATTTTAAGCTCAGATTGTTGAAAAATGCTCAATGATAATAGTAGTTGTCCAACACTCATTTGAATTTGATTGAGTCATTTTATAGTTGAAGTAGAAAATTAGTCGTTATTGATTTTCTGGTGCTAACTGGCTCGCTGTTTATGCTAACTAGTTTGCTATTTGATGTTACCGTTATAGTAAAAAAAGTTAAATTTTTGTATAACCAGTTTGTCGCTTTTCATTATCCGGTTAGCCGTTTTTATTTTAGAGAGCTTCTGCCTGATAATTAATTTGCCACTTGCTAAATTTGGTTAGCCGTTTTGATTCCAGAGAGCATCTCTCTGGTAATCGGTTTGCCATTTATTTTATCCGGTTATCCGATTAGTTCCAAAAATACATTTTTGTTTTAAACATACTCATTTTCTTGTTTTATAAGACCATAAAATGTAATTATTTGTTTATCCAAAACATGTGGCTTGTTATCATCAAAATCAACATTTTATAGCTATAGAGGCTAACACCCTAGAGTTTGAATGTACACATTTACTATCCTTTTTACATGAACTGTATTCTTTGCAGTTGCAGGGAAGAGTCCAGCCGTTGTGTGTCGTTGCTGATGACAGTGTTTTCTTAACAATTTGTGTTGCACGCCTTTCAAAAACAGCACATGTTTTATCATTGTTACCAGGTCTAGGAGACAAGGGTGCCCAATATTTGCGCACCGTTGCAGATCCAAATTGTTTCTCAATAGATCGTGTAGAAATTCTTCAAAAGGGGAAAAAATGCTTGACTATGGATGACACTCAACAAAAGAAGGTAATTTTCTCCACTCAGATTGTATACAGCTGTCATAAATTTGAAATACTGCCCTGCTTTACTTCCATGAATCTGTTGAAAAGTTGGTCCACATGAAACAAATATTTTTTGTGCTTCCATTTTGTGGACATTCTATGCTGATCTAAGCAGCTAATCCACAAGTCCTCTTTTACCATTAGATTAAGAGTGTGTTTGGGATTGAGGTGTTGTAATTTTTAAACTACATTTGCTATGGAAAAAAAGCTGTAACTATGAAATAAAAGTTAGTAATATGTAGTAAATGTAAATTTTAAACAATAATTTTGATAAAATTATTAAAGATATAATAGATTTTCCATTATATAAGTGAAAAATCATATTAACATAGCTTTCAAGGTACAACAGTTAGTGTTTACTAAACACTTTAGTGCTGTAACTTTTAAGCTACAATTGCCCAACCTCAATCCCAAATACACCCTGAATAGGTGATTTTCAAATTCCAAAGCTTAATAGAATACTTCGGTCATATGTGTTATCCTAGTCCTATTACTTGAGAGCAGCACTATGGCATTTTGGGTGCGTAATATGAATTGTACATCTTTTTGACGGAATGTAAACAATTTGGTATGCTTTTTCTATCACAGGTTGACCTGTTAATTGGAGAACCATATTATTTTGGAAATGATGGCATGCTTCCATGGCAAAATCTGCGGTTCTGGTATGTAACACAAATTGATTTCTGTTTTGTATAAGTTCTATTCCCATTCTTTGTGCTCTATTTAATTTATTCTGATATTTAACAAATTTCTCTTAGTTTCTTATAAAATAAAAACTAAATGACTGCAATATTGGTTCAGACCTTGTTAATGTGTTTACTTTACATGCCC

>CsPRMT7

ATGGCAGGAGGGAAAATAAGGAAGGAGAAAGGAAAGCAGAAAAGTGGACCGTATCAAGGACAAGGACTAGGAGCAGGAGGTATATCCTTCCACAAATCAAAGGGGCAGCACATTCTGAAGAACCCTTTGCTTGTGGAGAGCATCGTCCAAAAAGCCGGCATCAAGAGCACCGATGTCATTCTTGAGATTGGTCCCGGCACTGGTAATCTCACCAAAAAGCTTCTGGAAGCTGGCAAGATGGTCATCGCTGTCGAGCTTGACTCTCGTATGGTTCTCGAGCTCCAGCGCCGTTTTCAATCCACCCCTTACTCCAACCGTTTAAAGGTACCCCCTCTTTTCTTTTTAATCTTTAATGGTTTCATACTACCATTACAGTTCCTGCCTTATATATTTATTGCCTCAGAACAATATTATCAGACTAATCTCTTTCTCAAGGTTTAAAATCCTTTCAGACAAATTACTATGTTTCTAGCTAATAACATTTGTTTTGCATTAAGCACTGTTCTTCTACTTCAGAGAGAGTGTGTGTATGTGTGTGTTAATTAACATTAGTATGCTTTAACTAATCAATCACATTCCCAAATTTGGTCTGCTTTAAGGCAGAAGCAGAAACAATGTAGGTTTCCTTTTCTGACTATGCATGTGCTGACATTCTTAATTTATTTGAAGGAAGAAGATGATCGGAATGTTGTACGTTTGTAAAATTAATAGAAACTGATTTCTTTGCTGTACAGGTCATTCAGGGTGATGTCCTCAAGACTGATCTTCCCTATTTTGATATATGTGTGGCAAACATCCCGTACCAAATCTCCTCTCCACTCACATTCAAGTTGTTGTTTCACCAACCTGCCTTCAGATGCGCGATTATTATGTTCCAGAAAGAATTTGCTATGAGACTTGTTGCTCAACCCGGTGACAAGCTCTACTGTCGTCTTTCTGTGAATACACAACTTCATGCTCGGGTCTCCCACCTCCTTAAAGTTGGGAAGAACAATTTCCGTCCTCCACCTAAGGTTGATTCCTCAGTTGTTCGAATTGAGCCTAGGAAACCGCGTCCTCAAGTGAATCCTGTAGAGTGGGATGGATTTTTACGGATTTGTTTCATAAGAAAGAACAAAACCCTTAGCTCAATTTTCAGGCTGAAAAATGTCCTATCGATGCTTGAAAAGAACTACCGGACCCTACAGGCATTACAATCATCACAGAATAGTTCCTTGGGAAATACCGAAATCGGAATGGATACACCAAGTCTGGGGGATTCTAATGGGGATCAAAGTATGGGGGTGGATGATGGAAGTGATGACGAAATGGATGTGGAAGATGATGATGGCGATAGTGATGTTGAAGGTGAGGTGTCCGAGTTCAAGGACAAAGTTTTGGCTGTGTTGAGAGAGGGACAGTTCGAAGAGAAGAGGGCTTCTAAGCTAACACAACAAGAGTTCTTATATCTTCTCTCTTTGTTTAACAAGGCTGGCATTCACTTTTCTTAA

**CsHDMs:**

**CsHDMAs:**

>CsHDMA1

CAGCAAACTCCCTTACAAAAATTAGCGCACTCTTTTACTTGGGGTTATAGTCTTTTCGGGCAATTTTTTAAGCCGAAGGACTTTATGATAAACCCAAAATGGAAACACCGCAAGAACCCTCCGATAACGCAAACGACGATGTCGTTTCCGACGAGTCTTCGCCGGAGACCGATGCAACGCTCTCTCCCAGTCAAATCGAAACCCAGGCGGAAACTGATGAGCTCCAGAACGCCACGGAAACAAATACTGCACTCGAGGCTCCCGTTTCGGACTCGCTAGACGACTCTTCTGATCCAATTCCAGAAGACCAACAGCCCCAAAACCCTAACCCTTCCGAGCCGGGCCCGCCCCCGAGGAAACGACGCCGTAGAAAGCGCTTCTTCACCGAAATCAACGGCAATCCATCCTTGGCGAGGAACCGACGCCCCAGATTCTCCTGCCTTGCTAAAGAAGTCGACACCGAAGCCCTAATTGCAATCTCAGTTGGTTTTCCAGTTGATTCACTTACTGAAGAAGAAATCGAAGCGAATGTAGTTTCCAAAATTGGAGGTACCGAGCAAGCTAATTACATTGTCGTGAGAAATCACATTCTATCACTCTGGAGATCGAATGTTTCTGTCTGGTTGACGCGTGAGCAGGCGCTCGAGTCAATTCGTTCCGAGCACAAAACCCTAGTTGACTCAGCGTATGATTTTTTACTAGAACATGGGTACATCAATTTCGGGCTAGCGCCGCCTATTAAAGAAGTGAAATTGGGGTCATTTGGTAGAGTGGAGAGGGGTAATGTGGTGATCGTAGGTGCTGGTCTTGCGGGGTTAGTTGCGGCGAGACAGTTGATTTCAATGGGGTTTAAAGTAGTCGTTTTGGAAGGCAGGGAACGTCCCGGTGGACGTGTCAAGACACGCAAGATGAAGTGTGATGGAGTGGTGGCTGCAGCTGATGTTGGTGGGAGCGTCCTCACTGGAATAAATGGGAACCCTCTTGGTGTTCTTGCTAGACAGTTAGAATTGCCACTTCATAAGGTGAGAGATATTTGTCCTTTGTATTTACCTAATGGTAAAGCAATTGATGCTGATATTGATTCTGGAGTAGAGGTATCGTTTAATAAATTGTTGGATAGAGTTTGCAAGCTTAGACATGATATGATTGAGGAATTTAAATCTGTAGATGTTCCATTAGGCGTTGCACTTGAAGCTTTTAGAAATGTTTATAAAGTTGCTGAGGATTTGCAAGAGAGAATGCTCTTGAATTGGCATTTAGCAAATTTGGAGTATGCAAATGCTTCCTTGATGTCTAATTTGTCAATGGCTTATTGGGATCAGGATGATCCATATGAGATGGGTGGTGATCATTGCTTTATTCCTGGAGGCAATGAATGGTTTGTTAGAGCACTTGCTGAGGATCTTCCAATATTTTATCAACGGACTGTGCAGAGTATTAGATACGGTGTTGATGGGGTTATGGTTTATGCTGGTGGGCAGGAGTTTCGCGGGGACATGGTTCTTTGTACAGTACCATTAGGTGTGCTTAAGAAGGGAACAATTGAGTTTGTACCTGAGCTTCCTCAGCGGAAGAAAGATGCAATTCAGAGATTAGGATATGGATTGCTTAATAAAGTTGCAATGTTGTTTCCACATAATTTTTGGGGTGGAGAGATTGATACATTTGGGCATTTGACAGAAGATTCAAGTATGAGAGGTGAGTTCTTTTTATTTTATAGCTATTCTTCTGTATCAGGAGGTCCACTTCTCGTGGCTCTTGTTGCTGGGGATGCTGCGATTAAGTTTGAGGAAATGTCACCGGTAGAGTCTGTGAATAAAGTATTAGAGATACTGAGAGGTATTTACCATCCAAAAGGAATTGTTGTTCCAGATCCAGTTCAGGCAATCTGTACTCGTTGGGGGAAGGATCGATTCTCATATGGGTCTTACTCTTATGTTGCCGTTGGTTCTTCGGGTGATGATTATGATATTCTTGCGGAGACTGTAGGGGATGGGAGAGTTTTCTTTGCAGGGGAAGCAACTAATAAACAATATCCGGCGACAATGCATGGAGCCTTTTTAAGTGGGATGAGAGAGGCTGCTAGCATACTTAGAGTGGCCAAAAGGAGGTCATTGGCTCTAACTAATAAAGCATATAATGAGAGTGAGGACAATGGTAATTTGGATAAGTTGTTTGAGACCCCTGACCTGACGTTTGGGAGCTTCTCTGCGTTGTTTGATCCAAAGTCTATTGATCTAGAATCTGATGCCTTGTTAAGGGTCAAATTCCAAGGTGAAAATTTTGATTCTGGTCATCTCTGTCTCTATGGTTTGGTAACAAGGAAGCAGGCCGTTCAGCTGCGTGAGTTAGATGGTGATGGTAACAGGATGAAAATGTTACATGATAACTTCAGGGTGAAGTTGGTTGCGAGGAGAGGTGTATGTAATGCTACGGAGTCTCTTATTACAAGAATTAAAGCAACTAGATTCAGCCTAAATGACGGGGTTTGAAAAGACTCTGGGCACGGAAAAGATAAGATTTTTCATTTGCTTTGAGAAATAGGTAGAAGTAGTTGACTCTCTAGCTTCCCACATTTTTTCCTCTCTTGATTCATTAAATTTAAGATGGAGAAAGCCAAAATTCAATTTTGTGGATACTTGCAGATTCACAGGACTAGAGATATTGGATTTGAATTTTGAACGTGATTTGATTTCATTGCAAGTATTTGTCTTTTTAATTAACTACATCATTGTTGTAACTGTAAGTCATGAAATGAAGCTGCTTTCAATTTGACCATCCTAATAATCTCCTCAAGTGAGGTTTCCACTTTCAAAATTCCTTTGTATCCTCTTAACTTTATTCGTTCAAAACCCCCCCTTGAATTGCATGCATAACAGAAACTCTTCTCCCCCCTAAAATGTCTCCGCCAACGCAAGTACAAACAGACTTGAAAACGAATGCAAACCCAACTCCGAAAACCATGATACTAAACTAGGCACAAAATGGGC

>CsHDMA2

CTAAATCACAATGCCTCCGAAGCATAACAGCTTCTAACTAATCTTTATTCTCAAATTCGAACCCCAAGAAGGTCAGTTGACTCTTCCTACTTCAATTCTTTGCATAATTCATATTTTTTTAATTCAACTTCGATTCCAAATTCATAAGTGCTTTGATGTTTGTTTTAGCCAAACTTGTTAGTTAAATTCTTTTGTGTTGATTATGGTTATTGATTCACAAGAATATTTGTATATTCGTCGTGGGATTAAGTGAAAATGGAAGGAAATGATACCCATTTGCCAATATTTTGTGTTACGAGTAAATGTTAGTGTGGCTTGCAAGACTGATGCTTCTATAGAAACAATTTGATAATTCTTGCTGAGAGAATTTTAGGTATATGTAATAATGAAAACCCCGGTGTCTGATGGTGATGGTTCTGTCTCAAAGAGGACGTTGAGGAAGAAAGTGGGTCTGCGGAATTATGATGAGAATTTGATGGATGAGTTGATAGAGGGGCATTTAGGTGGCTCTTTCAAAAAAAGGAATAGAACTAGAGAGGCTTTGGAGAAAGAGACTGAAACTGAGGCTATGATTGCATTTTCATTGGGGTTTCCGATTGATGCACTGCTTGAGGAGGAAATTCGAGCAGGGGTGGTGGGGGTATTGGGTGGGAAAGAGCAGAATGACTACATTGTTGTGCGGAATCATATTCTTGCTAGGTGGAGAGGCAATGTGAGGGTGTGGCTAACGAAAGGGCAGATTAAAGAGACTGTGAGTAGCGAGTATGAACATTTGATGAATTCGGCTTATGATTTTCTTTTGTATAATGGGTATATCAATTTCGGCGTTGCGCCATCTTTTACTGCTAATATGCCAGAGGAGGCAAATGAAGGTTCTGTGATAATTGTTGGTGCTGGACTTGCTGGGCTAGCAGCAGCAAAGCAGCTCATGTCATTTGGTTTCAAGGTTGTTGTTTTAGAAGGCAGGAGTCGACCTGGGGGTAGAGTTTATACTCAAAAGATGGGTAAGAAGGGTGAATTTGCAGCTGTGGATCTCGGTGGGAGTGTTATTACTGGAATTCATGCCAATCCTCTTGGAGTTTTGGCGAGGCAACTCTCTATTCCGCTTCATAAAGTCAGAGATAATTGCCCTTTGTACAAGCCAGACGGGGCACCTGTCAATAAAGAAATTGATTCCAAGGTTGAATTCATCTTCAATAAGTTGCTTGACAAAGTCATGGAGTTGAGAAAAATAAAGGGCGGATTTGCAAATGATGTTTCTCTTGGTTCAGTTTTGGAGACACTTAGACAATTGTATGCTGTGGCTAGAAGTACTGAGGAAAGGGAACTTCTTGATTGGCATCTTGCTAACTTGGAATATGCAAATGCAGGATGTCTTTCAGATCTCTCAGCTACCTATTGGGATCAGGATGATCCTTATGAAATGGGTGGGGATCATTGTTTTCTTGCTGGAGGGAATTGGAGATTGATAAAAGCATTGTGTGAAGGAGTTCCTATATTCTATGAGAAGACTGTGAATACTATAAAGTATGGAAATGAAGGGGTTGAGGTGATTGCTGGCGACCAAATGTTTCAAGCAGATATGGTACTTTGCACCGTGCCACTTGGAGTTCTGAAGGAAAAGACAATCAAATTTGAACCAGAGTTACCTCAGAGAAAGGTTGCAGCAATTGATAGATTGGGTTTCGGGCTCCTTAATAAAGTTGCGATGGTTTTCCCATATGTGTTTTGGGGTGAAGAACTGGACACATTTGGTTGTCTCAATGAACAGAGCAGTAAGCGTGGAGAATTTTTTTTGTTCTATGGTTACCATACCGTTTCTGGAGGTCCTGTGCTAAATGCACTGGTGGCTGGAGAAGCTGCAAAAACTTTTGAATCCATGGATCCATCCTTTTTGCTCCATCGTGTTTTAAATGTCCTTAGAGGTACTAAACATATTCAAGGACATGGGAGAGCACGGCAATAAGCTTCACAATTTTCTTTCCTTTTTGTACTGCTTGTGCCTGGAACTTTGATGTATCTTTTCTAAATGCTTCATTGAATCTACCTTCTGATTCATAAATTGAGTCTCCCTTTTGCAGGTATATATAATCCAAAAGGTATTGATGTGCCAGATCCCTTACAAACAATATGTACAAGGTGGGGCAGTGACCCCTTTACCCATGGTTCATACTCTCATGTTAGGGTGCGCTCATCTGGTAGTGATTATGATATACTTGCGGAAAGTGTAGGAAGCCGGCTGTTCTTTGCTGGTGAGGCCACAACGAGGCAATATCCAGCCACCATGCATGGAGCCTATTTGAGTGGCTTGAGAGAAGCTTCACGCATTCTTCGAGCTACAAGAGTTCAGAAATATAATTCAAGGAGGTCTCTTCTGAGAAATGTTGGATCAAGCAATGACATACTGCTGGATCTATTCAGGAGACCTGATATGGAATTTGGAAAGTTTTTGTTTGTATTCAATCCTTTAACAGAAGATCCAAAGTCATTGGGGCTTTTGAGAGTTATGTTTGAGAATTGTGAAGATGATTTGAGAAAGGCTTCAGCTAACAGCTGTCAGAACCCCTTGAACCTTCCACTCTACACTCTGATATCTCGTGAGCAGGCGAATGAGCTACAACAGGTGATCGGAGGGAATGAAAGTAAGTTGTCCTATTTGACTAAAAATCTTGGGTTGAAGCTAATGGGATCTAGTGCTTTAGGAACTGTAGGTAGTTCTTTGATTGCTAATATTGCTAATGCACGAAGAGGAAGGGGTAGGAACCGCATTGCTGCCGGGCAACGACAAATTACCATGTAGACATGCTATCAGTAAATTCTAGTACTTTTTTCCCTTCTCAGTTGCAAATTCTTGTACATAACATGATAGATCTCTAGGTTATTCATTAACTCCAAGAGAAATAAGTTTCTTGGAGTTTGACTGGGAAAGCTCATTTGTTTGTTGTAGAGGACGTTTTAGTTTTGTCAGTGACCAACATGGTAGTGCTTCATCCATAATGTAGCCTTGTAGGTCAAAGACTGCTTTCTGGAAACAGTTTGTAGCTTGTAGATTCCATTGTAGAGAATTCATTGGAATTTTGGCTGCCTTTTGTCCCTGCAAATGGATATCATATTCGAACATTTCATTTTTATTGTACAACATCTTTCACATCTTTTACATCAGTTGCATGATACGTTGATACTCAATTTGGCCGAGTTCGCTTTTTACTAGTCTGAAAATGTGCTGGTGATTTTTCACACTGATTGATAGGGATTGATTTTTCCAACCTATTAACTCTTTCCTCCTAGTTGGTTCAACTGTTAAAATTTGAGGGTGCTAGTTGCTAGTTGGTTCAATTAGTGAAATAGTAAATGAGGTTTGAAAATTCATATTTTGCTTGCATTATGATTGAGGGATGGGTTACATCTATGTAATGATTATTTAAAAAATAAAAAGTAACCCATCAACCTATCAAATATATGACACTCAAGTTCAACCAACAATAAAAAGTTGTGCACTTATATTATAGCCCTGTTTCATATTATTTATTGTATACTAGTTATGTCGCTTAAAATTATGATTTGGAAATTAAAATGATTTTAGTTCGTAGATGTTTGGTGGAAATACACATTTATTAACACTTAACAACCTTAAAAACTCAATTCAATCATATTTATCAAACATCAATTT

>CsHDMA3

ATGGATGGTGATGATTCGGTACAACACTTGCAATTTAACTCAGGCACTAATGTTAAGCTAAGGTCAAGAAAGAGTGCATCAGAGAGTTTTTTAAAACCTAGGGTCAAGAAAATGGATGGTGAAGAGAAGAAATGTGAGTCCAAGAAAAGGTCGAAACCAGTGGAGGTTGGTTTTGATTCTGATGATGATGAGCCTATTGGGTCTTTGTTTAAGTTGAAGAAACAGAGAAACCCTAAGAAGGCTAAGGGCCAGAAGATTGAAGCTAGGGATGATAAAGTGACGGTTGAAGATGATGATTTGGTGGGGGGTATGGATGATACATTGGCAAGTTTTAGGAAGAAACTGAAGGGTCCTAAGAAAGATGCTGGATCTGGAGTCTTAAATGGAAGGGGTTCAGCTCTGAATGGGTCTTTAGATGATGACTGGGTTTTGGATGTGAAATTAGCGCCAAAGCATGATGAGAAAGTTGGGGTTAGTTGTGAGGATGGATCTGGTGTGACTCTGGATAAATGGGTTGAAACTAAATGCAAAGAAAGGGTTAAGAGATCCAAAATTGATTCAAAGATGACAATTATTGGGAATCATGTTGTGTGCGATGATGACTCAAAATGTTTGTGTTGTCGGGGCGATTCATTGGAAGATCAGAAAGAGGAAGAGTTGTCGACCTTTTTTCAGAGGACACCATCTGGATTACTCAGGAAATCTCGAACCAATTCTGGTTCGAAACAGAATATTAAGGAATGGAGCTTGCGTGATGGATCTATTCCAAGTTCTGAGGGTGATTCAAAGTCTTTAATGAGATCTCAGTCTGTTTCTGCTTCAAAACTGTCTAGAAAAGATCCAAAATCTGATGATAATTCAAATACTGTGTCCAATTTGAGGACATTGGAATTAGACTCTGACCAATGTAAGAAAGTCGGACCAATGTTAGAGACTTATCACTCAAATGTTCAAGATCCTTGCAGCTCAAACAAAGTTTGTGATAGTGATGGAAAAGCACATACTTGTCTTCCAGTTGGCCATGCATCTGCTTCTGGCCAAAAGGCTAGATCAGATACTCAAACTTTGGATGAGTTAAAGCTTTCTTCTATGGAAAAGGCAAGCACTTTGATACTTGATGTTGTTGAAGTGCCTGATCCTGCTTCTTGCTCAAAGGCGATGGAAGAATTTCATGAGTTTGACGGTGAATCTGACAGAGGTTTTACAGATGCACTGGACCTACAGTCTAATAGTATTTCAGCCATGAACATTTCTAGTCCAGACCCAGAAATTTCTTCCTCTTCGACTGGAAAAGAAGTCTCATTGCCTTGTGCTGAAGATGAGTTAGCAAGCAAATCTTGTAAAACTGCATCCAAACAGATTCATGTTTCGGCTTCTGAAAAAATTCTGCAGGCAACCTCAAAGCTTTTAACTCAGAAGTCCTTGGGAGCTGAAAAATCAGAAAGTTGGTTCAATTTTGATCAATGCCCTGCAGGTTCGGAACAAATTCCACTCTCTTTGACTAACCCTTCCTCTACTTTCTTGGAGATGGCAAAAACATCAAGGGATGATCCCGTTACCTGTACTGGAGAGCCGTGTTGTGCTGCAGATTCTTCTAACAAAGAAAATGCTATACCATCTGATGGTAAGTTATCTCCTATGACTAAACTGTCTTCTGAACTCCAGGAATCTGGATAAAATTTTCATAAGTGCAGCTCGGTTTTTAATAATAATCAAACTTCTGATGCATGTAAAGAGACTTGTGTTCCAGGTCATGCTGCTTTTTCCACAGACGAATATGCTAATGGAGGTTCTCCTTCCTCTGTAGCTCCAGATGAAAATGGAAGTTTTACAGAAGATACACTATCCATGCCTGATTATGAAAACAGAGATACTAAGCTATCAGCAGTCCAACGTGCTGTGCGCAATGCTAAAAAGCGTAGGCTTGGAGACATGGCTTATGAAGGGGATGCTGACTGGGAGGTTCTGATAAATGAGCAAGGGTTTCTTGAAAATCATCAGGTTATGGATTATGAACAAGCTCTGAGAACAAGAGACAAGTTTGATTCGTCTTCAACTACTCTAACAGAAGCTGAAAATGCTGGGGCTGCAGCGGTAGCAGTGGGCCTGAAAGCTCGTGCAGCCGGTCCAATTGAGAGAATTAAATTTAAGGAGATCTTGAAGCGCAGAGGTGGGCTTCAGGAGTATTTGGAATGCAGGTTAGCTGTTGTTCCCAAAACCTGCTTTAAATTCATGTACTTTACAAAGCTTAAGGGACTTGTTGCTTTACATAATACAGGAATCAGATCCTAAGTCTTTGGAGTGGAGATGTTGGCCGTATTTTACCACTTACAGAGTGCGGAGTTAGTGATACTCCATTGGGGGATGAGCCTTCACGTGCTTCCCTAATTAGGGAGATTTACAAGTTTCTTGATCAGAGTGTAAGTATGTAATACCCAGTGAGTGTTTTCTTTTCTATTAAAATTCACTTTTGCTTGCTTTTTTCTCATAAAAAATTGATTGTTGTATTTGTTTATTTTTATGCTTATCAGCTTTTTAGATATGCATTTTTGCAAAAAATTATTACTTTGGAGGATTGATGAATTGTCCTATCCTGGTCAAATTTAAATCTAACCACATTTTGACAGGGTTATATAAATGTTGGTATTGCTTCTACGAAGGAGAAGGCAGATCATAACGCTAAGCATAGTTATAAACTACTGAAAGAAGAAAGACTTGAGAAAAGTTCTGGGGCTTCAATTGCTGATTCAGAGGATGGAGTTGCCTTCATCCTTGGCCAGATTAAAAGTTCTGAAACTACTACAGAGGCTAAACATGGTGTTGAGTGTAATGATGGAAACCAGCAAATAGGAATCAAAACTGGAGGTTCAATGACACCCGAATTACCTAATGAGATAAGACAGAAAGAAAGTGTAGTTGATGATTGCCAGCAAAGAGTCGACAGTGATCCAAAAGCATCCAACAGATTGGTTGGTGTAGATGTTTCGTGTGATGACCCATCTTGTGGAATGGTTGATGGTGGAACAGTCCCTCTCACAATTGAAGAGAGGAGTGAGTCAGAGAGAGTGCAATCTGCTTCTTGTGATGATGCCGGAGAGAATCATTATTTGCGTTGTGATATAGATGTCAAGAAGAGAATCATTGTCATTGGTGCTGGTCCTGCTGGATTAACTGCCGCTCGGCACTTACAACGTCAAGGTTTTTCTGTAACTGTTCTTGAGGCTAGGAACAGGATAGGTGGTCGTGTTTATACGGATCGCACATCTCTTTCAGTTCCTGTGGATCTTGGGGCTAGCATTATTACAGGTGTAGAGGCTGATGTGGCCACTGAAAGAAGAGCTGATCCTTCCTCATTGGTTTGTGCTCAGTTGGGCCTAGAGTTGACTGTGTTAAACAGTGACTGTCCTCTTTATGATATTGTTTCTGGTCAAAAGGTTCCTGCAAATGTGGATGAAGCCCTGGAAGCGGAGTTCAACAGCCTTCTTGATGATATGGTGTTGCTTGTTGCGCAGAAGGGGGAACATGCAATGAAAATGTCTCTTGAGGATGGTTTGGAGTATGCCCTTAAGAGGCGTCGAATGGCTCGGTTAGGAAGAGGTCGTGAGGATGCCTCAATGCACAATTCAATGGATGTTTATTCTAAAACGAGCAGTGTTGACAGCAGAGTTCCTGATAAAGATTGTTCTAGAGAGGATATCTTGAGTCCTGTTGAGAGAAGGGTTATGGATTGGCATTTTGCCAATCTGGAGTATGGCTGTGCTGCTTTGCTCAAGGAAGTATCCCTTCCCTTCTGGAATCAAGATGATGTCTATGGAGGATTCGGAGGAGCTCATTGTATGATTAAAGGTGGTTACAGCACTGTTGTTGAGGCTTTGGGAAAAGAACTTCTCATTCATCACAACCATGTAGTCACAGATATTTCATATAGCTTCAAGGACTCTGACTTGAGTGATGGTCAGTCCAGAGTCAAAGTTTCTACATCAAATGGCAGTGAATTTTCTGGGGATGCTGTGCTTATTACTGTGCCGCTTGGATGCCTGAAAGCAGAAAGTATAATGTTTTCCCCGCCGTTACCACAATGGAAGTATTCTGCCATTCAGCGGCTTGGTTTTGGAGTACTTAATAAAGTTGTATTAGAATTTGCGGAAGTCTTTTGGGATGACACTGTGGACTACTTTGGTGCAACTGCAAAAGAAACAGACTTGAGGGGCCGGTGCTTTATGTTTTGGAATGTCAGAAAAACAGTTGGGGCTCCTGTTCTAATAGCCTTAGTGGTTGGTAAGGCTGCTGTTGATGGTCAAAATGTGAGCCCATCTGACCATGTAAACCATGCAGTGATGGTTCTTCGTCAAATTTTTGGGGCCGCTTCCGTACCTGATCCAGTTGCATCAGTAGTGACGGATTGGGGCAGGGATCCTTTCAGCTATGGTGCTTATTCTTATGTAGCCACAGGAGCATCTGGAGAAGACTATGATATATTGGGCCGGCCTGTTGAAAACTGTTTATTTTTTGCTGGTGAAGCCACATGCAAGGAGCATCCTGACACTGTTGGTGGTGCAATGTTAAGTGGGCTCCGAGAGGCAGTGCGTATAATTGACATATTGACAACTGGAAATGATTTTACAGCTGAAGTAGAGGCAATGGAGGCTGCACAGATGCAGTCAGAGTCTGAGGGGGATGAAGTTAGGGACATAACAAGGAGACTTGAAGCTGTTGAGCTTTCCAATGTCTTGTACAAGAATTCTTTGGATCGAGCTCTTATATTGACTAGGGAATCTTTACTACAGGACATGTTCTTTAATGCAAAAACCACTGCAGGACGTTTGCATTTAGCCAAGGAGTTGTTGAATCTCCCTGTTGCAACCTTGAAGTCATTTGCTGGGACAAGGGAAGGGCTTACCACCCTCAACTCATGGATACTGGTATTTTCATACTGATAATATACATCACCGTTCAAACATAATATGAAATTTTTTAATTAATCTGCATGTTCTTCTGCTCTTGGCCACATGCAGTGTTTTGTATGTGTATACTGAAAGCTTTCTGAGTGACTTTCCATTTATCACAGGACTCGATGGGGAAGGATGGGACTCAGCTTTTGCGTCATTGTGTCCGTCTTCTTGTGCGTGTTTCAACCGATCTACTTGCAGTGCGTTTGTCAGGTAAAATTTCCTCAGTTTTGGTTTGGTCTTATATATATGCATATACCTTGTATATCACAAGCACACATGCCTGCAGATAGTGTCTGTTTCAGAAACAAAAGATTCATTTCATAAACTCTATTTTTGGCCATGTTATACGGAGAGACTAGTTTGAGAATTTCAAAGCTTTCTCAACAGTGCTATTCTCATTTTGAGGGAAGGTGAAAAAGTAAACGATCTAATTTATCAAGTCTGTGTTGGAACTTTCAAACTAGTGAACTTGTTGCCATAGTGGGATTGAGTAAATTTCACAACATTTCAGCACAAATAGAATTATTGTGAAAGTTTGATATAATTATAGATATCTATGCACTTATGTATGGTTTAATTTATGCATTGTTGCATGTATGCATCTTTATGTGGCATGTATGTGCTTGTTAATCCACTTATGTTTTTTAGTTGTAAAGCAATGATAGAAGCAATGTGTTTCATTGCGGTATCTAATTCAATTATTTTTCTTTGGCATAGGCATAGGGAAAACCGTGAGAGAAAAAGTTTGTGTACACACTAGCCGTGATATACGTGCCATTGCAAGTCAGTTGGTTAGTGTGTGGCTTGAAGTCTTCCGCAAGGAAAAAGCTTCCAGTCGATTAAAGTTATTAAAGCAATCAACTGCAGTAGATTCTATCAAGAGAAAGTCTCTTAAAGATCCATCTTCAGGGAAGCCGCCTCTACACTCACACCATGGTGGTTTGGAGAGTAAAGTATCTCCTGGAAGCCATTTGACTTCTAATGCAAACAATAAGAAAGAGAATGGCAAAACAATTAAGTTGGGTAGTGAGTTGGAAGACAAATGCTTCGCTATGTCTGAAGAAGAACAAGCTGCATTCGCTGCGGCAGAAGCTGCTCGAGCGGCAGCTGAAGCAGCTGCTCTAGCTGCTGCTGAAGCAAATGCTAAGGTTTGTTTCCTTTGCCACTACTGTAGTAAATCATAACTGCAGTGTCCCAATCTACCATCTATCATCTACCATATACTGTCATGTTTGAAACTTGCTTTTGGGTAATAGTCGGAATTTGGACTATCCAGTCAGTCATCTCATGTTGTTTTTTAAAACATATGATGCAATTATTTCAGCTGGCTAAAGGCATTTAATTGTGTACCGCTCTTTCCACTTCCTTTTTCTTTATTCTGCTGATACCGCTAGCTCATGCACTGTCCTATTGATAGTAGCATGTATTGCTGTTTACTTACCCGCAAAAGTGTAGGGCCAATGACAGTGCATTGATGTACCCTAATTGCCCATAGGCCATACTATTTTCTCCTTTTATTTTTCTGCTCTTATTGATTTGTTCACAACTATTGGCTTATAATTTGTAGGCATATGCTACCTCGGGCCCACAACTTCCCAAGATACTTTCGTTTAACAAATTTGCCAAACTGGGGCAATATGGGCAAATGGATGATTATGATCTTAGAAGGAAGTGGTCCGGTGGTGTTTTAGGAAGACAAGACTGTATATCAGAAATAGATTCCAGGAACTGCAGAGTGAGGGATTGGTCAGTTGATTTCTCTGCTGCTTGTGTTAACCTTGAGAGTTCAAGAATGTCTGCAGACAACCTTTCACAGCGAAGCTATTCCAATGAGATTGCATGTCATTTGAATTTCACTGAGCGCTCTGGAGAAAGTGCTGCTGTGGACAGTAGTATTTTAACAAAAGCATGGGTTGATACTGCTGGTAGTGAGGGTATAAAAGACTACCATGCAATTGAGAGATGGCAATCTCAAGCAGCAGCTGCTGATCCTGATTTCTACCATCCAGCAATTCGTATAAAGGATGAGGAAGATTCAAACACGAGTTCTAAGCCACATACCCAGAAGCATGATAGACGTGCAAATGAGAGCTCTGTTTCCCAAGTCACTGTAAACAAGGAGTCACTTAAAAGTCATCCTCGAGGAGCAGATCGTATAAAGAAGGCAGTTGTTAAGTACGTTGAAACATTGCTTATGCCCCTTTACAAGGCAAAGAAAATTGATAAGGAAGGATATAAATCGATAATGAAGAAGAGTGCGACAAAGGTTTGTCTTTTCCTCCCTTTTCCTCCCCACTTTTGGTTTTATTTCAGTACAAATTTGATGCTGATGGTCTCATAAACTTCTTCGATTTGTTTCAATGTCCTCAACTTTTTTATTTTTTCACAACCAGGTTATGGAGCAGGCCACAGATGCTGAGAAAGCTATGGCTGTTTCAGTATTTCTTGATTTTAAGCGTAGAAATAAGGTATGTAATCTTTGCATAGCTTTTAACTTTAATGTTATTTATTTATTTTAATTTTATCATTATGTCTTAACATGTAGTTCCAATGATACCTATTATTTTAAAGTTCAACCATAATAAAGGTTCCTTCTGGCTTGATCAACTTCAGTGGATCTGTTTATATGTTTGACTTGTAGAATAACACTTTTAATAGAAAATATATATGAGTTGTTGTCTTGAGTGTCTTTTGTTATTGTTCTTTTCTTACTTTTCCTAAAACCTTGTCAGGATTATAACTGCTACTTCCATGAAAGAAACACATCCAGTTCTGGAATTTTTTAATTTGTCGCACTATCTTCTCTCTTTTTTACTGGGGATTTCTTCTAGGCAAATAGTGCCTAAATGGTAATCTGCAAGCTCTGTATCTTGATCAAACTTTTATTTCTTGGATGCCTTTGTGCAGATTCGTTCCTTTGTAGACAAGTTAATTGAGAGGCACATGGCCGTTAAGCCAACTGTTAAATCTTGA

**CsJMJs:**

>CsJMJ1

CTTAACTCTTTTTCATATTTTTATTTCCGTTTTCTTTTTTTTTTTAAGTTTAAAATAAAATTTCATTTCTCTCTCTCTCTCTAGCAGATCCAACTGACTTCTTTAACGAGGAAGCCTGGTTTCACGCTTTTTTTTTAATTATTATAATTTTCTTATTTATTTTATGATTATTAATTACCAATTATCTTATCAAAACACCATTAAAAAGATAATTAATTATCTAAACTACACGTCTTTCAACATAAAATCTTACCCGTTCATGTCTCTCTCACCTCCTCCTTCCTTCCTTCTATTCACCCCCCCTTTTTTTCTTTAGGTATTTTCATTTTTAATTTATTATTCAGTCAATTTGTTAGACTAATTTAGGGTTTACTAGGTCAAATCCAACTCTGATTTCCAAATAAAAATTTAAGATTTGAAAGAATATTTTGCTCAGGGCAAATACTTTCACAATCAAATTGAGTCTTATTAATAAAATTAATTAATTTATCCCTAACTGGATTTTTATAATAAAAAGGTTGATAAAATAAATTGTTTTCGGAGTTTCCTGGAATTTAAGGGGCTCAATTTGCTTGCTTGACAAAAAAGTTTCTGGGAATTGGGTAGGTATTTTGATCAATTAGGTTTTTGGGAATATAAATTAAATTTTTTTAAATTTGGTTTCTTAAAATCTTGTTTTTAGCCATCAATGGAACAGTCCAAATTGGCCGCAGAATCTCATATTAAAGAGGTTAGTTCTTTAAATCATCTTTTCCAATTAAATTAATTTCATGCATTTTTTCTTATGTAGTTATGCAATCAGCTATCTGTGTTAGTTTCACGTGTTTAGGTATATCGTATGCTTAGCTTTTGCTAATGTTTGTTAATTTCATGTTATGAAATGCAGATCTTTTTTTTTTTTTTTTTAATATTGTATTATAGAAAGTTACAAGCTTTCCAGATTAATGATGTGCATTAAGGTATCTGGGTTACTATATTTGATCACAACTCATTCACATTCATTTTTACAACAACTTATTCAAATCTCATGTTTTTACTCTTCTGATCATGGTGTCTGTTTGGGCATACCATGTTTATGATCTGCTATTTCTTTGCAGCAAGAGAATTATTTATATCTTTTTGATTCGGGACAATGATTTGAACTAGGATGTAGATGGATCAAATAAATTTAGTGGGTAGGCTCTCAAATTTGTTGGTTGATTGGGTATTAGGTTTATTGAGTATTGCCGGTGTTTTAAAACTGTCCGTGGAACATTTCATGTCGAATGCTCTGGGCGCGTGAATATCCTGTTAGTTAATATATGCGGTACTAACATTTCTTGCCGAATGCATTCTTATATTAGCTATTGTTCAACCTCCATTAACTTGTTTAGGATGCCCATCTTTAACTAATTTATTTGGATTTATACATTGTCTTGCCATGCCTGAATGTCGATATTGTAGTTCTATTTCTTATTGATTATGTGCAATCATTTTGAAGCTTGCAATCATATTTTCCATTAATACAGACTAGAGCATGCTGTGTTCTCGAATTAGATTGGGATAATAAATCAAAAGTCCAACCATGTAGATTTCTTTAAAAAAGTTGTTTTGCAGGATCATCCTTTAAAGCACACTTCAAAAAATGATAAGACCACCAGTTACTCAGGCAGTCCTCAAAGAGGAAAGCTACATAAAAGTTATAATGATTTCCAATGTTAAGTTAGGGCATCTTATGGAGGCTATTGATGGGCTAGTACAGTGCTTTGGTTATGTCCTAGTTGTGGTACCAATTTTGATAAGCTCTATTTTTGTTGCAGCGCATTACAAATTTGGGACATCTTTGGATTTCCTAATCTTTTAACTCTGTCAACTGTTTATTGCATTGTATTTTAACACATAATCCCCTTATGACATAGCGTCTGTAGTCATTGAAAGCGCTTATTTCAAGAAATTGAGATGTAGTTGGAGCATGTTAGGCTCATCTGACTGCATAAAAACATTACCTGTACATTTAGAATGCAATTTGATACAGAACGGACTTGAAATATTTTAAATTCTCCCCTGCTTTCTTCTGCTAATCCATGATGCTTATATAATGGAGACTACTGTTGGTTTTTTCAGATTTCCGCTAGATGGGATCCAGCTGAAGCATGCAGACCTATAATTGATGAAGCTCCTGTCTTTTATCCAACTGTGGAGGTATTCTGTTGCAAAAGAACAGAGATCATATTTTGGAAATATTTCATAGCAATATCATTTTCACTATTAAATATTATGTGATTTTTCATTCGTCAGGAGTTTGAAGATACCCTTGGATACATAGCAAAGATACGCTCAAAAGCAGAATCGTTTGGGATATGTCGGATTGTTCCCCCATCTTCCTGGACTCCACCATGCCCTCTTAAAGCTAAAAATATATGGGAAAATGCCAAGTTTTCTACTCGAATCCAGCAAATTGACTTGCTGCAAAATAGGGAGCCCATGAGAAAGAAAATCAGAAGCCGAAAGCGGAAACGGAGGAGGCAGTCTAGAATGGGATCCACCAGGAGAAATGCTAATTCTAGTTCTGAAGCTAATGCTGCTGAGACTGATGAGAAGTTTGGATTTCAATCAGGACCAGACTTAACACTTGAGGGTTTTCAGAAATATGCTCAAAATTTTAAGGAATGTTACTTTGGGATGAACGATTCTAAGGAGGATGTAAAATCTGATGGGTTTGAACATAAAAGATTGGAACCCTCTGTGGTGGATATCGAAGGTGAATACTGGAGGATAATTGAGCGACCAACAGATGAAGTTGAGGTATGAACTATGGTTCTCTAACTGTTTTTTCTTTCCTTTTTTTTTTTTTTTCTTTTTTCCGGTGGGCAAATTTGTTTTTCCATGTTGCAGGTATACTATGGGGCTGATCTGGAAACAGGAGCATTTGCAAGTGGATTTCCCAAGGCATCATCCTTGGGTACTGAAAGTGATTTGGATCAGTATGCAATGTCAGGCTGGAACCTTAATAACTTACCGCGCTTGCCAGGTTCTGTTTTAGCCTTTGAAGGAAGCGATATCTCAGGAGTTTTGGTGCCATGGCTCTATGTGGGAATGTGTTTCTCATCATTTTGTTGGGTAAGTAACTGGAATACTGCTTTCAAAGTTTTATCGATAATTGGTATAAAATGTTAGTCATATGTGCCACTAAGATCCTTGTCTCTGTATGCCTAATCAGAAGGTACAATATTCTCTGTCTTTGCCGTTGTCCCTTAGAAGCTCTAGATAGGAACCACCATAAGTTATTATAATTTGAATTTTGACCTAAAATGGGTTGCAAAATTTTAACTTTTCTATTTGTTTTAATCCAACACAGTTTAAGAATTGGAGTTAATTAAAGTCTAAAAGGTAATATTTATTATTACCAACTCAGAAACTCTCTAAACCAATGAATTAGAACAGTTAGATGGTTGGGAATTTGTAAGTTTTTCGTTCTAATGTATTATCTTTAAACTAGTCATGTTAAACATTTTATGATGCCATGTCATATTCTGGGTGTGTTGAATAGTTTCAAGAAATACCGATGTCGATTGATCTGGAATACGTTATGATCATAACATTTGAAGTCAAATAGTTATTTATTTTTTAATTGCAAGAACAATTAATTAACTACATGTTTTCACTTTCTTTTTCCCGTGATGATTTGGAACAGCATGTTGAGGACCACCACTTATATTCGCTGAACTATCTGCACTGGGGTGATCCAAAAATATGGTATGGAGTTCCTGGAAGCCACGCTTCTACTTTGGAGAAAGCAATGCGGAAGCATCTACCAGATTTGTTCGAGGAACAACCTGATTTATTGCATGAACTTGTAAGTGTTTCTTTCTTCTTTCTTTTTATTCTTAATTTTTTTTTAAATTGAAAAAATTAATGCTTCTTTGATTGTGCTAGACAACGCTTGTAAAAATGCTTTCTTAATTACATACCCGTCCTTTAAATAATTATTTACATAGTGCGTGATACTTATTCTGTATGTTTGTATTTGTATTTCTGCAATTTCTTAAGAAATTGAAAAAAGCTTGAAGGCCTCAGCATAGATTTAAAGTGAATGAATATTGTTCAACACTTTGCAAAAAGGGACTTCAAAATTTGAAGAGGCTGTTCATCTTGCCAAAGCTGGCTCATTATCTAAAAGTTAAGGAATAAATAACCGGTTCCATAGATCATTTCTATTAGATTCCAAGGCTATTTCTCTATTCATTTTAGCTTTTCTCTTGTTGTTTTCCCCAGCTGTCTTTTACTAGTTGATTGTTCTGAATTCATGTTACATTGTTCAGCTTTTGCCACCCTCTTGGACATTTCCTTCAGATTTCACTGAGGCATTAGAAATTCTGGCCTTTCTATTTTGGCCTCAGTCATTTTTTTCGTCTATGATCATATTTGAGGTGTAAAGTTTATAATGTTTCTCGTGGAACAGGTCACTCAACTATCGCCTTCAGTTCTTAAGGCAGAGGGCGTACCAGTATATCATGTTGTTCAGCATTCTGGGGAGTTCGTTCTAACCTTTCCAAGGGCATACCACTCTGGATTCAATTGTGGATTTAATTGTGCAGAGGCAGTGAATGTGGCCCCTGTTGATTGGCTAGCACATGGTCAACAAGCAGTGGAGCTCTACAGTGAGCAGCATCGGAAGACATCTCTTTCCCATGACAAGCTGCTGTTTGGATCAGTCCAGGCAGCTATTAAAGCCCTTTGGGAACTATCCGTGCTTCAGAAGAAAACTCCAGGAAATCGGAAGTGGAAAGATGCATGTGGGAAGGATGGAGTGCTTACCAAGGCAATTAAGGTTAACCTCTCTCTCTCACAACACTTGCACACAGACACAGTAATATTGTGAAAGCAAGAGATTGACTTTTTTTTTCCCCCTCTTTGTCAAAATGCTTTCAGACAAGGGTACAGATGAAGAAGGAAGGACTACAGAAGCTCCCATCTTATTTTAAATTGCAAAAGATGGAAATAGATTTTGATTTGAAAACTGAGAGAGAATGCTTCTCATGCTTCTATGATTTACACCTGTCTGCTGCTGGCTGCAAGTGCTCCCCTGATCGATTTGCATGCCTTAAACATGCAAATATTTTTTGTTCATGTGAAATAGACCATAGATTTGTCATTCTCCGTTACAGCACGGATGAATTGAACACACTAGTTGAAGCTTTGGAGGGAGGATTAGATGCTTTAAAAGAATTGGCATCCAAAAACTTCAAATGGGCTGATTGCAGTGACACTGATGGTGGTCTAGTTAAGATGGATATGGAGAGTGAAGTATTCCCAATGGACTGTTGTGAACAGAAGGAAAGTTCATCTTCTTCCCCAAGAGTAGAAAATATCGTGGAGGGCAATGGCCCTTGCTGTTCACGTAGCCATGTCTCTTCAGAAGTAGTCCAGTCAGAGCCCCAGCGTGGAACATCTGGTTTAAGTGCATCTCATGTAAGTGTAAATAGCCATAATGAAGGTAATGATGAAACCCAAGTCATGAACAAAAAGGCTAAGGTGAAACATGAGGTTTGTATTGATTTAAATATGGATGTTATACCTGATGGTAATGAAAGCAAGTTGCTGCTATCTGATAGCCATGGTAAAGAAGCTATTGAAAATCTGAAGGCTCATTTGTCTGCGTGCTATCAAGAGAAAGTCCTCTGCTCAGGCACAGTGAAAGAACAGGACACAATGCAAGTACGTAGTGATTGTAATTCATCCAATTCTCATAAAGATCCAAACAAAGATCAACCTTCATGTTCAAGGGTTATTGAGGGTACTTGTTCATTTGACGTTAAAAAATTGTTTGGGGTTGATCTTTCGTTGCCACATCAACAGTCAAAACTTCCACTAGTTGACTTTTTAAAGACTGATACCATCAATGGTTCAAACGTGAGGACGAGTGTGACTGACCAGAGATTTCAAAAGAAGTTGGAAACTTGTGTTGAACCAATAAATTTTGGATGCGTCATGTGCGGAAAGTTGTGGTGCAGTAAGCAGGCCATATTTCCAAAAGGTATGCTGCAGTGGGTATTTCCTTTTAATTTGTTTATAATTCTTTTAGCTTCATGCCATTGTAGTCACAGCTTAGTGAATGATATTTTCTGCAATTTTTGTTCATCCTGCAGGATTTAGAAGTCGGGTTAACTTCTATAGTGTACTTAATCCAGAAAAGGTCTGCAACTATATTTCGGAAGTCCTGGATGCAGGACTCCTTGGTCCTCTTTTTAAGGTGGAGTTTCTCTACTTAAATTGTCCTTAATGTACTAGAAATTAAATTGGGACCTGATATTTCATTGTTTGAAATGCACTATGCTTAATTGTTCTAGAAATCGCGGCTTAGGAAATTTGTTTCAGGTCCTATTCATTTCCCCATTCAATATTTATTTAAAAAGATCTTTGTTTATTGAGGTAAATGTTAGCAAAATTGGTTAATTTCACATTACTACATTTATAGCTTTTATCATGTTTAAACATTGTTTGCTCTATTTTGAGTATTCAAAACAAGCGCTCTTATTCATGCATAGGGATTTAAAACCACCTGCAAAACATGCTTCCACTGATCTATTTAATCATCTTTCATGATTACAGACAAGCTAAGCACCCTATCATTATTGAGTTTTCCAGCTTATTTCTTGTCTCGTCTCTAATATAGCAGAATCAATTTCAGGTTACATTAGAAGAATGCCCTAGTGAAACATTTGTGAATGTCTCCGCACAAAAGTGCTGGGAAATGGTGCTGCAAAGACTGAACCAAGAAATTGAAAGACAGGGTGGTTTACACGAAAGAGGACTGCCCCACCCCCAGTCTTTGCAAAGCATTGATGGCCTGGAAATGTTTGGGTTTCTATCGTCGCCCATTATTCAGGTAAAGCTTCTCCTGGTCCAAGTTGCATTATTTGATTATTCCTATAAATGAATATATTGATTCTCTTTCTATGTTTTCCCATGCATTTGCTAACATTTTTGTTCTGAATCCATTACTTGCAGGCTATTGAGGCTCTTGATCCAAATCATCTATGCATGGAATATTGGAATCACAAGCTTTTGACTTTTGGCAAAACTACTGAAGTAAATAAAAACTCATCATCTGGATTGAGTTGCTCTGAAGCGGAAACCAAATCAAAAATATTTGGTGTTGCCCTGATGGATGAAGACCAGAATAGTCCATCTGGCCAGAATTCTGTCGAAGAAGAGGCACAGCTAGTGTTACGGGGACTTTTCCAGAAGGCAAGTCCCAAAGAGTTAAAAGTAATGCAAAGAATCCTCTACAGCGAGGGACGGAGTGACGAATGGAGAGTGGCTTTGGCAACATTGATCGAGGAGATCCAGAAATCATGTAGATAAAATAAGAAGAGAAGTAGGTCCAATGATTCTTTCTCACTCAAGTCATTCATTAGGCCAATTCTTTTAGAAATCCGTAGAAGTGAAAGACGGCTGAAGGTCGCAAATTTTGCTCAAATAATTTTAGTTTGCCACTCATGAGGCAAATAGATTAACCGACTGCAAACATAGGGCTACATTGTAACTAACTTTTGATGAAATATAATTCGATTTTAGTATTTTTCGAGGAAGTTATTATTGGCCCTTCAGTTCTTTGCTGGCTTCATTTCTAGACAGACGCTAATGTCATTCAGCTTCC

>CsJMJ2

GATGGCATAATTAAGGCAATAAGGCAGTAAAAGGCAGGCTCCTGCCCCTTTATTAAGGTAACTTGATGCCTTGATTTTTTTTTAAAAAATAAAGAATAAATAAAAGAAAAGAGTAAAATTCACAAGGGAACAAAATTTCTTTTTTGTTTTAATTTTGTCTTGTTTAATTTATTTTTTTCCTGTAATTGTAGGTGTTACAGTTTCCAGAACTTTTTGAGATTTTTAACAAATCTTGGGTGCTGCAGATTCTGTTATTGCATAGACCTAGTAAAATTTTGTTCCAGATTTGCCCTTATCCGATTCTGTTGTGTAGTTGAATTTGGGATTTTCTGAATTCTAGATTATATACAACTAATTTTTTTTTCAGATGAAGAAATTCTAGGGTTTGTTCATTGATTTTTAATCTCATGATTTAGACAAAAGTGTTTCTCGTTTATCATAAGTTGATTTGTGGGGTACCCTTTTGAATCCATTTTTTTTTTTTGGGTAGAAAAGTAAACTTTTTTTTTCTCAAATTTCTCAACAGGGCATCTGGGTTTAGTGTTACGGTTAAGGTTAGATCTGATAATAATCAGATTCATTATTCTTCTTTTTTTGGATTATTTTTTTTCTTTCTATTATTGGGTTTTGGATTTGGATTTCATTTTTTTCCTCAACTGTTGCTGAATTTGTTAATTTACACATAATCTGGGTGTTTATATACATACTGGATTTTCAGCTGCTTTCAGTAAACTGAAGTTACAGAGACTTGGATCAGGACCCCGTGAGCTCATAATTTCGATTTGTTGTTAATTTTACAATTTCTGAAAATTTTGCCTTGATTTGGAGTTGTTTTGCCGATGAATGTATATTGTGTGGATTTATTTGTTTAAACATAATTGGATATTTGGCTATTCTTATTTCATGGGATTAATTATGATGTGTGCAGGATGCGTGATGAGTAATTTGATGCATGTTTTATGCGAATCTTAGTAATTTTGGGGAAAAATTGAGTGCGCTTGAGGTCTTTCTACCGGAAATTAATGGAAATTTTAAGATCATGAATATAATGTACATTGTCGATCTTGGAAGACGAACTAATCTGGTATCTTCACTTAATTTTTAATTTAACAATAAAAAGAAAATACTAATAGATATTCCTAGAAGAAAGGTGAAAGCTTGAACTTACCCATAAAAAATAAAAAAAGGAAAGTAGGAAAATTTTGTTGATTCAAATTTGTGAATTTTTATTGCTTAACCACATTGATATCACTTTTAAATAATTTTCATGTGATGACTATATAATAGTTTAGCACAACTGAAAAATAGAATTACATGATAACTTGGGATTTGATGGTAGATGATGTACGATCTTTGCAAATACTACAGTAATTTAGACTGCATTTCTTTTGGTCTTCAGTACATCTTTAATCTAGACAATGCACTAGGAATAGCACTTTCACGTGAAGTTGTTCTGTTGCTACTTTGTTATATTGTCAAGCTTATTTTGTAATAGTGATGATTGTTGTAGTCTCTAGGCTAAATAGATGTTGGTTTGGGTAGATGATGGCTGAAGTTTTTTAATGTGTGTGTCATCGTTATCTTATTGCAAGCTTTTGTAGGCACTTTGTGAAGATACTGGATGTTGGATTAGCCACTTTTCGTAATTTTCATTGTAGCATGCAGGATTTCTAAGCGCTAAAAGCCATTTTTTCTGTGTTGTCAACTTAAATAGTTTGTTTCTTGCTACAGAAAAGAATTTGGATAACAATATAATCTGTTGTCTTTGTGTTGCAGTACTGATGGGTACGGAGCTCATGAGAGTTTGCATTAAAGAAGAGAATGACGAAGTTCCATCAGTTCCGCCGGGTTTTGAATCATTTGCATCTTTCACCTTAAAGCGAGTACAAGATACTGAGAAACATGACTGTGATATCACGAGTTGCTCAGCCTCTGCAAGTGCTTCCGAATCACTTTCAGTTCATATGGAAACTGAGGTTAAGGTTGCTGATGCGGCAAAGGCTGCAAGGCCCCTCCGGCGTAGACCAGGGATAAATTATGGACTACTTGACCACAGTTCAGAGGATGAGTCTGATTCTGGAAAGCTTGGTCAAGTCAGTGTCATTTAGACCCTCTTTCAAGTTATTGACTTCTGGAATTCAATAATATTTTGTTGGAAGTGCTATGTGTTCTGATATTGTTAATTGCTGCTGGTTATTTCAGAATTTTACTGCAAGGCCTTGTCTCCCCAAGGGTGTTATCCGTGGATGTCCAACATGTAGTGATTGCCAAAAGGTTATTTCTTAAACCTTATTTGTAACTCTTACAAAAGATTCATGCATTCCGACTATATCAATAGGTTCCAGTAAGATATGTTATTCGTTTCCATCTTACTTTCATCAAAGTGATCAGCCTATTGAAGTTGCTTTTCAGTATGCAAATGAATGCAATGATTTTGAAAGTACTCCATTATAATTAGTGAGACATCATAGGAGGTGGCTTTAAGGCATTGCTGATTGACTGCTGACTGTGAGGTTACTAAAGATCTGGACCCTAGGCAATCTCGACTTGTACTTTGATGCCTAGTTCCTTTTCTTCGTCTTCCAGCAGTCTGTAATTGATATGTAAATTTTTGGAGCAGGTCACAGCAAGATGGCATCCAGAAGATTCCTGCAGGCCTGACCTTGAGGGTGCTCCTGTGTTCTACCCTACTGAAGAGGTCTCTGTTGTTACTCTTACTATCGAGTTGTTTCAATTAAAAGATCTAGCTGTTGTAAAACCTGGTGATATAAATCAAATTTTTTTCAATTTGTTTTGTTATTTTCCAGGAGTTTCAAGATACGCTGAAATATATTGCTAGTATTCGGCCAAAAGCTGAACCATATGGAATCTGTCGGATTGTTCCTCCCTCTTCTTGGAAACCCCCTTGTCCTCTCAAAGAGAAACCTATATGGGACAGTTCTACATTTGTTACGCGTGTTCAAAGGGTTGACAAACTTCAGAATCGGAATTCAATGAGAAAGGTCTCAAGGATCCATAACCATTCAAGGAGGAAAAGGCGTAGAAGCACAAGAATGGCAGTAGACTGTGGAAGTGATAGTGGAAATGTCTCAGCTTCTGGTGATGTTGGATGCTACGAGGATGAGAGATTTGGCTTTGAGCCTGGTCCAGCGTTTACTCTGAATACATTTCAGAAATATGCAGACTTATTCAAGGCCCAATACTTCAGCAGGGATAAGAATGATGCTAAAGGCTTGGGTGCTAACACAGCTGTGCTTGAAGAGCACTGGGAGCCACTAGTGGAAAATATTGAGGGGGAGTATTGGCGAATAGTAGAGAAGGCAACTGAAGAAATAGAGGTATGATCTATGAGTTGATAAATTTCTCTGCTTTAAAATGTTGCAAAGACACCTGGTGCCTTTAACTGTCTTGTTGGCTCTCTGTTGTAGGTGCTCTATGGGGCTGACCTTGAAACTTGTGTATTTGGTAGTGGGTTTCCTAAAACGCTTAACCAGGTTGGCTCTGCTTCAGATGAGCGGTACATTAAATCAGGCTGGAACTTGAATAACTTTCCACGGCTTCCTGGATCTGTCCTCTCTTATGAAAGTGGTGATATATCTGGTGTTCTGGTGCCATGGCTGTATATAGGGATGTGCTTTTCCTCCTTTTGTTGGGTAAGGGTTAATTACTTCCAATGGTGTTCCCATCATTAGGGCACCATAAAAGGATTGAGAAACAAGCATGGTTTTTGAAAGAGTTAGAGAGAATAATGCGGGTTGTTGGAATTCTTTCTGGCTATATGATAGAAGAAAACCCATCTGCTTGTCAACTTTCTGAATCAGTGCTCTATGTCATTAGATTTAACTTCAAAAGATGCATTTGTTTAGAAATTATCAGTTAAACAATAAATAAAGTTTGGTTTCTTTTTTTTTTTATTTAGAAAGAAAAAGCAAATGTATGTTATAGCATAAAACTTTCCAACTTTCTCACTTTAGATTAATGCATCCAAAAACCATATAATTGCATTAAAACTTAACTTATGTATCTTTTAGAATCAGTGATTCAAGGTTGCAGCTTGAAGCAATTTCTATTATCTTGTCATTGTTTTCTGATTTATTAGTTGTACCTCTTGTTTTACATGTGGTATAACAGCATGTGGAAGATCACCACTTGTACTCACTCAACTACATGCACTGGGGCGCTCCAAAAATGTGGTATGGTGTCCCAGGGAAAGATGCTCTTAAATTGGAGGAGGCCATGAGAAAGCATTTGCACGACCTTTTTGAAGAACAACCCGATCTGCTTCATAAGCTGGTGAGTAAAACTTAACTCAGTATTCGGAATTCGTAAACTTGTGCCTTGGGGCTAACGTAATCAGCTGTTTGATATACCTCAGTTAATGCTATCAACTCTGGCAGTGATTTTTTTGGCTAGAAGCCTGCACACAGTCACAGTTGTTTATTTCAGTTCTTAGAGAGTAGAAAGCCTAGTCCTACTTTTTGCAAATTCTTACTAGCAATTTTTTATGAAGTTTGTAAAGTTCCATTAGGTTGAGGGCAAAGAAATCCAGGACAACCTGATCATGTAGCCGGGGGGCCAGGATAGTACATTTTAGATTACAGGCAGTATAGGAGATCATGGATAAGCATGATTATCAGGGAATTGCTTACTTTAATATATTCCCTTCGCATAACAATCACAACCTGGACTTCTTGGTCTTTCATGTAGGTCACACAGCTGTCCCCCTCCATACTAAAATCTGAAGGACTACCTGTCTATCGGTGTGTTCAGAATGCTGGAGAATTTGTTCTGACCTTCCCTCGGGCATATCATTCAGGGTTCAACTGTGGCTTCAACTGTGCTGAGGCAGTTAATGTAGCTCCTGTTGACTGGTTGCCCCATGGACAGATTGCTATAGAATTGTACCGTGAGCAGGGAAGGAAAACTTCCATTTCCCATGATAAACTGTTGCTTGGTGCAGCAAGGGAAGCTGTAAGAGCTCATTGGGAGCTTAATTTACTGAAGAAGAATACTTCAGATAACTTAAGGTGGAAAGATTTCTGTGGGAAGGATGGTATCTTGGCAAAAGCACTTAAGGTAAGCTTTGTCTCTCCTTCTTTTCTTGATTTGTTCTTGGCTTTTGTCGTGATTTTTAAAAAAAATGAACTTAATTTATATAGCATGGGAATTAGGTAAAATTCAGGATCTAGTCACAGTATGCCAATGCTAAAAATAGCATTGGGTTAGGTAACAAGCAGTATAAGCAAAAGTGAAGAGACCTGTGTCAAAATTTTGAAATCATGTATTATTATTCTCAATTTGGAATGGATTTGTTGACCCCCCGTATTAATACTCACTGCATAATCTGCTGCAAATGTTGCTCAAATTTAAAAATCTCTGATGACTTTTGGATCAATACACTTTCAATGGTTGAGATTAAATCTGACAGGTGTAATTAGTGCAATGCAATTAACGCACACTTGAGATTTATTCTAAGCCATTATAAGTACATTAATCCAAGGTTCTAAAAACTCTTTAAGAACTTGTGTAAAAAAAGTGGTGCCATACAACCTGCCCCTTTATTGTTTCTATGTACAATAGTTCTCTCATCCGGTCTCCCGGATTTAAGTGAAAATGCAGACTATAAATTCTGATCATCAAATGAAGCAACTTTTAAGATTCATTACAAGATTATTATTAAAGTAATATATTTGTAAGCTAAAAGAAAGTGGGCAGCACATACCGTCAGCTCAATTAAGGAAGGAGGATTAATGCTATCAACAAACATCTTAATCATCCATTATACATATGTTTCTTCCTCTCCCAAAAAAGTGGTGATCATAAAACCTGCTTCTTTATATATGTACCCAATAGTTCTCTCGTCCAGTCTCTCGGATTTAAGTAAAAATGCAGCCCATCGTATGAAAGCAACAGTCGAGAATCATCACAAGTTTATTATTAAGTAAAATATTATAATGCTAAAAGAAAGAGGACAGCTCTTATTCTTAAAGGTGCCCATTTTAACTCAAGGTGCATGAATGCTCTCAGTCCTAAAACCATTTTCTTCCTGATTTCATCCTCTTATCTCTTCCTTTCTGAGAAGCTTTAAACCTTCAGTAAGTACTAGTTTCCTTCCATTTTAAGGCCTCTTTTCACTTGGATACTACACAATGACAGCAAAAGGGGCCTTTTAAATAAACTGGCCTTCTCTTATTTTGTTCTGTTGTTTTCCTTTACCAGAAACTGGTAAACTCAAAAAATATTGCTCTAAAGGCTGCTGTTAGTTTTAGTTTACAATATTCAAGAATGTCTTCTCTTAGCTAGGGAACCTTCAAGAAGTTTAAGTTTGCACAAAACAAGGAGACATGAGATGGTAGCCATGAAAGTTTTAGAAAGATGTACTACTCTTTGGCACAAAAATGATTGATTGTTGATCTTTGTTGTAGCTACGCTTCTTGAGATTGTACATCTCATGTGGTTCATTCTGCCAATCTGATGCCTTTCAAAGTTGCTTCATCTCAATATCTAGTGACTAACCATAAAAAGCAAATGCAATAACATGTTATTGCTATTATTTTATTTCAGATCATTCTGCTCAAGATAATTTGGCAATTTATGATAAGAATTTATAAGAAATGTATATAAAACTAGTGTTTACCAGCCCCATTAATGATCTTTCCTTGATTGAGTGGATGTGCATCTTTGGACAAGAGCGCTGCCTGTCTTATTTTAGCTAATAAATATATTTTTAATAATTTCTGTTGGTTCTTGATTGATGCTTTCATCAATTTTCAGCATATTTAGTCCACAATTTCACTTAAATTCGGGAGACTGGATGAGAGAGCGTACTGTTTGTGAGCGTGTATGTAGTAAGGATGGCATAGCGATAGCCATTGGGCTGACTCTTTGGCTTGCAATTCACTGCCTATTGTGTAATATAAAGAGACAAAGTTTCTCCACTACCAGCAAGCTTTATTACACGGAGGACAATGAATTGTATATGTATACTGCTGTAAATGTGCATGATTGTTTTGGCATTCTTACTTATCTTTTTAATTGCTTTATTTTTCATTTGATTTTCACATGAAAACCCCTGAATGTTATTTTTTATTTTCTATAGTACGTCCAGCCCAATACCTAATTGATTCGATCAATCCTAGGACAAATTAATAGTGAAAGAAGTAAAAAACTAACAATTACAACAACACAAACACAAAAGTTAACTCTAGACAGTTTAATTCTGCAAGTTCCATCATTATGTTGGCAATGAAGCATGCATTTGAATTACAAATGCTTTGATTATGTTTCCCTCCTAACTTTATTTATTTCTGTTACCTAGAAACGTGTTGACATGGAGCGTGCAAGAAGGGAATTTCTTTCCAGTTCCTCGCAAACAATGAAAATGGAGAGCAATTTTGATGCTACTAGCGAGAGGGAATGCAGTGTATGCCTTTTTGATTTGCACCTGTCTGCAGTGGGTTGTCATTGTTCCTCAGATAGATATGCATGTTTGATTCATGCAAAGAATTTCTGCTCATGTGCTTGGGGGTCCAAATTTTTCCTTTATCGCTATGACACCAGTGAACTGAACATCCTTGTTGAGGCATTGGAAGGAAAACTAAGTGCAGTGTACAGATGGGCAAGACTCGATCTTGGGCTGGCCTTGAGTTCTTTCATCTCCAGAGACAATATGGATTTTGATAAGCTATCTCATTCCATGGATGGCCCAGTATTTAAAAATGTGAAATCACAGCCCTTGGACATCCCTGTGAATTCGACAGGTATCTTTAGTGAAACCTCTTTCCAACAGAAAAGAAATCCAGCAGAAGCTTTTTTGCCTTTAAAAGACATGAAAGCATCATCTACATCTCATAGTTCTTCTCCAGAAAGTGAAATAAAAAATTATGACCTCAAGTTGAAGACAGAACAGCCTGCCCGTCTACCTTCCAATTTAAAATTCCCAGCTGGTCTGCTCTCTCAAAAAGACAGGTCATACAGCGCACGCCCAGCTGAGGAGAAATGCACACTCAAGAAGCCTTCAGTTTTGGCAAATGACAATGTCATACTTCTTAGCGATGATGAAGGTGATAAACCTGAAAAGCCTTTTTCAAAAAGAGCAACAGATGGTTCTGTAAAACATTCAGAACCTTCTGAGAGGGGTGCTCATTCTGGAGATAAAGCCAATGGTAAAGATCCAACTATGTTTACCCCTAAGATTGAAGCAGGAATGTTGAGTCATAAGGATTTGAGCTCTTCGCCTGATTTACAGAGGAGTAATTGTCTATCCTATTCTATGCAATTAAAAGATACACGTCATCCAGATGGTGGGATAGTGTTAGGGTTACCAAACTTTACTCGCCATGTAGGTTCCACCAGCAAAAAATCTGGTGGAATTGTTTCAAATTCTTCAATTAGTAAAGAGCCCAGCAATCATAAAATGGCAAATGTTGAGACTAACCTTCAGCATCTGCCACCATGTGACACAGAAAAACCTAATAATGAGGTTAATCTTGAAAAAATGGGACCAGCTTCTACCTTAAGCTCGGATGGGAATGTGAGAGCTAATGCTGGAAATTCAACTTGCTCTCAAAATAATTTGGATAAATATTTCCGCCAGAAAGGTCCTCGCATTGCAAAGGTTGTACGGAGGATCAACTGCAGTGTTGAACCCCTTGAATATGGAGTTGTGCTTTCTGGAAAGTTATGGTGTAACAGCCGGTCAATTTTTCCCAAAGGTCTGTTCTATATGGAATTCGTAGAGATCATTAATCCTGATAAAAATATTTATGCAAAGAAAGCATGTTATATGTACTCATGCATACTTTATTTTACTACGCAGGATATAGGAGCCGTGTTAGGTACATAAGTGTTTTGGATCCAACAAGCATGTGTTATTATGTATCTGAAATTCTGGATGCAGGACTGGATGGGCCTCTTTTTATGGTAATTAGCTTTATCATTTTGTTCCTAAAACTGCTACTATAGTGTCGTGCTATCAGCTTAACAAGACATTTTTGTGTTTACCACTTAAATGCTTCACTGAAAGAAATTTATTGAAAGAGGTCATTGTTATTTCTTACTTGTACAATAGGTCTTGAATAGTGATTATTTTTCAATTTTAACTTTTCCATCCTCGTTATATCCAGTAGCTGCTAAATTGTTTTGCACAGTTCATATACAAATAAATGCTGAGGAGACACTGAATAAATAATTTATAGCATATCTAACTGCTTAACAAATAATGGGCATGGTCAAAGTCTTGCTTGCTGACACAGCTATCATGCAGGTTATGCTGCCTGTATTGTGTATGCAAGATAATTAATAGTCATAATTGGGAATTTTCAACTTCTGATGCAACTCTCTGGTCACAAATGATTCAGTGTTGTGCAAATTACATTATATCATCAAGTGGCCCTTTAGATTAATGTTGTGTTGAGAAATGTTCAGTCTAGGATAGTTTGAAGTTAAGGGAATAAATTTAACAGTGGGCTGTTTTCTTTTCTTTTGTCTTTGTTTGGTAGGCAGTTGATTTGAAAGGCTTGCGAAAAAAGGTTCAATAAACTATTGTGAACCATTGAATTGTTTTTTGTTGAGATGGCATGACATACAGATTCATTTAATTTTCAGGTTTCATTGGAGCATTGTCCGAGTGAGGTATTTATTCATGTTTCAGCGGCAAAATGTTGGGAAATGGTGAGAGAGAGAGTGAATCAAGAGATAACGAAACAACATAAATTGGGAAGAATGAACCTTCCCCCTCTACAACCTCCTGGGAGTCTTGATGGCTTTGAGATGTTCGGGTTTTCTACTCCAGCAATTGTACAGGTAAATATGCTAGTGGAACTGACGGAGCATTCGCAATCCTACTACTAATTACTACTACTCTGTTTAACATTGTTTTAGCTTTTTTTTTCTTTCCTTCTGGGGCAATTGCTGTTAGCTTTACTGTTATAACTCATCTGCTTTTCCATCTTCCTTGTCTTTCTCTCAAGCATTCTTCATGTTCGTTGATTTCATGGCTCATAATTTTTCTTAGTTTAGGACAAATGCATCATGTCAACTGTCAAATACAGAACAAAGTTCATTTTTCTTTTCTTTTCTTTTAATAGGCCATAGAGGCAATGGACAGAAATCGAGTTTGTACAGAGTATTGGGACTCCCGCCCCTACTCCCGCCCTCAGGTACAGATTCCACAACCTTTGCACTTTAAAGACAATGGGGCAAACTTGCGTGGTTTACCTGGGGAACAACATAATCAGGAGCCCCATAAGGGCAATCTCTTGCCTGGTGGAGTTGAATCAATACTAAAGGGCCTTTTTAAGAAGGCATCTCCAGCAGAATTACATGTGCTGTATAGCATCATTAATAATGATAAGCCAGCAACTGATCAAAGCCTACTGAGTCGACTACTTAATGAAGAGATCCACACTCATCCTACATGATTCATTGTCGGCCTCACAAGGGTTTGAGTCCGAACTGCCATGACAGGTTTGACTTCTGTAAATTCCTACGTTCTTGCTTGTCCTTGCAGAGGCACTTAGGCATGTATTTTATTCAACCCATCATTTTTTCACATAGAGTAGTGATAAAATAGGACTTGTAGGTCGGAATTTTTTTTTTTTTTTTTTTGCCCTTTCTTCGACTGGATATTCATCCGGTGGATACCCCCATTTTTGTGATACGGTGTCCATGGTTGGCCTATGAAGGGGGTATAAATACCAAGTCGACCAAATTTGTTTTGTTCTTTTTTAGGTCGTTAACACTGTATAAGCTAAGCTTCTAGTAAAAAATGAATTTGCTTACGGTTCTGTTGTTAAACCTGAGATTCCTTTGAAATTTTTTGTTGATCTCTCAGCTCTCAATAATATAAAATCGAAATTCATTTGAGACACCAA

>CsJMJ3

ATGGGGAAGGGAAGAACTAGTGCTGTGTTAGGGCAAAAATTAAGTGTGGCATCAACATCAAAATCAGCATCATTGAGTATACCATCTGGGCCGGTGTATTATCCTACTGAAGATGAATTTAAGGACCCATTAGAGTATATTTGTAAAATTAGGGCAGAGGCTGAGCGTTATGGGATTTGCAAGATTGTTCCACCTAAAAGTTGGAAACCACCATTTGCTTTGGATTTAGGTTCTTTTACATTTCCCACAAAAACACAGGCAATTCACCAGTTGCAAGCGAGGTCGGCCGCTTGTGATTCTAAGACTTTTGAGTTGGAATACAGTAGGTTTTTGAAGGAACATGTTGGGACGAAGTTGAATAAGAAGGTGTTCTTTGAAGGCGAGGAGTTGGACTTGTGTAAATTGTTTAATGCAGCAAAGAGGTTTGGAGGGTATGATAAAGTTGTGAAGGAGAAGAAGTGGGGGGAGGTTTTTAGGTTTGTTAGGTCAAATAGGAAGATTTCTGACTGTGCTAAGCACGTTTTGTGTCAATTGTACTATAAACATTTGTATGATTATGAAAAATATTACAATAAGTTGAATAAAGAGGTGACAAAAGGTTGCAAGAGGGGGTTAGATGGGGATGTAAAGAGTGAAGACAAGGTTGAGCGTTCGAGCTCGAAAAGGAGGCGAAGGAATAACTGTGATCAGGAGAGAGTTAAGGTTTGTCATAAGGTTGTTAAGGAGGATGAGCTTGATCAGATTTGTGAGCAATGCAAAAGTGGGTTGCATGGGGAAGTTATGCTTTTGTGCGATAGGTGTAACAAGGGGTGGCACGTTTATTGTTTGTCACCCCCATTGAAGCATGTTCCTCCAGGGAATTGGTATTGCCTAGAGTGCTTGAATTCTGATAAGGACAGTTTTGGTTTCGTGCCAGGTAAGCGGTATACAGTGGAATCTTTCAGACGAGTAGCTGATCGGGCCAAAAAGAAATGGTTTAGGTCAGGATCTGCTTCGCGTGTTCAAATGGAGAAAAAGTTTTGGGAAATTGTGGAGGGAGCAGCTGGTAATGTTGAAGTTATGTATGGCAGTGACTTGGACACTTCCATTTATGGAAGTGGTTTTCCTCGTGTATGTGACCATAGACCAGAATCCGTGGATGCCAATGTATGGAATGAATATTGTAATAGCCCGTGGAATCTAAATAACCTGCCAAAGTTGAAAGGGTCAATACTCCGGATGGTTCATCATAACATTACTGGGGTGATGGTGCCTTGGCTTTATCTTGGCATGTTGTTCTCAGCTTTTTGCTGGCATTTTGAAGATCACTGTTTTTACTCAATGAATTATCATCACTGGTATGTAGCCACGCTGATTTACCCCCTTTTTTGGTAGTGTTGTCTTGGAGCAAACACATTATTGGTCGCGTTATTTTCCCTTCCAGTTTTTCATCTTAGGAACTCTTCATTTATTTTTTAAACGAATTAATGCATTGAAGTGGTTACTTTAGCATCTACCACCATTGAGCTGTAAAGAAAGTTGATACTTGGTAGAGTGTAGGTGAGTTGTCTTTTTAATACTTATTGGCATTGGGAGTTTTACTTGCCCTTACAAAGGCGTTTCAATTGCTTATTAATTGTCATGATTTTTTATTTTATTTTTTAAAATTTCAGTTAAATATCCAAAACTTGGTCTGGTGCTAAGTAGGTAGAATGGAATATCAATTTCTCATTTTCTTGTACTTCTGGTGGTTCAGTGTGATGGATACTTCTTTTATGCTTTGCAAGGTACTGCTAGCTAAACTTTTAAATTGATTTAGTTAGAAAGTTGATTCACTGCTCTTATGTCATAGGGGAGATCCAAAATGTTGGTACAGTGTCCCTGGAAGTGAAGCTGGTGCTTTTGAGAAGGTAGTTCTTCATATTGGAATTCATCTTTTCAGCTTTTTATTTAAAAGAAAAAAAAAGCCTGCCATTGCTTTTTTCTTTTTTAAATTGTTATTCAGGTTCTTTATTCCTCCCTAATTTGAAAGAAGAGGGGTATGCTATGTGTTGGCTGAGCTTATATACCCAGCGAATTTTGATTGTTCTTTTCGATGATGATTCATGTTGACTTTTGGCTACATCCTATCATGCACAAGAGAGCAAGTCACTGTGAAAGTTTGATTGTGCACTGTTGTATTCCACGGTATTGATTGACAGTTTCTCCTTCCAAACAAAATGATCATGACTGATGTCAACCTTCTTTTTTCAGGTTATGCGGAGTAGCCTTCCTGATCTATTTGATGCACAACCTGATTTGCTCTTTCAACTTGTCACTATGTTGAATCCATCAGTGTTGGTAGAAAATGGTGTTCCAGTCTACAGTGTACTACAGGTTTGTATTTTTAATATCACCGTTTTGCTTCTTATATGGTACATTTTTGTCTATATTCGTTGTACAGTTTCTTTTTTAGTCAACATATGAAATGACTAGGCCTTTAGAAAATGTGTTATTTTTTTGTGGTAGTCAATGCCATTTTTTTAATGTATTTAGTTGGATGCATTTTATGTGGCTCTTTTGTGTGTATTTTTTGGCAGGAGCCTGGAAACTTTGTCATCACATTCCCCAGATCTTACCACGCAGGATTTAATTTTGGTCTGCTGACTGCCATAATGTTCTCTATTAATGATGATTGGTTTAGAAGGATGCTTTTGTCTCCTTTAATGAGTTACTTGCACTTGATGAAATTTTTTGCTCTCTGGTCTTTTCAGGTTTAAACTGTGCAGAGGCTGTCAATTTTGCTCCTGCTGACTGGCTACCTCATGGTGGTTTTGGAGCTGATCTGTATCAGCAGTATCACAAAGCTGCCGTTTTATCTCATGAGGAGCTCCTTTGTGTTGTAGCCAAGGTTGTAAGTTTTCTGACCTTTCTTACGTGCTTTAGTTGAACTAAGATTGAAGAGGAGGACATTCATATTTTCATGGATGTAAAAACAGATGAAGTAGAAATGAGACTTAGGCCCTCAAGAATATATAGAGAACACATGGCATAATATGAAGTCACTGTCCAAACTGCAGTAATAGGTTATAACTCATAATTGAGTATACACTGCTTCCCAGAGTCTTTCTGTGAAAGGTTGACCTCAGGGCACCGAACTTTTAAACTAACAATGTGATGTGAAAACTTTGTACTGAACTGTTGAAGAGATGAAAATGCTGTTGCAAAGGGTCCCTTACATAGGTATATTTATGTTTGAACTAGGAATAAGTAGAGTTTAATCTAGGAGGGACTAGTGGAGTATAATTGAGGAGAGTTAGTTTATCTTAAGTTCCACGAGATCTTTCCTGTAATGCTATGCTCTTGTTATTCATACTTCTGCAGTTGAGATGGAGAGTTCAAAATCTCTGAGGTTGTTTTTCCATTTTTCCTTCCTATAAGTAATTTCTTTATGCACGAACTGGAATGTGTATGTGTGTGGAACTTAGTCTATTAATTGGCCGTGCTATCAGCTACTTCTGCTTATTATTAGGGCATCTGGTTTGCTTCTTGTCTACCTTTAGTCATCGTAGATAATTCTAGCTAGTTACTACCAAAAAAAAAAGCATTTTGCACTTTCATTTGATTTCTGCTAGTTGAATTATGTTTTCGTCTGTCTTTTGCTGCTCTTTTCCTAGCTAATTGATCATTATTTTGTTTTGAGCAGAGTGATCTTGATAGCAAAGTGTCACCTTATTTGAAGAGAGAATTGCTGAGAGTATATACCAAAGAAAGAATGTGGAGAGAGAGGCTTTGGAGAAAAGGCATCATCAAGTCAACTCCTATGGGCCCTCGGAAATGCCCAGAGTATGTGGGTACTGAAGAGGTAATTCCATATTTTCATTTTATATTTGGTTCATGGCTTGATACAATATGGTTATATTGATTGTGATAAATTCTTTTGATGGATTTTCTAGGATCCAACATGTATTATATGTCGGCAATATTTGTATCTCAGTGCTGTTGCTTGCCGTTGTAGGCCAGCTGCTTTTGTGTGTTTGGAGGTGAGCAGAGTTTTGTTGTTAAGATGCTACAGTAATTTTATGGTTATTTAAATTATCTGCATGTTCCATCTGTGGTATTCCTTTCAGTGTAGACTTTGAGAAACTTTATTAATGAGAAGGAAATACCCAGGTAGTCCACCTGGTTTTACCATGGATGGGGGTAGTGGAGTAGACTTTACAATTTGTATGTTTTCCTGAGTTGGCTTACAAGTAAAGACTTTAATTCTATTAGCTTCAGCCTTTTGGCCCTTTTTATACAACCATAAGTTGAATTTAATTTTGTTCTTTATCAAGACTTTTTTTTTCCTTCTTTGAATCTTAATCTATTAAAAAAGTCTTTTCTTTCTTAACTAGTTCGGTGCACATTAGAACATGCACTCATTTCAAGGGGGTTCAACACTTCTCAGTTTGGGCCTCCCCAGAAATTGTTGGATTTCTTGCAATATGAAATTGAATGCCATAACTCTTATTTGTACTTTTTATTGGTGCTGTTGGGATATACTAACTTATTGGCTTATTGGTGGATTTCCTTATTTATCGATCAGGATCAGAGTTTTAGGAGAAAAGAACAGTGACACCCCTGTTGTTTTGGCGAAAAGATCGTGGACAATTCCTTGTTTTCAGAAATCGTCGCATAGATTCCTTTTGGGGCACATAAACCCCCTTTATAGCCAAACCTTTGGGGTCGTATCATTTCTGAACACAAAAGGGGTTCTGTGACCATTTTTGAAAATAAGTGGCCTTCATGACCATTTTGTTAAACCATGAGGGCGTCGTCCTTCCTTTTTCATATTTTTAGGTTAATGAAATCAAGTAGATTGAAAATCTTGTAGATGCAAAAGGCGCGTTATTGTATGTTTATTCATTTTCTTATCTGAAGTTGGAATGCCATTGATAAGCTTCTCAAACTGTTGAATCTTCTATGTTCAACATTTTTGTTCAAGTTTTTATTATTGCATTTTATATTCTTACTCTTTTCGTGAATAATCTGATAAATAAGATGATTCAAATCAATTTGGGCCTCACTAAAATTTTTCTGCTTTTTAAAATTTCAAATCTACTGTTGCAATTTCTCCTAGGCAATTACTGATTCTATGTGGCTTTTATGCAGCACTGGGAGCACCTCTGCGAATGTAAAACCAGAAAACTTCATCTTCTTTATCGTCATACCTTGGCAGAATTGTATGATTTGTTTCTTACCGTTGATAGAAATAGTTCTGAGGAGACATCAGAAAGTAATAATTTAAGAAGACAGATCTCATCTTCCAACCGGCCAACTACTTTGACCAAAAAGGTTTAGTTATAACTTCTTTGTTCTTTTCTGTTTCTGAGTATGAGCAACTTTCTTACATAATGCTTTTGGTTGATAGGTTATCTTATTTTATGGGTTTTAAATTATTGTGACTGAAGAGTTCTTAGTGCGACAAATTGAGTACTTACTACCTGGCTATGCATTTGTCCTTGATCATGCTGGGGGTTTCCTTTCAAATATTTCATTGCATCTCACTATTAAGACTGCTTTCAAACAGTGGAAATCTGTGCTTCATTCTTGATATTTTGTCATTCAAGTTGGATGAGAACATAGAGATTTGGTGAATTGTCTATTTTCCCTGGTTGGATTATTGGAGTTATGAGACCAGTTGGTCTTTAAGAGGCAGATGTAACAAGAGAGTCTTCTTTGTGCATCCTTTTGTTATTTAAGTGTTAATTAATTCTCCTTTCTTAGCAGATATGGGCTGTCTTTCGGGAACTTTGAAGAATTTTTTTTTTTTTTTCTATTTTGAGACTTGAAGAATTATTTGAAACTAATTGAATTCATATTTTCTAATTCCCCTGGTATGTATAGAGTAAATTTATTGTGGTGACTACATATAGCAATTATATTATTATTTTTATGATGCTTGCATTTGTGGAGATTATCCATGTTTGTATAAATCATGATATCAGCTTACAAAAGTTTTATTTCTAATAGATGTGATATCTTGATATTAATTGTGTGGTGAAGCTGTGTCTTTGATGAGCAAATATTTCTGCCAAATGTTAAGATTGAACTATCTGAGCCATTCCTTTGGAATTCTGAAAAGTTTCCTGAGTAATGCTATACTTACTCAGGATACTTAGTTATATCCCATTTGCTGTGGCATCTTATATGCATCAATTAATTGGGTTTGTAAAAATAAAATCAACCAATGCCATACACATGGCAATAAATAATTCATGACATATTAGTTGGTATACAAATTGAGCATCCCCACTGAGTAAGCATAGCATTATTGAATATTATATTAAGAATGTCAAATGGATGCAGGTCAAAGGTGTTCGCGTTACCATGTCTCAGCTTGTTGAACAATGGCTTTCTTGTTCACTTAAGGTTCTTCAGGGCCTGTTTTCTAGTGATGCTTATGGCACTCTTTTGAGGGAAGGTGAACAATTTCTTTGGGCTGGTTTTGAGATGGATGCGGTGAGATTTGGGCCCTCAATTGTTTCACGAACAGTTGAATAAATAAATTTATTACATCTTGTAACAATAATTTACTCACCAAGAAAAAAAATGTGTGTTTTGTATATAGGTCAGGGACATGGTGAACAAATTGATTGAAGGTCGGAGATGGGCAGAAGGGATAAGAGATTGCCTTCATAAAGCAGAGAACTGGTCATCTCTTCCTGGCTCTGATTCAGAGAAAGTGCGCCTGGATTGTGTTAATGAGTTGCTTGGTTTTGATCCTTTGCCGTGTAATGAGCCTGGACATCTTATCCTGCAGGTTGTAATGCTTTACAGAAAGCTGTACTTTATAAGTGTTGCCTCACAGATGTATGATCAGGAATTTGCAAGGAAACGTTATCATTTCTTCTATCATCAAGACATTTACTTATAGCTATGAACCTTGTTTCAAAATTGTTTATGCAGCTTACTTATGGCTATGAATTTCAAAATTCTTTATGTAGCTTTTACTTGCATCTTCTCCACCTAATCTGCCAATTGAAATTTCCACTTGATCTGCCTATTGAAATCTCTACTATTTTTATTTTTATTCCTAATCAGAATTATGCAGAAGAGGCTAGGTCACTGATTCAGGAAATTAATGCTGCCCTCTCAGCTTGTTCAAAGGTATGCGTCTGCTCTCTTATGACTGATTCCAATTAGTAGTATTGTGTTTAAGTTGATAGAAAGTGTGCTCTGGCTGAATTGATTGGTTTTTATTAACTATTGTTTATTTTTCGTCATTGTCTCTCCAGATTTCTGAGTTGGAGCTATTGTATTCCAGAGCTTCTGGCTTACCAATTTGTATAGTAGAAAGTGAGAAGCTATCTCAGAGAATTTCTTCTGCAAAGGTATATTGAATTGTCAAATTTAGTTATTATACGTGTTTGTGCATTAATTCAAGTTCTTTCTTTTTGTTCTTGATAATCCTTTGTTTTGTCTTTTGTATGTGTTTCTTTCTTGGGGGCTGTGGTTATTTAACATTCATTAATGCATGCTTTAAATTTGATTGTGGTCGGTGGTGTGACTATAGTGAATAATGCTGCTGTCATTTTACTGTTCTTTAGGTCTGGAGAGACAGTGTGAGAAAATGCATCTCGAACAAATGTCCTGCAGCAATCGAGATTGATGTTCTTTACAAGTTAGAGTCAGAGGTAACCTTAATTTCTTAGTCTGTGCTTCTGTATGGTTTGACATTTTCTAAAGTATTGGCTCTAATATTTCATGTTATGTTGAACAGGCATTGGATCTGAAAATCGATGTTCCAGAGACAGATATGCTTTTGAAAATGATAGGGCAAGCCGAATCATGTAGGGCTCGATGCAGTGAAGCATTGAGGGGTTCTATGAGTCTAAAGGTTAGCCTAGTTGATCTCTTTTGTTTCTGTCTCTTTCTCTTCTATGCATTCATCGAGTTTTTCTTTGGTTAAGTTGTAAGTTTTCAAAATCTTGACTTTTCAGACTGTTGAACTGCTTCTTCAAGAACTGGGTGATTTAACTGTTAATATGCCGGAATTGGAGCTTCTAAAGCAATACCGCAGTGATGCCATTTTTTGGATTGCCCGCTTAAATGATATTTTGGTGAACATTAATGGAAGGAAGGATCAGCACAATGTAATTGATGAATTAAATTGCATTTTGAAAGAAGGAGCATCTTTGAGAATTCAAGGTTTCTTACTTTTGATATGCAATTTCCTTGTTACAGTCCCATCTTTGCCCATTTTTTGTTTCCATTTTATGTCTCCTTAATGCTGGTTCTATTTTATTTCTTTGAGGTCAGTTGATGACTTGCCTCTTGTTGAGGTTGAGCTGAAGAAGGCTCATTGCAGAGAGAAAGCTCTGAAGGTAATTCCTTGTTCTGTTTCCTCTCAAACTTCTGTTTGTTGGTGTTTATGTTATAACAATGGATCTTATTGTGCTCCTTTCTCTCTGGTCTTTGTTCGTTTTGATAGCTTCAGATGACCCAAAAAGTGACTGGTTTTCATTAATTGAGTCTGATGCAATGCTAATTTGTTTAGGCTTCAAGCTTTTAGTGGATGCTTATTATTTGATTTAATAAAATGTTTGGAACTTCCTTACAGGCATGTGATACTAAAATGCCTCTGGACTTCATTCGGCAGGTGACGGCGGAGGCTGTCATGTATGCTCTCTTGCCTGAATGCAATCTCAAAAATTGAGTGTGTGGAAGGTTTTTCACTTTTTTGTACATGATATCTTATGTAAGTTTTACGTGGCATATCTGTTTACCAGACTACAGATTGAGAGAGAAAAATTATTCATTGATTTGTCTGGAGTACTGGCCGCTGCTATGCGTTGGGAGGAAAGAGCAGCAGATATTCTTATACACAAGGCTCAGATGTGCGAATTTGAGGATATTATCAGGTTTGGGCATTTCTTATCTTATTTTCTTATTTTTTTGTTTTGTAATGCTTCTTTATTTCTTTTTACGGTTTGAGTTCCTTTGCTAACTTCCTTTTGTCTTATTATCATTGAATTCAAATCTTTTAGAGCTTCACAAGACATATTTGTGGTTCTACCTTCACTAGATGAAGTCCAAAATGAAATATCAACAGCTAAATCATGGTTAAAGAATTCTGAGCTATTTTTAGCTTCTGCGTTTGCTGTAGCACCTGCCTCCTGTTCTTTGCTGAGGCTTGAGAGTTTGAAGGTTCTTTTTATGTTAATAGACTCTTCTTTTTTCACGTCGTCTCCTGCATGGTGTTCAAAAACTGGGTGGAGCACAAAGTATGACAAGTCGATCTAAATGTTGCAGGACTTGGTTTCTCAGTCAAAGTTTCTTAAGATATCATTGAAAGAGCAAACAGAGCTTGAAAAAGTTATAAATAATTGTGAGAGATGGCAAAATCATGCCTCTTCTCTTCTACAAGATGCTAGATGCTTACTTGATAAAGATGACATTGGTGATGGTCTGAGCAACAGTCTTGTTTCAAAAATTGAACAGCTAATTACATCAATGGAATCCGCTGCGAATTGTGGTTTGTCTCTTGGTTTTGACTTCCATGAGATTTCAGAACTTCAAAATGCATGTTCTACCCTGCGTTGGTGCAAGAAGGCTCTTTCTTTCCTCTCTGTTTCTCCTTCTTTGGAGGTGATCATTTGTTTTTCAATTATTTGCATGACCTTAGTTTATCCTGATTTTTGTGTTAGACGATCTTGTGGTTTTTTCCTTCTATTAAAAGTCTAATCTGTAGAAGAGCCATGCTTCTTTATGGGAATTTGTATAAAATTGCCCTAAATTTGTGCATATAATTTATATCTGTTCTTCTTGTTTCTTCCTTTCTTTTTAGGTGGCTTTTCCTTTCTCATCTGATACTTGAAACTTACCAGAACAGCTTTACAAATTGATTGTTCTAAACAATACCTATTCTTACACATTGGCATATTATGAAGTTTACTCTGTCTTACCTGTAGGATGTGGAGAGCTTGATGGCTGTTGCAGAAGGCCTTTCTACCAGATGTTTTTCAAGTATGTTGTGGAACTCTTTGATTCATGGGGTTAAATGGCTTAAAAGAGCATTGGAAGTCATTTCTGCTCCGTGTAAGTTTAAAAGATGTAAGCTGAGTGATGTTGAAGAAGTTCTTGCTGGATGTAAGGTAGTATTATAATTATACATGTTAGTTCTCTGTAGTGCCTTTGGGTCCTCAAGAATATGCAGCTCTAACTTTGATTCTGAAGATACTGTATTTGGTCTTGTCAAGGAATCAGTGCTATGCCGAACTTACACTTGGTTTTTCTGCTTGCAGGGAATTAACGTCTCCTTTCCAGTTGTGATTGGTGAACTTACGAGTGCTATTCAGAAACACAAGTGAGTTTTATGATTATTCTATATGTTTTTTTCTTTTCATTATTTCTTTTTCTAACCTAAAATTTGACATGTAGATGCATAGTGGTTATTGGGTCTCTTTGAATGATGCTCTAACAAATGTATTCAATACATGTCGTTTATACATCATGTAATGCATGGTGATGTTATATGTCAGACTTGGGAATCTTTCTTAGACAGGTCCATTGGGATTTTGATCTAATGTCGAGCACTGTAAATAAATGATTATTATGAGGACAAACTCACTAACATGAAGCATATAGAGACATATACCTTCCCATTATGGTTTGTCTCTATTGATTATTAGCAACTTTTTGCATAGTTTATTGCTCTGTTGGTTTTAACTGTAATGCAGGTTGTGGCAAGAACAAGTTCATCAATTCTTTAATCTCAAGTGTGCGCAACAATCTTGGTCTCTTATGTTACAGCTCAAGGTATGCTGTCTGTGTGCTTGATAAACTGTGATTACACATGAATGCGTTATGCTTATTTATTCCTTTTCTTACCCCTTTTGCATAAATCCTCTTTGAGAATCTCAAGCACTATGTTTATTAGTGTTGCTTGCTTTAAAATTTTATCATAAACAGGAACTAGGAGAAGCTGCTGCTTTTGATTGTCCAGAGCTAGAGAAGGTTCTATCCGAAGTTGATAAGGTTGAAAACTGGAAGCAACGCTGCAAGGAAATTGTAGGGACTTCTGTAGGAGATAAGAATTCTTTGCTTGGTCTTTTGCAGAAGGTCTTTATCAGATGATTGTTTTTATTTCCTACAGCTTAGATGGTGGTCGTTTAGATATTTATCATGTAAACTGAGAATCATTTTTAATATTGTGCAGATCAAACAGTCTCTACATAGATCACTGTACATATATAACAAGCCACATGGTTCAGTATCGATGACTCTGTGTATGTGCTGTGAGAGTGATTCCAAGGAGCTTGAATTCTTAATTTGTTCTGCATGCAAAGACTGGTAAGGTATTTGTTGCTATGTTCAATTTCATATTTGATTTTTGTCTCTGTGGTAGGCTAATAGTCAGGGGTTCTACTATTTAGGCTCCTTTTCATGTAAGAATATTGTGGAAACTTTGAGCAGCAACCCAATATCTAAAATGTAAATTCCTCTGACCTTAAGAGATATCTGTTAGTGATCTTTGAAGTAGTTGTAATTGGTTTGTGATTCACTCATCAGTTTCTCTATGAAATGCATATCTAGTCATGATTTCTGGTTGCGTTGCTGCATGTTCTTCTAAATTCATCTGATCTGGCAAATGCATGATTTTGATTTCTTCCATATGAAACAGCTATCATTTGCAGTGCTTAAGACCAACAGAAGTTAATAGAAATCATGCAGAAGCCTACATATGTCCTTATTGCCAATATTTTGAGAGTGAATCAGTATCTCAGTTCGGAGGCAGCCCTCTGGTGTGACAATTTCCATCTGTTACCCCAATTTTCTTTTGGAGTAATTCATAGCAGAATCTGATTTTATTTCTTCTTGTGAACAGAGATTTGGAGGGAAGCGCCCTGATTTGAGAATGTTGATTGAGCTTCTCTCAGATTCTGATTTTTTTTGCAGAGGGTAAGCTGCTATGTGTATGCTTATCATGTTAAAAGCATCCTGTATCAAATCAATAGTTGATCTTTAAGAATTTTTTTCATCTTTCACTTACCGCATCTTGTTACTTCTTCTATGAAAAGTTTGTGGAGTATTTTTTAAAAGGAAAATACATGAAAGTTTTAAGAAACTTGGGGATTAGGGAACCGTATAATTCTTTTTTTTTTTTTTTTTTGTAGCATCTTTTTAAACCTTTAATTACAATTAGCATTTATGAAATATGATGCATGCTAGTCTTAGTTTTCTTGTCCCTCTATAAGTCAATTCAGACTTTAATTATGTTATTGCATTCTGCAAACTAGTTGGGAGTGTTGATAACATGTTTGTTCAGCTCCAGAATCTATGATCCTTTCTTGAAAGTTCAAACATACATTTATTTGGACAAGTGCTGTTGCTTTTTACAGGATTGAAGCAAAAGATGTACTGCAAGAAGTTGTGGATGTAGCTCTTGAATGCAAGACCTGCTTGACTGATATAGTGAAGTTTGAATCCTGTTATTTGGATAAAGATCTCCACGTCATCTCCAATAAATTAACTATCACTTTGAAGGCACTTATTTCAATCTTTCTATCTCTGCCTTGTATTCCGCAGAGGGTTTTTCTTTTTCTTTTGGTTTTTTGTGTTTAGTCATAGATTCAGCATATTTCTCTCTCTATCCCAATTTGTGATGGTCATCTTCCAGTCATTAGAGACGGCACTAGCCACCATAAAAATGAAAATAGCATAATTACTGACTGGCTTGATTATCCTGATTAGAGGTGGTTCTGCTGCACAGGCCAGGGAAGCAGCTGGAGTTTTCGATCGTCAAAGTAATAGCGCACTTGACTTTGCATTAGCGAGAAACTTGTGGAGAGTTAGAGTTAGTAAGTTATTAGAGGGTTTAACAAAGCCTACAATTGGACAGATACAAAATTATTTGAAAGAGGTATGTTGCCCCTGGCTTCTCAGAGCATTGTACAGATCATGATGAAATTTTGTTGACCTAGAATAATCTTCTTGTGGTTTGTTACTGGTGTTTTAATTGTGAAATTGGGATGACAGTGTGAAAACATCAGCTGATAGTGTAGTGATGTGTGTGTGTGTGTGTGCATCCAAAGTAATAGTAAATAAATATACTCAGGGTAATTTAGTACTTTATATTTCGAAAAGCACTAGGAAAATGGTATTATAGTCACAAATATCTAATCTCTTAGATTTTAATGGGAGCAAATATGGTTGTGCCATGAACACTAGCATGAATGAATATGAAATTGTGCATGGAACTGTGCTGGTGATCCTTGAGGTTTTAGGTCGGTGAGAAGTCAAACCCTCACCATCCTTCATGTTTTTGCTGGTGGTGAGGTGTTTGTATTTGGAATTTGTGTTTCAATTTCTGCCTGAATTTGTGATTTTTGCATATTTCTCTAGCCAATGTTGAAATATCATTGTCAAATAGCATCTGGTACCTTGGAGATGGATGACATGTTTTATGGTCTATATTCGTTATGGTTTTCCTTTTACTAATTTGATTATTAGCCTCTCTGTCTGCACTGGACCTCTAGTCCAGCTTACTTTTTGATAGTAATAGACGTATATCATTTTTTATCAAAAAAGATTATTAGCCATTTTAAGTATGGTGACAACCCAATGATATGTTCCCGCTAAAAAAATTGTAGTGTGTTTTTCTGTTTGTGCATTTAATCTGATTGGATGTAGATATCATTGTAAGAAATTGTGTATTTATTACCCACCATTATTATTTTCTCTATTTCAAATTTTCTGTTTTGCAGGGAATTTTTTGCTTTTTATTTATAACTTCTCCTAAGATGGATAATCTGTGCAGACTTTTCTTGCTCTGTTTGGCAATATGCACATTACAATATAAATGTTAGCAATGGAACTTTTTATATGAATGTGCTATAGGTGCTAATTGCAGTTACAAATACTTTGTAGGGACTGTTAATGAACATATCACCCAAAGATCACTACAGGCAAAAGCTGATGGAACTGAATCGCATTGGCTCGCAGTGGGCAGATGTTGCCAAAAAGGTGCATAGATTTTATTTTTTATTTTTTATTTTTTTTGGGGGTTGTGTTTGCAGTTAAGTGAAATCTCCCTCAACTGTACCACTTAAATGTTCTTTCTTGTTGATTTGGTATTATTTATATGATTATTATTACAATTTTTGTTTTCTTTTACAGGTAGTACTGGATTCTGGGGCTCTAAGCTTAGATAAAGTCTTTGAACTTATTGCAGAGGGAGAAAATTTGCCTGTTTACTTGGAGAAGGAACTTAAGGTAACTTATTTGGTTGCCGTAAAGCCGAATGCTTCACATTTTCATCAATTATTTATTTTCTTGATTGGTGCAGTCACTAAGAGCTCGAAGTATGCTATACTGCATCTGTCGGAAGCCCTATGATGAGAAAGCAATGATCGCTTGTTATCAGTGTGATGAGTGGTATCATATTGATTGTGTAAAATTACTTTCTGCCCCAGAGATCTACATCTGTGCAGCATGTAAGCCTCAAGCAGAAGAGTCTTCTACTCCACAAAATGTGGATGGTGGGAGGTAAGAATGATACTCATCCATGGATATTGTCACTGTGTGAGCATGTTCTGTTCTTCGTGTTTCCTCAATGTTTTTCAGGTGGTGCTTGTTTTTGTCCCCCCCACGCCCCTGCCCATGGGATGTAATTTAGTGAAAAAAATACAGTAAAATGACTGTTTTTTCTTGTATGACTTTGACAGAACAAACGCCGAATTTCTAGAGCCCAAAACACCTTCTCCTAAACACACAAACTCGAGAAAGAAACTGAGGAAGGCTGAGCCAGGCCTAGCGCAGAAGATGCTAGCAATCGCAAATAACAGCAGTGTATTTGACTGTTCTAGTGGAATTGATAACTTATGGTGGCATAATAGGAAGCCTTTCAGAAGAGCAGCTAAAAAACGCACAGTGCTGGATAGTCTCTCTCCATTTATTTACACACAACAGTGA

>CsJMJ4

CTCGTCGCTCTTTTTCGGTTTTCTAAAGCTTTCTCTTTCTCTCTCTCTCTTTCATCCACATTTTTCTCTCTCTTCCAATTTCTCGCCGGAAAACCACTTTTTCAGTCGAAAAACCGCTTCGTCCGGCGAGAACTTGTCTCCGTCGCCGGAAACCGCTCATTCTGTCTATTTCAATTGCTTCCAAGCGATTCAATCCTTTGGCGTGACGAATTTCTCGAAGGAATTGGATCTCCAAAGTTTGGCTTGGTTTCGACGTTGCTTGATAGAATGTGGCTGGCGTTGAAGGCTCCGTTTTCATCTCTGAACAGATAAGGTCGTGATCTAGGGTTTGCTTTGCGGATTCGGTTCGAGTTTCGTTGTTTTTCATTTTTCTTGTTTTGTCTGTTCGTTGAATCGCAAGTTAAGACGGAGGCCTGTCGGGAACGATTGCGGAATGAAATGGTACGTGATTCATGCTTTCATTTGTTTTCATTTTTTCGTTCTTTTTTTTTTGTTTTCAATTCCATTTTTTAGGTTTCTTTTTTGAAAAGTATAAGAATTTGTATTCGTTTATGCTGTTCAGATCTTACACATTGTTGTAATTTTATCACCATTTCTTAAACGTTTTGCTGTATTTTCATTTCCCTTCATTTTTTATAAATTTGTTTCGGAATTGCTGTAGTTATTTATTTTTTGATAATGGAATCAGTAGAAGGTTCATGACATATATCAATTTGGTAGATGTATTTCAAATGCAATTGAATTGATTCTGATGTCTGATGTAATTTTTTTTATACTAATTATAAAATTTAAATTTTATATTTTCTTTTTCGGAATTGCACTGAGCGATCGCTGAACATAGGCGCCAGATGGATGGTGGGGAGATGCTGCCATTTCCCTTTAATATCTTTAATTTGTTGAACATTTGTTCTTGCTTTTGATCGTTTGACCATTTGGATAAGATTGAGGGCAGTGATTTAACGATAACAATATTATTTTCTTTTAATTTTGAAAAAAAAAATTATTGGGTTTTGGGAAAAAGGATATGGCTAGACCCCAGATGAGTCAATTTATTTTTGTTTGCTGTTGAAAATATGTGGAGGACGTATGGATGTATTAAACTGAAAAGATCAACATGGGTGCTGCAATCAGCCAATTACTCTTTTTTGACTTGAACAAAACCGATAAATAAAATTTGATGTGGTCTTCTTTGGTTGACTTTTGGATTTATTCATATTTTGGGTTTAAATCTTCTTCCTTTCTTAGTCTTATTCTTCTGGGTAATTGAGATTTTATATCATGAATTGCATTCTTAATAAAGATGATGAAGTTCCTTGAAATTCCTTCCAGTCTTACACACTTGAGTTGTTTGCAGCTGTATTTTTCCCATTGTTAAAATTGATGGATCCAACTTTAAGTTTGTGTTGAATTAAATGATAAATGTAGATGCCAAAGGTAAGGTTTGTCAGGTTGCAGAACAAGGAATTTTGCGACTATTTCTGGTTGACATATGTGTGCTATCTGAAATCAATTTTTAGTGTTCCAGTATGACTCATTGAGTGGTAGTCATCCATAATTTCTCAGATTCTTTCAATGCAGTGGACATTTATAGTCATAGTTAGCTGAAGCTATAAAATGTTGGTTGTGTCAAAGCTCTGTGAAATATGACTGAGAATTTTTATGGTGCAATTAGATTTCATTTAATAGTATTTATGCGTCAAAATTTTGTTTGGTAATCTTATGTTGAAGAATTCCCAGGTGAAAAGTTCTTAGAAAATGGTCTTCTAATTTCAATTAGCGTACAAGTGATGTTGGCCATTATGGCACTTTGTTCTGTAAGGTGTTGTTTCCTTGTTTTGTTTCTAATATTATTGGTATTGCACAGTTTTACAAGAAATTTCAAACATTAGTTTTTTTTTTTTTTTTTTTTTTNNNNNNNNNNNNNNNNNNNNNNNNNNNNNNNNNNNNNNNNNNNNNNNNNNNNNNNNNNNNNNNNNNNNNNNNNNNNNNNNNNNNNNNNNNNNNNNNNNNNNNNNNNNNNNNNNNNNNNNNNNNNNNNNNNNNNNNNNNNNNNNNNNNNNNNNNNNNNNNNNNNNNNNNNNNNNNNNNNNNNNNNNNNNNNNNNNNNNNNNNNNNNNNNNNNNNNNNNNNNNNNNNNNNNNNNNNNNNNNNNNNNNNNNNNNNNNNNNNNNNNNNNNNNNNNNNNNNNNNNNNNNNNNNNNNNNNNNNNNNNNNNNNNNNNNNNTTTTTTTTTTTTTTTTTTTTTTTTTAACTCATCTAGTTGTCTTATCTACTTTAATTTCTGCTGACATACTTGTTAACCTGAAAATATTGTTTTGTGTTTAATTGTGACAGGTTGAAGGGAAGGTATGTTTATCAAAAGAGGCTAGAAATGGCTTGGAATTTTTGAAGCGTAAAAAGCTTCAGCGAATGAAATCAGAAACTGTCAATGAGACTATTGGTATCTCCAATATGATGTCTAGAAGTGGAGGAGATGCTTTAAGAGCTTCAGCTTCATGTGGTATTAGATTACATGGTAACGCCGATTCATTTTCTCGGCCAAATACTGCACCAACTGGGAAAGTTGTCTTTTCAAAGCGCAAGGTGGATAAGTTTGACACAAATGATTTGGATTGGACTGAGAAAATTCCAGAGTGTCCTGTGTTCCGCCCAACAAAGGAGGAATTTGCGGATCCTTTAGTTTACTTGCAGAAGATAGCTCCAGAAGCTTCAAGTTATGGTAATTTCCCTTCTATTGTTTATGTGAAATGAATGTTGGTTTTGTGTGATTTTGTTTTCAGCGTACTAAAAATTTTGTATTTATGAACTGAGTAGCCTCAACTTGAGGACACCATTTGGAAAAAATGTTTATATTTATGCTCAAGTATAGAACTAGTGGAATCTGTATTACATGCTGATGAGCCAATTATATACCACAGTTTGAGCATTGAATCAAAATAAATTTCTGACAACCTTTTTTTTTTCATAGTCTTATTATCGTAGAACTAATTTTATTTTTAGACAAATAGTTTTAACGAGTAATTGTATAAGTGATTAAAAAGGGGTAATAGCGGGTGCCCTAAGCATAATTGTCGGGCATTTGTATTCATTTTGAAGGCTTGATATCTGAAAATCAAACGATTTATTATTTACACAATTCTTTGAGTGTTAGGAGATTGGGAAATCTTTAATCATAGAAATTGATTTGTATATCCTCGTAAAAGTTAATATTTTAATCCGTTCTGTTATCTGCCATTTTCTTGGTTAATATGGTAGGCAACAGTGTTTTCTCTTCTGTGGAAGATTTACCTCATCCAATGTTTGTGATTATGGATATTGAAACTCTTTTCCAGTTTTCTTCTGTCCATATAGGTACTAGTTTGACTGACTTTTTCTCTTCTCAAATCTACACCTTAACTTTCAGGTATATGCAAGATTGTTTCTCCTGTTAGTGCATCTGTCCCTGCTGGGGTTGTATTGACAAAGGAGAAAGCAGGGTTCAAGTTCACAACTAGAGTACAACCTCTTCGTCTTGCTGAATGGGATGCTGATGACAAAGTTACCTTTTTTATGAGTGGCAGGTGAGTTTCTAACTCTAACAAATATATCGTTTGACTTGAGTACTTTTTTCTAGGTATTGAGTAAGTGTAACTTTTCAGAAATTACACATTTCGTGATTTCGAGAAAATGGCAAACAAGGTTTTTGCTCGTAGATATTGTAGTGCTGGTTGTCTTCCTGCCTCGTACATGGAAAAAGAATTTTGGAATGAAATTGCTTGCGGAAAGACAGAAACTGTTGAATATGCATGCGATGTTGATGGTAGTGCTTTTTCATCTTCTTCGGGTGATCCTCTTGGAAATAGCAAGTGGAATTTAAAGGTGTTGATTTCATTGCATTATTATTATTTTTAACCTCATTTTCTACTTTAATAGCATCACCAGGAAAATTCATTTTGAAGTGGAGTCATTTGTTGGTGCAATAGCTGATTGAGATTATCTCTTTCTTTCTCTTTGCTAGTGCTCATTTTGCTCCTTTTAATTCCTTGCCGAGTGGTACAACTTGGAATTTGGACATTTTGTTTTACTAGCCTATGTACGCATAAGAATGAACTGTTGTAGTACAGATGTCTGACACAATAAGAAATAACCAGGGATGGAGAGGTAGCCTTAGTGTGAACAGTATAATGTTTGTAGCATAGGACTTCTGCTCATATTCATCCAATTCTTGTTTCGTTATTTTATATATTTGACTGTACTTCCATTGTGTTTAGTACTTTTTTCTTCTACAATCTTGGATTCTTAGACAATTTTTGACTAATAGTGACATAAATATTATTGGGCCAATTTTACAATTATGATTGTCTTTCATGCATCCCATGCAATAAACTGACTAATTCCGGTTCCTCCGTGTATTTTCAGAATCTTTCACGGCTGCCCAAGTCTGTTTTACGTCTTTTGGACACAGTGATTCCGGTGAGATTATGATATTTCCTTGTATTTCAGGCTTACTATTTGTACAAAGTGTGTTCTCCATACTCATATATAGTTTATTCCAGGGAATAACTGACCCAATGCTTTACATTGGAATGCTGTTTAGCATGTTTGCTTGGCATGTGGAAGATCATTATTTGTATAGGTATTTATTCCTGTTTTCAAGTTTAAGCACGCCCAATATATTTCCTTTTATGGCAAAAATGATCTATACCCTTTTATATCTGTTATATTTATTCTGGCAGTATTAATTATCATCACTGTGGGGCATCAAAAACTTGGTATGGGATTCCGGGTCAGGCAGCTTTAAAATTTGAAAAGGTGGTCAGGGAGCATGTCTACACCCGTGATATTCTATCAACTGATGGGGAGGATGGAGCTTTCGATGTCCTTCTTGGAAAAACGACTCTGTTTCCTCCAAATATTCTATTAGAAAATGATGTCCCTGTTTATAAAGCTGTTCAAAAGCCTGGAGAGTTTATCATTACTTTCCCCAGAGCATATCATGCTGGATTCAGTCATGGTAAGATATCTACATCTTCAGCTTCAGGAAGGAAATTGATGAAAAAGTTCTAGTAGGAAATCTCATAAATATTGTTTCTGTATGCATGTCTGTGATACCATAATTACAATTATCATTCTATCTTTTGTGTACGTGTAGGTTTCAACTGTGGTGAGGCTGTGAACTTTGCAATTGGTGATTGGTTCCCCTTGGGGGCTGTAGCCAGCTGGCGTTATGCACATCTGAACAGGATACCTCTGCTTCCTCATGAAGAGCTTTTGTGTAAAGAAGCAATGCTTCTGTATACGAGTCTGGTACTGGAAGATTTGGAGTATTCATCTGCAGACTTGGTCTCTCACCGTTGCATTAAGGTCTCATTTGTGAATCTGATGCGTTTCCAGCATCGTGCTCGCTGGTTGGTTATGAAATCAAGGGCATGCACCGGCATTTCTCCAAATTATCATGGAACCGTTGTGTGCAGCATATGCAAACGTGACTGTTATATTGCCTACCTCAACTGCAATTGTTACTTGCATCCAGTGTGCCTCCGTCATGGTATGTTATACTTGAGAGTTGTTCTTTATGCTAGAAATTGAATTAGTTGGATACTATGATGAACCTTAATTGCCTCTTCCAGATATCGAGTCTCTTGACTTCTCATGTGGGAGCACTTATACACTTTTCTTAAGGGATGACATTGCAGAAATGGAAGCTGCAGCCAAAAAGTTCGAGCAGGAAGAAGGAATATTAAAGGAGGTTCAACAGAAAGCTGAAAGTGATGACTTGTATTCATATCCATTCTCAAAAATGTTTCATAGCGTTCGAGAGAATGGATACTCTCCATACTGTGAGATAAACATGGAGTTGAACCATAAGCCTGCTGCAAAAACTTGGAATCGGTCAGGGAAATCTGAGTATAGTTGTCATATCCAGCCTATACTGAACCAAGAAGCTGCAAATTTTAGATCTGAACATGCAGAAACTTCTGTTTCTGATGCAGCATCAACAATTTGTTCTTTTGTAAAGCCAATCGAAAGCTCATCCACAGCCAATAATGATGTAAGAAGGCTTCCTTACTGTTAAATGTGGTTTTGCTTGATATTCTCTCTCTCTCTCTGTTCACGCGTACATAGTAGATAGTACATACAAACTTCTCCTGAACCAAAAACCAACTACTAAAATATTTACTGTAAGAATTTTCTGAGTGCAAATTTTTGAGTTATCTCTTCCTGAGCAGGTGCGGTGGCAATCTAAATTTAACTTAGGGATTCTTGCTGTTAAAAACTCCCCTGAAGAAGTATCACGTACCACATATGAATCTTCTCAAACTTGTAATGAATGCCCGAGTGCCAATGGCAGCAATTTTCACAGATCAGAGGTTGGGGCTGTAATGAATCAATACAGTGATGATTCTGATTCAGAGATATTTAGGGTTAAGCGTCGTCCATCAAAAGTGGATAAAAGATGTATGAATGATGTCACGTCTTCAACACATACTGAACATCAGGTATTATTTGTGGCATTCTCAGCTTGAATTATGATGTCTGAGATATGATAATAGAGAAATATGTTCTTACATGTATTCATAACTACAGGGGCTCAAGCGATTAAAGAAACTCCAACCTGAAGGAAGATGTGGTCAGTTGATGCTGACAGAGTTTCGTAGAACTGACGAATCTAACCATAAAAGCAGTCATACCTCTAACTATAAAGAAACGTCAGAACGTGGTTCGAAGGACAGGTTTGCCAGAGTTGGCGGTGCCGTTCCTATTTCTATTAAATTTAAGAAGTTGGCCGATGAGGAAGCGAATAGTAGACAGCAAGAGAACTGCAGAAAAGAAAGGTTCCAGCATGAGTGTGGAAAAGCTCCAAGGGAACCACCTCCTATTGAGATGGGGCCAAAACGCCTCAAAGTCAGAGGCCCATCATTTATAGGATCAGATAGATCAGATTGAAGTTTAGATTGCAGCCTTTCCTTTCACACGCATCATCTAGAACCTGACTTTGCTAACTTCATTTATCTCTAATGGGGTTCAAACAACAAGCAGACCAACACGCAGAAGATTGATTCAGGGAGCAGCGGCAAAAATTAACCAAGTTTAGGACACATTTTTATTCTTTGTGCAGGAGACCTCAAACTGGGGAAACCTGTGCTGACGATTCTTGGGGTTCTCAGGCCGGGTATTTTTCCGACTTTGGCAGGGGAGCCTTCATCTTCCTGCAAGGCTGAAATTAGAAATTGGGTTAGTATATTTTTATGTAAATTTTTTTTTCTTTTCTTTTATCAGTCAAATAAATTTTTGTAGGGCCTGTGGGGTATTCTCACTAGTTCTTTGATTTAAATTTCTTGTGTCTGCTGCTTCCGATTCCTCGTATTTTAGGTTCTTTATTCTTTTTGTAGCAATGGACTACTTGAATAAAATGTGCAGAGAAGAAAAATGCCCATTTTTTTCTTCTGAATACAGAATCCATTAGTTTCCTATCAAAG

>CsJMJ5

ATGGCACGTCCAAGAACCGCGCGTAACAGGAGTCCAGTGTCCAAACAAGAGTCATATAAGTTTAAATTTTCTGATGTAGATTGGACACATAAAATTTCAGATTGTCCTGCATATTATCCAACATTGCAAGAGTTTGATGATCCTTTCATTTATCTGCAAAAGATTGCCCCTGAAGCATCCCAATTCGGTATGTTACCGTCAGATATCATGTTGCTTTAATTTGTTTTATTTATGATGTTGTTAGATTCTTATGTAACTGACATGAGAAATGAATATTGTATCTGTAAGCTAATTAGTCCATCAAAATTTCATATCTTAGGCATTTGCAAAATTATATCTCCTGTGAAGGCTTCTGTTTCTGCTGCTGATGTACTAAAAAAGGAGATAAAGGGTTTTGAATTTGGGACTTATATACAGCCTCTTAGGCTTCCTAAATGGAATGCAAATGATACGGGTGTCTTCTTTTCGGGGGAAAGGTAATGCATTGCGGCTTCTTCCGCAAGTTTGGTTCTCTAAACCTACTGCTTAGTTTTGTGGCTTTTTTTTTTTCGTATCTGATATTTATGAGCATTTTGTTTTCTGTACAGAAAACATACCTATGATACCTTTGAGAGTGAGGCAATTAAGATGCTCAAGCGCCAATCTCCTCGTCTTGGGGATCTTCCTCCTTCGTATGTGGAAAAGAAATTTTGGCTTGAAATGACTCATGGAAGGAAAGGCACGGTTGAGTATGGAGTCAATATTGAAGGCAGTGCCTTTTCATCAGATCCTAATGATCAGCTCGGAAAATGCAAATGGCATTTGAAGGTAATGTTGTCTGTGGCAATGACCCATCTTTGATTTTTCATCTTTCCGTTTGCTTACATTTTGTCTCTTTGATTTCAGACTCTACGGGGGCTGCCACAATCTATCTTTCGCTTTCTTGAACATATAATCCCAGTATACTGAGTAAAATTCAATCACAGTTCATTTTTCTTATTTATAGTTTTGAGCAATTTTCTGAATTTTGTCAATGTAAATTCAGGGAATTACTGATCCCATGCTTTACATCGGTATGCTATTCAGTATGTTTGCTTGGCATGTGGAAGATCACTACTTGTACAGGTACATGGGCTATTTCTACATCTTATCTTCTTTGCTTCTATAATTTACACAATGTTCATACTAAGATCCACCACTATTTCAATTTTCAGCATCAATTATCATCACTCTGGTGCTCCCAAAACTTGGTATGGCGTTCCTGGACATCATGCTCTTCAGTTTGAGAAGGTAGCCCGCAATCATGTGTACTCTCGAGACATCTTATCAGCTGCTGGAGAGGATGGAGCTTTCGAAGTGATTGCAGAGAAAACAACAATTTTTCCTCCTAAGATTTTGCTGGACAATGGCGTATCCGTTTACAAGGCTGTGCAGAAGCCAGGGGAGTTTGTCATCACCTTTCCTAGAGTATATCATGCTGGATTTAGTAATGGTAAGATATCCATTGACCATTTCTCACAATCCTTGCTTGCTGCACAATGTTTAACTATTTGCCGATTTATGTTATAAAATTTTCGGTCAAATTTCATTTTTCATTTGTACTATGGCAGGTTTTAACTGCGGTGAGGCTGTGAACTTTGCAATCAGAGATTGGTTTCCCTTCGGAGAAGAGGCTGGCAAGCGTTATGCACGTCTCCACAAGATGGTGATCCTTCCTTATCAAGAACTTCTTTTTAAAGAAGTATCTGAACAAGAAGCCACAGATATCCCATCATCGGTTAAGGCTACAGTTTTACACCATATAAGATCTTTGAACAATACTTTATTTTGCTTAAACAATCTGAAAATGCCATTTGATTATTTACAAAATTCACAAGGATCCTTTGTCTGTGATCTCTGCAAACGCGACTGTTACTTGGCCTTTACTGAGTGCAAGTCATGCCAACGTTACACGTGCCTTTTTCATGGTATGAGCAATGCATACATTTTTACTTTTCCCGAAATTCTAGTCAGCTCAGAAGCTTGTTGAATTTAACACCAATTTTTTTTCCTTGGATTCTCAGAGTTTAAGTCACGACATTGTTCATGTGGGTACAATCGAGTTGTCCTTTTAAGGAAGGATATACAGGAAGTGGAAGTAGTAGTGAAAAAATTTGAGGAAGAAGAAATAATGTCCCGACATTTGAATATAATTTCTTGCATTGGAGAAGGACTTGCCCGGAACAGCTAG

>CsJMJ6

TGTTGCTCAAAAAAACCTAGGCAAAAAGAACTCCACCTCTTCTCTCTCTCTCTTGGGGCTTGGTTCTTTTGAAGGCAAATCTTTTCAAAGTCTCTAGGAGAAATTACCAGGTTCTGTTCTTTTCTGTTTACATTTTTGTTACTAGTATCAGTTTCTTTGCTTTAATGTTAGCTTCAATTCACAAGCAAGGAAAACCCTTATAAGTTCTCCTCACTTCAATTCTTTTTCTAGGGTTTTCATTTGCAAATACCCACTTGAGTGTTTCGTTGTAACTTCAACAATTAATTGAACAATGTCATGAGTTTGAGTTGTAGTCTTAGGTTACTGGGTTTAGTTATGAGATTGTTGTAATTGTGGAAATTTTGTTGTTTTAGATGGGTAATAATAGTAATAATAATGTGGAAATACCCAAATGGCTGCAAGGGTTGCCATTGGCACCTGTGTTTTATCCAACTGATACTGAATTTGCTGACCCAATTGCTTACATATCAAGAATTGAGAAGGAGGCTAGTGCTTTTGGGATATGTAAAATTGTCCCACCGTTGCCAAAACCCTCGAAGAAATATGTTTTTGGTAACTTGAATAAGTCACTCTCAAAGTGCTCGGAATTGGGTTCCGATGTGAATTTGCCTGATGCTGGTACTGTGGCGACAGTGGGTTGTTGTGAACGTGGTAACGAGGGGGAGGCTAGGGCAGTTTTTACAACTAGACACCAGGAGTTGGGGCAGAGTGTGAAAAGAATTAAAGGGGTTGATAATAAAGATAATCTGCAGTTAGGTGCACAAAAGCAAGTATGGCAAAGTGGGGAGGTTTATACCTTGGAACAGTTTGAGTCCAAGTCAAAGGCTTTTGCACGGAGTTTATTGAGTGTGATTAAGGAGGTTTCACCATTAGTTATCGAAGCGTTGTTTTGGAAGGCAGCCTCAGAGAAGCCTGTATATGTGGAGTATGCGAATGATGTGCCCGGGTCAGGTTTTGGAGAACCGGAGGGTCAATTTCGCTATTTTCATAGACGGAGGAGAAAGGTGACGTCTTGGAAATCATATCGGAACAGAGGAAAGGCTGATGAAAAGAATATTGAACTAGAAAGTGCCAGAAATTGCCATAATGATCAGATTACACATTCTTGTGATAAGAACGATTTAGAGACCCCCACCTCATCTACGCCTTCATCAACTCTTCCATTTGATGAAAATTCACGATCTTCTAGACGAAAGAGTGTAACTGGTAGTAATGATGTGGAAGGAACGGCTGGTTGGAAGCTCTCAAACAGCCCTTGGAATCTGCAAGTCATTTCACGCTCACCTGGATCACTGACACGTTTCATGCCAGATGATATCCCAGGTGTTACTTCTCCCATGGTTTATATTGGTATGCTGTTCAGCTGGTTTGCCTGGCATGTTGAAGATCATGAGCTTCACAGCATGAATTTCCTTCACACTGGTGCTCCAAAGACTTGGTATGCCATCCCGGGAGACTATGCATTTACTTTTGAGGAAGTTATTCGCAATGAGGCTTATGGTGGTGACATTGACCGATTAGGTATGTCAACTTTTAATTCATTTTACTGTCAGATGTTGCATAATAAATCTCTGTGTTATAGAAAGATGGAAAAAAAAATCTAGCATTTCTACAACACTGGGTTTATCTTCACTGGACTGGGGAAAAAAAGAAAAAATCTGCAACTCCATGTTCTTGGTGTGTCAAATTAGGTTACCCCCTGATAGATGGACCTCATCTTGTTGGGATAACAATTTTGGACCTGGTCAGTATTTTGTGTTTTATGTGGGTAGATAAATTTTGATAAATCCTAAACTTACAGTCCTATTTTAGTTCATTGATTAATATGCTTACAACAGTCTTACATTATTATATAGACTAAAGAATTGTCGTTGCTTTAGGCATATTTCTCAGTCTGACCATTGACCAGTGGCTTTTTGGTAGAATTCTTCTTTTTATGTTGGAATTTGTTAATTCCATATCATTTTGGTAGCTAAATCCCAGTTTCCTGTATTGCCAGCTGCTCTCTCATTACTGGGTGAGAAGACAACTCTCATATCACCCGAAGTAATTGCTGCATCGGGCATTCCTTGTTGTAGGTGAGTTGCATTATTATTATTTTTCTCAAAATTAATGGCAAATTCGTCACTTTCTGATGTTCTTTTAGCATTTATGAGTTCATGGATGTGATCAATTTAGTTTGCCATGCATAAAACTGGCTAGGGTTGATTTGTGCTGATAGTGTTGTTGAAGTTCTACTTATGCCTATTATGGTTTCTCTTTTAGGTTAGTTCAGAATCCTGGTGAATTTGTTGTGACTTTTCCACGGGCTTACCATGCAGGATTCAGCCACGGTAATTGCTTTACAGTTTGGTTGCACATTTACAAGATCATTTCGACATCTTCGCTGTTATGACTCCCCAAATGGCAATTGGTATATGGTCAAATGGTTTGTTAATACAAGCAAATTGGAATATACAAATCTTAGAATATTTGGGTTGATGCATGGTTTGTTGAATGTCAATATATGCATGGTTTGTTGAATGTCAATATATGTTCCTGGTTTGTTGAATGTCAATATATGTTCCAAAAAAAAAAAGACGTGAAGGGTTTAGCTCAAATGTTCAGTCTTTACATGGTTGTAAACTAAGATTGGTAAGATTCCAGTCCTTTTCGTAGGAACCTAAATCATTAACTATATACGTGAGGAGTTTGTATAAATAAGATTTTGTTTGTGGCCGTTACCACATCCATGTGTCAGCTATATGGGTGCTGAAAAAGTAGTCTGAAAGTTTTTCACTCTTTACCTCGGAGTACATAGTGTTATAGCACAAATTTCAAAGTTGGCATTTCACAAGTTGATCATACGAGAACAATATTGTTAAACTTTAATTTCATCAGAAGAAGTAAAATAGAGTAGCAATATTAAGTAGACAATAGTCCTGACTGAAAGAGCTGCTGGCTTCTGATTTTATTTATTTAAGAATTGCATGCAAGTTTAAATTTCAACGAGAGGTTATGTTCTCATCTACTAATAATACTGTAAACGTTTACATGATTAGGTGATGTTGATTTTACATGTTCATTTAGAGAATAAAATTGTTGTCACTTCCACAACTGTCATTAAATTAATCTTTATCCTGCCCTTAAAATTTTTTGAATTGAATGTATTGTTGCTGAGATGCTTTCCATACGCTGCAGCAGGAAGGATTTTCCTGAAGTTTACATTTCACTCTTTCTACATCATTAATTTTTTCAAGGATTACATAGCTTTCTTTCTCCAACCTAGGTTTTAATTGTGGGGAAGCTGCTAACTTTGGAACTCCACAGTGGCTTATGGTGGCTAAGGAAGCTGCAGTTCGAAGAGCTGCTATGAATTATCTTCCTATGCTTTCCCATCAGCAGCTGCTATACCTGTTGACCATGTCATTTATTTCAAGGTGAGTTTGTATCCTACATGCATGCTTTATAATTGATATGTTCCTCAGTTGAGTAATATTGATTGAAATTATAATTGATATGTTCCCAAGTTGAGTAAATATTGAGTGAAATTATCACATTTTCATAGCTTCAGCGACTTGCTCTATAAAGTTTTGTACATTCGTAATTTTCATTATTGTCCTTTATCGAATGTAATGAATGGTTACCAGCCTACCCCTATACCTGATGAATTGCTAAAACTCTTTTTTAAAAAAAAAAAAATATTATGGGATTTGGTTGTGTGTTGGGTCCAGAATGTCTTTCAAATGATTTCTGGCTTGGCAGGCTGAGAAGGAAGTTATGGCTGATTTAGAAAGAACAATCTTTATAGACGAATAGTAATCAGGTCCTGATTCACGCTCACGAAGACTTCTTTCTGTGCATAAATAGCTTAGTCCATATGTATTGCCTGTGCCTATGTAATTGTTGACTTTATTTTTCTTTTTTGGGGCTATTTGGCTATTCATTGCTTTAATCTTTATGTATCTTTAATTCTTAAATTACTTTAGTTACATGGTTTCAATGGTAATTGAGTTAAATTATTTTGAGTTACTGTAGACTGCACTCATCTTCCTTTGAAAGAAATTGCAATTTATGTTGAACTGCACTAGAGCTCAATGGATCTAGTTCTGTTGAGAGTGTTTTCTACAATACAGGACGTTATGGATTCTGAGAAGTTTGATCTCTGAATTAGTCAAATCATTCTGTGGTTGTGCCCTTCTTATTGTGTCTATAAAGATATAATTTGACACATCTTATTAATTGTTTCTCATTTTTCTTGTACTGTATTTGTTCTGATGGCAGGGTGCCAAGATCCTTATTGCCAGGTGCTCGGAGTTCACGGTTGAGAGATCGTCAGAAGGAAGAAAGAGAGCTGTTAGTGAAAAAGGCTTTTGTAGAAGATATTTTAAAAGAAAACAACATATTGTCTGTTCTTCTTGGACGACAATCAACTTTTAATGCTGTACTATGGAATGCTGATTTGTTACCATGTCAAAGCAAAGAGTCTCAAATGCCCTCTGCAAATGAAACGGTTTCTACCACACCTGGAGAAACTGTTCCCAATAATCCTTATGAGAAACACAATGATCACAATAATCTGCTTGATGAGATGAACGTGTATATGGAAGCTCTAAATGATCCGTATATGGGTGATGATGACATCTCACGGGATTTTCACATTGATTCTGGAGCACTGGCATGCGTAGCTTGTGGGATTCTAGGTTTTCCATTTATGTCTGTGGTACAACTGTCTGAGAGGGCATCGATTGAACTGCTTGCTGATTTGGTTAAAGAAGGACCTGGAGTTTCAGAATTAAAAAATACTCATCACCATACAAACCTTGATGGCTCTGTAAAGAGCTCTGTTTCAGGTATTCTGCCACCTACTTTATCCGATAATTTGCCATCACTCTCAGCTATTTCCTGCCTTTTCTTCTATGATTCTAGCATCAGTTTAGAATTTAGGACTACAAATTTCTTGCTAAAATTAAAGTTTATCAACCTGATACTCTACACTTCAGGAAAGATTTGAAAAAAATAAAATTGAAAGAAAAATGATGAATCCTGTGTATGTTTAAATCTAATATTGTTAGCCACTGTAGAATAGTTTAAATCTTAGATTCTGCAGATCTTTTGGACATTTGGAGGTGTTCTGCATCAGTTTGTCAGTGAAATAAATAATAATTAATTGCTGTGGTGTAAAGAAGTGCCTGTATGATTCTGGCAATCATTTTACTAATCTTTCATTTTTGCAATTTTGGGTGTTCACTTTCTAGATTAAATTGAGTATGAAACTAGGGTGTTAATGTTTGAATGATTTTTGTATTTATTTAATGTTCCAATATGTCTATCTGATTTAGGACTTTCAATCTGCTAATATTGTTAGTTACCATACAGAACTTGGTCAAAGAAAGGTCATGATTCTTTAATAGTAAATATAGCGTCTGTCTTGATCATTATTCTTATAATTTAAACTTGTACTATCATATGGAGATGCACGAGCTGATGTATCAAATTTAATTACACACGAAGAAATCTTTTTGGGATGCAAGGGGTGTAAATTTTTCATTTGTTGGCGAGAATTAAAGTTTATGAGACACAATCCTTAGCCCTAAAATAATTCTGATATCTCTTGATATGAGCAGATGAAAATAGGATTAATTAACATCATCACCAACTGGACTTCAAAGTGGCTAAATAGCATGCCTTGTGTTTTATTTTTGCAGATGATCTTTCCCTTGTTCCTGATATTTCTCTGCTTCAAAAGGATTTGTCGGTACCCTCGATAACCAAGTCCAGTAGGATATGGAACACTTCTAATAAGTATTTGAGACCCCGGATTTTCTGCCTCGAGCACGCTGCTCAGATTGAGGAGATATTGCAGTCTAAAGGTGGAGCAGAGATCCTTGTCATTTGTCATTCAGGTGAGGCTGCCTTTAAGTCTATAGTTGACTGGTTCAAAAATTGACTGACTTTACGAGTTTTCCCCTTAACAGTTTTGTTTCCAAAGGACAGGCTAGATTTTGCTGAATTATCCAAATTTTAATTTGTTTTATTTGTGGAAAGAGGCATTCAGTCGAAATCAATTCCCTTTTTTTGTGTCACAATCACATCATGCCGTTAGTTAACCCATCATTATCACTCCAAGACCAACGATAGCACCAATTCCTGGCTTTCATATTTTCTATTGATTGTGAACTGAGTTTGTAACTTTAATAATTAATTCTTGTAGACTATCAGAAAATAAAAGCACATGCAGCAGCTGTTGCTGAGGAAATTGGCAGCCCTTTCAATTATATTGATGTTCCCCTAGATGCTGCATCTGAGGAAGATCTGCATTTGATTGATCTTGCAATTGATGATGGAGAACTTGATGAATGTAGAGAAGACTGGACCTCAAAACTGGGTATCAACTTACGACATTGCGTCAAGGTTAGAAAGAACTCACCATCTATGCGTGTTCAGCATGCATTGTCACTGGGTGATTTGTTCTCCGAGAAAAGTCTTAGCTCAGATTTCTCCAAAATCAAATGGCAGTTTAGAAGATCTCGCTCAAAAATTAAGCTTTATGGCCGAGCCCACTCTAAACCATGCCAGAACATTGAGATAAAGAAAGATGAAGTAACAGGGCGAAAGTTAGATGGCGCCACTGTTAAAAAAGAAGAAAAACTTATTCAGTATTCAAGAAGGAAATTTAAGCAGAAACCAGATTTGTCAACTGGGGCATGTGGGGACCAGGTACACCCTAGAGAGCTCCTGCCAGAAGTTTCTGCTGCCACTTGTGACCATCTTGATGGGCATAATAGAAGTGATTTTGAAATTAACCCTGATGGTACTGGGAATAGTGGAAGCATTTCTGCTGGGTCCATTCACTCTCCTATTGGGATGTCCGAAGGGCTGCATGACATCCCTGTCCGTGAAGCAACTAGCAATTTGAGTTTGAATTACTCGCCTTCACGGGTGGCAGATTCACTTGCAACTGCTACTTTAGTGGTAGATAGCATTGTGCAGAATGATACTGAATCAATGAAGGAATTAAATATCGAGGGTGATATTTTTCACATGGCAACATGCAAAAGTGCCGAGATGCAACAAAACAGTGGCACTGATGTAACTAGTGAGGAAACTGAAATTTCTCACCATACCGTTGCATCTAATGAGGGATCTATAATAATGAGATCGGATCAAATTACAGAAAGTATGACCATCAAGAATGAGAAATGTAATCTGGCTTCTGAGGGACATTGTAGGAAAGTGGCTGATAAGGATGTTTTGATGATTGAGGTTTCCGGTCTTGCTAACTCTGCAAGTTTTCGTGTTGCTTCTTCACCTCTGAGGAGTCTTGATGCACAGATTGAGAACTTAGCTCCGGATAATTCCTGTATGATTAGTGAAGCATGTGACCATCTGATTTCTGACAATGAAGTGCGGCAAAATGTGCAATCTACTAATGGAGGCAATGATGTGGAACCCATTTCATGTGATCATAAGCTGATAGATGAGCCTCCTGCCTCAACAGGTGAAAGTTGTGAGGATATGAGAGAAATAAGCACTGCAGAGTCTTTACAGGATAATTTGCAGCATGAGAGAAATATTGGAAATGGATCTAATGAGGAACTTGTTTCCAGTTCTGTTACAATGATGATTCAGCCTACTTCCGCTCCAATGGAAATTTCAGAAGTTCCAAGCAAGGAATGTGCTGCAGCAGACTTGCTCAATGTTGGGACCAAACAGAAACTTATCTCAAGTTGTGTTTCGCGGATGGAAGTAGATCAGCCTTCCCCACTCAAGGTTGGAGGATGTTCTGAAGTTCCAATAGAAATTTGTACCAAAGAAGACTCAGGTGCTGATATGACGTTAGACCCTAGAACACGGCTGCAAAATCATACTACTGCTGAAGCTATTATGGATGAGCTTGTTTGTAATTCTAGTGCACAACTGGAAGAAAATGAGCGTATTCCTACCTCCATAGCAGCATGTTCTGAAGAATCAAATGGTATATTTGCTGAAGAAAAAATGGATTTTGAAATGACAATAGGTACCCAGACAAAAAATGCTGCTAGCGAGGAACCAAAACCTACTTCTTTGATCCCAATAGATCAGCCTATCCCTGCCGTAATCCGAAAGTACTCTAGAACTCGAAGAGAGTCGTACTCTGCTGAAAAATTTTGCAATGGCAATGAAGCATATTCATCAAAAGACAACAAAGAGAGGGGATGCAATGAGCCCAATTTGGAAGATCCCAGCTCTAGCGCTGGAAAAGGAAGAAAAAGGAATAGAGAATTGGAGCGGTTGACAGAAAACAAGTTAAATGGCAGTGGGTTTATCAGGAGTCCTTGTGAAGGATTGAGGTCACGGGCTGGGAAAGATGCAGCTAATACAAGTGAAGTTGACATCAGAAAGATTGCTGAGAAGCGTGCAACAAAGACAATGAGAAACCGTGAAAGTGTTCCAGCGCCTTGCCAGGATAAGAAAAAGATCTTAAAGGGGCATCACAGGTGTGATCTAGATGGCTGCCGCATGAGTTTTGAGACGAAGAGAGAACTATCCTTGCACAAACGCAACCGCTGCCCGCACGAAGGGTGTGGGAAGAGGTTTAGCTCCCACAAATATGCAATAATTCATCAGCGTGTTCATGATGATGAGAGGCCGCTCAAGTGCCCATGGAAAGGTTGCTCTATGTCATTCAAGTGGGCTTGGGCTAGGACTGAGCATATACGGGTGCACACAGGCGAGCGGCCATACAAATGCAAGTTTGAGGGTTGTGGCCTTTCTTTCAGGTTTGTATCTGATATTAGTCGACATAGACGGAAAACAGGGCATTATGAAAACTTATCTGCCTGAAGATATCCTGTAAATTACGCTGGTATTGTAAAATATGGCCACGGGAAATGAAGATTCTGGGGCCAAAGAGGCATTCAGAATCATGTACTATATACAGAACTCTTTTTGAGTGTTGTTTCTTAAATATAGTTTAGGGGTTAGGCCGATCACTTTCTTAGGGCGCATTCTTCAAATTATTTGAATGCAGTGGTAGTTGAAAAGCCTTCTCTTTTTTATCTTTCTGGAATTTTTCATGTTATTTTTTCGTTTTTGGATAAGGAAGGGAAGGTTTCCAGAAAGATTTGTTCAAATCATTTTAGCTTCATAGATTGATTGTCTAGATTGGTCTTCATTTTTAAGCAGGGAATGAAGAAAGGATATGGATGCCGACCGTCATTCATATTGTATCCAAGGGTTGGGAAGATGTCTCTTTTTTTTTTTTTTCAATTGGAATGTGGCAATGTGGATTGTGGAAGATGGCATAATGAGTCTGTTGTAATTTTGACAATGGGTCAAAAGCAGGACATCAATTCTAAAACAATGTCTTCCTCTTTGTCCTTTGACATTTTTTAAATGATTTCCTTCGTGTTTTGTTGACAAAATTGAATATATAAGCACCCCTTTA

>CsJMJ7

CAGAGGACGAAAAATACAACAAACCAACACTTAACATGTAAATAGAATAAAATAATAATCCAAAACAAAAAGGAAAATGAAGCAGCAGCAGCAGCACCAAAGAAGAATCTGAACAGCTTCTCCCTCTTTCTAGTCTCCATAATGGAGACTTCCTTATGATTAGAACCCCACTATTTTTTTTTCTTTTCTTTTTTCTTTTTTTTTGTTTTTTGGTTTTAAATATCCACTGAACCTCTAATCAATGGCGGAGCCAATACAGCAACAAGATATATTGCCATGGCTGAAAACCCTACCTGTGGCTCCAGAATTTCACCCGACTTTGGCCGAGTTTCAAGATCCAATTGCTTACATTTTCAAGATCGAAAAGGAAGCCTCTCAGTACGGCATCTGCAAAATCGTTCCCCCGGTCCCACCGCCTCCCAAAAAAACCGCCATCACGTTCCTCAATCGCTCCCTAGCTCAGCGCGCGGCAGCAACTGGCGGTGCTACTTCCAGCTCTGGTCCTACATTTACCACTCGGCAGCAGCAGATCGGCTTCTGTCCTCGAAAACCGCGTCCGGTTCAGAAACCGGTCTGGCAGAGTGGCGAGTACTACACTTTCCAGGAATTTGAAACCAAAGCCAAGAATTTCGAGAAGAGTTATTTGAAGAAATGCGGCAACAAGAAGGCTGCTCTTTCTGCTTTGGAAATCGAGAGCTTGTACTGGAAGGCCAGCGTGGACAAACCGTTTTCTGTTGAATATGCTAACGACATGCCGGGCTCGGCGTTTGTGCCTGTGCGGAAAATCAGGGAGGCGGTTGGGGAAGGGGTGACGGTGGGCGAGACGCCCTGGAATATGAGGGGAGTGTCGAGGGCTAAAGGGTCACTGTTGAGGTTTATGAAGGAGGAGATTCCGGGTGTGACTTCGCCCATGGTTTATATAGCCATGCTGTTTAGCTGGTTTGCTTGGCATGCCGAGGATCATGACTTGCATAGCTTGAATTACCTGCATATGGGGGCTAGTAAGACGTGGTATGGGGTGCCCATGGAAGCGGCCAATGCTTTTGAGGAGGTGGTTAGAGTTCACGGTTATGGAGAAGAGATTAATCCTTTGGGTAAGTAGTAATTCTGACTCCCCTCTTTTTACTATTTAATGTTGAAGTTAGTTTCTTTAGTACATTGTGGAATGTAGTGCAATTGTTGGTAGTTTAAGTTGGAAATTGCTTGGAATACAAGTTCTGTGTATGTAGAATTGAAACATGAGGTTGAGGTTTGGTAGGGACTTAGGATTGGCGGGTTTTAGAGGCTTCTAGGAGTGTATTTGAGGTATTATGATGGGCGCTGCGATTTGGGTTTCTAATCATAATAACAGCTGATGAGAGCCAGGATCACATGATTGGACTAGTGGCTTTTAATTTCTCCACGAGGTTCAACTTGTTTAATATGCAATTCTTGGGTTTGGTTCAACTGAAAAGCAACTTAGCTAGTAATAATCAAGGGGGTGATACTTGAATTTTATCTTTATCTATTTGTTACTTCATGCTGTTCTACTTAACTTTATTTATGTTGTTTTCTATCAGTTACATTTGCTACACTTGGTGAGAAAACAACAATGATATCACCTGAAGTGTTTGTTGGTGCGGGGGTTCCATGCTGCAGGTGAGTCTTTCTCTCTGTTCTTTTATACTTGTCAATGCTATCTTTTGACTCTGAACTAATTTCATGTCTTCATTTAAGGCATGATGCATCTATTTATTCTTAACTCATCAGTTTCTTTAAATCAGGTTGGTGCAAAATGCTGGAGAGTTCGTTGTGACTTTCCCAAGAGCCTATCATATGGGATTCAGTCATGGTAAGGCCCTTGAGTTTGATGATGTTACTTTTAGGTCAGAGTAGTTGGTATTACTGTTATGAACTGGAGAAACTTATTTCTTCTGTCTGAATTTTGTCCACTAGGATTTAATTGTGGGGAGGCAGCCAATATTGCAACCCCTGAATGGTTAAACATTGCTAAAGATGCTGCTATTCGAAGAGCTTCAATCAATTATCCACCTATGGTTTCTCATTTCCAGCTACTCTATGATCTTGCAATAGCAATGCATTCAAGGTATTTGCTGTTATGTTCTTAGGATTATCCTAGTTGTATACATGAGTTGATTGGCTTCATCATTTTGCTTATCAGCTGTTAAATCCCATTTGATCTTTTTAAACTGAAATAAATTGGACACAAGAGTCACTGGAGATTTCGATATACTTTGCTACACTCTCTGGAGCAGATTTTGCATTTATTTGAACTTGCATTTGAAATTTAATTGTTCTTTTTCCTTCTCATTGTCACTGACACTTTTTTCTTTAGTATTCGTTAAAAATTCTACCGAAACTATCTTCTTTTTGTTGACAGTTGGAAGTACAGTAAATATACATCTTAATTAGTATTCACAGTCTCTCTCAATTTTCTTATTTTCTCAAATGCAGTATTCCTGTGGCAGTCAGTGCTAAACCACGGAGTTCTCGACTAAAGGATAAGAATAAAGACGAGGGAGAAACTTTGGTTAAAGAATTATTTGTGCAGGATGTAGCACAGAATAATGAATTGCTTCACGTTCTTGGACAAGGATCTCCAATAGTACTTCTTCCTCAAAGTTCTTCGGGGGCTTTAGGAGCAAACCCCTGGATCCCCCTTGGTCTATGCAGTTACAGAGAAGCAATAAAATCCTCGGGTGGTTTAGTTTCTAACGATATCATGGTTGGCAAGAATAATGGAATCAATCCTGTGAAGGGTTACTGTTCTGTGAAAGGAAAATTTGCTTCTTTATATGCAAGAAATTCCTCATTGAGTGAAACTGATAACATAAGAACTTGGAATTCTCAGATACTATCCACAGATACTGAAAGACAGAATACTGTTCAAGGTGATCAGTCGTCCGATCAGAGGTTGTTTTCTTGTGTCACATGTGGGATCCTGAGCTTTGCTTGTGTAGCTGTCATTCAACCTAGAGAACCAACCGCTAGATACCTTATGTCGGCTGATTGTAGCTTCTTCAATGATTGGATTGTTGGTTCTGGAGTCAGTGGTGCATTTCGTGCTGCTGGAGAGGATGTAATAGCTTCAGAGCACAATTCTCGTTCAAGTAGGATATTGCCATTCTACTTTTGTTTATTTGCCTCGTCGAGTGATCTTATGTAGTGGTTGGCATAGATATTTGCTGCCCCCCTATTATATATCTTGTCAGTAATTAGATTGTGTCCACACCTGCATTCTACAGAGGGCATGGCCTGCATGGATGAGTTTCTTGTGCTCAACTGGCAGGATCCCTTGGGTTTGATTCTGTTATTATTATGATGTTTATCCGCTTTGCAATAGCTACTTTGTCTCTTCCAGGTGCATGGGTCCTTGCCTGTTTCATGCTTAAGCTTTCAGGTCATGTGATAACTTTAACATGAGGCAGACTAAGTTTTAGTGACTGGTGATTCCATGGTTGTGAGGGAGTTAAATCAGACCTTTGATGTTGATGACCTCCTTGTTTGGTTGGTTGAGAGTGTCTTGATGTTTTGTCTCTGTGATAACTGATAACGTGGTTAAGGCATGATCAGAGCTGATCTTGTTTATGTCTCTTATTTTCTTACTGATGGTTGCTCATCTCTGTTCCTCTGTCCACAGATGAAACCATCTGTTTTCTCTTCTCTTTTGATCTTTCATATGGCTATAGCATAGATCCTTTAACTTTCCATACTGAAACGTAGTGTATTGTTTGCAATTCAGGTTTTGATCCTGTTTAGATATTACATAGATGACTTTATAGGTTTATTAGGCCAATGAAATTGCCTCTGTTTCATGGTGGAATATGACAATTCTAGGAGAACGGCCATTAATGAAAGCACATGAGGTGTAACTTAAGGAAAGGAAGAGTGGACTGGGATAAAGTGCCTAATAATATTGTTGTATCAACCAATTTTTTTTTAAAAAAAATTGCTTCTTGGTAAACATGAGATCTTTTTAGTGTGAGCAGAGTGGAGAGTATTTAACAGTCATGTGTATTAGGTAGTGGATATAACTTGATCCCCATATGTCCCCATATGAAGATTGCATACAATATCTCTTTTCAGCACTTGCTTACATAATTATAAAAGATCTGTCGTATCAGTGGAAATATGATGACTAATTCTTCTGTTAAAATTATATTTATTAATATCTCATGACTATATCTAGCTTTACTGGTGATTTTTTTATGAGGCTTCAATACCCTATTAAAACATAGTACTCCTTATGGATGGCTGTTGTTGATGCAATTTTCCTCTTTGGCAAATGATGACTCACTGCCTTGCACATTAGATCTTTTAACTCTGGATCCGAGCAGCATACTTTACAGTTAACCTAAATAATACTCAATTTTTGTTCCCTTATTGTGCTTTTCCTAATTATGTTGAGCTACTGTTCCCGTTTCCAACATTTGGTTATTTTTGTATAAAATACAGGATGGATCGGAAAGAGTGGTCGGAATAGTTTATATGATGTCCCTGTTCAATCAGCTAACCAAATTCAAGCAGTGGATCAAAGTAATGAAACAATTTCAGATAGAGAAACAAAAGGAGATACCTCTGCTCTTAACTTACTAGCTATAACTTATGGAAATTCATCTGACTCTGAGGAAGAACAGGTTGAACCAAATGTTCCCATGTGTGATGATAAAGAAACTAAATTGACGGAGTGTTTGTTAGAAAGAAAATATCAGCAAAACTTTCATGCTGCTGCTGCTGCTGCAGGGAGTCAAGATCTCTCCTTCATAAGCCTTGATTGCGAGGATGAAGCTTCGCTTCAGATCAGTAATGTGCAACCTGAATTTAGAAGAGATTACTTGAATGATAAAAATCCTGAAATGTCTGAATGCTCCGTAGAGTTTGAAACTGATAAACACGATTGCTCTAAACCTAATGGTTTTGATGGTTGTTTTGGAGATCCAATAGCAGCATCTTATGCTTCAAAATGCGCTCCAGTCATCCATGGTGGTGAAAATGTGGAGTTTAGCAAGGCCATTGTACCAGTGATGAATGCTGAAATGTCCTTTGCTCCAAGATCTGATGAAGATTCTTCTCGAATGCATGTCTTCTGTCTTGAACATGCTGTGGAAGTAGAGCAACAACTTCGTCCTATTGGAGGGGTGGATATATTTCTTCTTTGTCATCCAGGTATGTTGTCATTGAGCAATCATTCATGCCTATCATGGGTATCTCCTTTTTTCTGTCGTTTTATTGTTTTACTTATTACTACTGTTATTTTTCATTTATTCCTGAACAAATAAATAACTATCATACACGAATTGTTGGAAGTTGAATATTGTATATCAGTTCTTTCATAGTTTCATAGTGATACTGTAATCTGGCGATTACAGTCAATGTTCTTATCGCACTTATACCAGGGAAAAATTTGTAGTTCTCATTGCATGACGTGGAGTGACTGAAGGCATGAATCTATACTGATATGTGGAATAGTAGCTATCGCATGATTGAGTGTCAACTATGCTATACATTAGTAAAAAGACACCATTTGTAAATGCTGTGGATTGTGCATGAATAGTGCTGACCAAGGTAATAATTTTGATGCTTTTTACTAATACCATTTTTAGCCTTCCCAAATTTTTGTGGACTCTAAAATTTTGAGTGTTTTAAGGGAGAATGAGCATCATGCCTCCTAAAGTGGTGATGGTGCTTAAAAAAAAGATTCAGCATACTGTGCATAGTTATCTAGCTGCCTAATGTAGTAATGTTTGGTTAGTATTGAGTGCTCTGCTGCAAAATTATCTGCCCTCGGCCATACTCTTTACTTTAAATGTTTCTTTTTGTTGGACAAAATTGCAGCCATGTTGAAACTGACATCTCAGGAAACCGTGATCATTTTATTTTTTCAGTCATTATATTACTCTCTTCAGTAGTGAAGAGAGTTTCTTCTTCCTAACAAGCTTGTGCATGTTTTCTGTGTTACACTCTTGTAAAAATATAGTTCTATTGAGAGCAGTTGCAGCCCCCTAATAATTCATAATTCTTTCTAAAAACTTGCAGATTATCCCAAGATGGTGGCTGAAGCGAAGTTAGTGGCTGAAGAATTGGGAATTGATTCTCTCTGTGATGAAATCTCATTCAGGGTTGCTACCAAAGAAGACGAGAAGAGAATCCACTTGTCTCTGGATAGTGAAGATGCAATACCTGGTAATGGGGACTGGGCTGTAAAGTTAGGAATCAATCTTTTCTACAGTGCCAACCTCAGCCGCTCTCCTCTTTACAGTAAGCAGATGCCATACAATTCTATAATATACAATGCTTTTGGCCGTAGTTCTCCTGCTAGCTCGCCCAACAAATATGATAACGGGAGACGTCCTGCCAGACAGAGAAAAGTAGTAGCAGGGAAATGGTGTGGGAGAGTTTGGATGTCAAATCAGGCTCATCCTTTTCTAGTGCAAAAGGATCCTGAGGAACAAGAGCTAGAAAGGAGCTTTCATGCTTGGACAACACCAGATGAAAATTTTGAAAGAAAACCAGAAAGTATTTGCCAAACTCCCTCGACCTTGGTGACTAGAAAGTACAGTAGGAAGAGGAAAATGGTAGCAGAGAGTGTGTCAACCAAGAAAGCCAAATGTATCGATACAGAAGATGCAGGTTCAAAATATTCACTGGAGGGTGATACTTGTATCCAGCAAAGGAGGATTCTTAGAAACAAGCCAGCCAAATTAATGGAAAAAGAAGATGTGGACTTGCCCGATTCATCAGAAGTTAGTTCTTATCAACAGAAGAGGAGTGTTTCTAGAAGAAAACAAGCCAAATGTATTCAGAGGGAAGTTGGAGATTCAAATGATGCATTGCCAGGCAGTTCTCTTAAACAGTATAGGAGGATTCCTAAGAGGAAGCATGCCAAATGTATTGGTAGGGAAGATGCAGTTTTGGATGATTTAACTGATGATAGCTCCCTTAAGCAGTACAGGAGGATTCCTAGGAGCAAGCTAGCCAAACATGTTGCGAGAGAGGATGAAGTTTCAGATAATTCGCTGCGAGGTACTTCTGATAGGCAGCATACAAGCATTCCAAAAGGCAAGGAATTCACATGCATTGATAGAGATGATGCAATTTCAGATGATTCACTGCAGGATAATTCTCGTCAGTTGCAGTTTAACAGGGTTCCAAAAAGCAAGCAAGCTGAATGGATTGAGAGAGAAGATGCAGCTTCTGATGATTCATTGGAGGATTATCCTCATCGCTTGCAGCATAAAAAAATTCCAAATATCAAGCAACCCAAATGTATTGAGAGTGAAGATCCGGTTTTAGATAATTTACTGGAGAAAAATACTCTTCGGTTGCATCATCAGAGGATTCTAAAAAGCAAGCGAGCGAAGTGTGTTGAGAGAGAAGATGCAGTTTCAGATGATTTACTGGAAGATAGTTCTCATATGTTGCCTAGGAGATCTCAAAAAAGCAAGCAAGCCAAATGGATGGTGAGAGAGGATGCTGTTTCAGATGATTCACTGGAAGATAATTCTCGTCAGTTGTACCAGAGGATTCCAAAAAGTAAGCCATCCAAGTGGATTGAGAGAGAAGATGCTGTTTCAGATGATTCACTGGAGGATAATTCTCATCAGCAACATAAGAGGATTCCTAGGGACAAGCAATCTAAATGCACTGAGATGGAAGATACTGTTTTCTATGATTCCGCTGAGGATAACATTCAGCAGTTTAGGAGAATTGCTAGAAAGAGGGCCAATTTTACTGAAAGGGAAGATGCAGTCTTGTATGATTTACTAGAGAATAAATCTCATAGAAGACATTGTAGGACTCTCAGGAGCAAACAACTGAGAACCGAAACACTTCGGAAAATGAAACAGCAAACCCCTTCACATATGAAGCCCGGGAAAAGTAGATTAACAAAACAAGAGACCTCTCGGCTAGTGAAACAAGTGACTTCTCGGCAGCATAGTGTTAAAAGTGAACAGAATGCCAAACTGTTTGATTCAGTTGTTGAACAGGAGCTAGAAGGCGGACCTAGCACTCGACTTAGGAAAAGAATCCCAAAGCCTCAGAAAGAGTTTGAAACCAAACCAAAGGAGAAAAACCCAGCCGCCAAAAAGAAGGTAAAAAATGCTTCAGTGGTGAAGGCTCCTGCTGGTTTGAATAACGCAAAAATTAAGGATGAGGAAGCAGGATACCATTGTGACATGGAGGGTTGCACGATGAGTTTCGGCACAAAACAAGAGTTGGTGCTGCACAAGAAAAACATCTGTCCAGTCAAGGGATGTGGGAAGAAGTTTTTCTCACACAAATACTTAGTGCAGCACCGTCGTGTTCATTTGGATGATCGTCCCCTTAAATGCCCCTGGAAGGGATGCAAGATGACTTTCAAGTGGGCATGGGCGCGTACTGAGCACATAAGGGTTCACACAGGTGCTCGACCTTATGTGTGTGCTGAGCCAGGCTGTGGCCAGACATTCCGGTTTGTATCAGATTTCAGTCGTCACAAGCGGAAGACCGGACATTCTGCAAAGAAGAGCAGAGGGTAGTAAGTGTAAAGGAAGAAGATTGCTTGTTAACATTTGCTAATGAACCTGTTGTTGAAATTTAGAGGGTAGGGCTAAGATAGGTTTAGGATGTTCCTTGTCCTGTAACTGTCAATTGTTAGTCAATTGTCATTCAGAGCAACTGTTAAATTTGTGTTGCCCTGGGGCTTCATTGGGCCAATTTATTTATGAATTGCTCGAATCTTGAAGCAGATTAACCTCTATTCAATTTCCCTTCAACATCTCTTTAATCTCTTTGAATGAAATTCTTTTCAATACTGAAGAGAATTTTTTGCAGTATTTCAATTTATTCGCCG

>CsJMJ8
[truncated: 376,373 more chars]
