# Supplementary material for: Genome-wide identification of sweet orange (Citrus sinensis) histone modification gene families and their expression analysis during the fruit development and fruit-blue mold infection process
Source: Front Plant Sci. 2015 Aug 5;6:607. doi: 10.3389/fpls.2015.00607 (PMC4525380; doi:10.3389/fpls.2015.00607)
Supplement: Supplementary file 4 [file Image_1.PDF]

**PRMTs:**

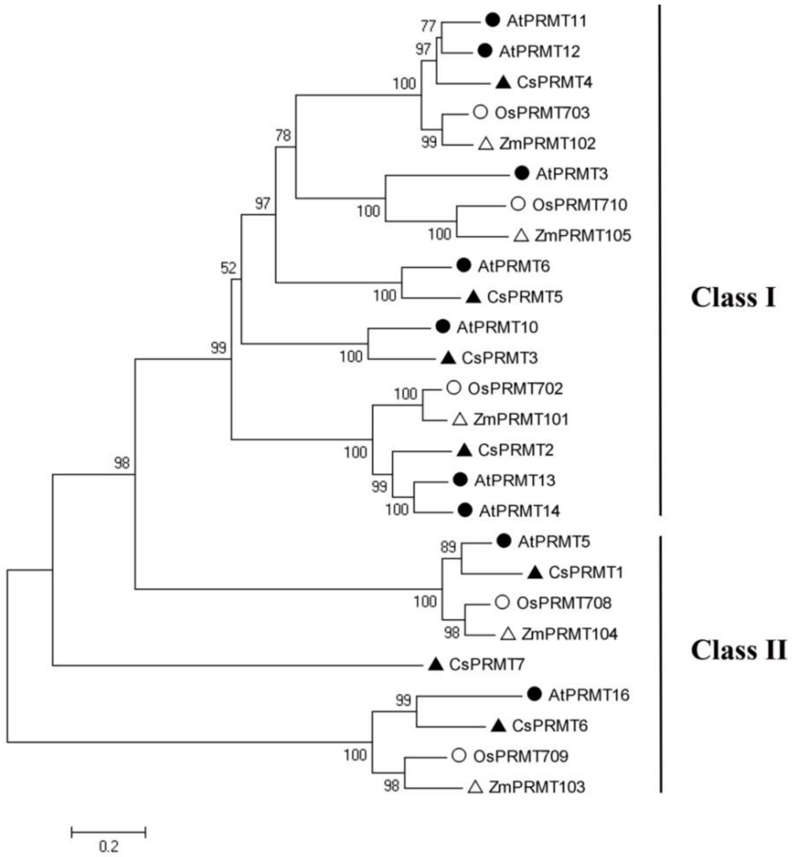

AtPRMT11
AtPRMT12
CsPRMT4
OsPRMT703
ZmPRMT102
AtPRMT3
OsPRMT710
ZmPRMT105
AtPRMT6
CsPRMT5
AtPRMT10
CsPRMT3
OsPRMT702
ZmPRMT101
CsPRMT2
AtPRMT13
AtPRMT14
AtPRMT5
CsPRMT1
OsPRMT708
ZmPRMT104
CsPRMT7
AtPRMT16
CsPRMT6
OsPRMT709
ZmPRMT103
Class I
Class II
0.2


**HDMAs:**

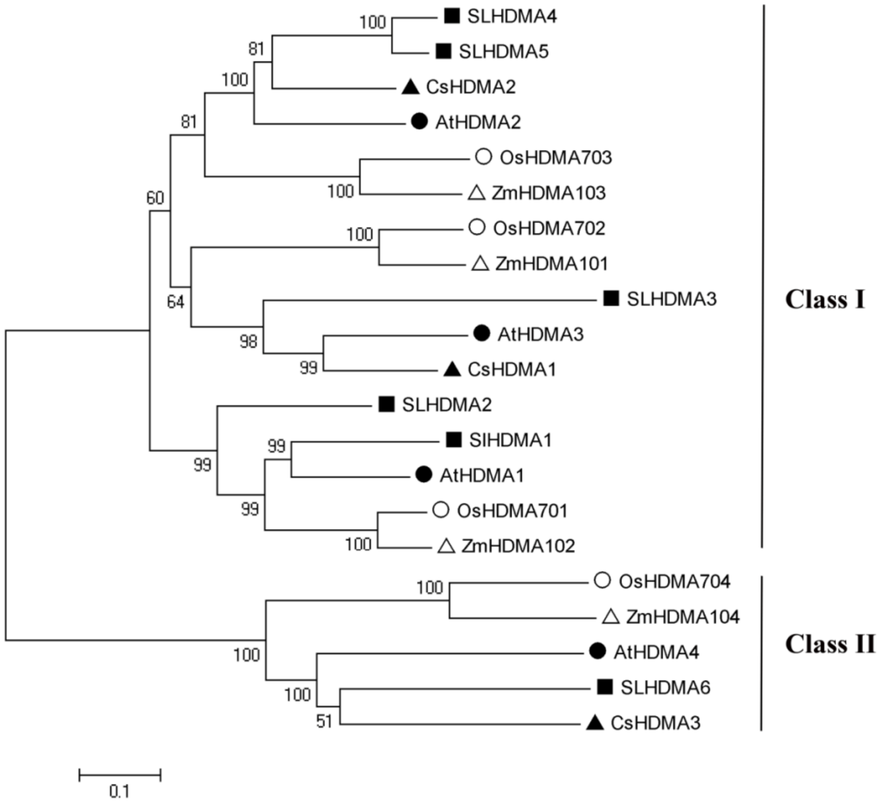

SLHDMA4
SLHDMA5
CsHDMA2
AtHDMA2
OsHDMA703
ZmHDMA103
OsHDMA702
ZmHDMA101
SLHDMA3
AtHDMA3
CsHDMA1
SLHDMA2
SlHDMA1
AtHDMA1
OsHDMA701
ZmHDMA102
OsHDMA704
ZmHDMA104
AtHDMA4
SLHDMA6
CsHDMA3
Class I
Class II
0.1

**HAGs:**

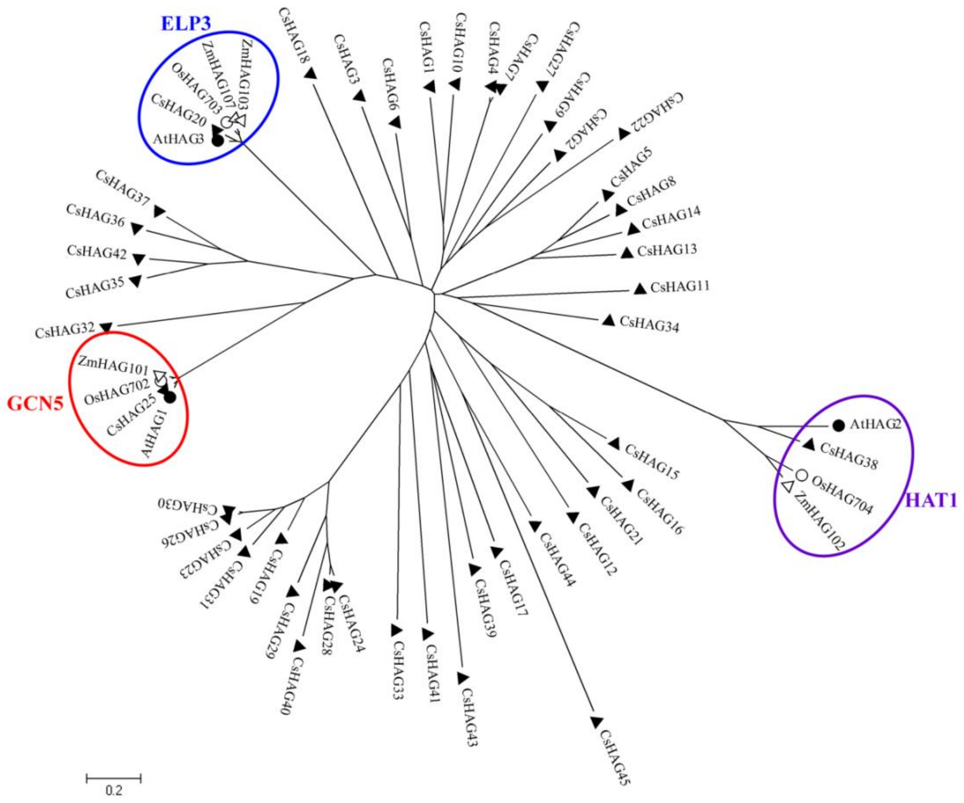


**HAMs:**

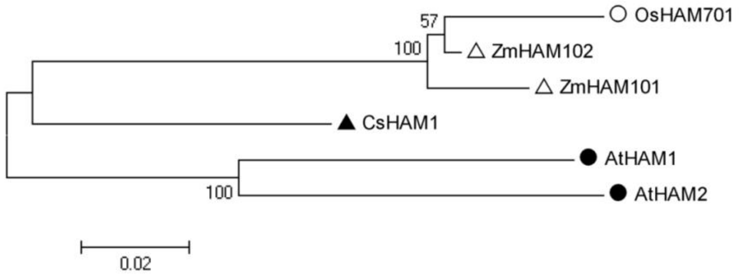


**HACs:**

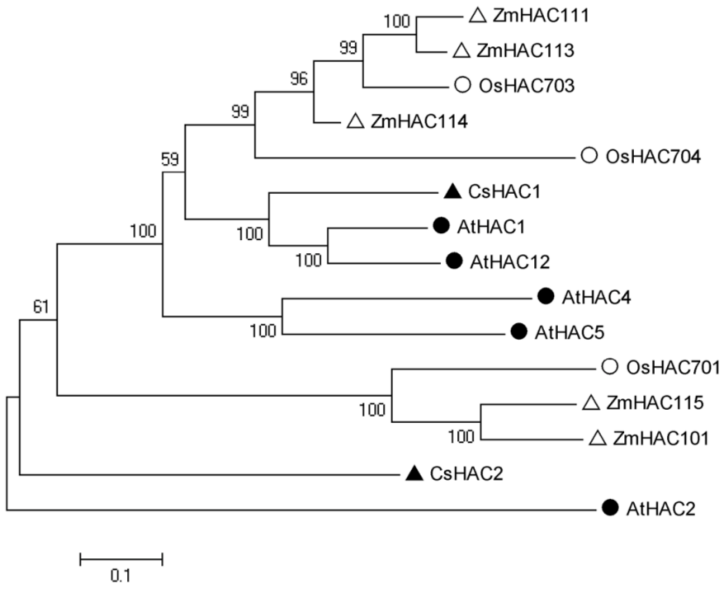

**HAFs:**

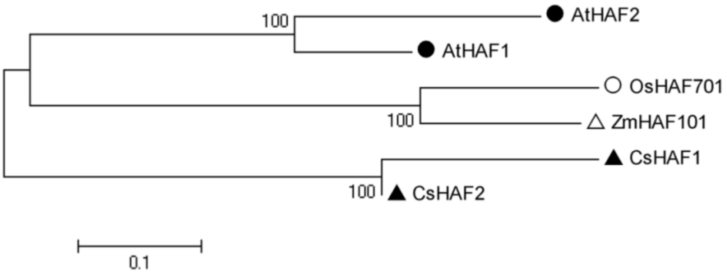


**HDAs:**

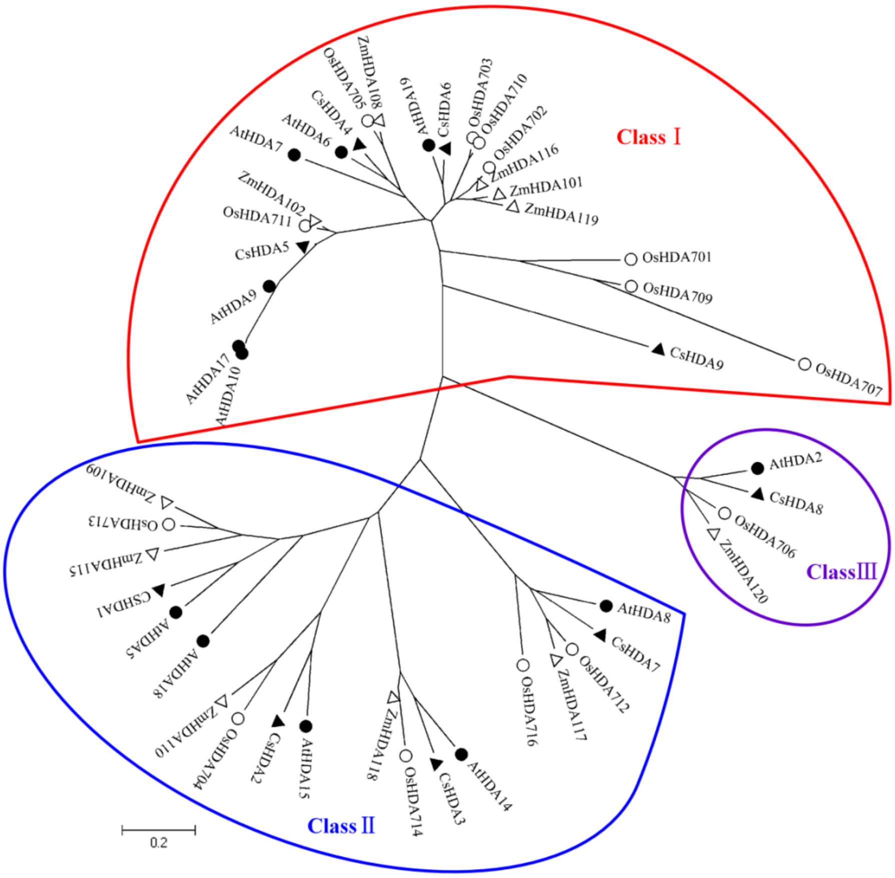


**SRTs:**

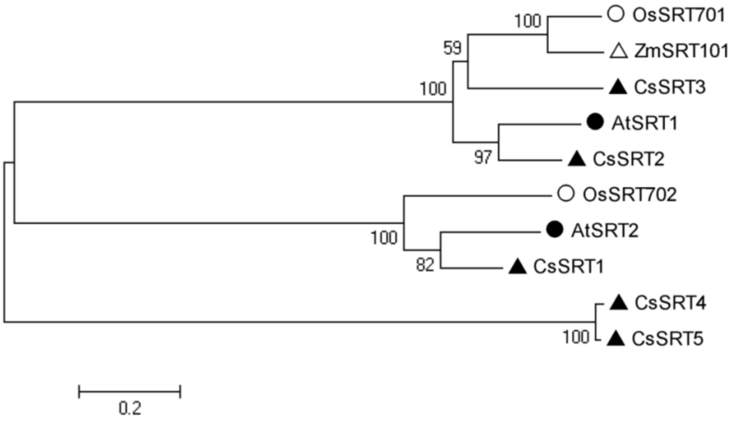

## HDTs:

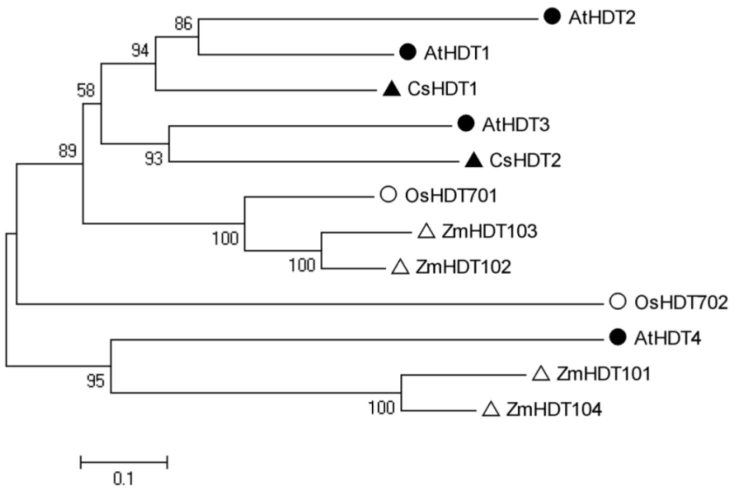


**Supplementary figure 1. Phylogenetic tree of PRMT, HDMA, HAG, HAM, HAC, HAF, HDA, SRT, HDT families.** The protein sequences of *Citrus Sinensis*, *Arabidopsis thaliana*, *Oryza sativa* and *Zea mays* were aligned using ClustalW program and the phylogenetic analysis was performed using MEGA5.05 program with Neighbor-Joining method.
