# Supplementary material for: Genome-wide identification of sweet orange (Citrus sinensis) histone modification gene families and their expression analysis during the fruit development and fruit-blue mold infection process
Source: Front Plant Sci. 2015 Aug 5;6:607. doi: 10.3389/fpls.2015.00607 (PMC4525380; doi:10.3389/fpls.2015.00607)
Supplement: Supplementary file 5 [file Image_2.PDF]

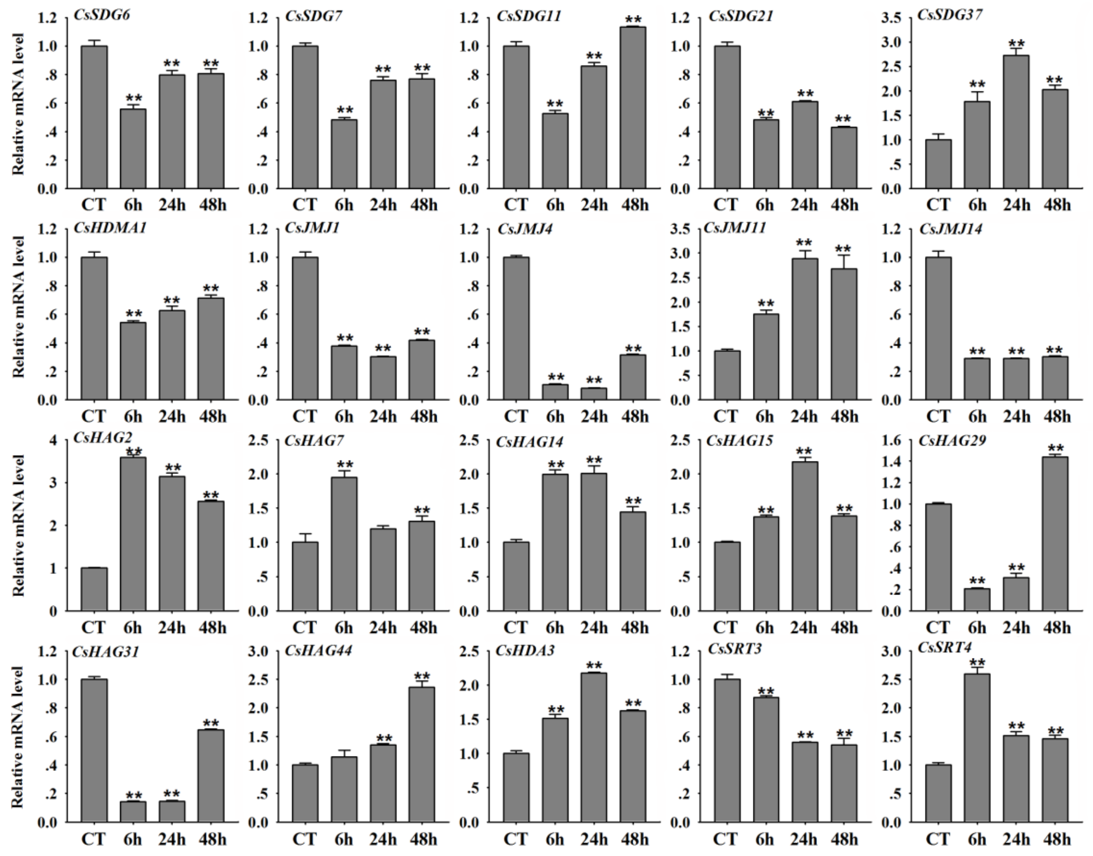

Supplementary figure 2 Expression profiles of 20 selected *CsHMs* (log2 value >1.0 or <-1.0 in Fig. 10) in response to blue mold infection were performed with bar diagram. Data were means ± SD of three separate measurements. ** indicated significant differences at P<0.01.
