# Supplementary material for: Genome-wide identification of sweet orange (Citrus sinensis) histone modification gene families and their expression analysis during the fruit development and fruit-blue mold infection process
Source: Front Plant Sci. 2015 Aug 5;6:607. doi: 10.3389/fpls.2015.00607 (PMC4525380; doi:10.3389/fpls.2015.00607)
Supplement: Supplementary file 6 [file Image_3.PDF]

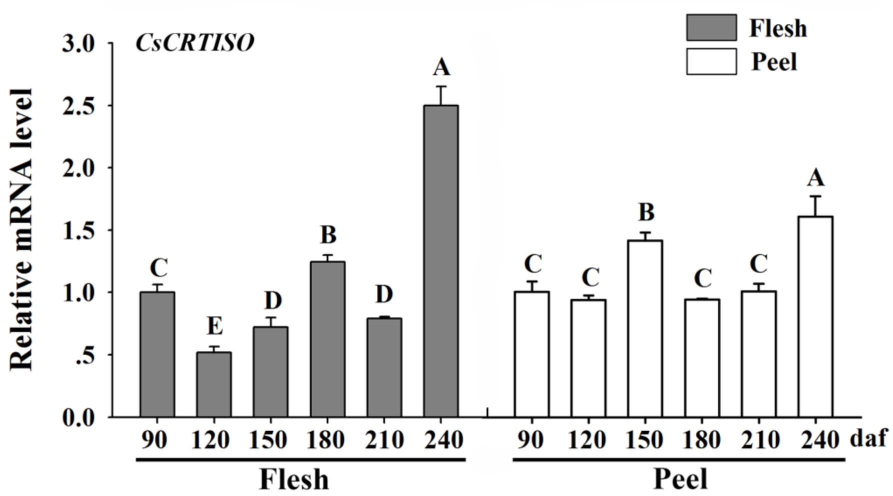

Supplementary figure 3 Expression profiles of *CsCRTISO* in flesh and peel during six fruit developmental stages (90-240 daf-days after flowering). *Capital letters* indicated significant differences at *P*<0.01.
